# Dapagliflozin protects against nonalcoholic steatohepatitis in db/db mice

**DOI:** 10.3389/fphar.2022.934136

**Published:** 2022-08-19

**Authors:** Panshuang Qiao, Yingli Jia, Ang Ma, Jinzhao He, Chen Shao, Xiaowei Li, Shuyuan Wang, Baoxue Yang, Hong Zhou

**Affiliations:** ^1^ Department of Pharmacology and Department of the Integration of Chinese and Western Medicine and Department of Pharmacology, School of Basic Medical Sciences, Peking University, Beijing, China; ^2^ Department of Physiology and Pathophysiology, School of Basic Medical Sciences, Peking University, Key Laboratory of Molecular Cardiovascular Science, Ministry of Education, Beijing, China; ^3^ The Department of Pathology, Beijing You An Hospital, Capital Medical University, Beijing, China

**Keywords:** sodium-glucose cotransporter-2 inhibitor, nonalcoholic fatty liver disease, *de novo* lipogenesis, inflammation, fibrosis

## Abstract

Nonalcoholic fatty liver disease (NAFLD), which is the most common liver disease, is associated with type 2 diabetes mellitus and metabolic syndrome. Although there is no consensus on the treatment of NAFLD, growing evidence suggests that tight glycemic control would contribute to the improvement of NAFLD. However, some insulin sensitizers cannot improve NAFLD, especially nonalcoholic steatohepatitis (NASH). Whether insulin-independent hypoglycemic drug dapagliflozin, a sodium-glucose cotransporter-2 inhibitor, may improve NAFLD keeps unclear. Therefore, 12-week-old male C57BL/6 wild-type and db/db mice were treated with 1 mg/kg dapagliflozin or vehicle for 12 weeks. Dapagliflozin alleviated NASH, manifesting as decreased alanine aminotransferase and NAFLD activity score in db/db mice. Also, dapagliflozin reduced *de novo* lipogenesis by the upregulation of FXR/SHP and downregulation of LXRα/SREBP-1c in the liver of db/db mice. Moreover, dapagliflozin treatment reduced inflammatory response by inhibiting the NF-*κ*B pathway and alleviated fibrosis by restoring the balance between fibrogenesis and fibrolysis in the liver of db/db mice. In summary, dapagliflozin alleviates NASH mostly by reducing lipid accumulation, inflammation, and fibrosis. These findings provide new insights for understanding the protective effect of dapagliflozin in NASH and suggest that dapagliflozin may be used to treat NASH.

## Introduction

Nonalcoholic fatty liver disease (NAFLD) encompasses a broad spectrum of liver disorders, ranging from nonalcoholic fatty liver to nonalcoholic steatohepatitis (NASH) with inflammation and hepatocyte injury (ballooning), which may further progress to cirrhosis and hepatocellular carcinoma (Schuppan and Schattenberg, 2013). The global prevalence of NAFLD is about 25.24% in the general population, and the number of patients with NAFLD is growing rapidly worldwide (Younossi et al., 2016; Chalasani et al., 2017). Correspondingly, liver-related mortality observably increases and liver-related disease is the second or third cause of death among patients with NAFLD (Younossi and Henry, 2016). NAFLD is often associated with impaired fasting glucose and metabolic syndrome (Adams et al., 2005; Younossi et al., 2013). Patients, especially, with histological NASH are at higher risk for cirrhosis and liver-related mortality (GBD 2015 Mortality and Causes of Death Collaborators, 2016; Sayiner et al., 2016). However, there is no licensed and effective pharmacological approach to treat NAFLD, especially NASH, clinically.

Risk factors associated with NAFLD include obesity, type 2 diabetes mellitus (T2DM), hypertension, and dyslipidemia. In fact, the prevalence of NAFLD in patients with T2DM is very high and ranges from one-third to two-thirds (Leite et al., 2009; Prashanth et al., 2009; Byrne and Targher, 2015; Fan et al., 2016). More importantly, NAFLD in turn promotes the progression of T2DM and worsens most chronic complications of diabetes, such as cardiovascular disease and chronic kidney disease (Targher and Byrne, 2013). Because metabolic impairment and poor glycemic control cause insulin resistance and NAFLD, hypoglycemic agents are expected to improve NAFLD (Tilg et al., 2017). Nevertheless, metformin and GLP-1 agonists are not recommended for the treatment of NASH in adult patients (Chalasani et al., 2017). Only several hypoglycemic drugs, such as insulin sensitizer thiazolidinediones, are recommended for the treatment of biopsy-proven NASH by the American Association for the Study of Liver Disease (Chalasani et al., 2017). It is reported that the hypoglycemic agent dapagliflozin combined with metformin or exenatide improves NAFLD in T2DM patients (Choi et al., 2018; Gastaldelli et al., 2019). Dapagliflozin is one of the insulin-independent oral antihyperglycemic medications and lowers blood glucose levels by promoting urinary glucose excretion by inhibiting sodium-glucose cotransporter-2 (SGLT2). It is widely used for the treatment of T2DM as monotherapy or added to metformin, sulfonylurea, or insulin. It significantly reduces hemoglobin A1c (Bailey et al., 2010; Kaku et al., 2014), increases insulin sensitivity and β-cell functions (Merovci et al., 2014; Merovci et al., 2015), decreases body weight, and lowers systolic blood pressure (Musso et al., 2012).

However, it is still uncertain whether dapagliflozin improves NAFLD, especially liver histology. Therefore, further evaluation regarding the long-term effect of dapagliflozin on liver histology and the mechanism is implemented in diabetic db/db mice with NAFLD.

In this study, we used db/db mice with NAFLD to evaluate the long-term action of dapagliflozin on the liver. It shows that dapagliflozin treatment for 12 weeks not only lowered alanine aminotransferase levels and NAFLD activity score but also alleviated histopathological changes in db/db mice.

## Materials and methods

### Animal models

Male C_57_BL/6 wild-type mice and male C_57_BL/6 db/db mice at 12 weeks of age were purchased from the Animal Center of Peking University Health Science Center. Mice were maintained in a standard environment with a 12-h/12-h light/dark cycle at 22 ± 3°C, and the humidity was kept at 50–60%. Mice had free access to water. All animal experiments conformed to the Guide for the Care and Use of Laboratory Animals published by the US National Institutes of Health (NIH Publication, eighth Edition, 2011) and were approved by the Peking University Health Science Center Animal Experimentation Ethics Committee (laboratory animal use license No. XYSK (JING) 2011-0039, laboratory animal production license No. SCXK (JING) 2011-0012).

The wild-type mice and db/db mice were randomly divided into the control group and dapagliflozin-treated group. Mice of the dapagliflozin-treated group were orally administered with 1 mg/kg dapagliflozin (MCE, Shanghai, China, HY-10450) once daily for 12 weeks. We use saline to dissolve dapagliflozin and the dapagliflozin solution is suspended. In the control group, mice received a vehicle in the same manner for 12 weeks. The fasting blood glucose levels of the mice were examined using a glucometer (One Touch UltraEasy, Life Scan, Wayne, PA, United States) after 8 h of fasting. The mice at 24 weeks of age were sacrificed by inhalation of isoflurane. Serum was collected by centrifuging whole blood at 3500 r/min for 15 min. The tissue samples were collected, frozen in liquid nitrogen, and kept at −80°C for further studies. Data were collected from six animals in each group.

### Liver lipid and serum enzyme activity assays

Liver extracts were prepared by a 1:2 chloroform: methanol mixture. The triglyceride (TG) content in the livers of mice was detected using commercial kits (NJJC Bio, Nanjing, Jiangsu, China, F001-1-1). The blood alanine aminotransferase (ALT) level was examined using commercial kits (NJJC Bio, Nanjing, Jiangsu, China, C009).

### Histological analysis

The liver tissues were fixed with 4% paraformaldehyde (Sigma-Aldrich, Saint Louis, MO, United States, 158127) overnight, dehydrated in graded alcohol (Tong Guang, Beijing, China, 104022) and then embedded in paraffin (Leica, Wetzlar, Germany, 39601095) for staining with hematoxylin (Amresco, Radnor, PA, United States, 0701) and eosin (Amresco, Radnor, PA, United States, 0109) to visualize lipid accumulation and hepatic damage in the liver. The NAFLD activity score (NAS) was assessed according to the method described in the study by Kleiner et al. (2005). In brief, NAS was scored in a blinded manner according to the percentage of damages including steatosis, inflammation, and ballooning. The grade of steatosis was defined: 0 = < 5%; 1 = 5–33%; 2 = 33–66%; 3 = > 60%. The grade of lobular inflammation was defined: 0 = 0; 1 = < 2 foci/field; 2 = 2–4 foci/field; 3 = > 5 foci/field. The grade of hepatocyte ballooning was defined: 0 = none; 1 = few ballooned cells; 2 = many ballooned cells. Then, 10 random pictures per liver section were quantified.

For oil-red O staining, liver samples were embedded in Tissue-Tek OCT embedding compound (SAKURA, Tokyo, Japan, 4583), and frozen on dry ice. Moreover, 7 μm-thick frozen sections were cut and stained with an oil-red O (Sigma-Aldrich, Saint Louis, MO, United States, O0625-25G).

For ultrastructural evaluation, liver samples were fixed in 2.5% glutaraldehyde, postfixed in osmium tetroxide, and stained with uranyl acetate and lead citrate. The specimen was thin-sectioned and examined under a transmission electron microscope. Electron microscopic pictures were randomly taken in each group.

### Immunohistochemistry

Paraformaldehyde fixed and paraffin-embedded liver tissue slices were deparaffinized and rehydrated. Hydrogen peroxide blocking was performed with 3% H_2_O_2_ for 10 min. Antigen retrieval was performed in a steamer for 15 min. The slides were blocked with goat serum for 30 min and liver sections were incubated with the following antibodies: fatty acid synthase (FAS) (Cell Signaling Technology, Danvers, MA, 3180, 1:1000 dilution), acetyl-CoA carboxylase (ACC) (Sigma-Aldrich, Saint Louis, MO, United States, 05-1098, 1:1000 dilution), carbohydrate responsive element binding protein (ChREBP) (ABclonal, Wuhan, China, A7630, 1:1000 dilution), p-nuclear factor kappa B (NF-*κ*B) p65 (Bioworld, Nanjing, China, bs1253, 1:1000 dilution), α-smooth muscle actin (α-SMA) (ABclonal, Wuhan, China, A7248, 1:1000 dilution), fibronectin (Abcam, Cambridge, UK, ab45688, 1:1000 dilution), and tissue inhibitor of metalloproteinases (TIMP)-1 (Abcam, Cambridge, UK, ab38978, 1:1000 dilution). After incubating with the primary antibody overnight at 4°C, liver sections were incubated with the goat antirabbit IgG secondary antibody (Immunoway, Suzhou, China, RS0002) for 30 min. Liver tissue slices were washed and then mounted with hematoxylin. Immunohistochemistry for F4/80 (Abcam, Cambridge, UK, ab60343, 1:100 dilution) was also performed on paraffin-embedded liver sections. Images were captured and recorded by a microscope (Olympus, Tokyo, Japan) and 10 random pictures per liver section were quantified. The integrated optical densities (IODs) or numbers of F4/80 positive cells of different indices were calculated by Image-Pro Plus software 6.0 (National Institutes of Health, Bethesda, MD, United States).

### Western blotting analysis

For the western blotting analysis, total protein samples were isolated from liver samples using RIPA lysis buffer (Applygen, Beijing, China, C1053) containing a 4% protease inhibitor cocktail (Roche, South San Francisco, CA, United States, 11873580001) and 1% protein phosphatase inhibitor (Applygen, Beijing, China, P1260). Protein concentrations were examined using a Pierce BCA Protein Assay Kit (Thermo Fisher Scientific, Shanghai, China, 23225). The obtained protein was separated through SDS-polyacrylamide gel electrophoresis, transferred to polyvinylidene difluoride membranes (PALL, Beijing, China, BSP0161), followed by blocking with 5% nonfat milk or 2% bovine serum albumin, incubation with primary antibody overnight at 4°C. The primary antibodies were ACC (Sigma-Aldrich, Saint Louis, MO, United States, 05-1098, 1:1000 dilution), FAS (Cell Signaling Technology, Danvers, MA, 3180, 1:1000 dilution), carnitine palmitoyltransferase 1a (CPT1a) (ABclonal, Wuhan, China, A5307, 1:1000 dilution), farnesoid X receptor (FXR, Huaxingbio, Beijing, China, HX19905, 1:1000 dilution), small heterodimer partner (SHP, ABclonal, Wuhan, China, A1836, 1:1000 dilution), liver X receptor α (LXRα, Huaxingbio, Beijing, China, HX15716, 1:1000 dilution), sterol regulatory element binding transcription factor (SREBP)-1c (Novus, Shanghai, China, NB600-582, 1:1000 dilution), ChREBP (ABclonal, Wuhan, China, A7630, 1:1000 dilution), glucose transporter (GLUT) 1 (Sigma-Aldrich, Saint Louis, MO, United States, 07-1401, 1:1000 dilution), GLUT2 (Abcam, Cambridge, UK, ab54460, 1:1000 dilution), p-ERK (Santa Cruz, Dallas, TX, United States, sc-7383, 1:1000 dilution), ERK2 (Santa Cruz, Dallas, TX, United States, sc-154, 1:1000 dilution), p-p38 (Cell Signaling Technology, Danvers, MA, 9215, 1:1000 dilution), p38 (Cell Signaling Technology, Danvers, MA, 9212, 1:1000 dilution), p-JNK (Santa Cruz, Dallas, TX, United States, sc-6254, 1:1000 dilution), JNK2 (ABclonal, Wuhan, China, A1251, 1:1000 dilution), p-NF-κB p65 (Abcam, Cambridge, UK, ab86299, 1:1000 dilution), NF-*κ*B p65 (Bioworld, Nanjing, China, bs1253, 1:1000 dilution), cyclooxygenase (COX)-2 (Bioworld, Nanjing, China, bs1076, 1:1000 dilution), p-JAK1 (Bioworld, Nanjing, China, bs4108, 1:1000 dilution), JAK1 (Bioworld, Nanjing, China, bs1193, 1:1000 dilution), p-STAT3 (Cell Signaling Technology, Danvers, MA, 9145, 1:1000 dilution), STAT3 (Cell Signaling Technology, Danvers, MA, 9132, 1:1000 dilution), transforming growth factor (TGF)-β1 (Santa Cruz, Dallas, TX, United States, sc-146, 1:1000 dilution), connective tissue growth factor (CTGF) (Santa Cruz, Dallas, TX, United States, sc-14939, 1:1000 dilution), plasminogen activator inhibitor (PAI)-1 (ABclonal, Wuhan, China, A6211, 1:1000 dilution), TIMP-1 (Abcam, Cambridge, UK, ab38978, 1:1000 dilution), metalloproteinase-9 (MMP9, Abcam, Cambridge, UK, ab38898, 1:1000 dilution), fibronectin (Abcam, Cambridge, UK, ab45688, 1:1000 dilution), and α-SMA (ABclonal, Wuhan, China, A7248, 1:1000 dilution). Goat antirabbit IgG or goat antimouse IgG (Easybio, Beijing, China, BE0102) were added at room temperature for 1 h and the blots were developed with ECL plus kit (Meilunbio, Dalian, China, MA0186). Quantitation was performed by scanning and analyzing the intensity of the hybridization band. β-Actin was served as a loading control.

### Statistical analysis

All data were analyzed using SPSS (version 19.0) and were shown as the means ± standard error (SEM). To examine the significant differences between groups, the data were compared using a one-way analysis of variance (ANOVA), followed by Bonferroni’s post hoc tests (assuming equal variances). Nonparametric analysis (Kruskal–Wallis one-way ANOVA on ranks plus Dunn’s multiple comparison test) was used to assess the significance of semi-quantitative measures (that is, histologic scoring). *p* < 0.05 was considered significant.

## Results

### Dapagliflozin ameliorated NASH in db/db mice

**FIGURE 1:**
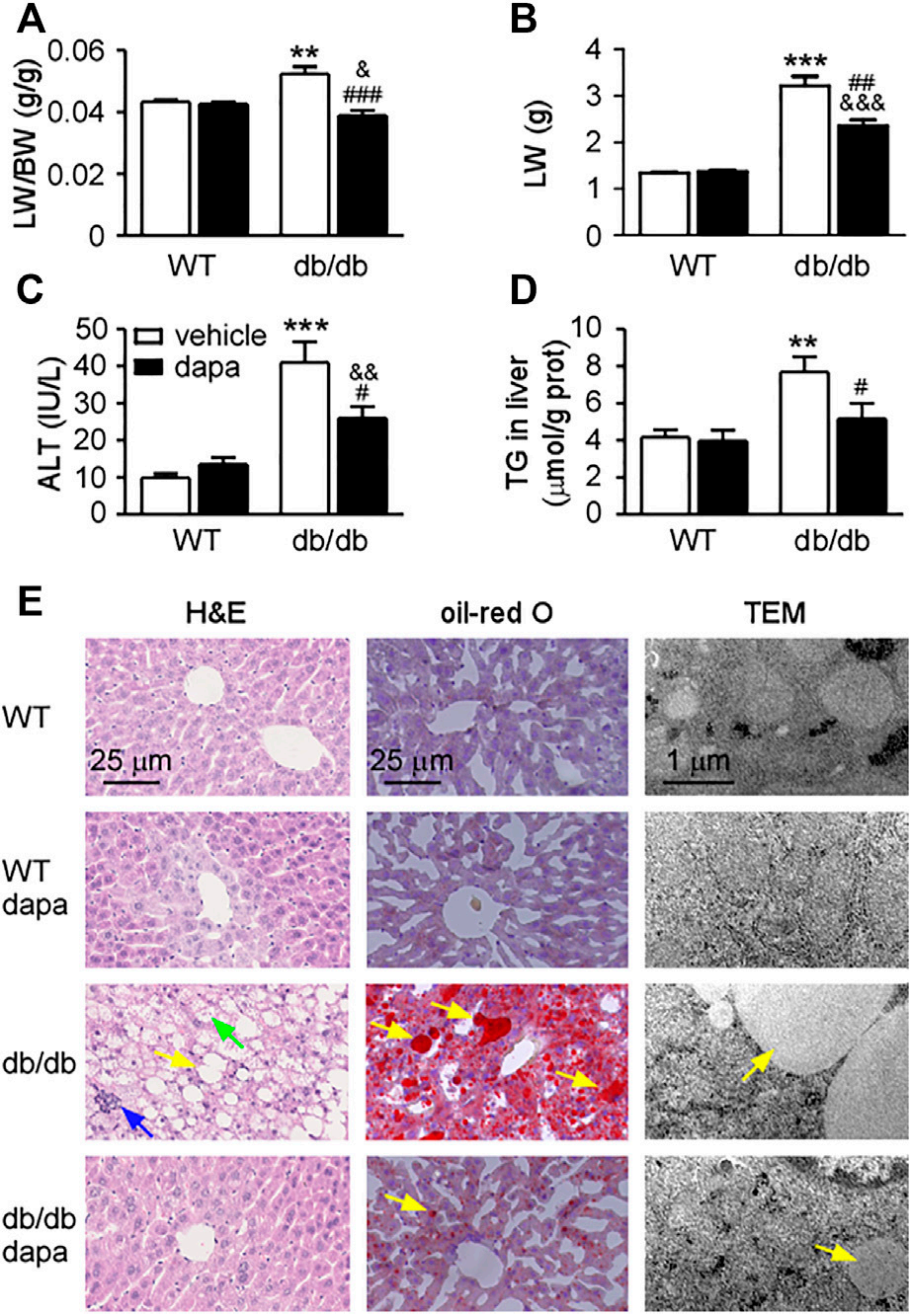
Dapagliflozin ameliorated hepatic damage in db/db mice. **(A)** The ratio of liver weight to body weight (LW/BW). **(B)** Liver weight (LW). **(C)** Blood alanine transaminase (ALT). **(D)** Triglyceride (TG) in livers. **(E)** Liver tissue images. Hematoxylin and eosin (H&E, up), oil-red O staining (middle), and transmission electron microscope (TEM, down). Yellow arrows represent steatosis, blue arrows represent lobular inflammation, and green arrows represent hepatocellular ballooning. Mean ± SEM, n = 6. *p < 0.05, **p < 0.01, and ***p < 0.001 *vs.* wild-type (WT) mice. ^#^ *p* < 0.05, ^##^ *p* < 0.01, and ^###^ *p* < 0.001 *vs.* db/db mice. ^&^ *p* < 0.05, ^&&^ *p* < 0.01, and ^&&&^ *p* < 0.001 *vs.* dapagliflozin-treated wild-type mice. One-way ANOVA, Bonferroni’s multiple comparison test.

To investigate the effect of dapagliflozin on livers of db/db mice, the hepatic function and morphology were detected. In our study, db/db mice showed increased body weight (Supplementary Figure S1A), and fasting blood glucose (Supplementary Figure S1B) compared with wild-type mice. Dapagliflozin treatment did not affect body weight, but significantly decreased fasting blood glucose levels in db/db mice. The ratio of liver weight to body weight (Figure 1A), liver weight (Figure 1B), serum ALT level (Figure 1C), and hepatic TG content (Figure 1D) significantly increased in db/db mice, indicating that hepatic function was impaired in db/db mice. Dapagliflozin treatment significantly decreased the ratio of liver weight to body weight, liver weight, serum ALT level, as a proxy of necroinflammatory activity, and hepatic TG content, showing that hepatic function was improved after dapagliflozin administration in db/db mice.

There are also pathological changes (Figure 1E) and increased NAFLD activity score (NAS) (Table 1) in the liver of db/db mice, including steatosis, lobular inflammation, and hepatocellular ballooning. These results indicate that hepatic damage in db/db mice had progressed to NASH. The steatosis grade was significantly lower in dapagliflozin-treated mice than that in db/db mice (Table 1). The livers of dapagliflozin-treated mice had fewer and smaller lipid droplets around the central veins compared with db/db mice (Figure 1E *top* and *middle*). Electron microscopic images revealed that a lot of lipid droplets were present in the hepatocytes of db/db mice, while lipid droplets were decreased in dapagliflozin-treated mice (Figure 1E *bottom*). Electron microscopy also shows that mitochondrial swelling was present in hepatocytes of db/db mice, which was reduced after dapagliflozin administration (Figure 1E). Lobular inflammation and hepatocellular ballooning grade were also reduced after dapagliflozin administration in db/db mice (Table 1). In addition, dapagliflozin had no effect on hepatic function and morphology in wild-type mice (Figure 1 and Table 1).

**TABLE 1:** NAFLD activity score (NAS).

	WT	WT dapa	db/db	db/db dapa
Steatosis	0	0	1.225 ± 0.160***	0.175 ± 0.048^### &^
Lobular inflammation	0.125 ± 0.048	0.15 ± 0.065	0.400 ± 0.087*	0.100 ± 0.058^#^
Ballooning	0	0.050 ± 0.050	2	1.250 ± 0.218^# &&^
NAS	0.150 ± 0.050	0.200 ± 0.071	3.67 ± 0.236***	1.525 ± 0.197^### &&&^

Values were mean ± SEM, *n* = 4. *p < 0.05, **p < 0.01, and ***p < 0.001 *vs.* wild-type mice (WT). ^#^ *p* < 0.05, ^##^ *p* < 0.01, and ###*p* < 0.001 *vs.* db/db mice. ^&^ *p* < 0.05, ^&&^ *p* < 0.01, and ^&&&^ *p* < 0.001 *vs.* dapagliflozin-treated wild-type mice. One-way ANOVA, Bonferroni’s multiple comparison test. Dapa = dapagliflozin.

### Dapagliflozin reduced hepatic lipid accumulation through suppressing *de novo* lipogenesis in the liver of db/db mice

**FIGURE 2:**
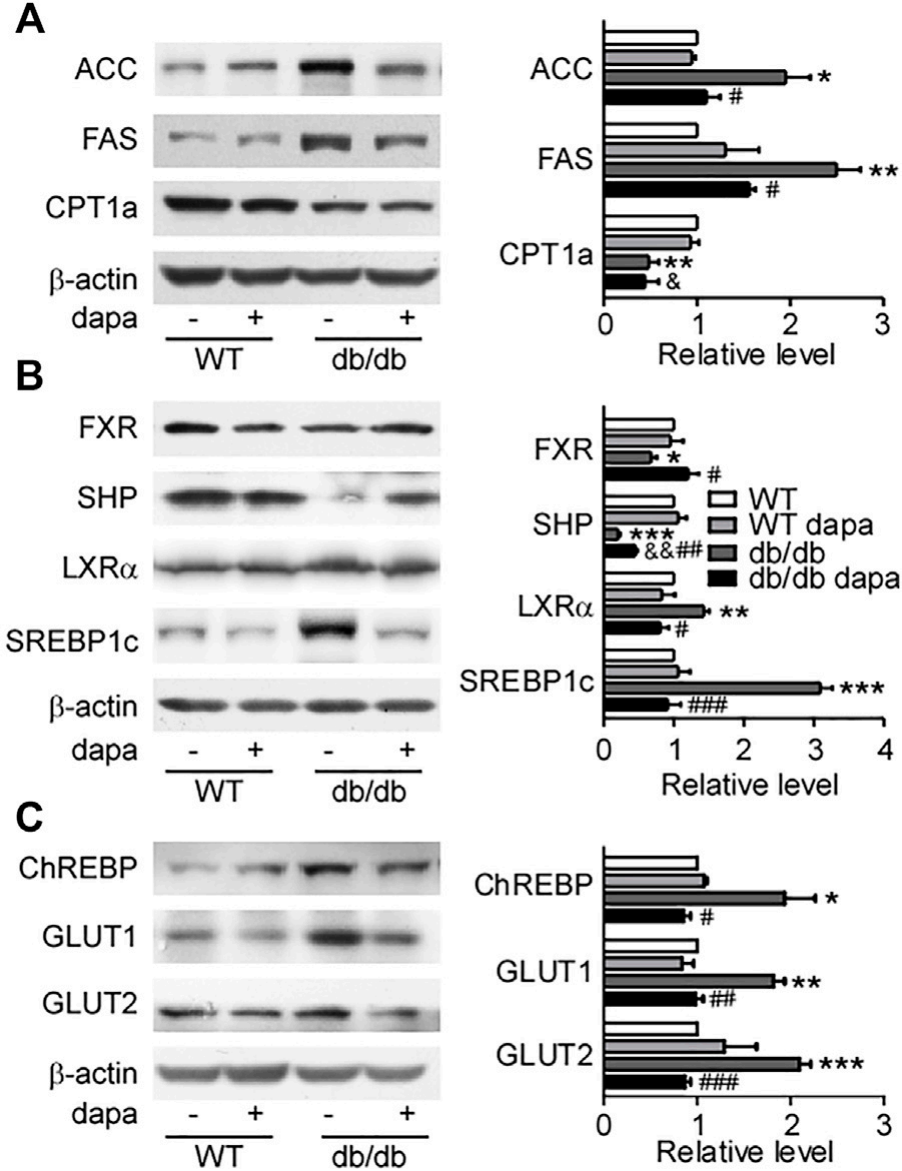
Dapagliflozin improved hepatic fat metabolism in db/db mice. **(A)** Representative western blotting (*left*) and quantification of protein levels (*right*) of fat acid metabolism-related proteins. **(B)** Representative western blotting (*left*) and quantification of protein levels (*right*) of SREBP-1c pathway-related proteins of livers. **(C)** Representative western blotting (*left*) and quantification of protein levels (*right*) of ChREBP, GLUT1, and GLUT2 of livers. Values were means ± SEM, n = 3. *p < 0.05, **p < 0.01, and ***p < 0.001 *vs.* wild-type mice (WT). ^#^ *p* < 0.05, ^##^ *p* < 0.01, and ^###^ *p* < 0.001 *vs.* db/db mice. ^&^ *p* < 0.05 and ^&&^ *p* < 0.01 *vs.* dapagliflozin-treated wild-type mice. One-way ANOVA, Bonferroni’s multiple comparison test. SREBP-1c = sterol regulatory element binding transcription factor; ChREBP1 = carbohydrate responsive element binding protein; GLUT1 = glucose transporter 1; GLUT2 = glucose transporter 2.

**FIGURE 3:**
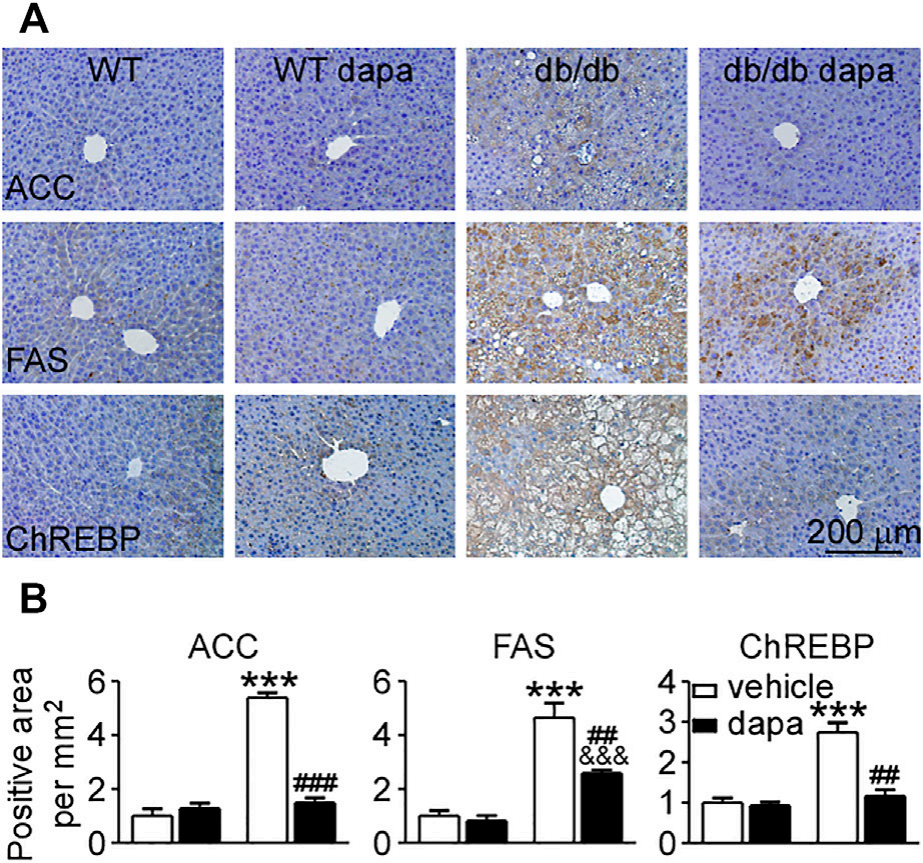
Dapagliflozin inhibited DNL in the liver of db/db mice. **(A)** Immunohistochemistry of ACC, FAS, and ChREBP in liver tissues. **(B)** The relative level of integrated optical densities (IODs) of ACC, FAS, and ChREBP. Values were mean ± SEM, n = 3. *p < 0.05, **p < 0.01, and ***p < 0.001 *vs.* wild-type (WT) mice. ^#^ *p* < 0.05, ^##^ *p* < 0.01, and ^###^ *p* < 0.01 *vs.* db/db mice. ^&^ *p* < 0.05, ^&&^ *p* < 0.01, and ^&&&^ *p* < 0.001 *vs.* dapagliflozin-treated wild-type mice. One-way ANOVA, Bonferroni’s multiple comparison test. DNL = *de novo* lipogenesis; FAS = fatty acid synthase; ACC = acetyl-CoA carboxylase; ChREBP1 = carbohydrate responsive element binding protein.

To determine how dapagliflozin reduces lipid accumulation in the liver of db/db mice, the expression levels of proteins related to lipid synthesis and degradation in the liver were analyzed by western blotting analysis (Figure 2) and immunohistochemistry (Figure 3). The enzymes involved in the *de novo* lipogenesis (DNL) such as FAS and ACC, were significantly increased in the liver of db/db mice (Figures 2A, 3A). CPT1a, a key regulatory factor in β-oxidation, was significantly decreased in the liver of db/db mice (Figure 2A). Moreover, nuclear transcription factors related to DNL including FXR and SHP were significantly downregulated and LXRα and SREBP-1c were significantly upregulated in the liver of db/db mice (Figure 2B). In addition, increased ChREBP expression was followed by increased expression of GLUT1 and GLUT2, indicating that hepatic glucose output was increased in the liver of db/db mice (Figures 2C, 3A). Treatment with dapagliflozin significantly decreased the expression of FAS, ACC, LXRα, SREBP-1c, ChREBP, GLUT1, and GLUT2 and increased the expression of FXR and SHP (Figures 2, 3). Dapagliflozin treatment also significantly decreased fasting blood glucose levels in db/db mice (Supplementary Figure S1B).

### Dapagliflozin ameliorated inflammation through inhibiting the NF-*κ*B pathway in the liver of db/db mice

**FIGURE 4:**
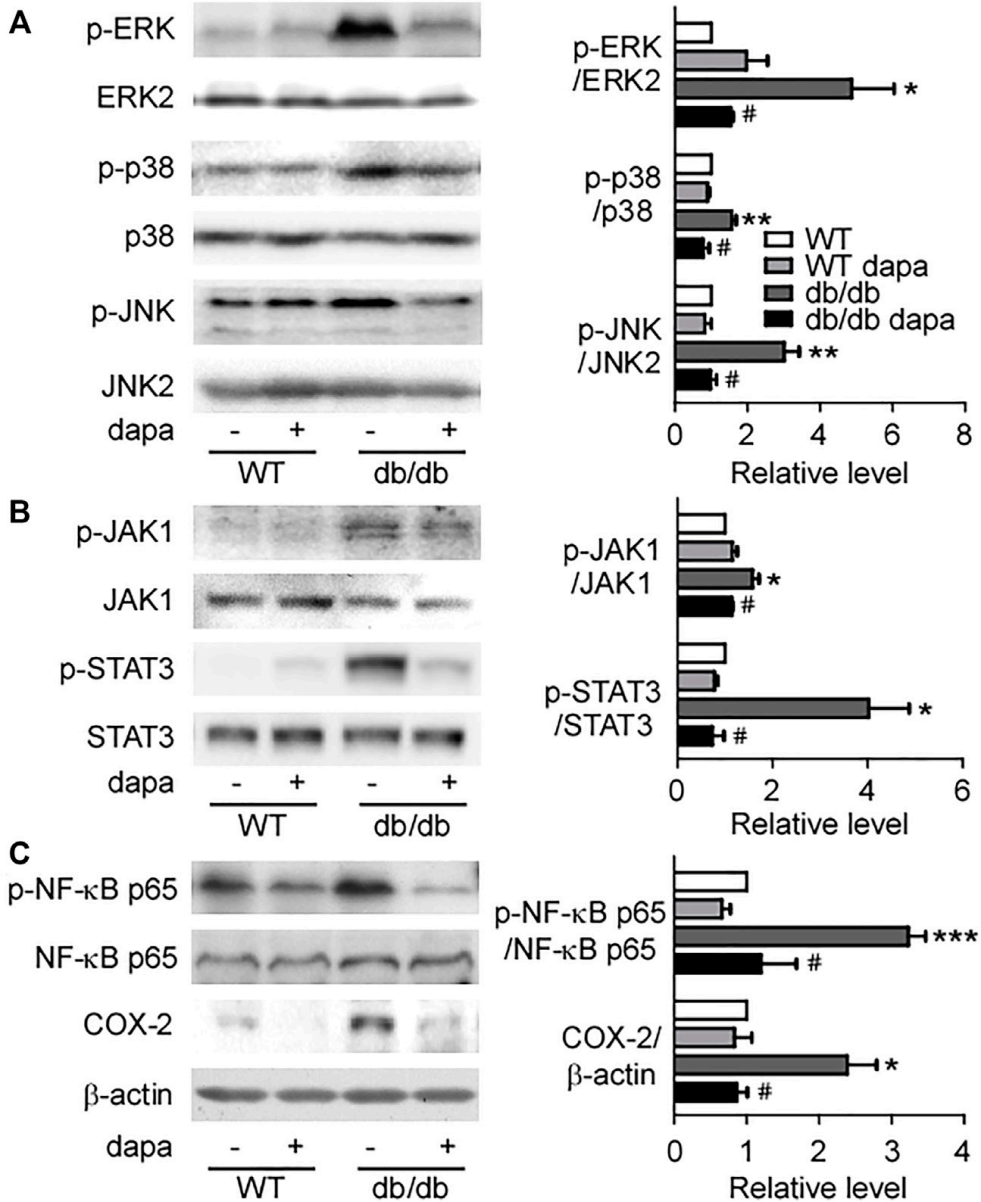
Dapagliflozin inhibited hepatic inflammatory response in db/db mice. **(A)** Representative western blotting (*left*) and quantification of protein levels (*right*) of ERK, p38, and JNK of livers. **(B)** Representative western blotting (*left*) and quantification of protein levels (*right*) of JAK1 and STAT3 of livers. **(C)** Representative western blotting (*left*) and quantification of protein levels (*right*) of NF-κB p65 and COX-2 of livers. Values were mean ± SEM, n = 3. *p < 0.05, **p < 0.01, and ***p < 0.001 *vs.* wild-type (WT) mice. ^#^ *p* < 0.05 *vs.* db/db mice. One-way ANOVA, Bonferroni’s multiple comparison test. ERK = extracellular regulated kinases; JNK = c-Jun N-terminal kinase; JAK1 = Janus-activated kinase; STAT3 = signal transducer and activator of transcription 3; NF-κB p65 = nuclear factor kappa B p65; COX-2 = cyclooxygenase-2.

**FIGURE 5:**
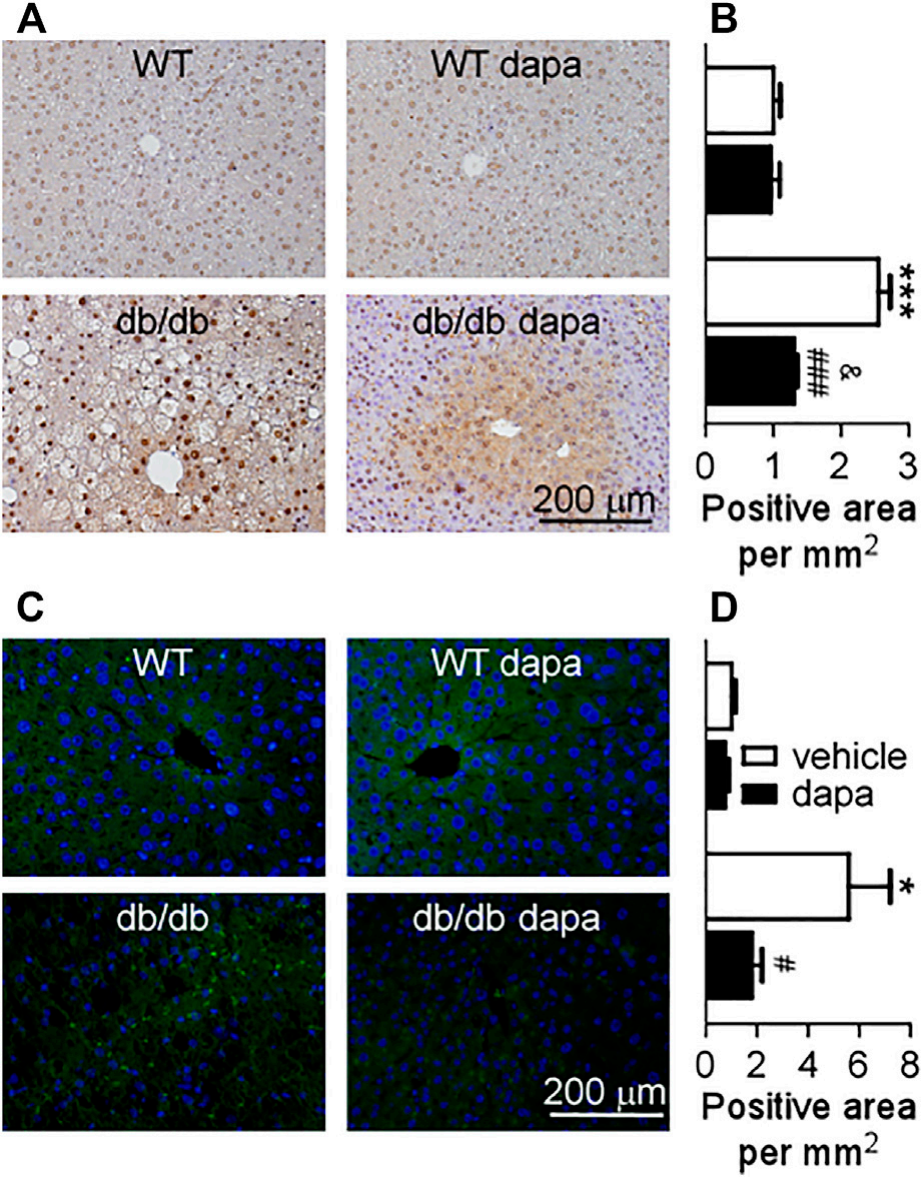
Dapagliflozin inhibited the expression of p-NF-*κ*B p65 and infiltration of immune cells (F4/80^+^) in the liver of db/db mice. **(A)** Immunohistochemistry of p-NF-*κ*B p65 in livers. **(B)** The relative level of integrated optical densities (IODs) of p-NF-*κ*B p65 in livers. **(C)** Infiltration of immune cells (F4/80^+^) assessed by immunohistochemistry in the liver. **(D)** The relative level of the number of F4/80^+^ cells per mm^2^ in the liver. Values were means ± SEM, n = 3. *p < 0.05, **p < 0.01, and ***p < 0.001 *vs.* wild-type (WT) mice. ^#^ *p* < 0.05, ^##^ *p* < 0.01, and ^###^ *p* < 0.001 *vs.* db/db mice. ^&^ *p* < 0.05 *vs.* dapagliflozin-treated wild-type mice. One-way ANOVA, Bonferroni’s multiple comparison test. p-NF-κB p65 = p-nuclear factor kappa B p65.

Inflammation has been recognized as a pivotal driver of NAFLD development. The mitogen-activated protein kinase (MAPK)/NF-*κ*B signaling pathway plays a key role in cellular inflammatory response regulation (Zhang et al., 2017). Here, we first detected ERK, p38, and JNK proteins in the liver and found that phosphorylation levels of ERK, p38, and JNK were markedly increased in db/db mice. Dapagliflozin treatment suppressed the phosphorylation levels of these three proteins (Figure 4A). The phosphorylation levels of JAK1 and STAT3 remarkably increased in db/db mice, which were significantly decreased by dapagliflozin treatment (Figure 4B). It was reported that STAT3 has a critical role in retaining NF-*κ*B in the nucleus and p-STAT3 inhibition reduces NF-kB transcriptional activity (Yang et al., 2007; Kaffe et al., 2017). In line with these results, the NF-*κ*B pathway was activated with an increased phosphorylation level of the NF-*κ*B p65 subunit in the liver of db/db mice (Figure 4C). Dapagliflozin treatment significantly lowered phosphorylation of the NF-*κ*B p65 subunit in db/db mice (Figures 4C, 5C). Moreover, the expression level of COX-2, the downstream molecule regulated by NF-*κ*B, was markedly elevated in the liver of db/db mice, which was inhibited significantly by dapagliflozin treatment (Figure 4C). Correspondingly, the ratio of infiltrated F4/80 positive cells into the liver tissues was significantly exacerbated in db/db mice, whereas dapagliflozin inhibited F4/80 positive cell recruitment in the liver (Figure 5B).

### Dapagliflozin ameliorated fibrosis in the liver of db/db mice

**FIGURE 6:**
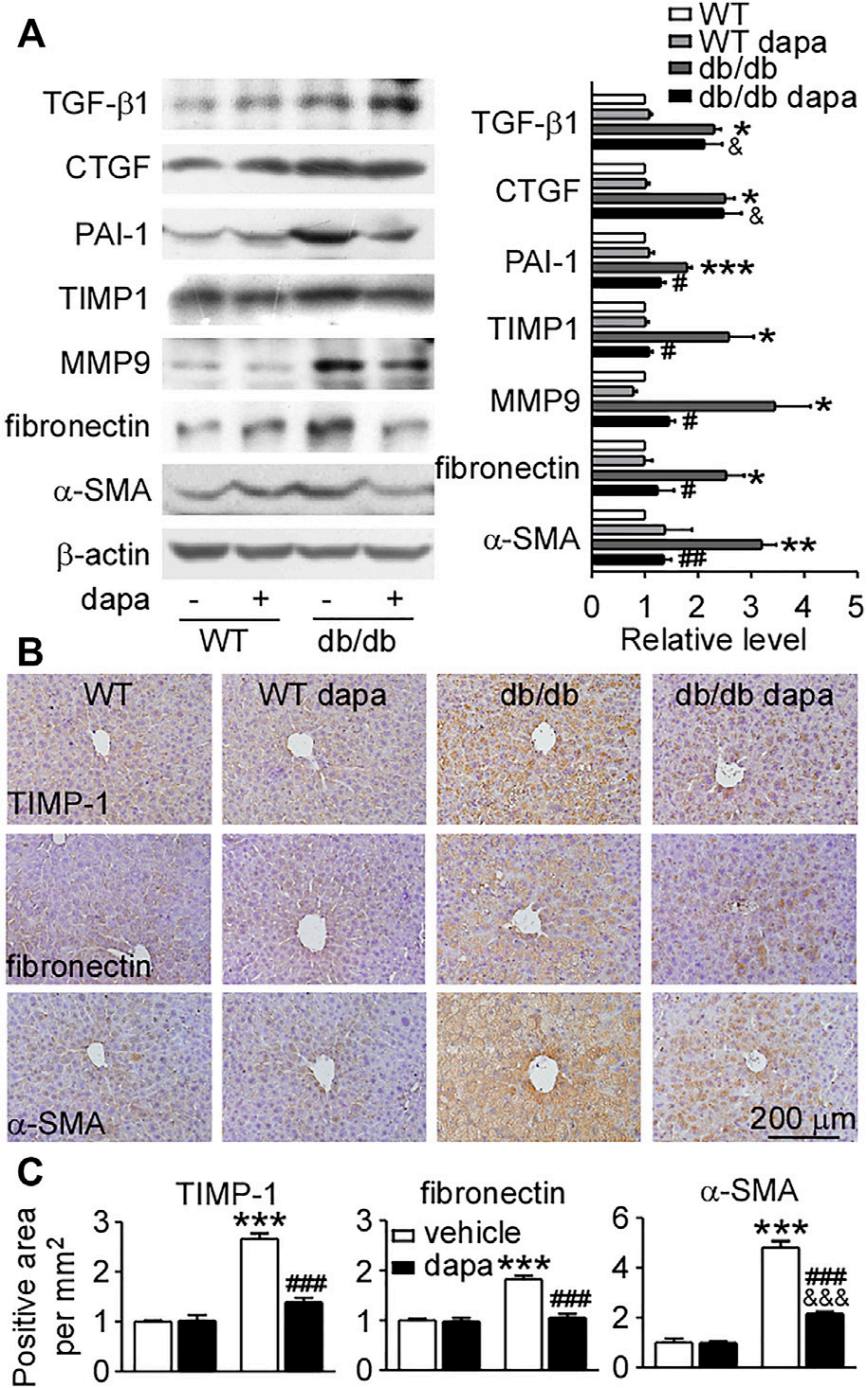
Dapagliflozin protected against fibrosis in the liver of db/db mice. **(A)** Representative western blotting (*left*) and quantification of the expression levels (*right*) of proteins associated with fibrosis in livers. **(B)** Immunohistochemistry showing the expression of TIMP-1, fibronectin, and α-SMA in livers. **(C)** Relative level of integrated optical densities (IODs) of TIMP-1, fibronectin, and α-SMA. Values were mean ± SEM, n = 3. *p < 0.05, **p < 0.01, and ***p < 0.001 *vs.* wild-type (WT) mice. ^#^ *p* < 0.05, ^##^ *p* < 0.01, and ^###^ *p* < 0.01 *vs.* db/db mice. ^&^ *p* < 0.05, ^&&^ *p* < 0.01, and ^&&&^ *p* < 0.001 *vs.* dapagliflozin-treated wild-type mice. One-way ANOVA, Bonferroni’s multiple comparison test. TIMP-1 = tissue inhibitor of metalloproteinase-1; α-SMA = α-smooth muscle actin.

We further investigated the preventive effect of dapagliflozin on the expression of pro-fibrotic factors and extracellular matrix accumulation. The expression levels of pro-fibrotic factors TGF-β1 and CTGF, and fibrolysis-related proteins PAI-1, TIMP-1, and MMP9 increased in the liver of db/db mice (Figures 6A,B). Dapagliflozin had no effect on the upregulation of TGF-β1 and CTGF, but remarkably reduced the expression of PAI-1, TIMP-1, and MMP9 (Figures 6A,B). The anti-fibrotic effect was further supported by decreased expression levels of extracellular matrix accumulation fibronectin and α-SMA in dapagliflozin-treated db/db mice (Figures 6A,B).

## Discussion

The motivation of this study was to determine whether dapagliflozin could improve NASH db/db mice, which are leptin receptor deficient and have obesity, hyperglycemia, hyperinsulinemia, insulin resistance, and fatty liver, widely used as an animal model of NAFLD (Santhekadur et al., 2018). In the present study, we found that NAFLD in db/db mice had progressed to NASH, manifesting as severe steatosis, lobular inflammation, ballooning and fibrosis, and increased levels of ALT. Surprisingly, dapagliflozin not only lowered blood ALT level, liver weight, and TG content in the liver but also reduced NAFLD activity score, indicating that it can alleviate NASH in db/db mice (Figure 1 and Table 1).

The initial hallmark of NAFLD is the excessive accumulation of fat in hepatocytes without alcohol consumption. Several reports have shown that hepatic lipid accumulation leads to cellular injury and death (Anderson and Borlak, 2008; Tetri et al., 2008; Brunt, 2009). Hepatic lipid content increased when the hepatic fatty acid uptake and DNL is more than the fatty acid oxidation and export (Fabbrini et al., 2010). It is estimated that 30% of the TG content in NAFLD came from DNL (Postic and Girard, 2008). Hepatic DNL can be stimulated by insulin via SREBP-1c and by glucose via ChREBP (Chen et al., 2004; Roden, 2006; Musso et al., 2009). The expression level of SREBP-1c increased, which was significantly decreased by dapagliflozin treatment in the liver of db/db mice. We also found that both overexpression of LXRα and downregulation of FXR and SHP were inhibited by dapagliflozin in the liver of db/db mice. FXR is an important regulator of hepatic lipid metabolism through promoting TG clearance and fatty acid β-oxidation concomitantly with a reduction of lipogenic gene transcription (Zhang et al., 2004; Kalaany and Mangelsdorf, 2006). SHP can be strongly induced by FXR, and then inhibits the activity of the LXRα to interfere with SREBP-1c expression, further reducing hepatic lipogenesis (Musso et al., 2009). Therefore, dapagliflozin treatment notably decreased hepatic lipogenesis by increasing the expression of FXR and SHP, further reducing the expression of LXRα and SREBP-1c, and then inhibiting FAS and ACC in the liver of db/db mice.

ChREBP is activated and upregulated by glucose to promote the expression of lipogenic enzymes (Dentin et al., 2004; Ishii et al., 2004). In db/db mice, dapagliflozin not only alleviated hyperglycemia but also lowered the expression of GLUT1 and GLUT2, showing that glucose output in hepatocytes was reduced by dapagliflozin. ChREBP overexpression induced by glucose was also reduced by dapagliflozin treatment accordingly. Taken together, our results firstly revealed that dapagliflozin treatment relieved steatosis and reduced the expression levels of enzymes involved in DNL by upregulating FXR/SHP and downregulating LXRα/SREBP-1c and inhibiting glucose output in the liver of db/db mice.

First-line treatment for nonalcoholic fatty liver is lifestyle intervention to reduce body weight. Medicinal treatment for NASH is required to improve hepatic injury in patients (Chalasani et al., 2017). In our study, dapagliflozin ameliorated steatosis in T2DM-related NASH. Therefore, we are concerned about whether dapagliflozin can alleviate hepatic injury, and what the mechanism is.

Inflammation and fibrosis induced by excess lipid accumulation play a vital role in the process from NAFLD to NASH (Takaki et al., 2013). Accordingly, terminating inflammatory response has been actively investigated as potential pharmacological target (Musso et al., 2016). The inflammatory response in NAFLD is a process involving a series of cytokines, chemokines, and inflammatory cells. Among them, the transcription factor NF-*κ*B, as a master regulator in inflammation (Baker et al., 2011; Michelotti et al., 2013), is activated in rodent models and in patients with NASH (Dela Pena et al., 2005). The activation of NF-*κ*B within hepatocytes is sufficient to trigger hepatic inflammation, steatosis, and insulin resistance (Cai et al., 2005). In db/db mice, the NF-*κ*B pathway was activated with increased phosphorylation of the NF-*κ*B p65 subunit, which was inhibited by dapagliflozin treatment. In animal experiments, inhibiting NF-*κ*B not only decreased the level of inflammatory cytokines but also protected mice against hepatic injury and cancer (Pikarsky et al., 2004). The NF-*κ*B pathway is regulated by the MAPK signaling pathway (Zhang et al., 2017) and JAK/STAT3 pathway (Yang et al., 2007; Kaffe et al., 2017). Both of them were activated with elevated phosphorylation levels that were further decreased by dapagliflozin in the liver of db/db mice. Therefore, it is confirmed that dapagliflozin treatment inhibited NF-*κ*B pathway activation and further lowered inflammatory response via reducing the activity of the MAPK pathway and JAK/STAT3 pathway.

Inflammation occurs in all tissues in response to injury or stress and is the key process underlying hepatic fibrogenesis. The process of fibrosis is characterized by the accumulation of excess extracellular matrix. When the synthesis of extracellular matrix exceeds its degradation, the pathological accumulation of the extracellular matrix leads to hepatic fibrosis (Hu et al., 2009). A study showed that dapagliflozin slowed the progression of hepatic fibrosis via inhibiting extracellular matrix accumulation in db/db mice with right uninephrectomy (Tang et al., 2017). Consistent with the report (Tang et al., 2017), dapagliflozin also reduced the expression levels of fibronectin and α-SMA in the liver of db/db mice. Dapagliflozin had no effect on the increased expression levels of pro-fibrotic factors (TGF-β1 and CTGF), however, it can reduce the expression levels of fibrolysis-inhibiting proteins. PAI-1 is involved in the inhibition of matrix degradation and is a promising therapeutic target for fibrotic diseases in the cirrhotic liver (Zhang et al., 1999; Hu et al., 2009). Downregulation of PAI-1 ameliorates hepatic fibrosis by facilitating matrix degradation through upregulation of MMPs and downregulation of TIMP-1 (Hu et al., 2009). Both PAI-1 and TIMP-1 were upregulated and then downregulated by dapagliflozin treatment in db/db mice. Furthermore, the increased expression of MMP9 in the liver of db/db mice was also decreased by dapagliflozin. These results suggest that dapagliflozin mainly recovered the process of fibrolysis and further inhibited fibrosis in the liver of db/db mice.

NASH is not only the feature of metabolic syndrome prevalent in NAFLD patients, but components of metabolic syndrome also increase the risk of NAFLD (Argo and Caldwell, 2009; Younossi et al., 2011; Byrne and Targher, 2015; Younossi et al., 2016). Therefore, research on metabolic targets for the treatment of NASH has become increasingly popular. Several clinical trials of metabolic pharmacotherapies for the treatment of NASH including farnesoid X receptor (FXR) agonists (obeticholic acid) (NCT02548351, NCT03439254), PPARα and PPARδ agonists (elafibranor) (NCT02704403), glucagon-like peptide (GLP)-1 (semaglutide) (NCT02970942) have been studied. Attenuating metabolic drivers of NASH should be an effective therapy. Either FXR agonists, PPARγ ligands, or GLP-1 all act on insulin sensitivity, so we aimed to find a new mechanism that was independent of insulin sensitivity.

As an SGLT2 inhibitor, the glucose-lowering effect of dapagliflozin does not increase the risk of hypoglycemia (Bailey et al., 2010; Kaku et al., 2014) and is independent of insulin secretion or action (Musso et al., 2012). In this study, we further demonstrated that dapagliflozin can alleviate NASH. Blood ALT and aspartate aminotransferase levels were significantly decreased by dapagliflozin in patients with T2DM in a 24-weeks clinical trial (Hayashi et al., 2017). It is very beneficial for patients with T2DM to use dapagliflozin in the long term.

**FIGURE 7:**
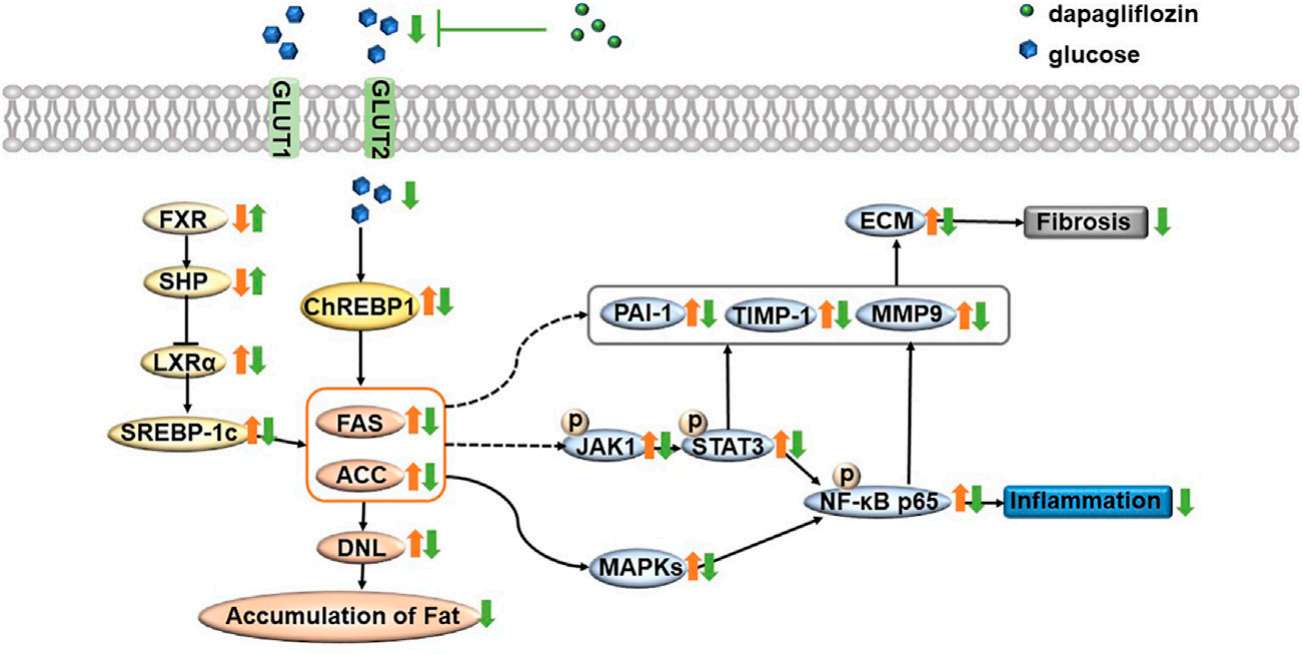
Summary of the underlying mechanism of dapagliflozin in alleviating nonalcoholic steatohepatitis. In brief, dapagliflozin treatment alleviates steatosis and reduces fat accumulation by inhibiting the FXR/SHP/LXRα/SREBP-1c pathway and reducing ChREBP1 expression in the liver of db/db mice. In addition, dapagliflozin treatment inhibits NF-*κ*B pathway activation and further lowers inflammatory response via reducing the activity of the MAPK pathway and JAK/STAT3 pathway. Importantly, dapagliflozin restores the process of fibrosis and further inhibits fibrosis by downregulating the expression of PAI-1, TIMP-1, and MMP9 in the liver of db/db mice. Orange arrows represent pathological states; green arrows represent dapagliflozin treatment status; FXR = farnesoid X receptor; SHP = small heterodimer partner; LXRα = liver X receptor α; SREBP-1c = sterol regulatory element binding transcription factor; FAS = fatty acid synthase; ACC = acetyl-CoA carboxylase; ChREBP1 = carbohydrate responsive element binding protein; DNL = *de novo* lipogenesis; NF-κB = nuclear factor kappa B; MAPK = mitogen-activated protein kinase; JAK = Janus-activated kinase; STAT3 = signal transducer and activator of transcription 3; PAI-1 = plasminogen activator inhibitor-1; TIMP-1 = tissue inhibitor of metalloproteinase-1; MMP9 = matrix metalloproteinase-9.

In conclusion, dapagliflozin treatment alleviates steatosis and reduces fat accumulation by inhibiting the FXR/SHP/LXRα/SREBP-1c pathway and reducing ChREBP1 expression in the liver of db/db mice. In addition, dapagliflozin treatment inhibits NF-*κ*B pathway activation and further lowers inflammatory response via reducing the activity of the MAPK pathway and JAK/STAT3 pathway. Importantly, dapagliflozin restores the process of fibrosis and further inhibits fibrosis by downregulating the expression of PAI-1, TIMP-1, and MMP9 in the liver of db/db mice (Figure 7). These findings provide new insights for understanding the protective effect of dapagliflozin on NASH and suggest that dapagliflozin may be used to treat NASH.

## Data Availability

The original contributions presented in the study are included in the article/Supplementary Material; further inquiries can be directed to the corresponding authors.
