# Supplementary figures and images for: Dapagliflozin protects against nonalcoholic steatohepatitis in db/db mice (part 1 of 3)
Source: Front Pharmacol. 2022 Aug 19;13:934136. doi: 10.3389/fphar.2022.934136 (PMC9437261; doi:10.3389/fphar.2022.934136)

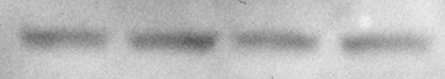

Supplement: Supplementary file 1 [file DataSheet3.ZIP › WB MAPK/JAK1.tif]

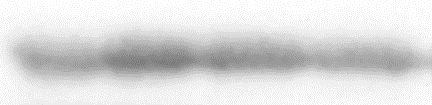

Supplement: Supplementary file 1 [file DataSheet3.ZIP › WB MAPK/JNK2 上1,2 下3 (3) liver.tif]

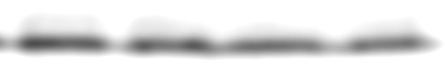

Supplement: Supplementary file 1 [file DataSheet3.ZIP › WB MAPK/liver ERK2 上1,2 下3 (3).tif]

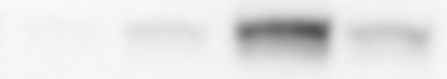

Supplement: Supplementary file 1 [file DataSheet3.ZIP › WB MAPK/liver p-stat3 上 1,2 下3 (8).tif]

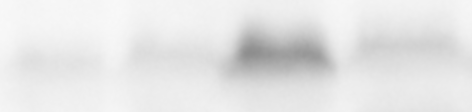

Supplement: Supplementary file 1 [file DataSheet3.ZIP › WB MAPK/p-ERK(42,44) 上1,2 下3 liver (8).tif]

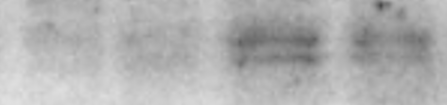

Supplement: Supplementary file 1 [file DataSheet3.ZIP › WB MAPK/p-JAK1(137) 上3 下1,2 liver (7).tif]

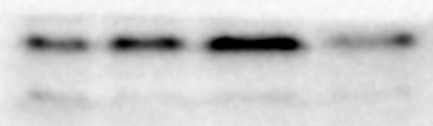

Supplement: Supplementary file 1 [file DataSheet3.ZIP › WB MAPK/p-JNK(46,54) 上1,2 下3 liver (3).tif]

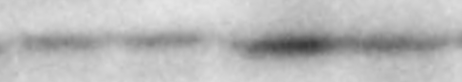

Supplement: Supplementary file 1 [file DataSheet3.ZIP › WB MAPK/p-p38(43) 上1,2 下3 liver (3).tif]

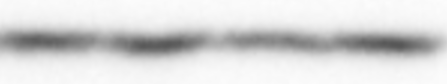

Supplement: Supplementary file 1 [file DataSheet3.ZIP › WB MAPK/p38(45) 上3 下1,2 liver.tif]

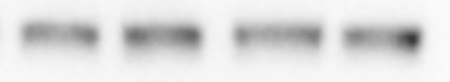

Supplement: Supplementary file 1 [file DataSheet3.ZIP › WB MAPK/stat3(79,86) liver上1,2 下3 (10).tif]

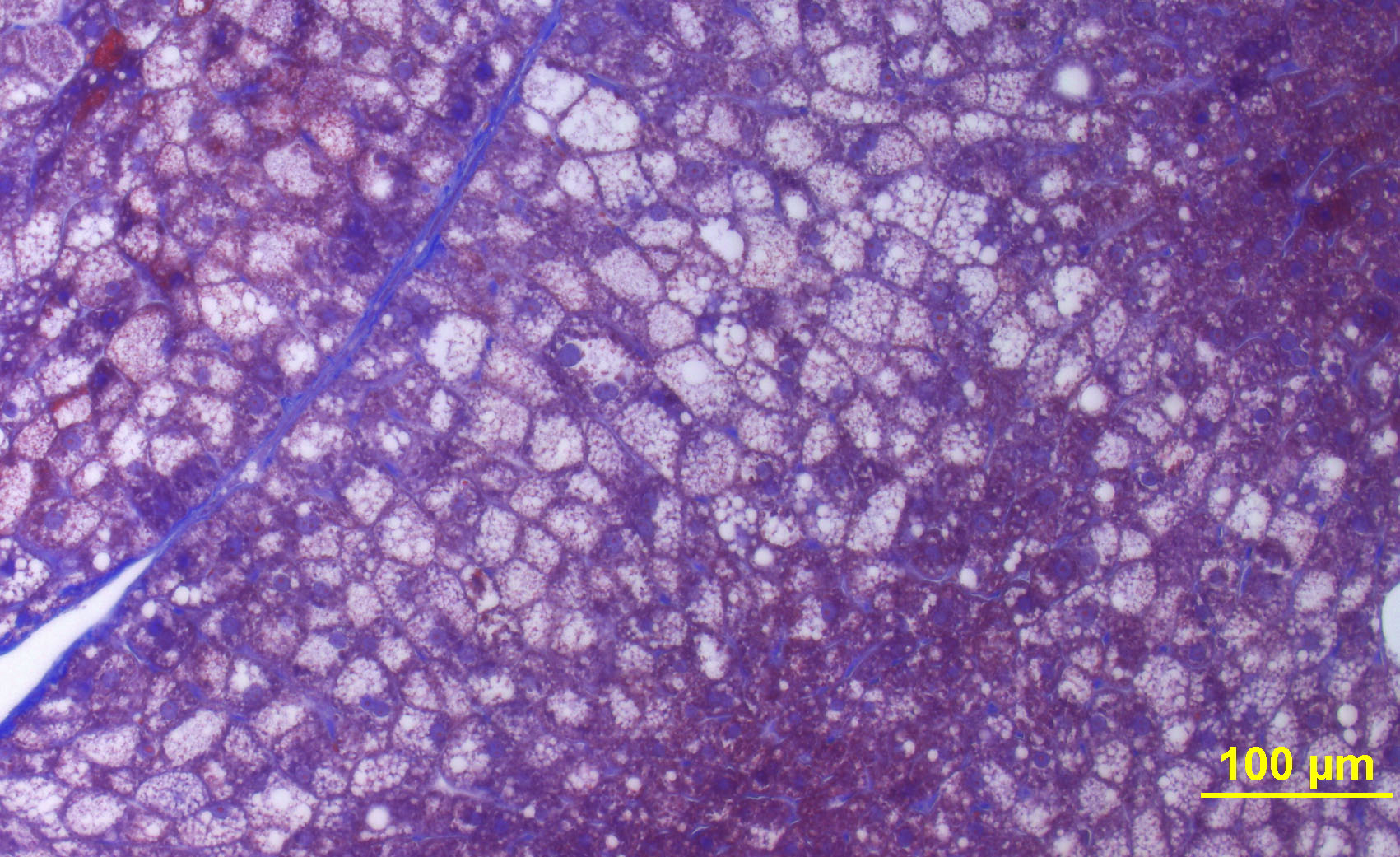

Supplement: Supplementary file 2 [file DataSheet11.ZIP › oil red O liver/20X 标尺.jpg]

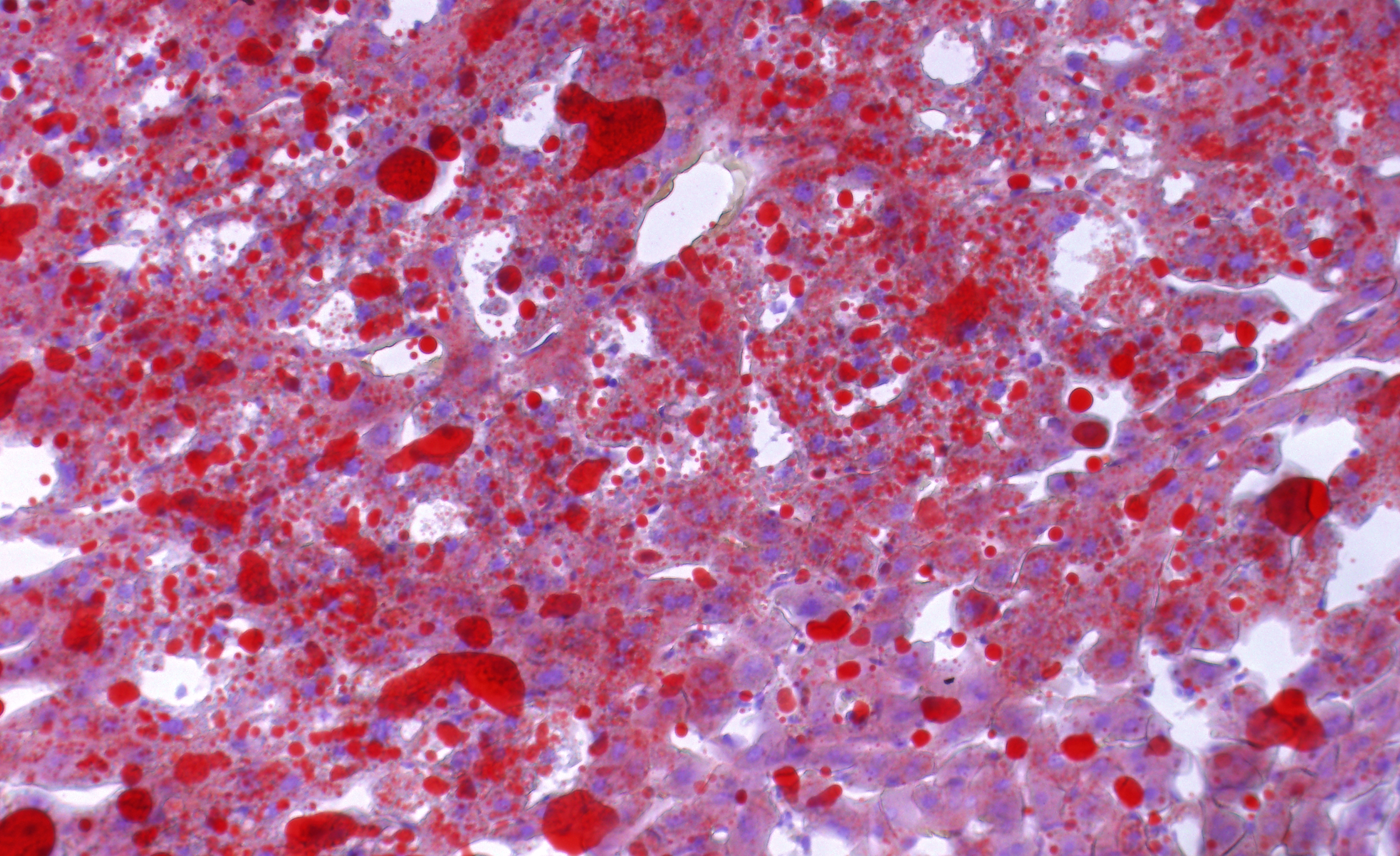

Supplement: Supplementary file 2 [file DataSheet11.ZIP › oil red O liver/db Image222.tif]

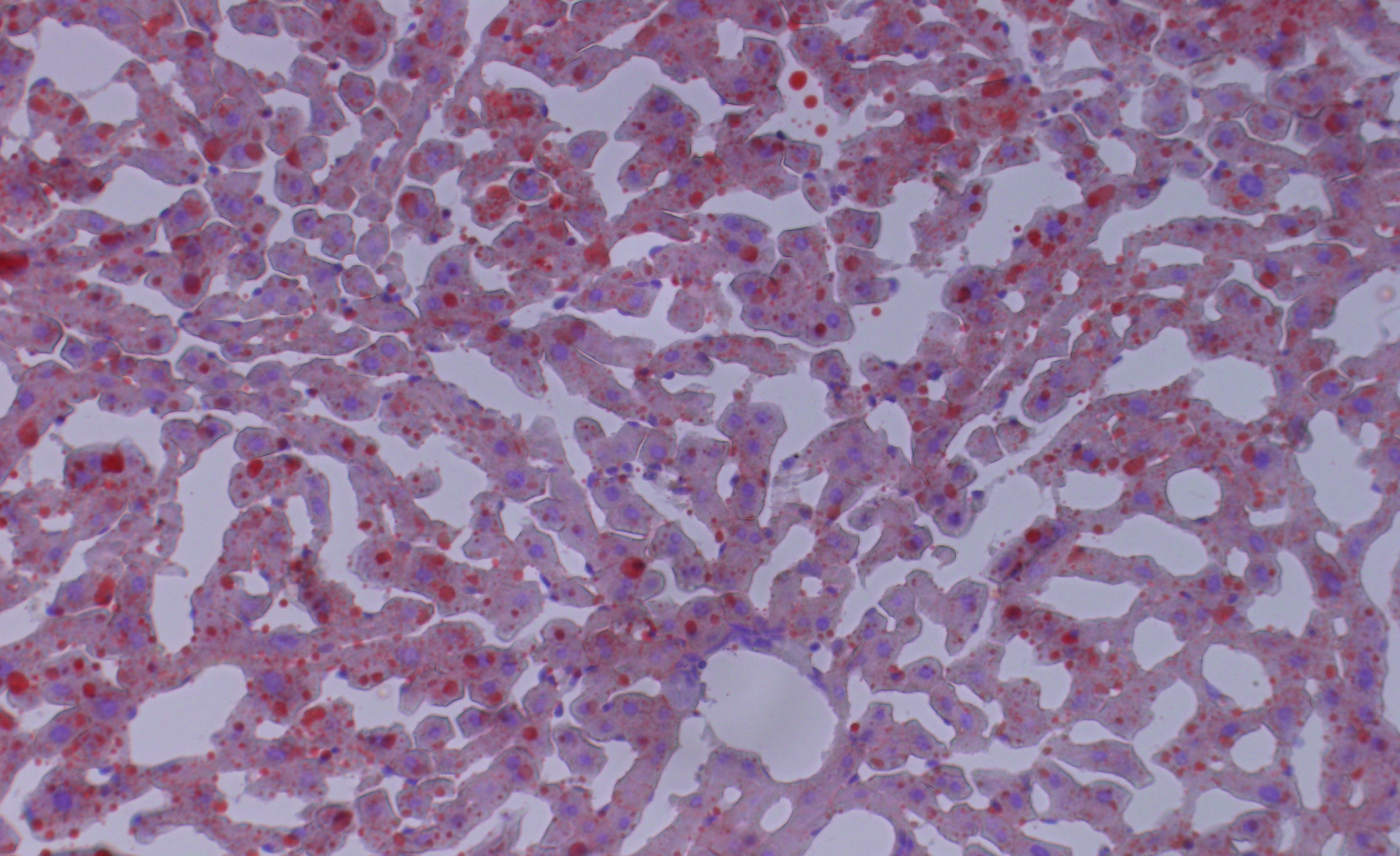

Supplement: Supplementary file 2 [file DataSheet11.ZIP › oil red O liver/db+ Image219.tif]

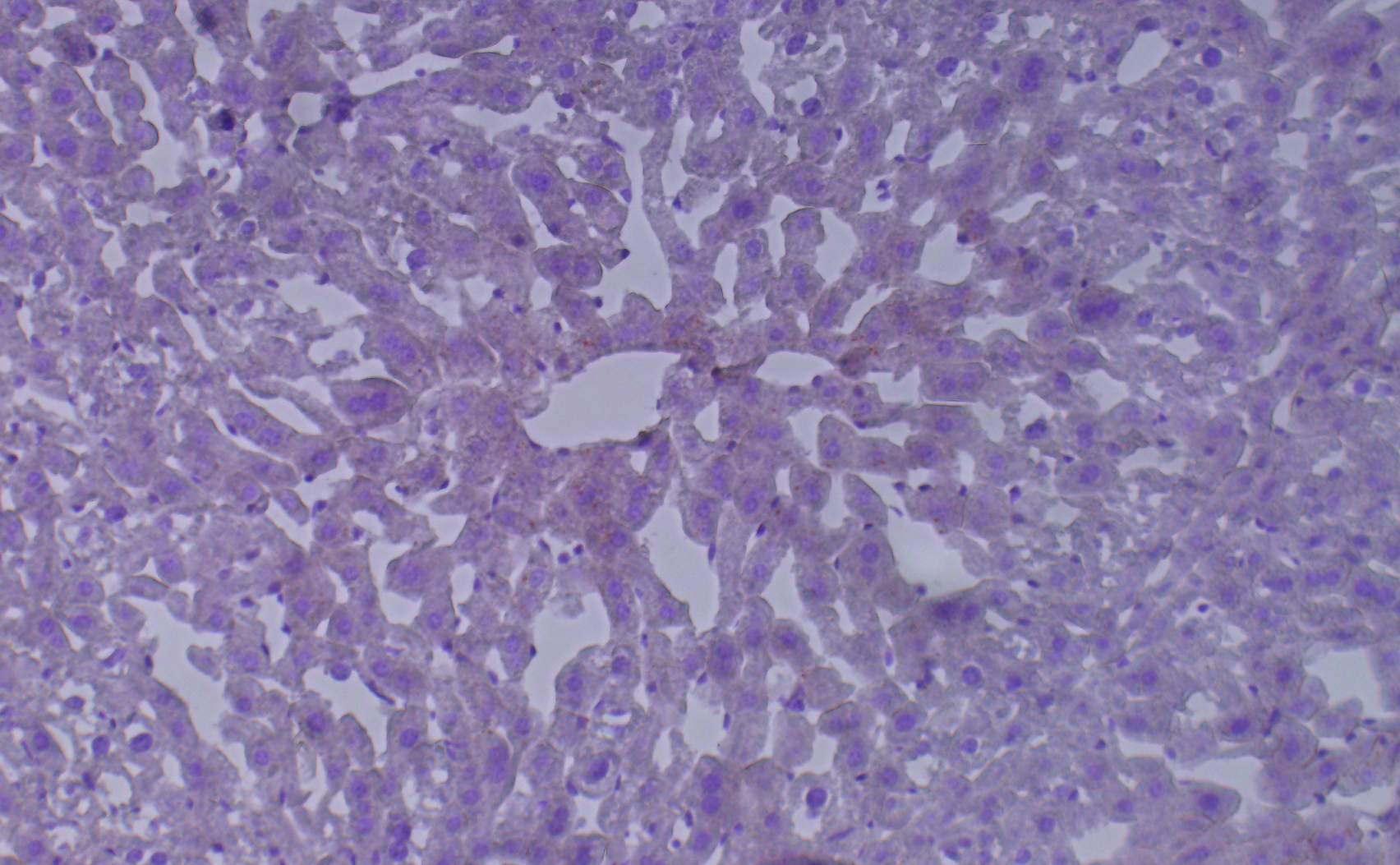

Supplement: Supplementary file 2 [file DataSheet11.ZIP › oil red O liver/wt Image182.tif]

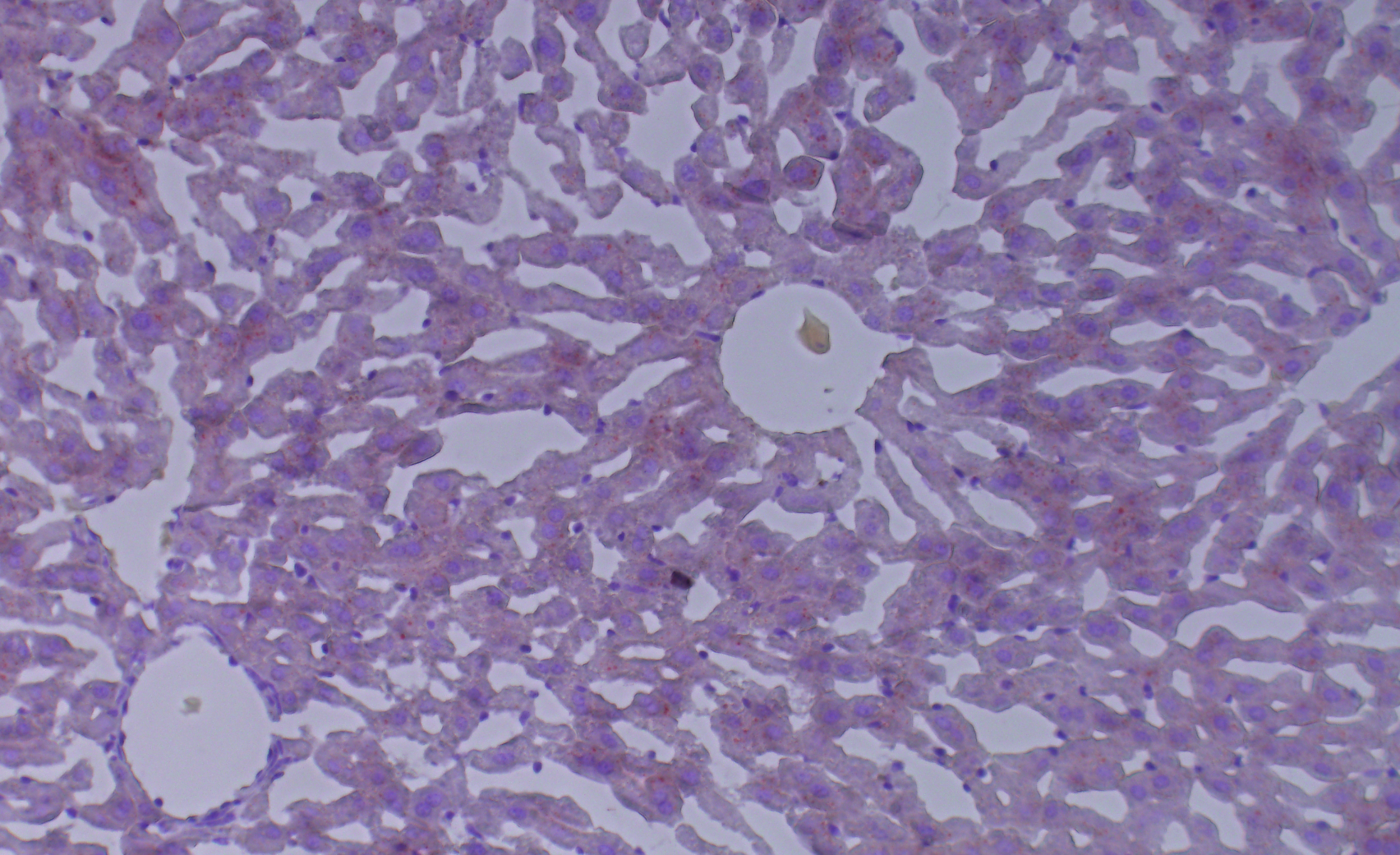

Supplement: Supplementary file 2 [file DataSheet11.ZIP › oil red O liver/wt+ Image208.tif]

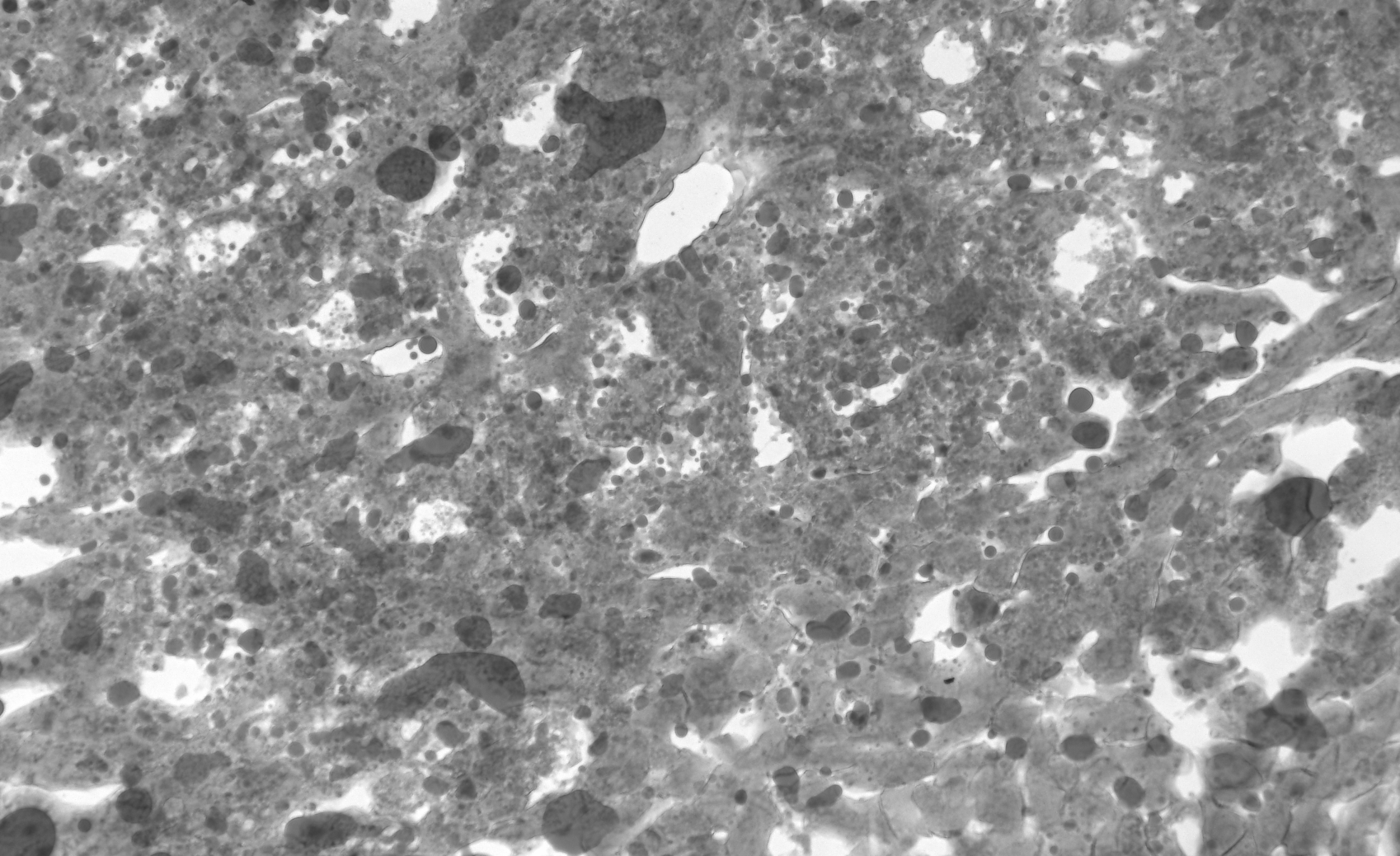

Supplement: Supplementary file 2 [file DataSheet11.ZIP › oil red O liver/模版 切.tif]

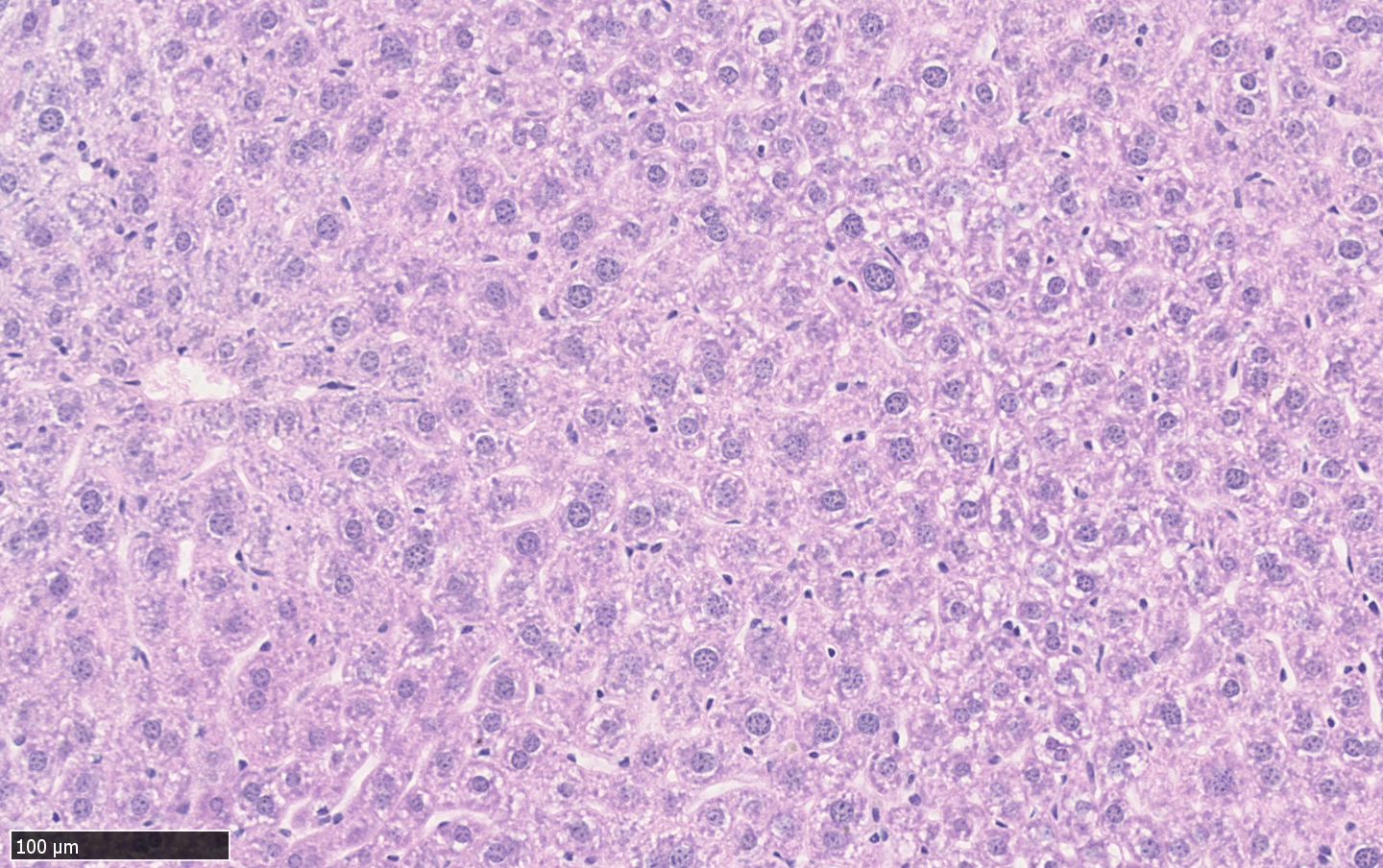

Supplement: Supplementary file 3 [file DataSheet8.ZIP › NASH SCORE-WT/WT11,12/1.jpg]

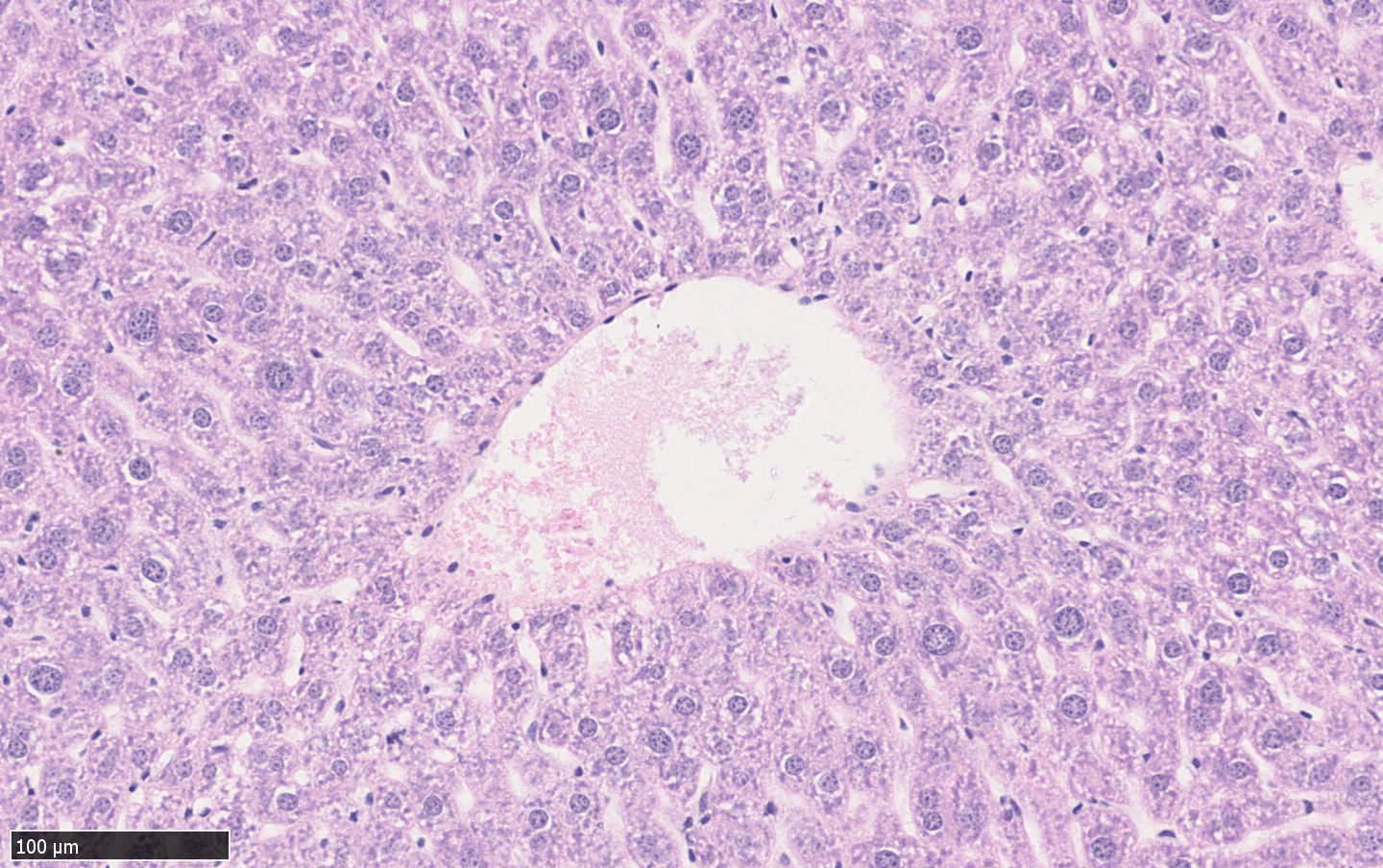

Supplement: Supplementary file 3 [file DataSheet8.ZIP › NASH SCORE-WT/WT11,12/10.jpg]

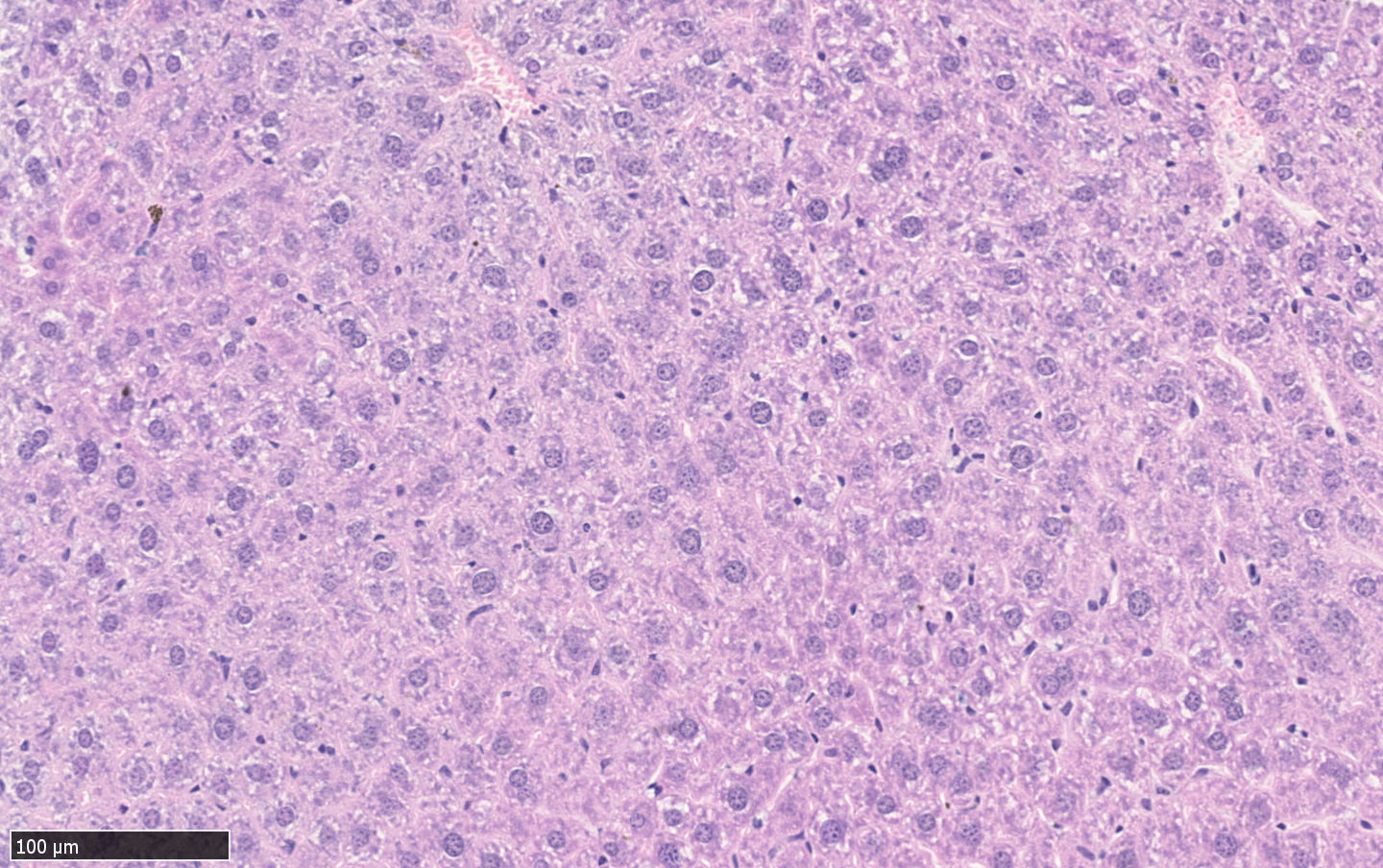

Supplement: Supplementary file 3 [file DataSheet8.ZIP › NASH SCORE-WT/WT11,12/11.jpg]

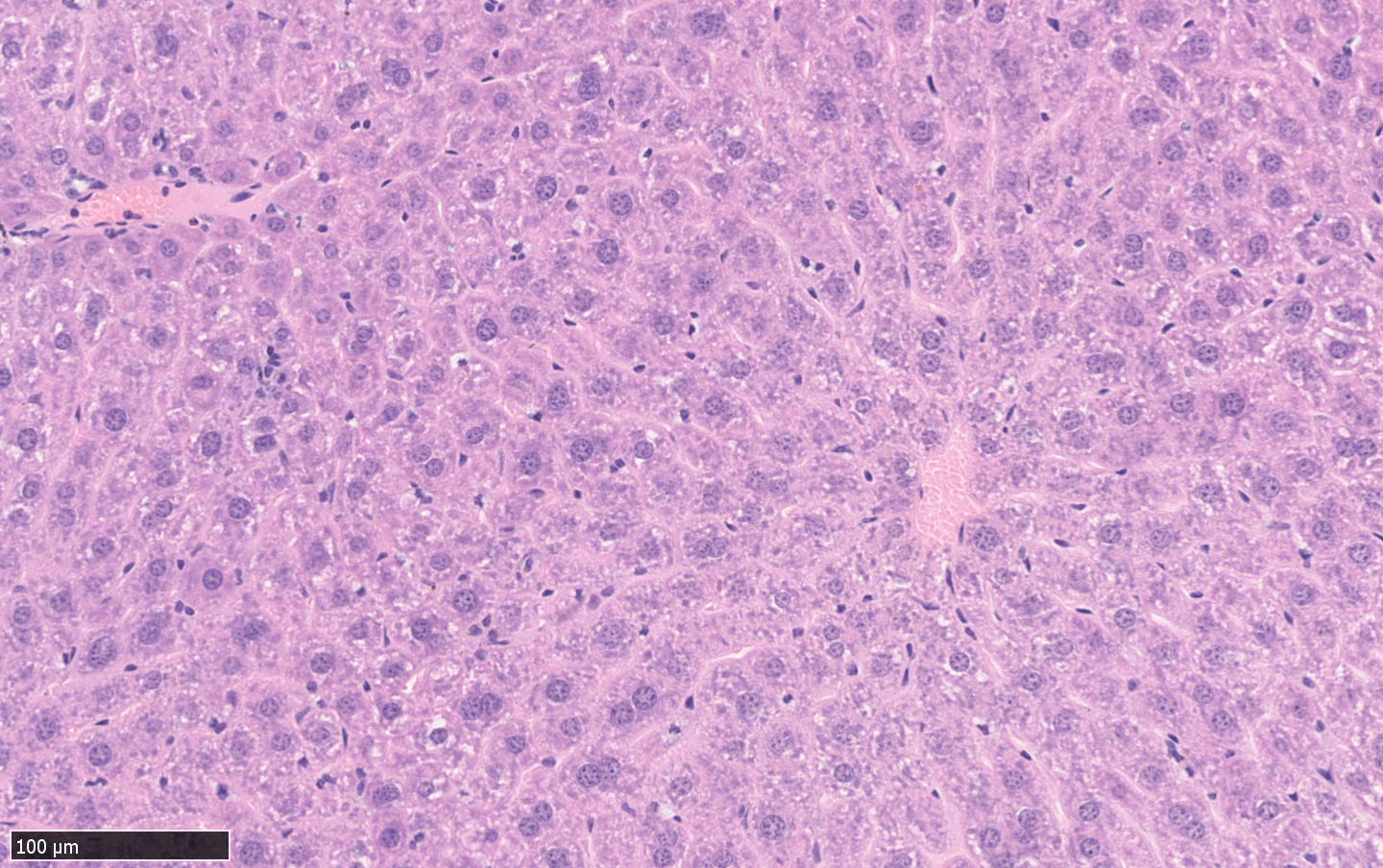

Supplement: Supplementary file 3 [file DataSheet8.ZIP › NASH SCORE-WT/WT11,12/12.jpg]

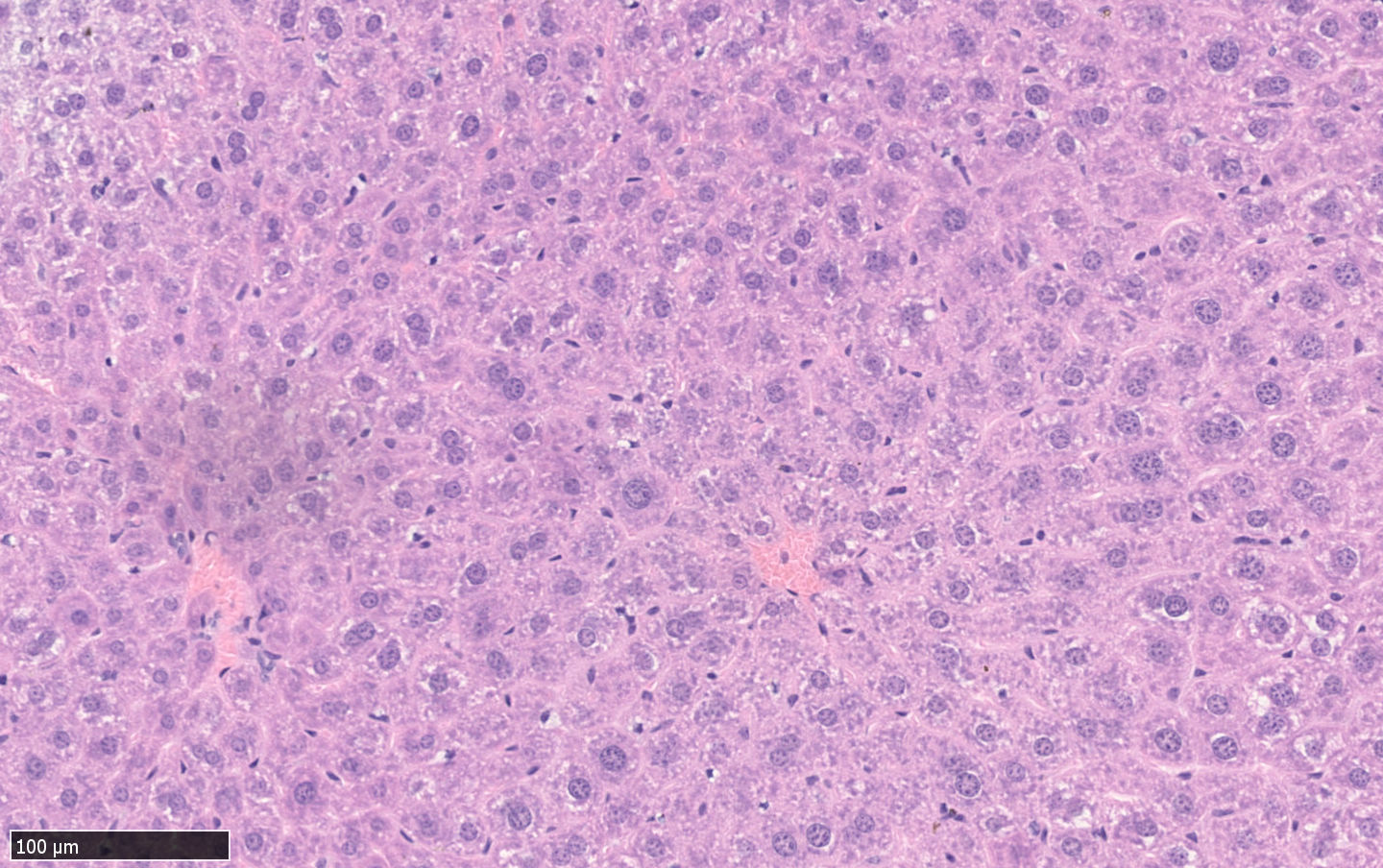

Supplement: Supplementary file 3 [file DataSheet8.ZIP › NASH SCORE-WT/WT11,12/13.jpg]

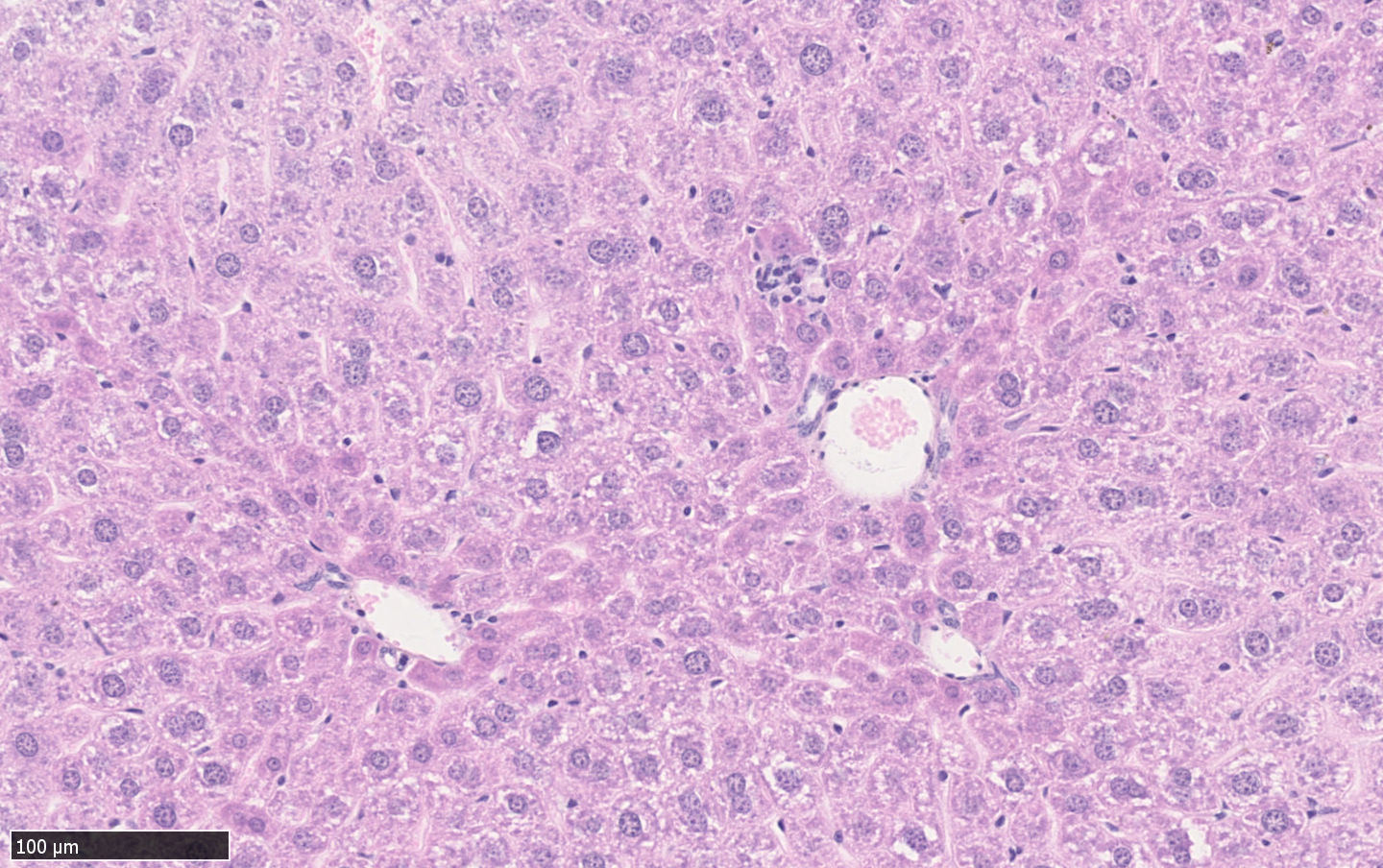

Supplement: Supplementary file 3 [file DataSheet8.ZIP › NASH SCORE-WT/WT11,12/14.jpg]

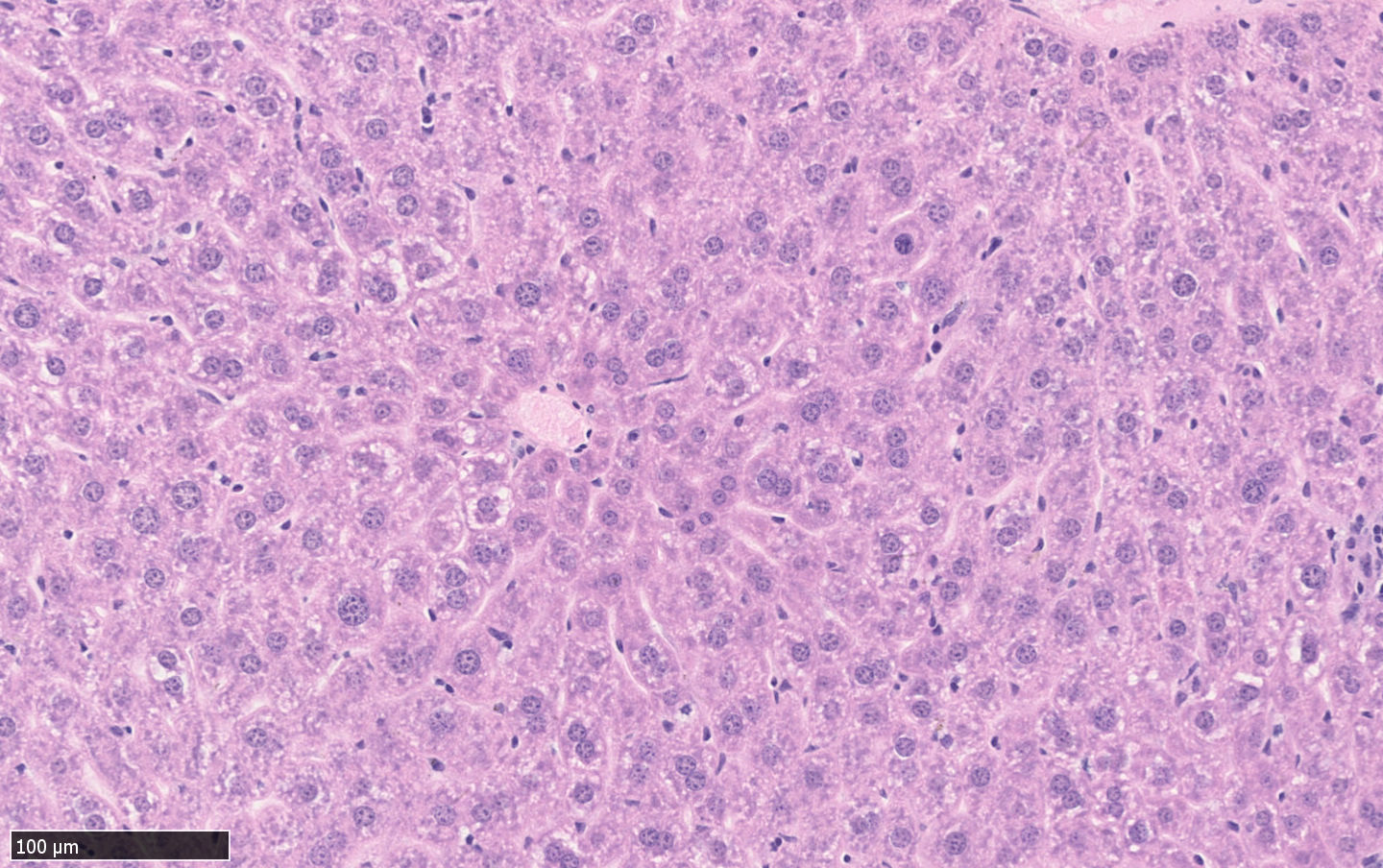

Supplement: Supplementary file 3 [file DataSheet8.ZIP › NASH SCORE-WT/WT11,12/15.jpg]

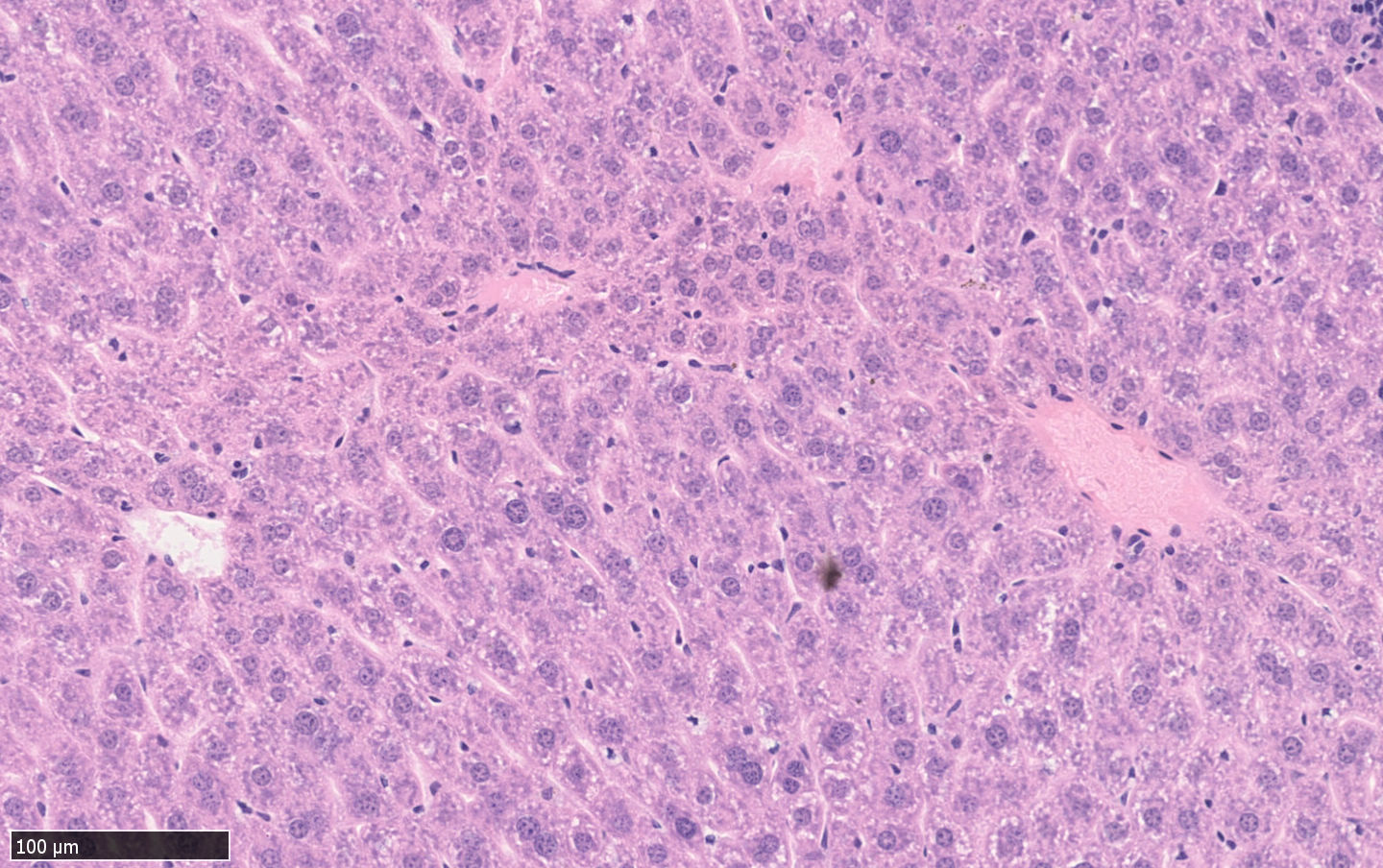

Supplement: Supplementary file 3 [file DataSheet8.ZIP › NASH SCORE-WT/WT11,12/16.jpg]

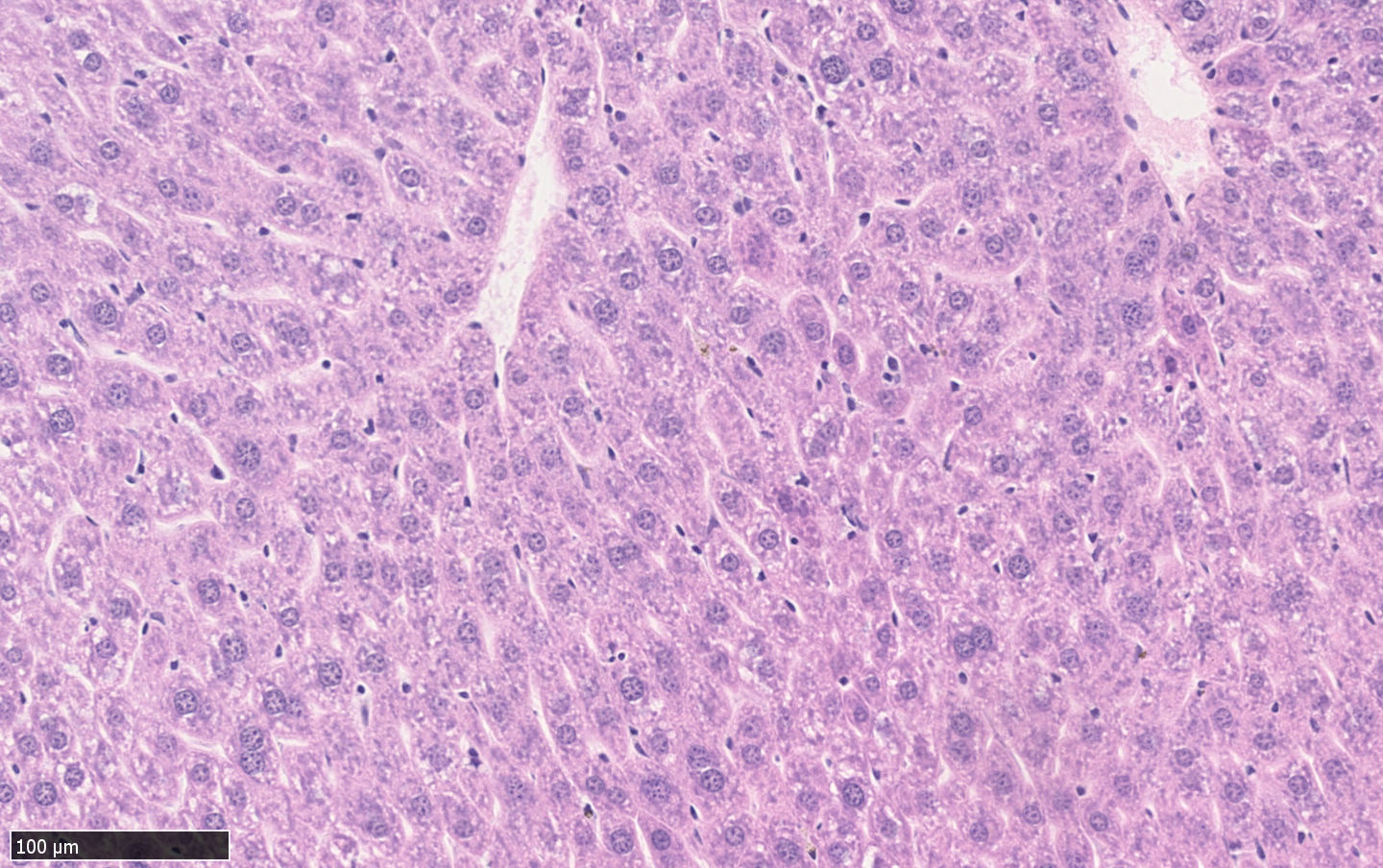

Supplement: Supplementary file 3 [file DataSheet8.ZIP › NASH SCORE-WT/WT11,12/17.jpg]

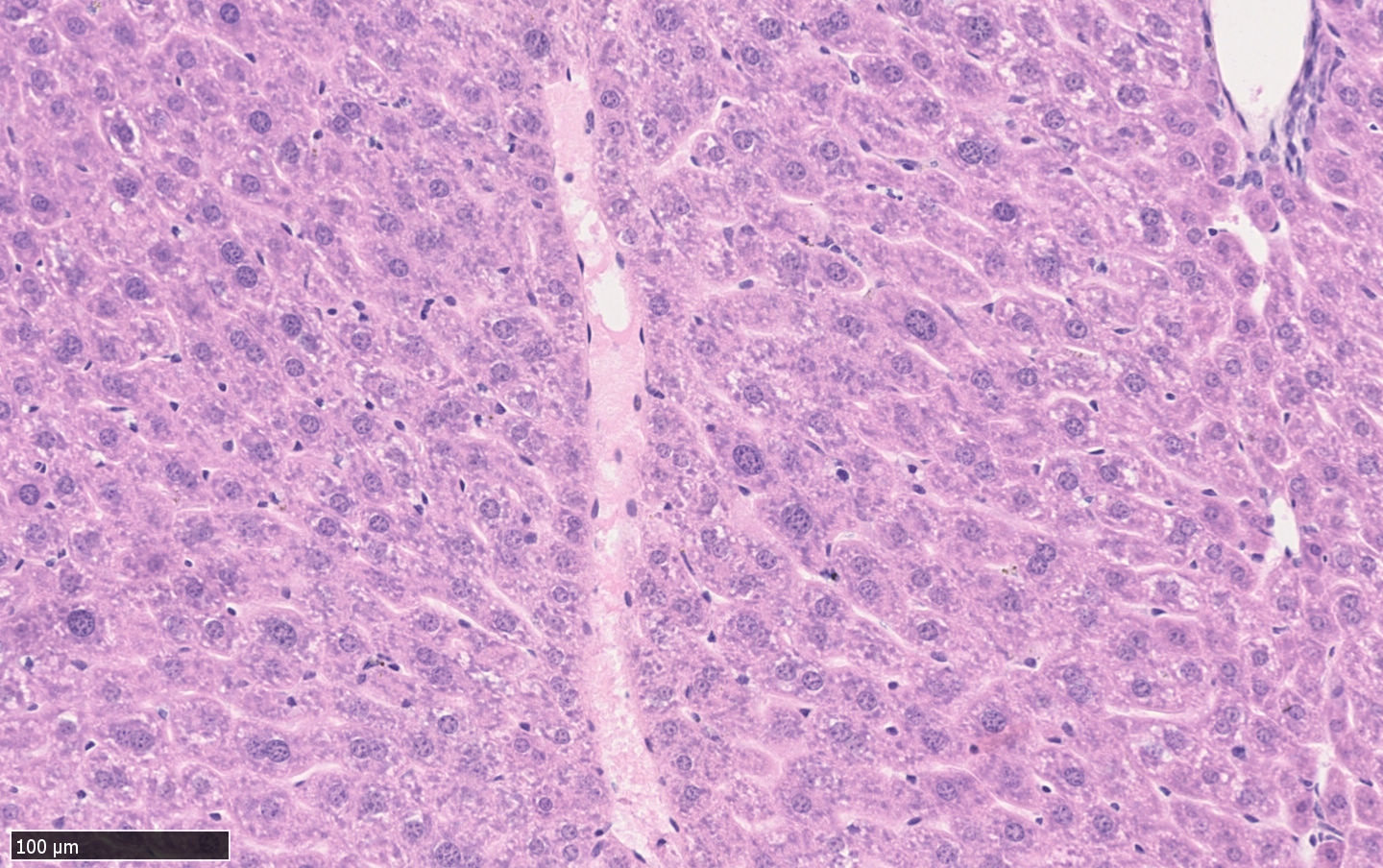

Supplement: Supplementary file 3 [file DataSheet8.ZIP › NASH SCORE-WT/WT11,12/18.jpg]

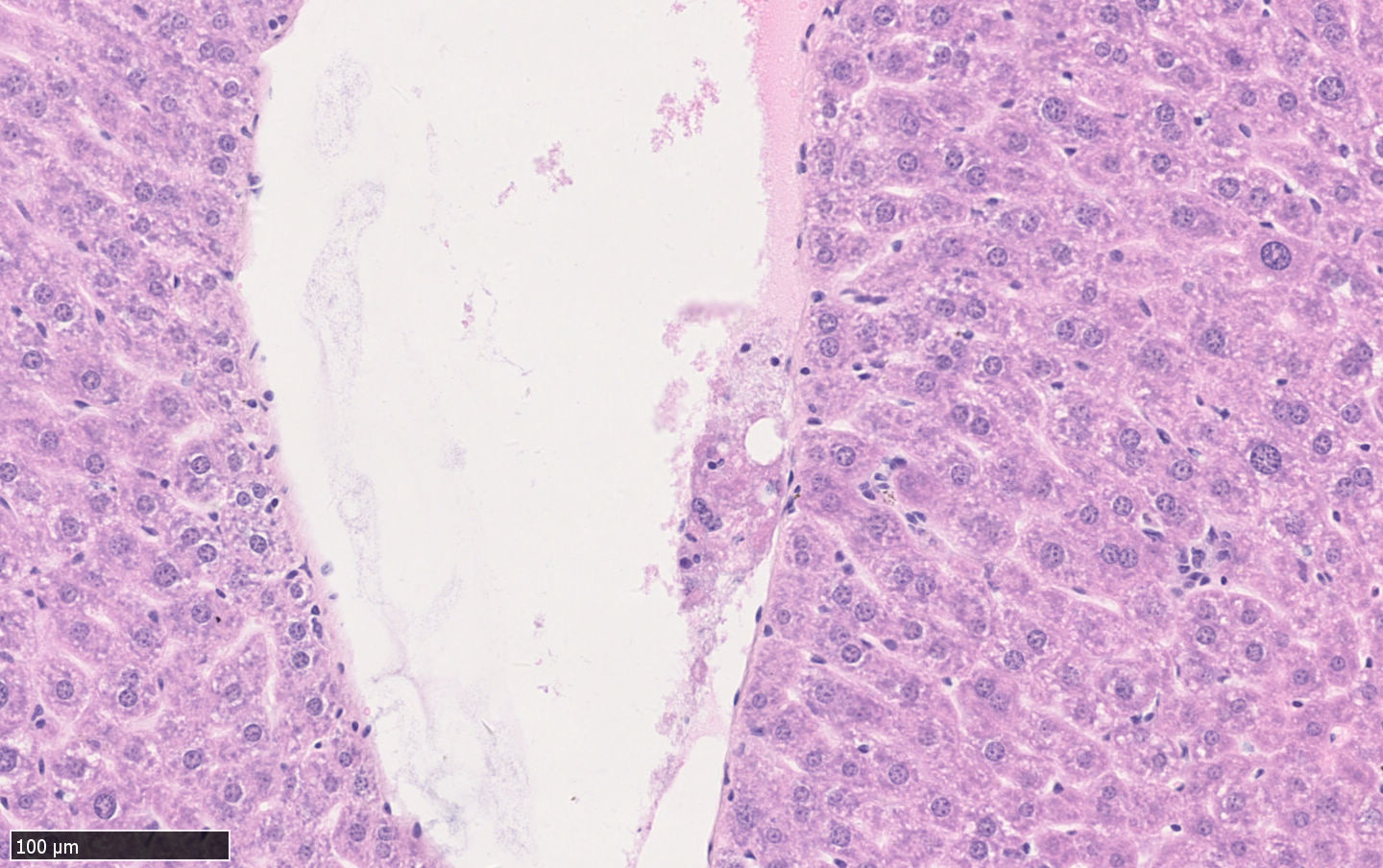

Supplement: Supplementary file 3 [file DataSheet8.ZIP › NASH SCORE-WT/WT11,12/19.jpg]

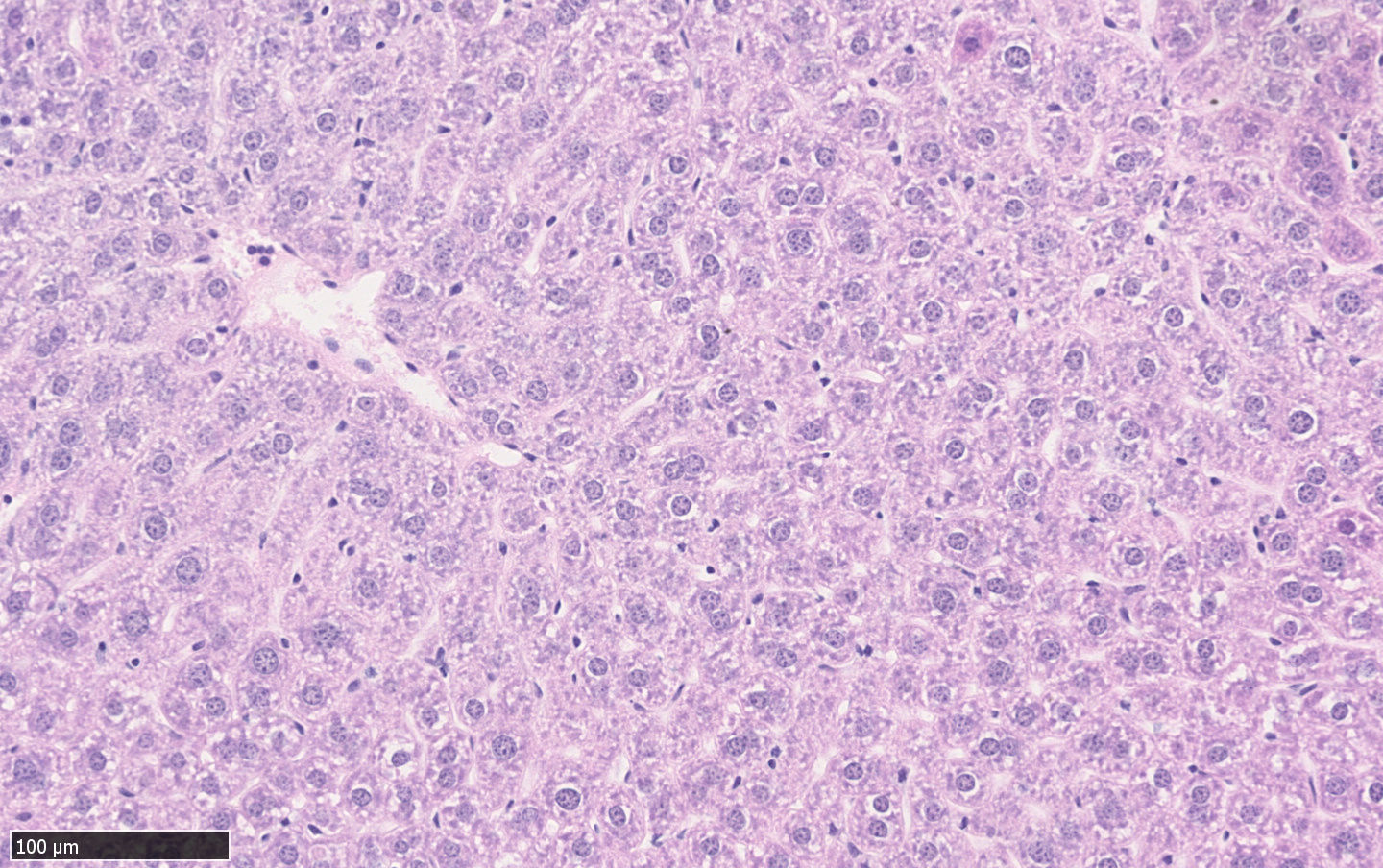

Supplement: Supplementary file 3 [file DataSheet8.ZIP › NASH SCORE-WT/WT11,12/2.jpg]

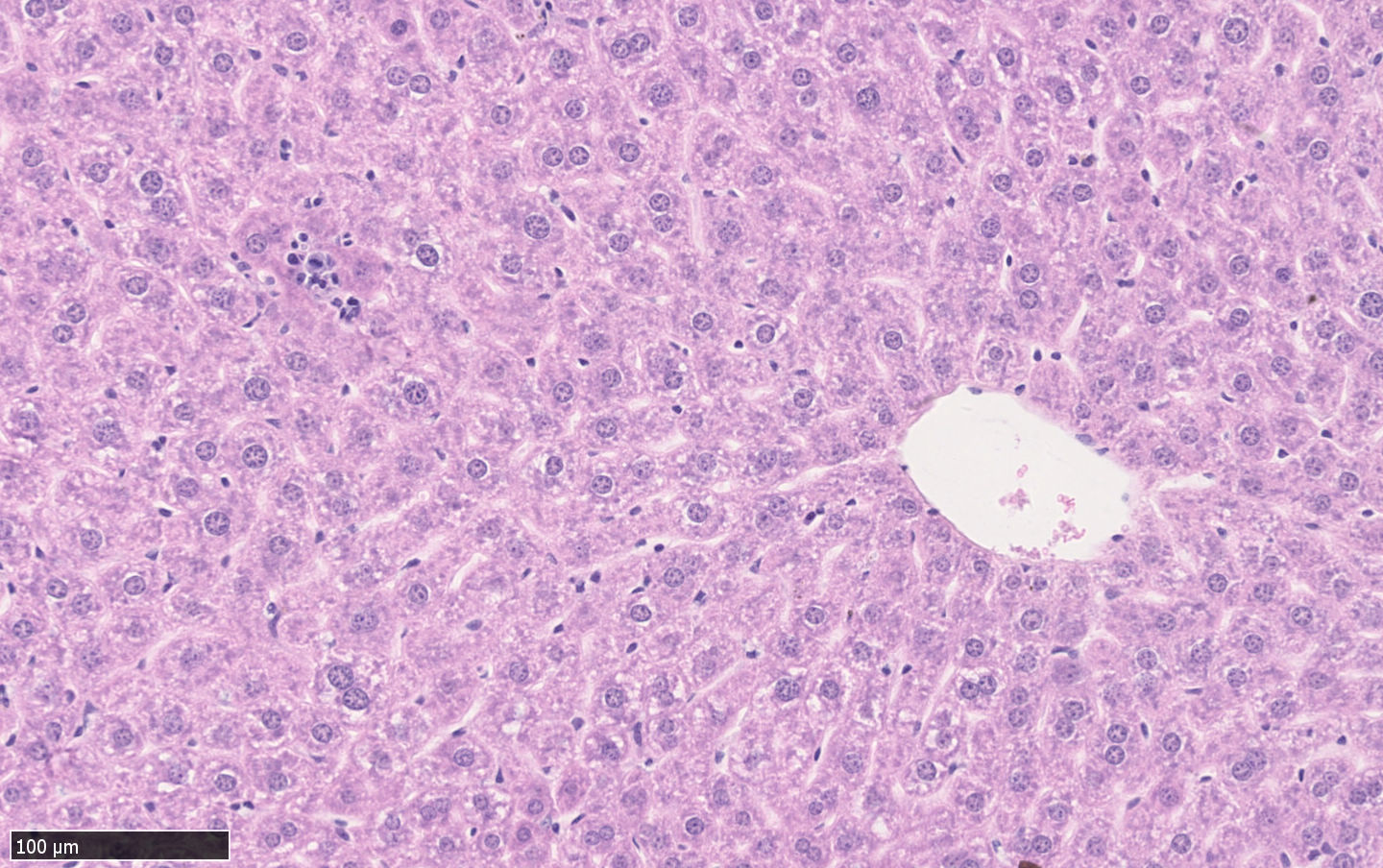

Supplement: Supplementary file 3 [file DataSheet8.ZIP › NASH SCORE-WT/WT11,12/20.jpg]

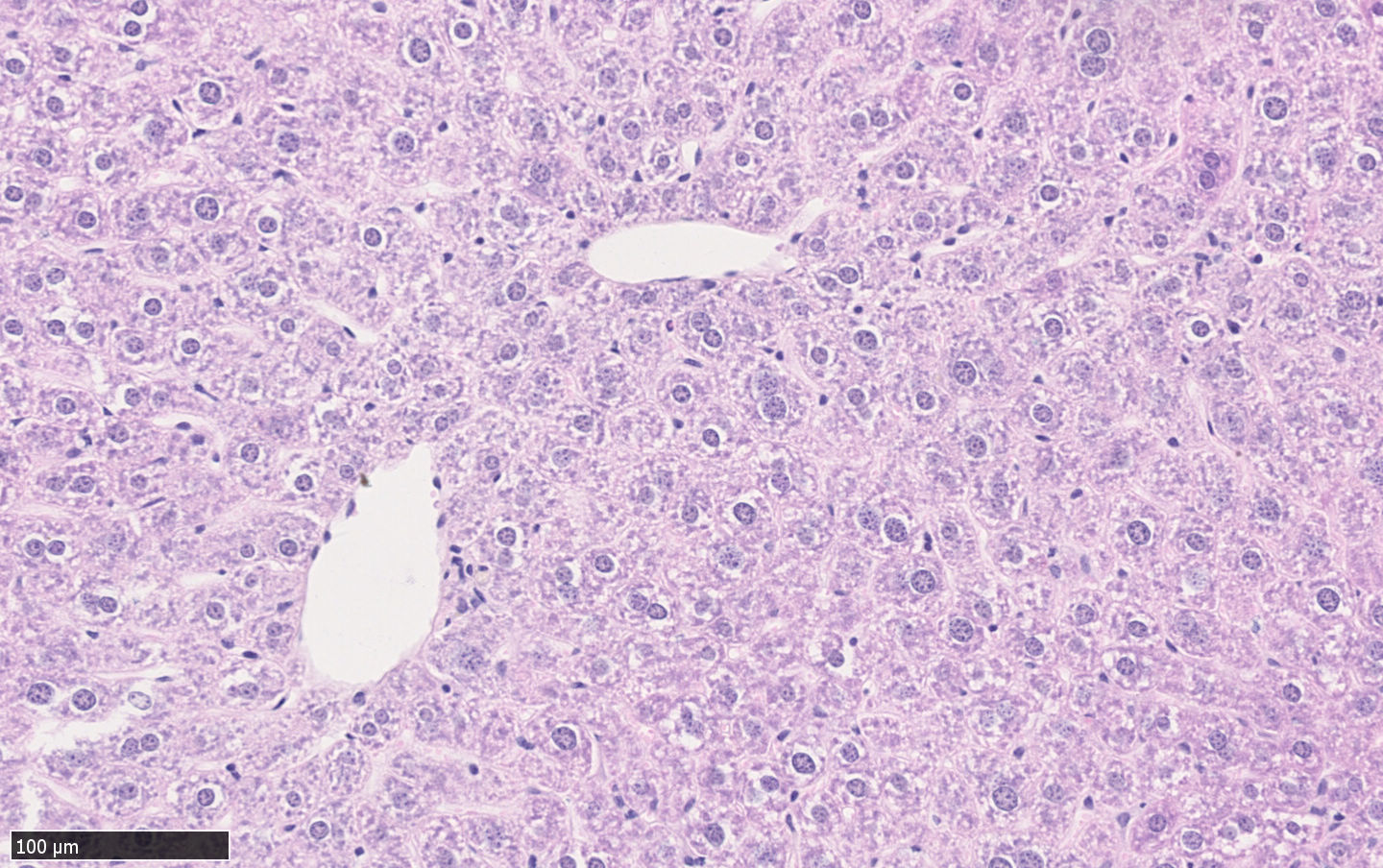

Supplement: Supplementary file 3 [file DataSheet8.ZIP › NASH SCORE-WT/WT11,12/3.jpg]

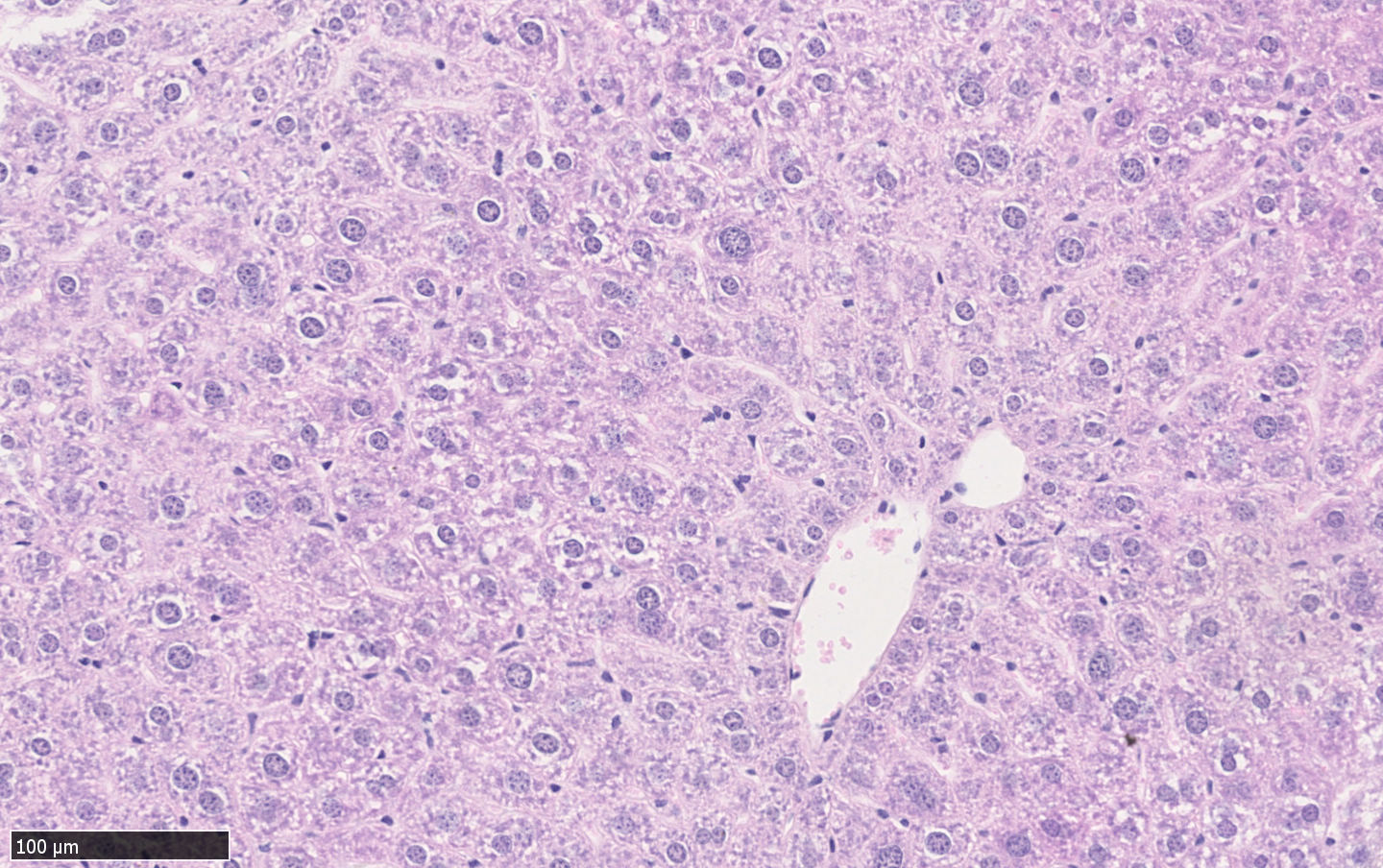

Supplement: Supplementary file 3 [file DataSheet8.ZIP › NASH SCORE-WT/WT11,12/4.jpg]

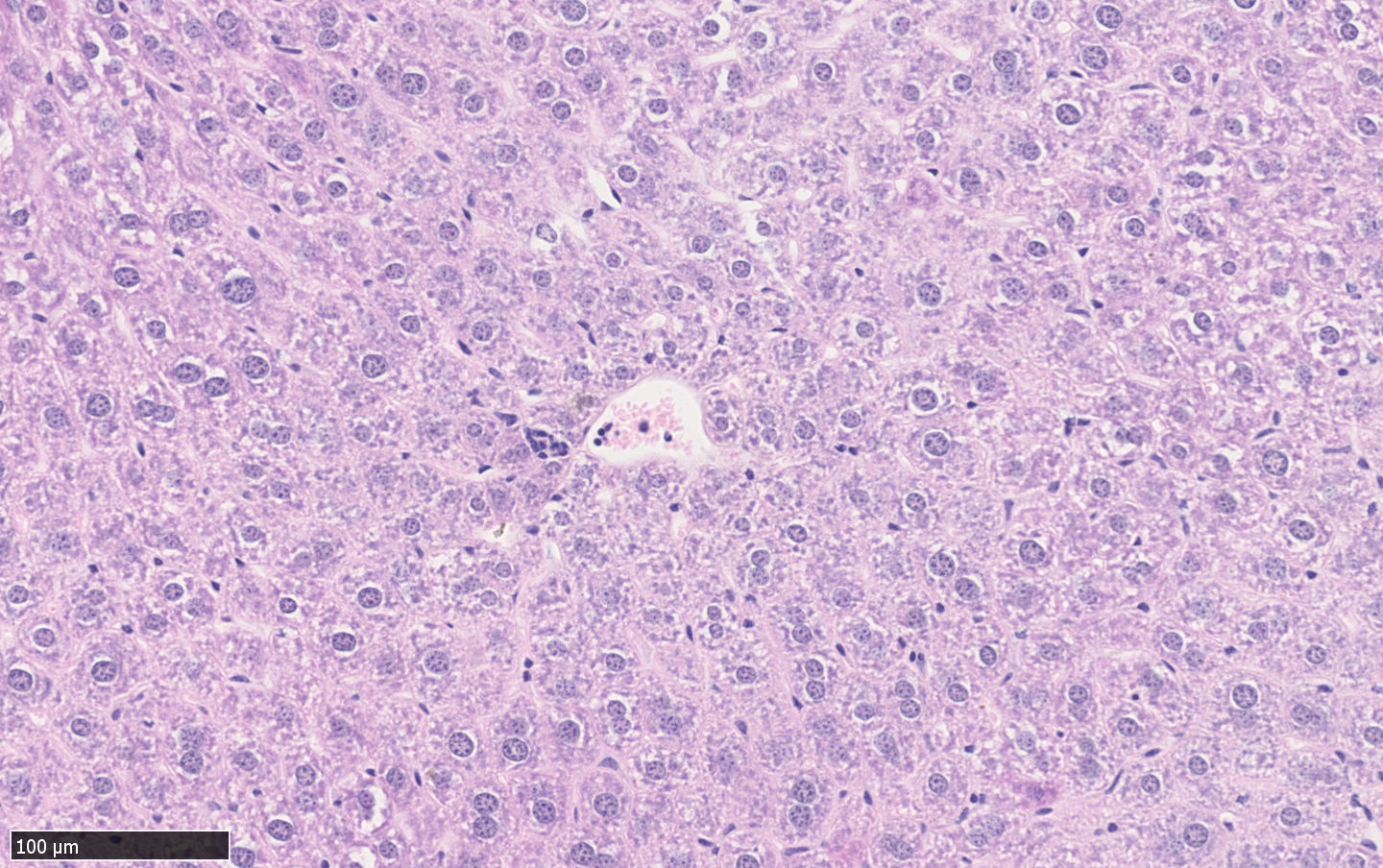

Supplement: Supplementary file 3 [file DataSheet8.ZIP › NASH SCORE-WT/WT11,12/5.jpg]

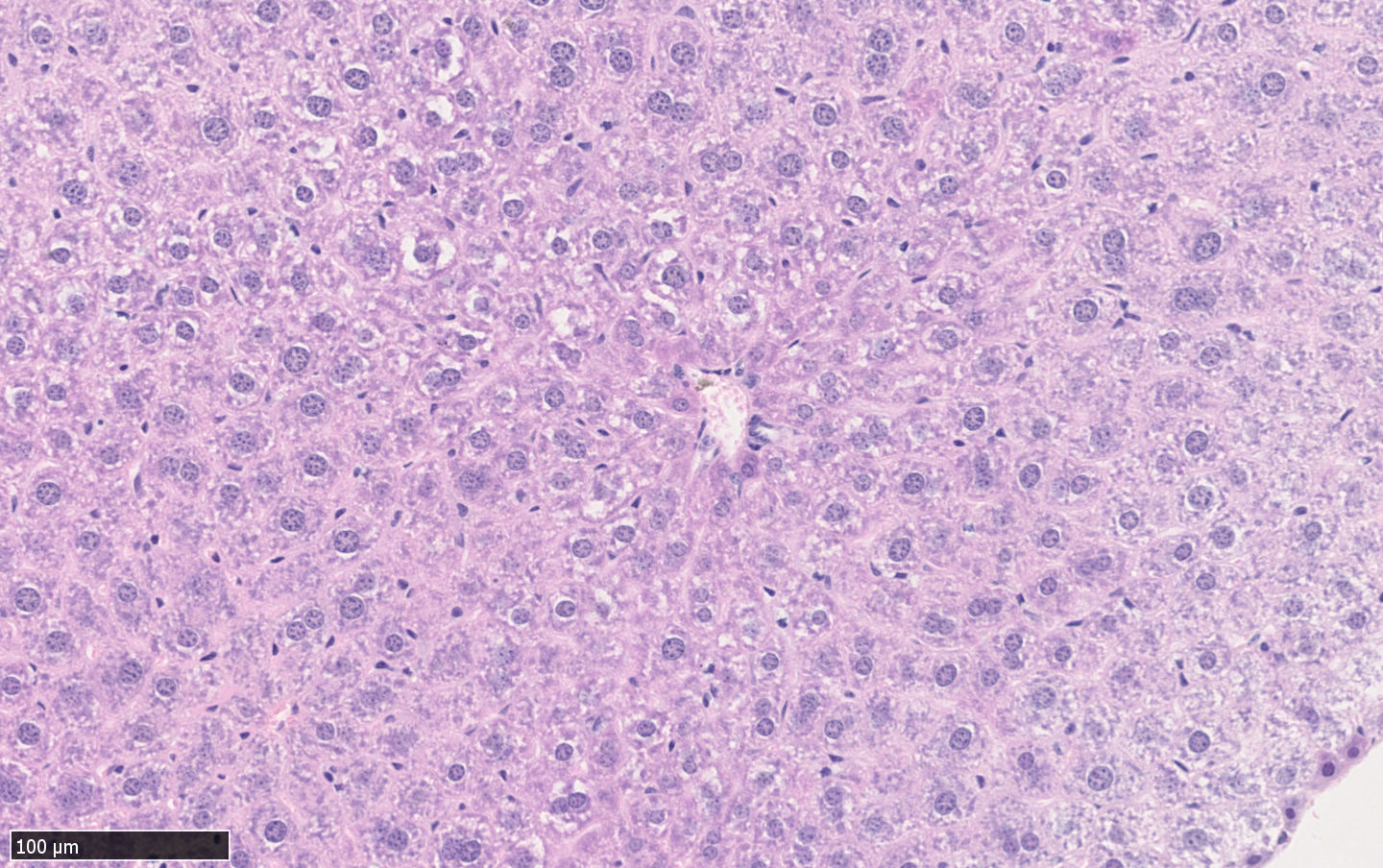

Supplement: Supplementary file 3 [file DataSheet8.ZIP › NASH SCORE-WT/WT11,12/6.jpg]

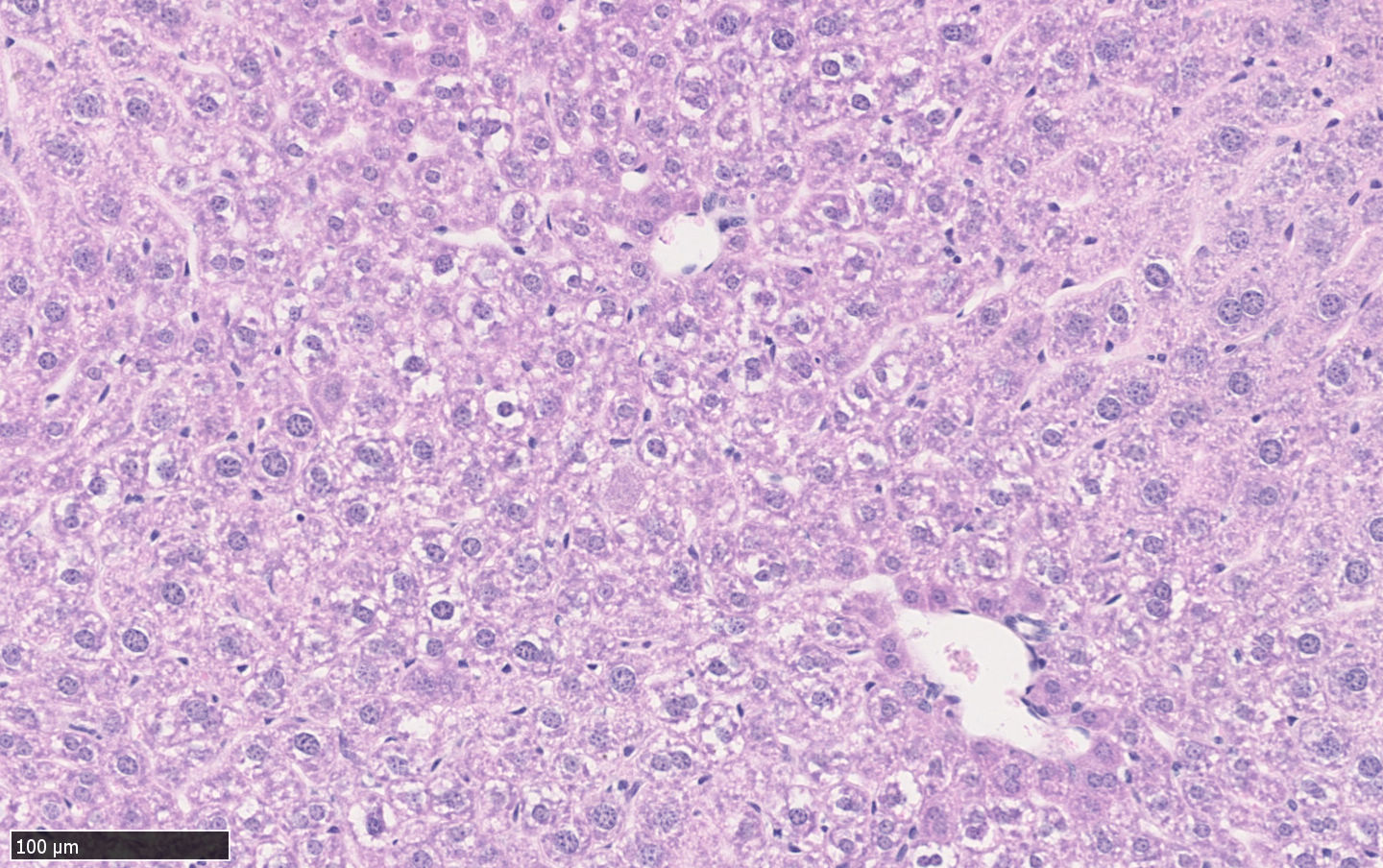

Supplement: Supplementary file 3 [file DataSheet8.ZIP › NASH SCORE-WT/WT11,12/7.jpg]

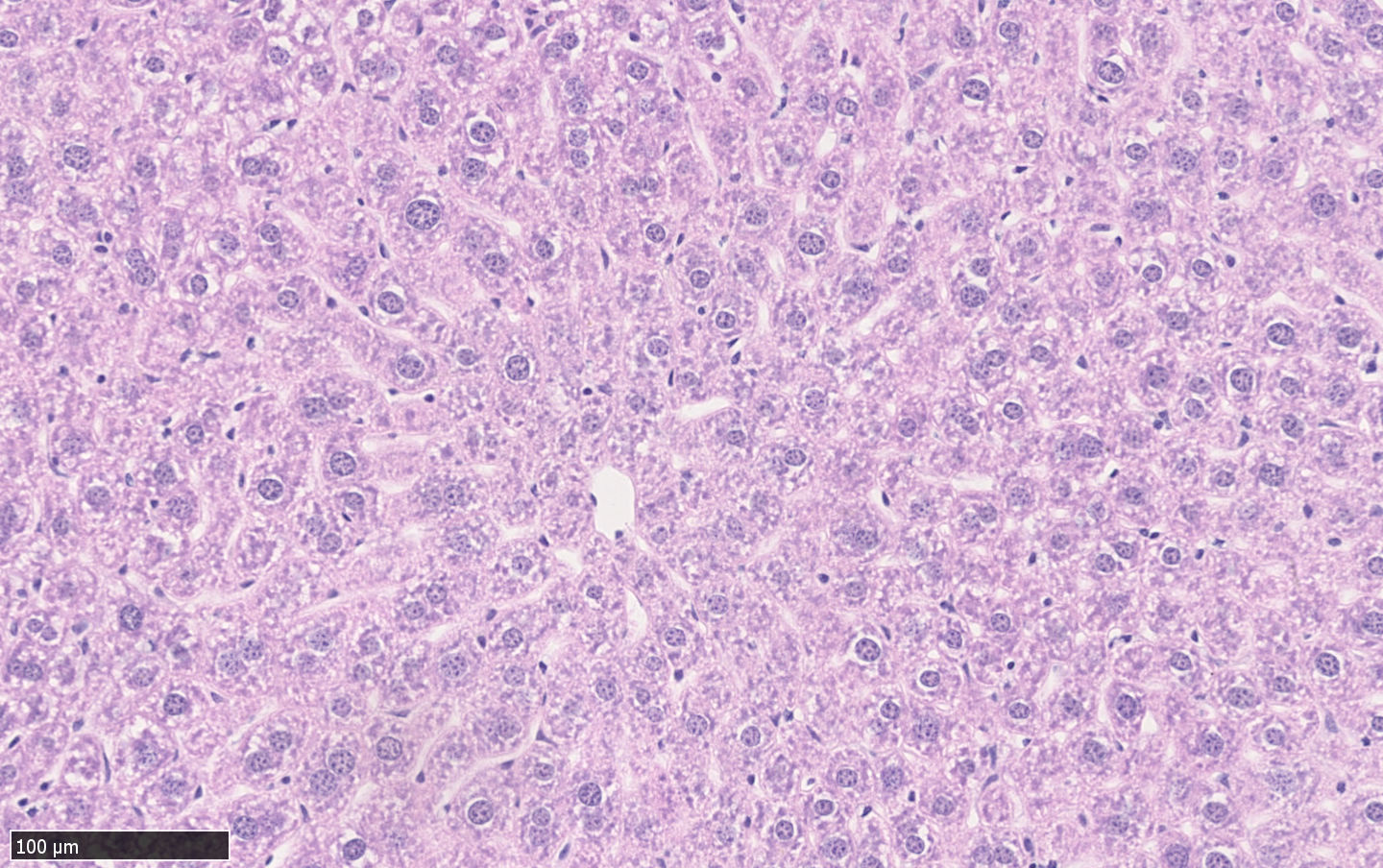

Supplement: Supplementary file 3 [file DataSheet8.ZIP › NASH SCORE-WT/WT11,12/8.jpg]

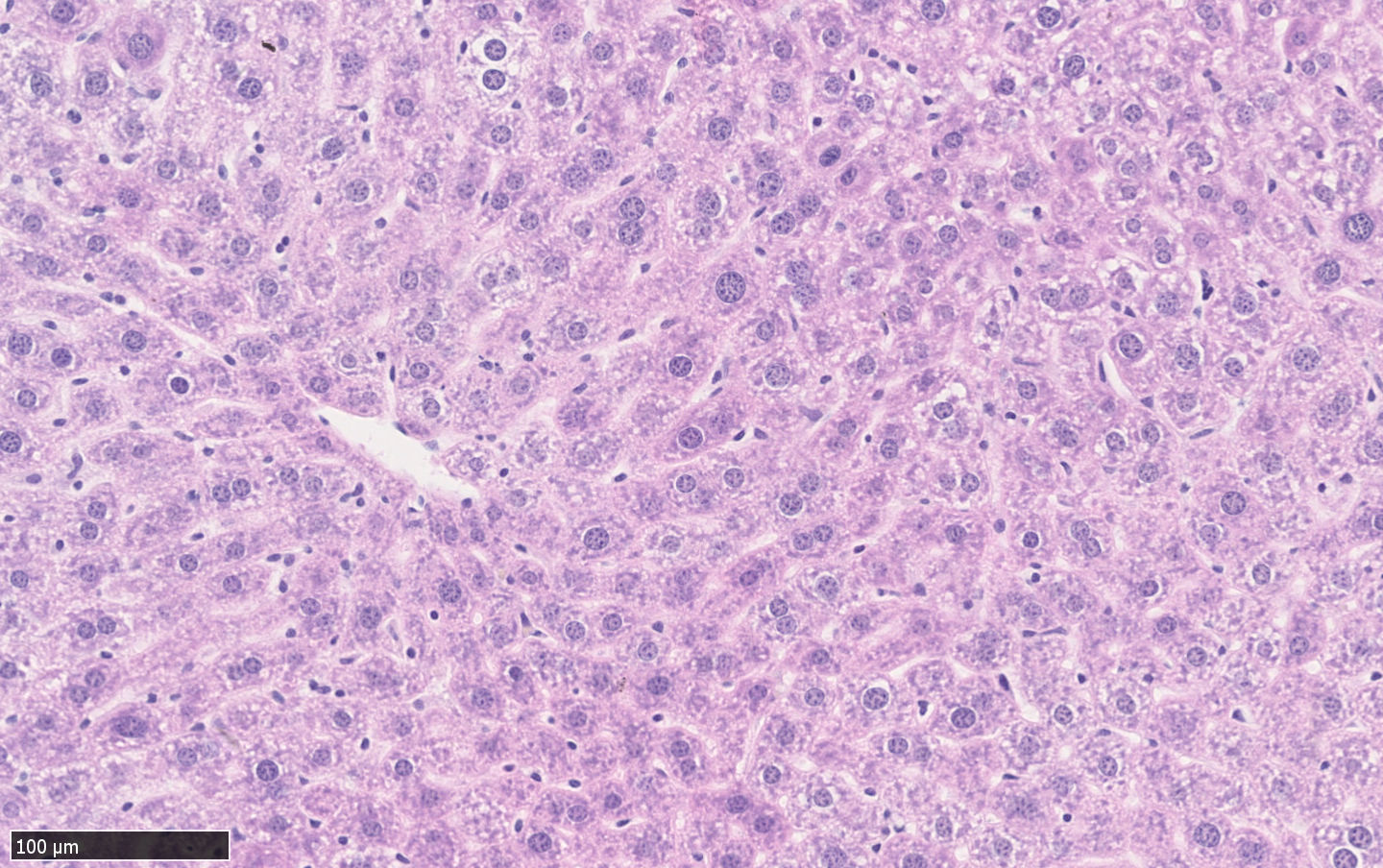

Supplement: Supplementary file 3 [file DataSheet8.ZIP › NASH SCORE-WT/WT11,12/9.jpg]

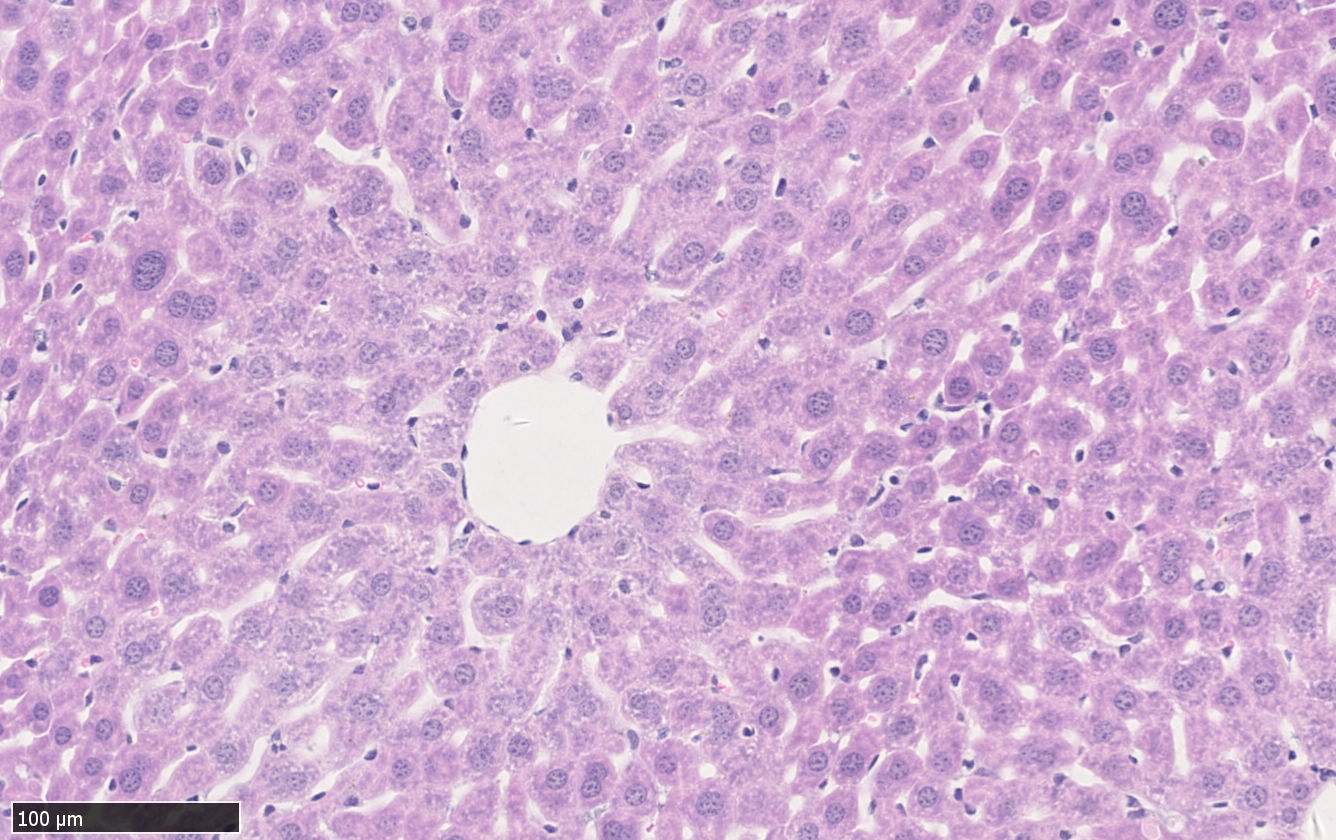

Supplement: Supplementary file 3 [file DataSheet8.ZIP › NASH SCORE-WT/WT14,15/1.jpg]

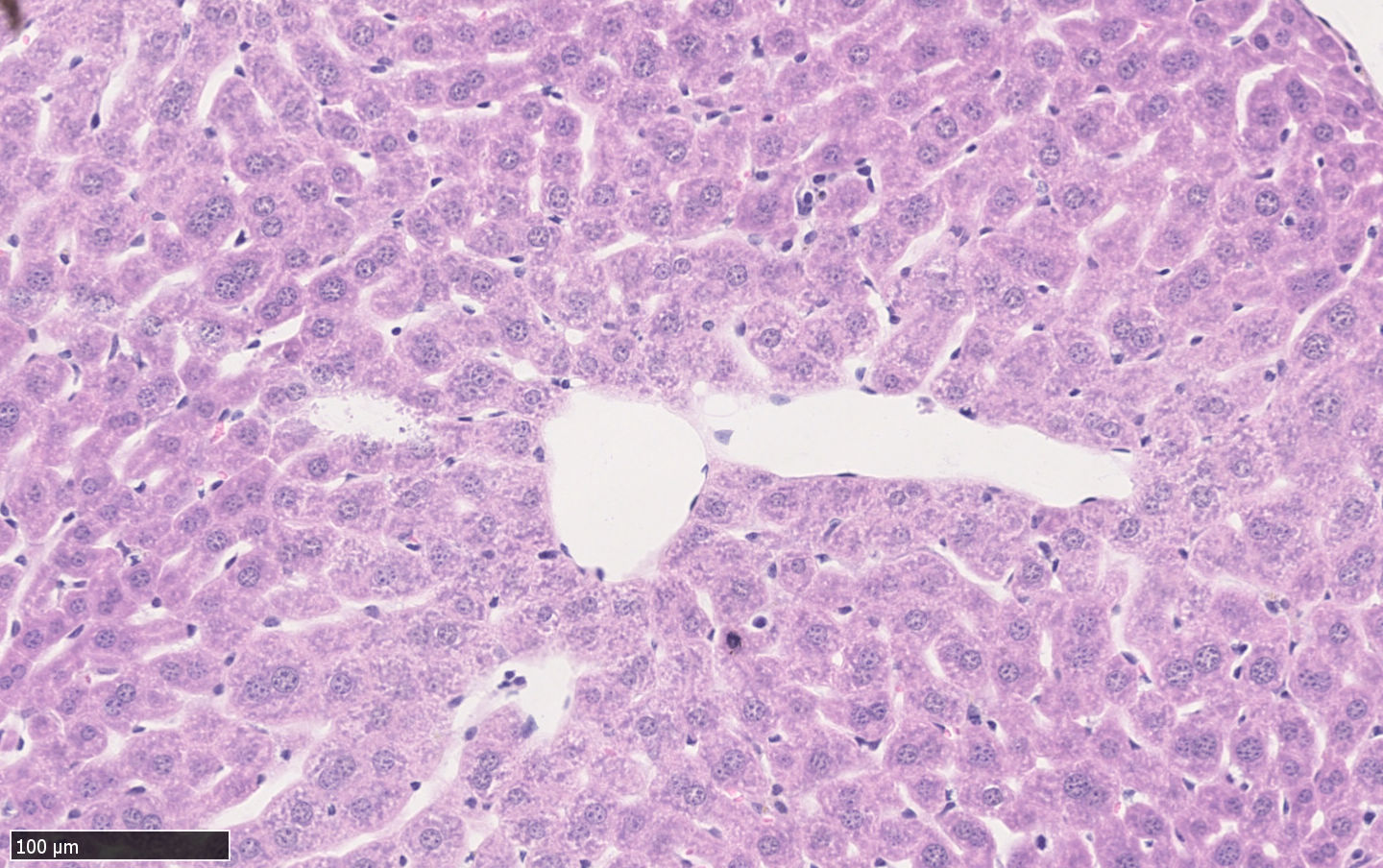

Supplement: Supplementary file 3 [file DataSheet8.ZIP › NASH SCORE-WT/WT14,15/10.jpg]

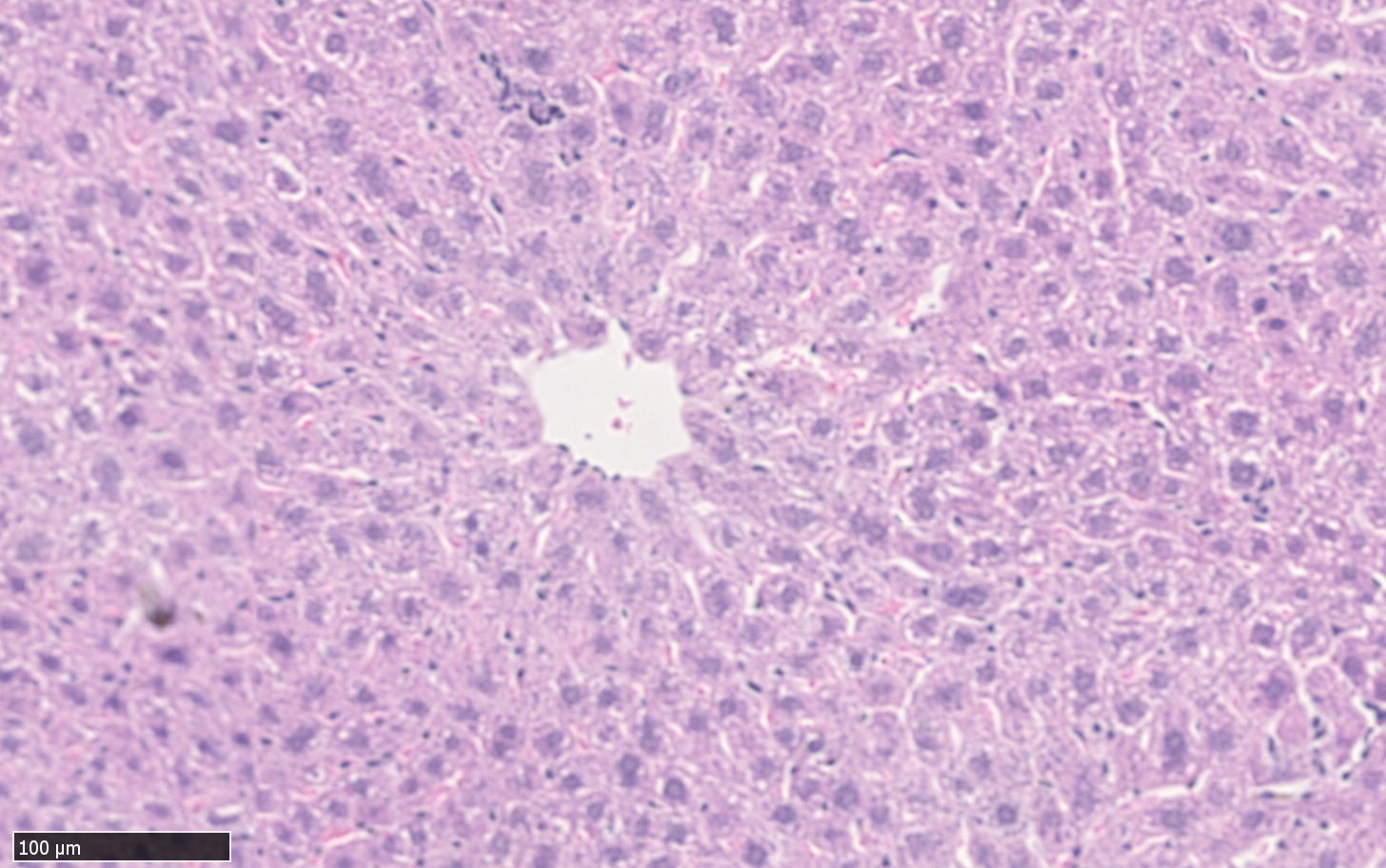

Supplement: Supplementary file 3 [file DataSheet8.ZIP › NASH SCORE-WT/WT14,15/11.jpg]

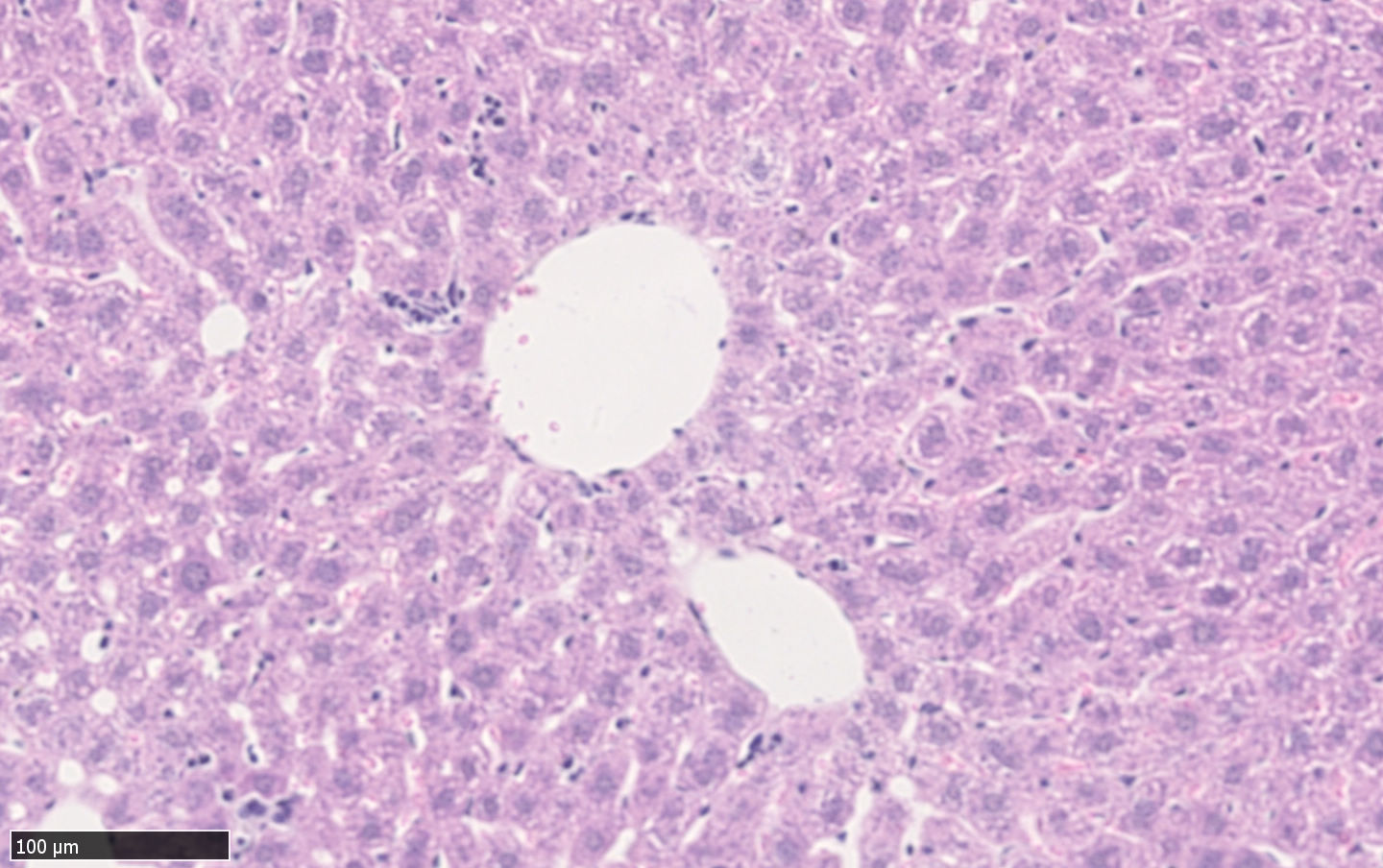

Supplement: Supplementary file 3 [file DataSheet8.ZIP › NASH SCORE-WT/WT14,15/12.jpg]

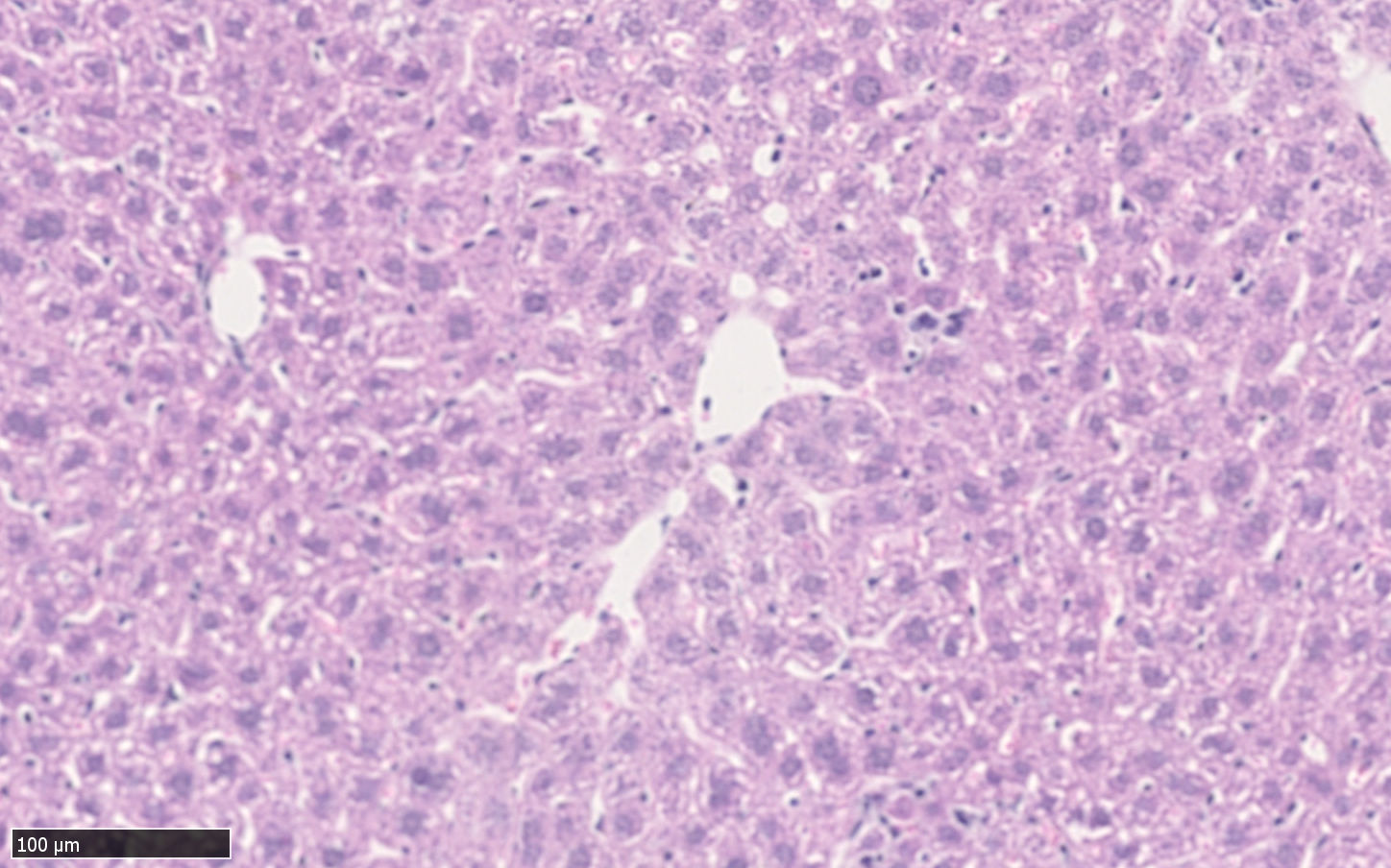

Supplement: Supplementary file 3 [file DataSheet8.ZIP › NASH SCORE-WT/WT14,15/13.jpg]

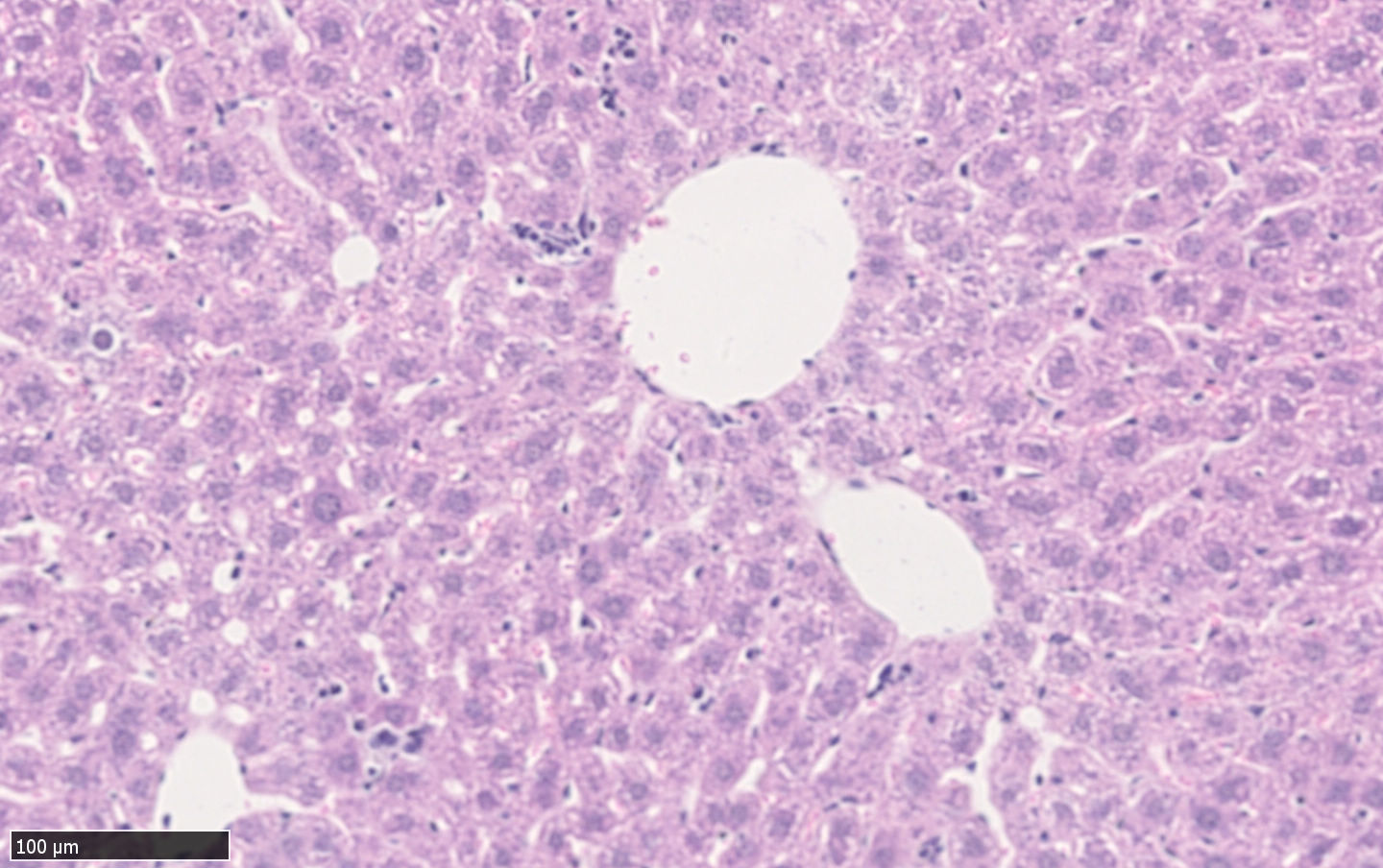

Supplement: Supplementary file 3 [file DataSheet8.ZIP › NASH SCORE-WT/WT14,15/14.jpg]

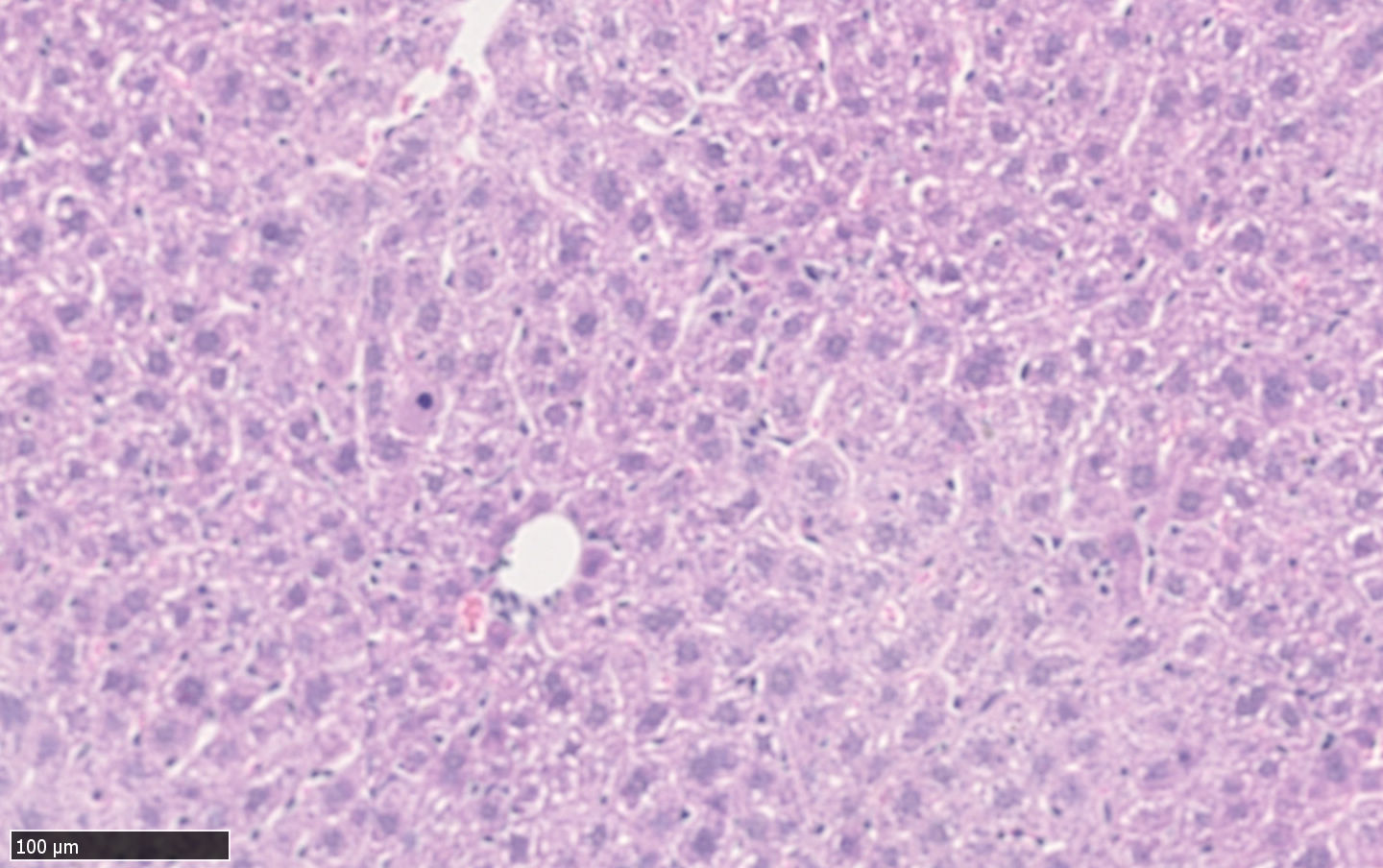

Supplement: Supplementary file 3 [file DataSheet8.ZIP › NASH SCORE-WT/WT14,15/15.jpg]

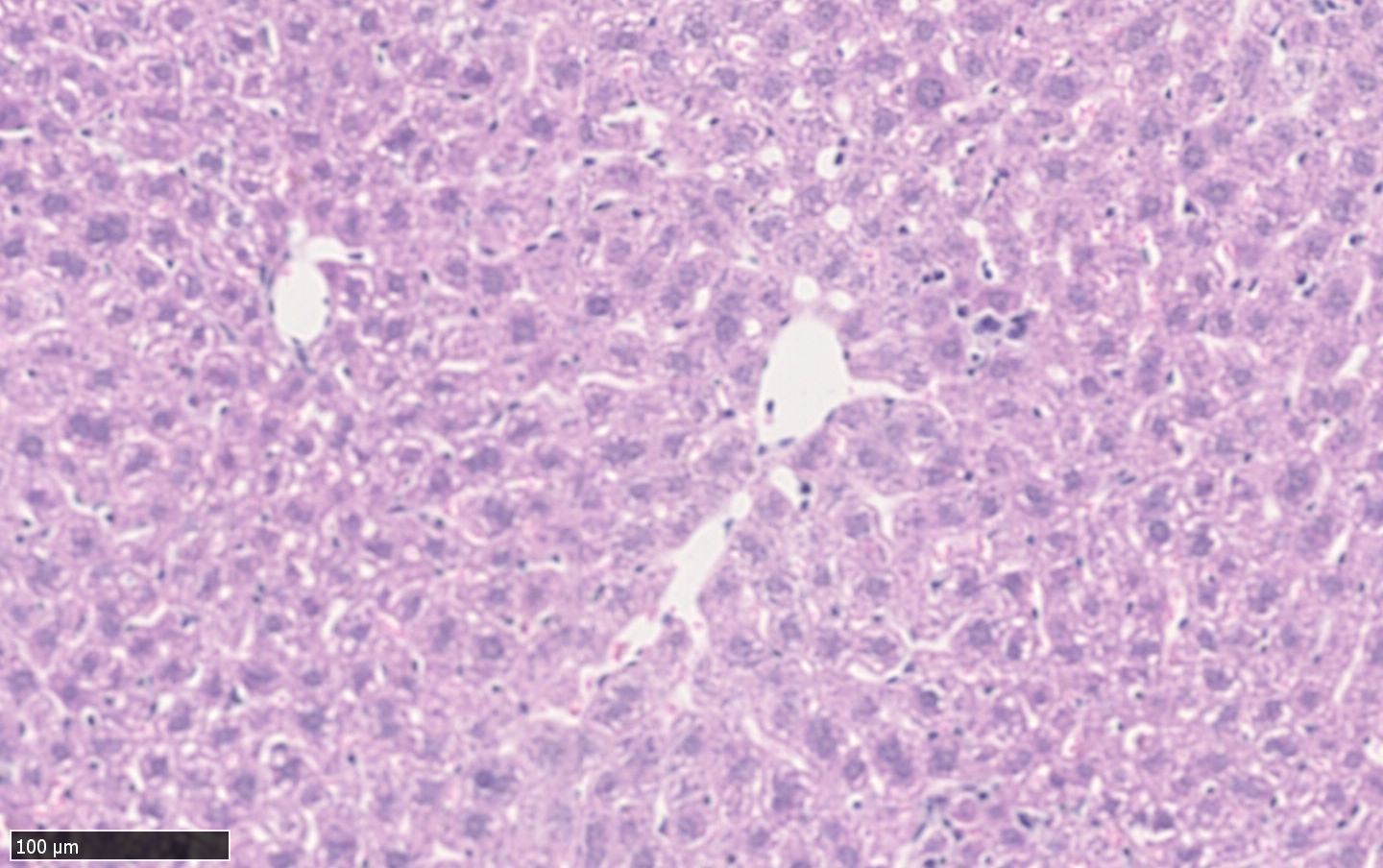

Supplement: Supplementary file 3 [file DataSheet8.ZIP › NASH SCORE-WT/WT14,15/16.jpg]

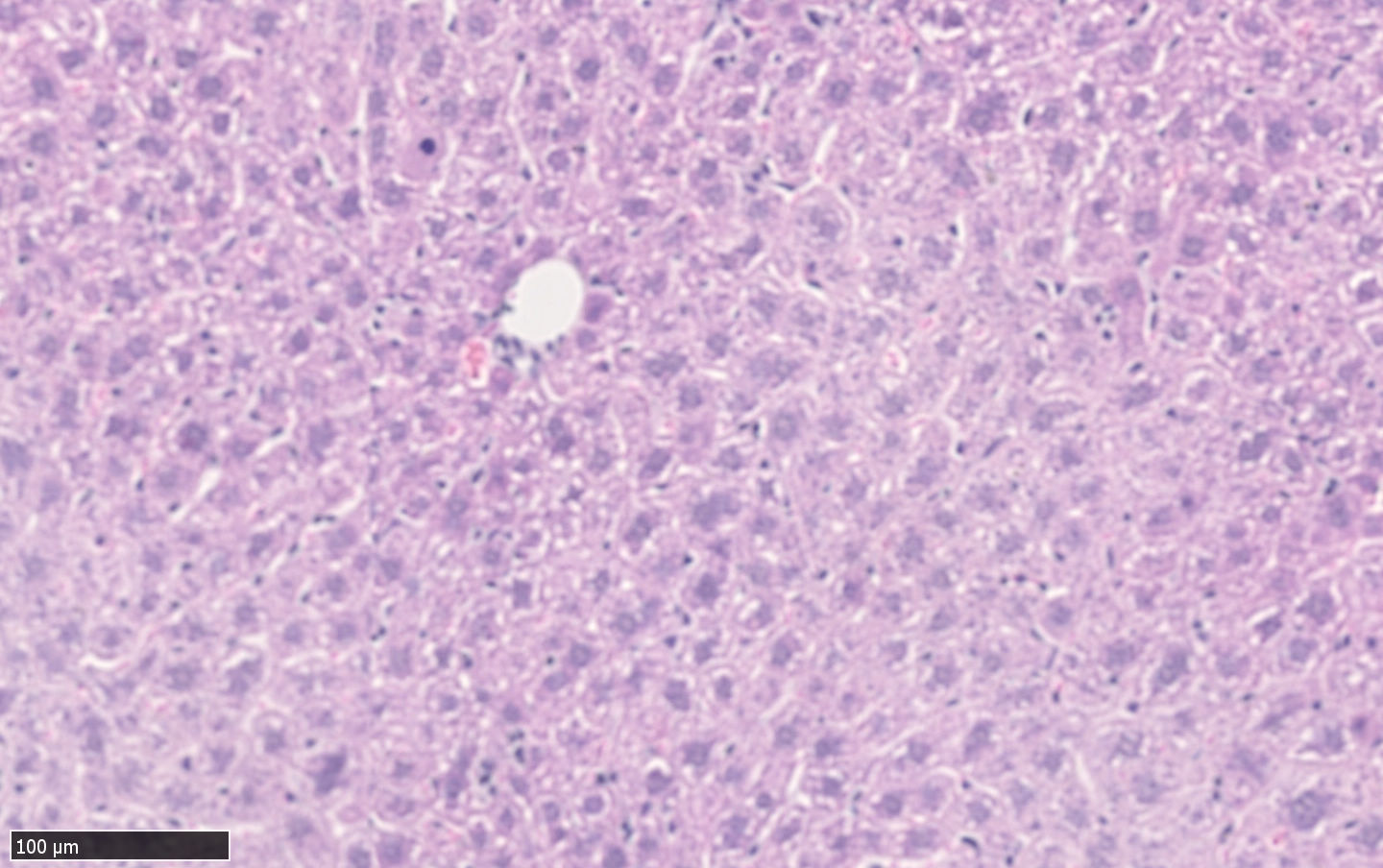

Supplement: Supplementary file 3 [file DataSheet8.ZIP › NASH SCORE-WT/WT14,15/17.jpg]

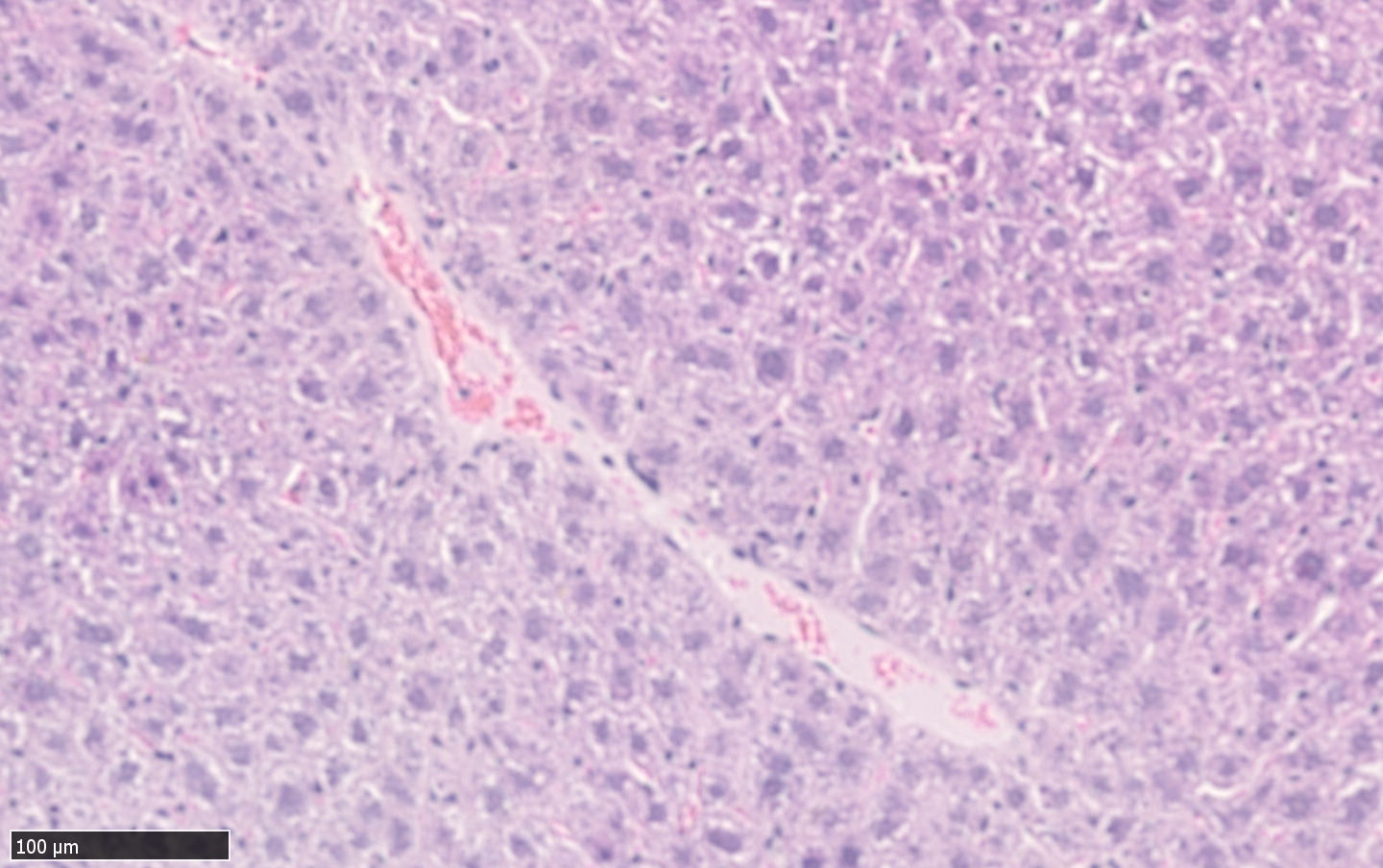

Supplement: Supplementary file 3 [file DataSheet8.ZIP › NASH SCORE-WT/WT14,15/18.jpg]

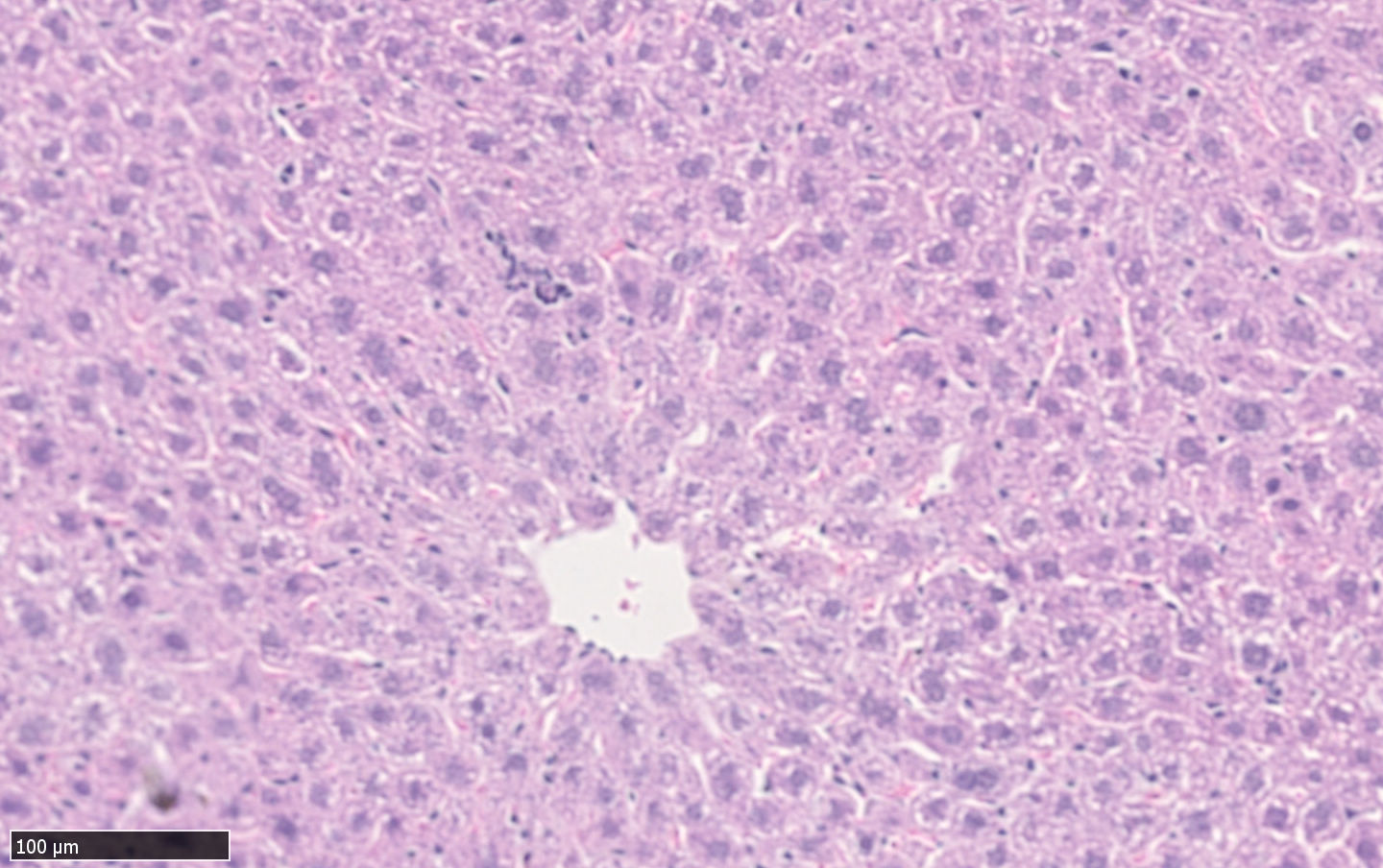

Supplement: Supplementary file 3 [file DataSheet8.ZIP › NASH SCORE-WT/WT14,15/19.jpg]

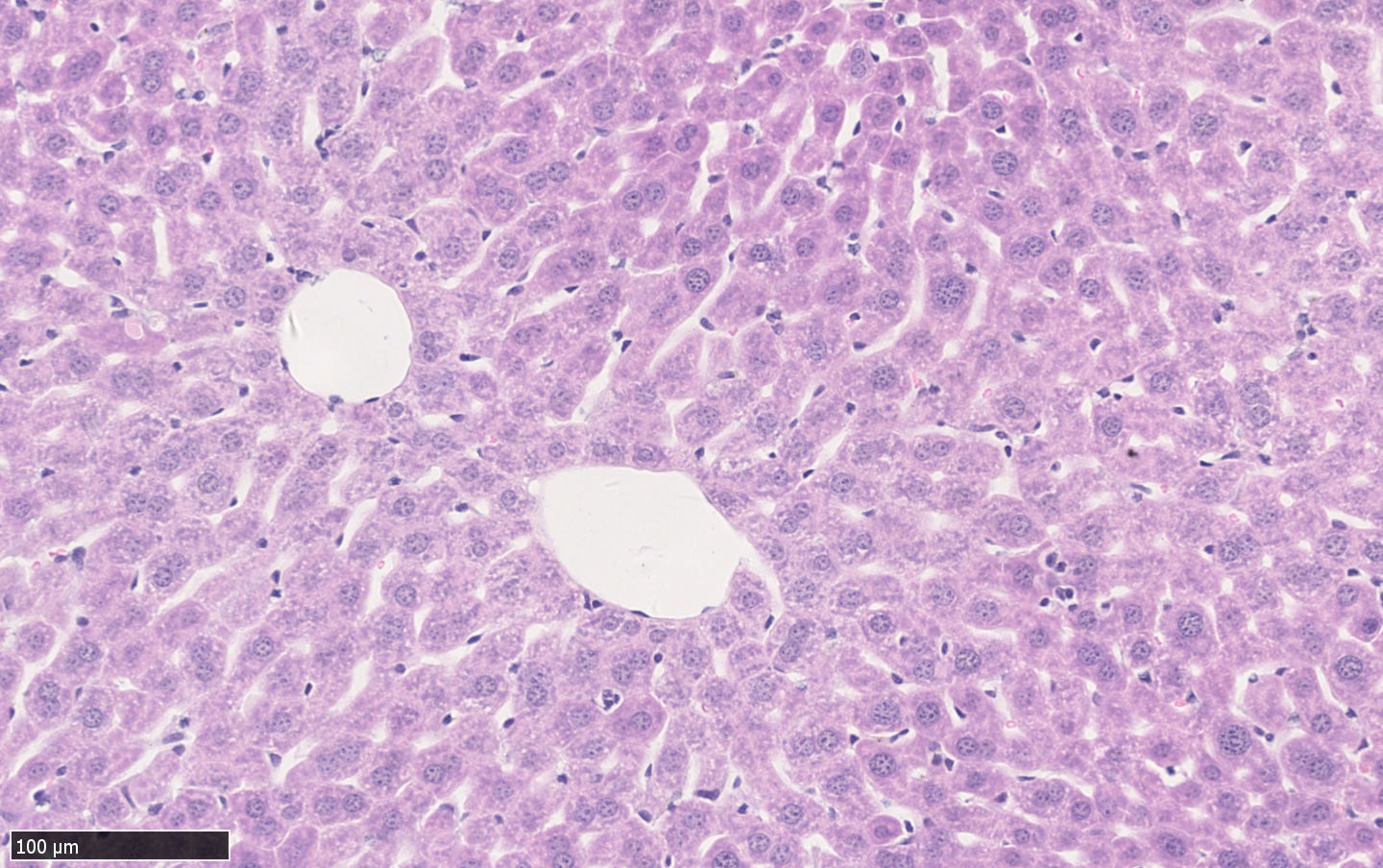

Supplement: Supplementary file 3 [file DataSheet8.ZIP › NASH SCORE-WT/WT14,15/2.jpg]

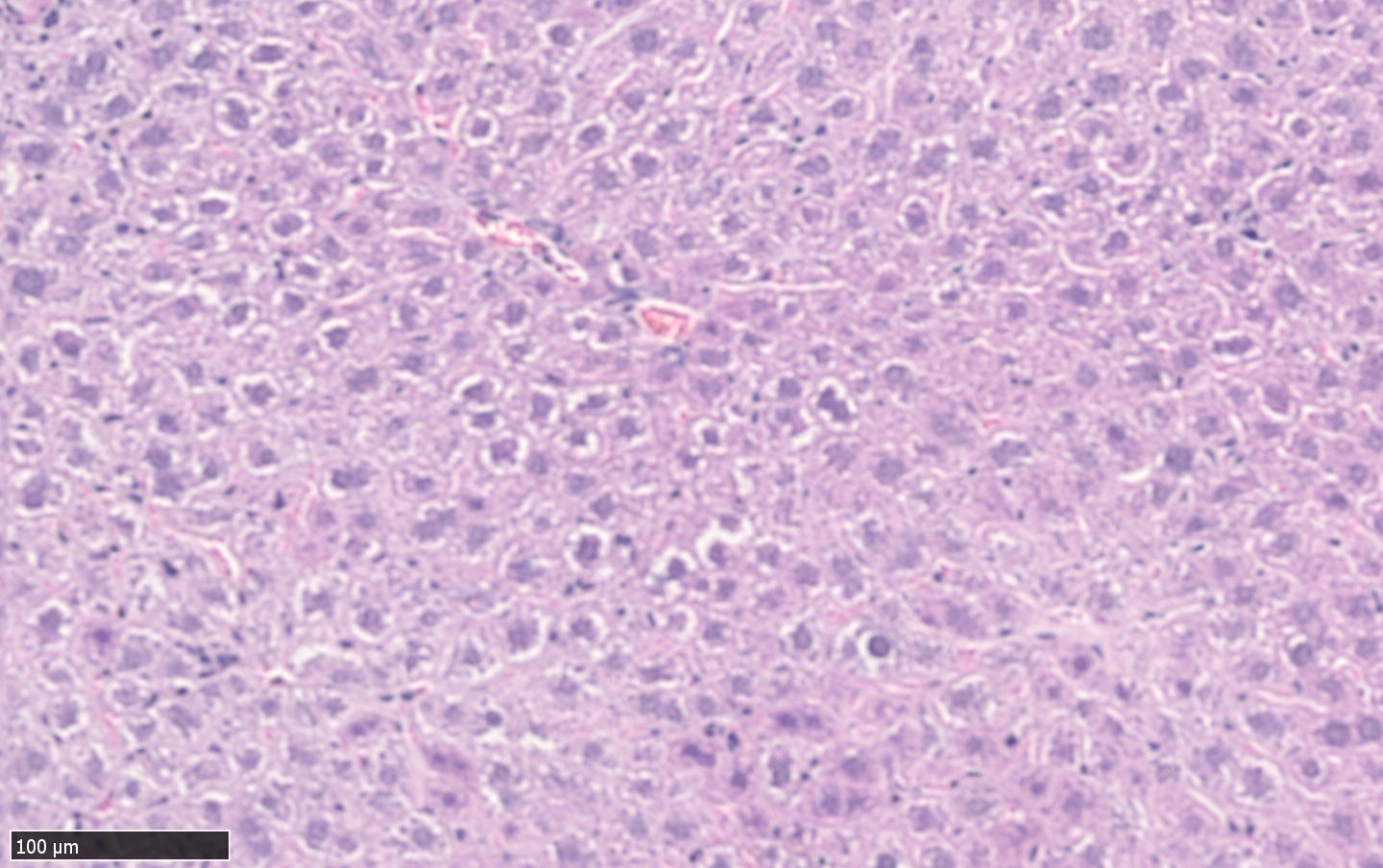

Supplement: Supplementary file 3 [file DataSheet8.ZIP › NASH SCORE-WT/WT14,15/20.jpg]

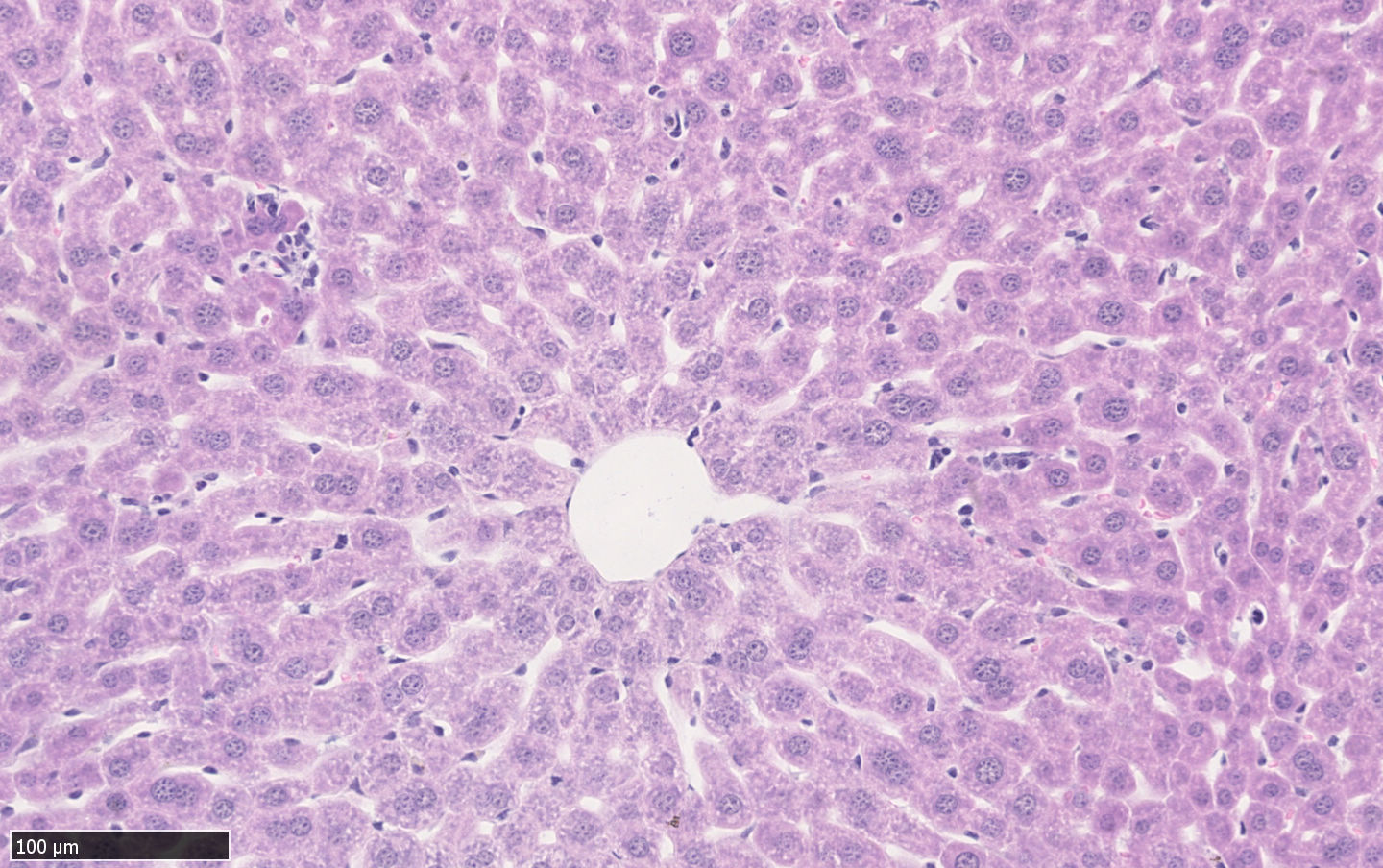

Supplement: Supplementary file 3 [file DataSheet8.ZIP › NASH SCORE-WT/WT14,15/3.jpg]

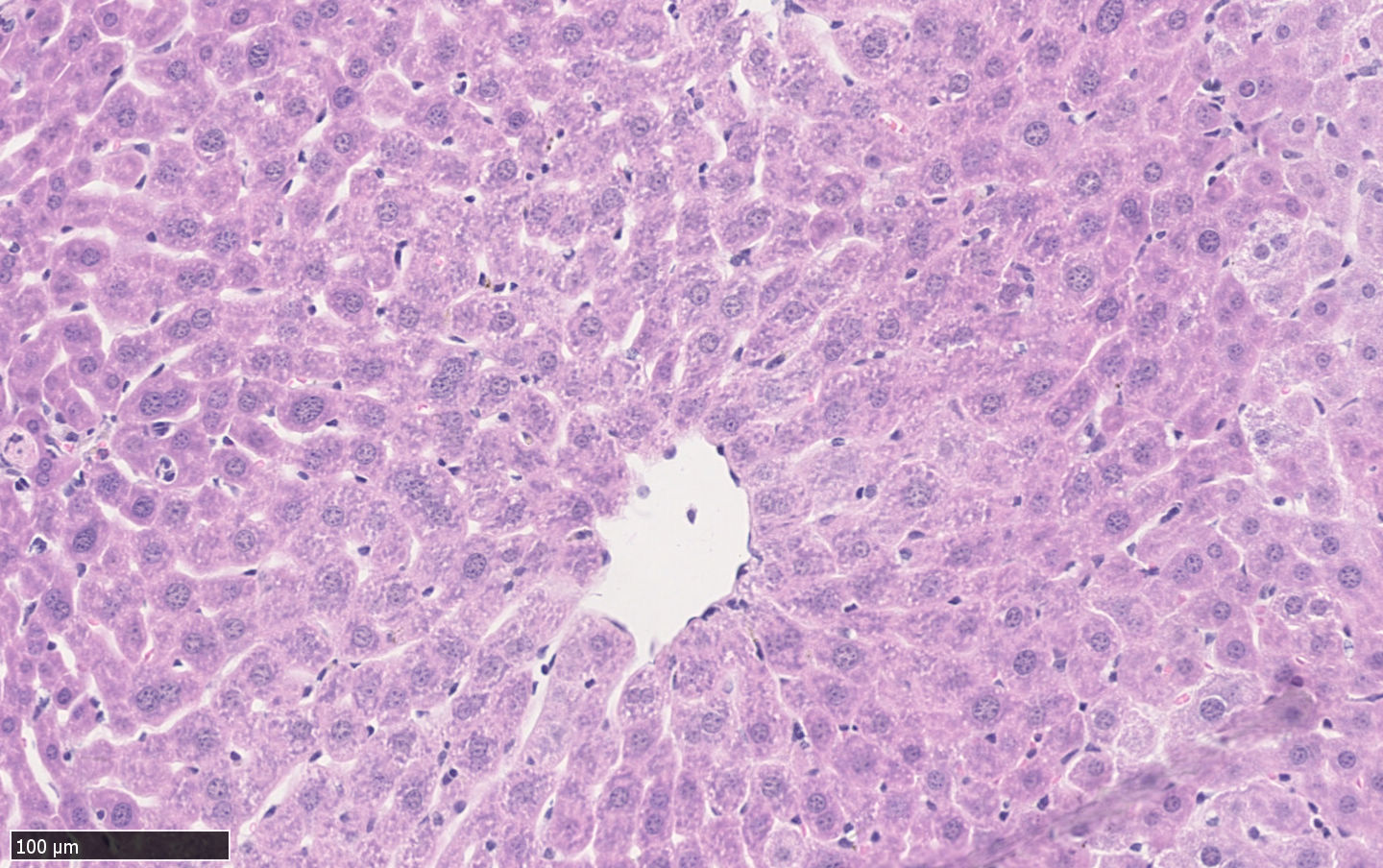

Supplement: Supplementary file 3 [file DataSheet8.ZIP › NASH SCORE-WT/WT14,15/4.jpg]

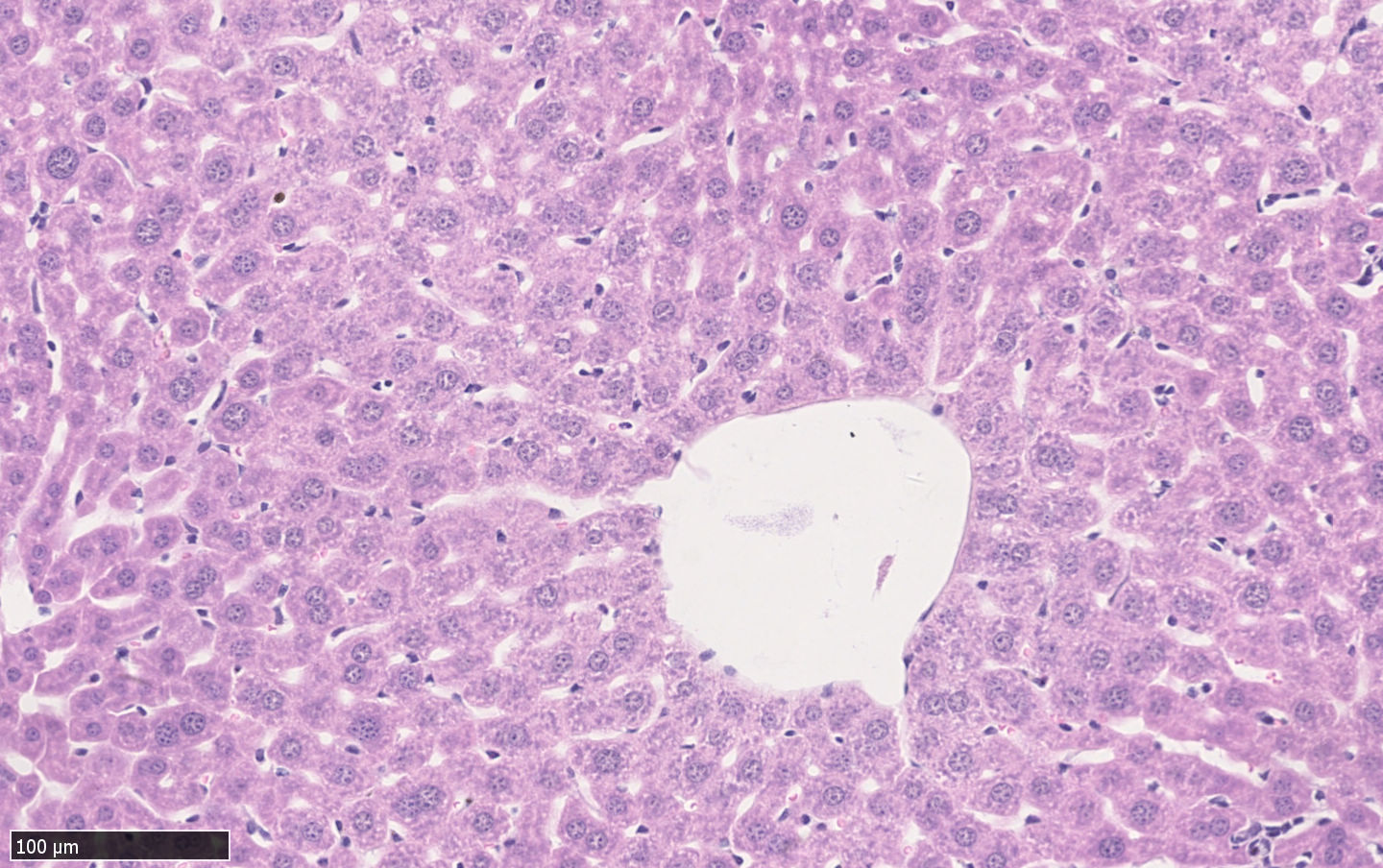

Supplement: Supplementary file 3 [file DataSheet8.ZIP › NASH SCORE-WT/WT14,15/5.jpg]

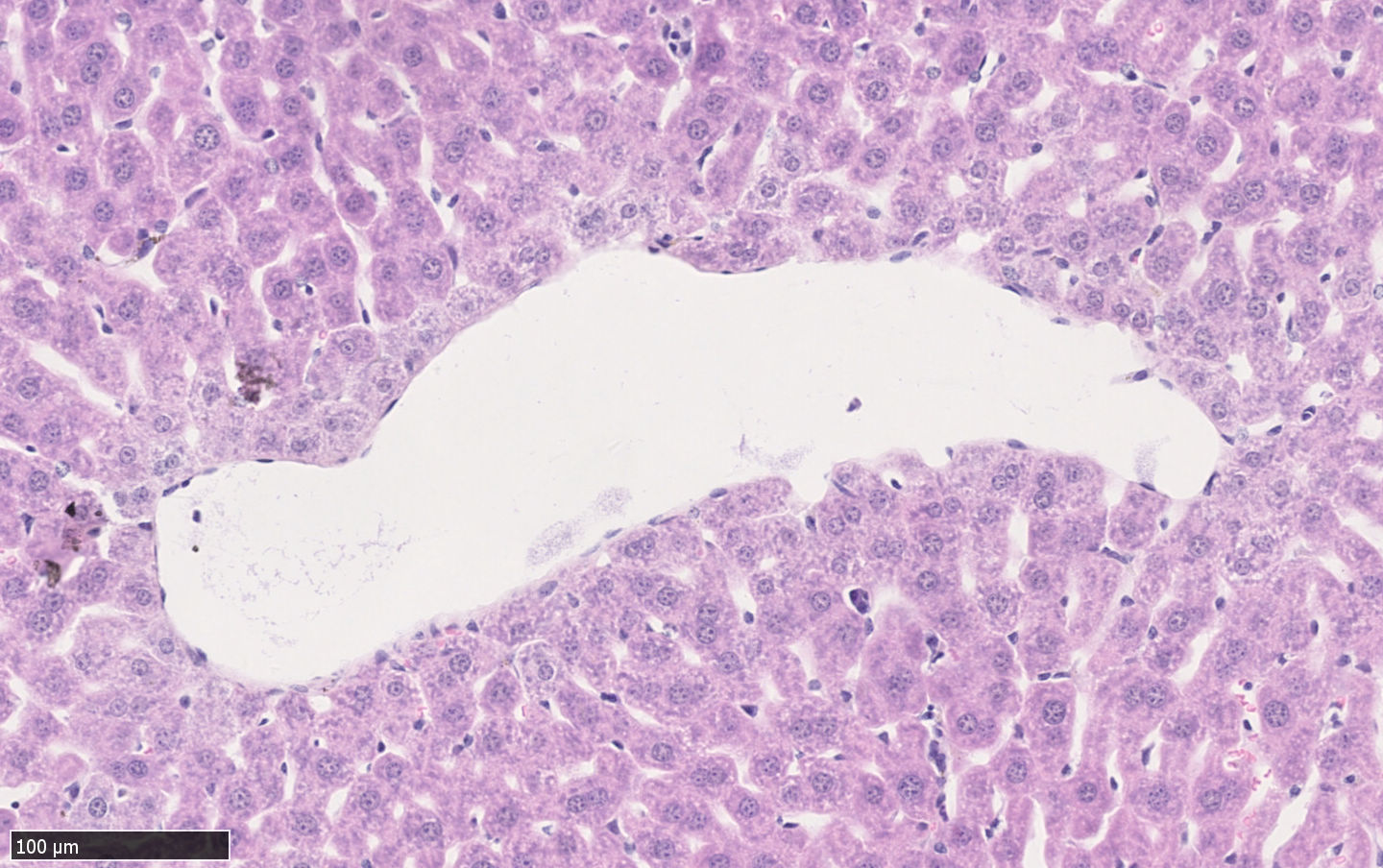

Supplement: Supplementary file 3 [file DataSheet8.ZIP › NASH SCORE-WT/WT14,15/6.jpg]

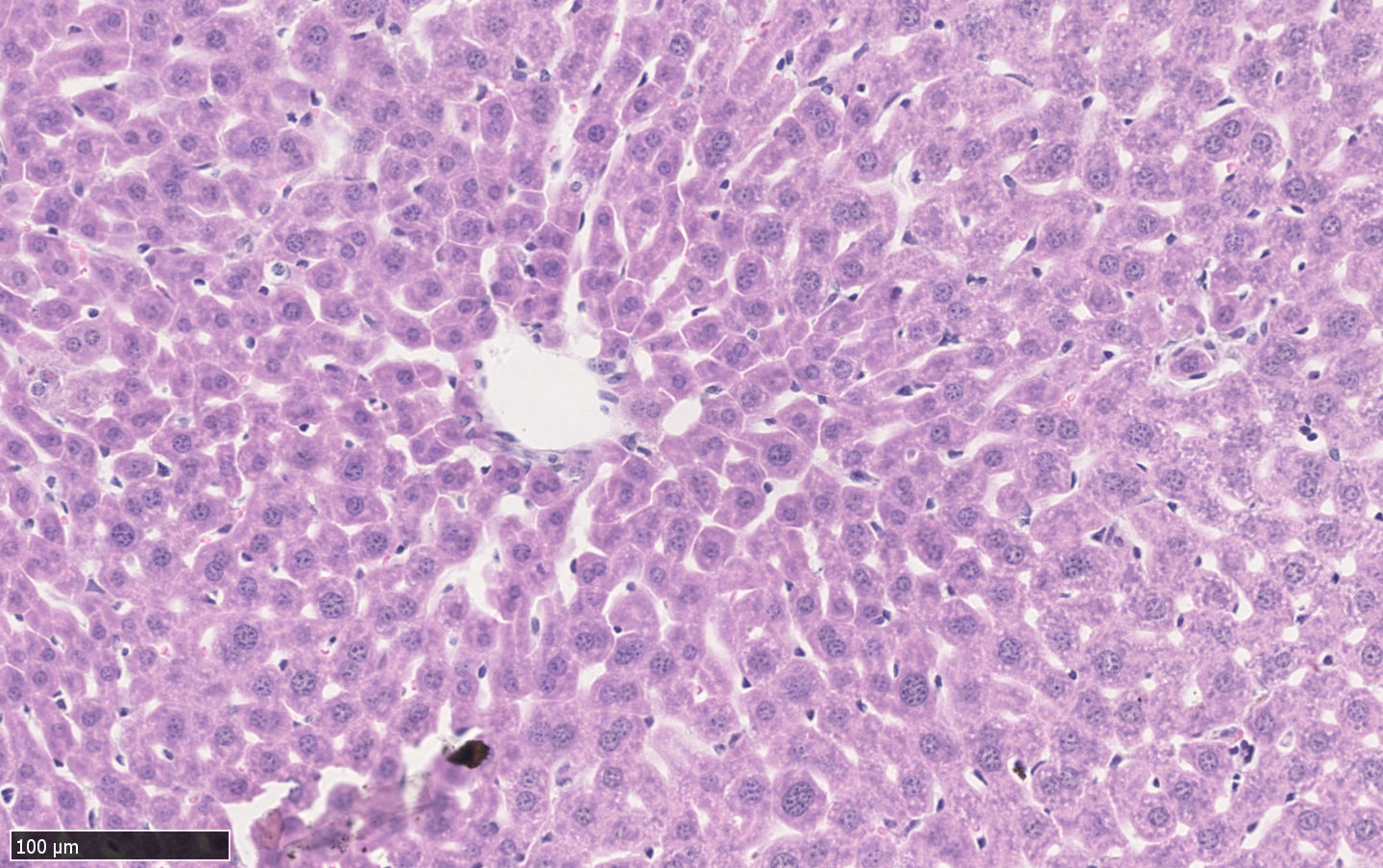

Supplement: Supplementary file 3 [file DataSheet8.ZIP › NASH SCORE-WT/WT14,15/7.jpg]

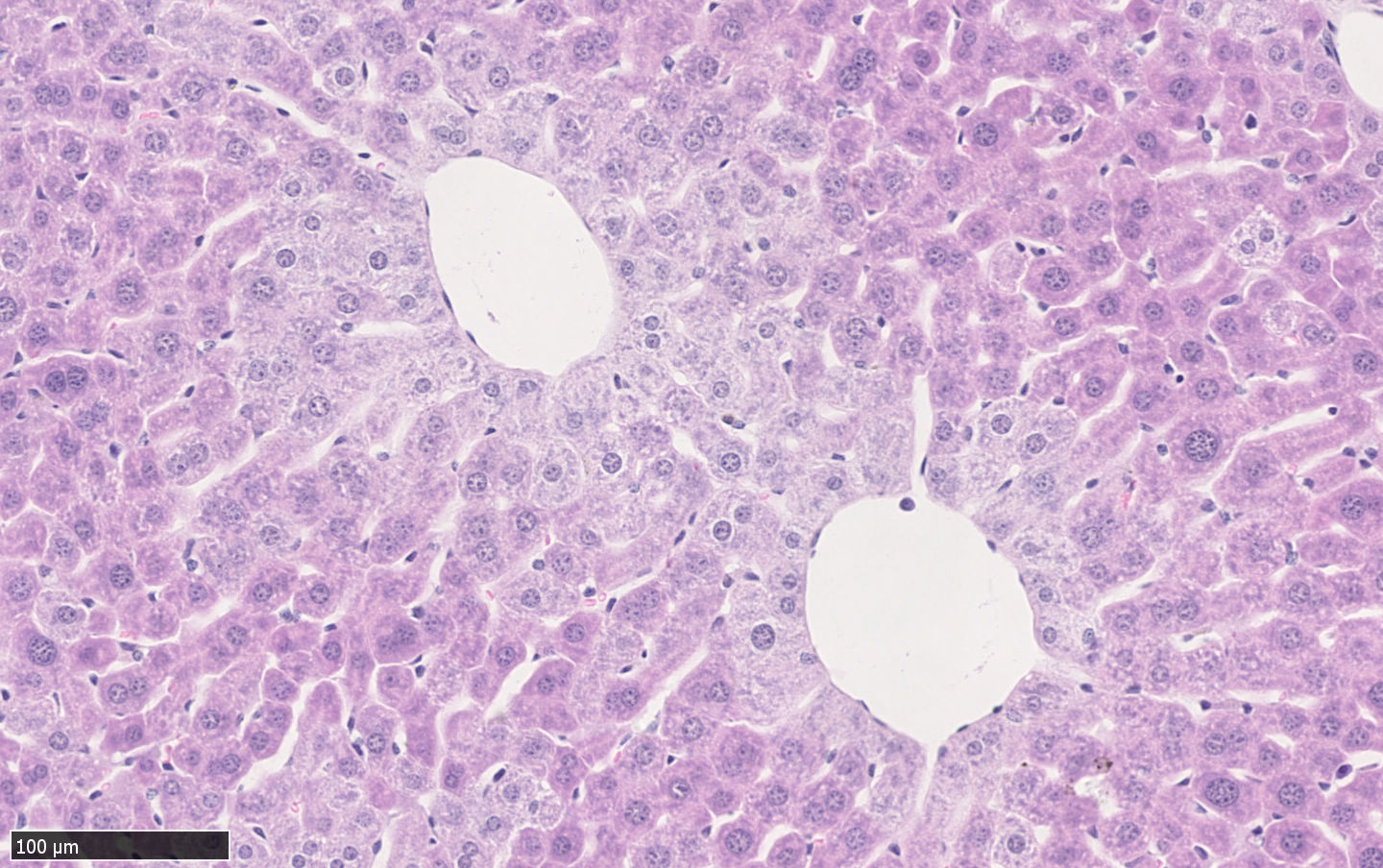

Supplement: Supplementary file 3 [file DataSheet8.ZIP › NASH SCORE-WT/WT14,15/8.jpg]

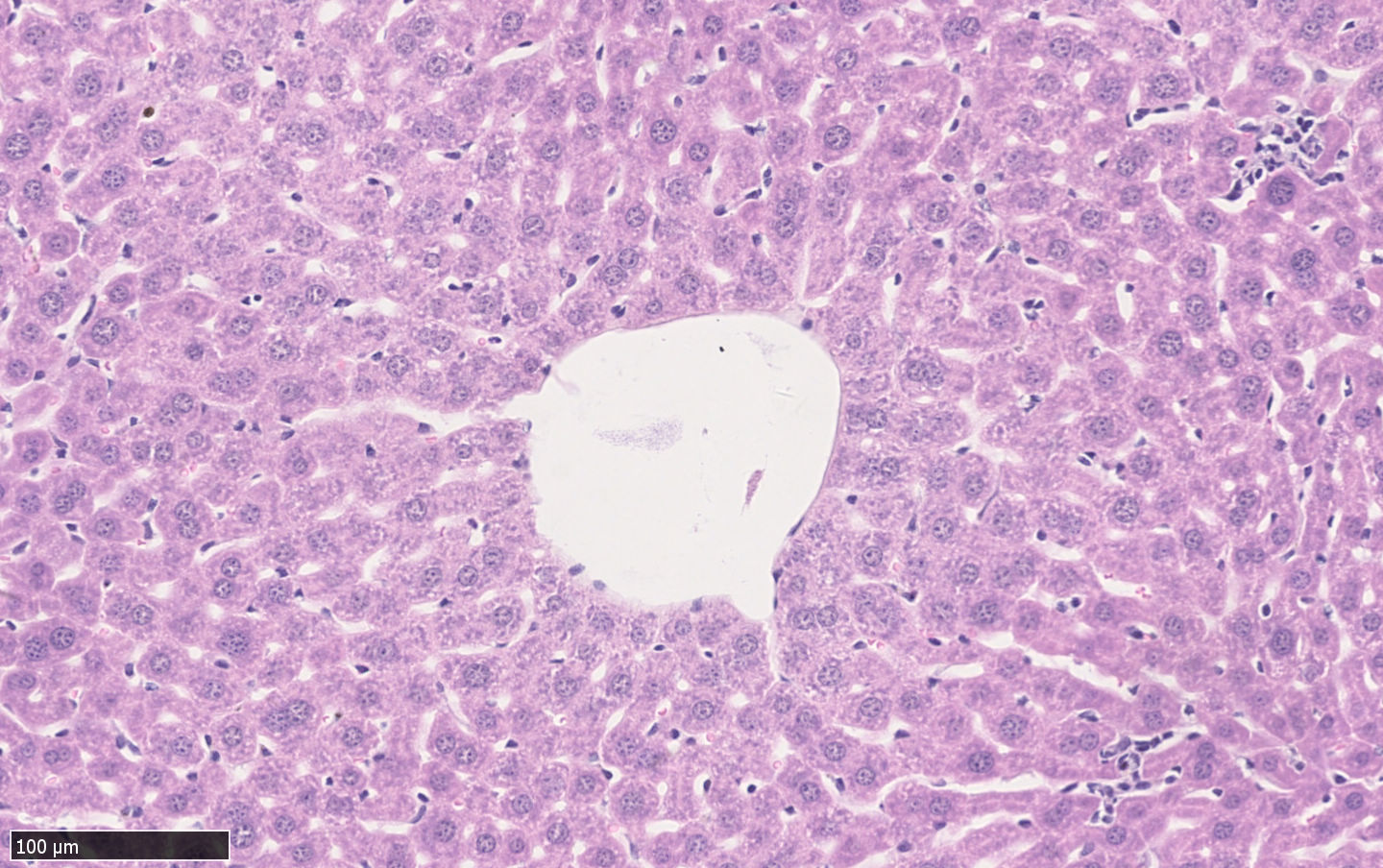

Supplement: Supplementary file 3 [file DataSheet8.ZIP › NASH SCORE-WT/WT14,15/9.jpg]

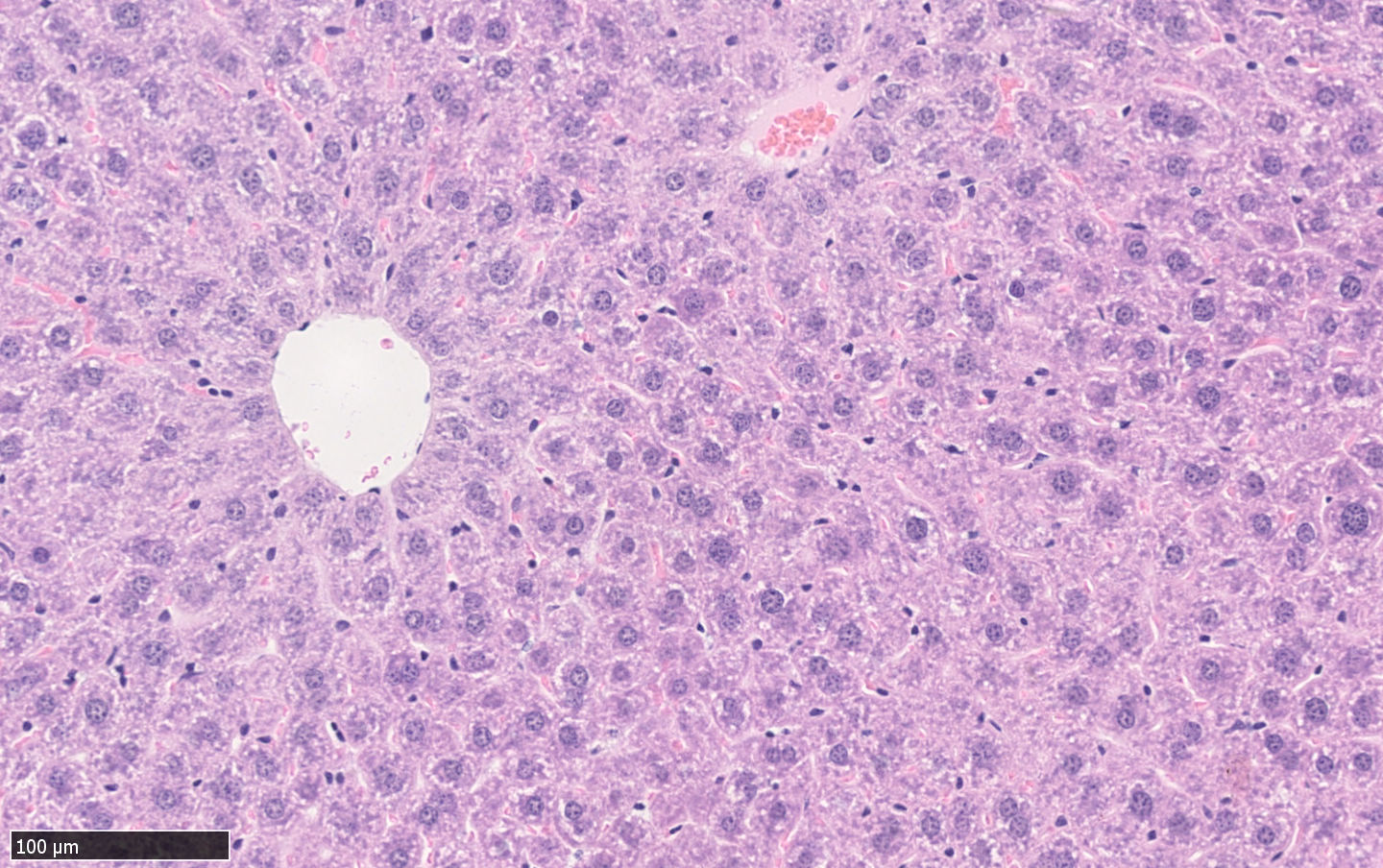

Supplement: Supplementary file 3 [file DataSheet8.ZIP › NASH SCORE-WT/WT17,18+/1.jpg]

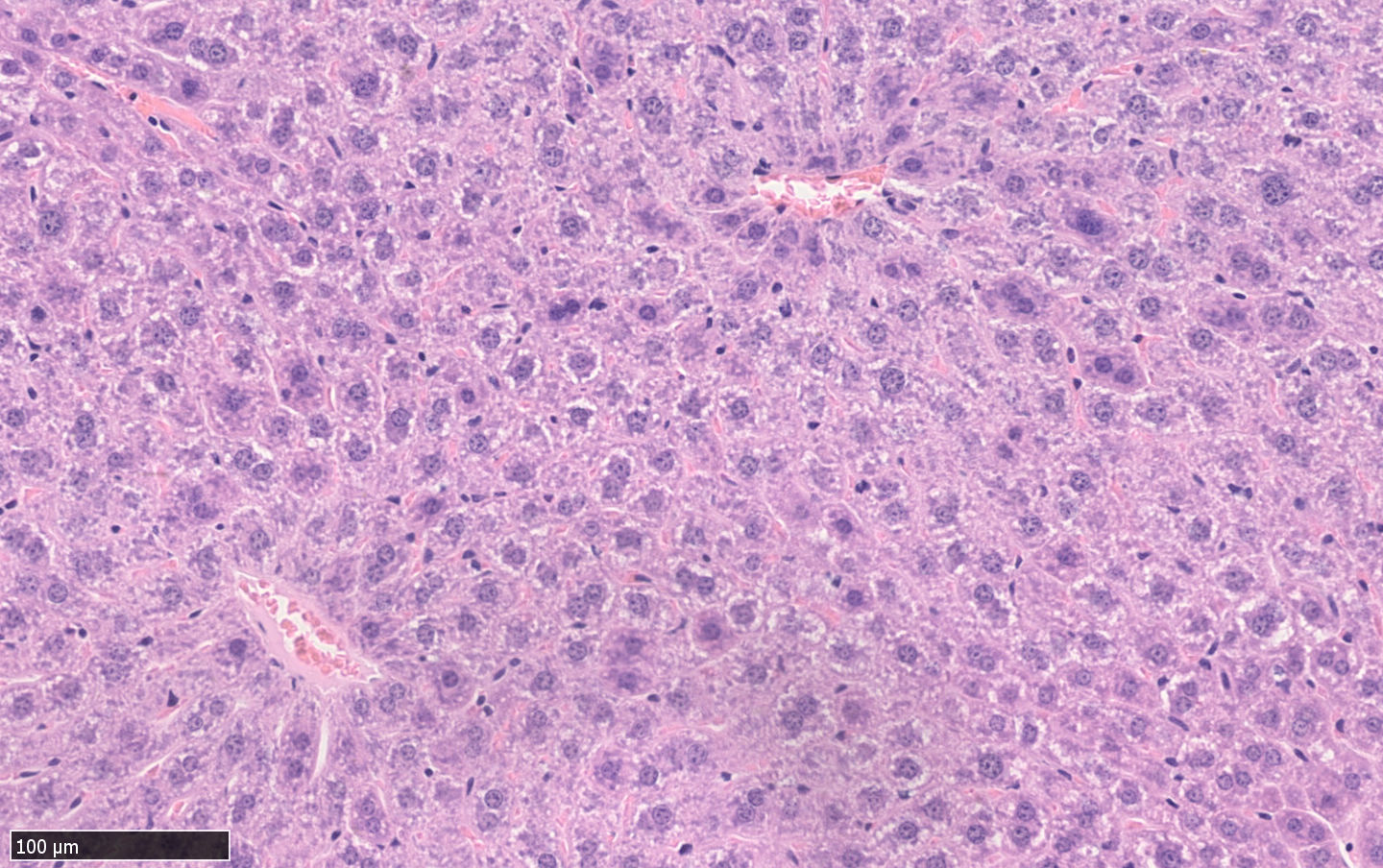

Supplement: Supplementary file 3 [file DataSheet8.ZIP › NASH SCORE-WT/WT17,18+/10.jpg]

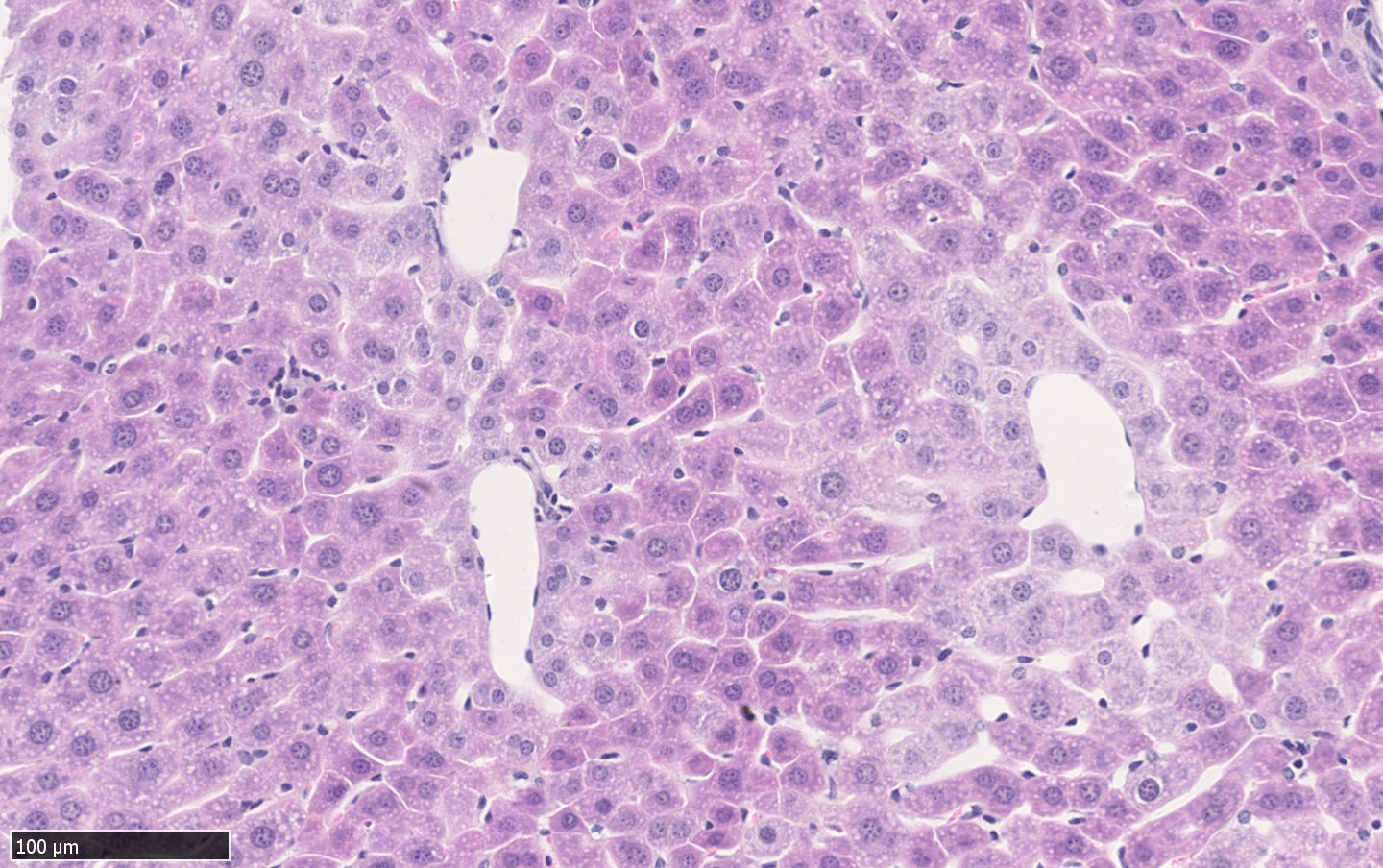

Supplement: Supplementary file 3 [file DataSheet8.ZIP › NASH SCORE-WT/WT17,18+/11.jpg]

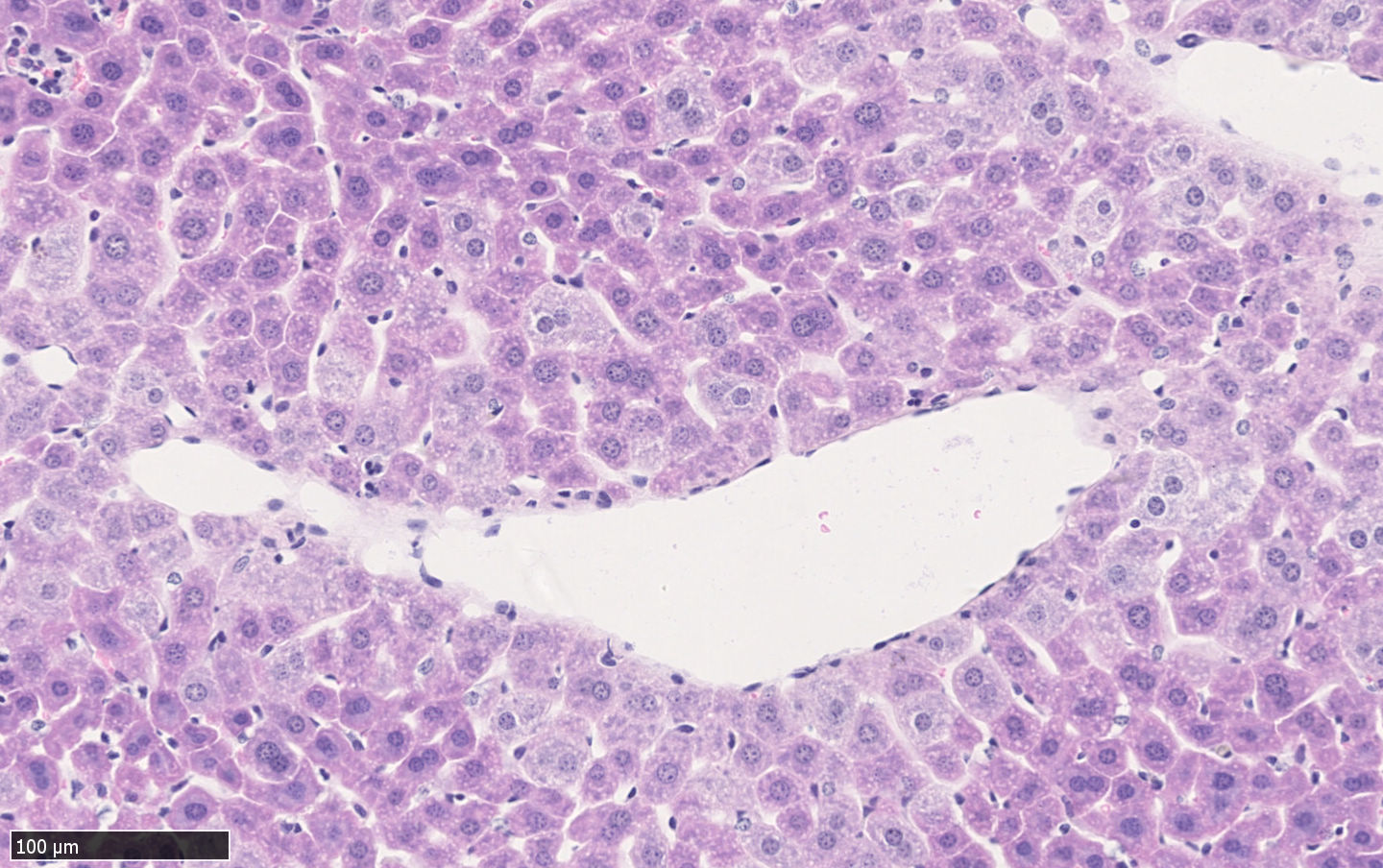

Supplement: Supplementary file 3 [file DataSheet8.ZIP › NASH SCORE-WT/WT17,18+/12.jpg]

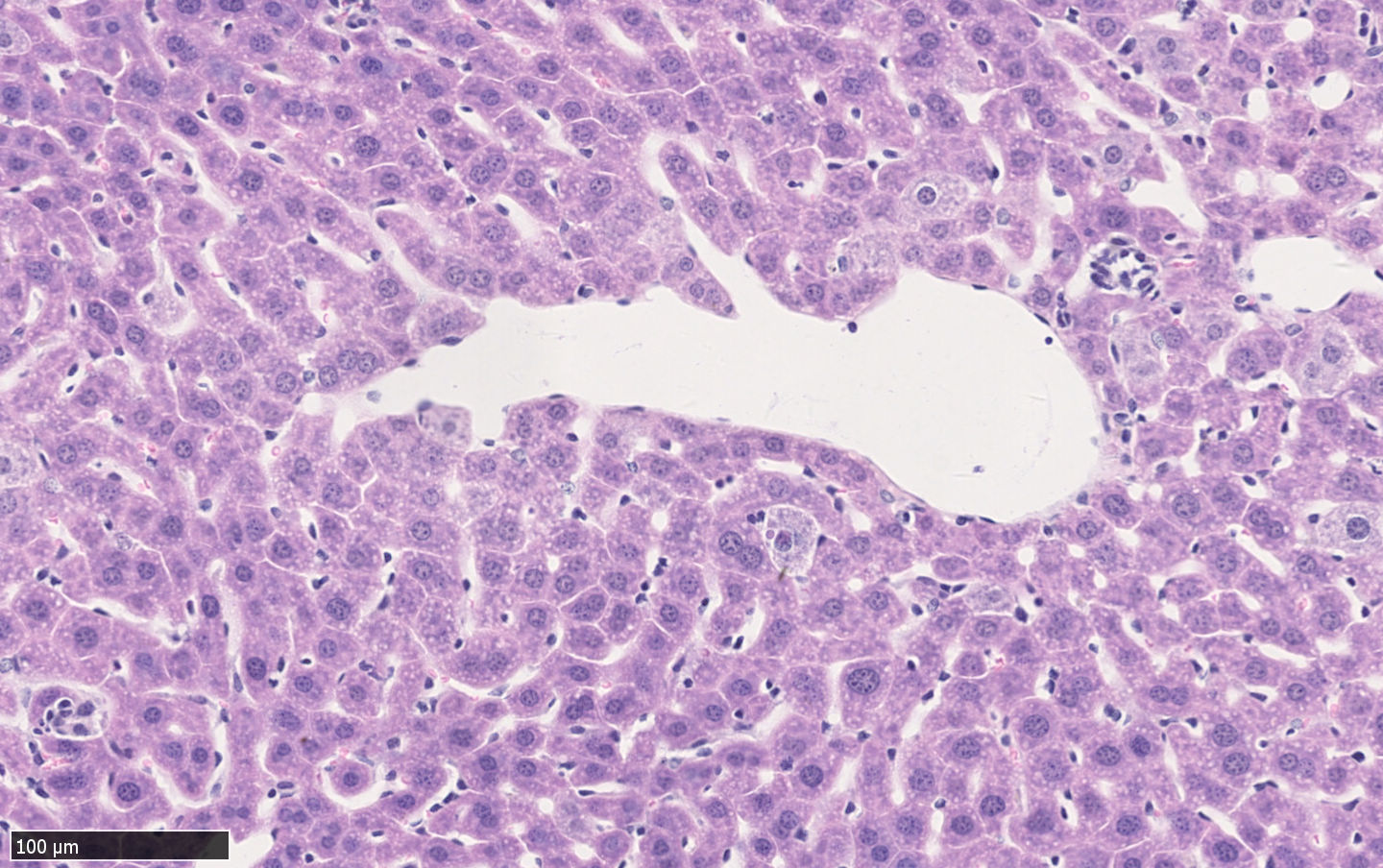

Supplement: Supplementary file 3 [file DataSheet8.ZIP › NASH SCORE-WT/WT17,18+/13.jpg]

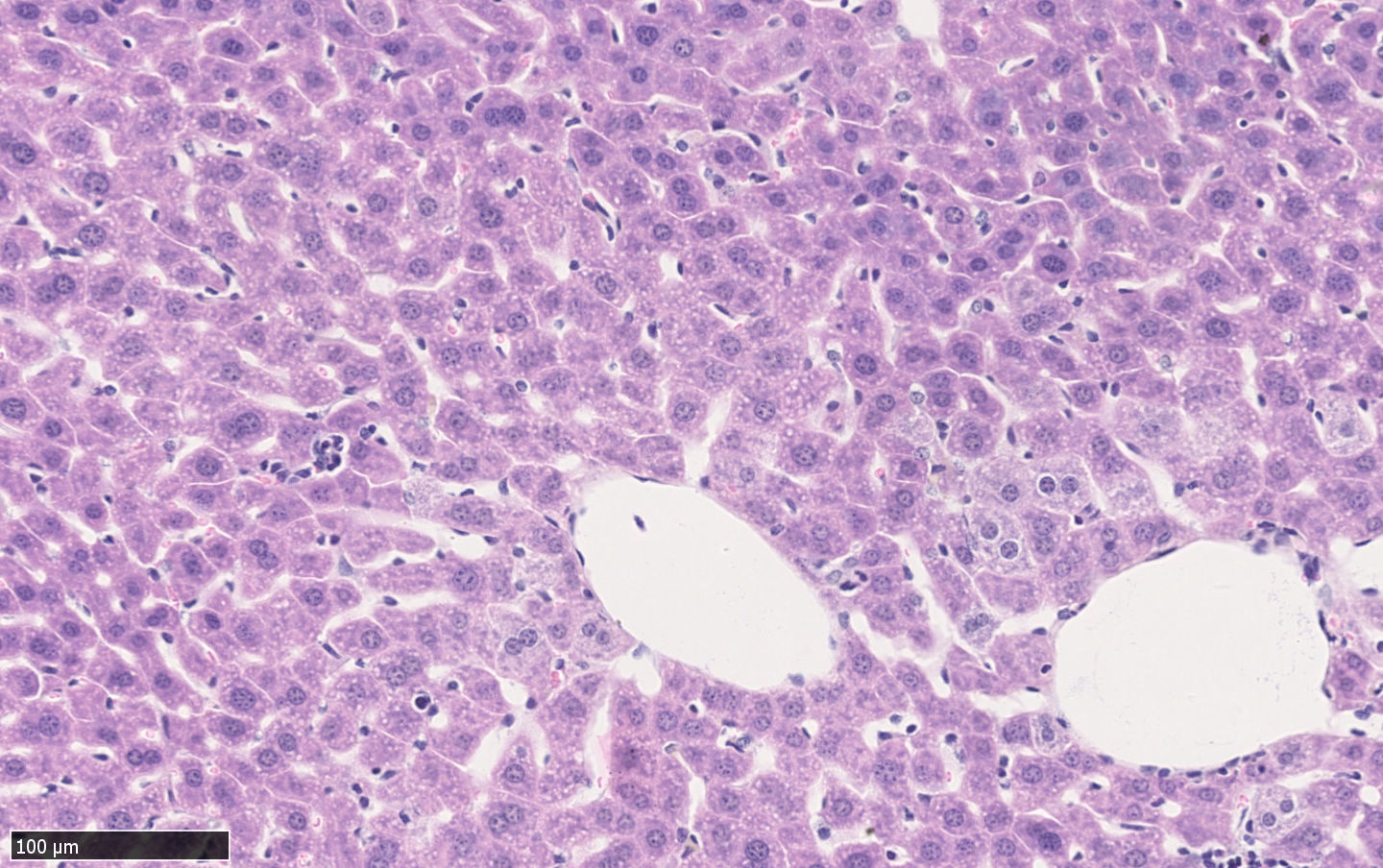

Supplement: Supplementary file 3 [file DataSheet8.ZIP › NASH SCORE-WT/WT17,18+/14.jpg]

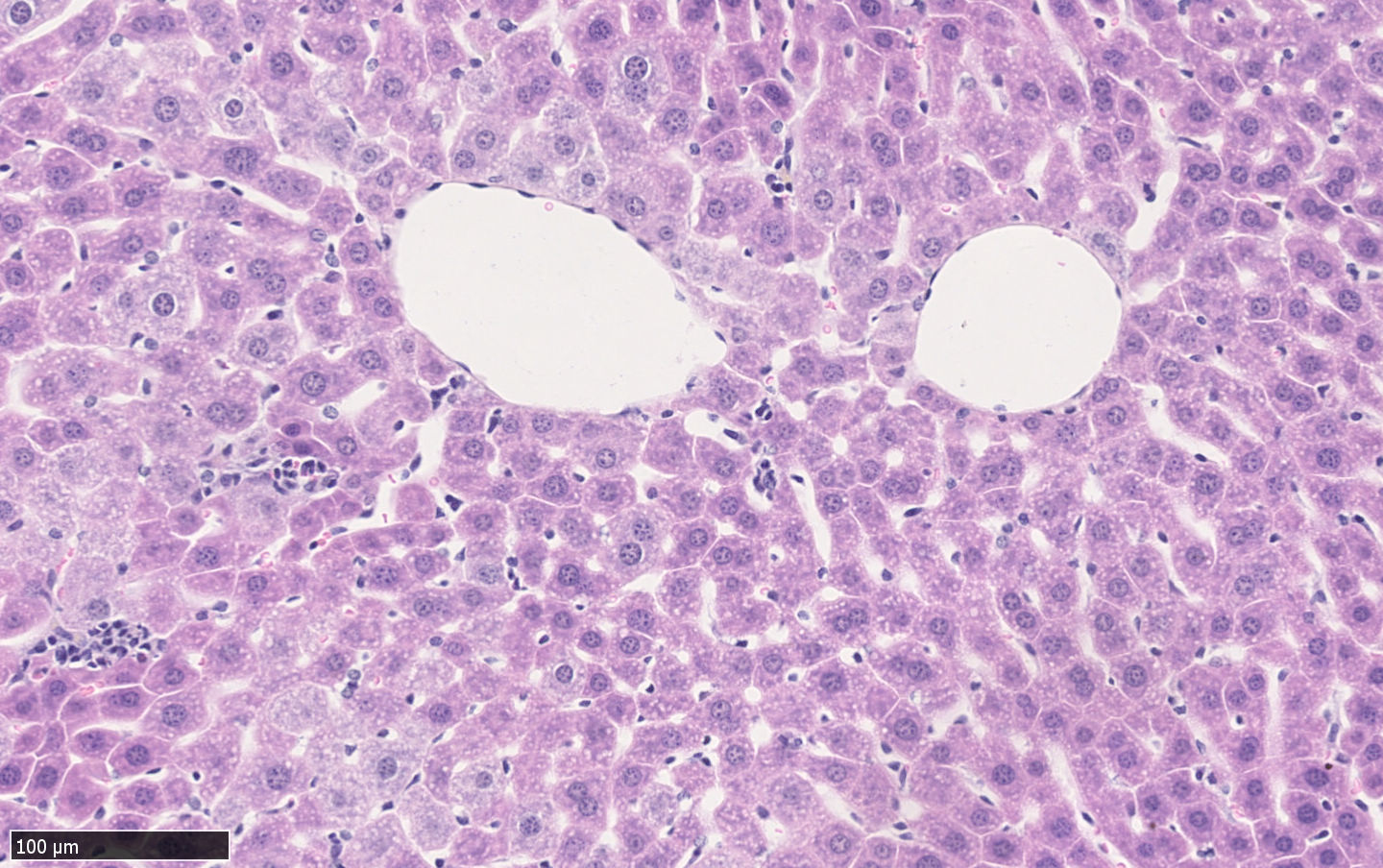

Supplement: Supplementary file 3 [file DataSheet8.ZIP › NASH SCORE-WT/WT17,18+/15.jpg]

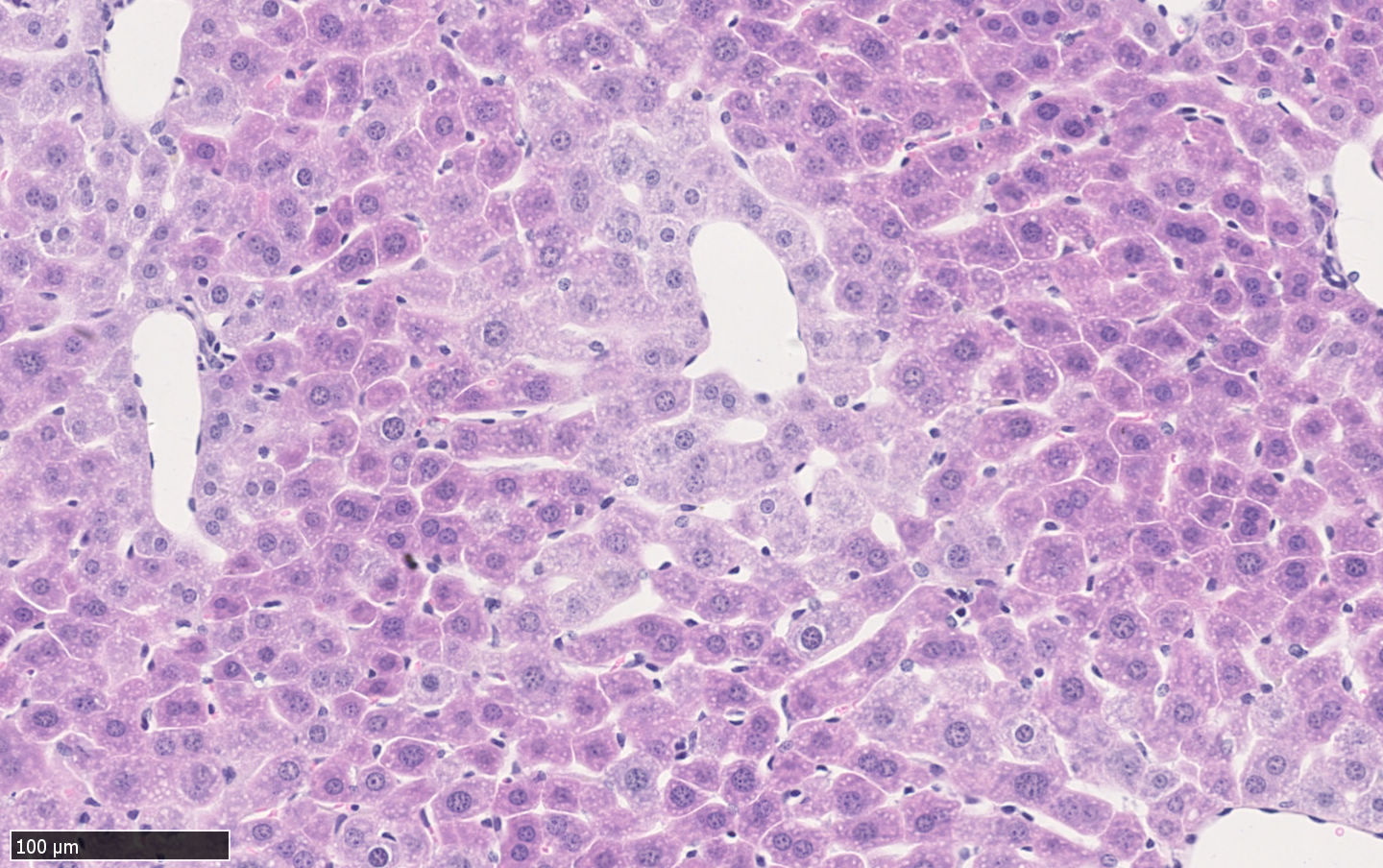

Supplement: Supplementary file 3 [file DataSheet8.ZIP › NASH SCORE-WT/WT17,18+/16.jpg]

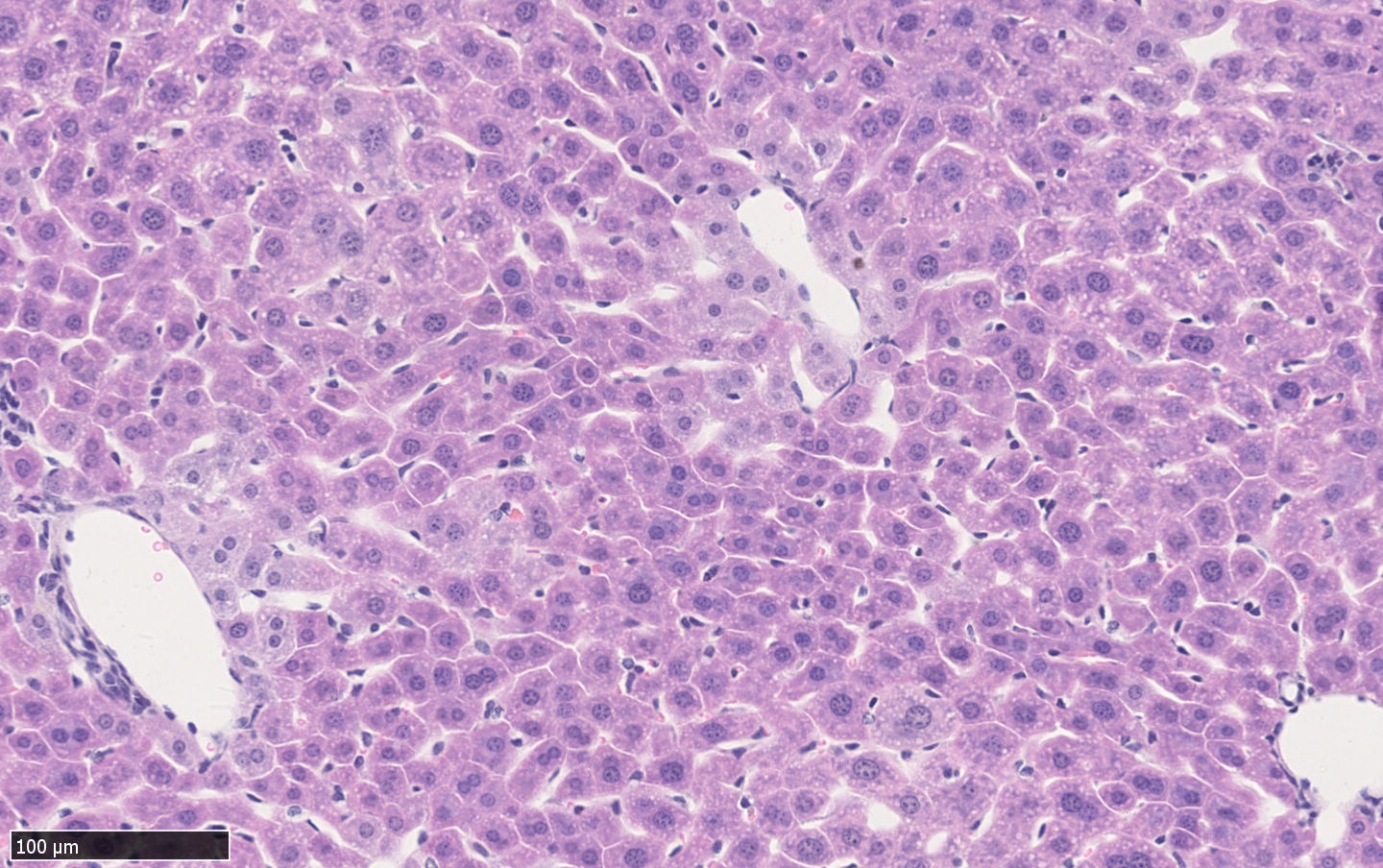

Supplement: Supplementary file 3 [file DataSheet8.ZIP › NASH SCORE-WT/WT17,18+/17.jpg]

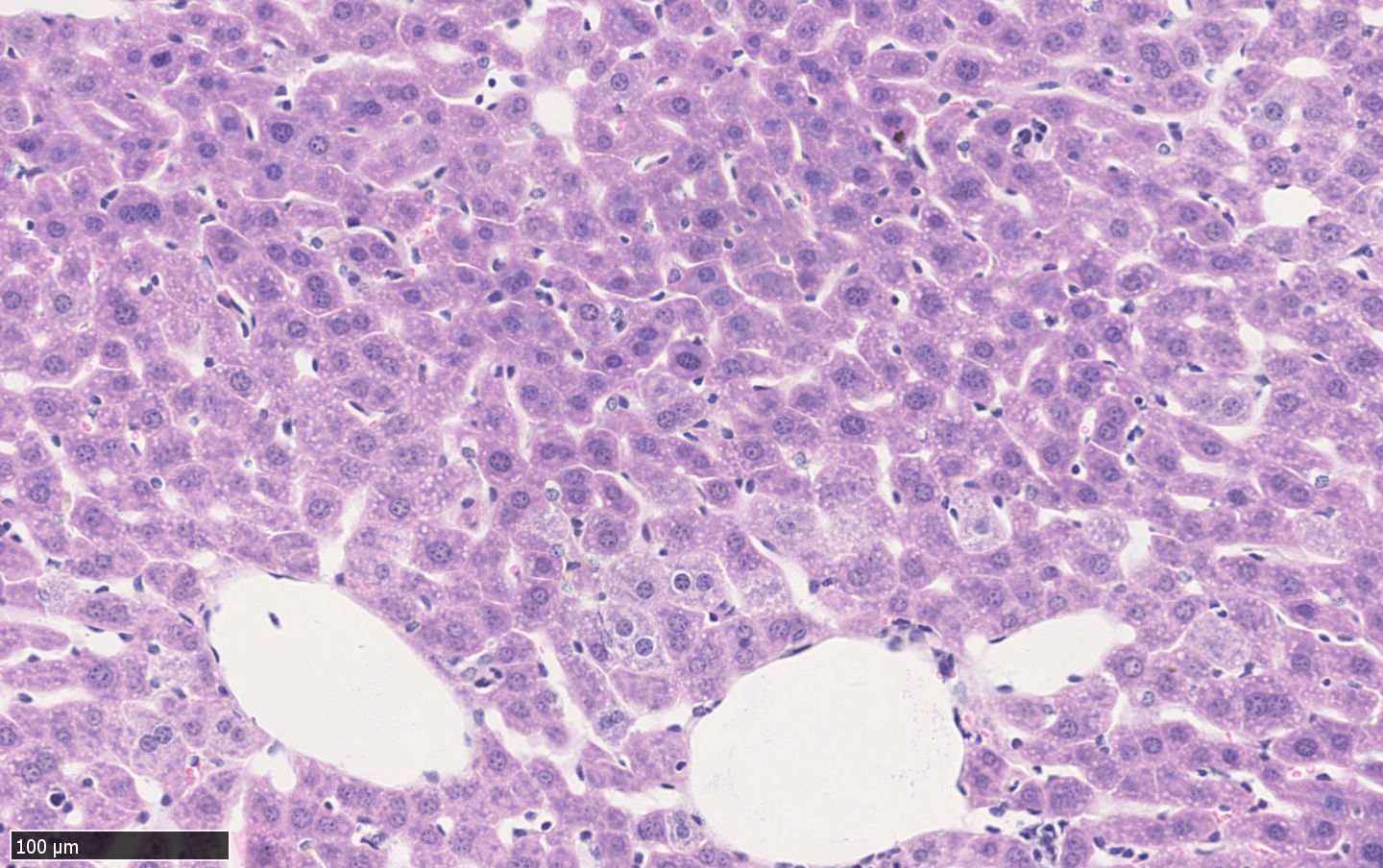

Supplement: Supplementary file 3 [file DataSheet8.ZIP › NASH SCORE-WT/WT17,18+/18.jpg]

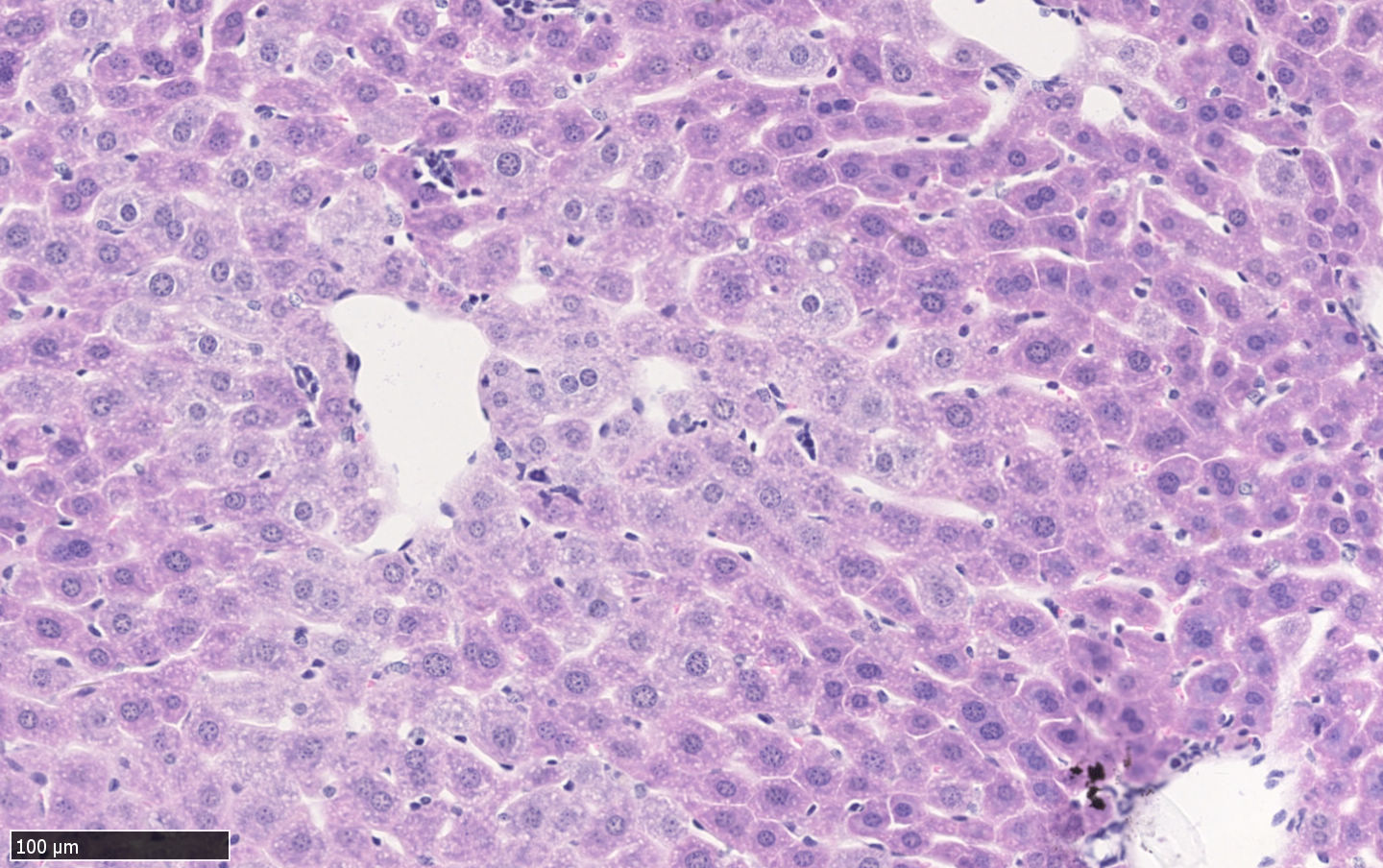

Supplement: Supplementary file 3 [file DataSheet8.ZIP › NASH SCORE-WT/WT17,18+/19.jpg]

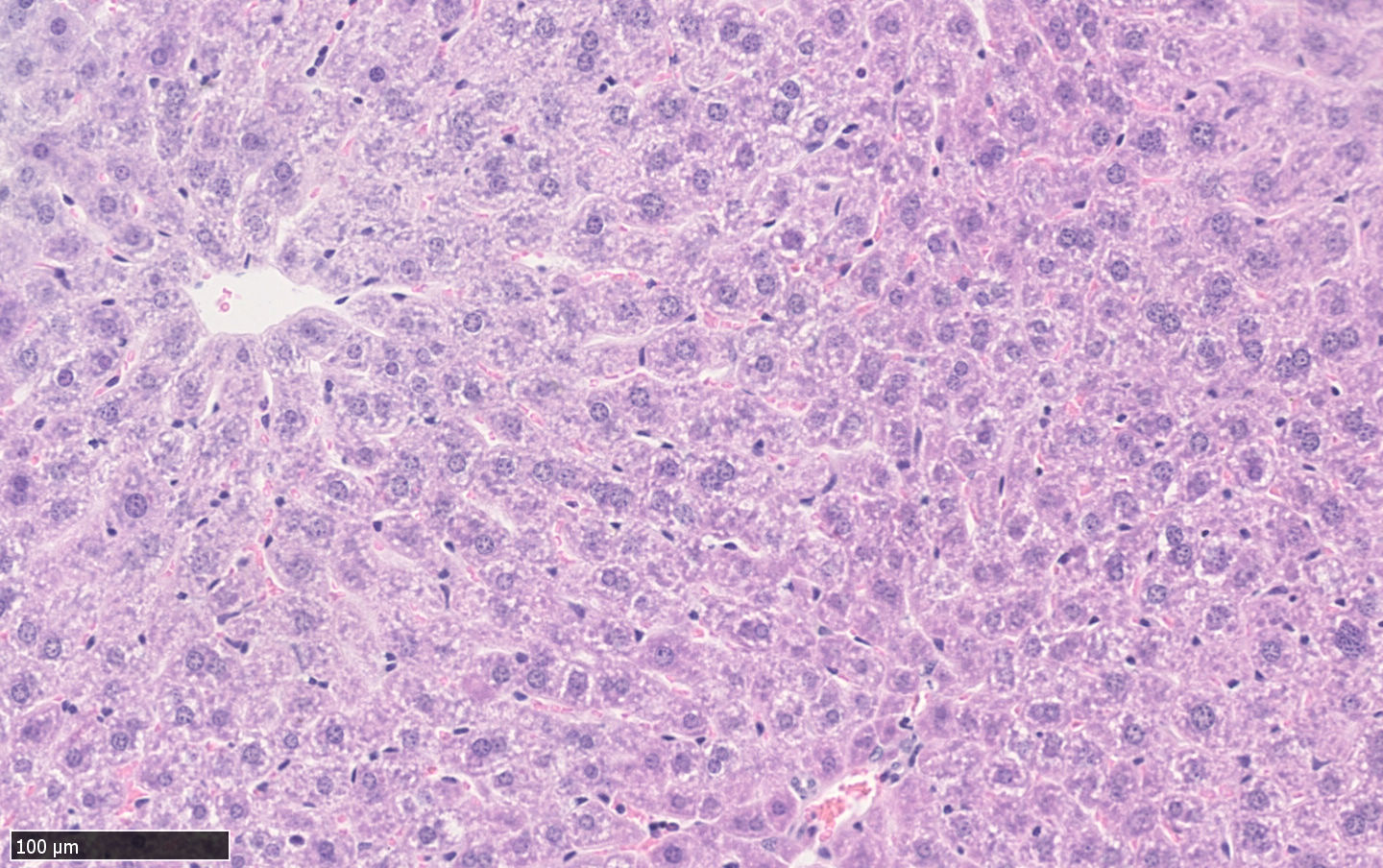

Supplement: Supplementary file 3 [file DataSheet8.ZIP › NASH SCORE-WT/WT17,18+/2.jpg]

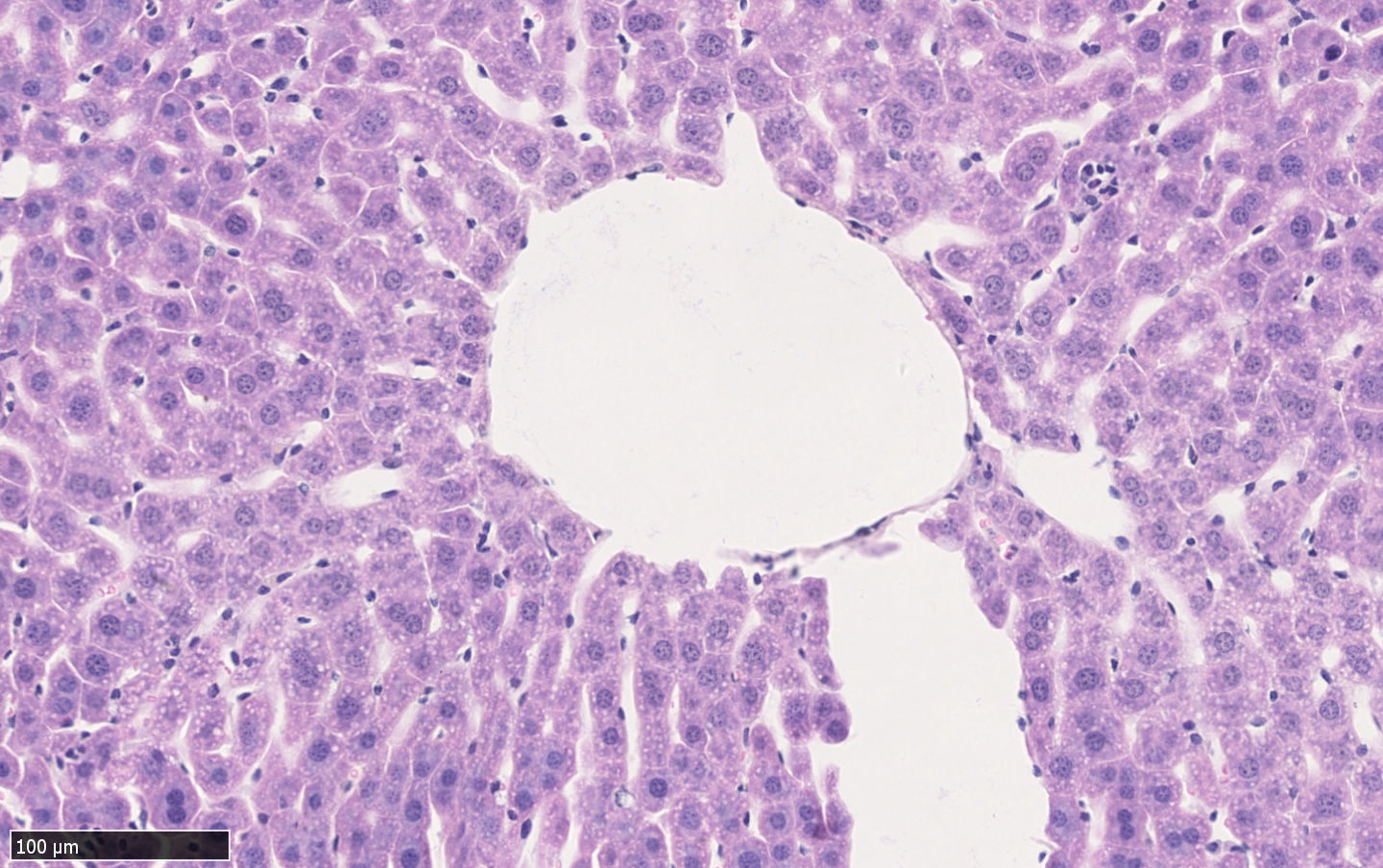

Supplement: Supplementary file 3 [file DataSheet8.ZIP › NASH SCORE-WT/WT17,18+/20.jpg]

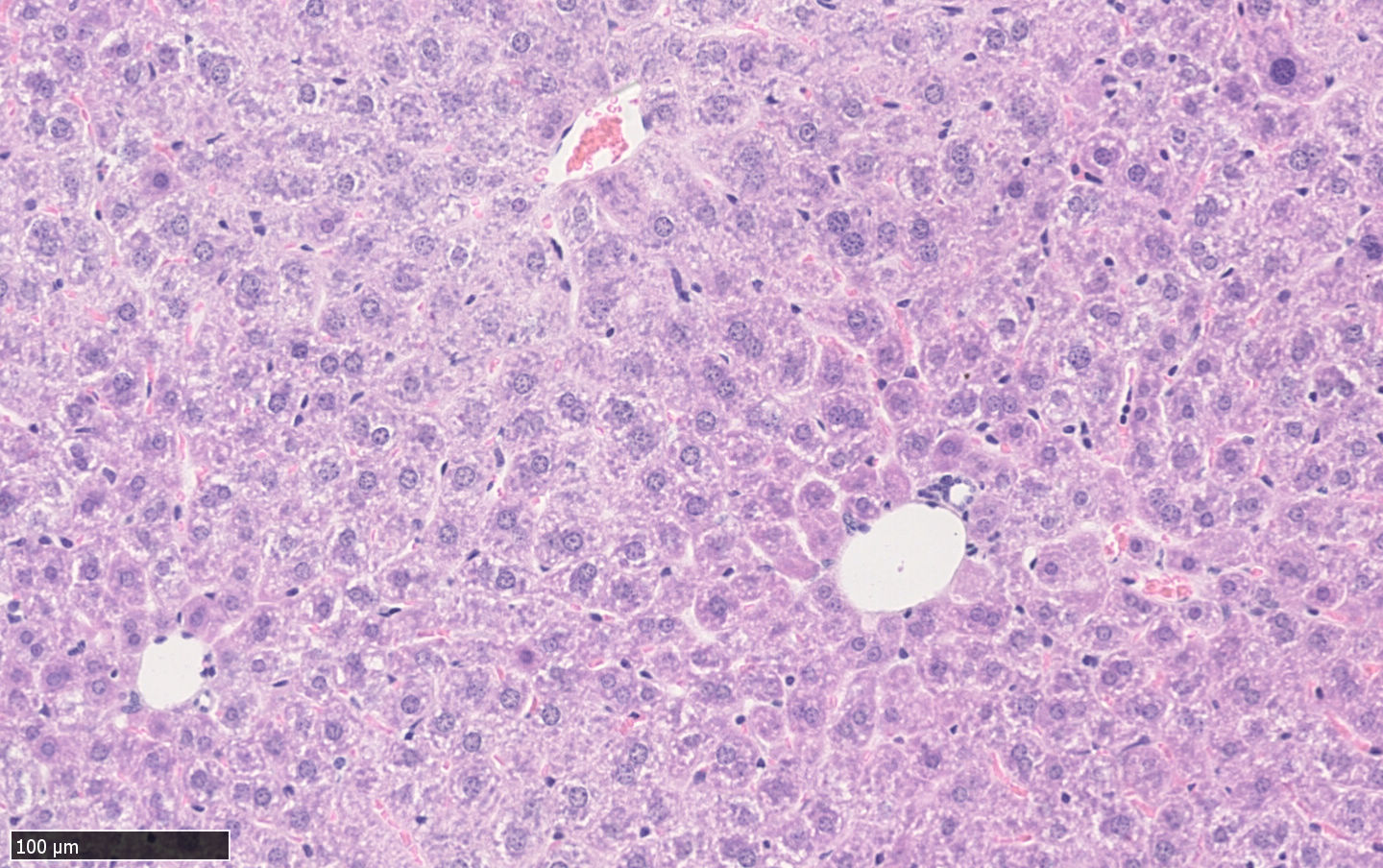

Supplement: Supplementary file 3 [file DataSheet8.ZIP › NASH SCORE-WT/WT17,18+/3.jpg]

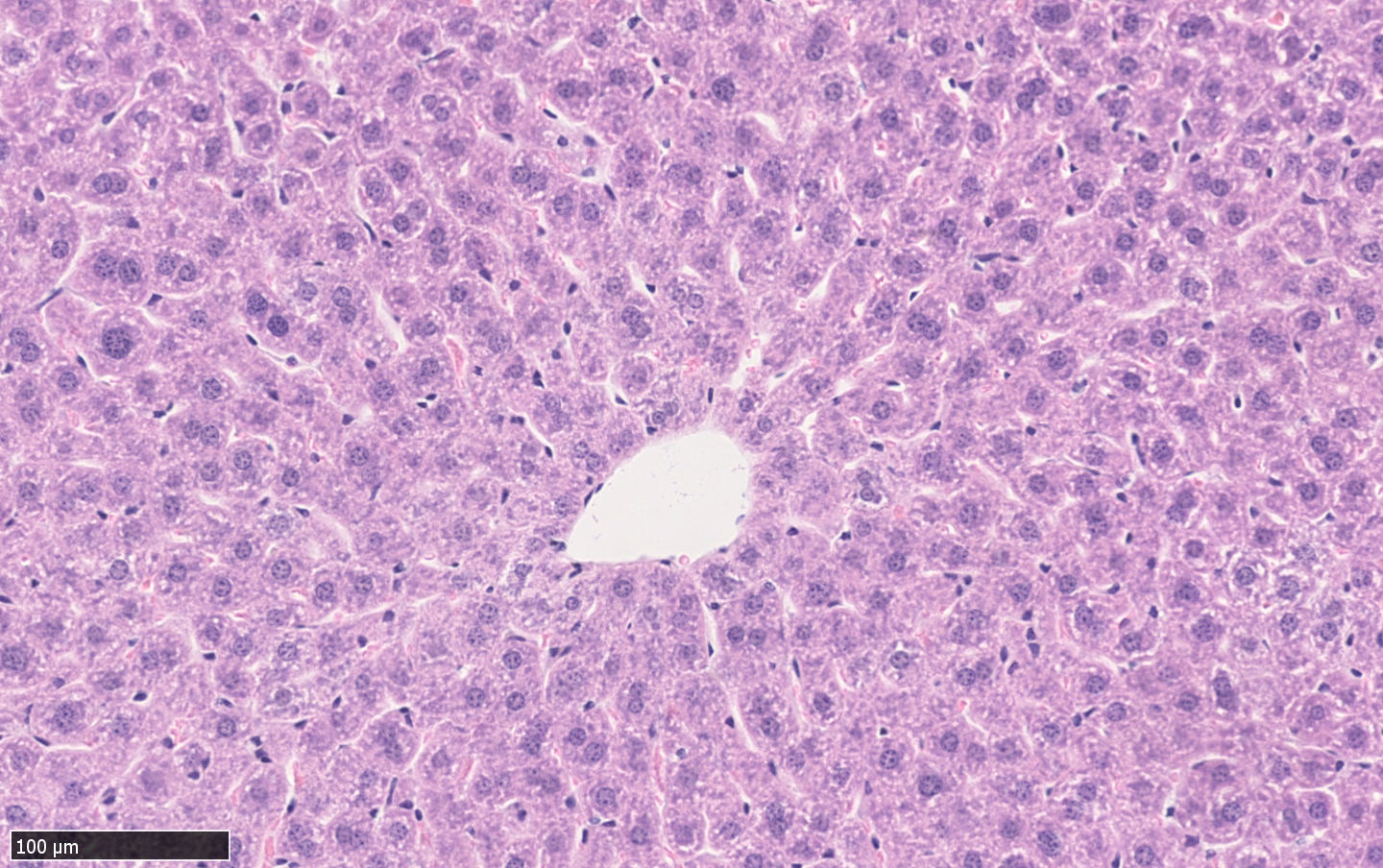

Supplement: Supplementary file 3 [file DataSheet8.ZIP › NASH SCORE-WT/WT17,18+/4.jpg]

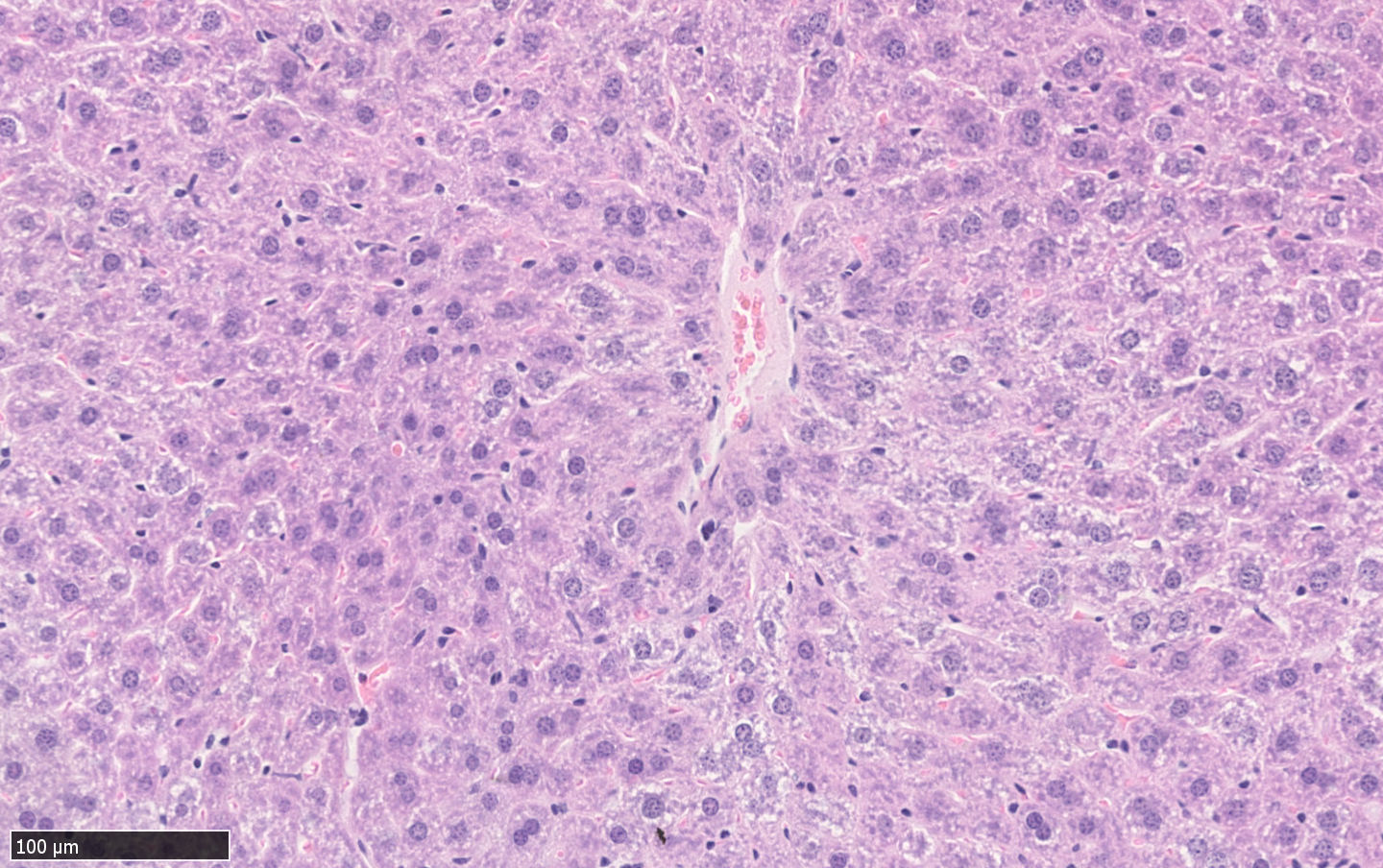

Supplement: Supplementary file 3 [file DataSheet8.ZIP › NASH SCORE-WT/WT17,18+/5.jpg]

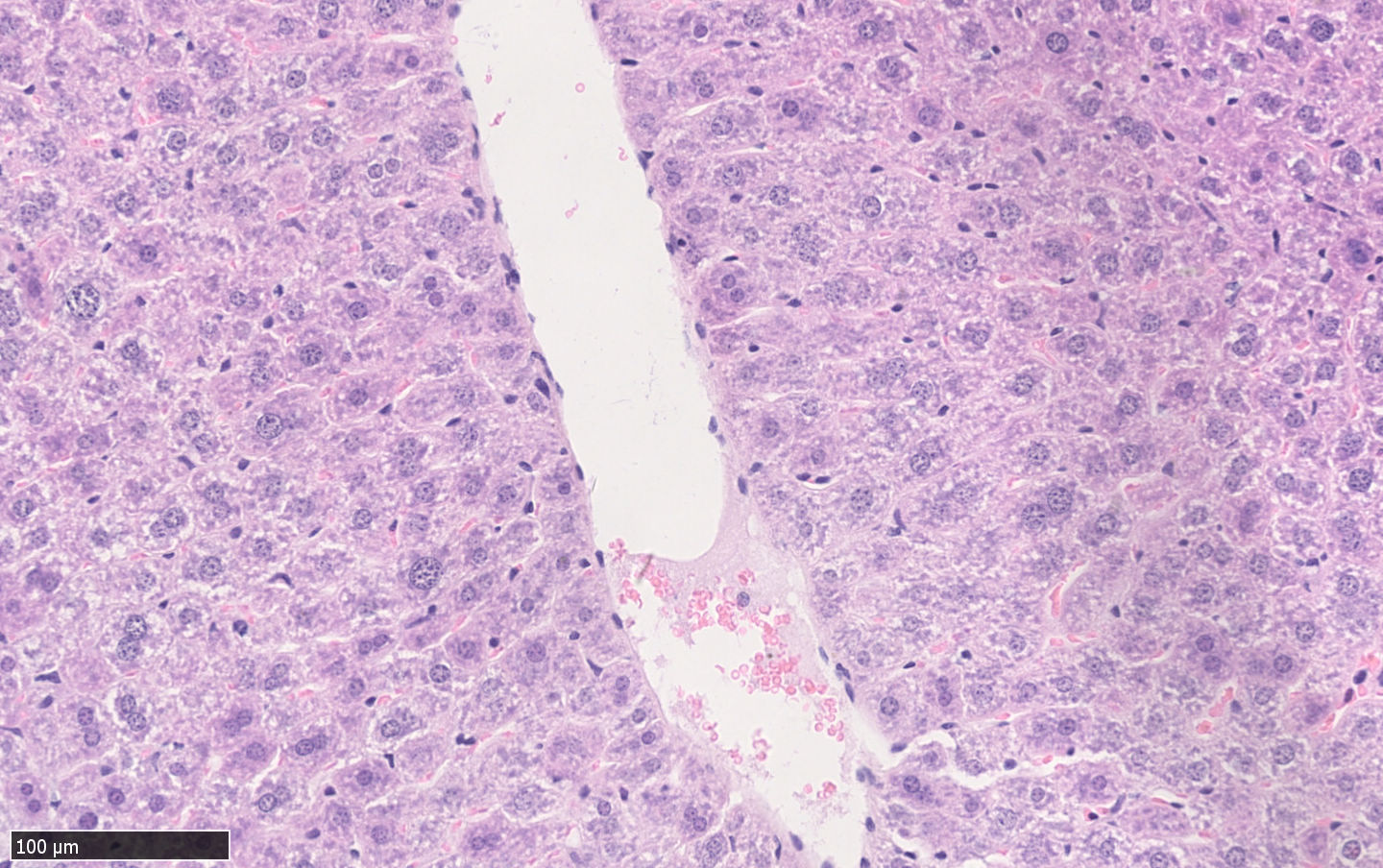

Supplement: Supplementary file 3 [file DataSheet8.ZIP › NASH SCORE-WT/WT17,18+/6.jpg]

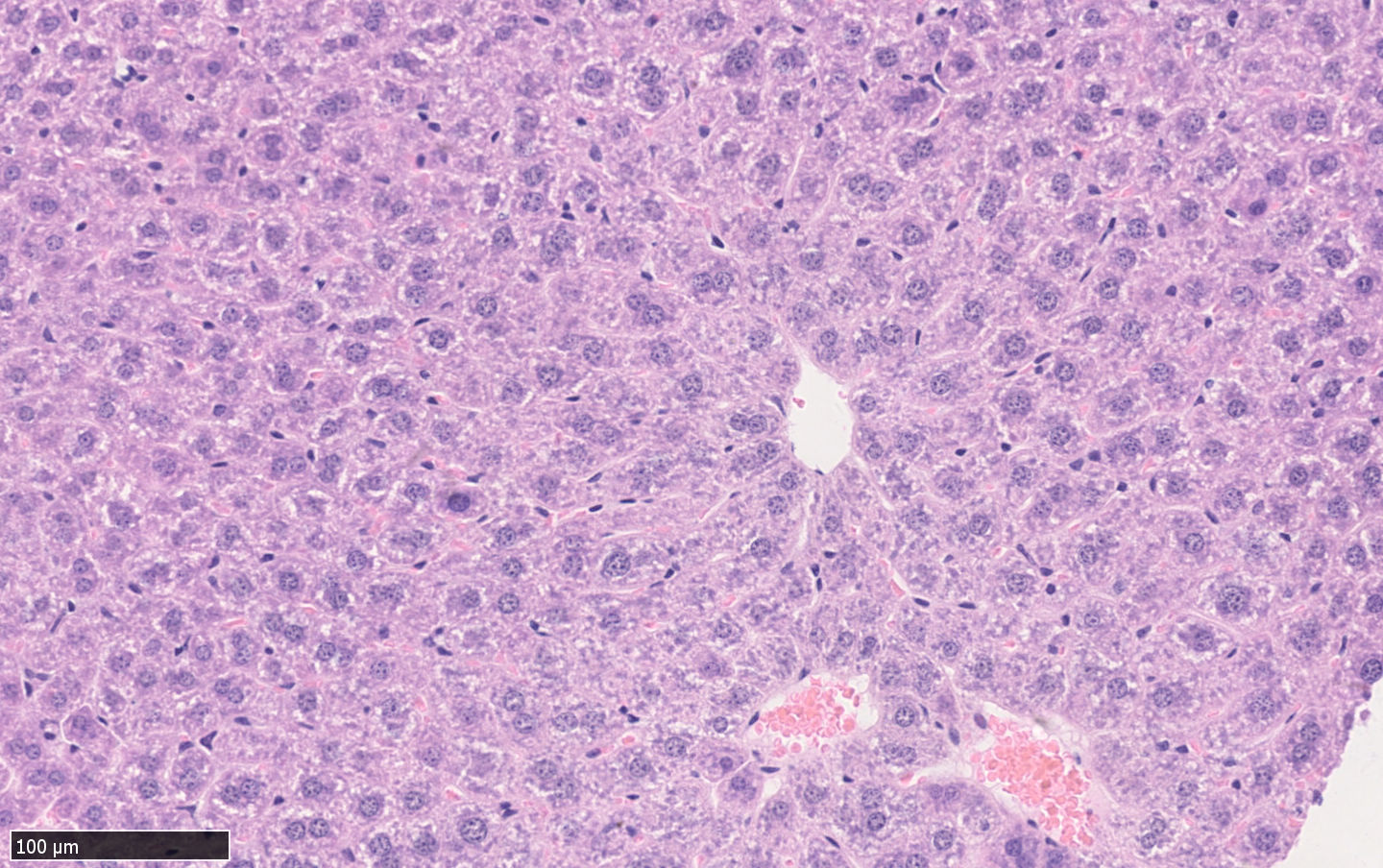

Supplement: Supplementary file 3 [file DataSheet8.ZIP › NASH SCORE-WT/WT17,18+/7.jpg]

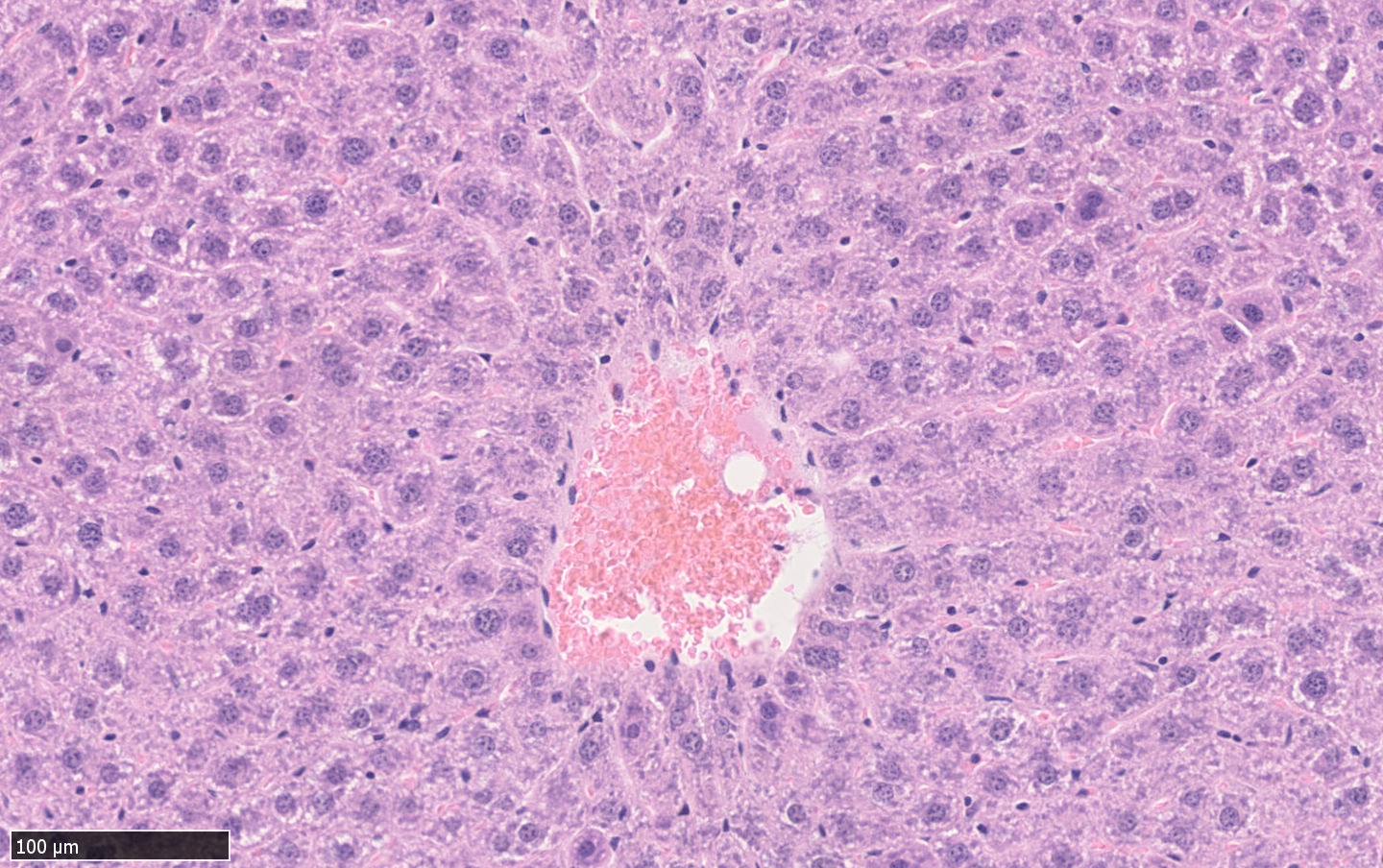

Supplement: Supplementary file 3 [file DataSheet8.ZIP › NASH SCORE-WT/WT17,18+/8.jpg]

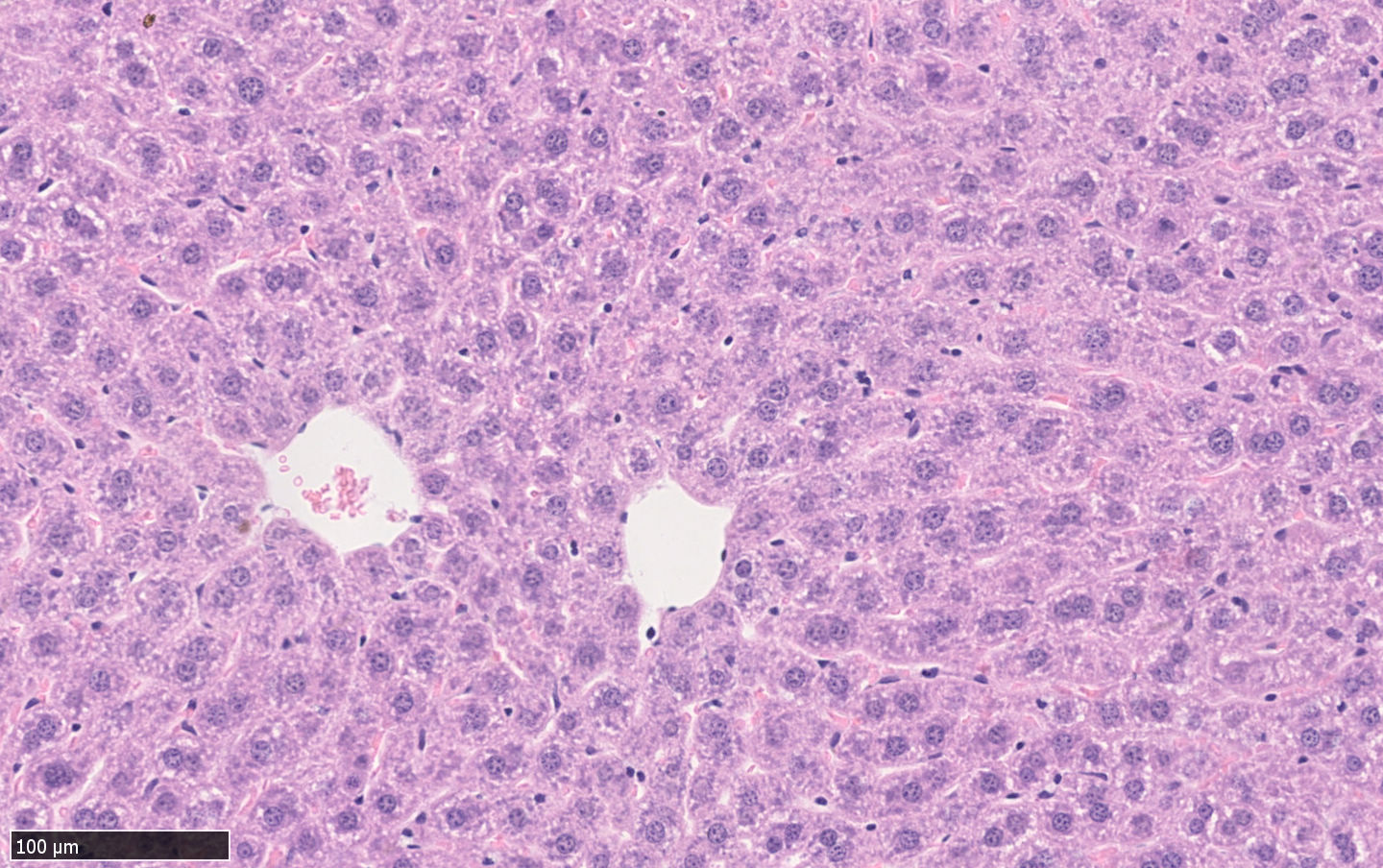

Supplement: Supplementary file 3 [file DataSheet8.ZIP › NASH SCORE-WT/WT17,18+/9.jpg]

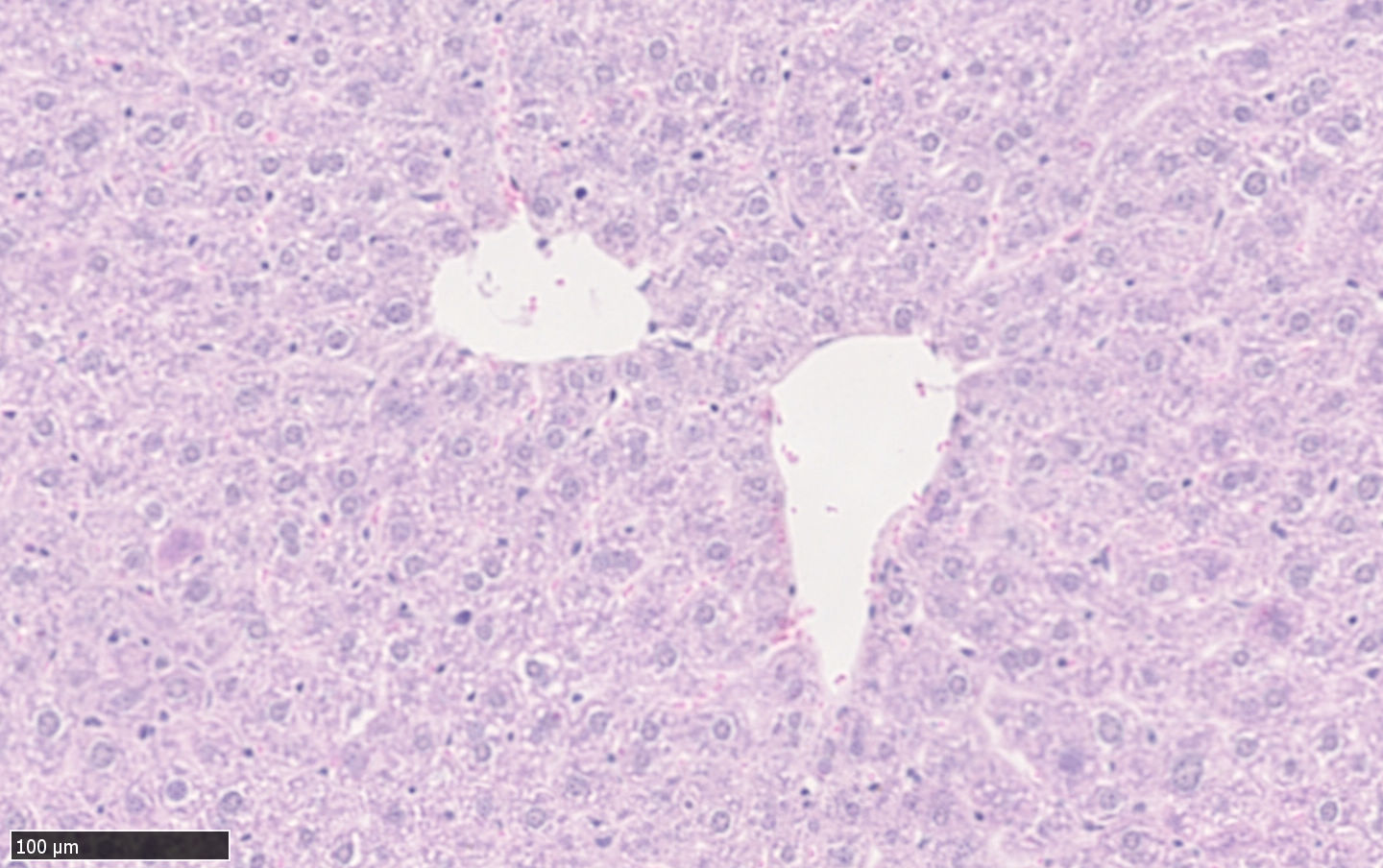

Supplement: Supplementary file 3 [file DataSheet8.ZIP › NASH SCORE-WT/WT9,10/1.jpg]

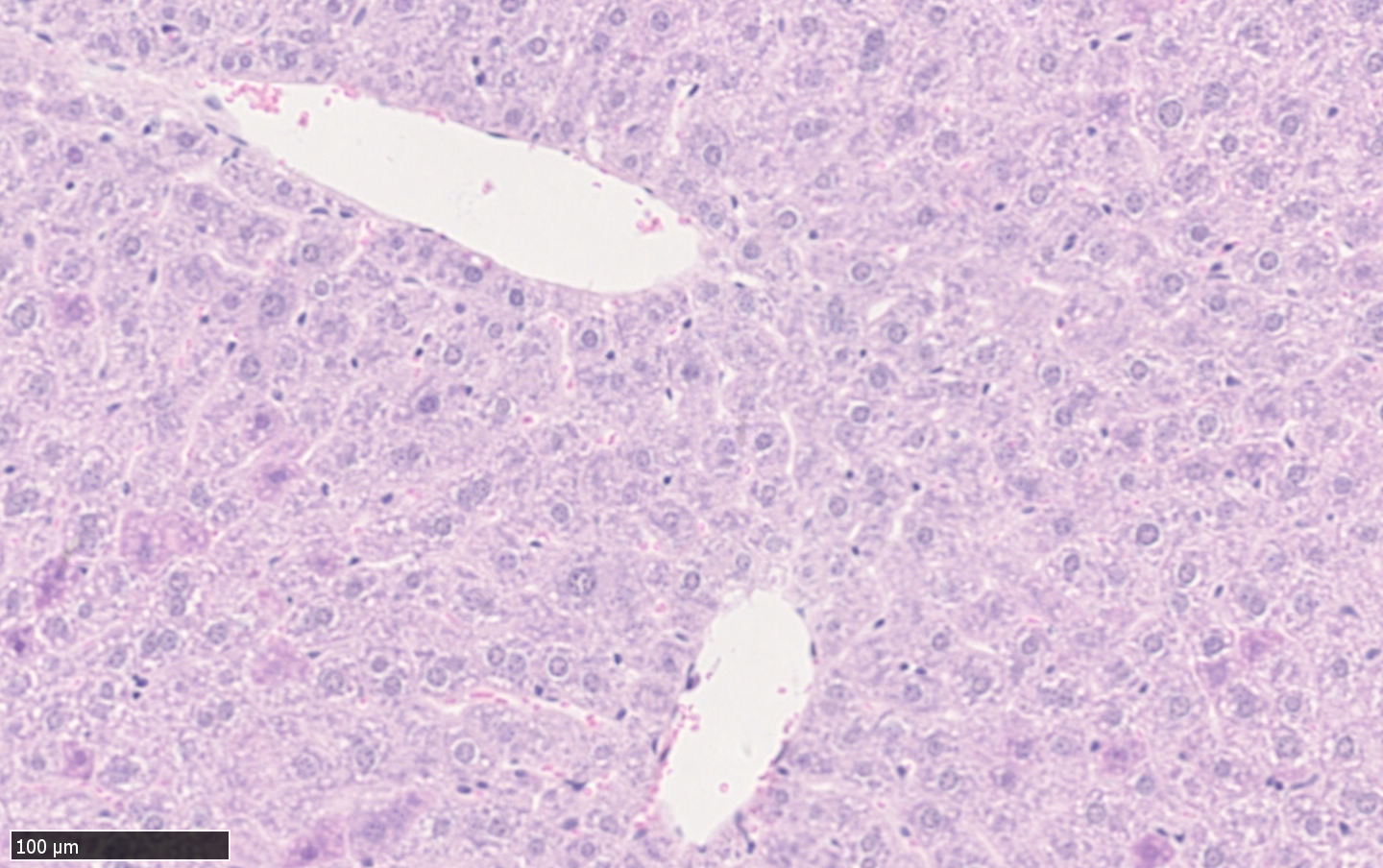

Supplement: Supplementary file 3 [file DataSheet8.ZIP › NASH SCORE-WT/WT9,10/10.jpg]

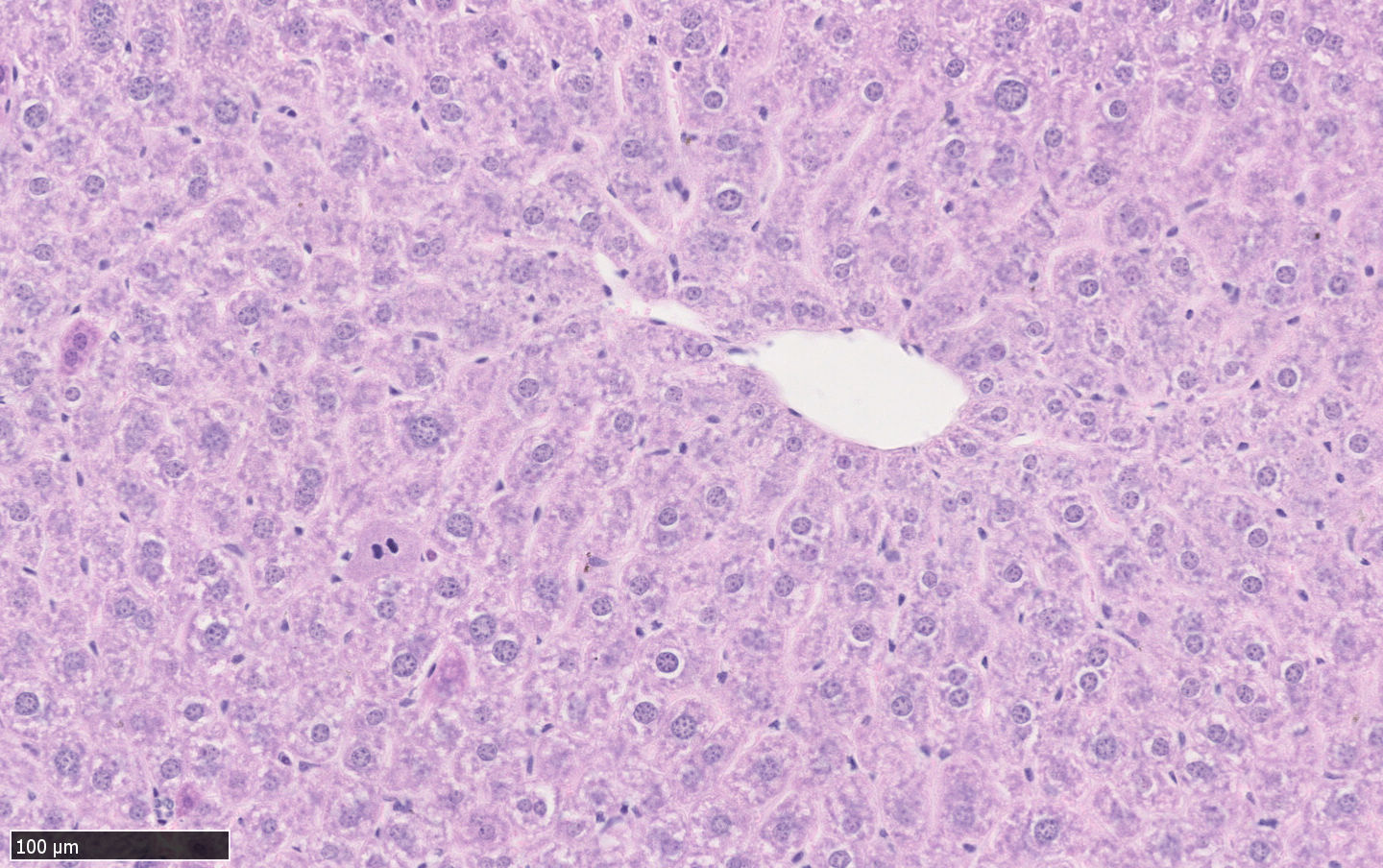

Supplement: Supplementary file 3 [file DataSheet8.ZIP › NASH SCORE-WT/WT9,10/11.jpg]

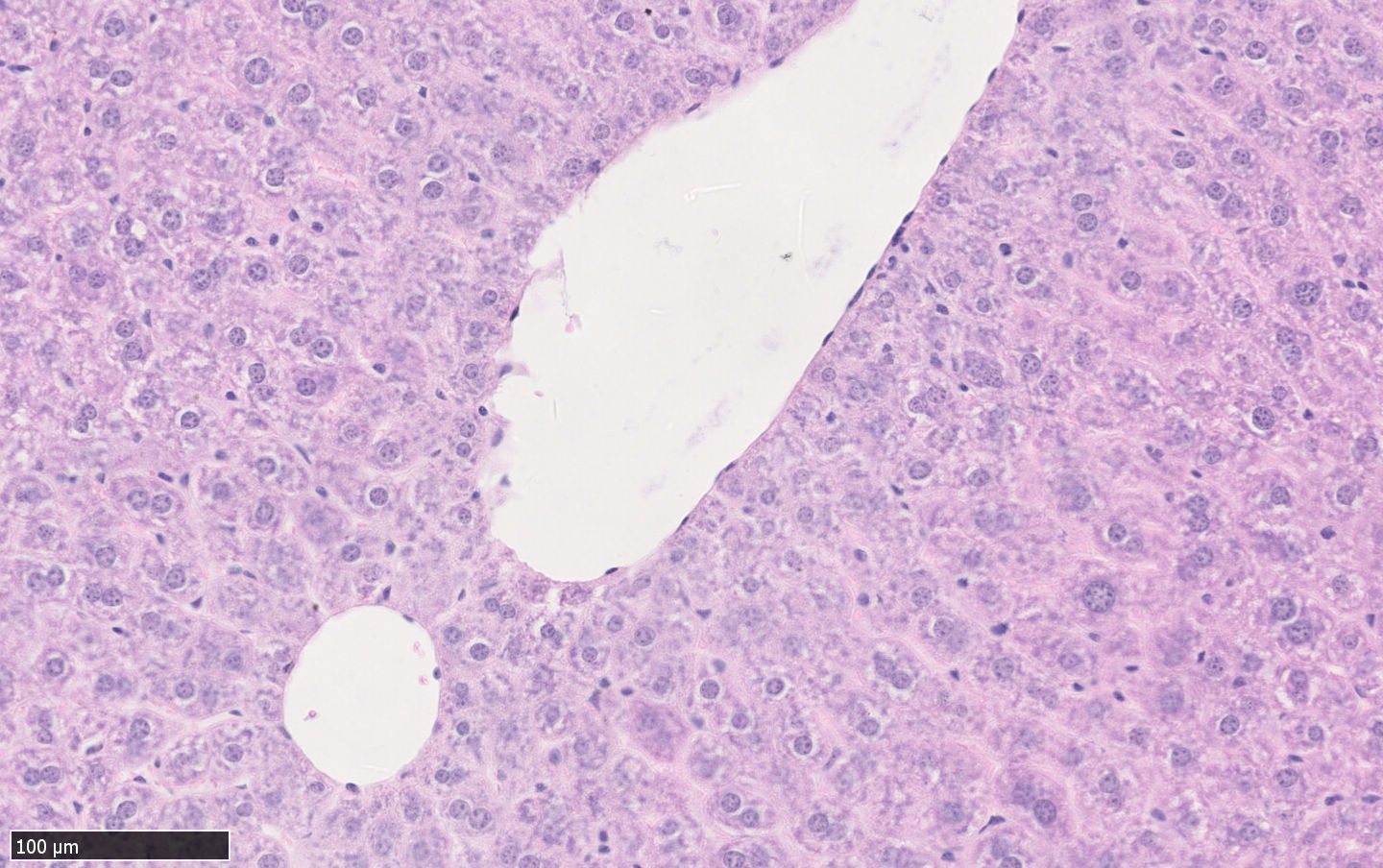

Supplement: Supplementary file 3 [file DataSheet8.ZIP › NASH SCORE-WT/WT9,10/12.jpg]

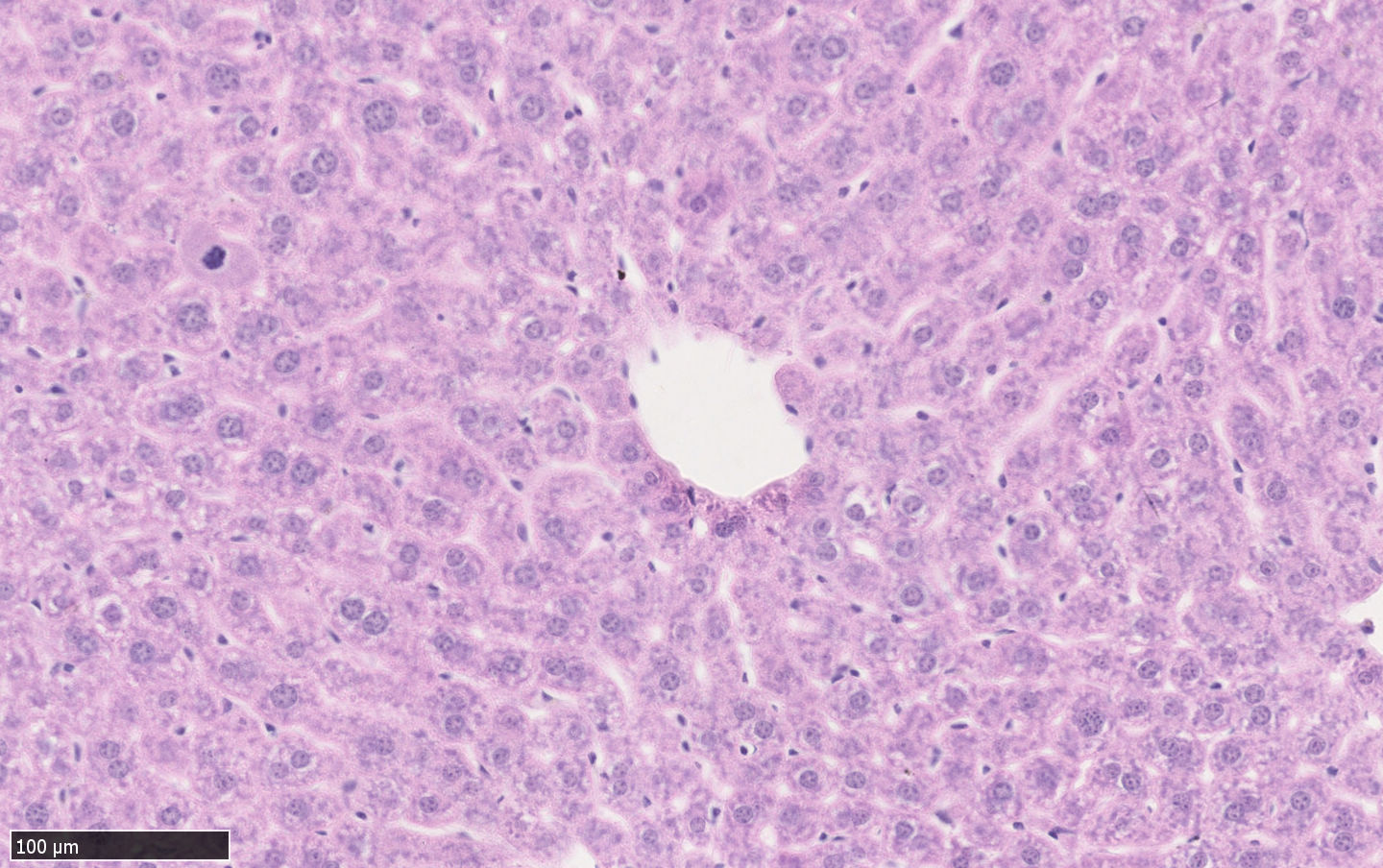

Supplement: Supplementary file 3 [file DataSheet8.ZIP › NASH SCORE-WT/WT9,10/13.jpg]

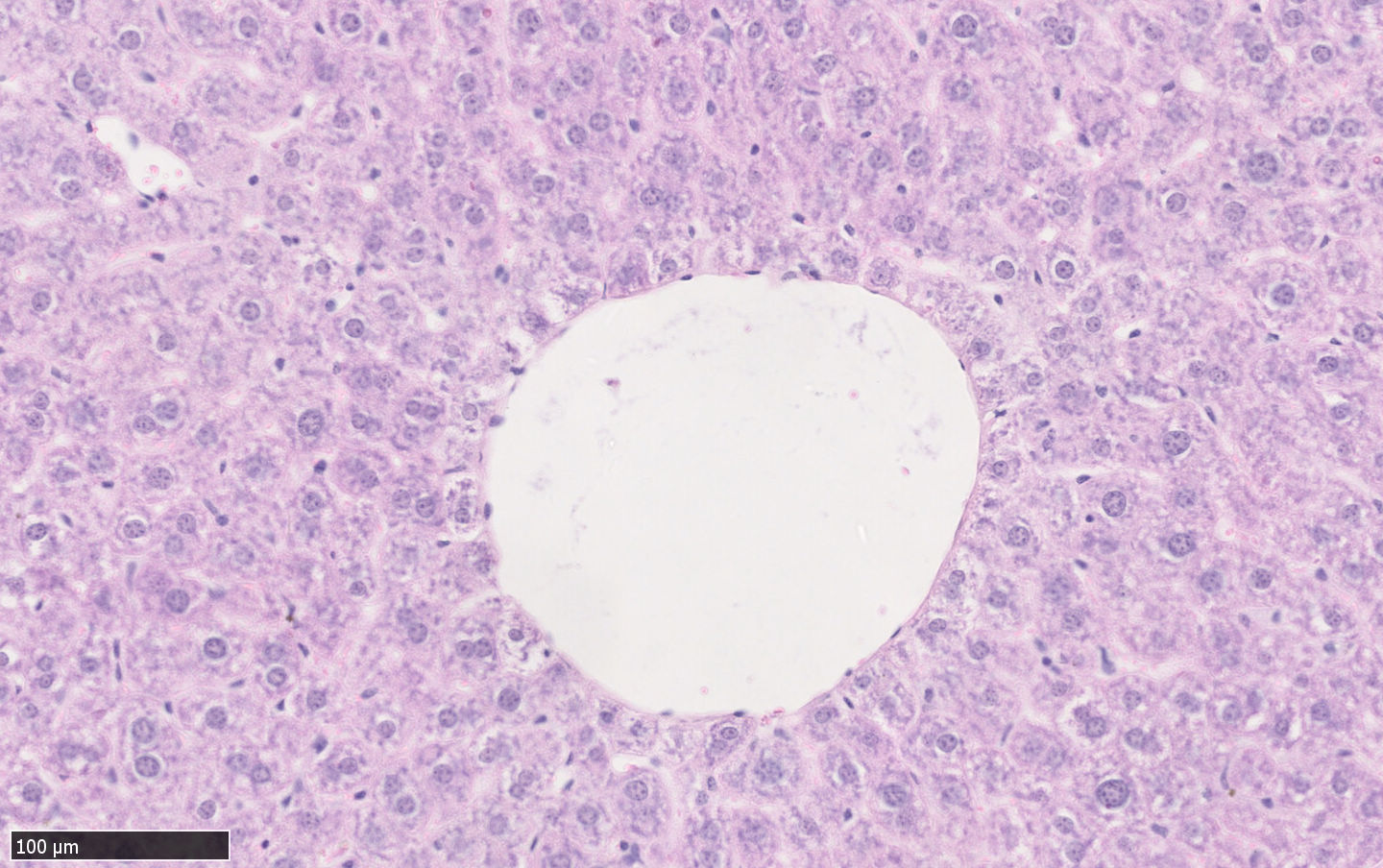

Supplement: Supplementary file 3 [file DataSheet8.ZIP › NASH SCORE-WT/WT9,10/14.jpg]

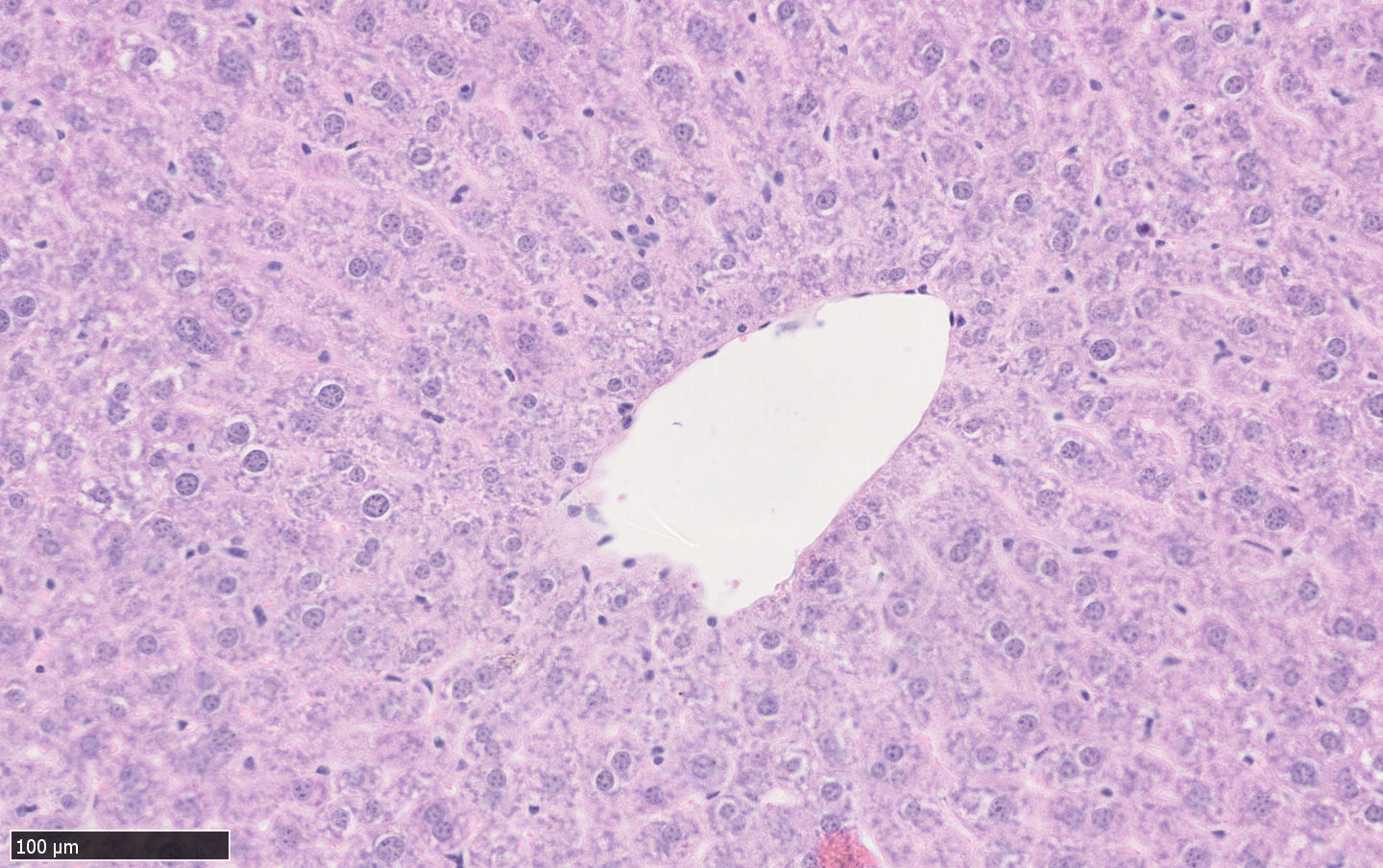

Supplement: Supplementary file 3 [file DataSheet8.ZIP › NASH SCORE-WT/WT9,10/15.jpg]

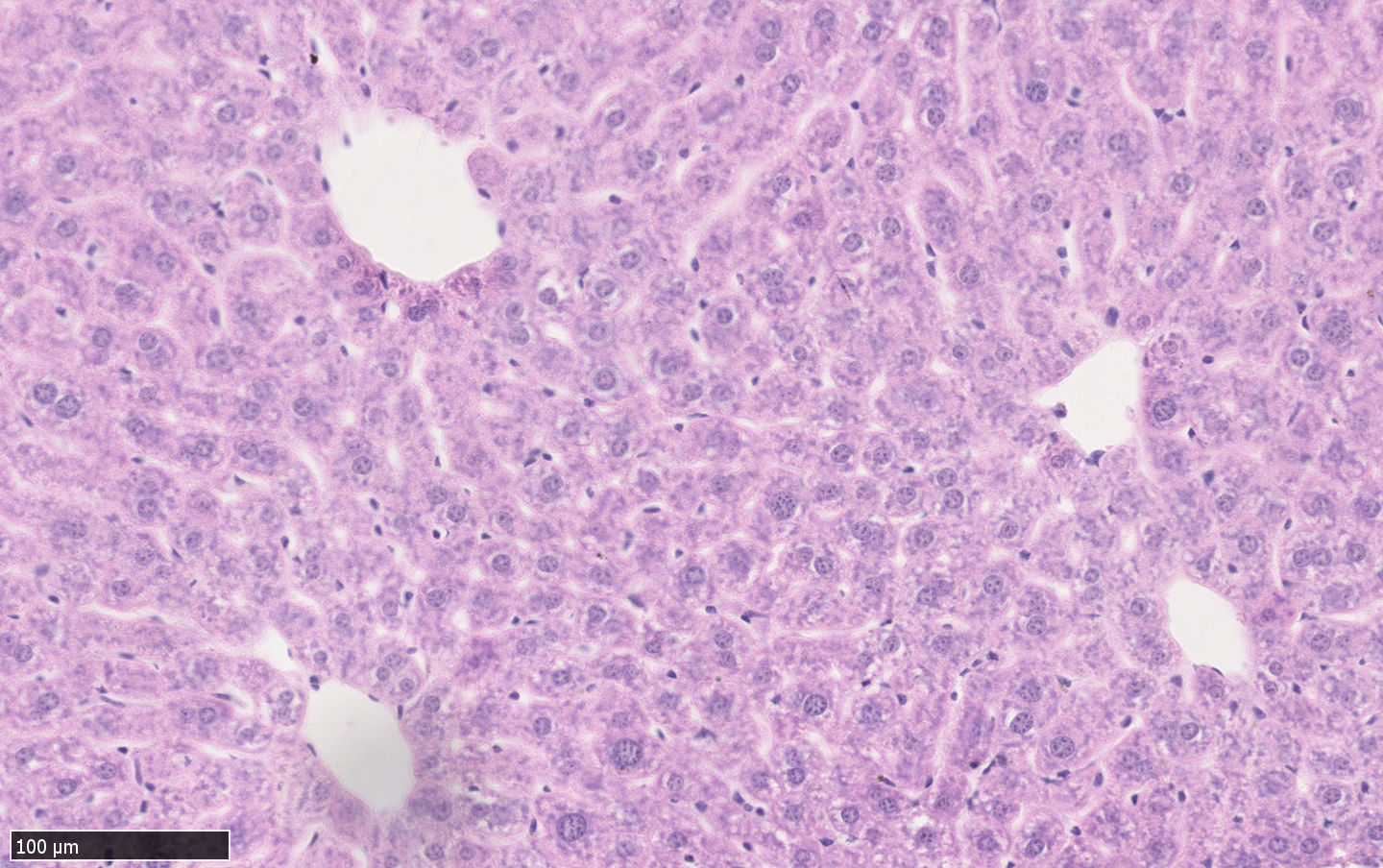

Supplement: Supplementary file 3 [file DataSheet8.ZIP › NASH SCORE-WT/WT9,10/16.jpg]

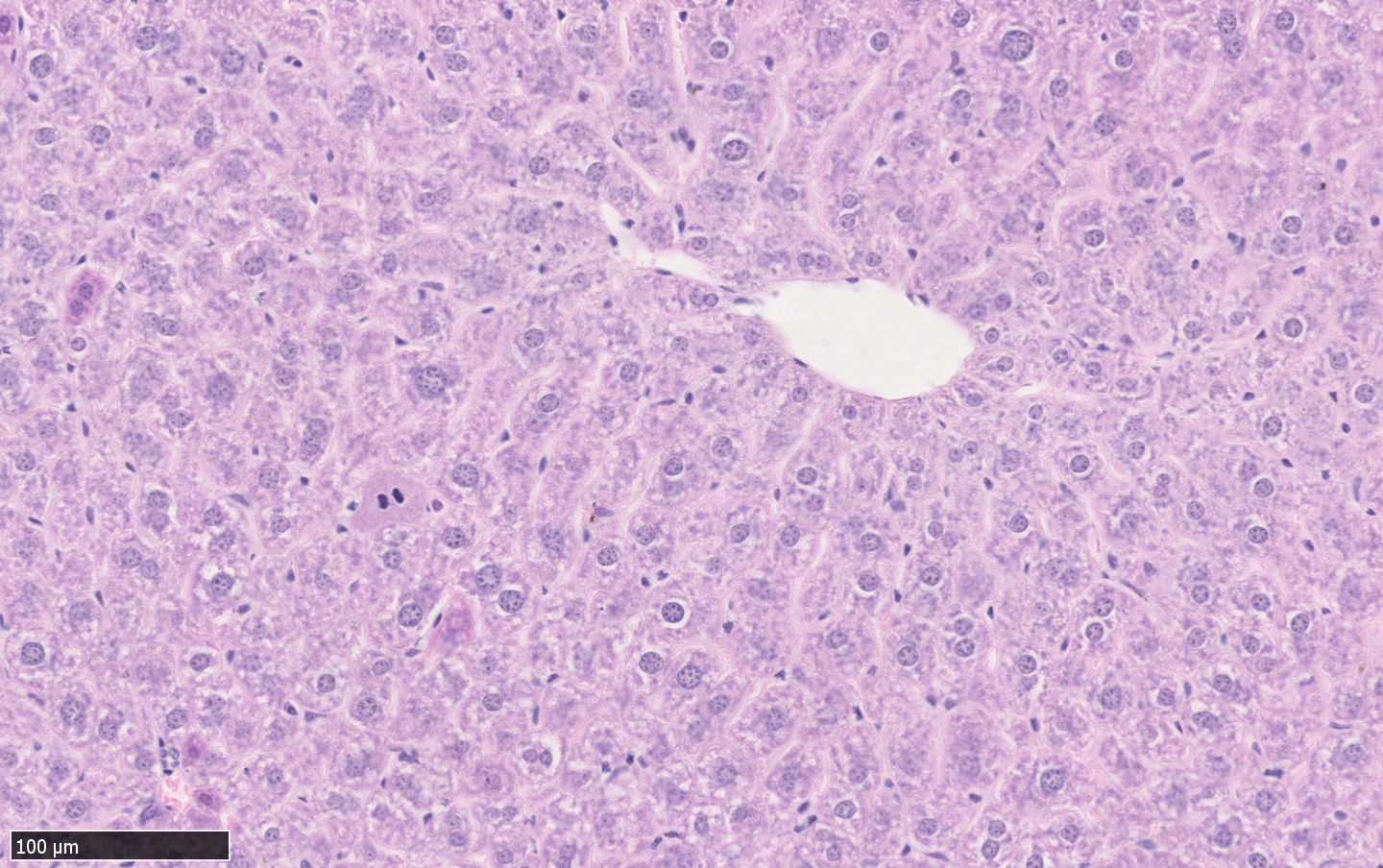

Supplement: Supplementary file 3 [file DataSheet8.ZIP › NASH SCORE-WT/WT9,10/17.jpg]

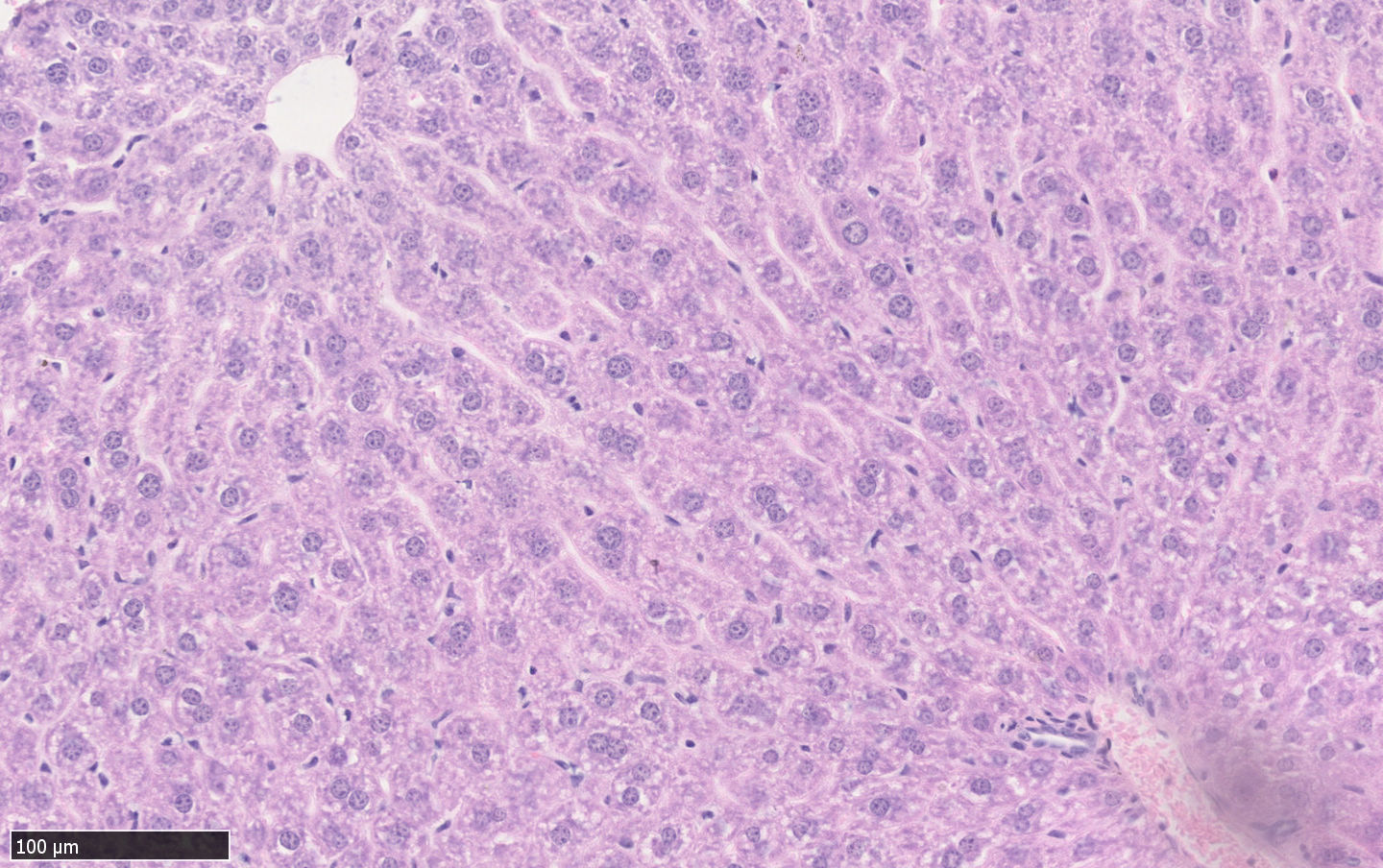

Supplement: Supplementary file 3 [file DataSheet8.ZIP › NASH SCORE-WT/WT9,10/18.jpg]

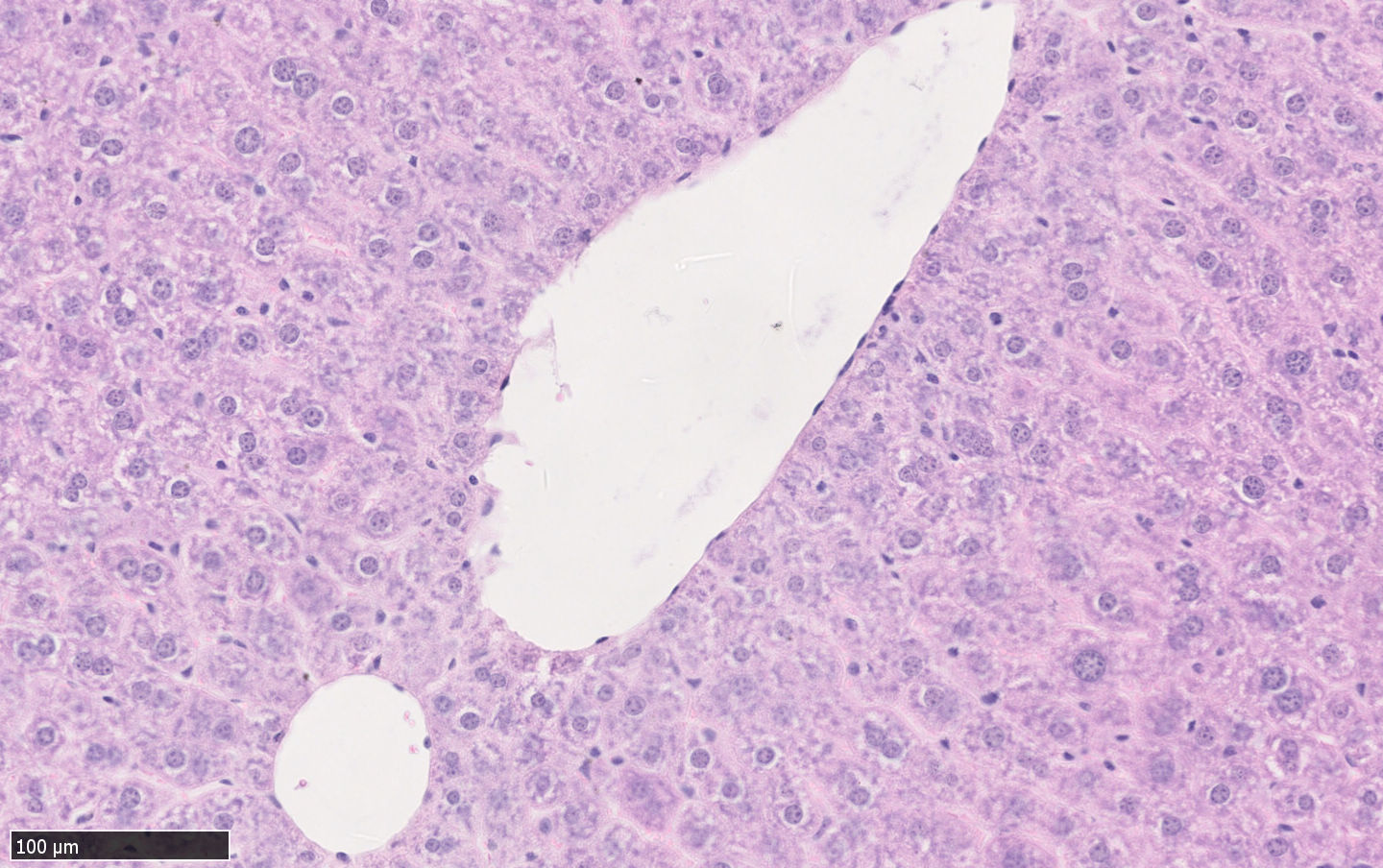

Supplement: Supplementary file 3 [file DataSheet8.ZIP › NASH SCORE-WT/WT9,10/19.jpg]

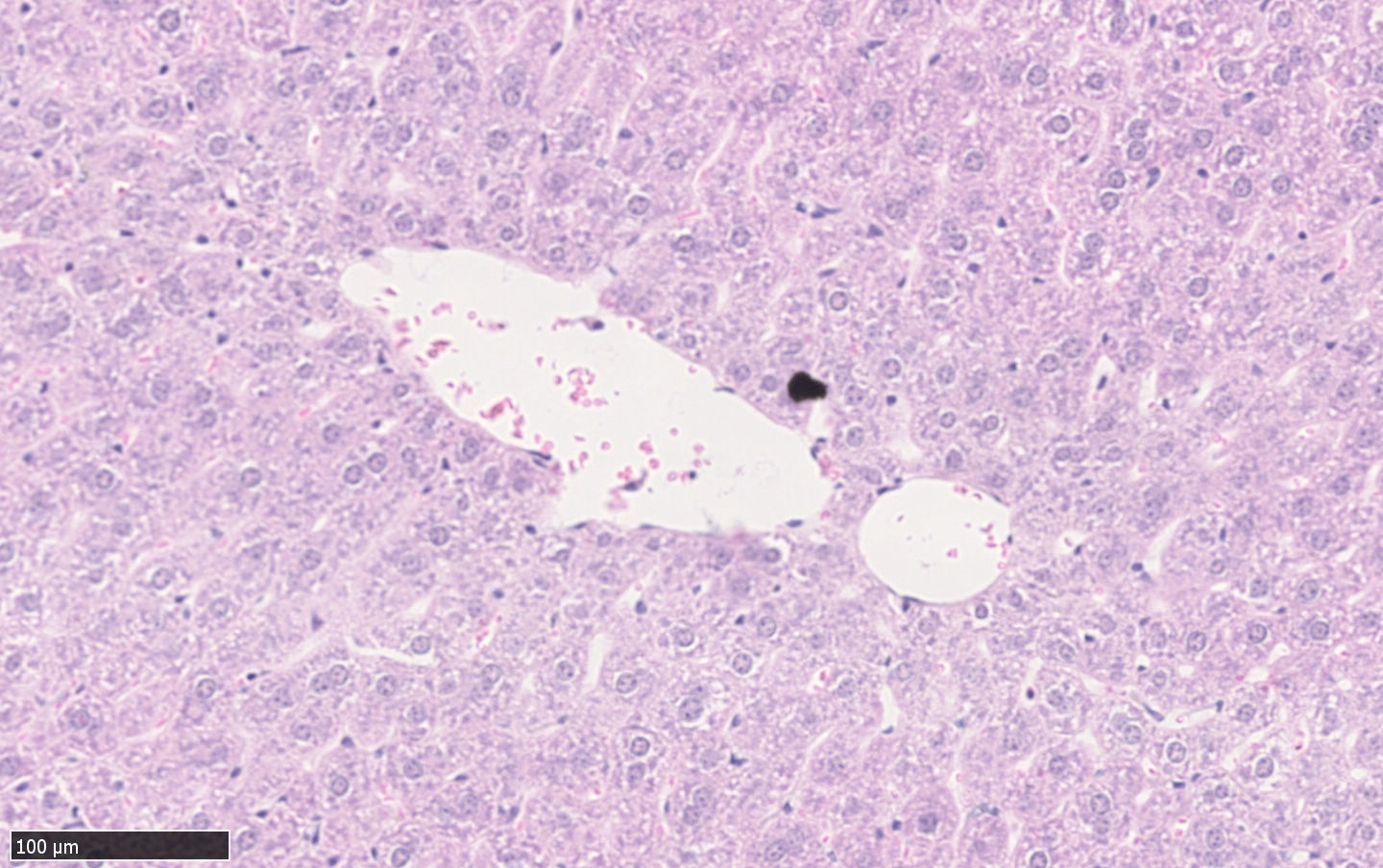

Supplement: Supplementary file 3 [file DataSheet8.ZIP › NASH SCORE-WT/WT9,10/2.jpg]

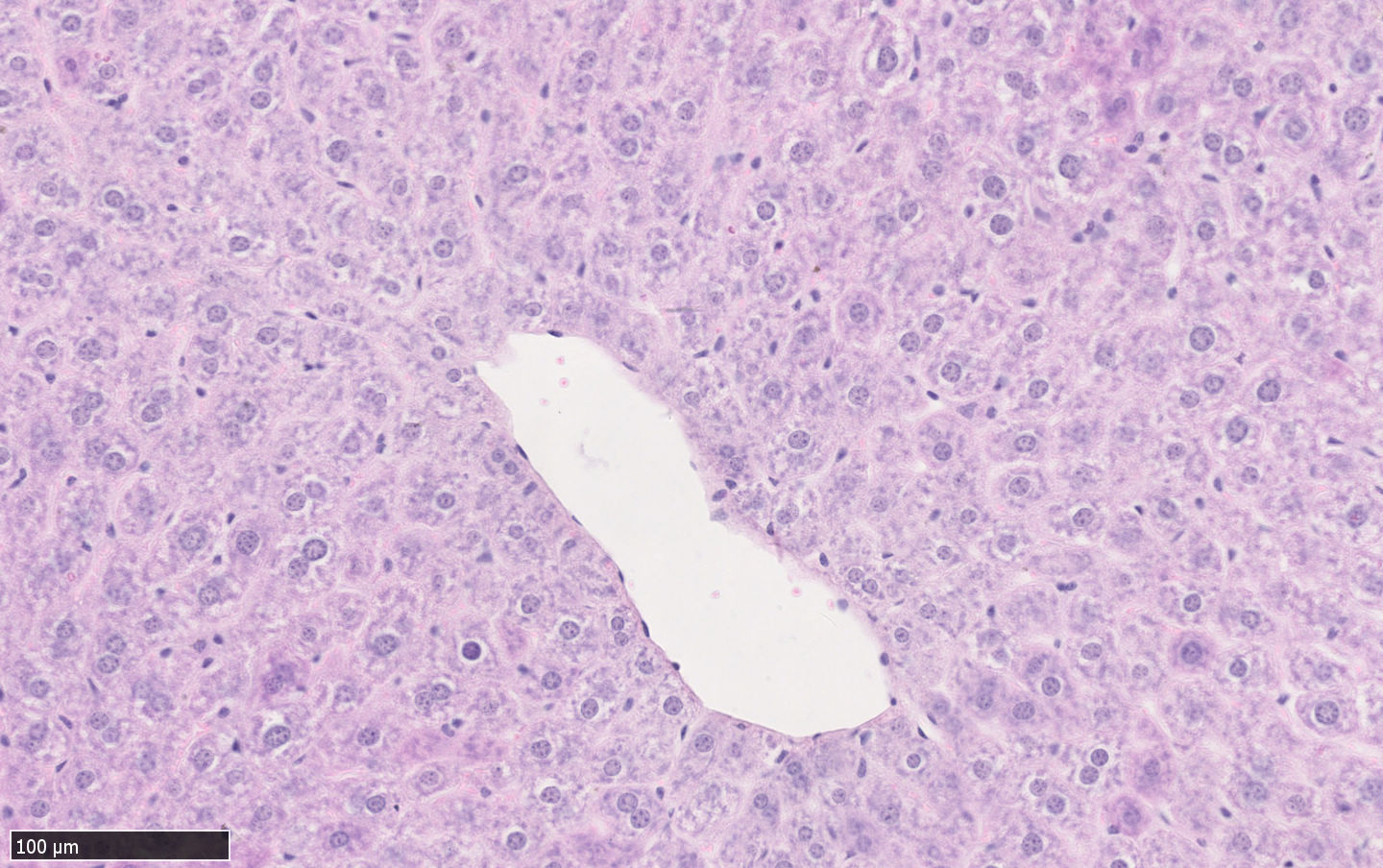

Supplement: Supplementary file 3 [file DataSheet8.ZIP › NASH SCORE-WT/WT9,10/20.jpg]

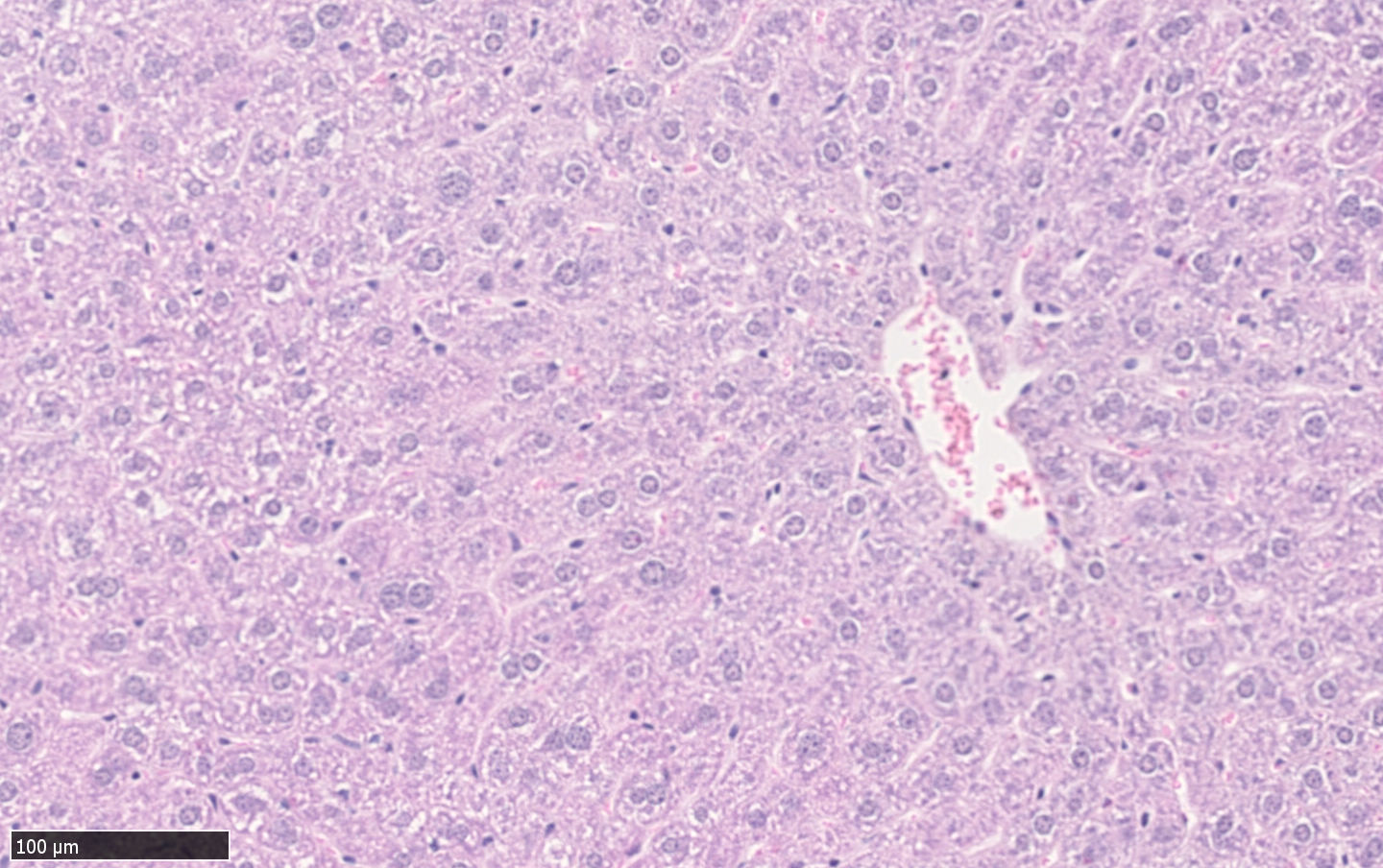

Supplement: Supplementary file 3 [file DataSheet8.ZIP › NASH SCORE-WT/WT9,10/3.jpg]

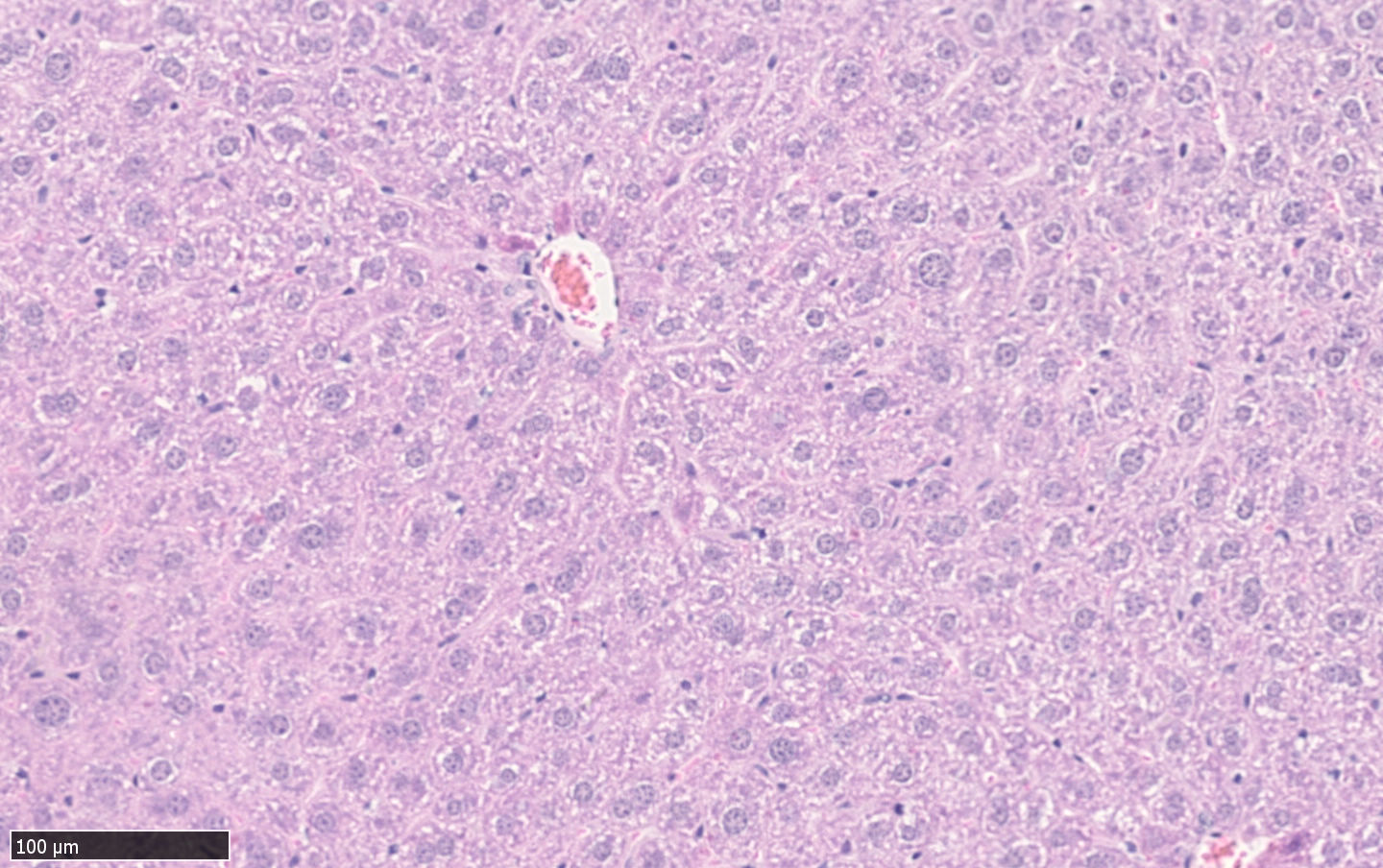

Supplement: Supplementary file 3 [file DataSheet8.ZIP › NASH SCORE-WT/WT9,10/4.jpg]

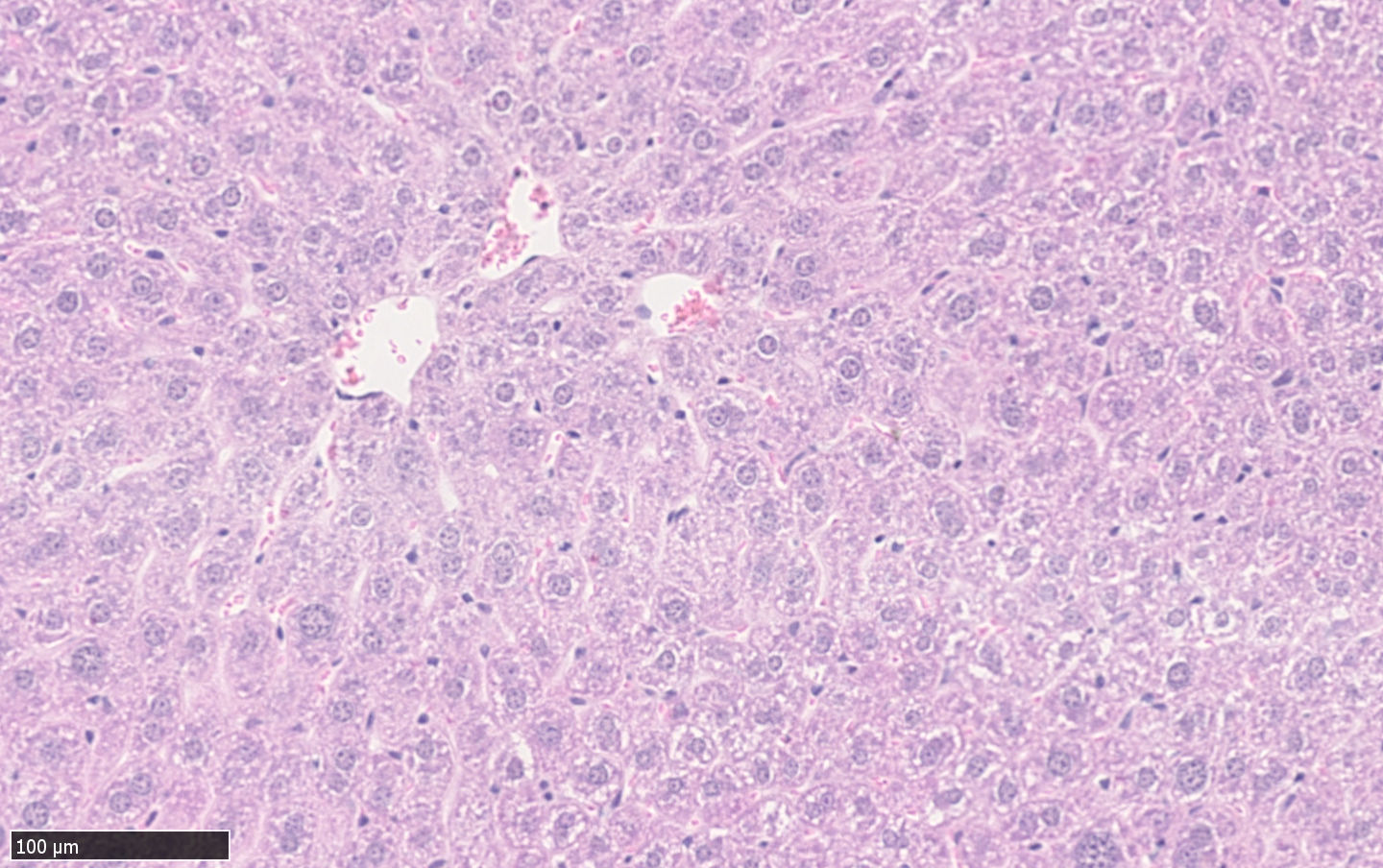

Supplement: Supplementary file 3 [file DataSheet8.ZIP › NASH SCORE-WT/WT9,10/5.jpg]

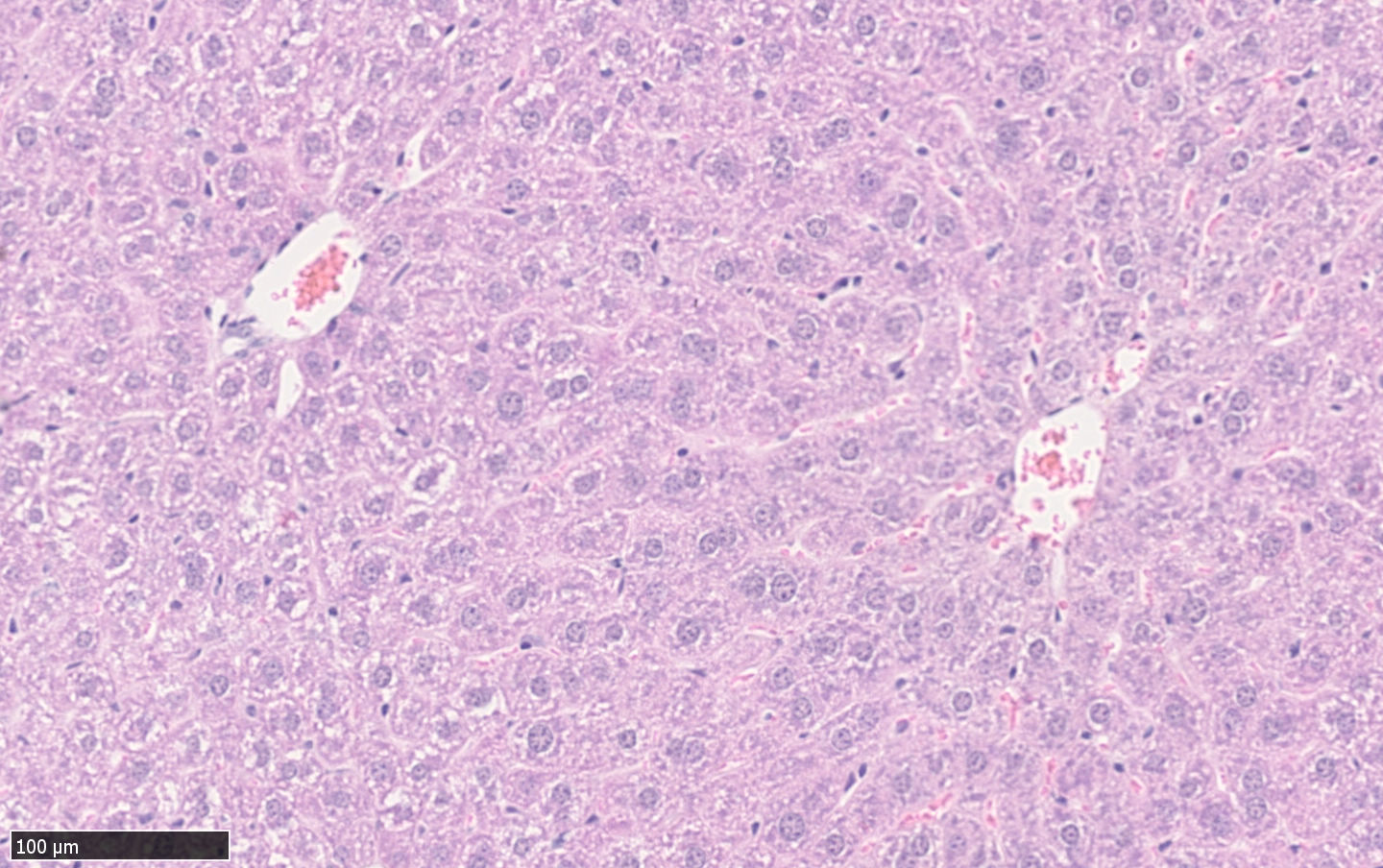

Supplement: Supplementary file 3 [file DataSheet8.ZIP › NASH SCORE-WT/WT9,10/6.jpg]

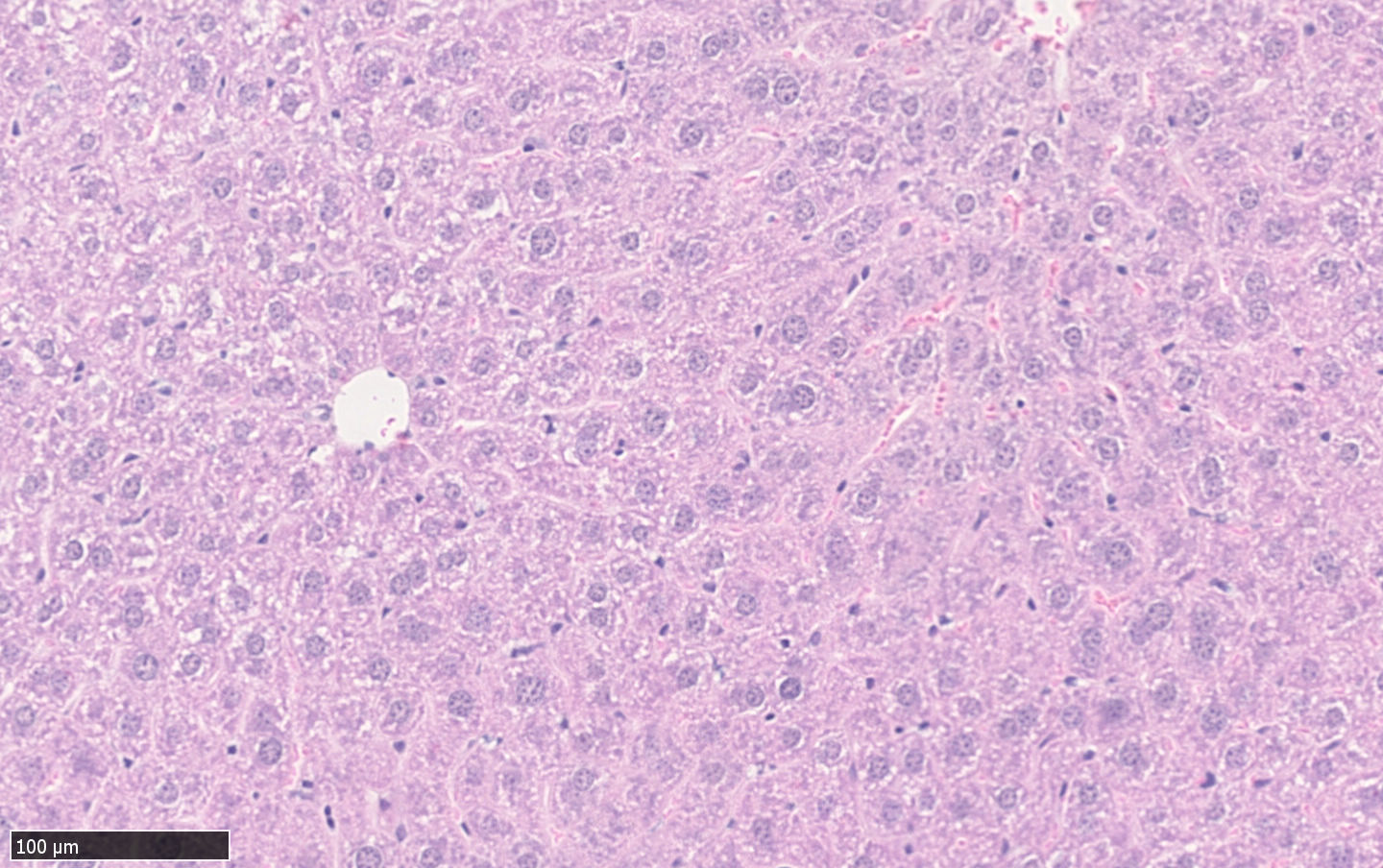

Supplement: Supplementary file 3 [file DataSheet8.ZIP › NASH SCORE-WT/WT9,10/7.jpg]

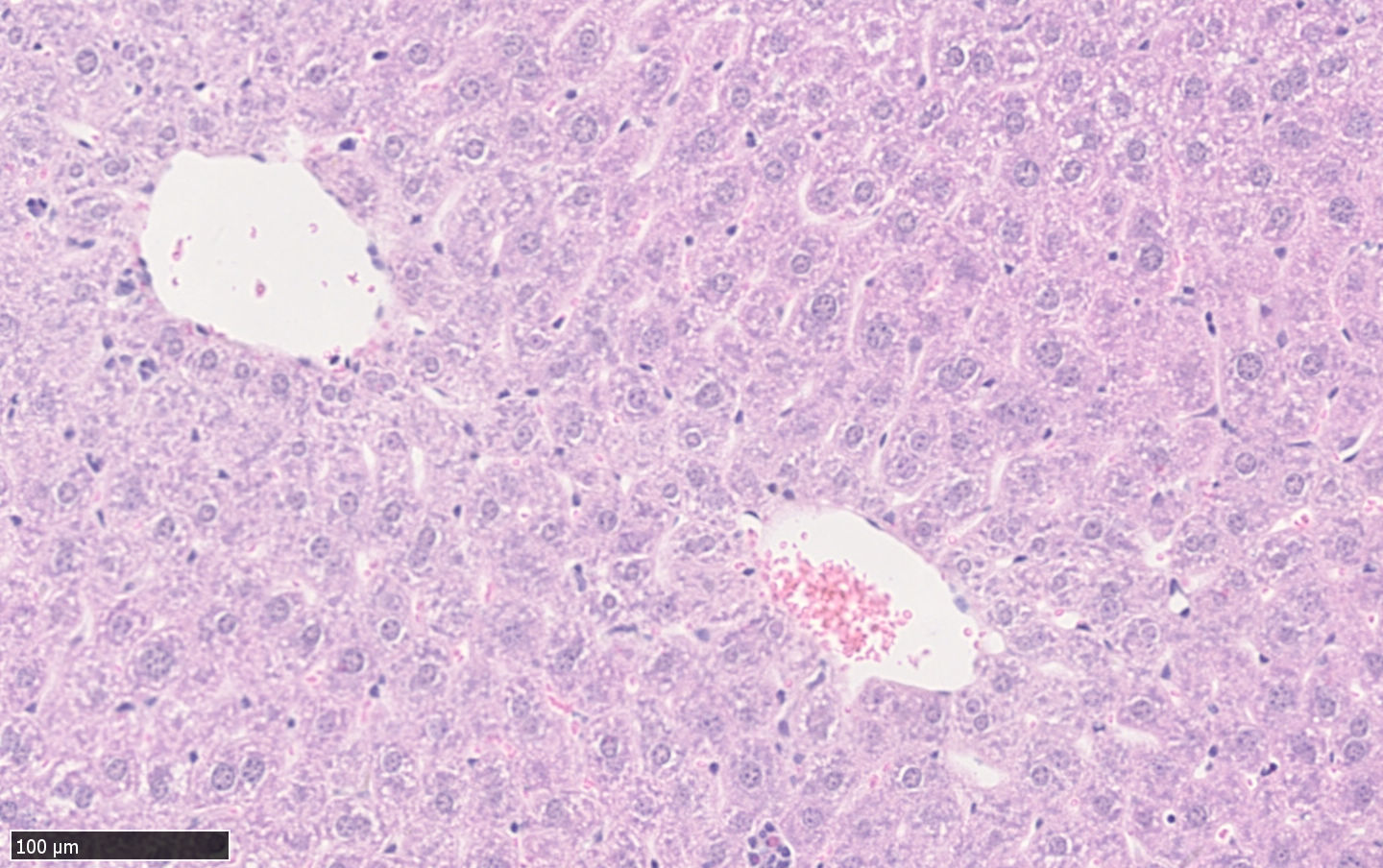

Supplement: Supplementary file 3 [file DataSheet8.ZIP › NASH SCORE-WT/WT9,10/8.jpg]

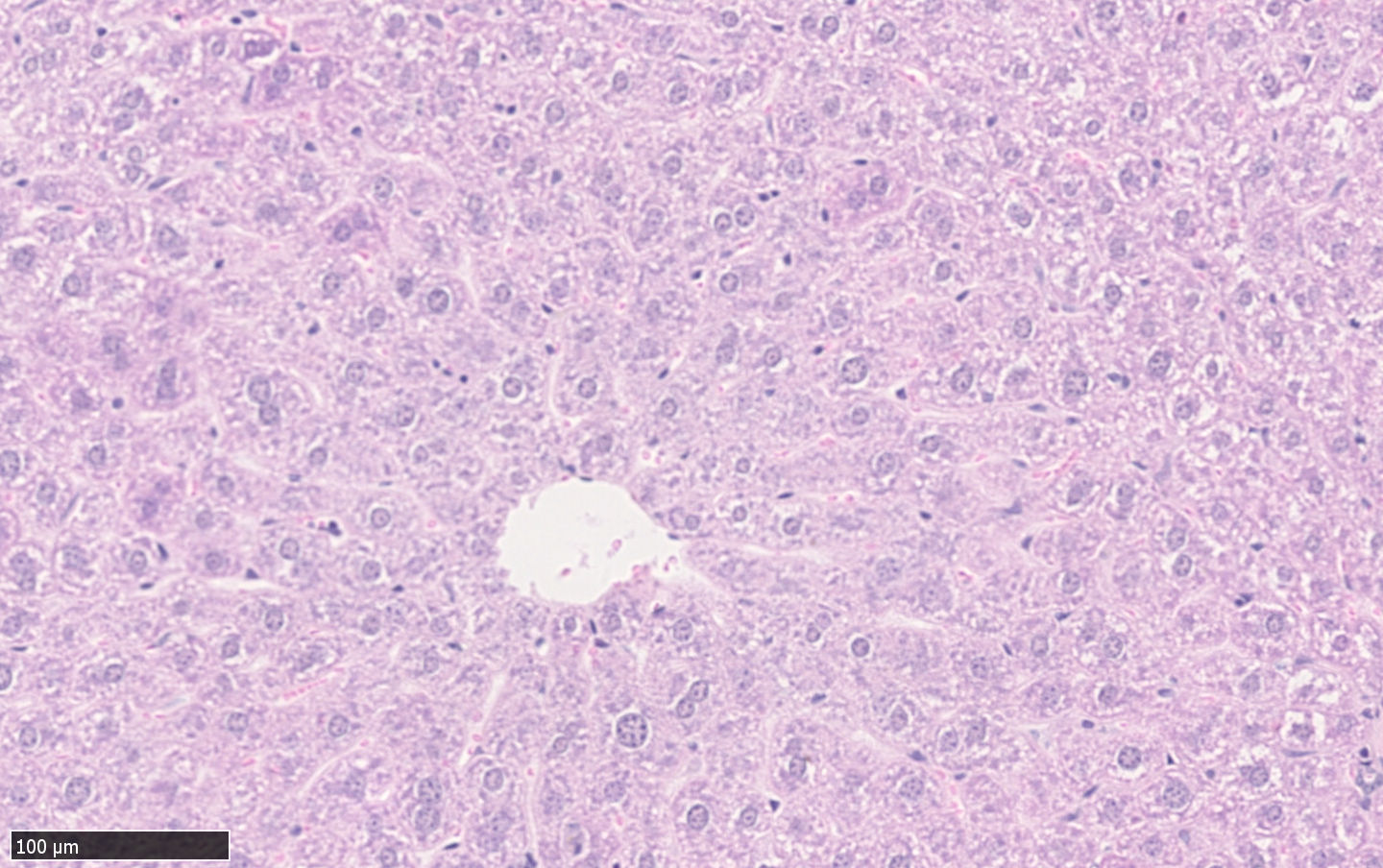

Supplement: Supplementary file 3 [file DataSheet8.ZIP › NASH SCORE-WT/WT9,10/9.jpg]

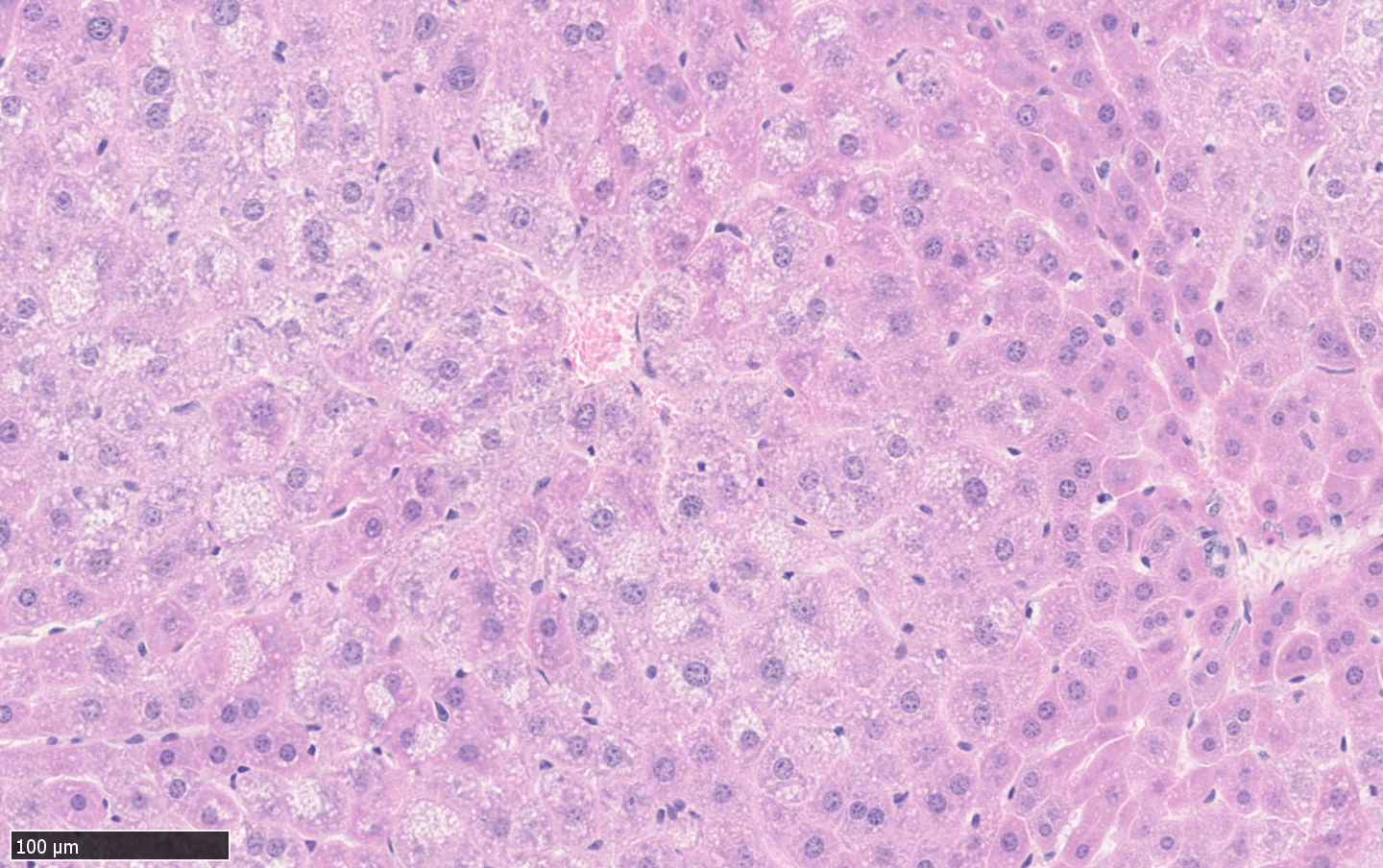

Supplement: Supplementary file 4 [file DataSheet9.ZIP › NASH SCORE-db(1)/db10,11/1.jpg]

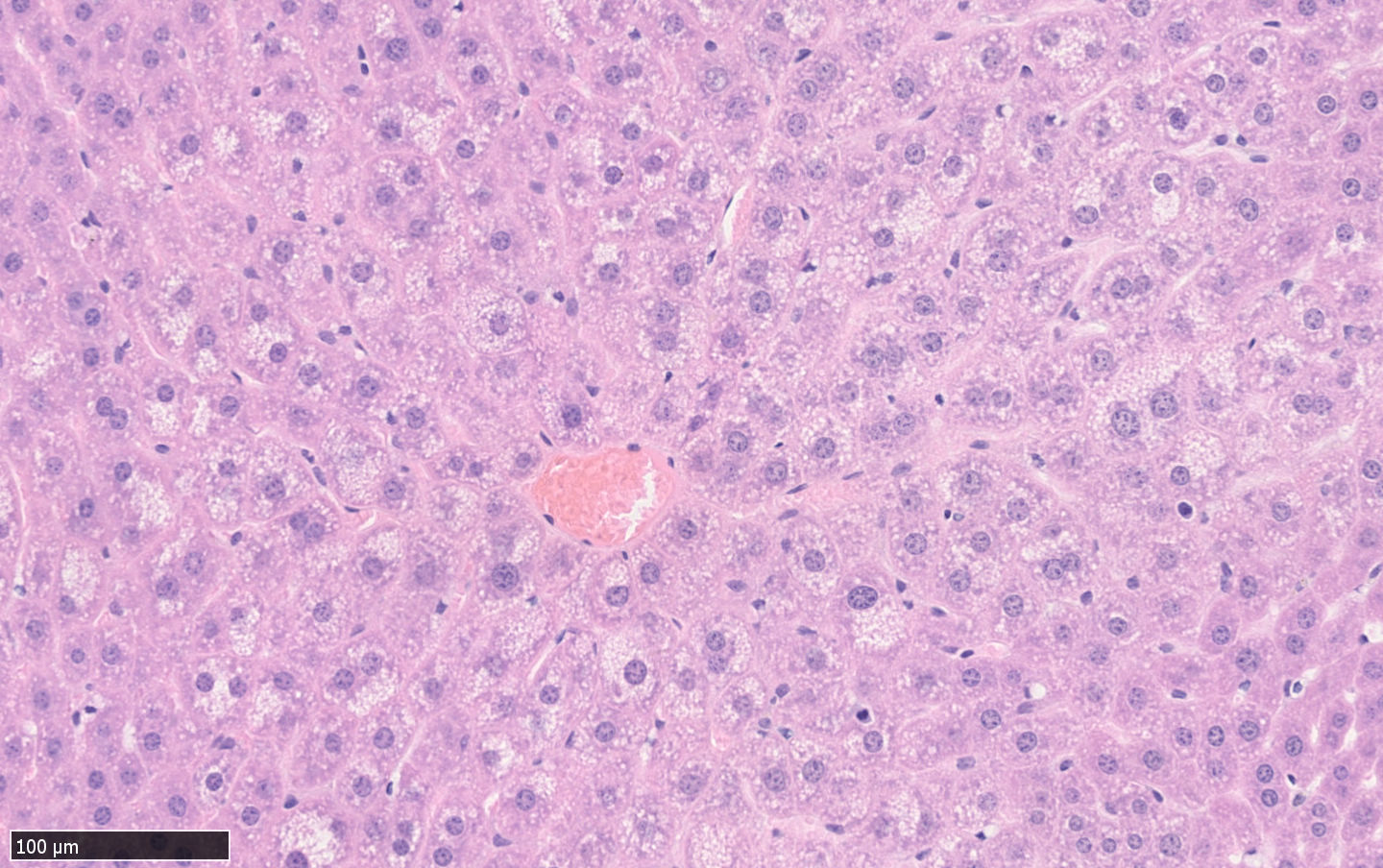

Supplement: Supplementary file 4 [file DataSheet9.ZIP › NASH SCORE-db(1)/db10,11/10.jpg]

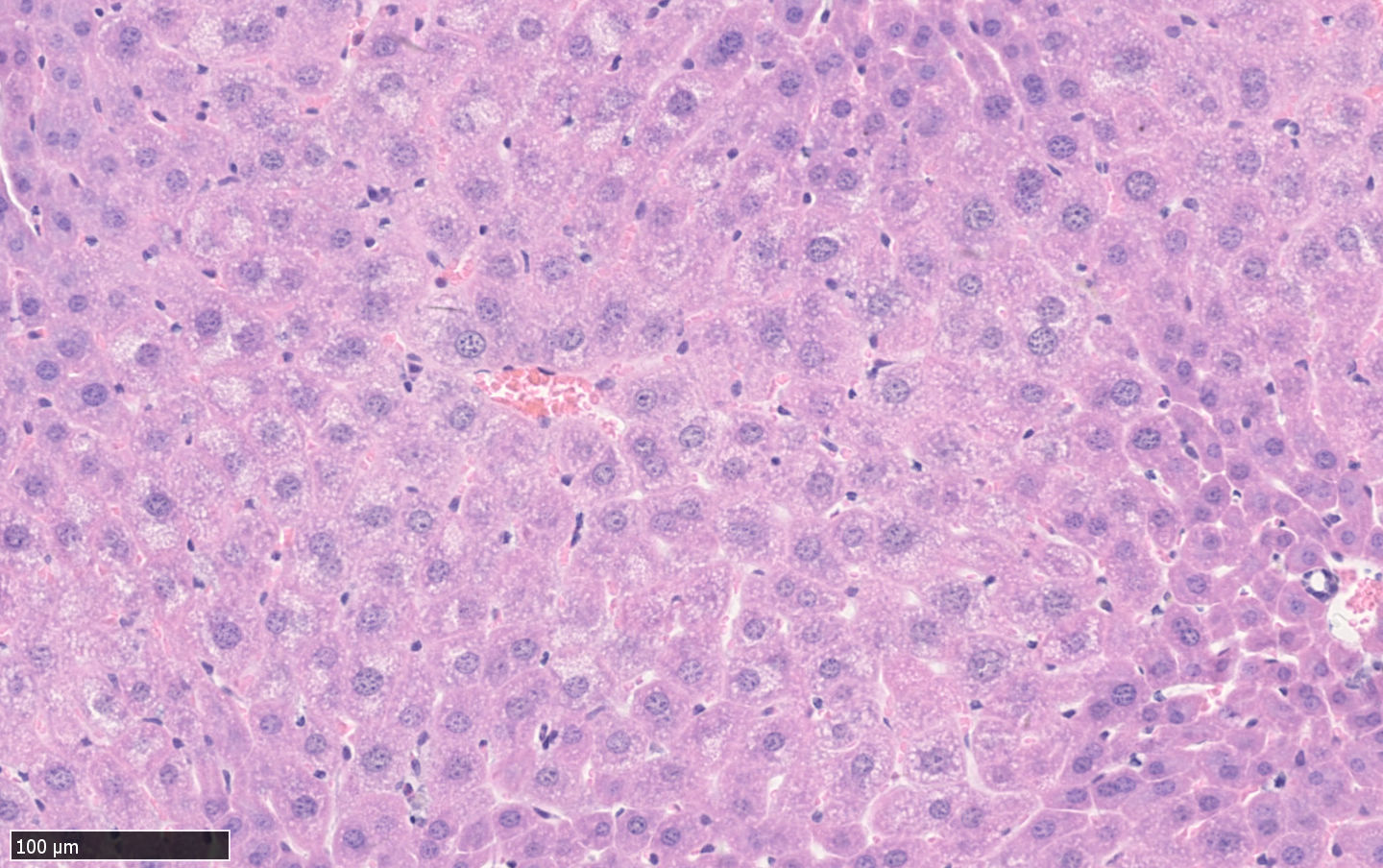

Supplement: Supplementary file 4 [file DataSheet9.ZIP › NASH SCORE-db(1)/db10,11/11.jpg]

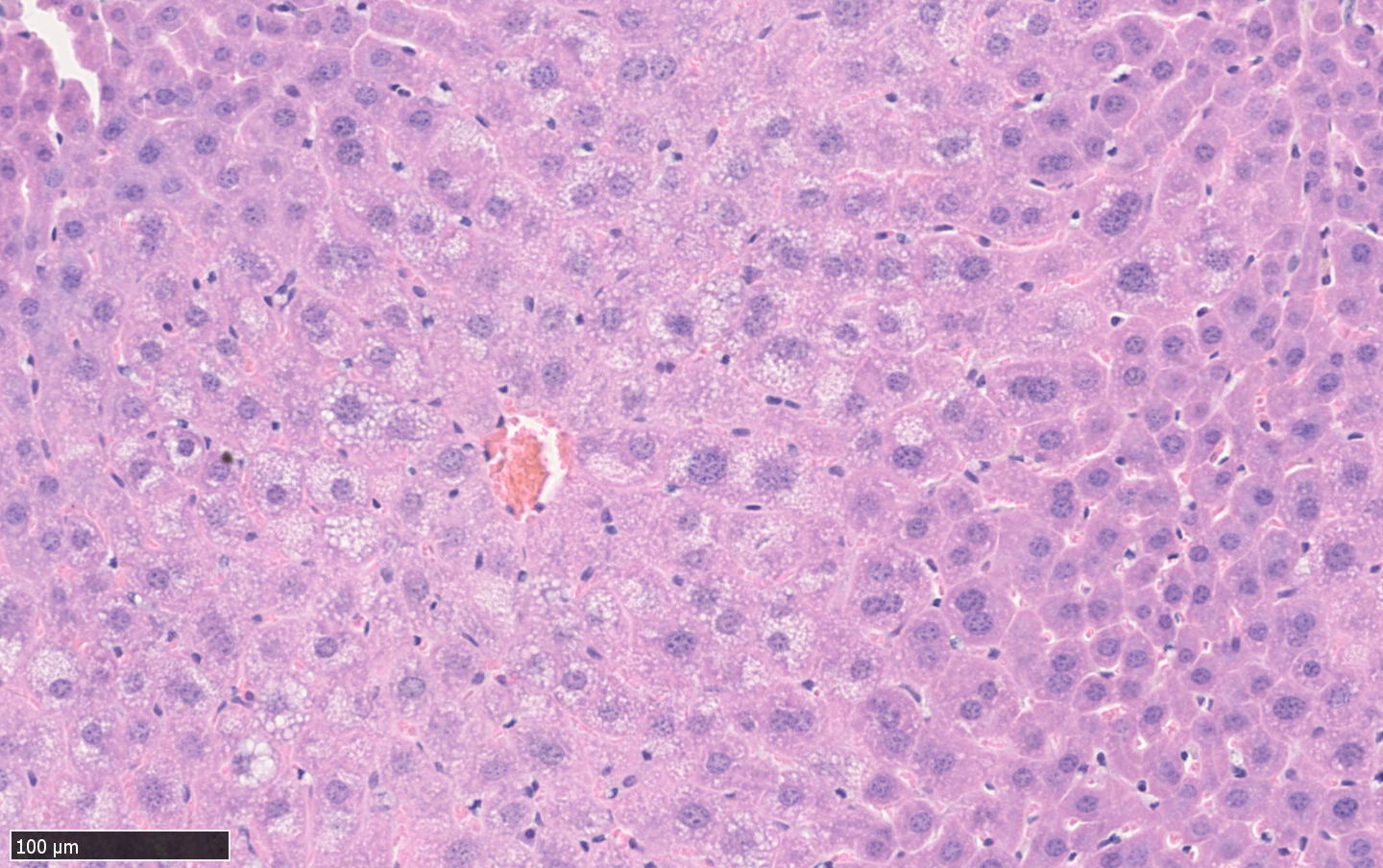

Supplement: Supplementary file 4 [file DataSheet9.ZIP › NASH SCORE-db(1)/db10,11/12.jpg]
