# Supplementary material for: Dapagliflozin protects against nonalcoholic steatohepatitis in db/db mice
Source: Front Pharmacol. 2022 Aug 19;13:934136. doi: 10.3389/fphar.2022.934136 (PMC9437261; doi:10.3389/fphar.2022.934136)
Supplement: Supplementary file 2 [file DataSheet11.ZIP › oil red O liver/新建 Microsoft Office Word 文档.docx]

将显微镜拍摄20倍图片直接导入canvas后，调成2.260（宽），1.702(高），再将切成1.613（宽），0.968(高）。
