# Supplementary figures and images for: Dapagliflozin protects against nonalcoholic steatohepatitis in db/db mice (part 2 of 3)
Source: Front Pharmacol. 2022 Aug 19;13:934136. doi: 10.3389/fphar.2022.934136 (PMC9437261; doi:10.3389/fphar.2022.934136)

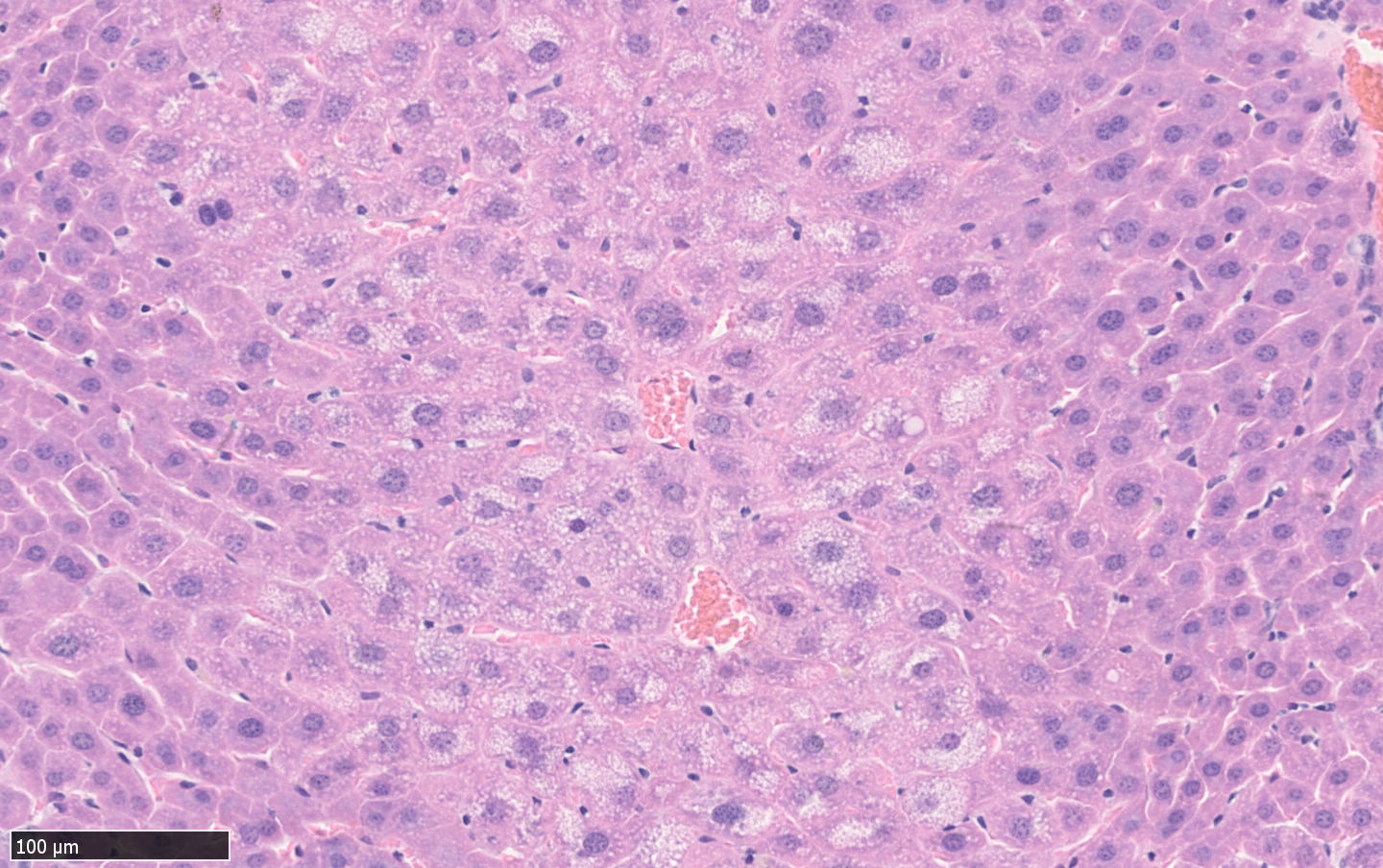

Supplement: Supplementary file 4 [file DataSheet9.ZIP › NASH SCORE-db(1)/db10,11/13.jpg]

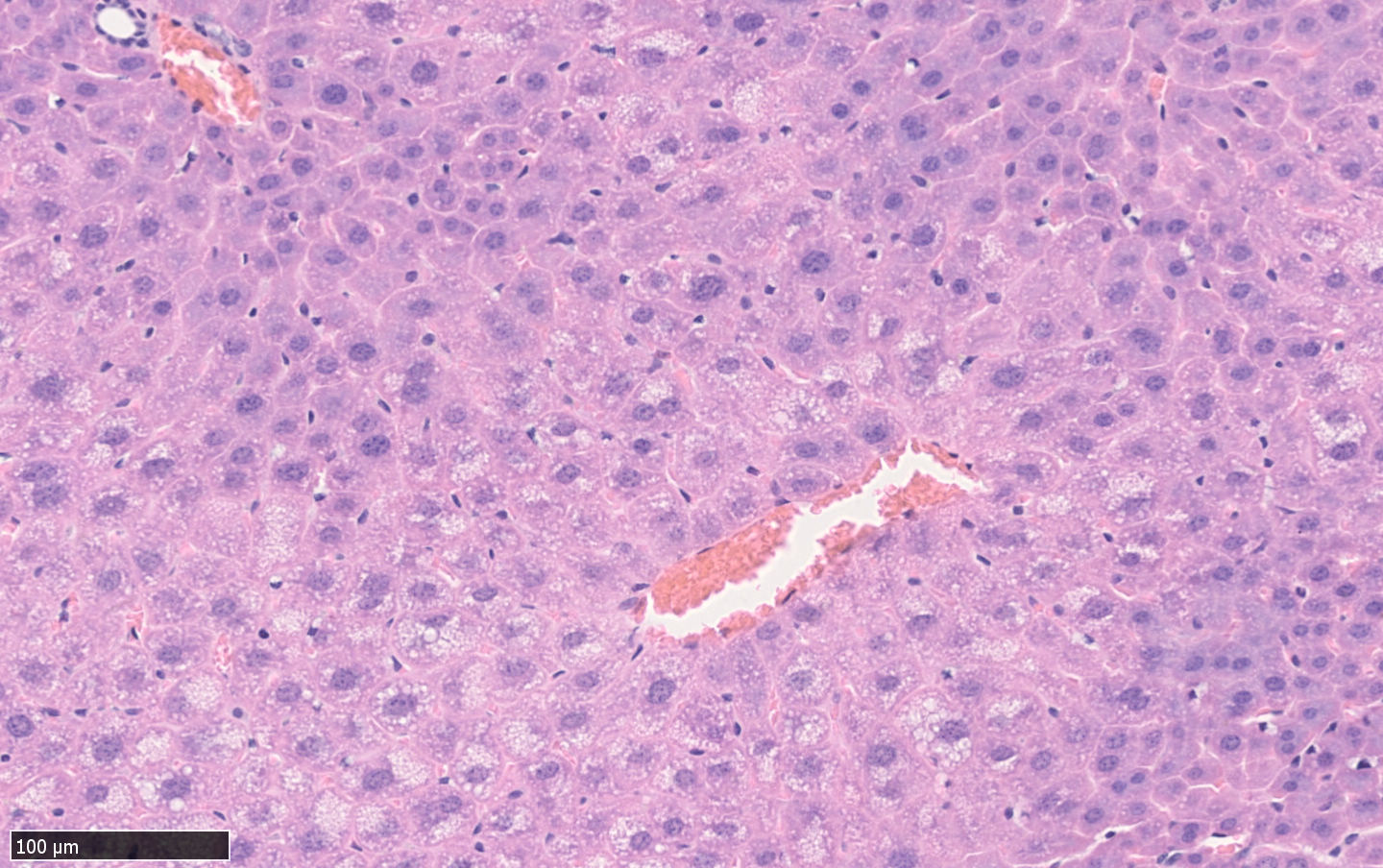

Supplement: Supplementary file 4 [file DataSheet9.ZIP › NASH SCORE-db(1)/db10,11/14.jpg]

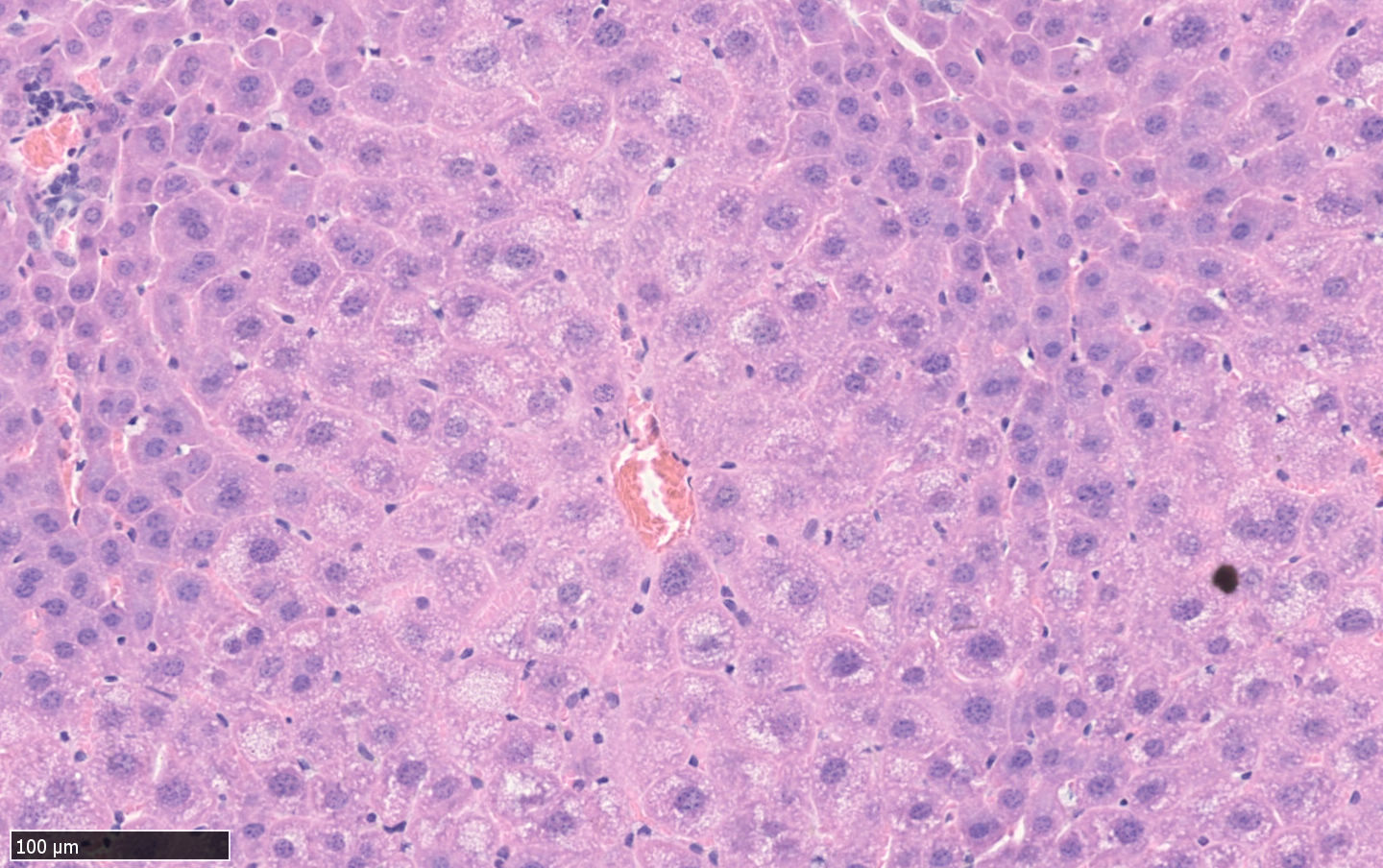

Supplement: Supplementary file 4 [file DataSheet9.ZIP › NASH SCORE-db(1)/db10,11/15.jpg]

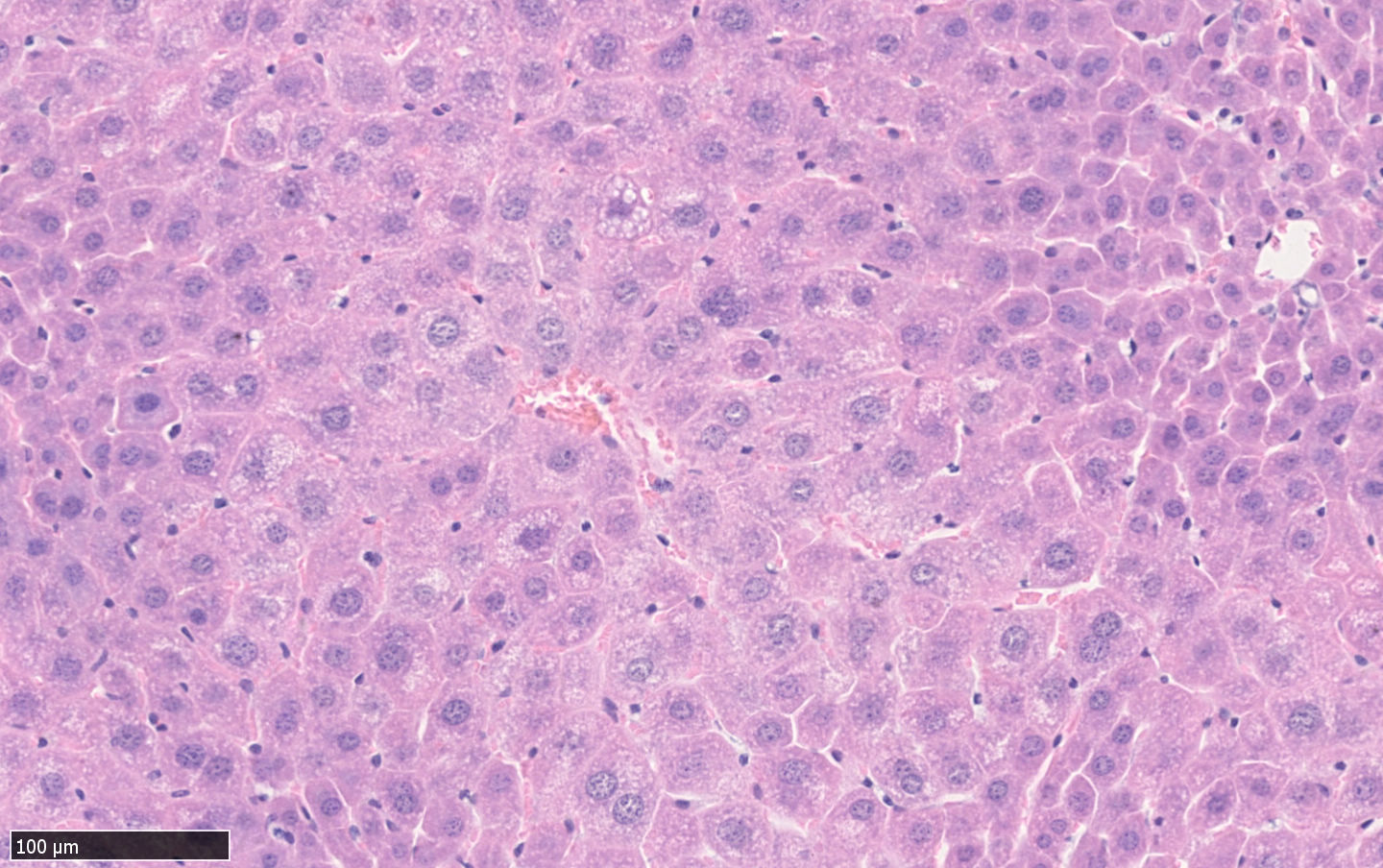

Supplement: Supplementary file 4 [file DataSheet9.ZIP › NASH SCORE-db(1)/db10,11/16.jpg]

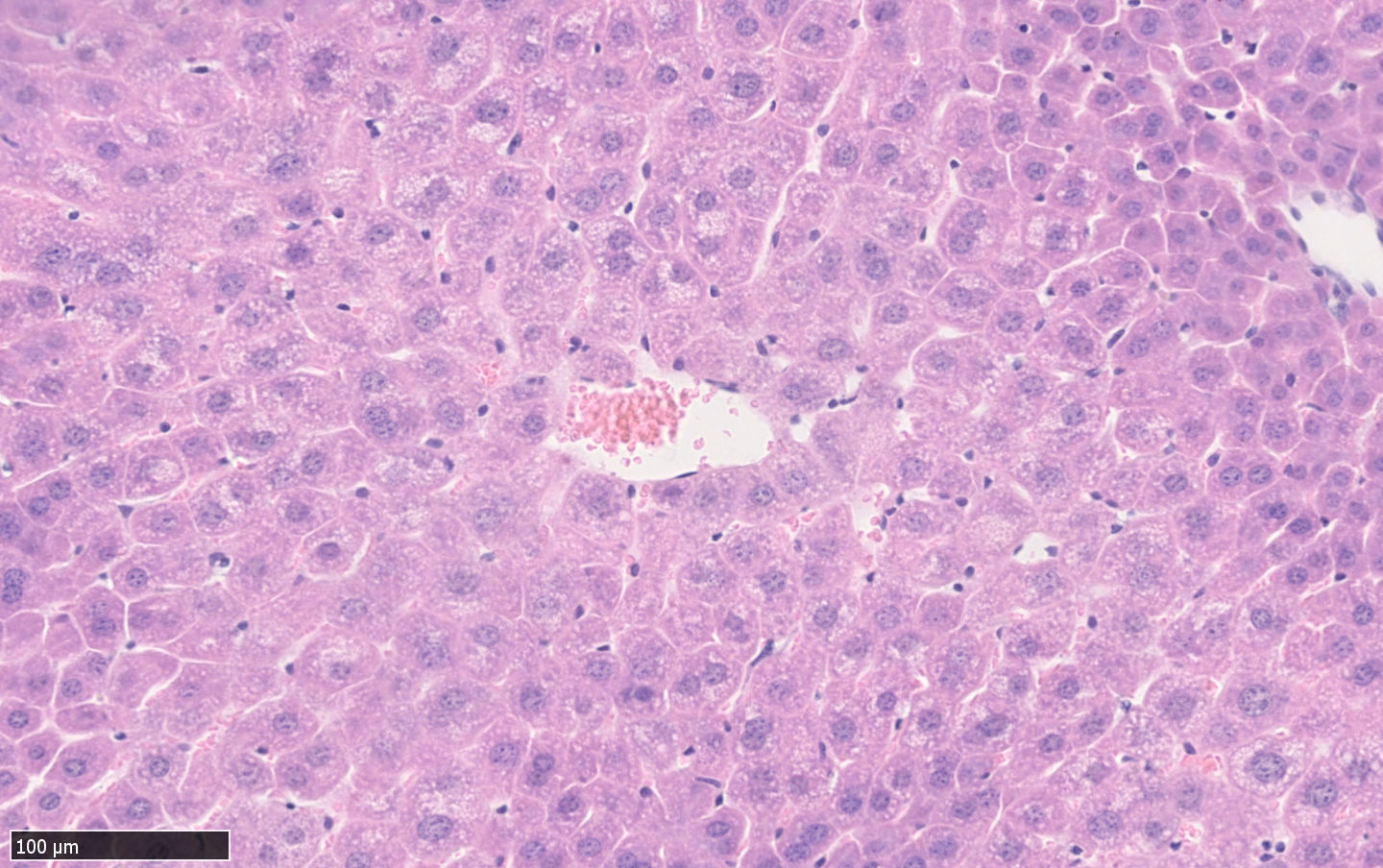

Supplement: Supplementary file 4 [file DataSheet9.ZIP › NASH SCORE-db(1)/db10,11/17.jpg]

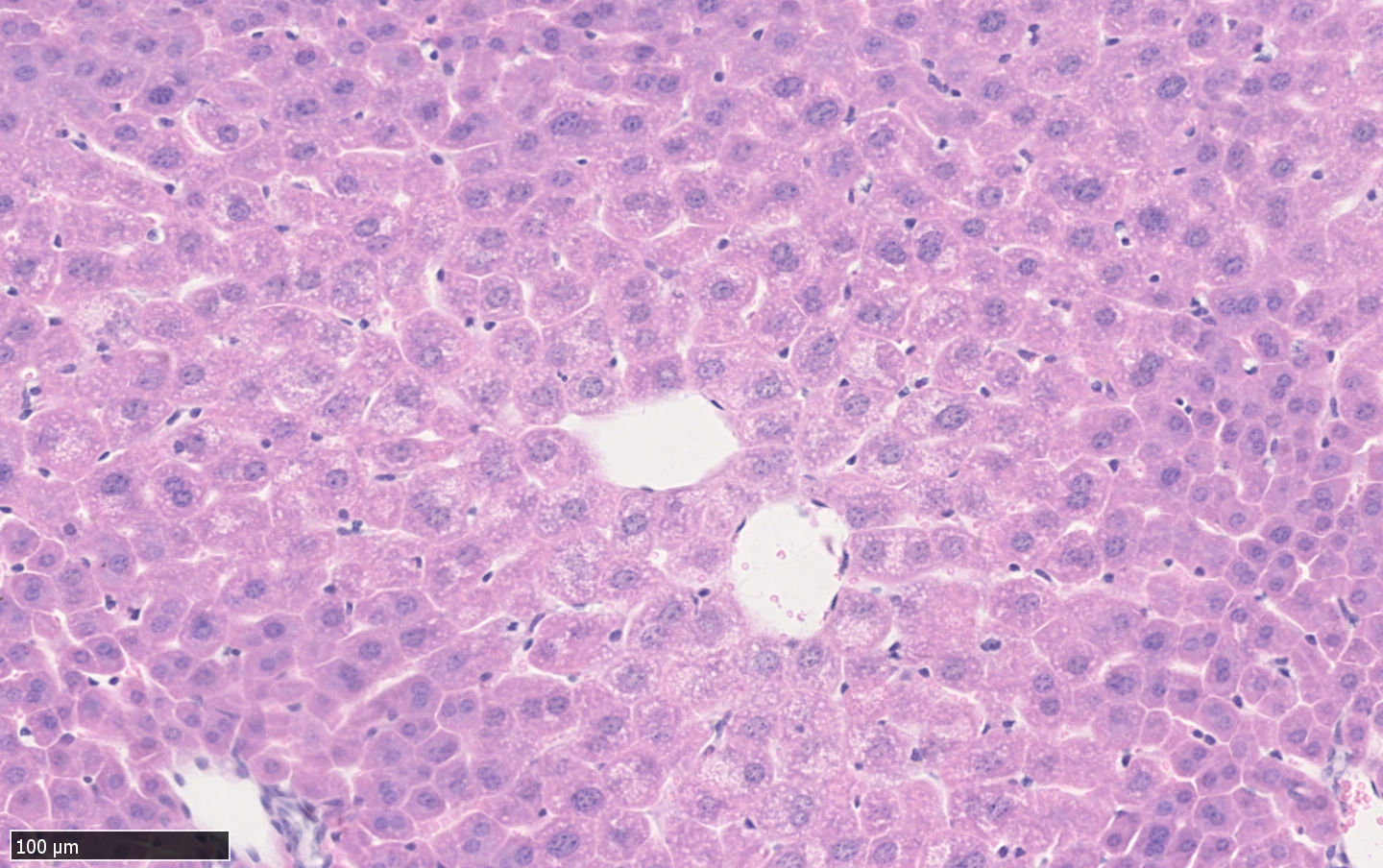

Supplement: Supplementary file 4 [file DataSheet9.ZIP › NASH SCORE-db(1)/db10,11/18.jpg]

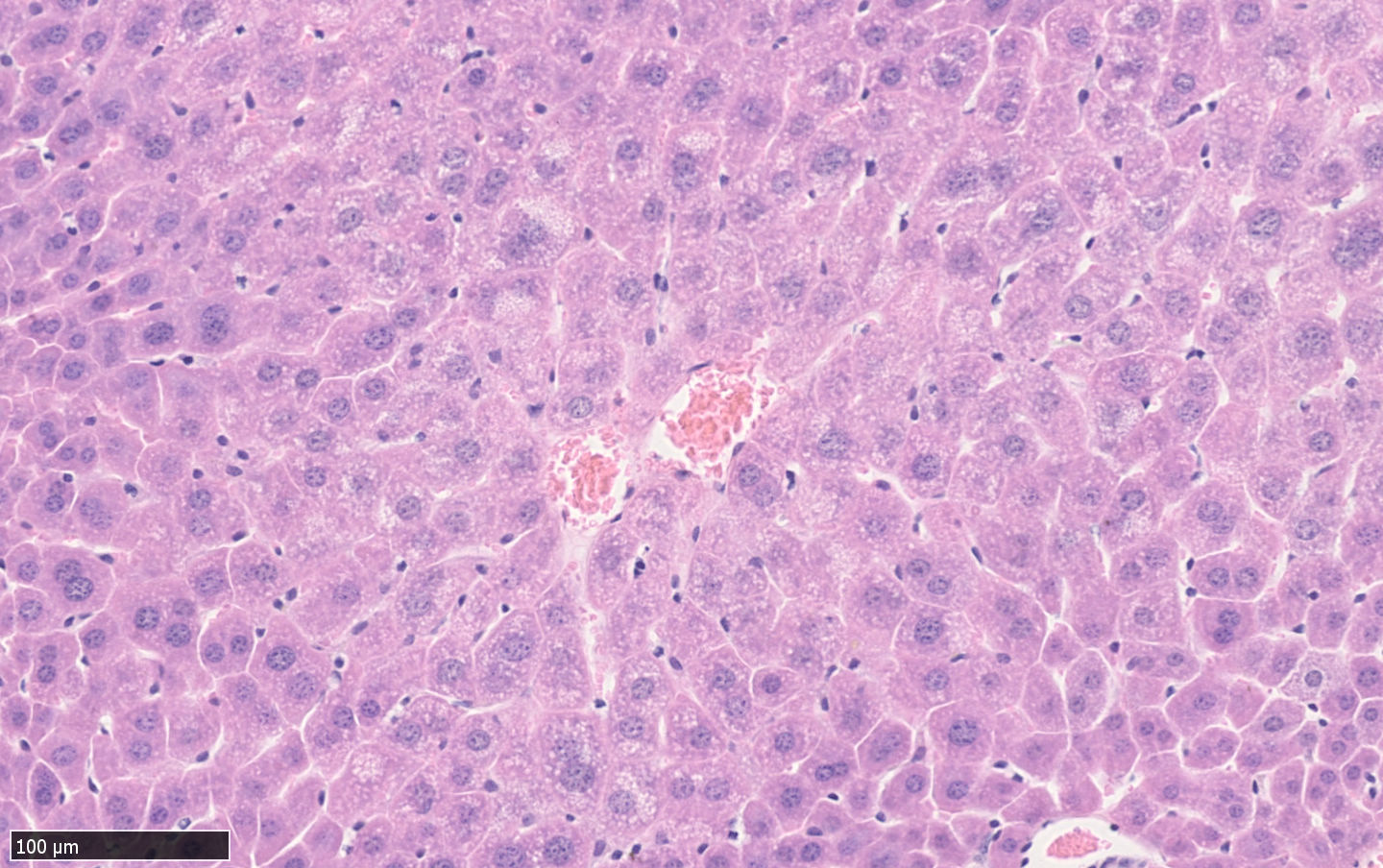

Supplement: Supplementary file 4 [file DataSheet9.ZIP › NASH SCORE-db(1)/db10,11/19.jpg]

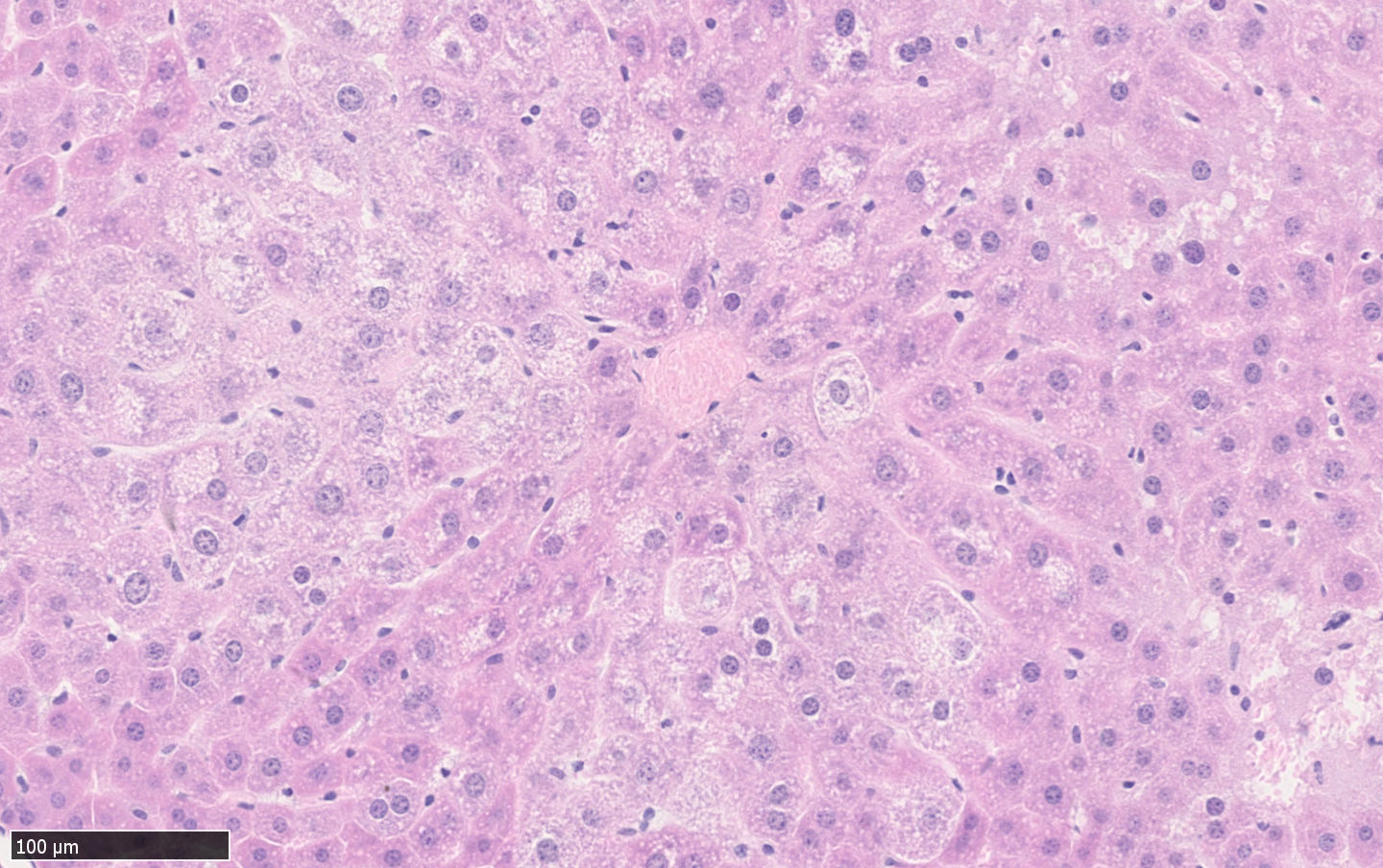

Supplement: Supplementary file 4 [file DataSheet9.ZIP › NASH SCORE-db(1)/db10,11/2.jpg]

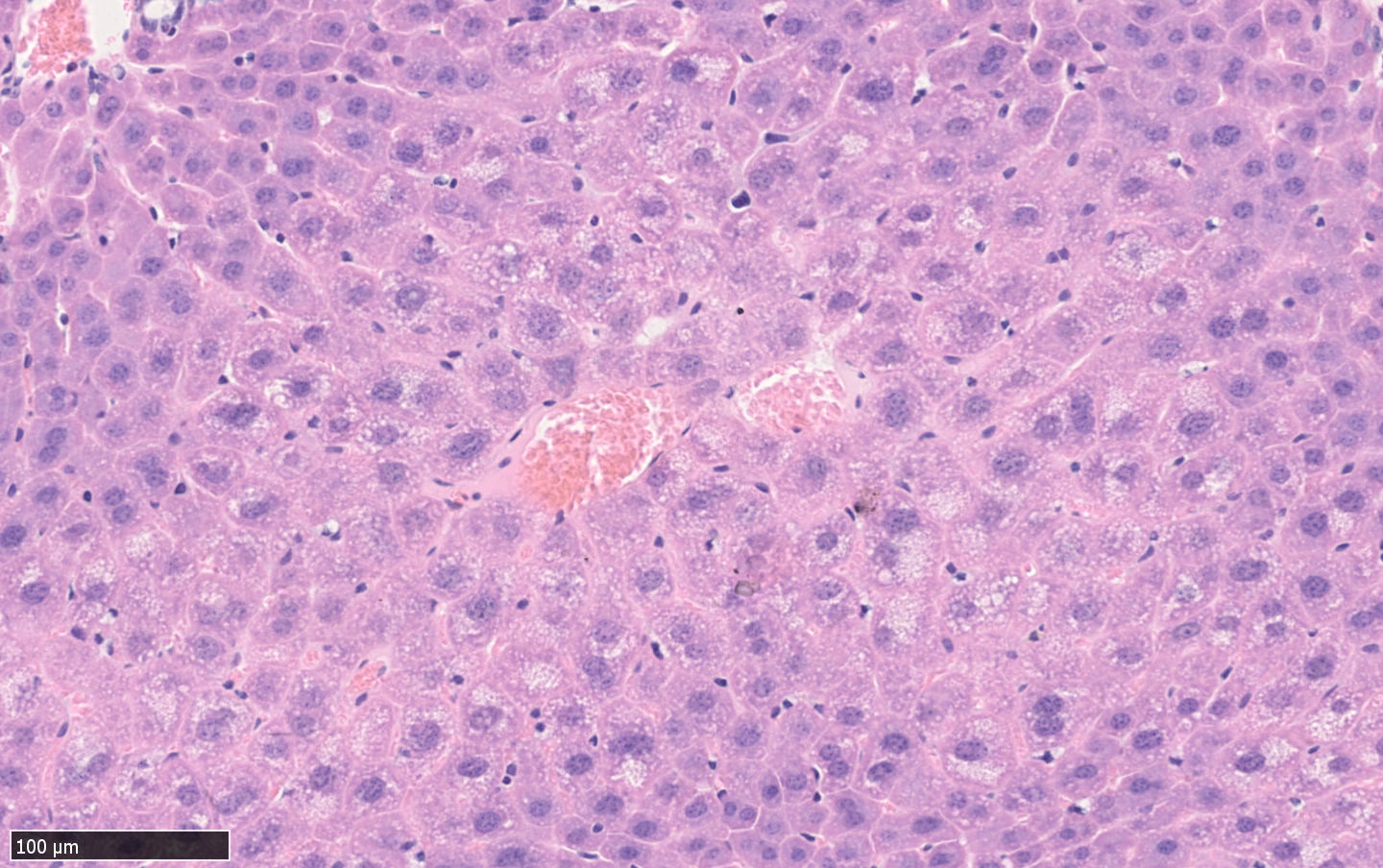

Supplement: Supplementary file 4 [file DataSheet9.ZIP › NASH SCORE-db(1)/db10,11/20.jpg]

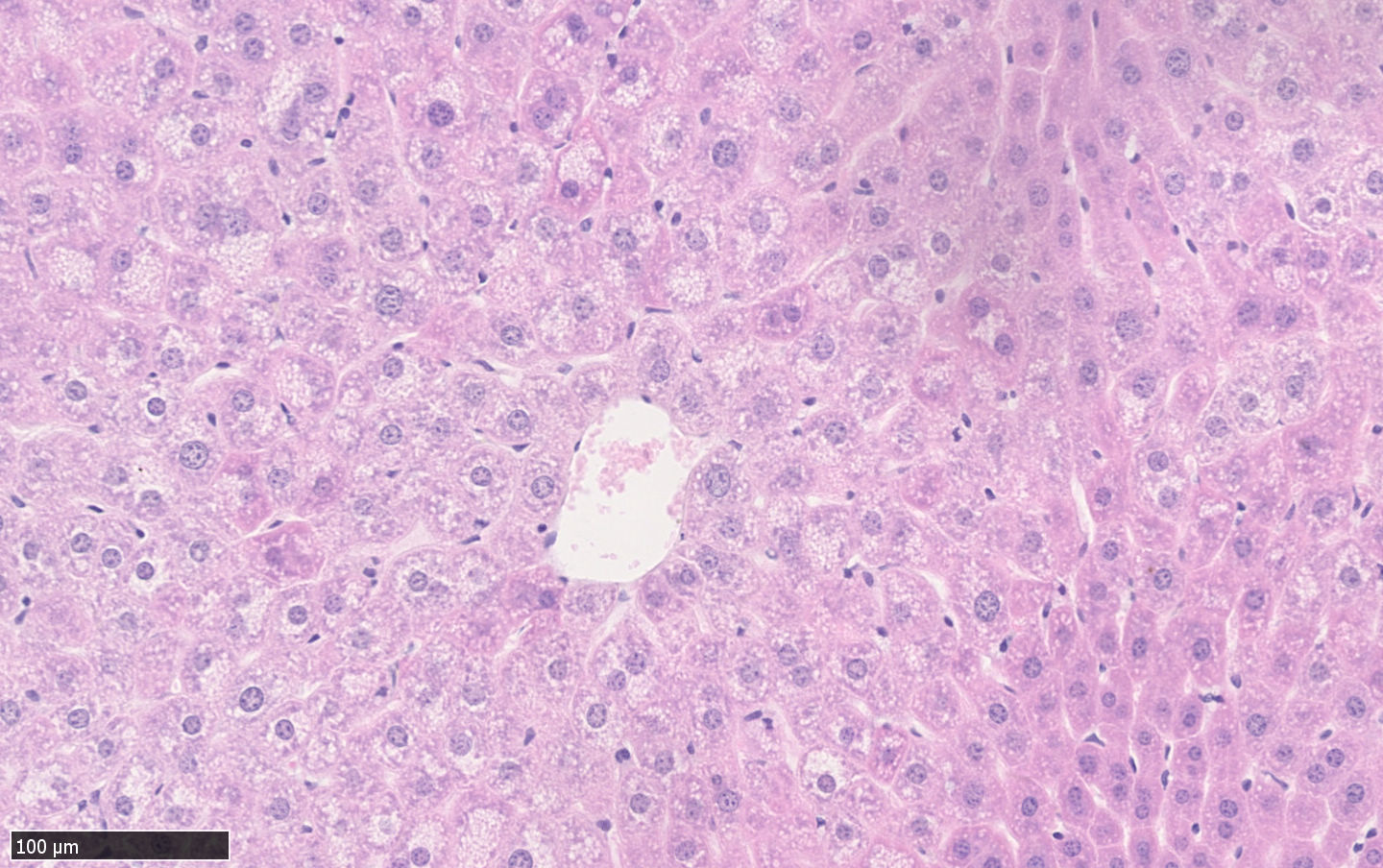

Supplement: Supplementary file 4 [file DataSheet9.ZIP › NASH SCORE-db(1)/db10,11/3.jpg]

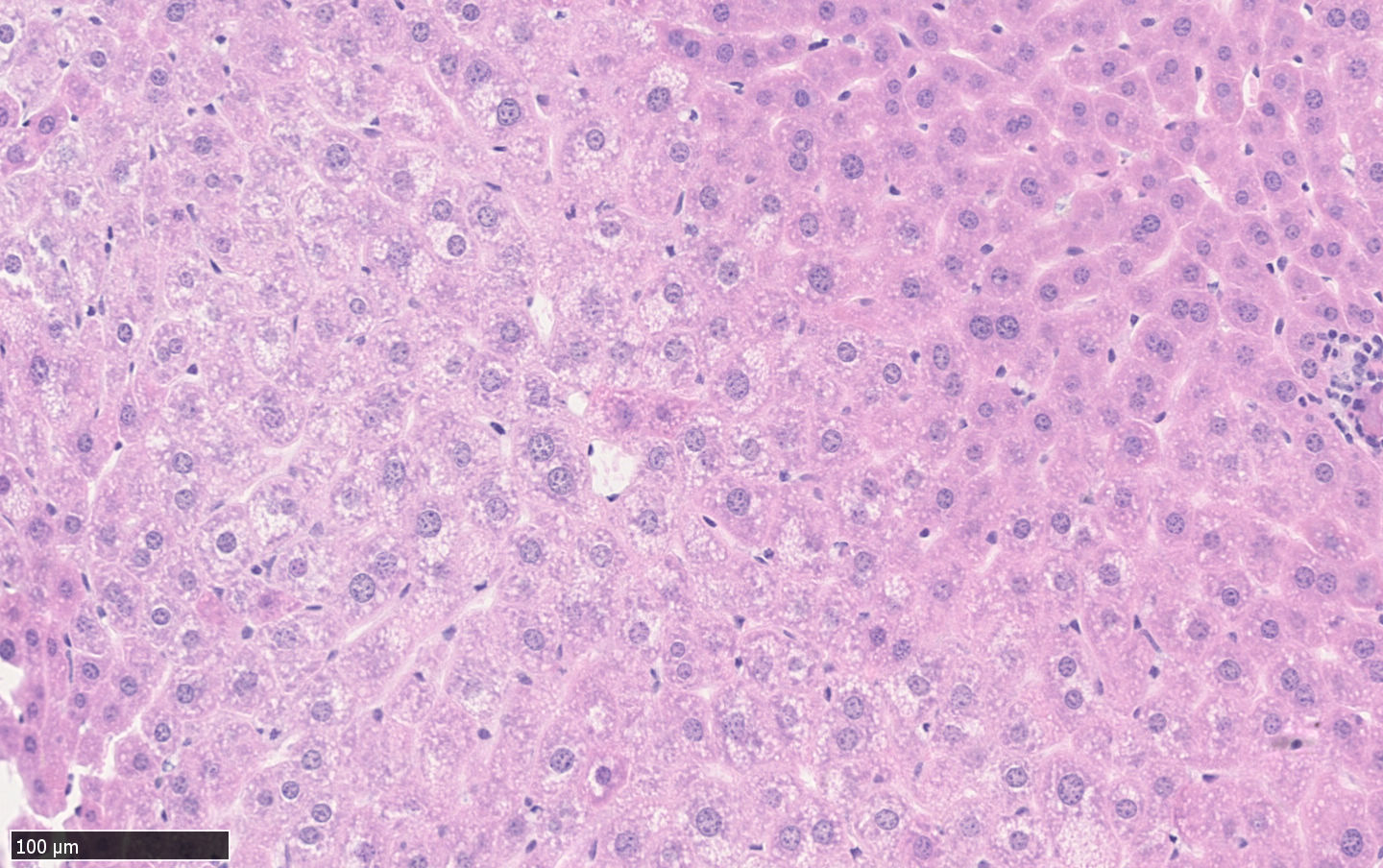

Supplement: Supplementary file 4 [file DataSheet9.ZIP › NASH SCORE-db(1)/db10,11/4.jpg]

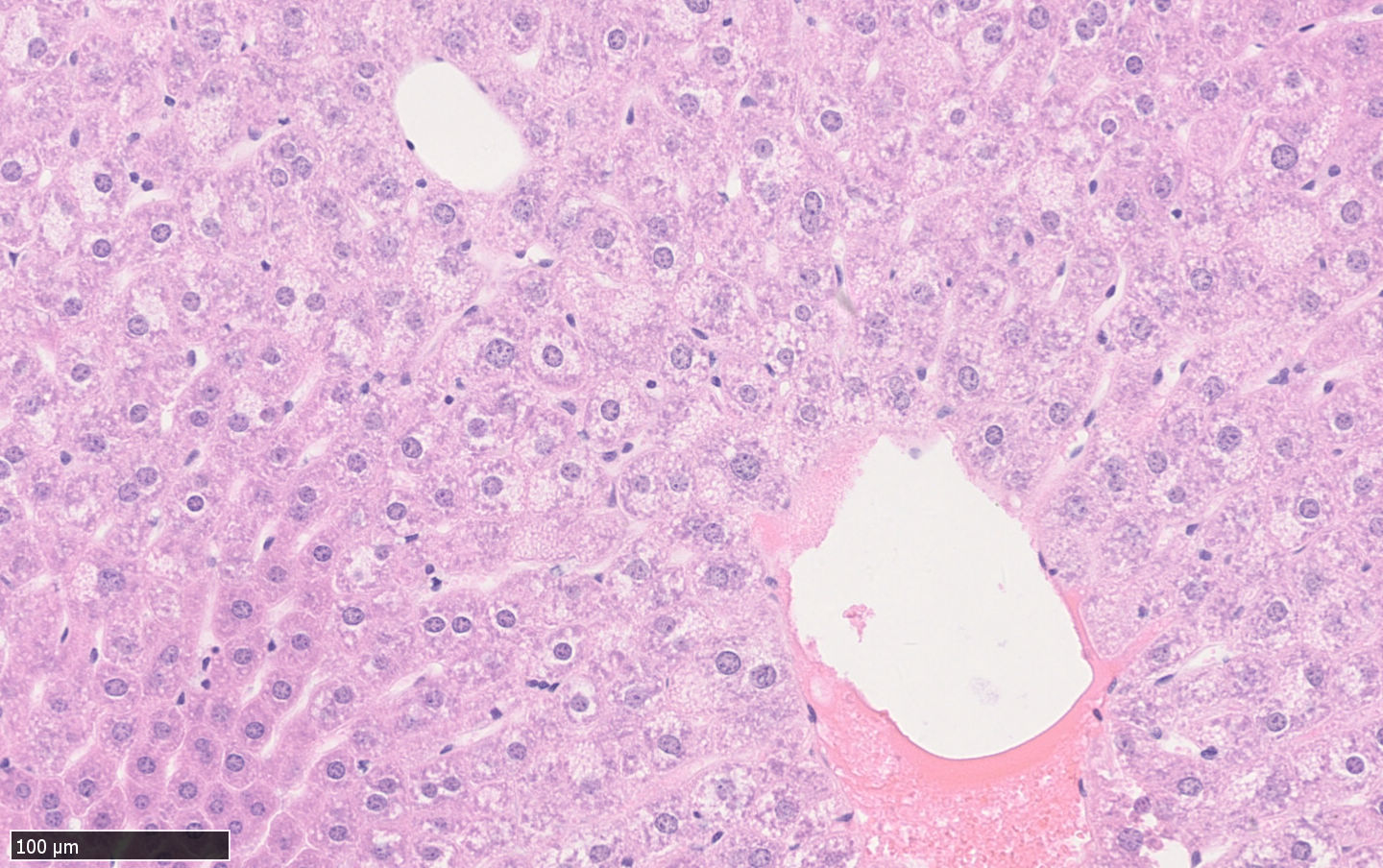

Supplement: Supplementary file 4 [file DataSheet9.ZIP › NASH SCORE-db(1)/db10,11/5.jpg]

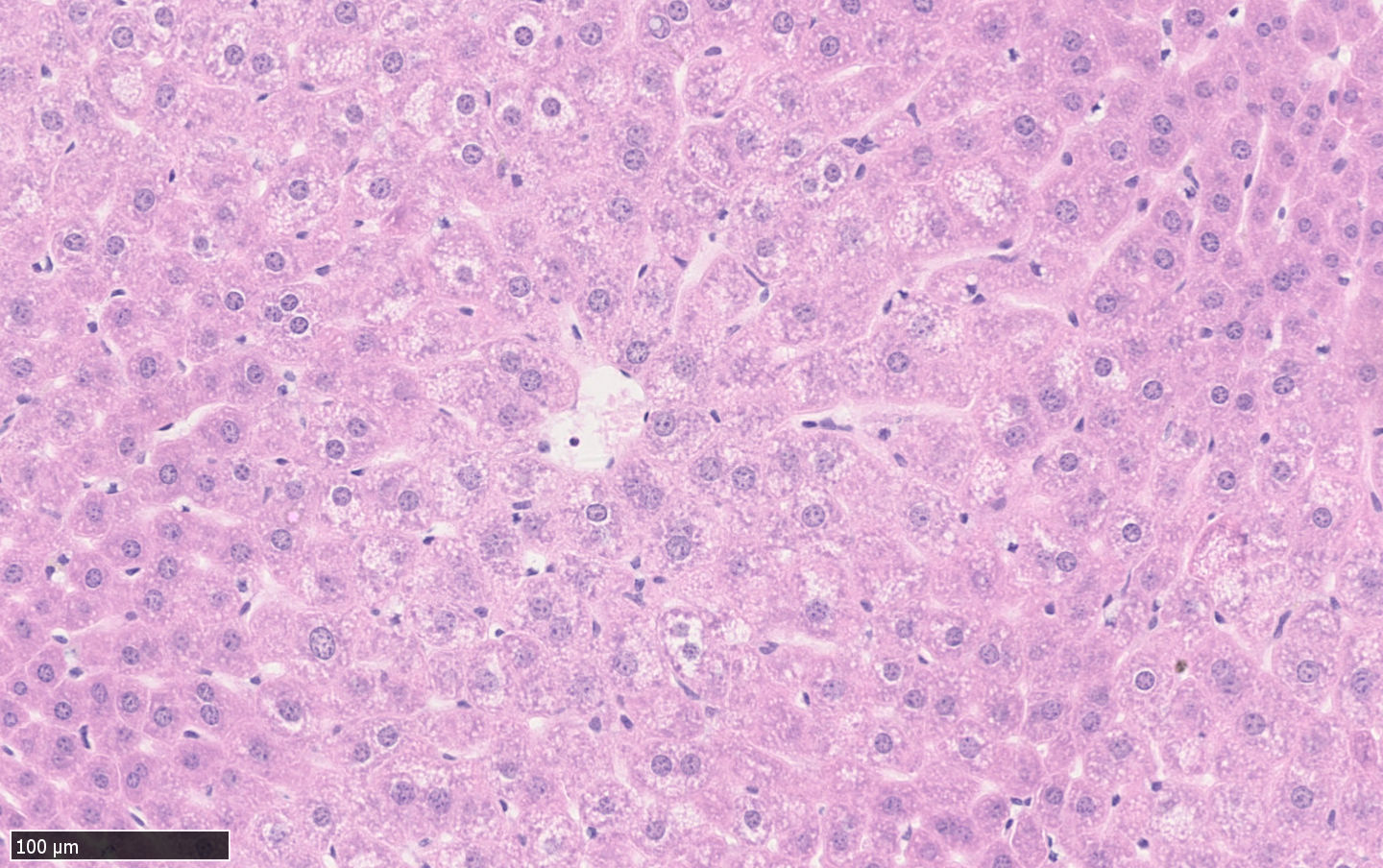

Supplement: Supplementary file 4 [file DataSheet9.ZIP › NASH SCORE-db(1)/db10,11/6.jpg]

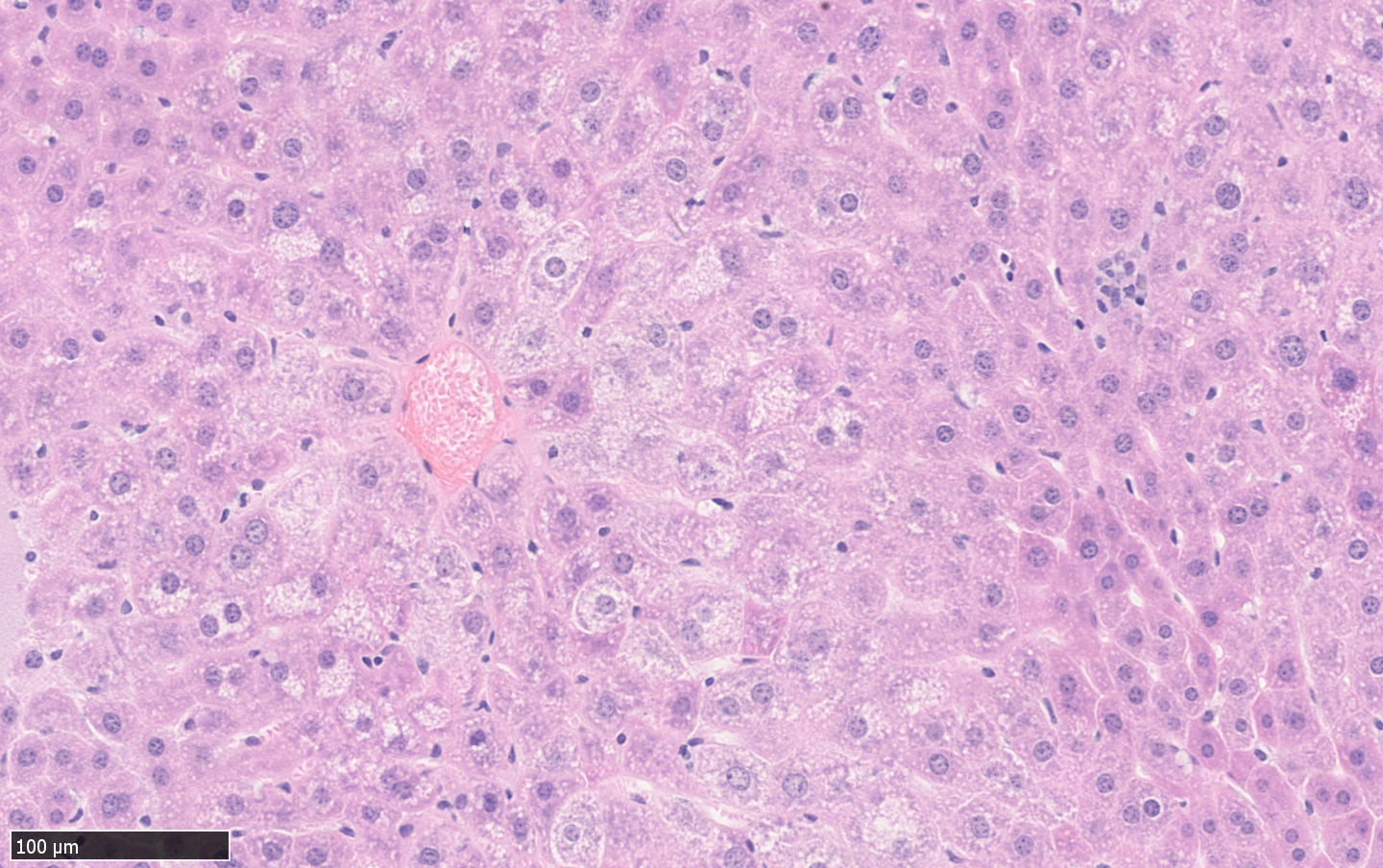

Supplement: Supplementary file 4 [file DataSheet9.ZIP › NASH SCORE-db(1)/db10,11/7.jpg]

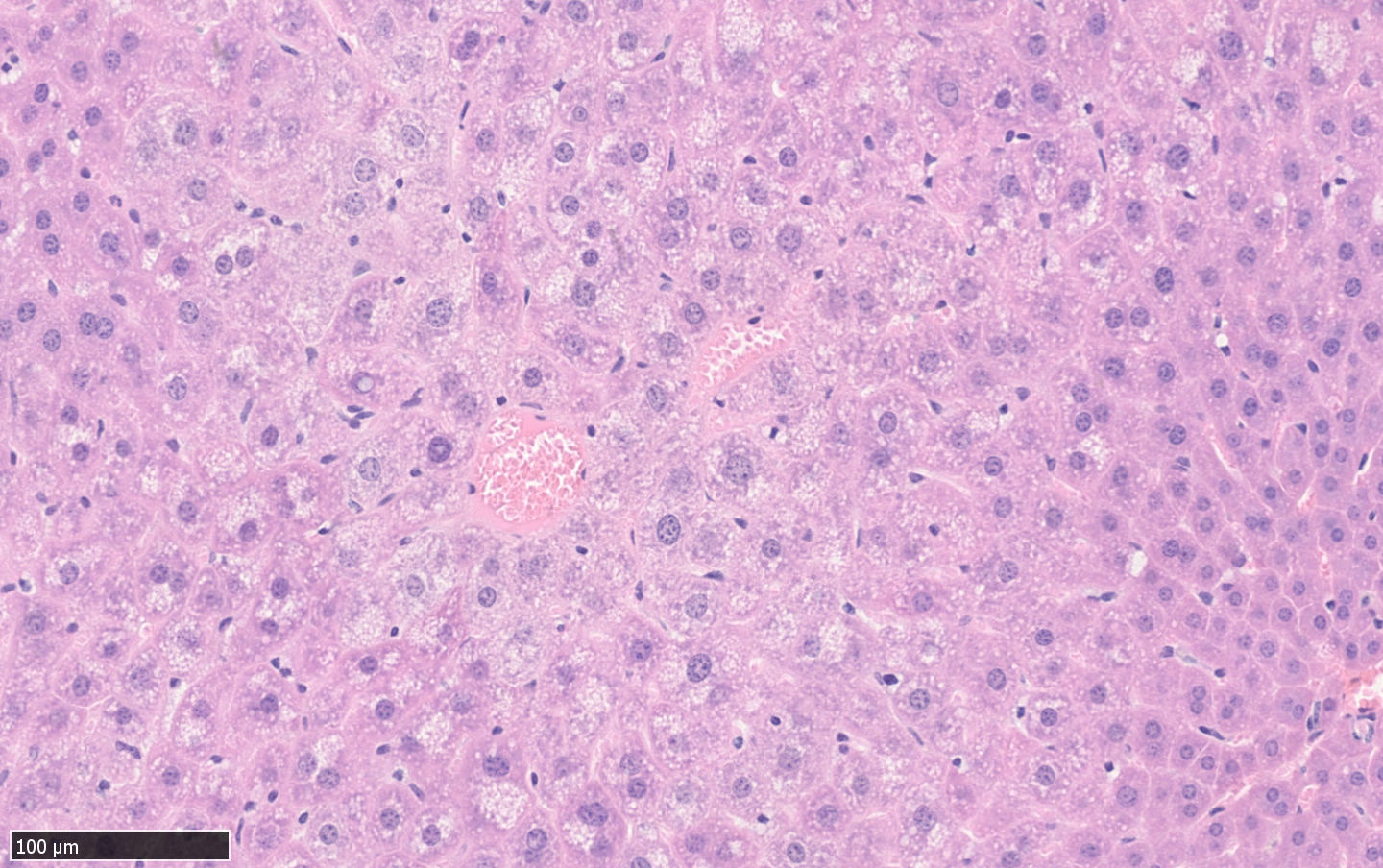

Supplement: Supplementary file 4 [file DataSheet9.ZIP › NASH SCORE-db(1)/db10,11/8.jpg]

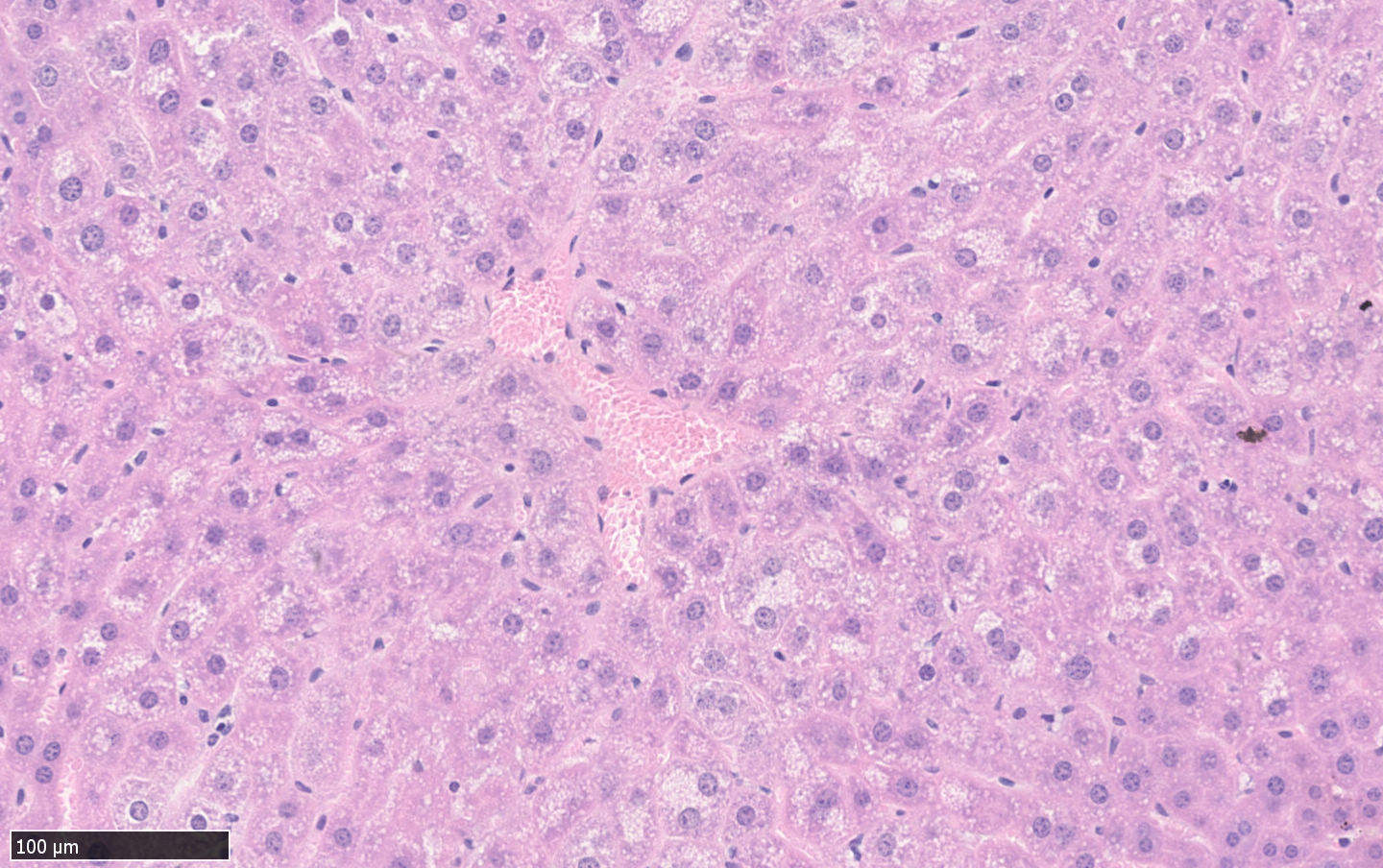

Supplement: Supplementary file 4 [file DataSheet9.ZIP › NASH SCORE-db(1)/db10,11/9.jpg]

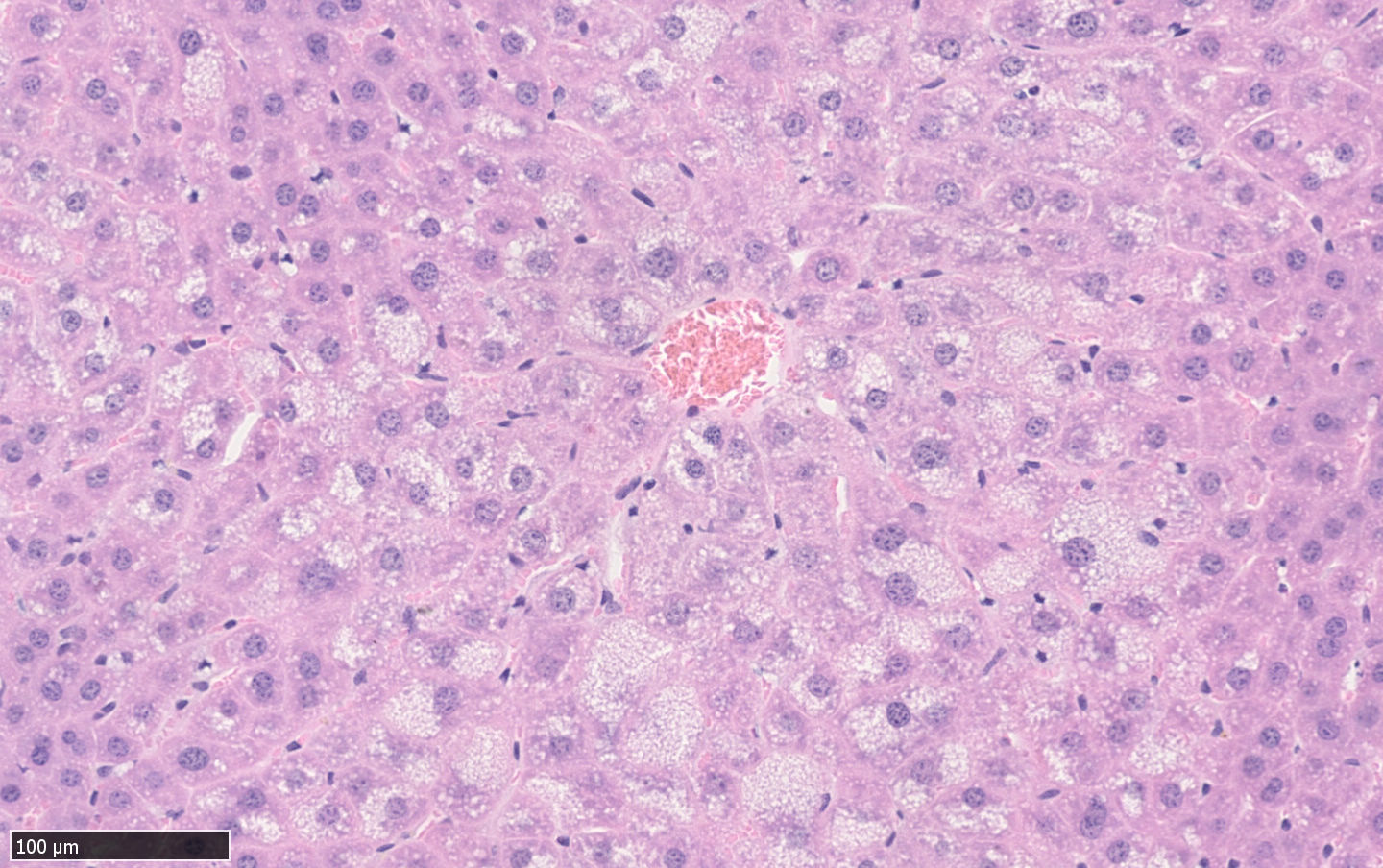

Supplement: Supplementary file 4 [file DataSheet9.ZIP › NASH SCORE-db(1)/db12,14/1.jpg]

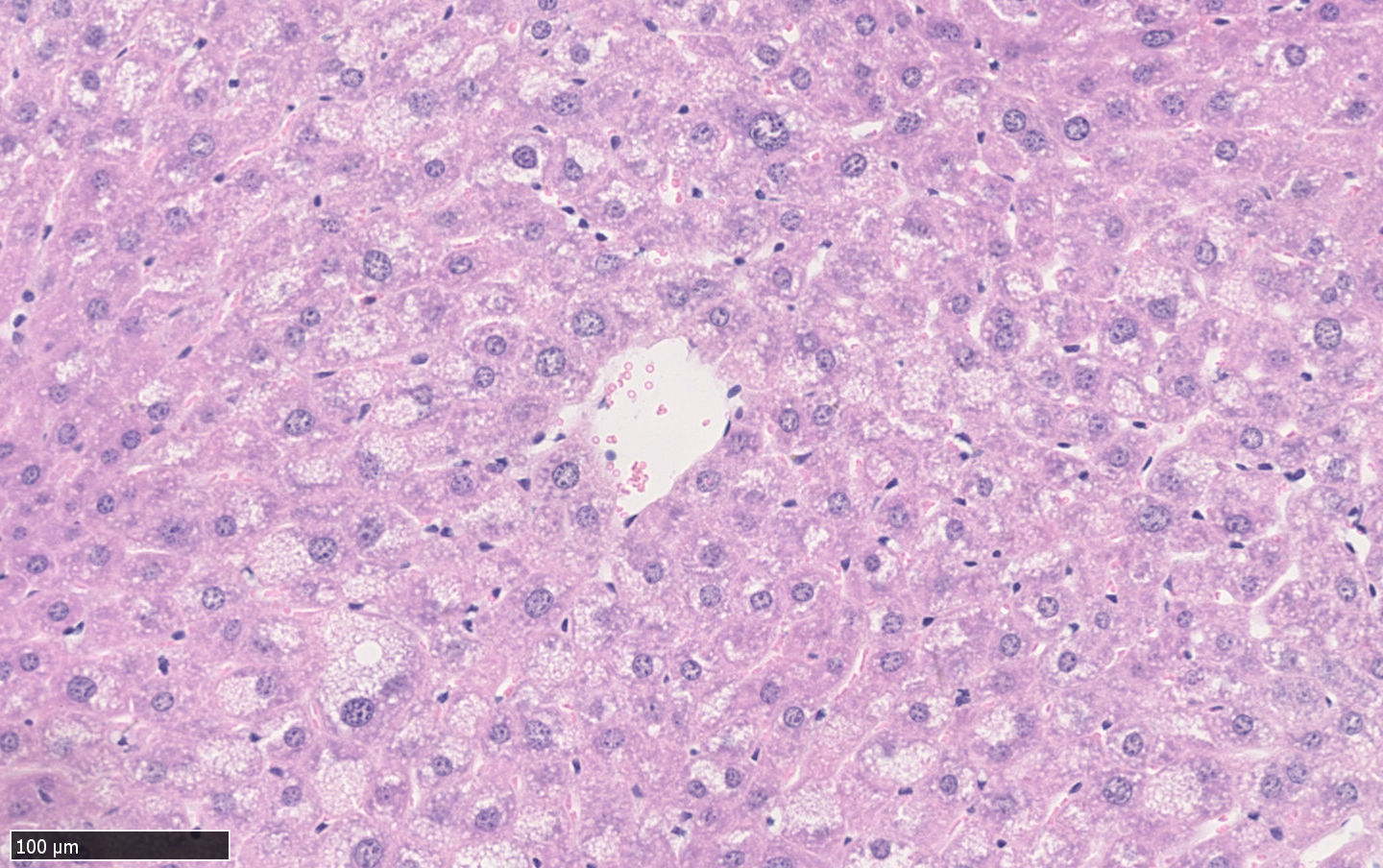

Supplement: Supplementary file 4 [file DataSheet9.ZIP › NASH SCORE-db(1)/db12,14/10.jpg]

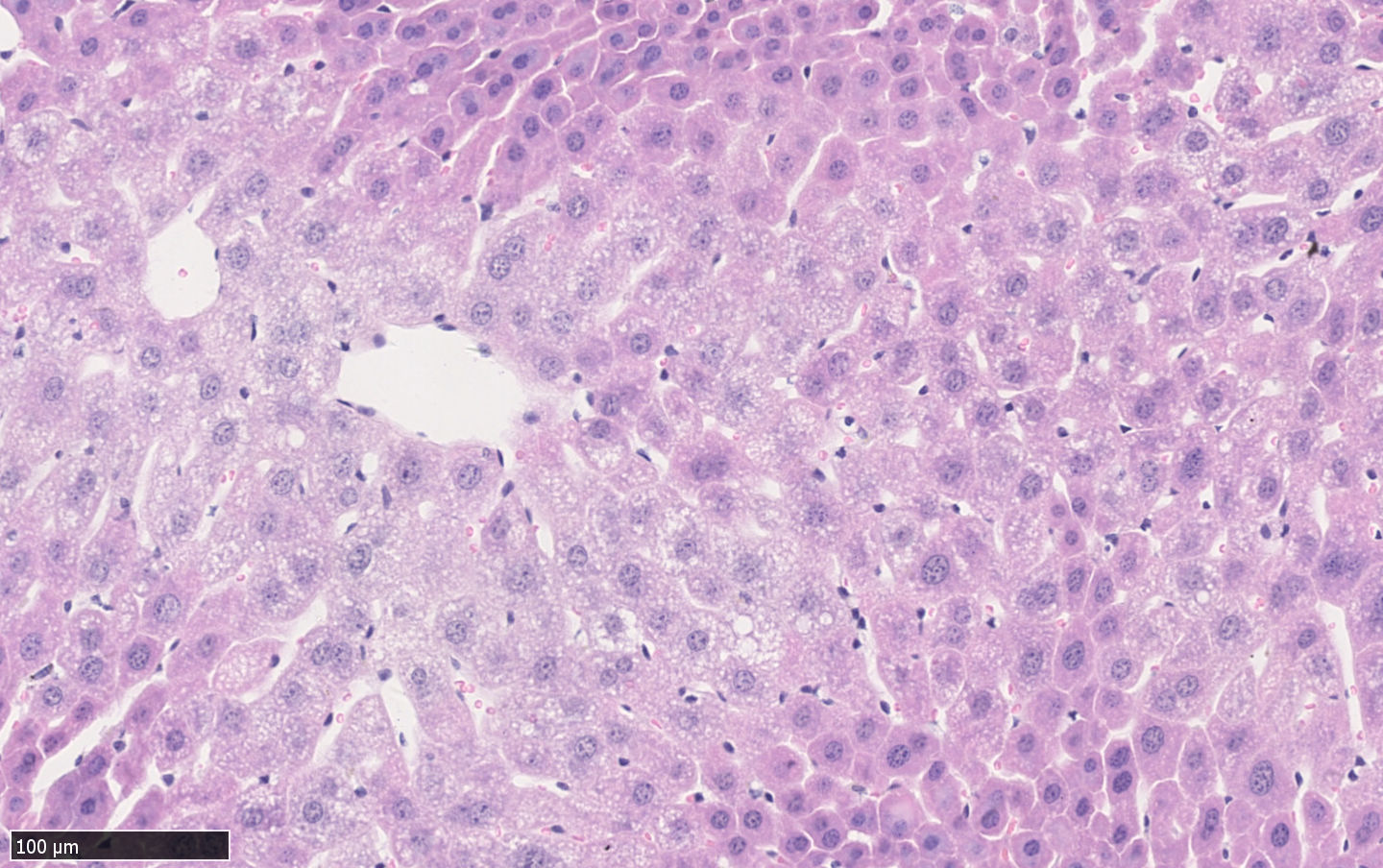

Supplement: Supplementary file 4 [file DataSheet9.ZIP › NASH SCORE-db(1)/db12,14/11.jpg]

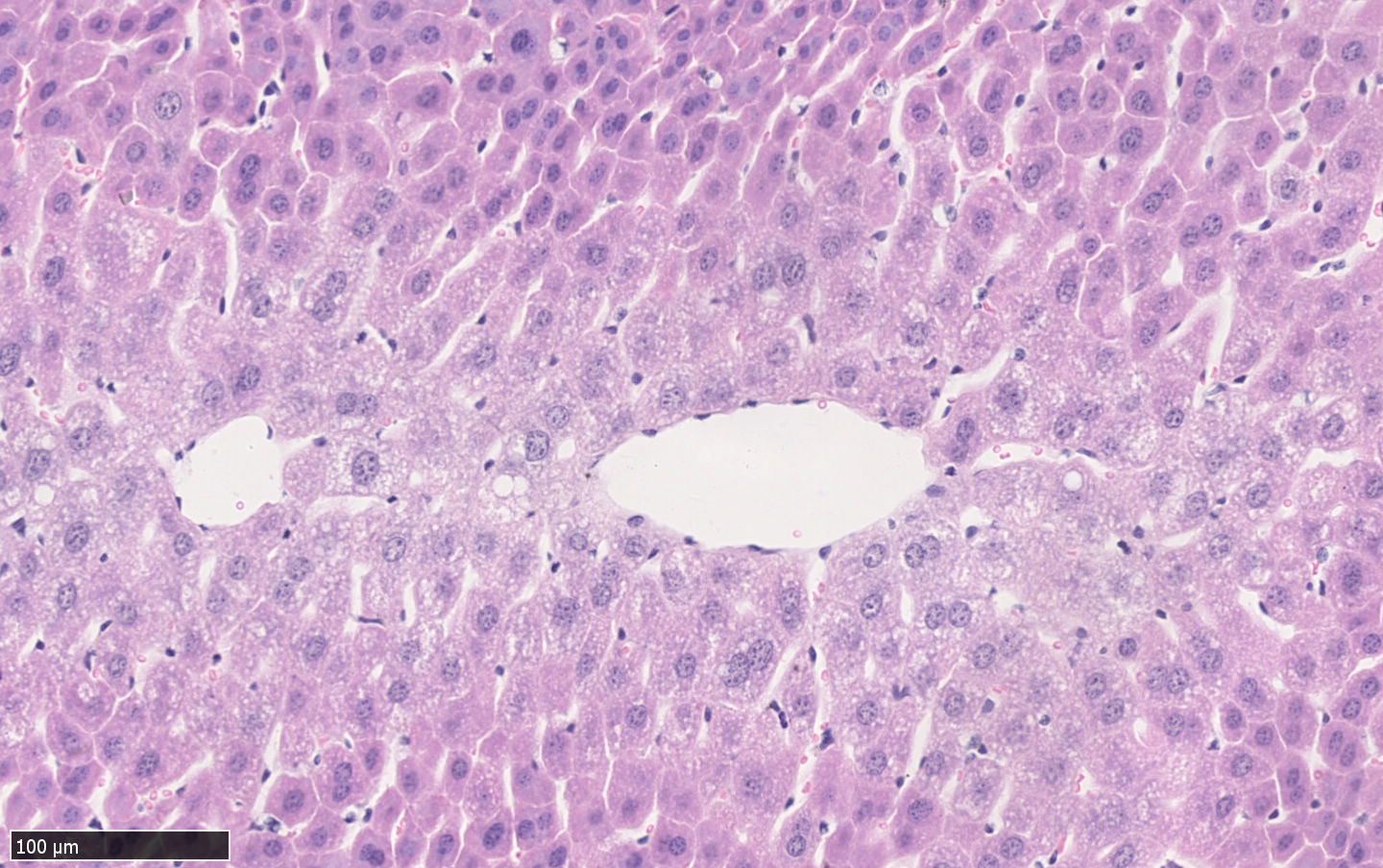

Supplement: Supplementary file 4 [file DataSheet9.ZIP › NASH SCORE-db(1)/db12,14/12.jpg]

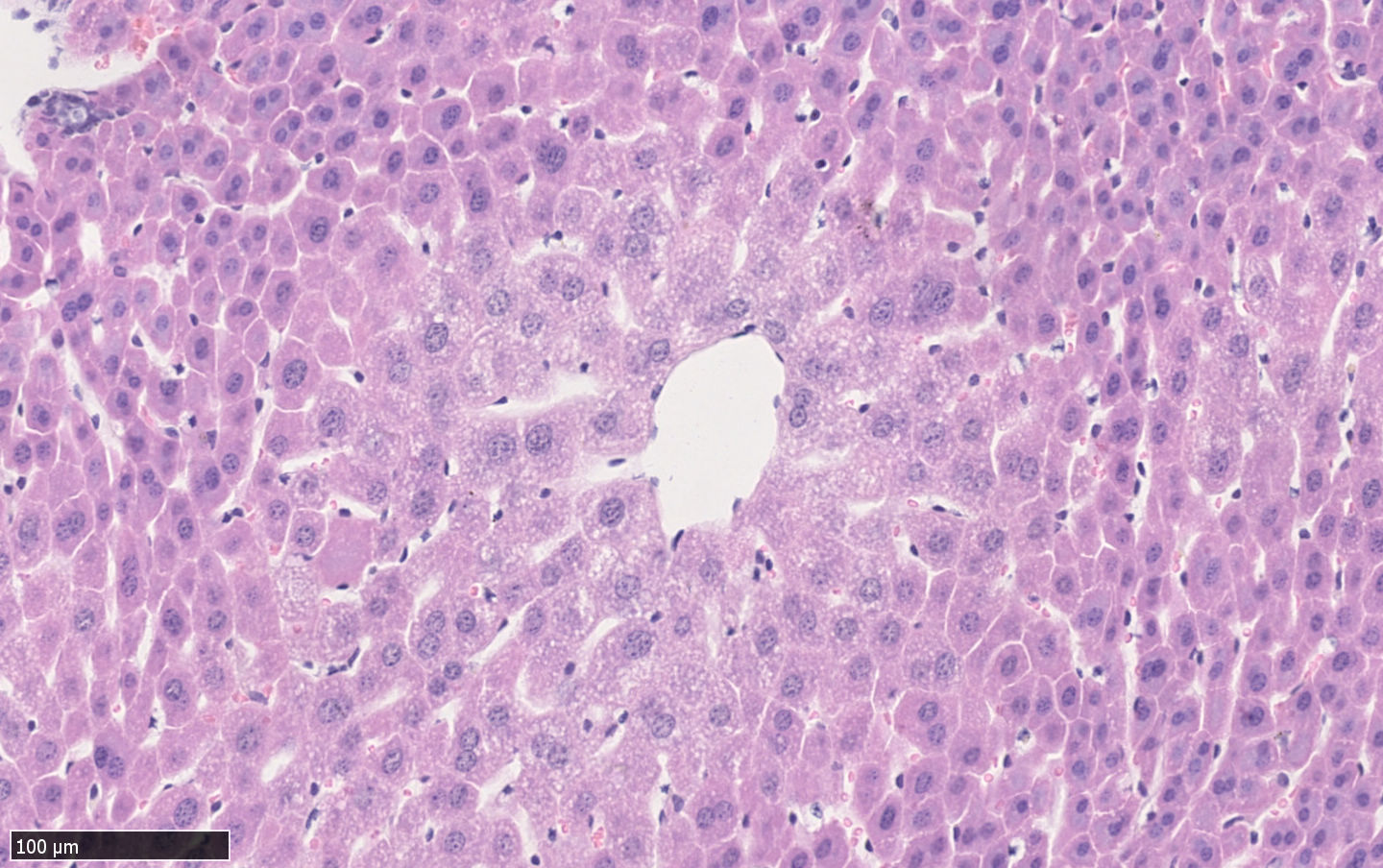

Supplement: Supplementary file 4 [file DataSheet9.ZIP › NASH SCORE-db(1)/db12,14/13.jpg]

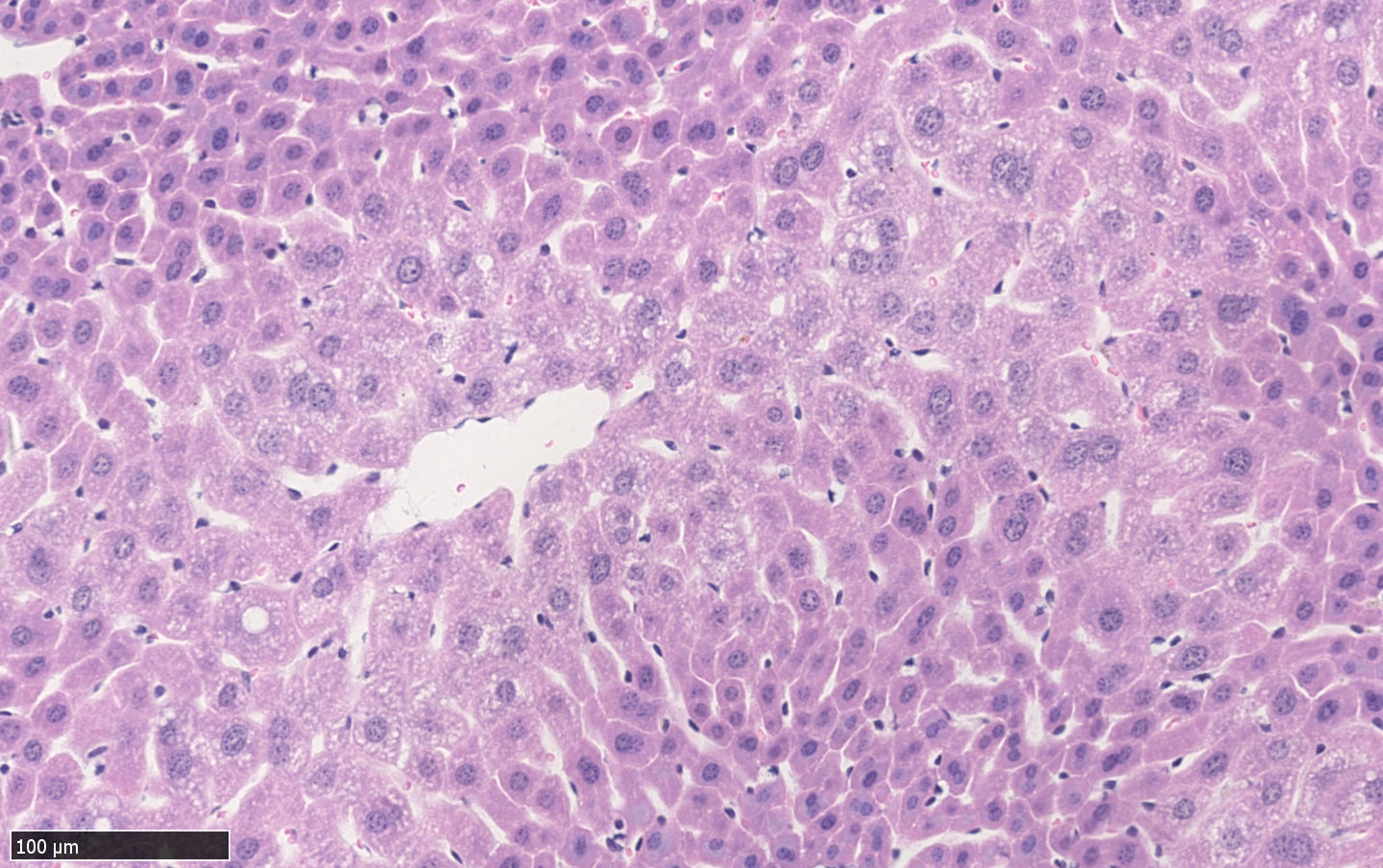

Supplement: Supplementary file 4 [file DataSheet9.ZIP › NASH SCORE-db(1)/db12,14/14.jpg]

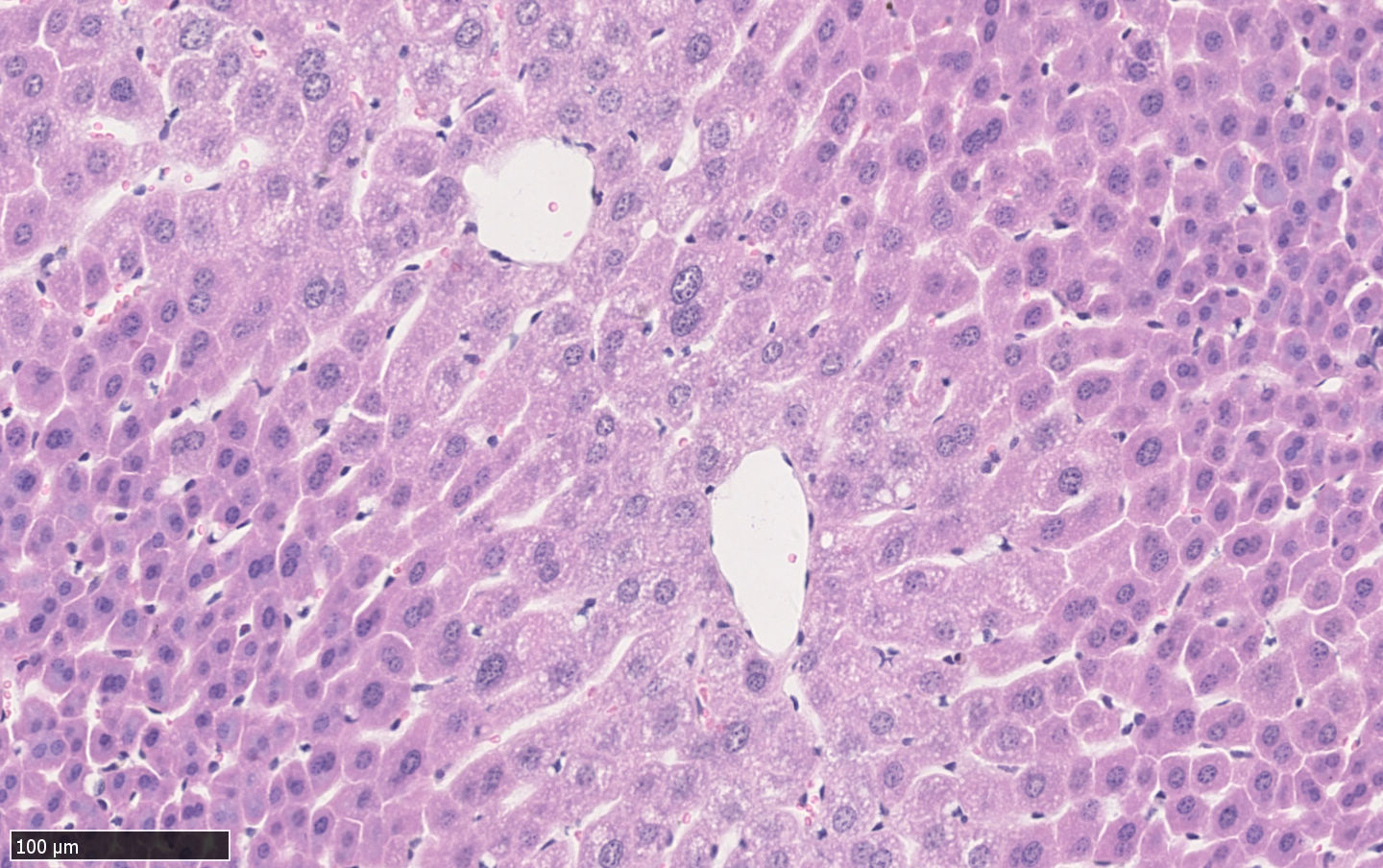

Supplement: Supplementary file 4 [file DataSheet9.ZIP › NASH SCORE-db(1)/db12,14/15.jpg]

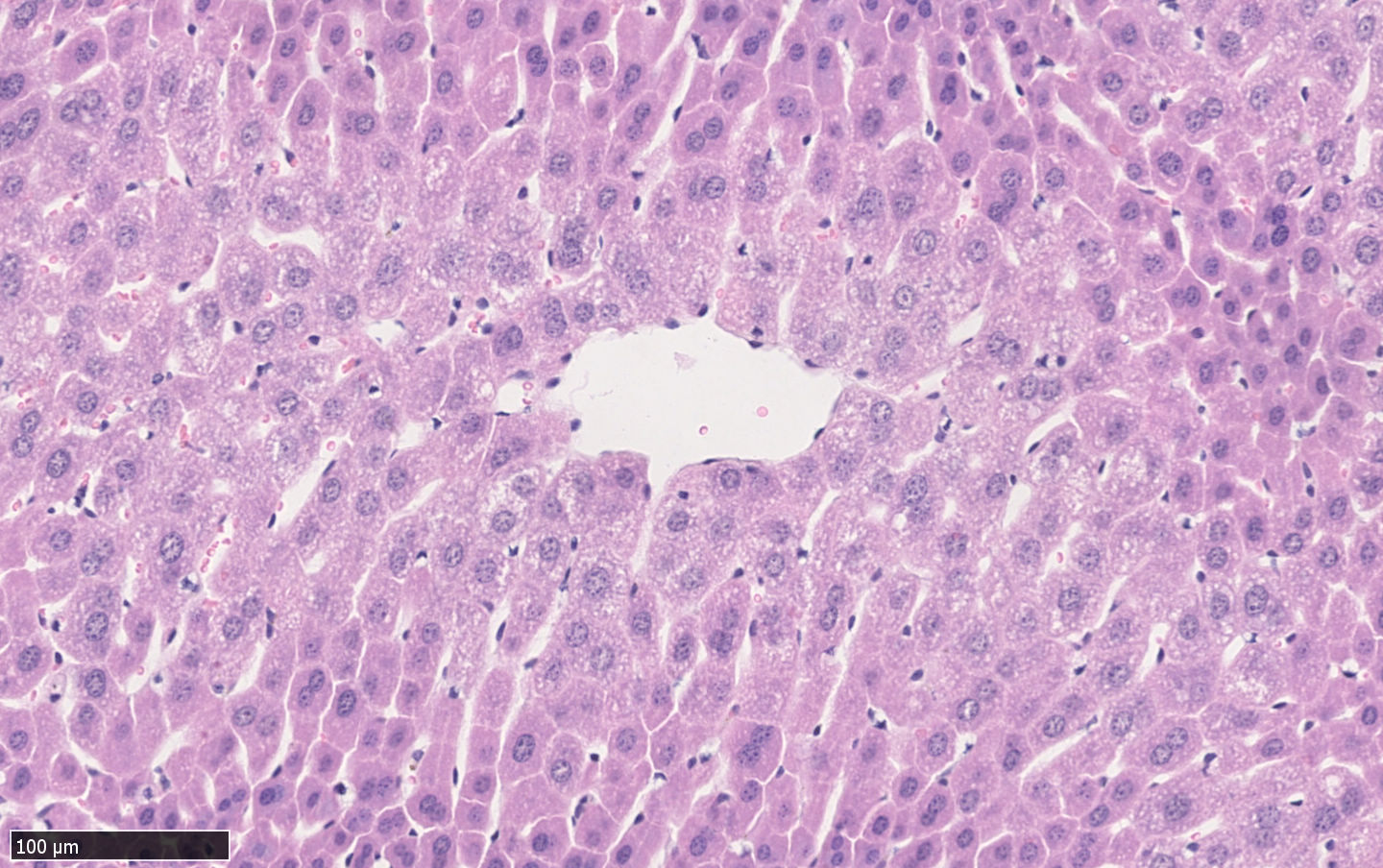

Supplement: Supplementary file 4 [file DataSheet9.ZIP › NASH SCORE-db(1)/db12,14/16.jpg]

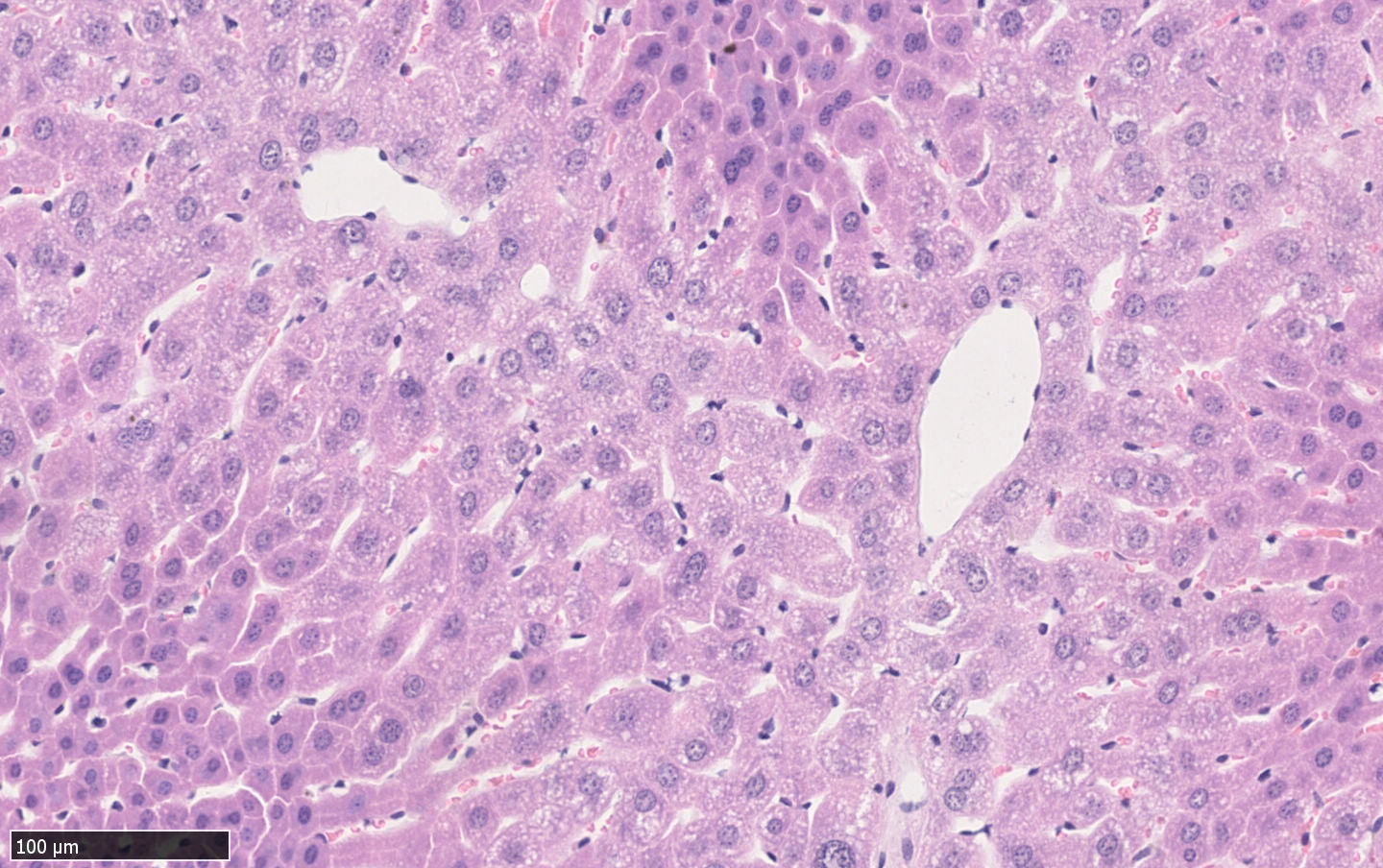

Supplement: Supplementary file 4 [file DataSheet9.ZIP › NASH SCORE-db(1)/db12,14/17.jpg]

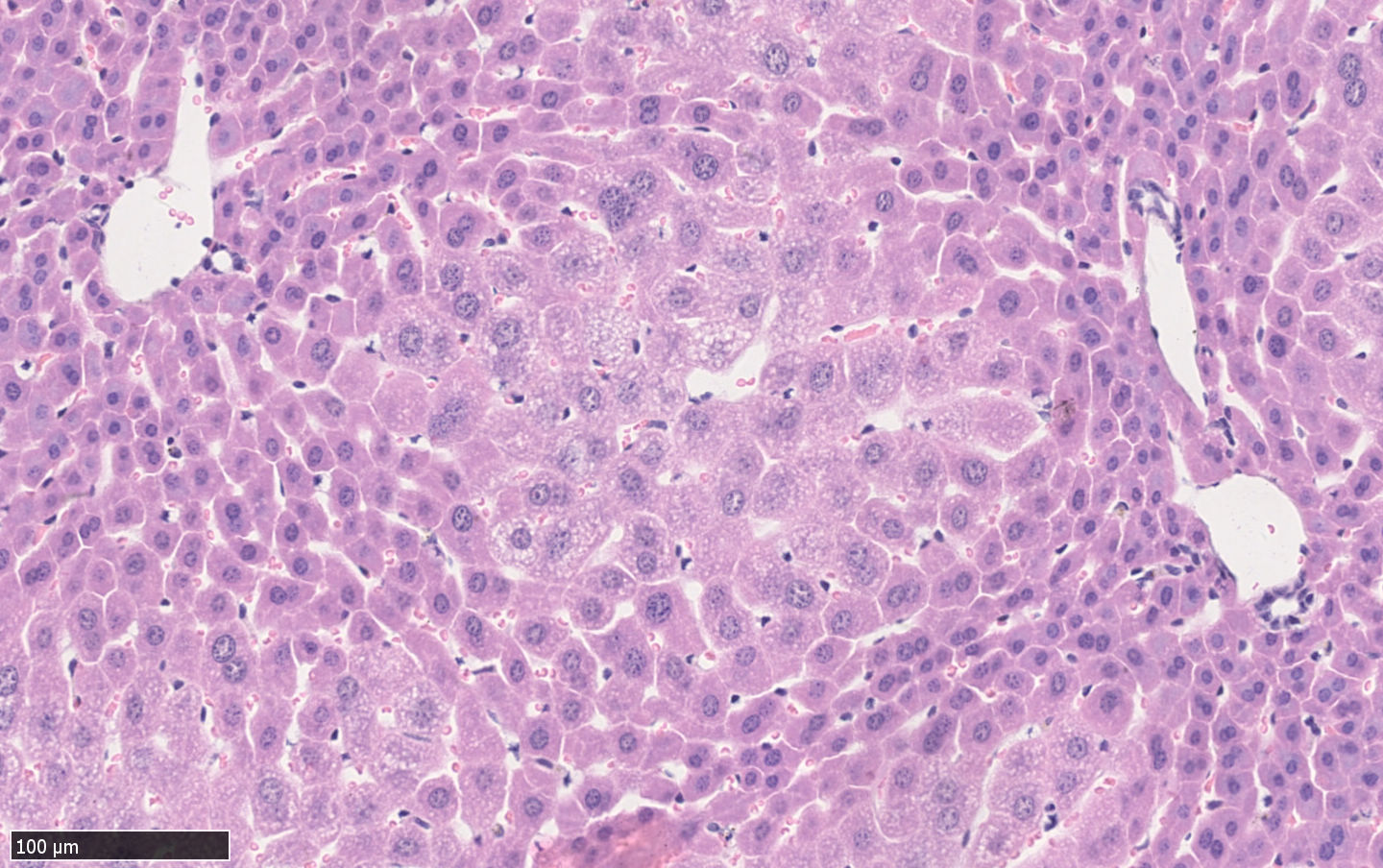

Supplement: Supplementary file 4 [file DataSheet9.ZIP › NASH SCORE-db(1)/db12,14/18.jpg]

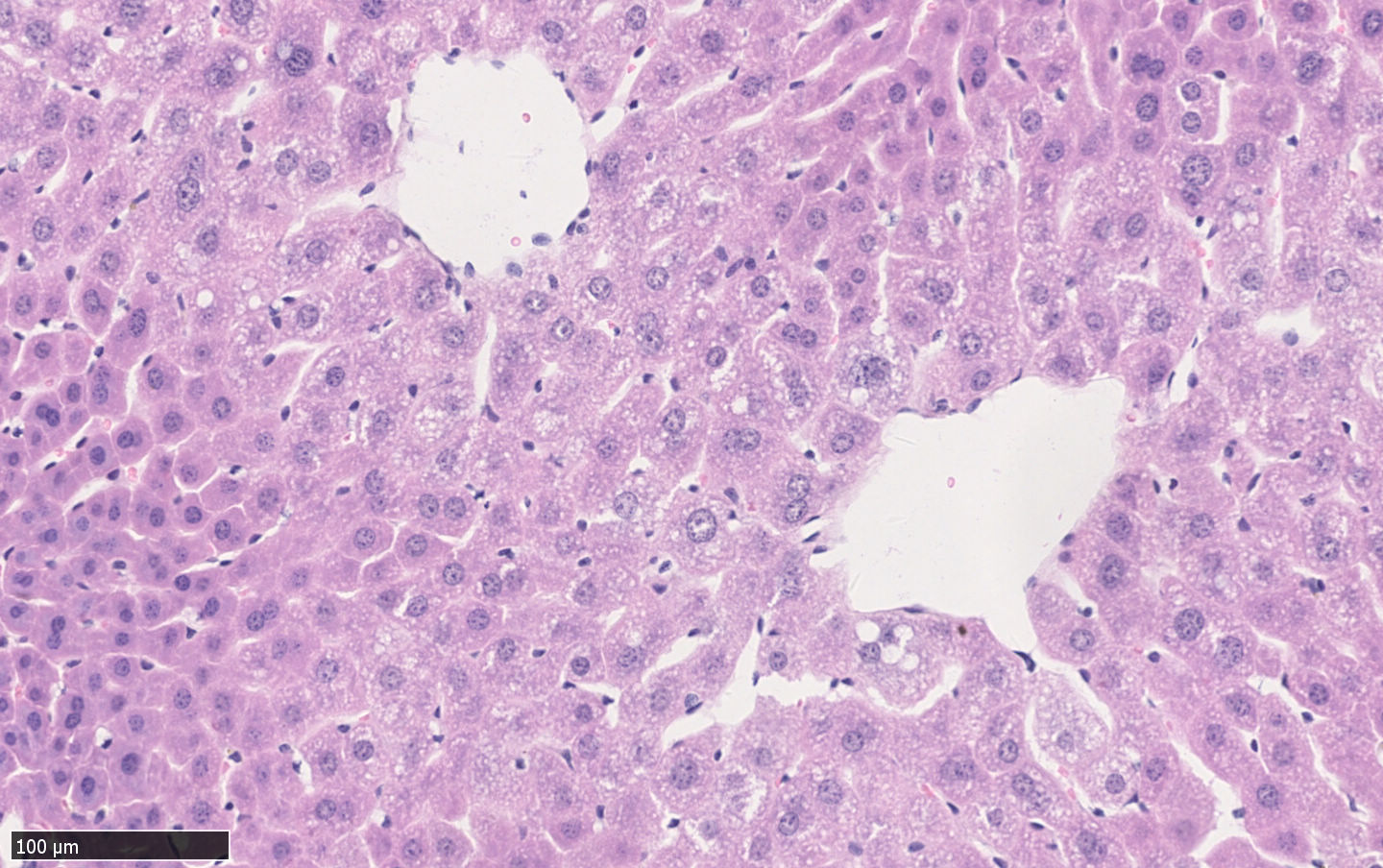

Supplement: Supplementary file 4 [file DataSheet9.ZIP › NASH SCORE-db(1)/db12,14/19.jpg]

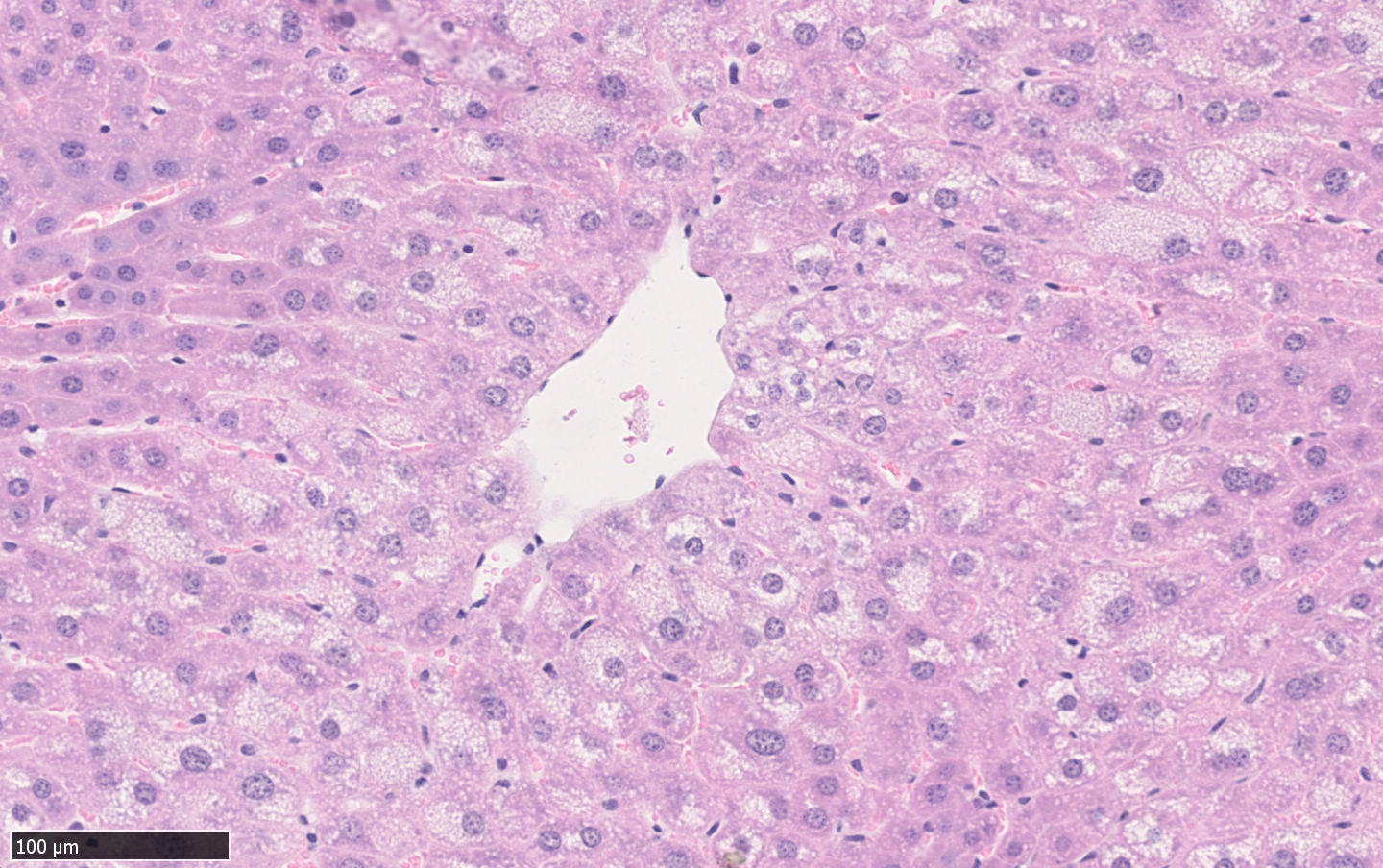

Supplement: Supplementary file 4 [file DataSheet9.ZIP › NASH SCORE-db(1)/db12,14/2.jpg]

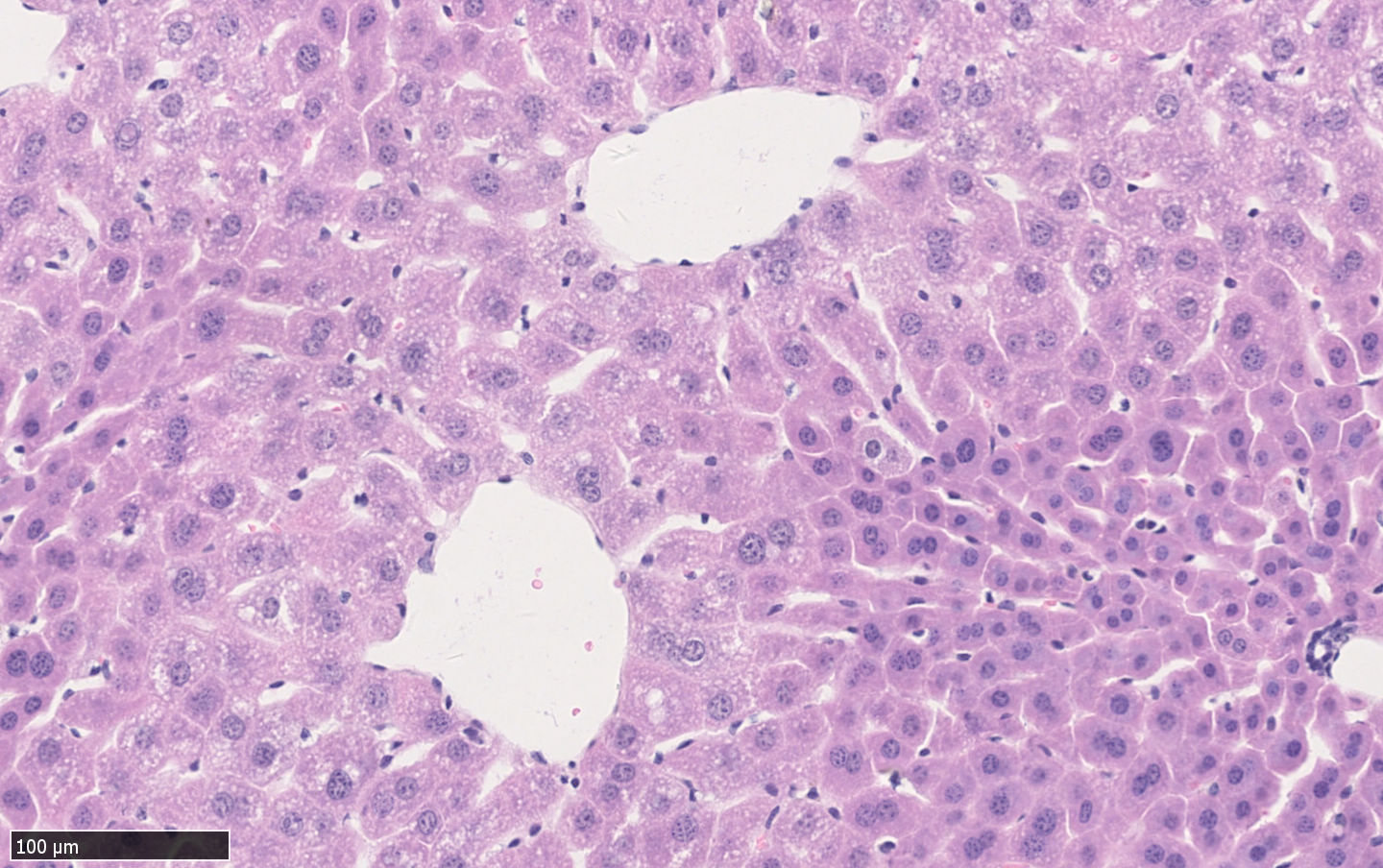

Supplement: Supplementary file 4 [file DataSheet9.ZIP › NASH SCORE-db(1)/db12,14/20.jpg]

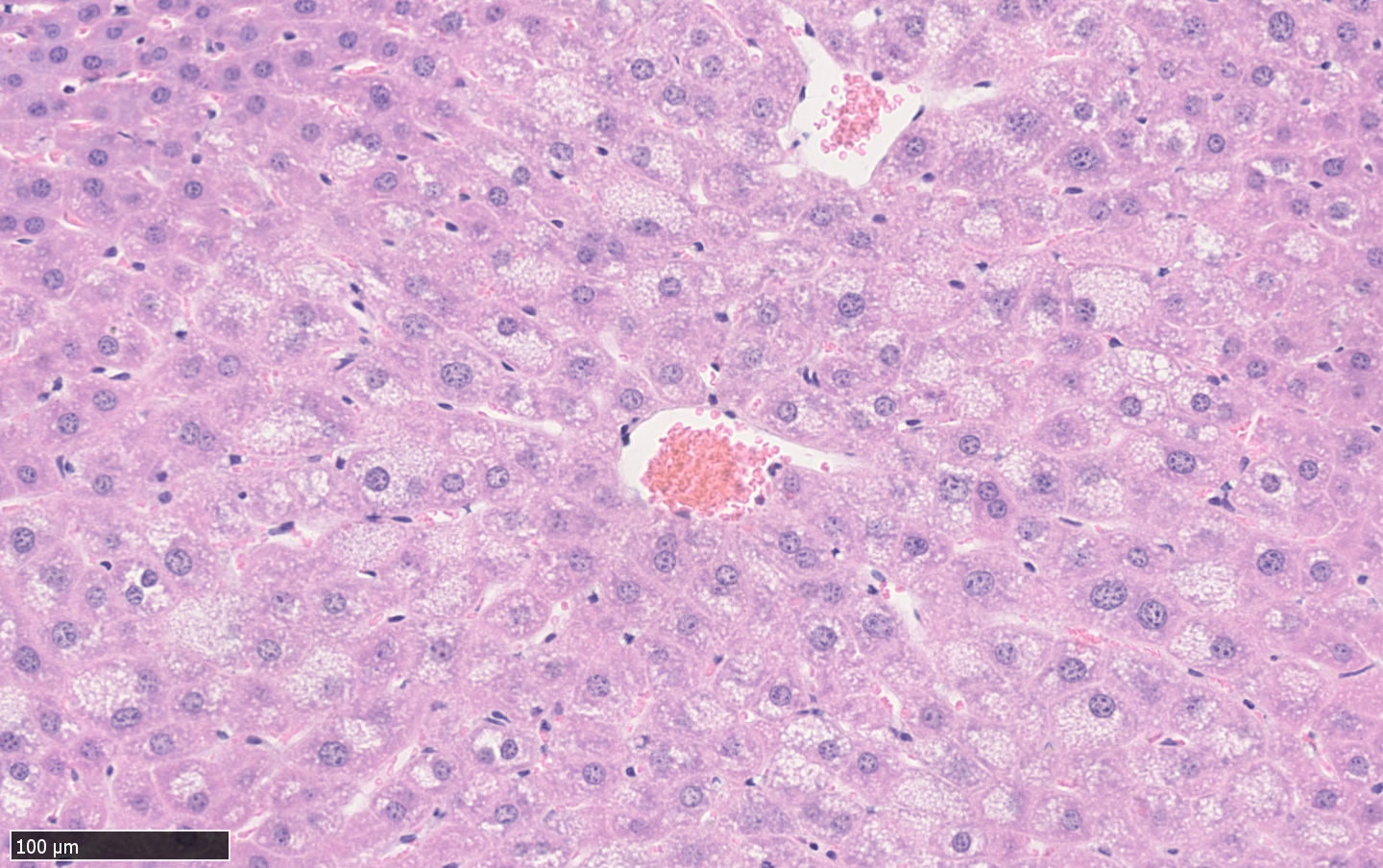

Supplement: Supplementary file 4 [file DataSheet9.ZIP › NASH SCORE-db(1)/db12,14/3.jpg]

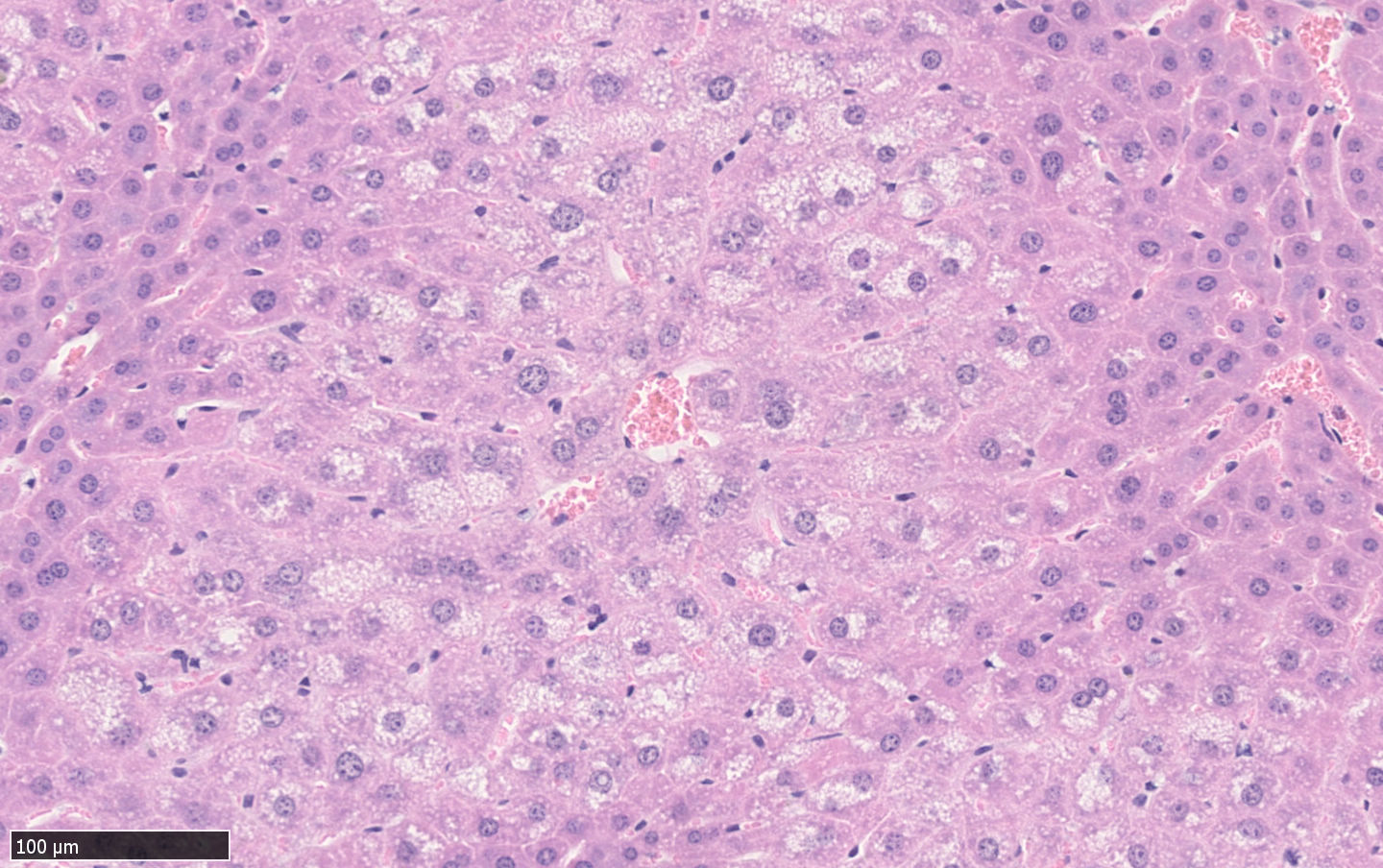

Supplement: Supplementary file 4 [file DataSheet9.ZIP › NASH SCORE-db(1)/db12,14/4.jpg]

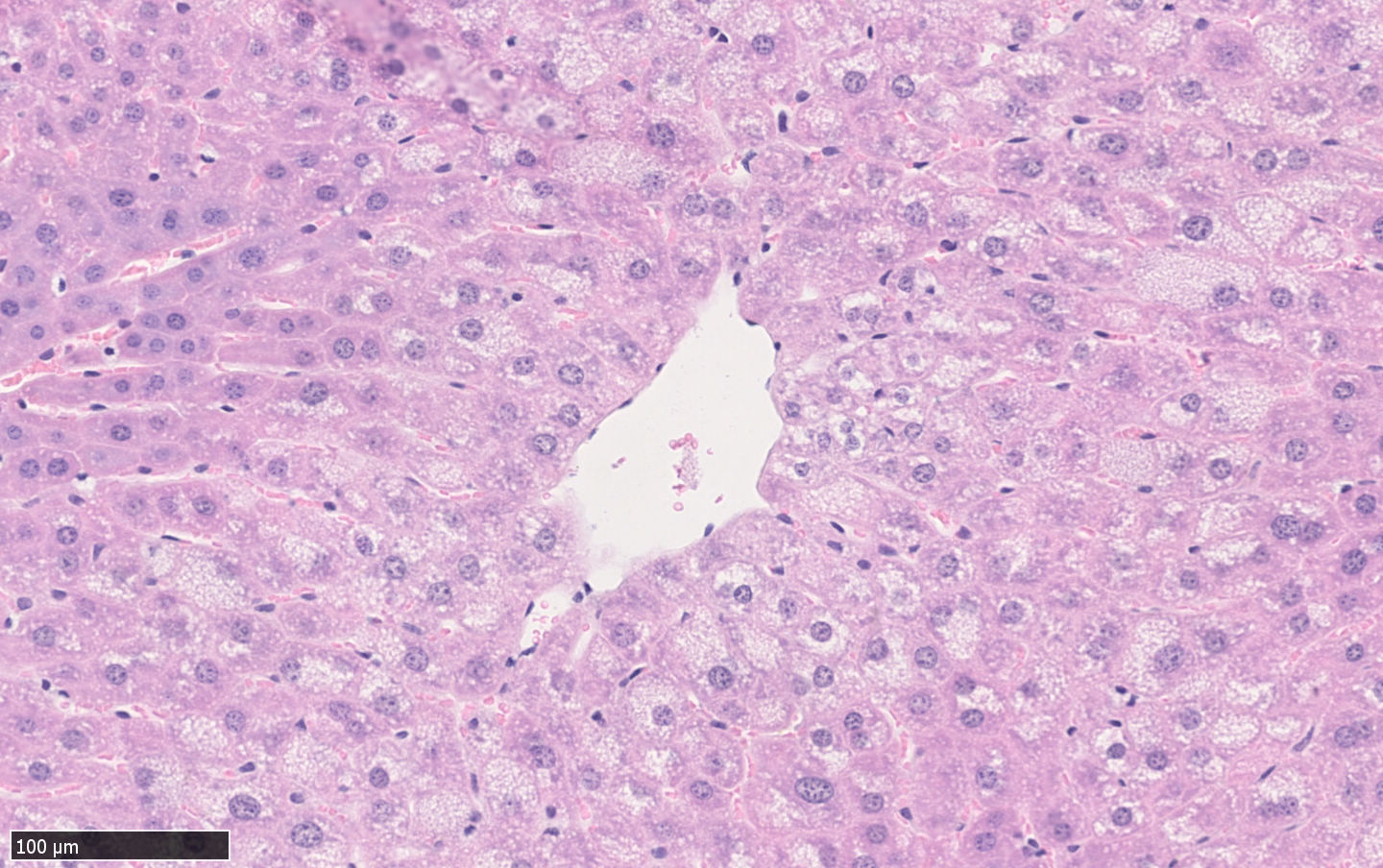

Supplement: Supplementary file 4 [file DataSheet9.ZIP › NASH SCORE-db(1)/db12,14/5.jpg]

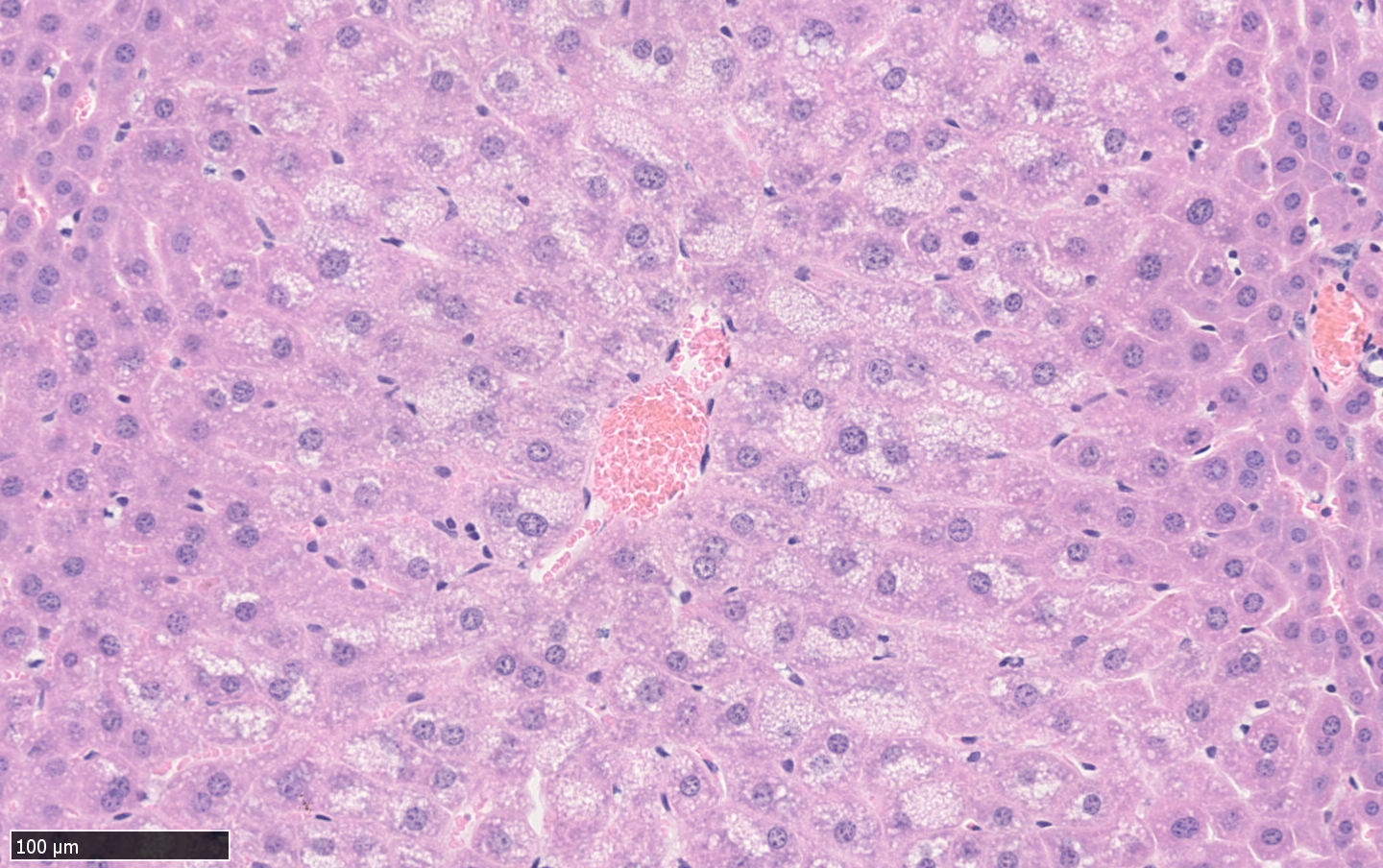

Supplement: Supplementary file 4 [file DataSheet9.ZIP › NASH SCORE-db(1)/db12,14/6.jpg]

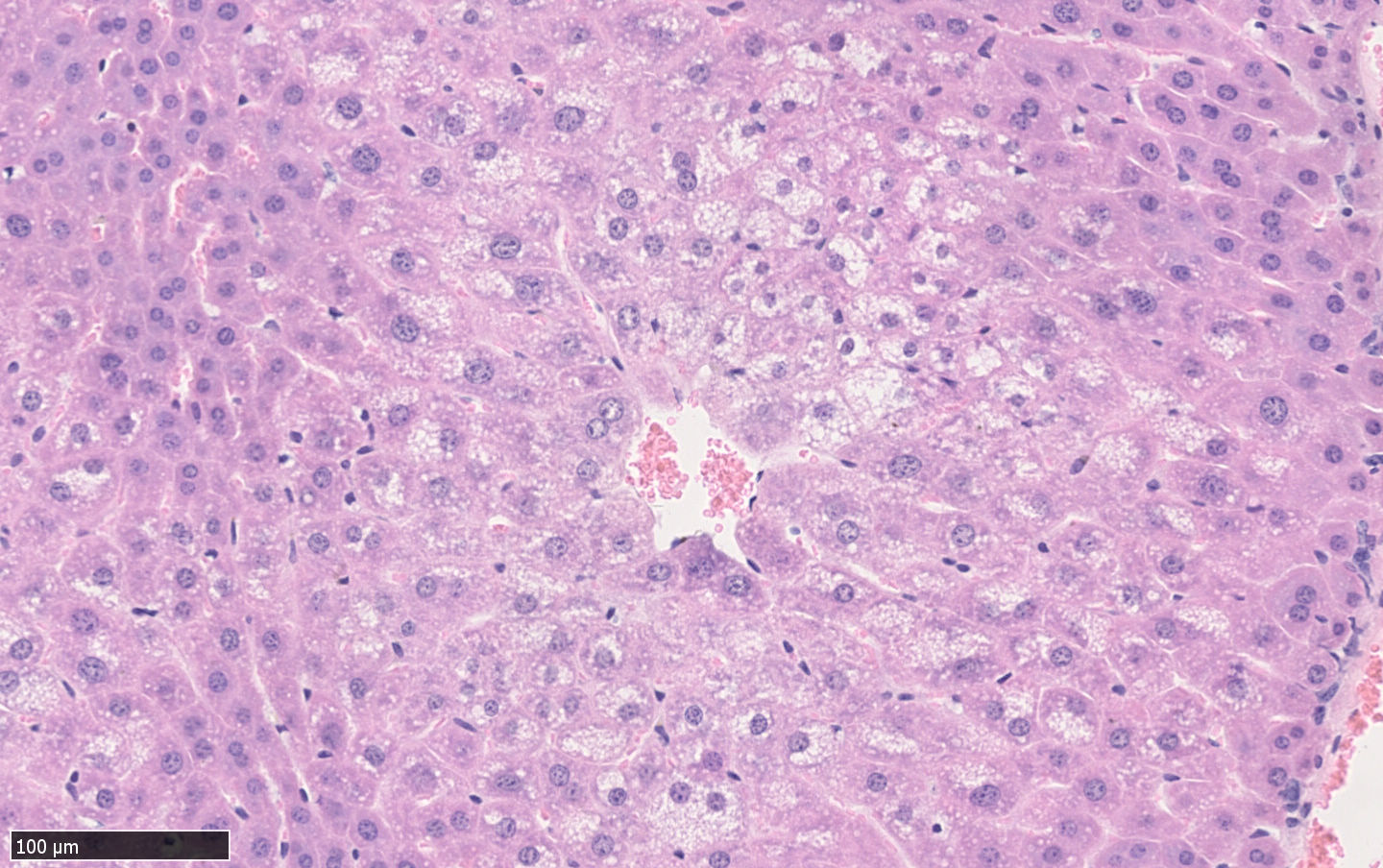

Supplement: Supplementary file 4 [file DataSheet9.ZIP › NASH SCORE-db(1)/db12,14/7.jpg]

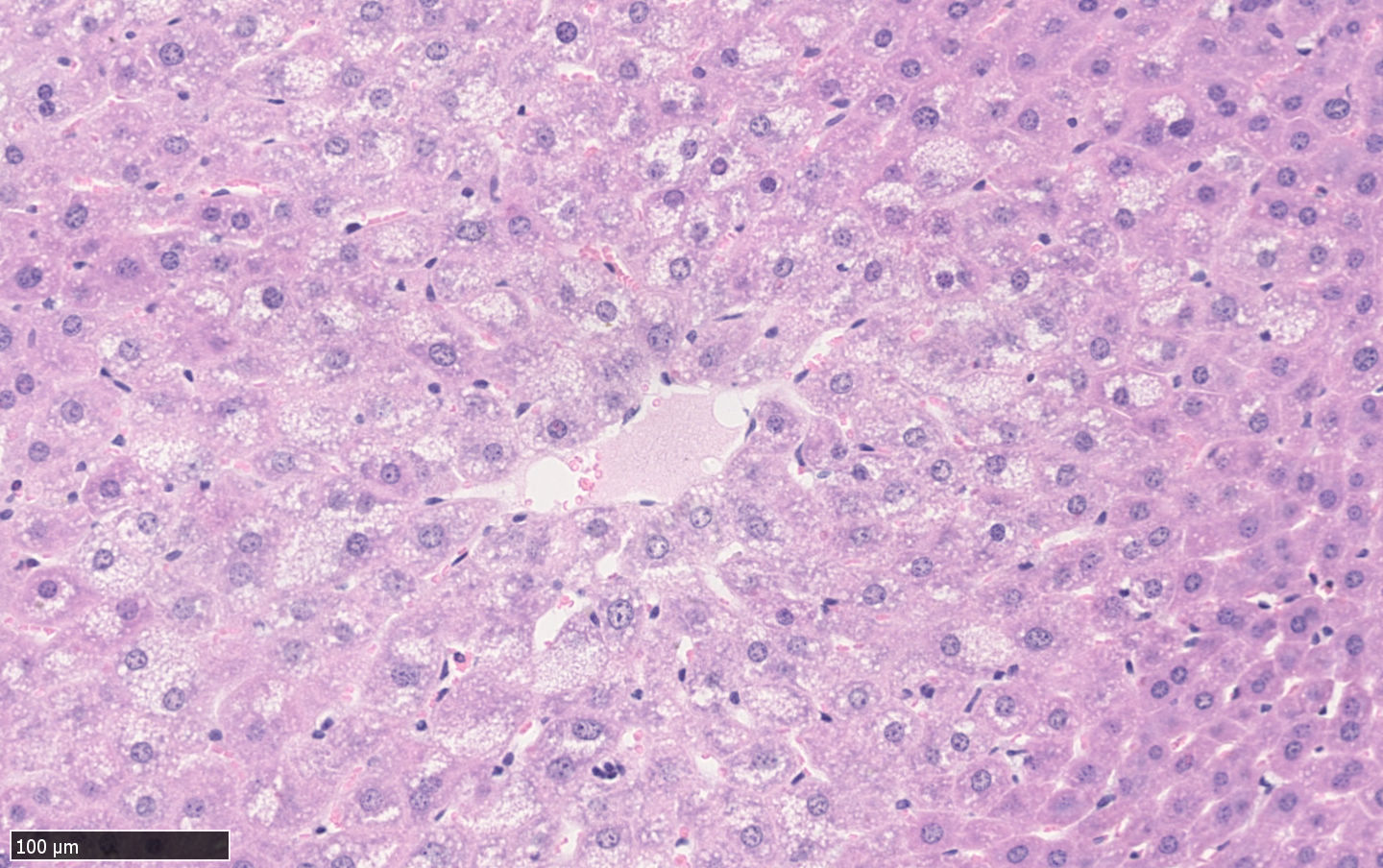

Supplement: Supplementary file 4 [file DataSheet9.ZIP › NASH SCORE-db(1)/db12,14/8.jpg]

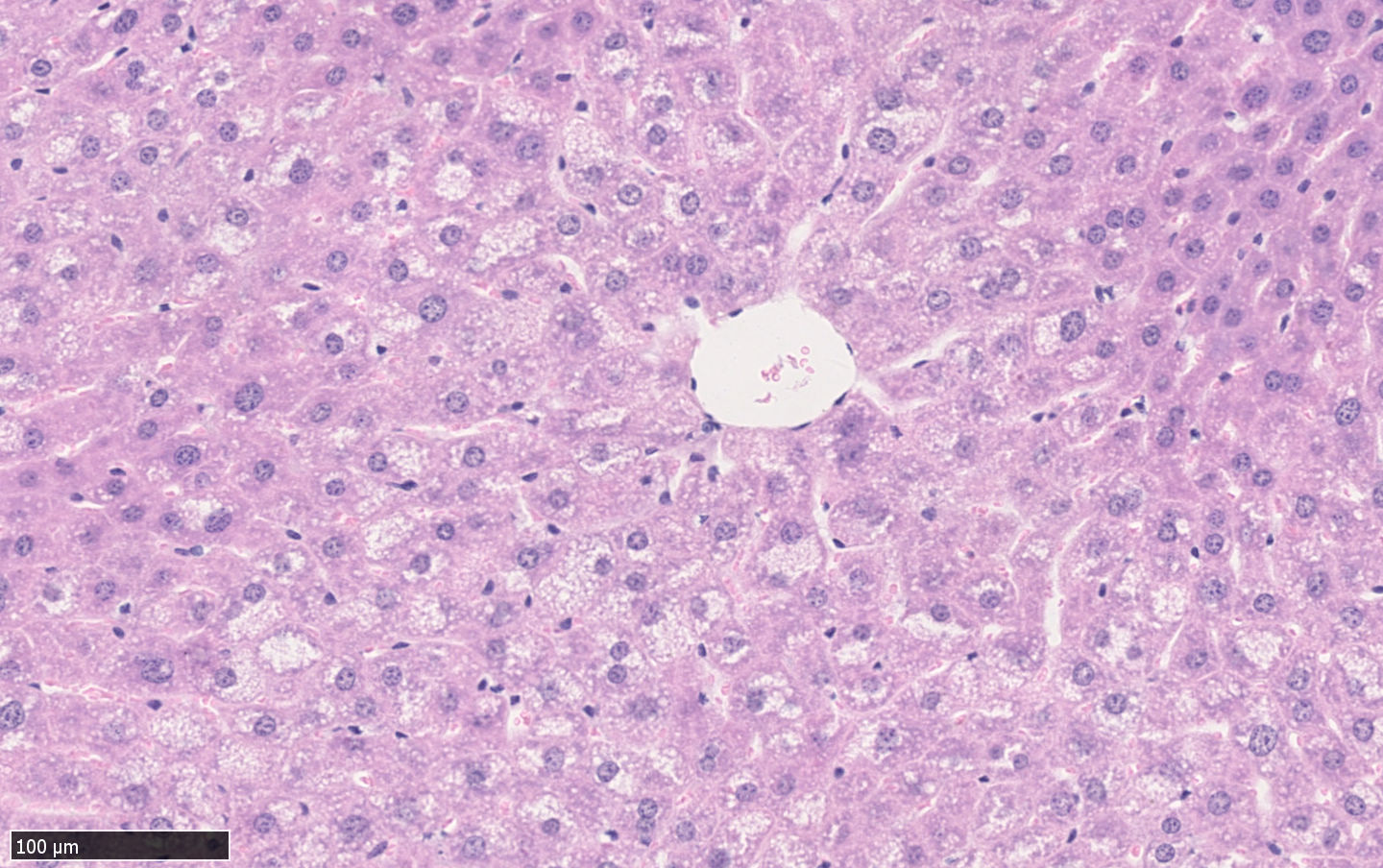

Supplement: Supplementary file 4 [file DataSheet9.ZIP › NASH SCORE-db(1)/db12,14/9.jpg]

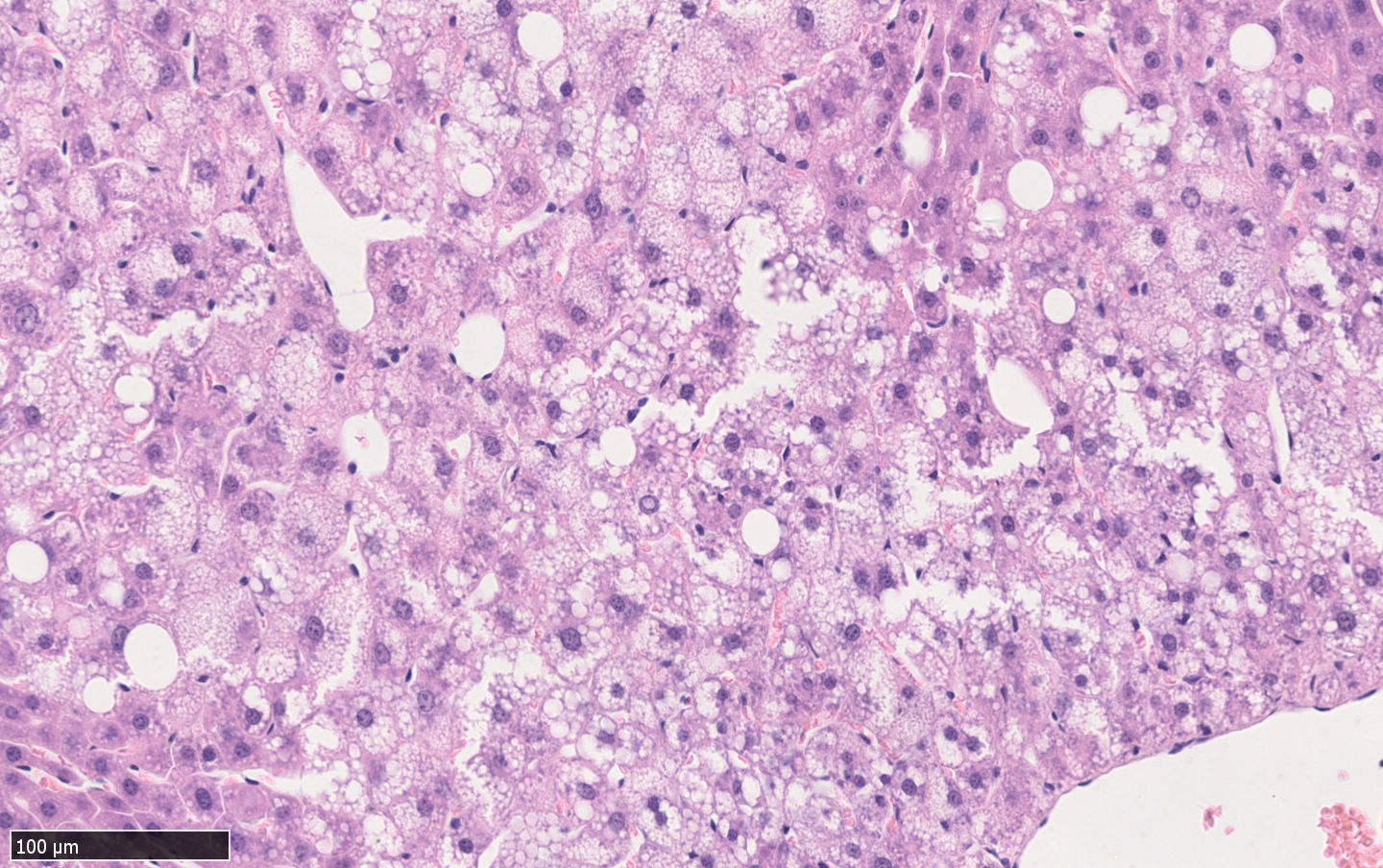

Supplement: Supplementary file 4 [file DataSheet9.ZIP › NASH SCORE-db(1)/db17,18/1.jpg]

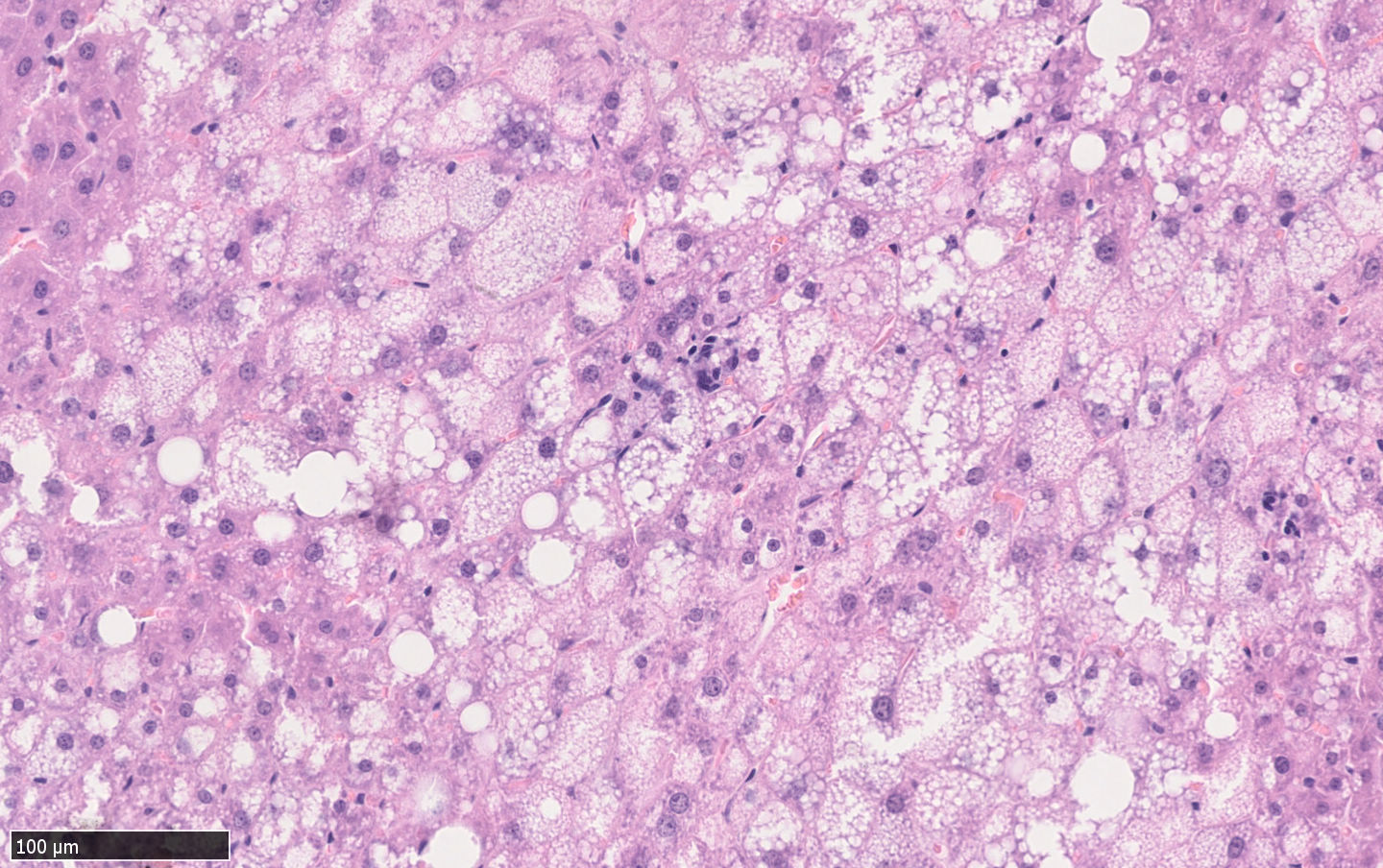

Supplement: Supplementary file 4 [file DataSheet9.ZIP › NASH SCORE-db(1)/db17,18/10.jpg]

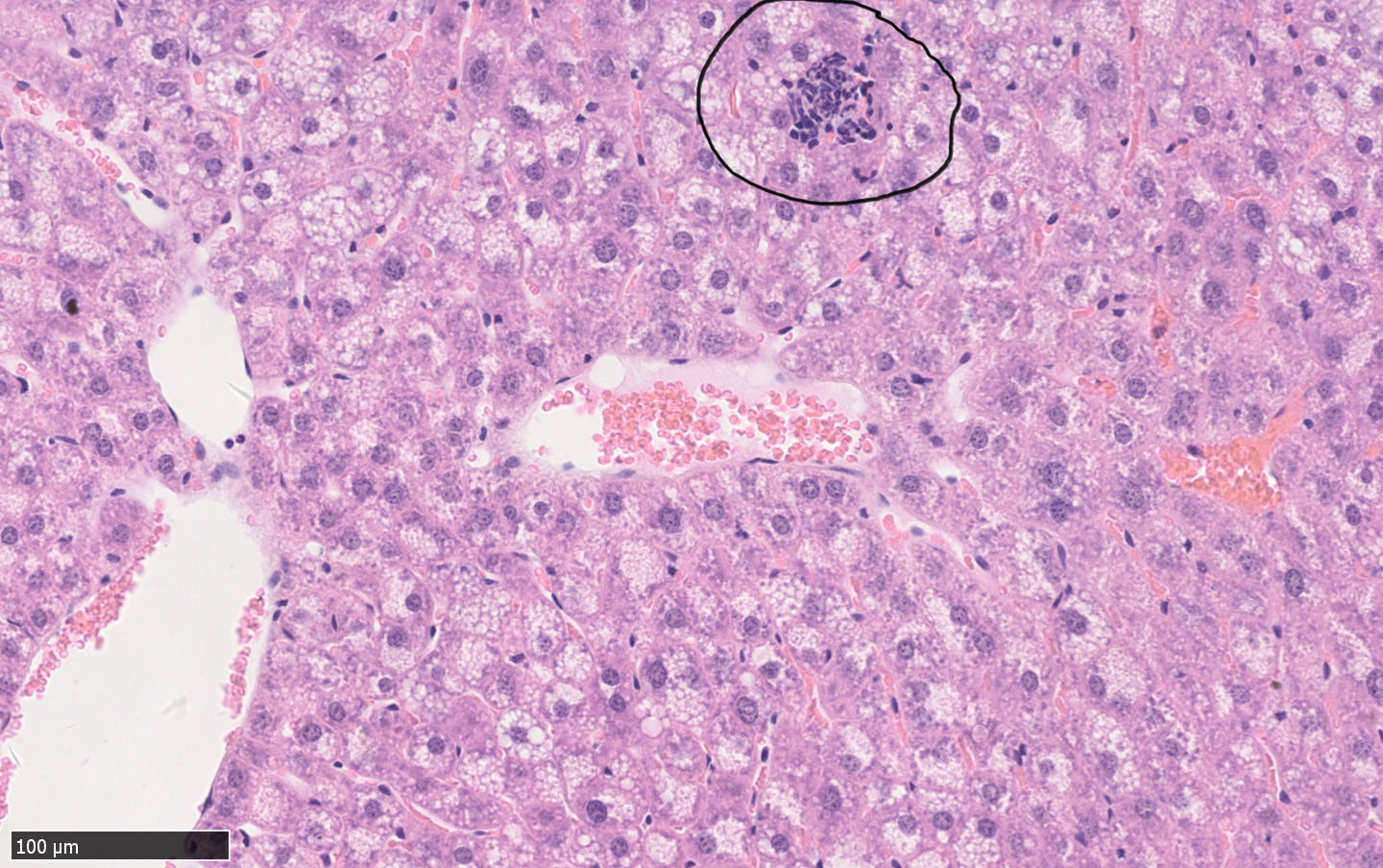

Supplement: Supplementary file 4 [file DataSheet9.ZIP › NASH SCORE-db(1)/db17,18/11.jpg]

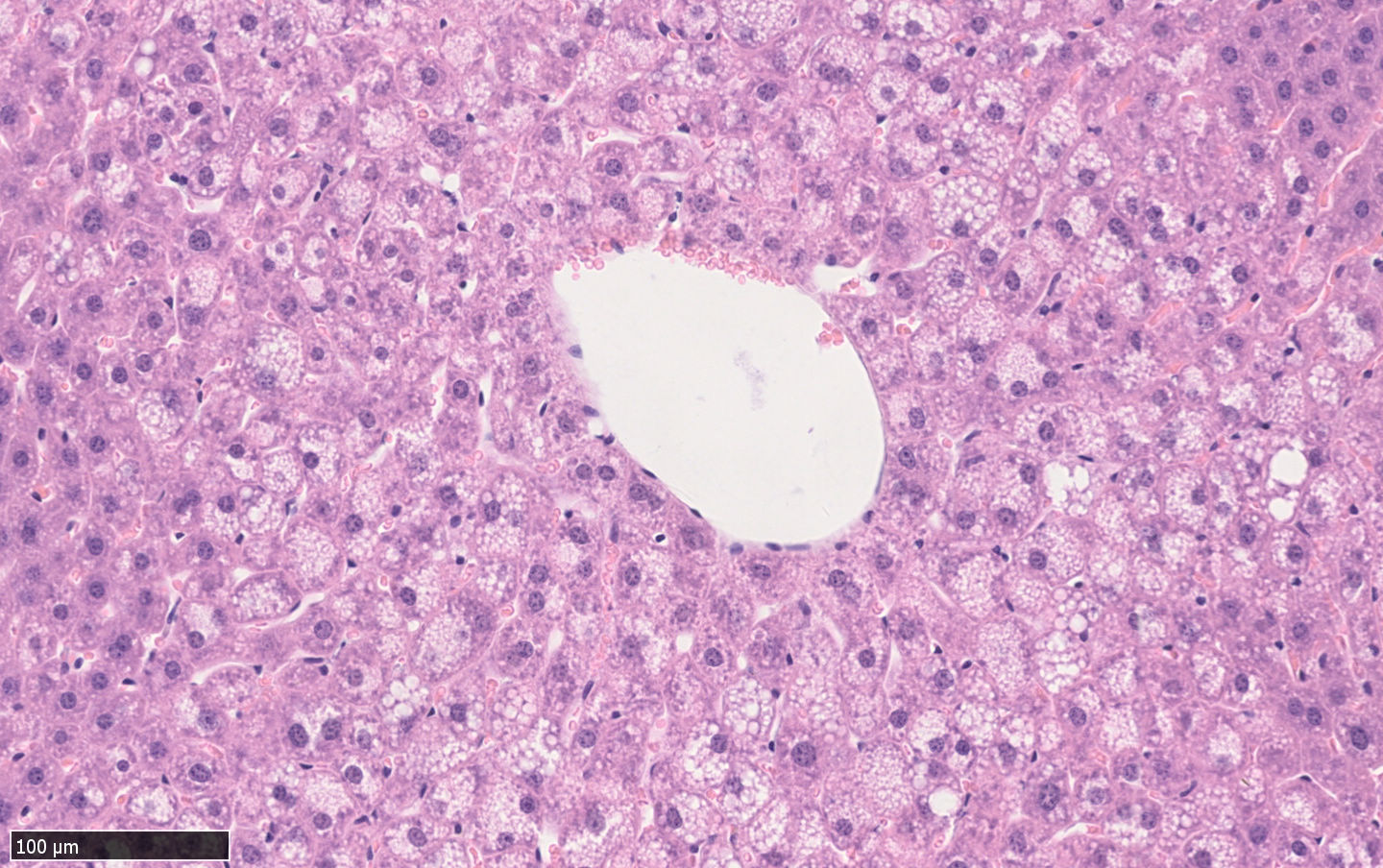

Supplement: Supplementary file 4 [file DataSheet9.ZIP › NASH SCORE-db(1)/db17,18/12.jpg]

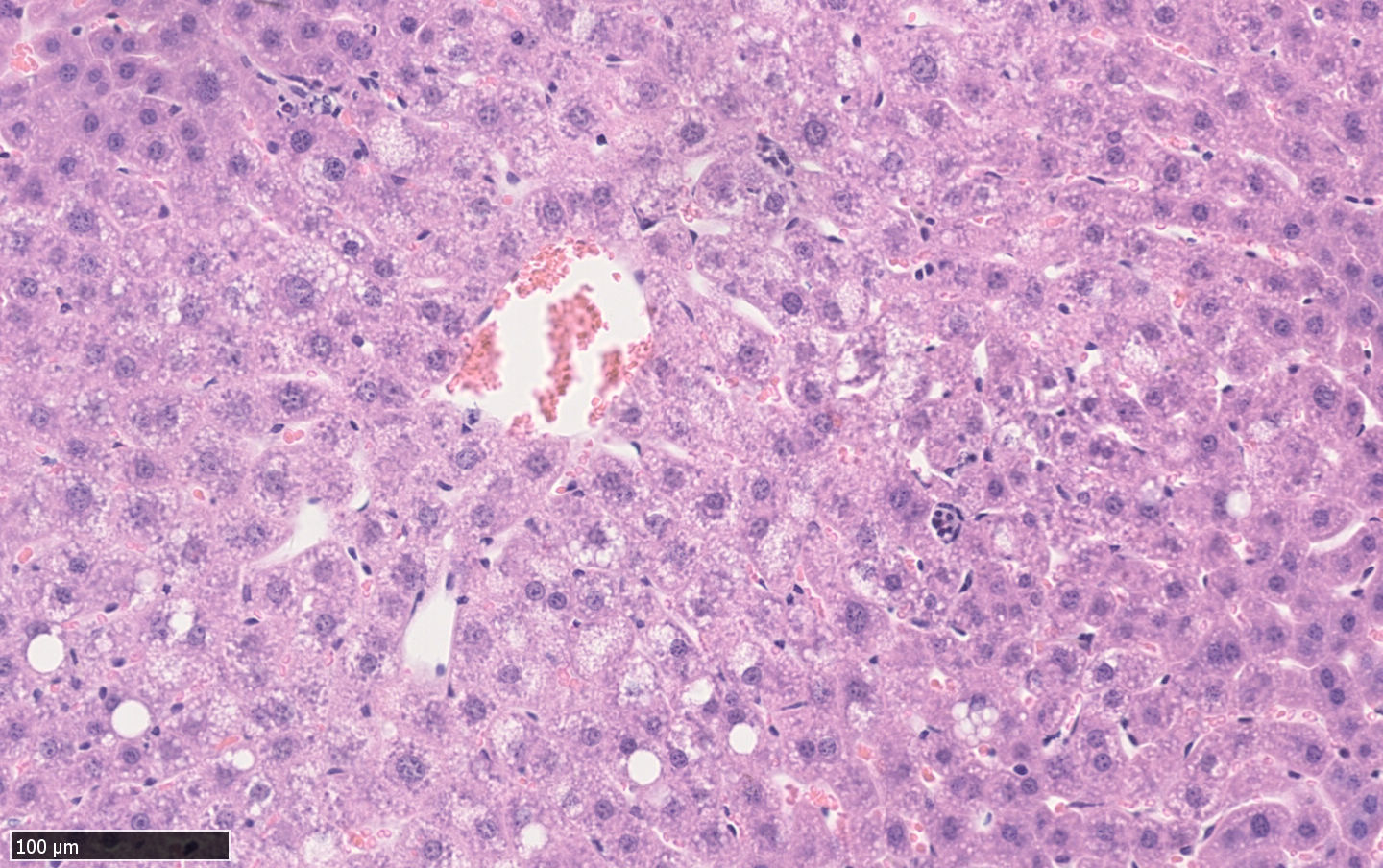

Supplement: Supplementary file 4 [file DataSheet9.ZIP › NASH SCORE-db(1)/db17,18/13.jpg]

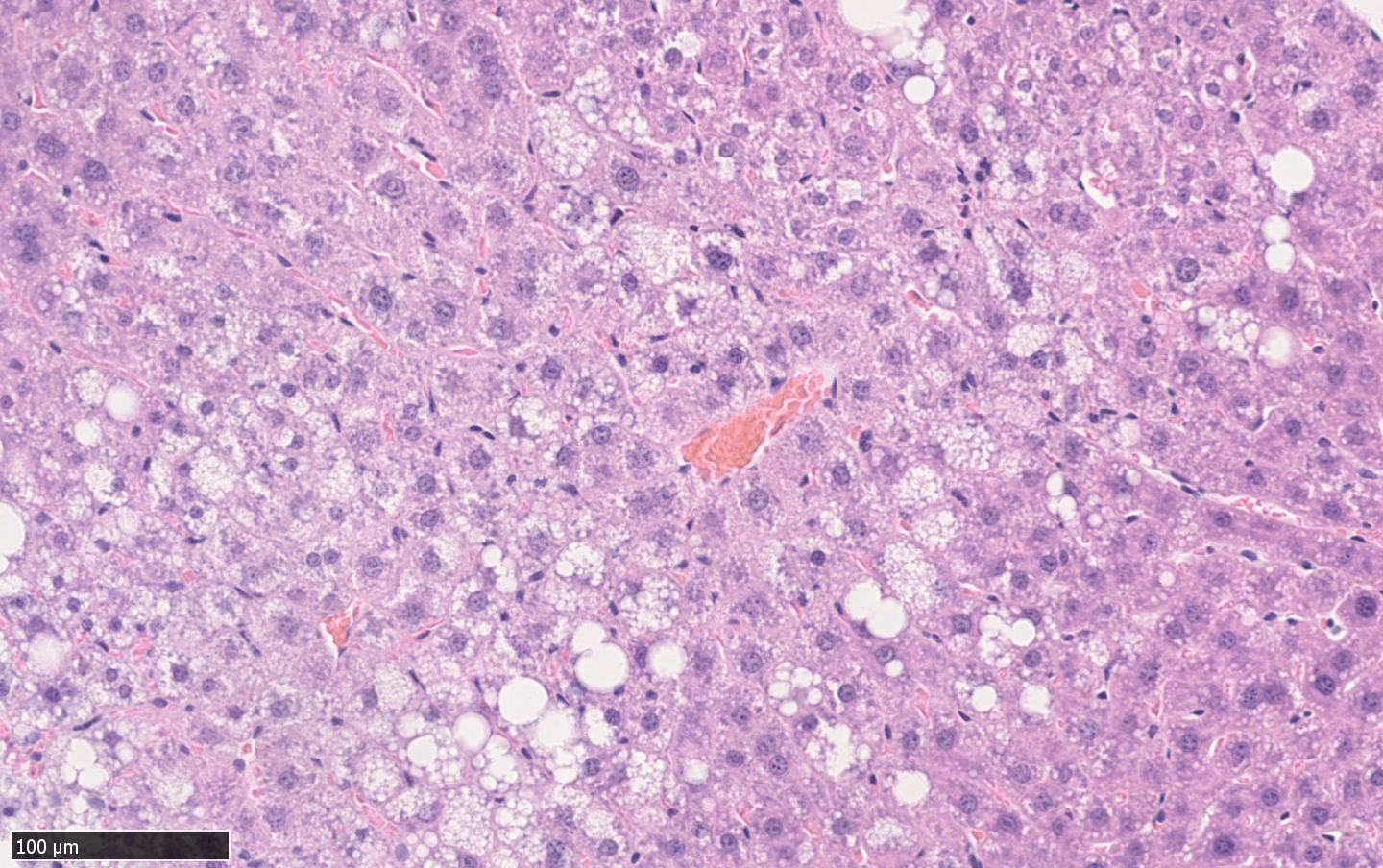

Supplement: Supplementary file 4 [file DataSheet9.ZIP › NASH SCORE-db(1)/db17,18/14.jpg]

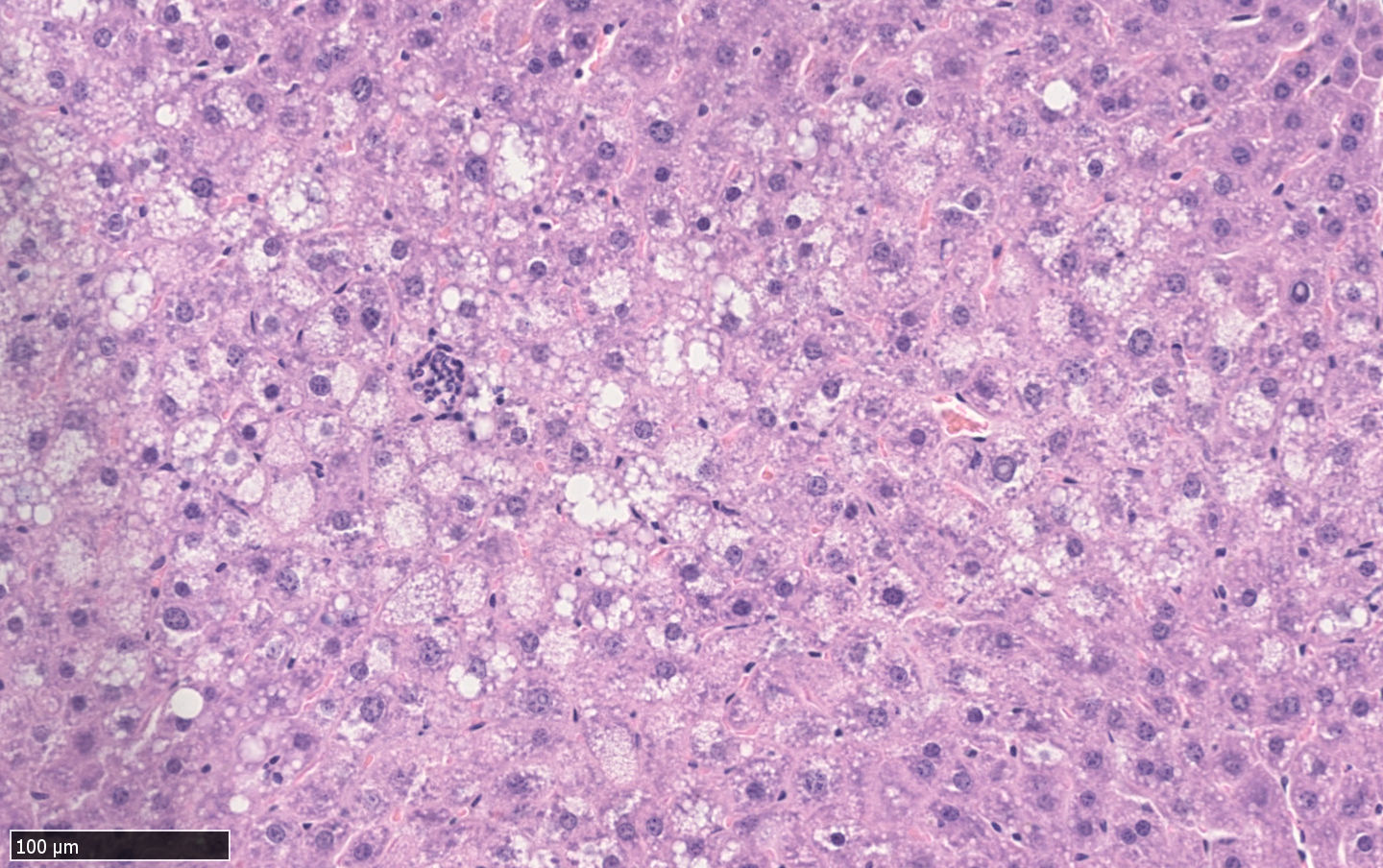

Supplement: Supplementary file 4 [file DataSheet9.ZIP › NASH SCORE-db(1)/db17,18/15.jpg]

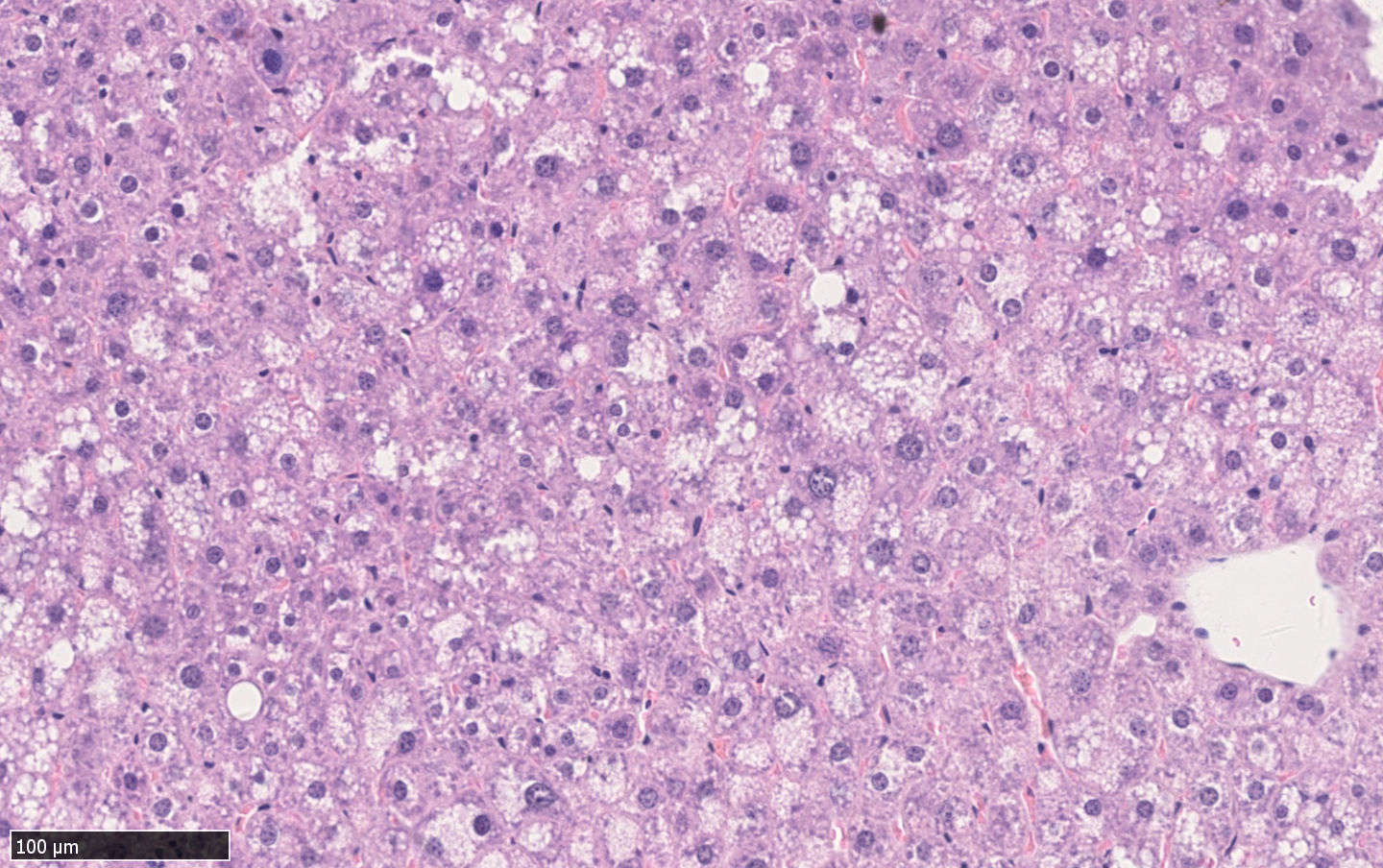

Supplement: Supplementary file 4 [file DataSheet9.ZIP › NASH SCORE-db(1)/db17,18/16.jpg]

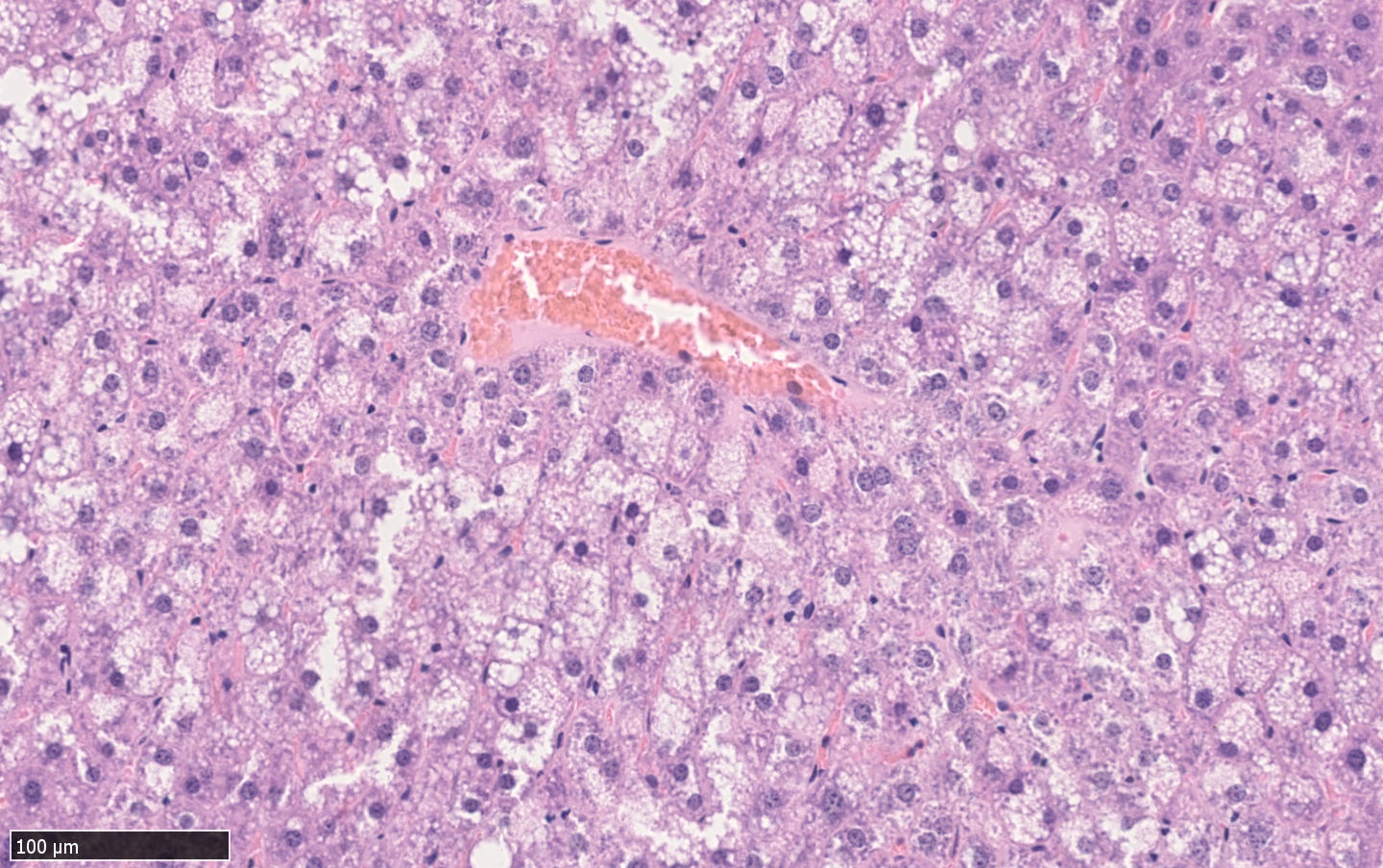

Supplement: Supplementary file 4 [file DataSheet9.ZIP › NASH SCORE-db(1)/db17,18/17.jpg]

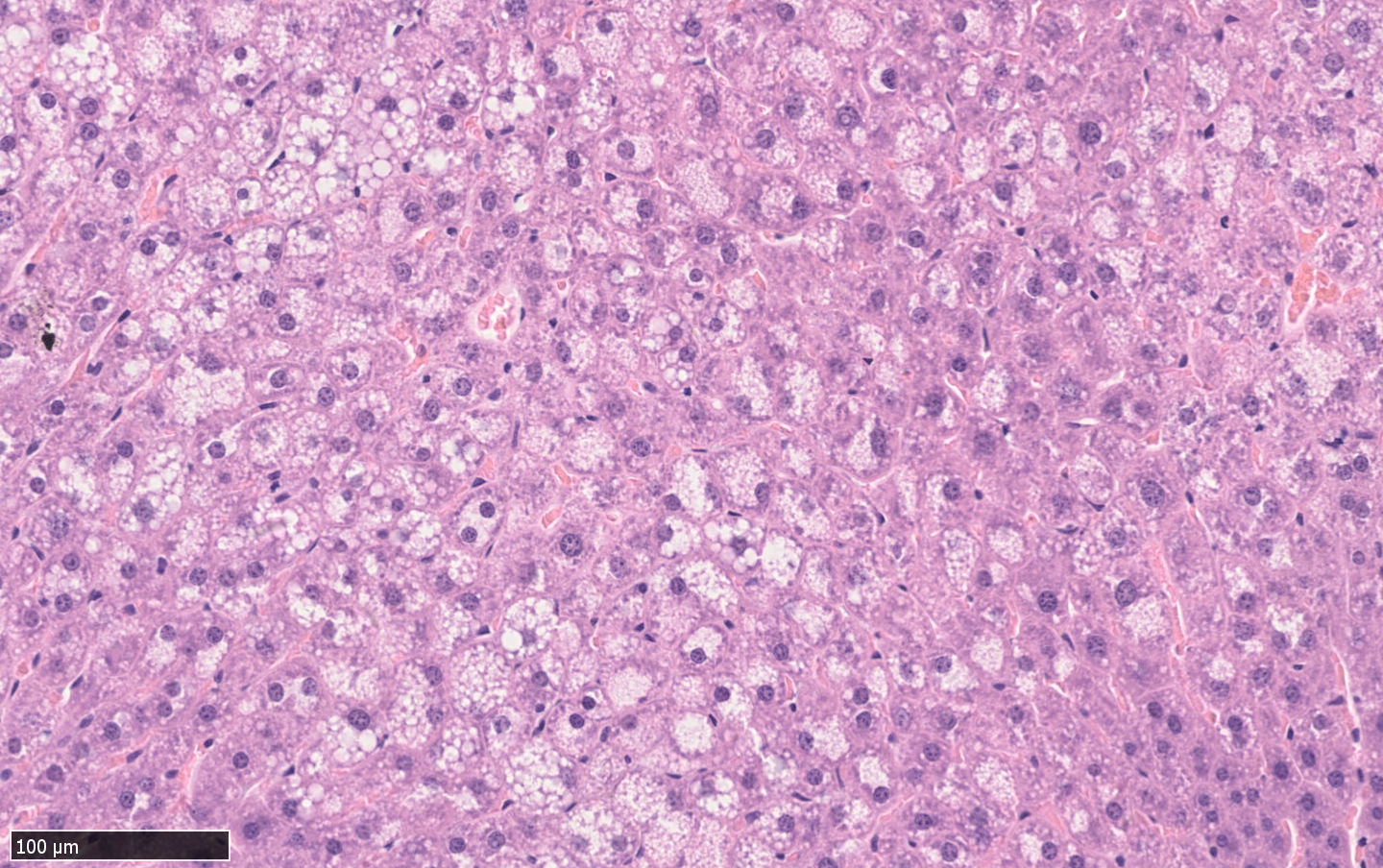

Supplement: Supplementary file 4 [file DataSheet9.ZIP › NASH SCORE-db(1)/db17,18/18.jpg]

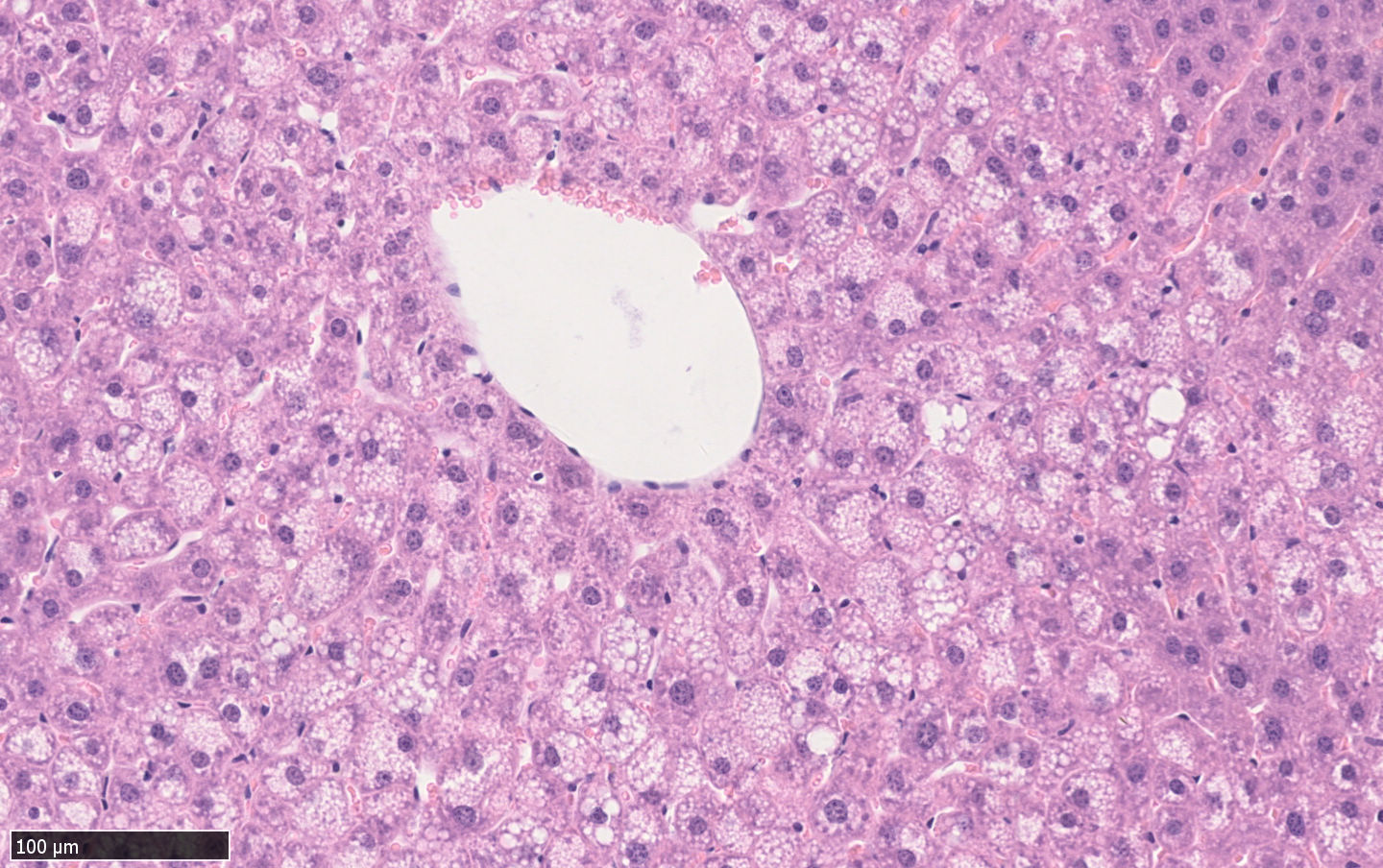

Supplement: Supplementary file 4 [file DataSheet9.ZIP › NASH SCORE-db(1)/db17,18/19.jpg]

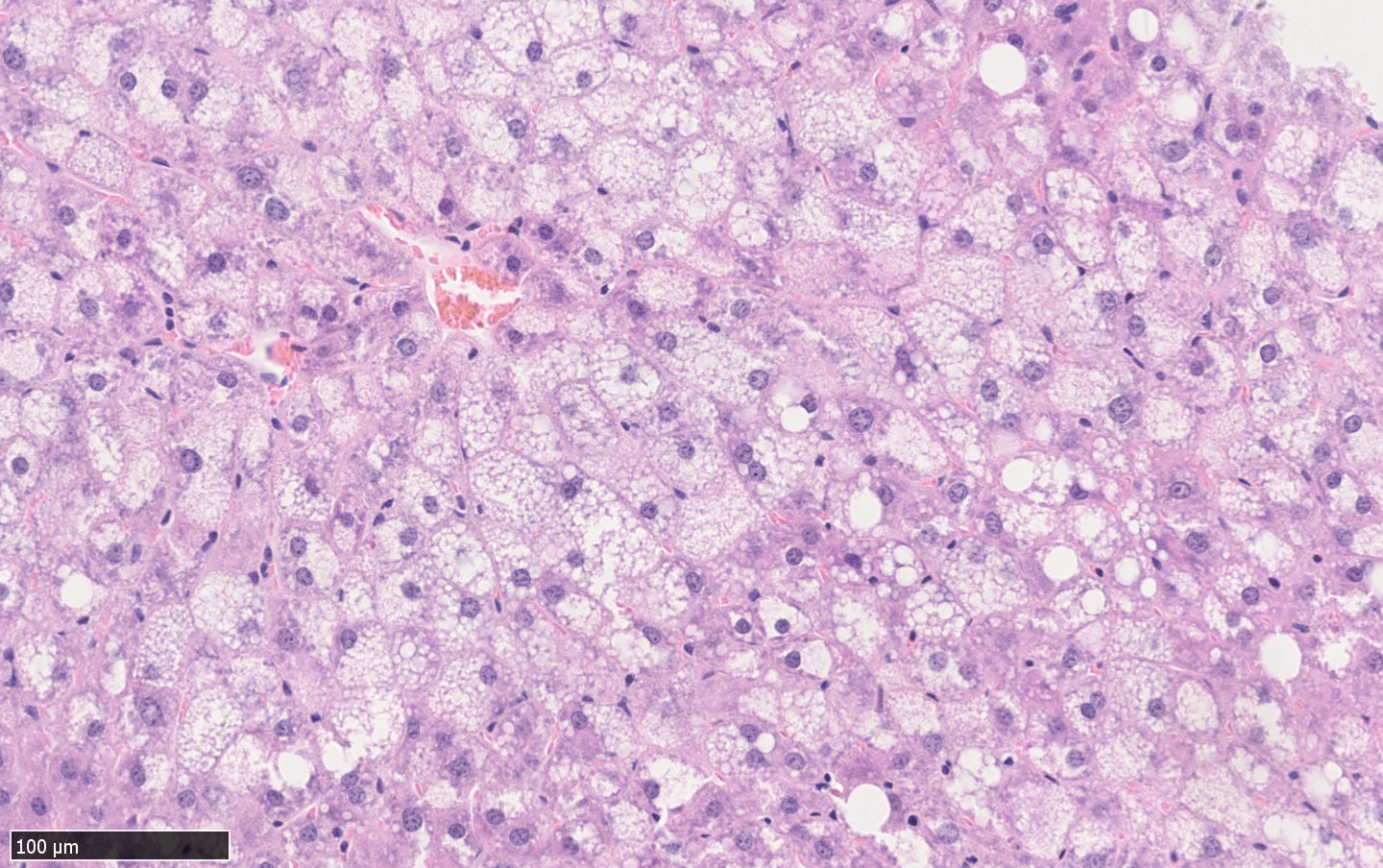

Supplement: Supplementary file 4 [file DataSheet9.ZIP › NASH SCORE-db(1)/db17,18/2.jpg]

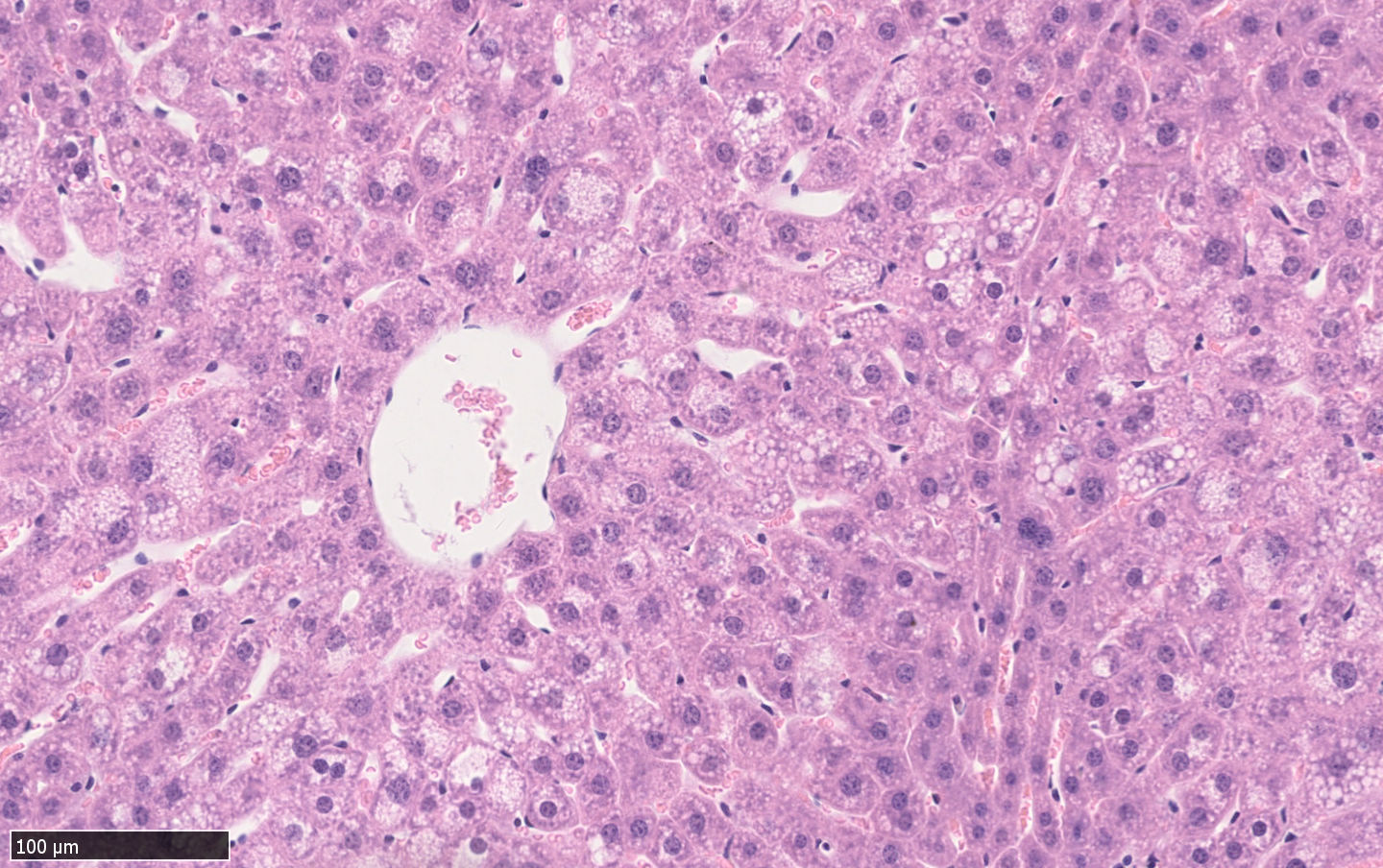

Supplement: Supplementary file 4 [file DataSheet9.ZIP › NASH SCORE-db(1)/db17,18/20.jpg]

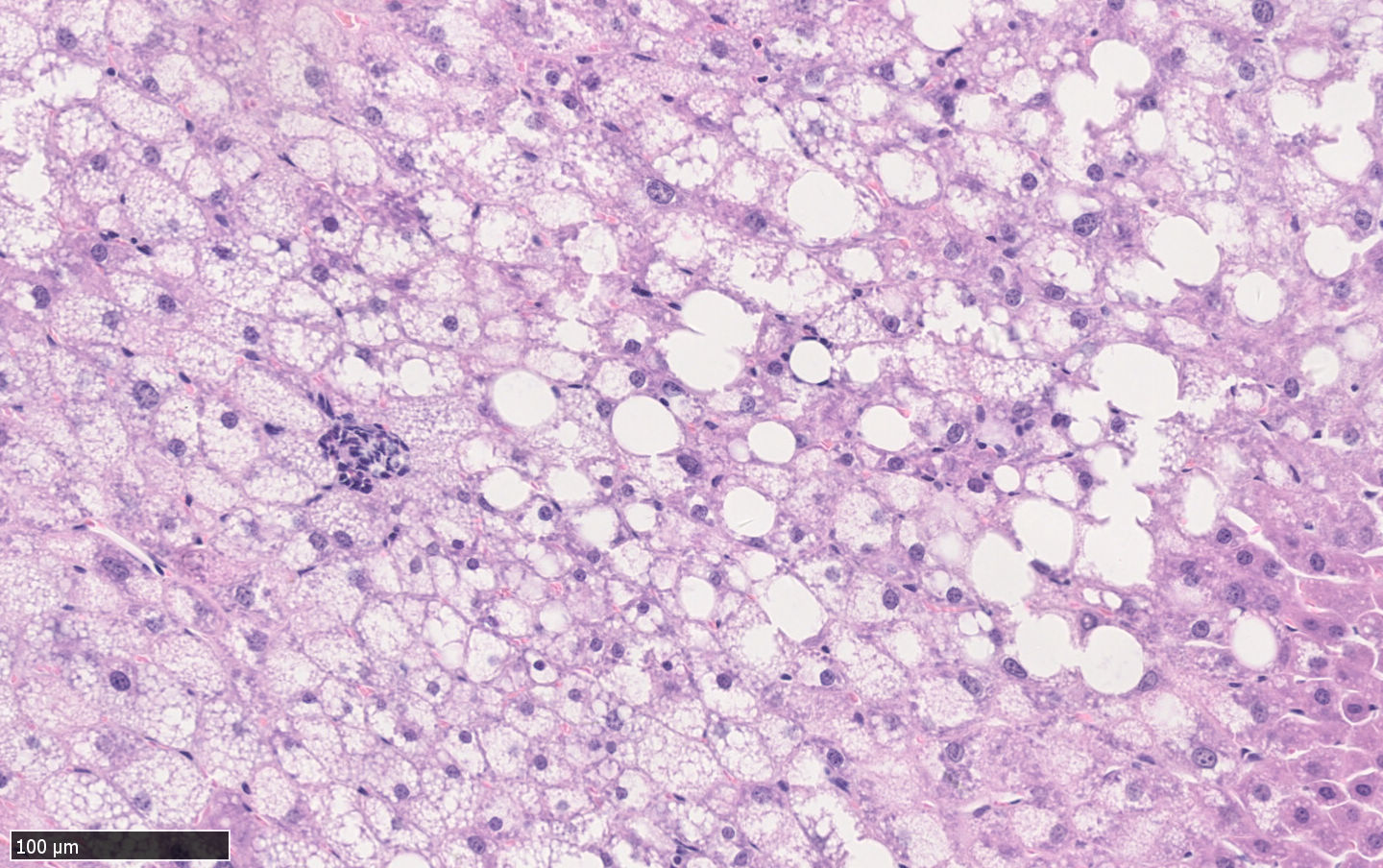

Supplement: Supplementary file 4 [file DataSheet9.ZIP › NASH SCORE-db(1)/db17,18/3.jpg]

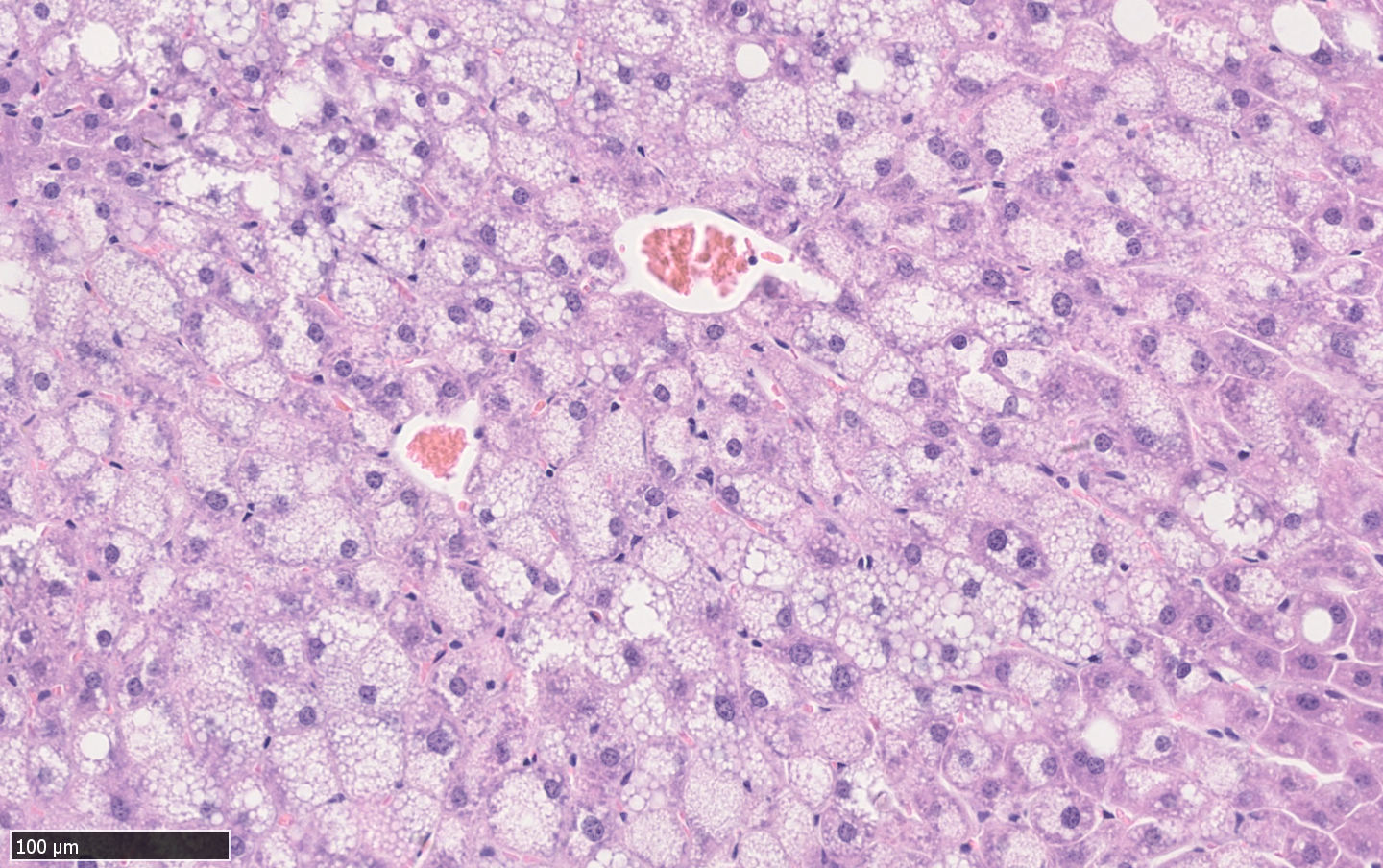

Supplement: Supplementary file 4 [file DataSheet9.ZIP › NASH SCORE-db(1)/db17,18/4.jpg]

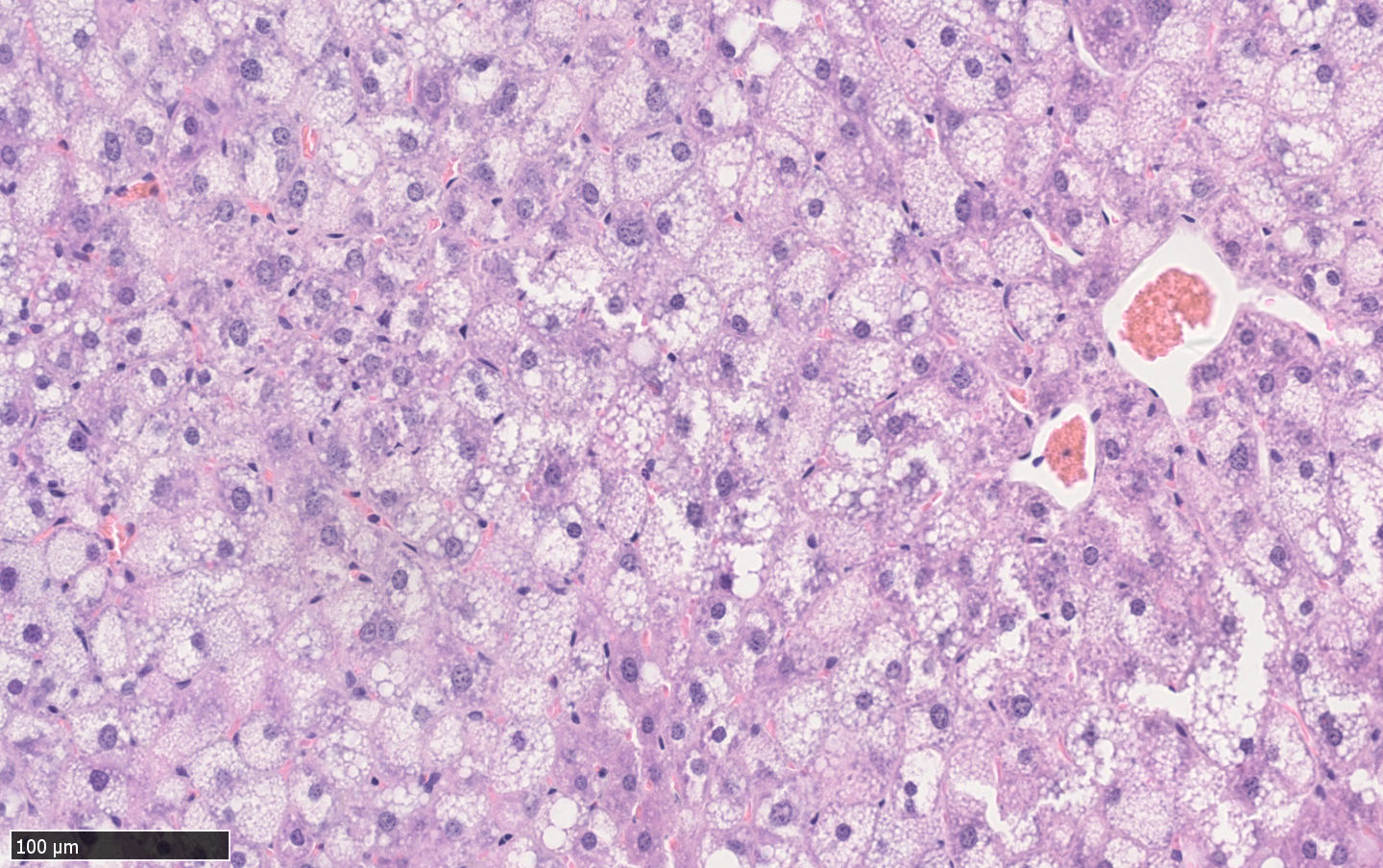

Supplement: Supplementary file 4 [file DataSheet9.ZIP › NASH SCORE-db(1)/db17,18/5.jpg]

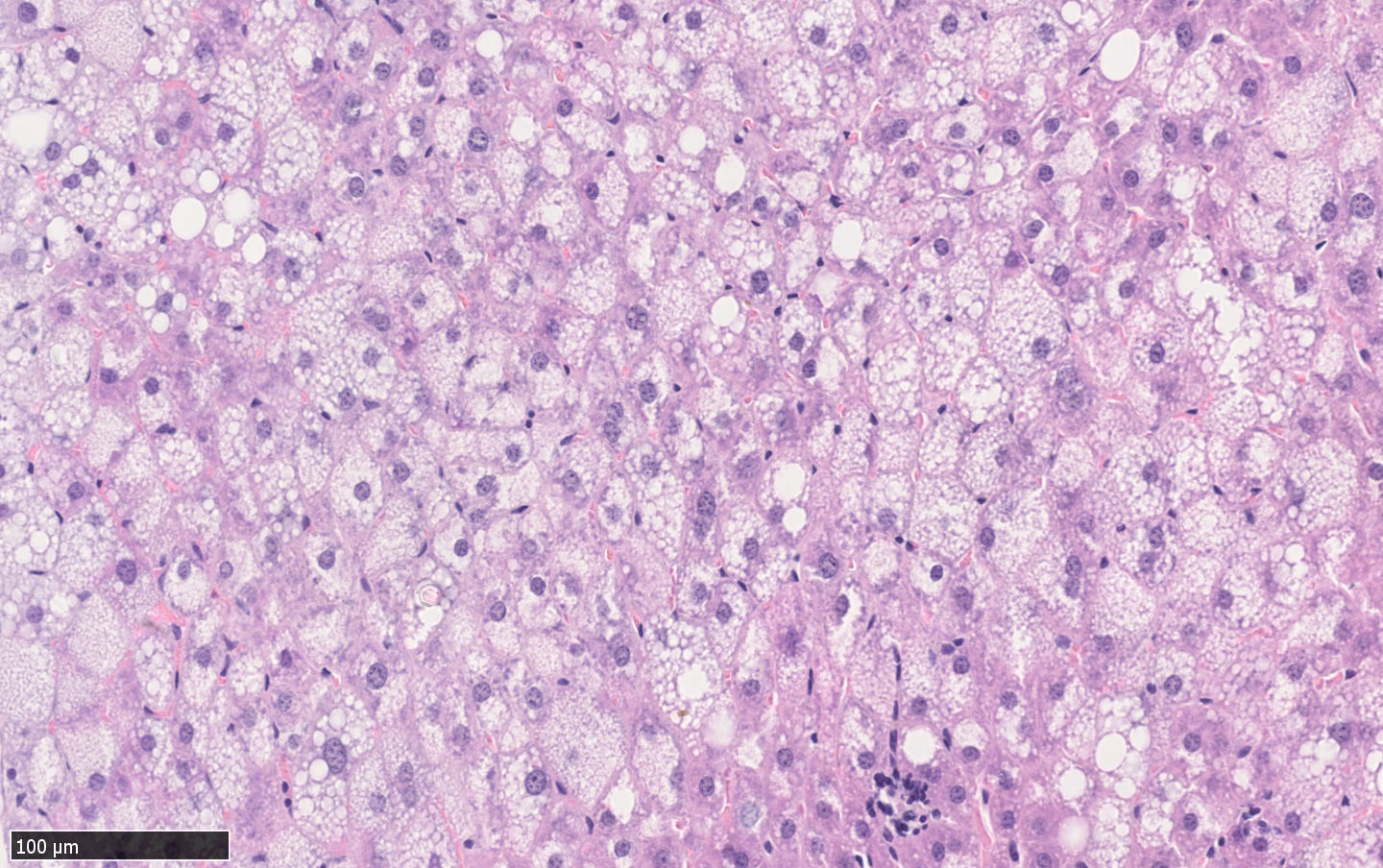

Supplement: Supplementary file 4 [file DataSheet9.ZIP › NASH SCORE-db(1)/db17,18/6.jpg]

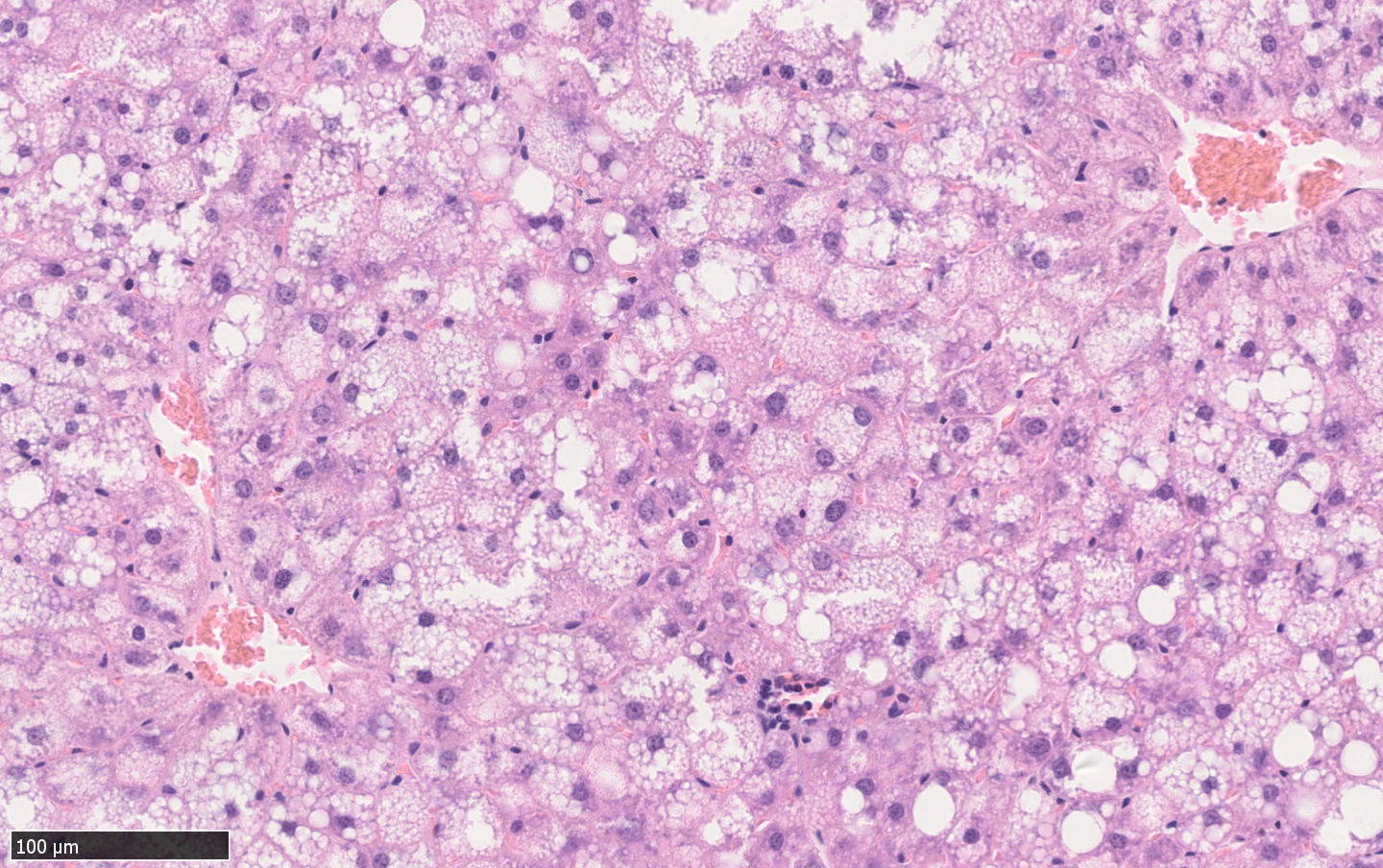

Supplement: Supplementary file 4 [file DataSheet9.ZIP › NASH SCORE-db(1)/db17,18/7.jpg]

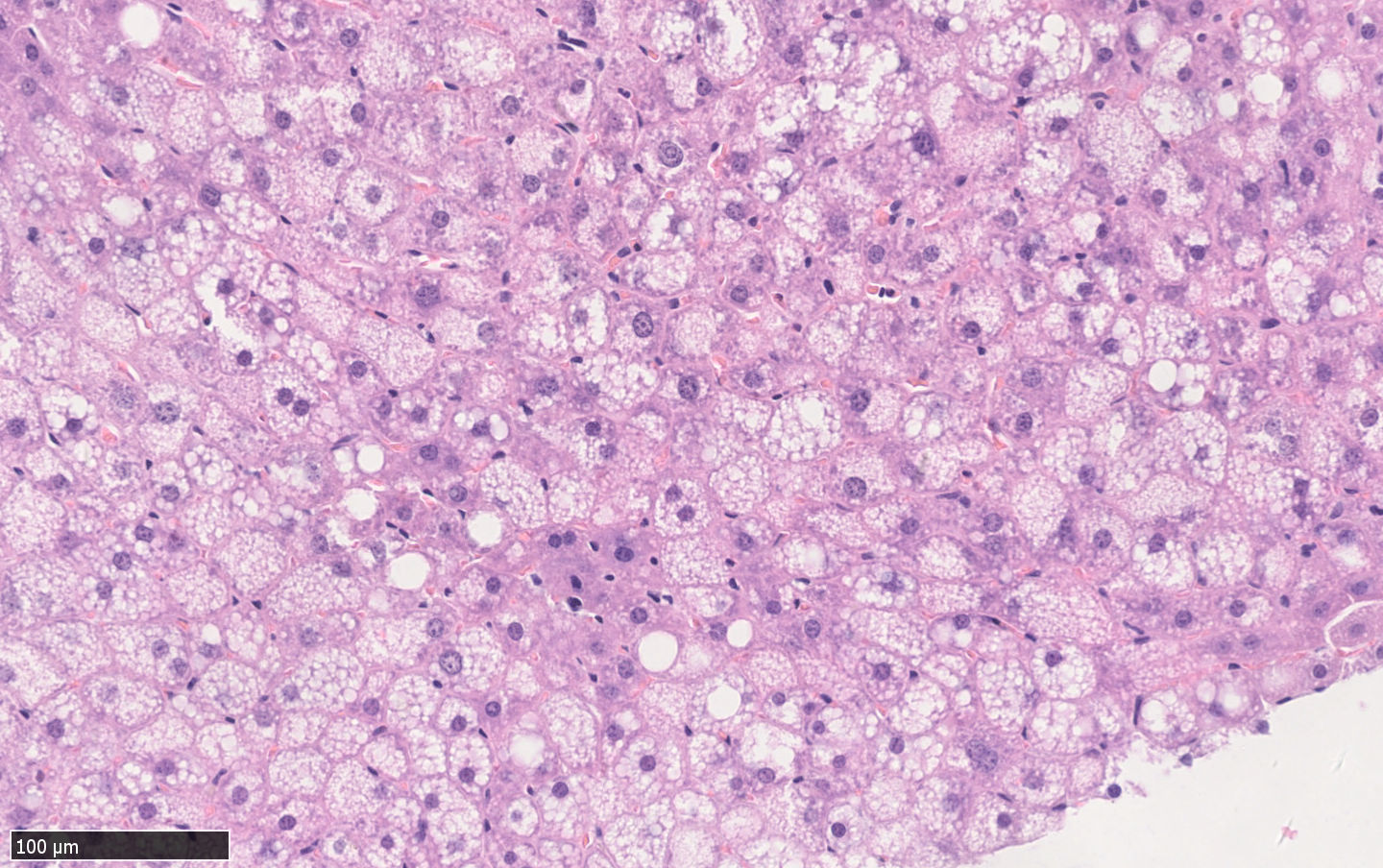

Supplement: Supplementary file 4 [file DataSheet9.ZIP › NASH SCORE-db(1)/db17,18/8.jpg]

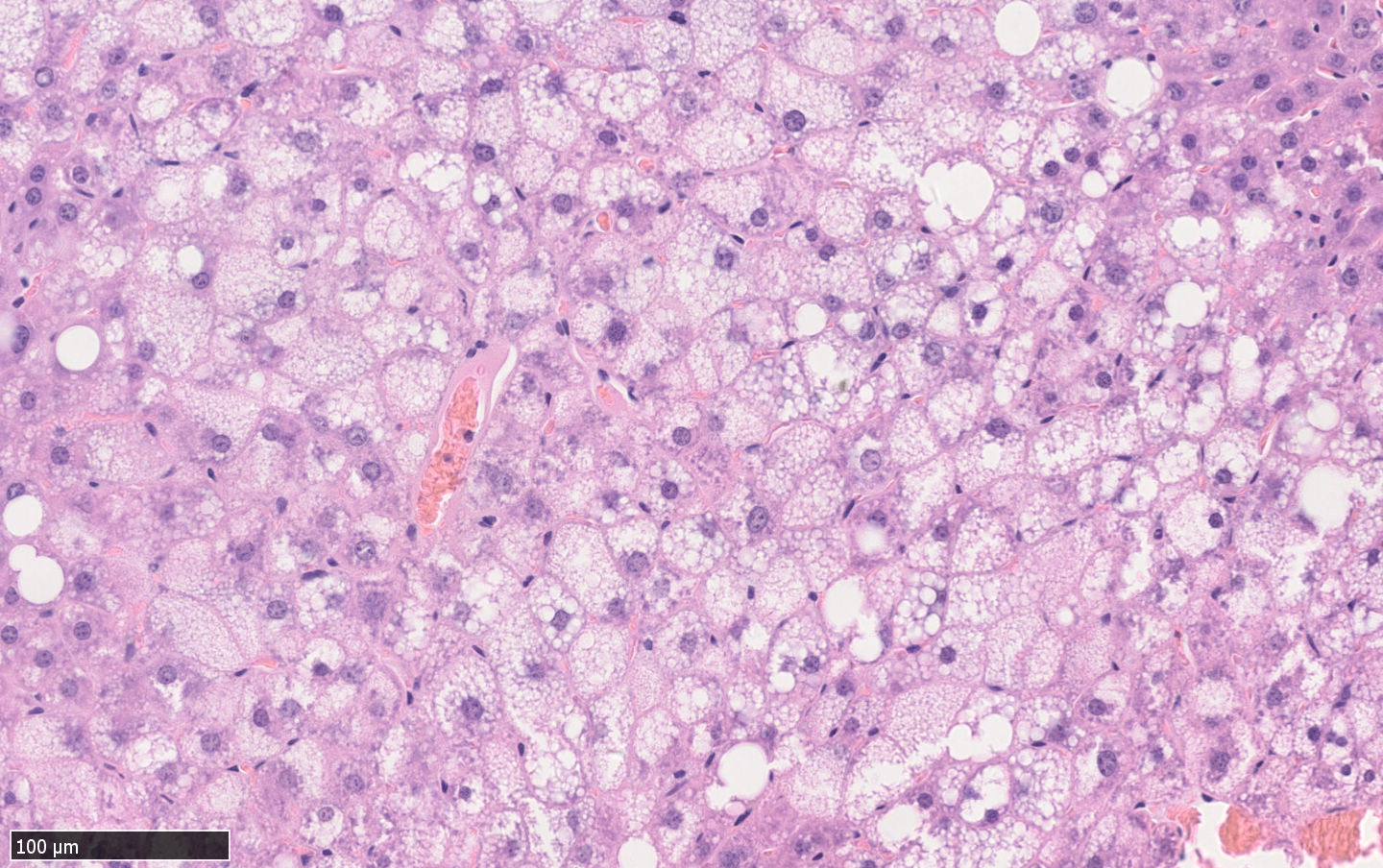

Supplement: Supplementary file 4 [file DataSheet9.ZIP › NASH SCORE-db(1)/db17,18/9.jpg]

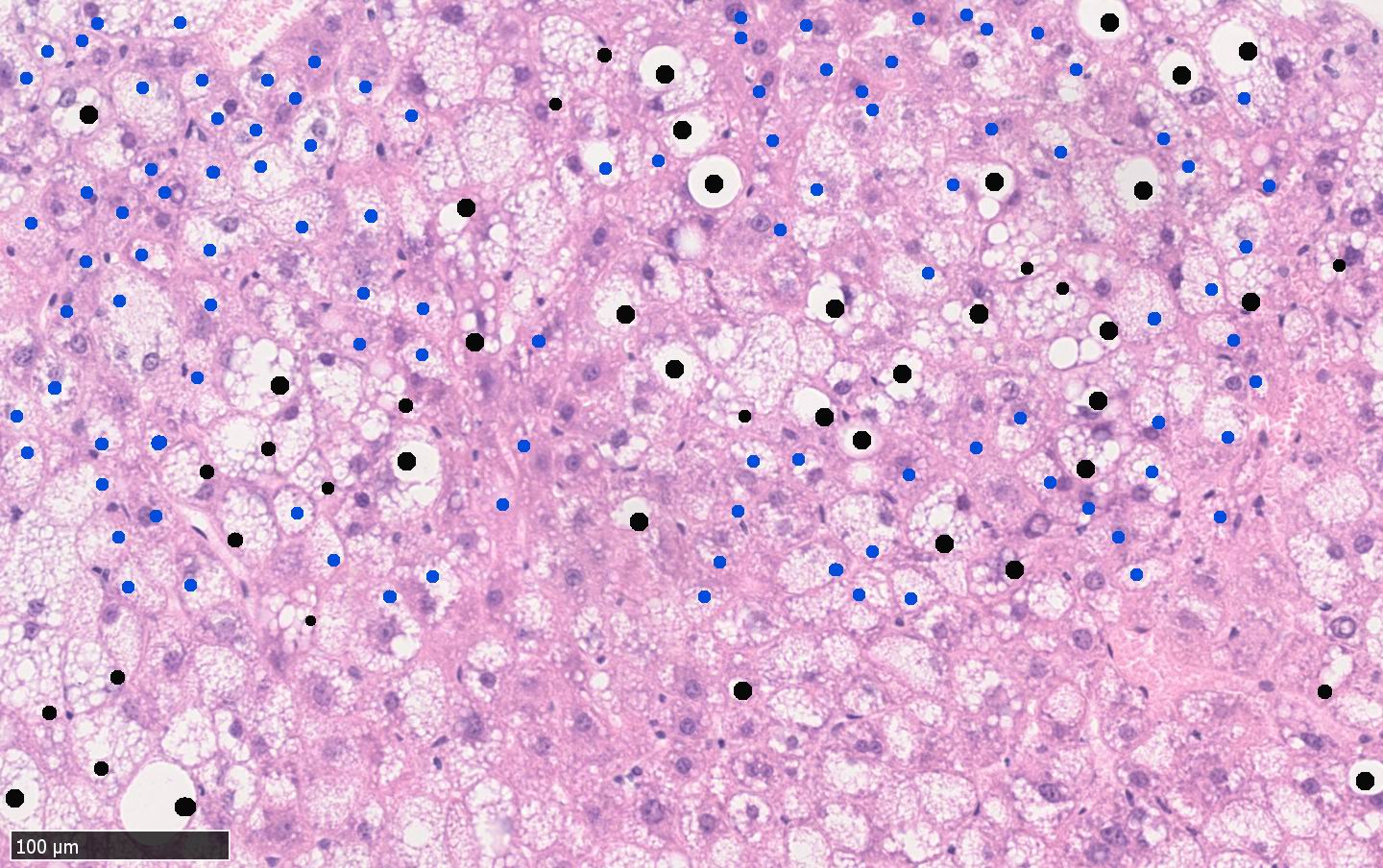

Supplement: Supplementary file 4 [file DataSheet9.ZIP › NASH SCORE-db(1)/db8,16/1.jpg]

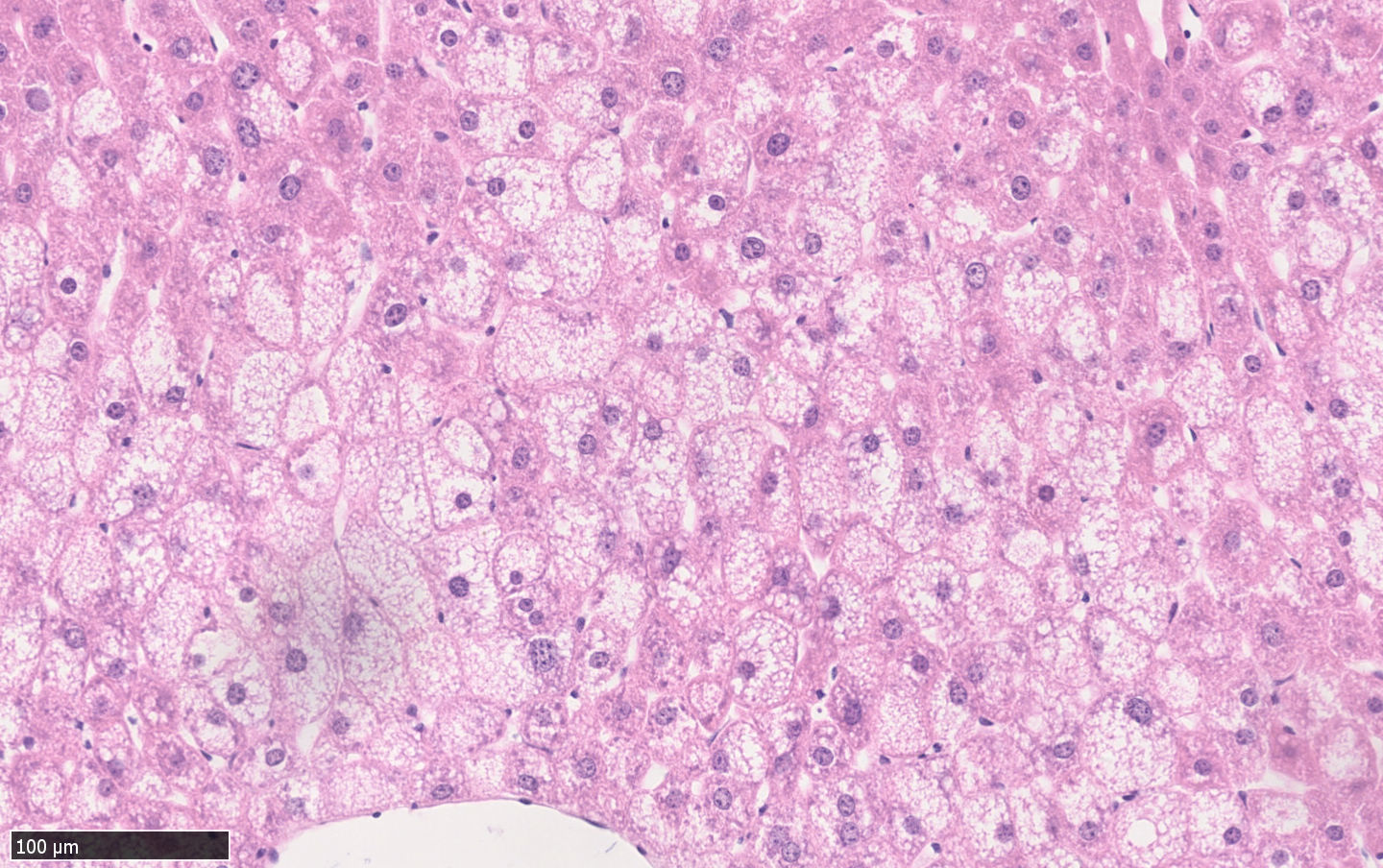

Supplement: Supplementary file 4 [file DataSheet9.ZIP › NASH SCORE-db(1)/db8,16/10.jpg]

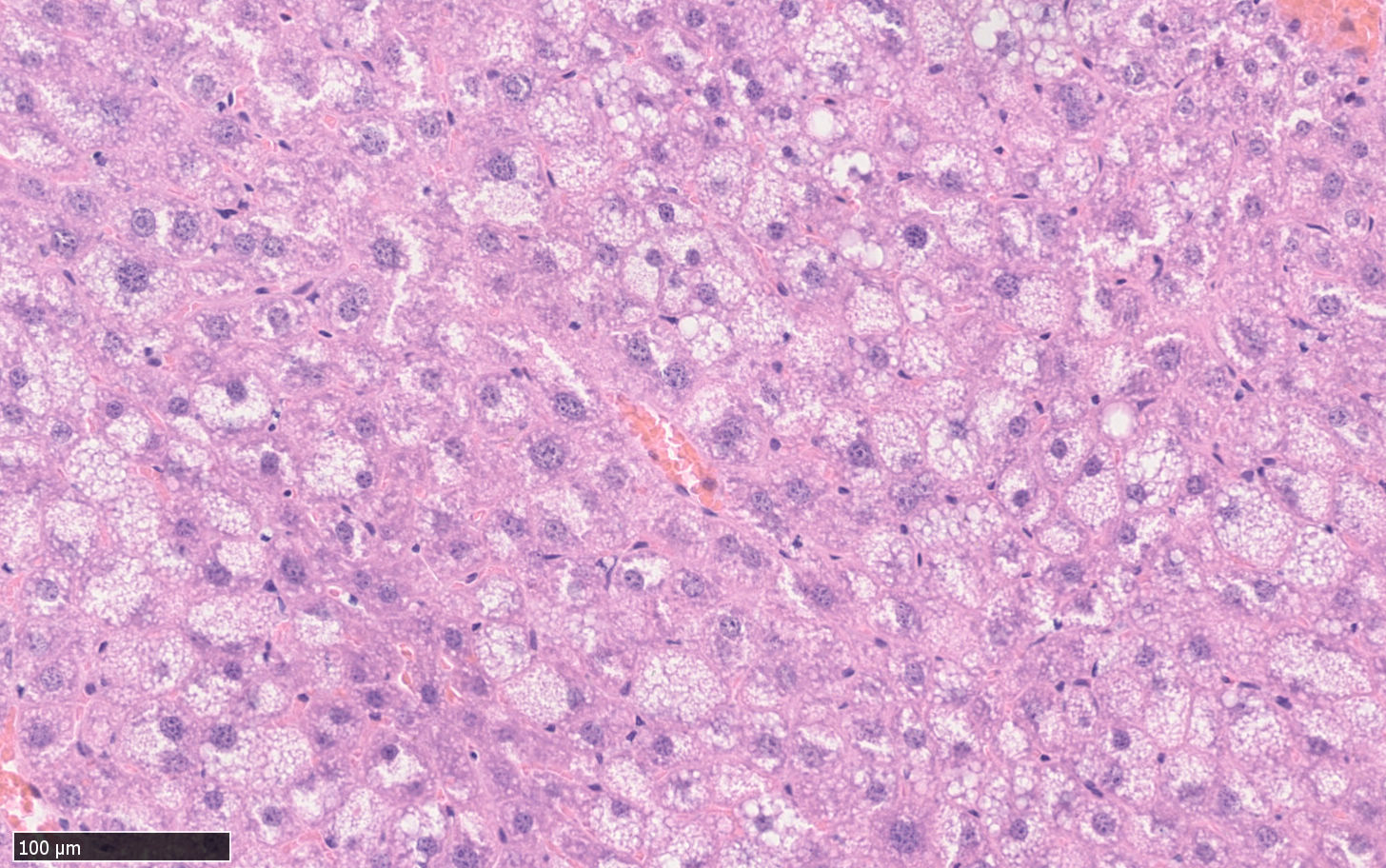

Supplement: Supplementary file 4 [file DataSheet9.ZIP › NASH SCORE-db(1)/db8,16/11.jpg]

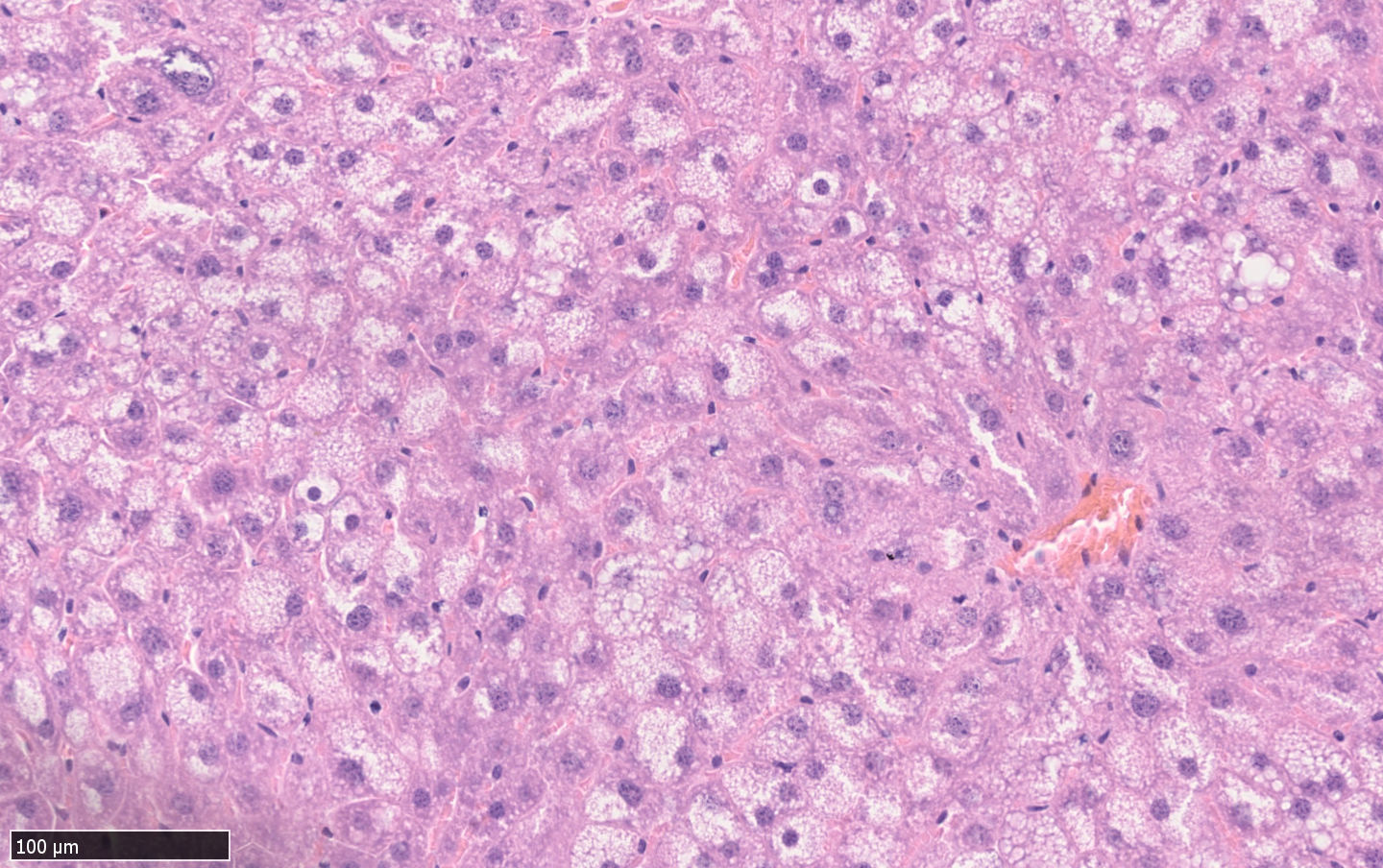

Supplement: Supplementary file 4 [file DataSheet9.ZIP › NASH SCORE-db(1)/db8,16/12.jpg]

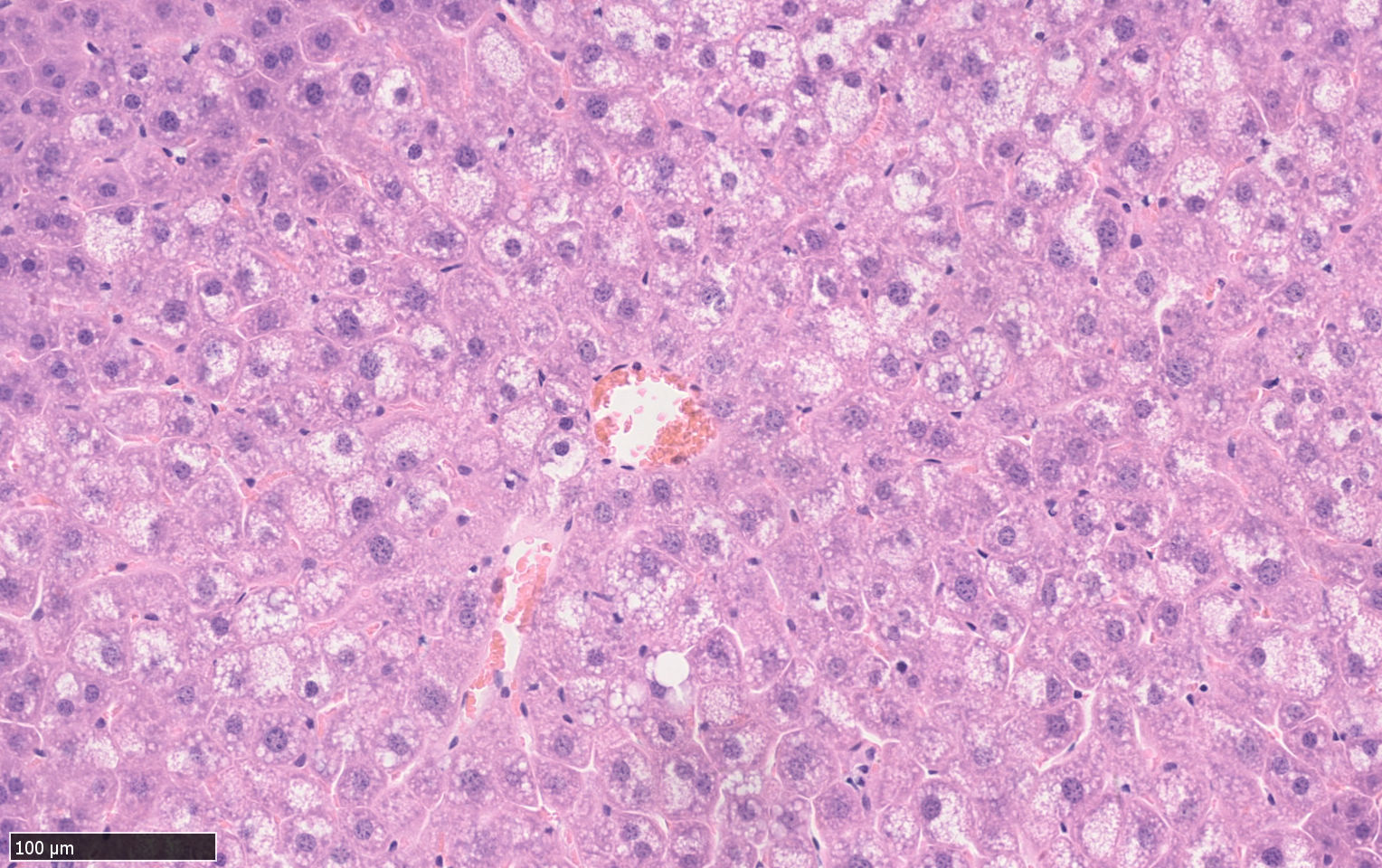

Supplement: Supplementary file 4 [file DataSheet9.ZIP › NASH SCORE-db(1)/db8,16/13.jpg]

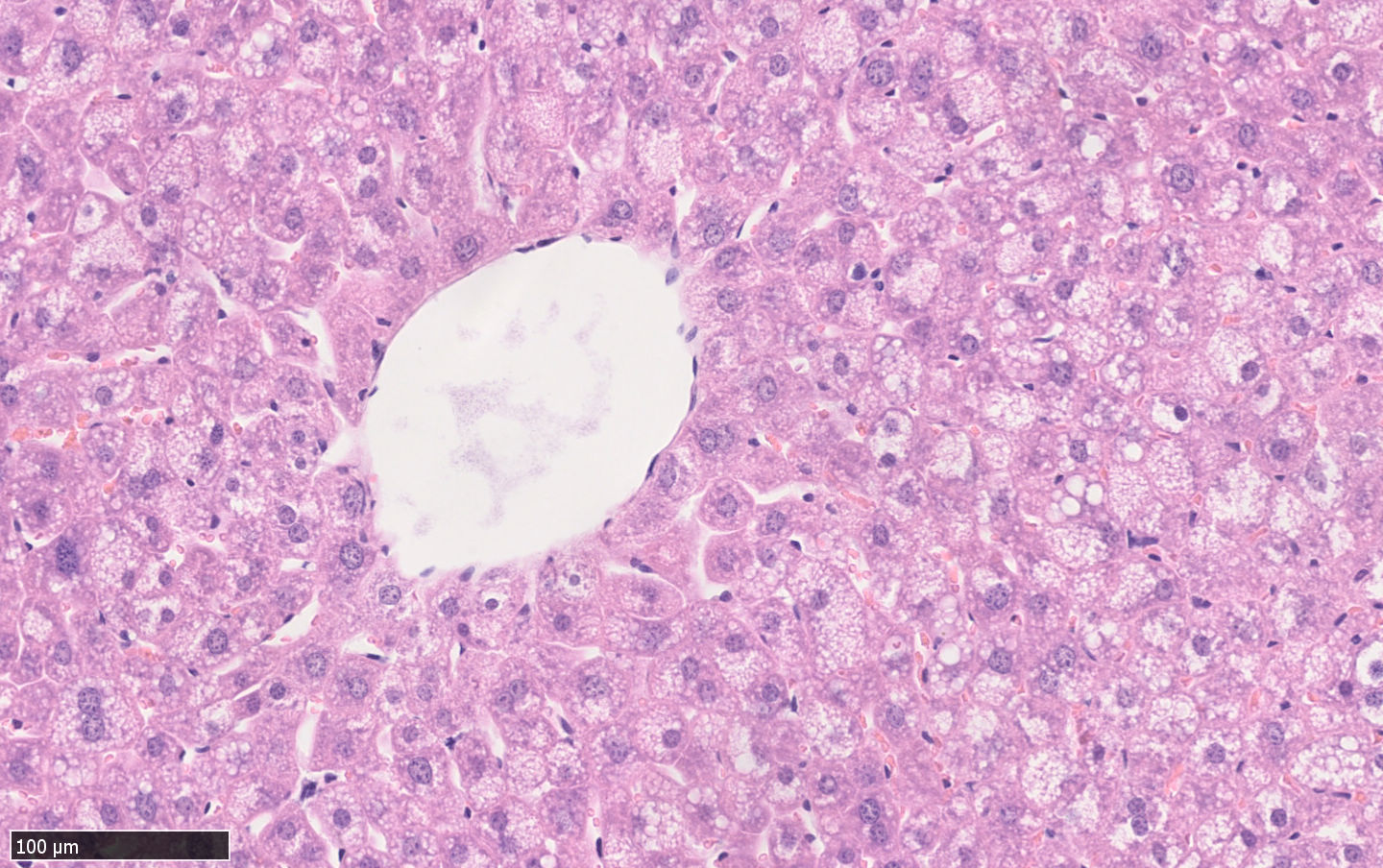

Supplement: Supplementary file 4 [file DataSheet9.ZIP › NASH SCORE-db(1)/db8,16/14.jpg]

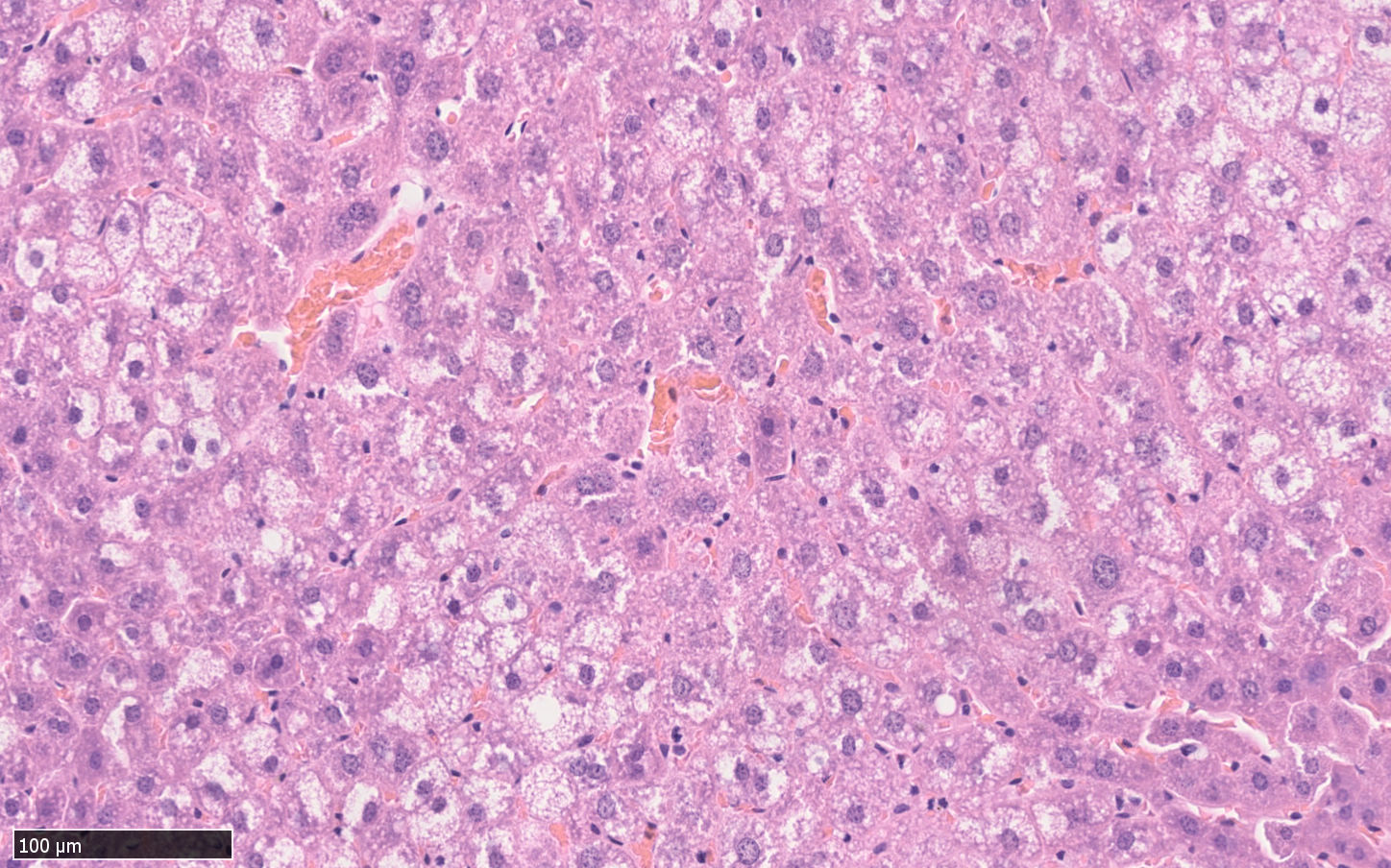

Supplement: Supplementary file 4 [file DataSheet9.ZIP › NASH SCORE-db(1)/db8,16/15.jpg]

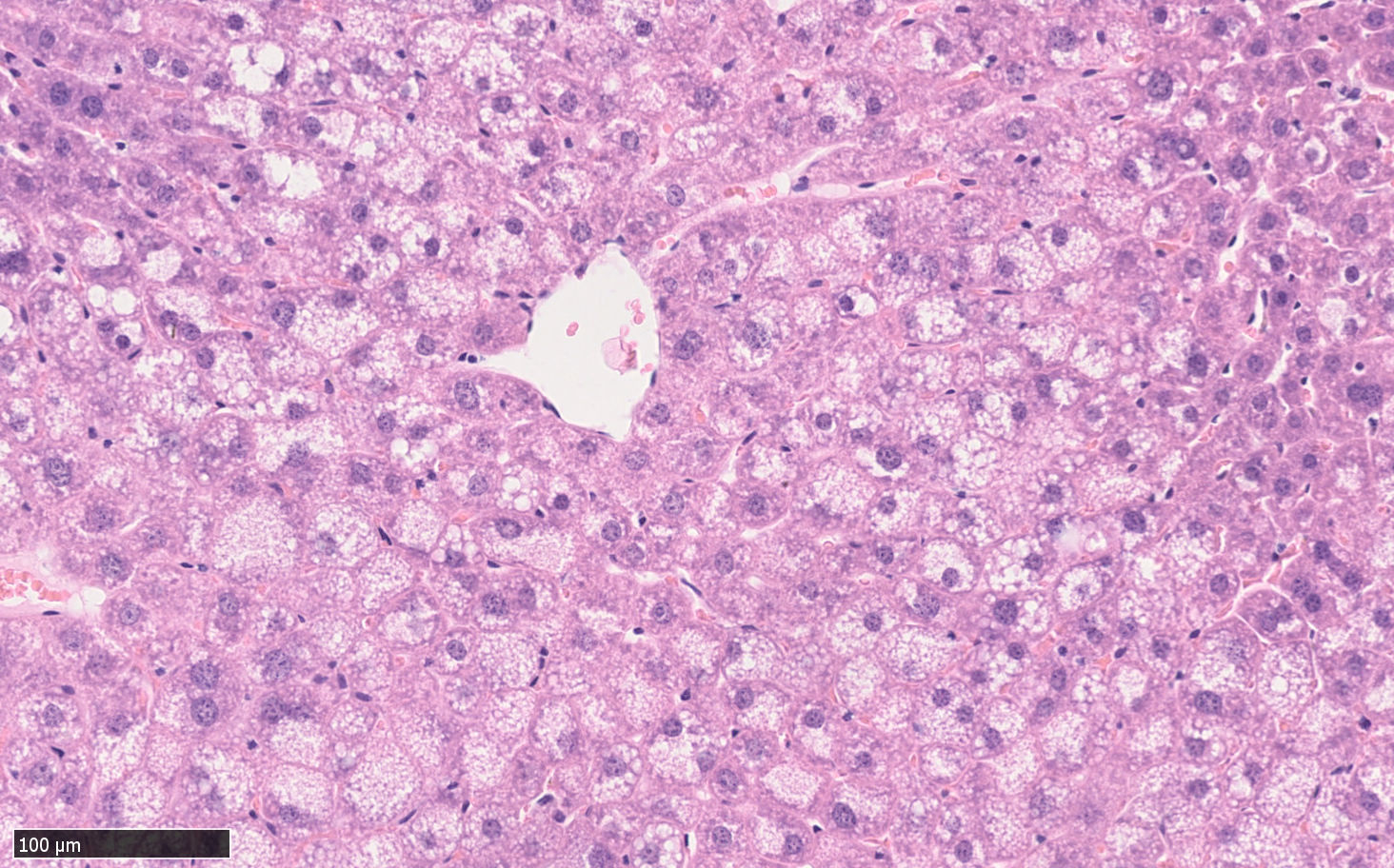

Supplement: Supplementary file 4 [file DataSheet9.ZIP › NASH SCORE-db(1)/db8,16/16.jpg]

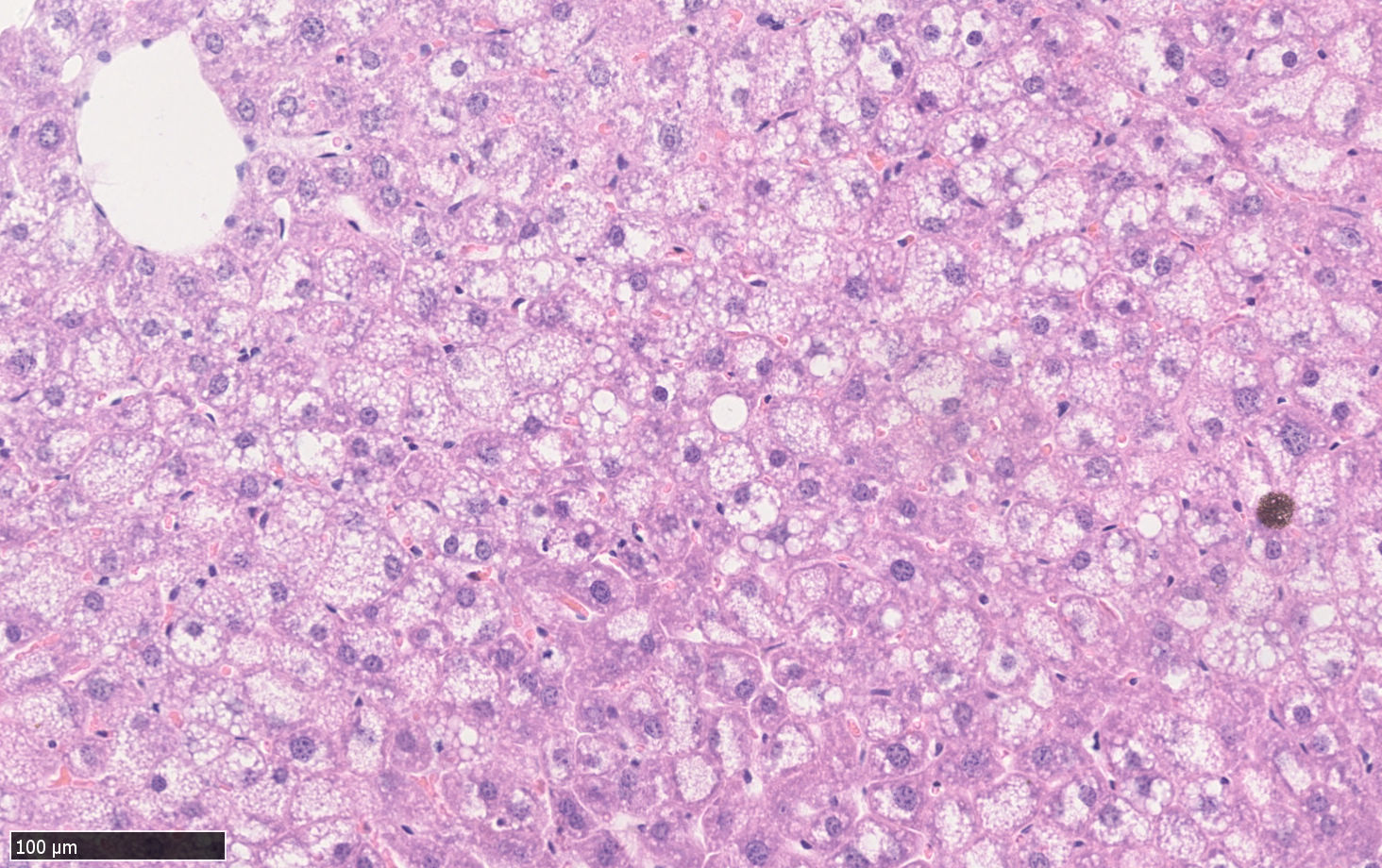

Supplement: Supplementary file 4 [file DataSheet9.ZIP › NASH SCORE-db(1)/db8,16/17.jpg]

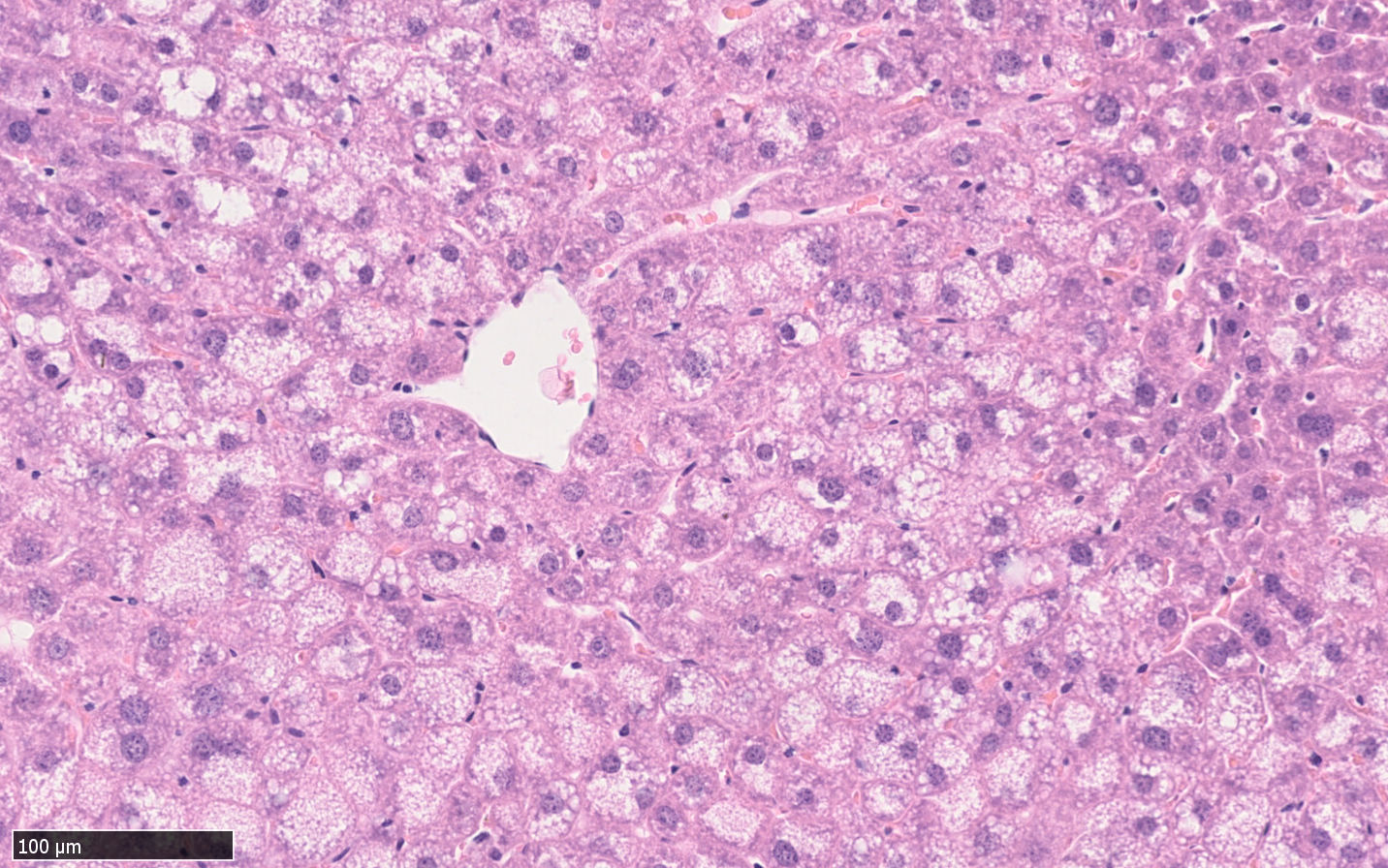

Supplement: Supplementary file 4 [file DataSheet9.ZIP › NASH SCORE-db(1)/db8,16/18.jpg]

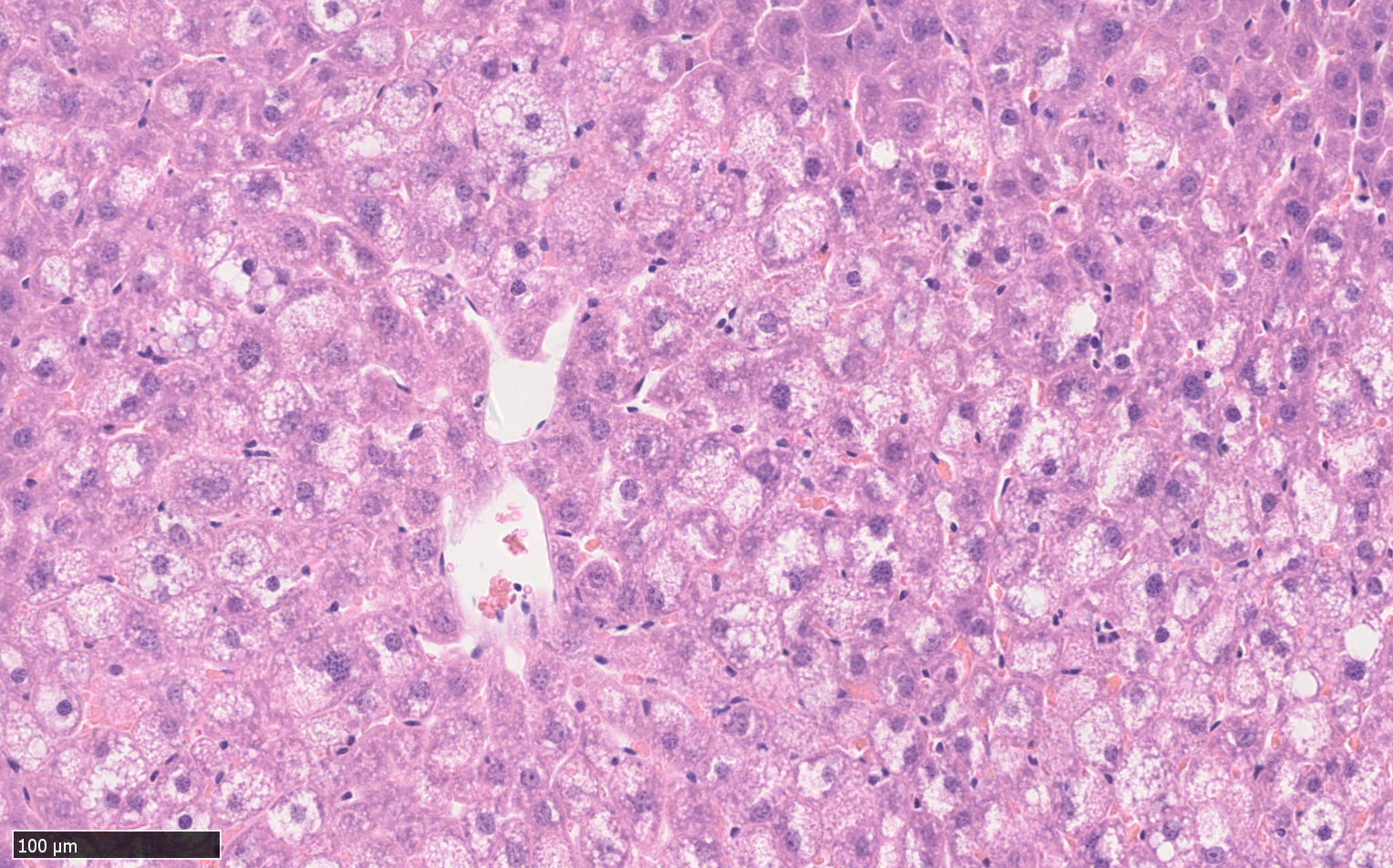

Supplement: Supplementary file 4 [file DataSheet9.ZIP › NASH SCORE-db(1)/db8,16/19.jpg]

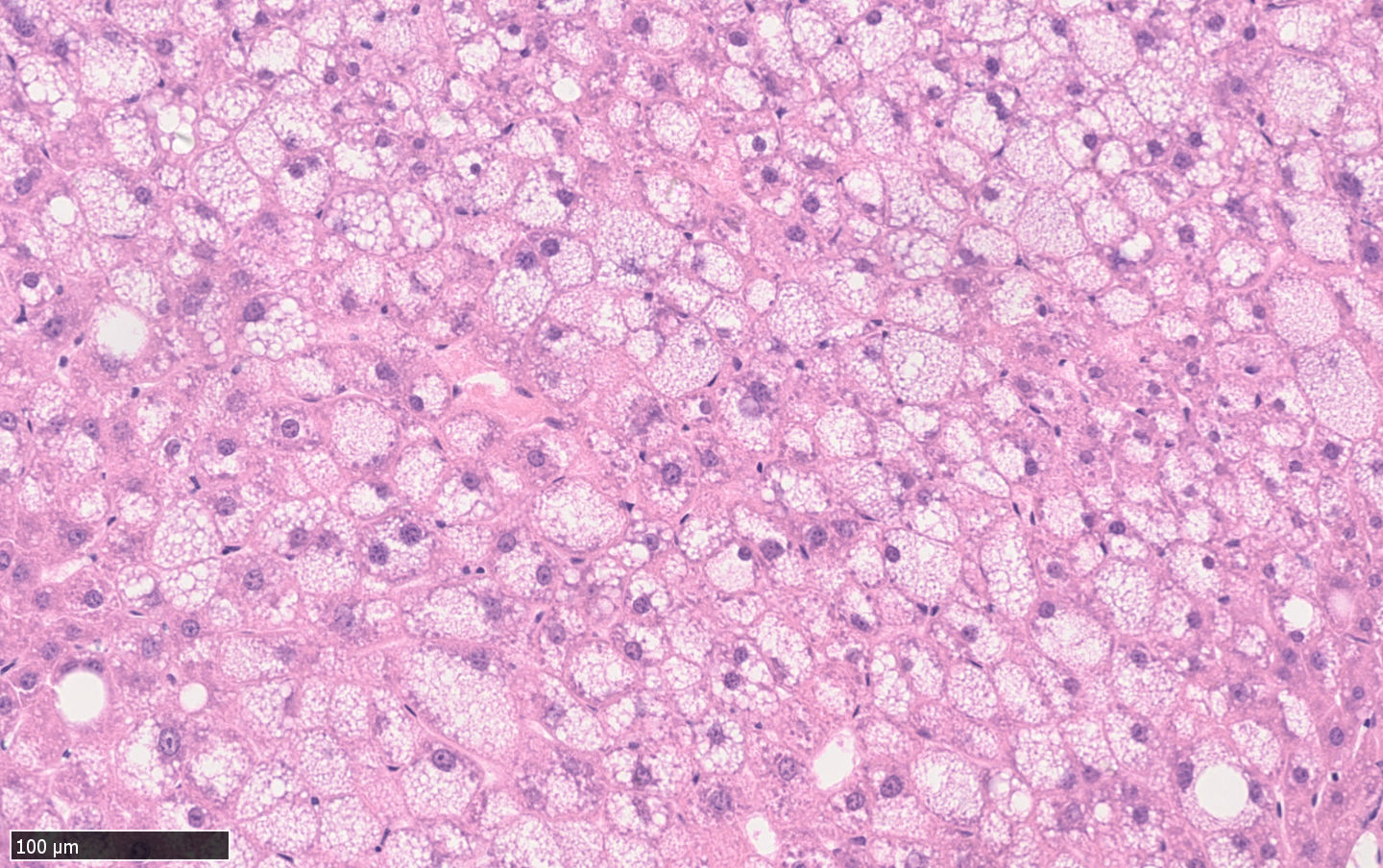

Supplement: Supplementary file 4 [file DataSheet9.ZIP › NASH SCORE-db(1)/db8,16/2.jpg]

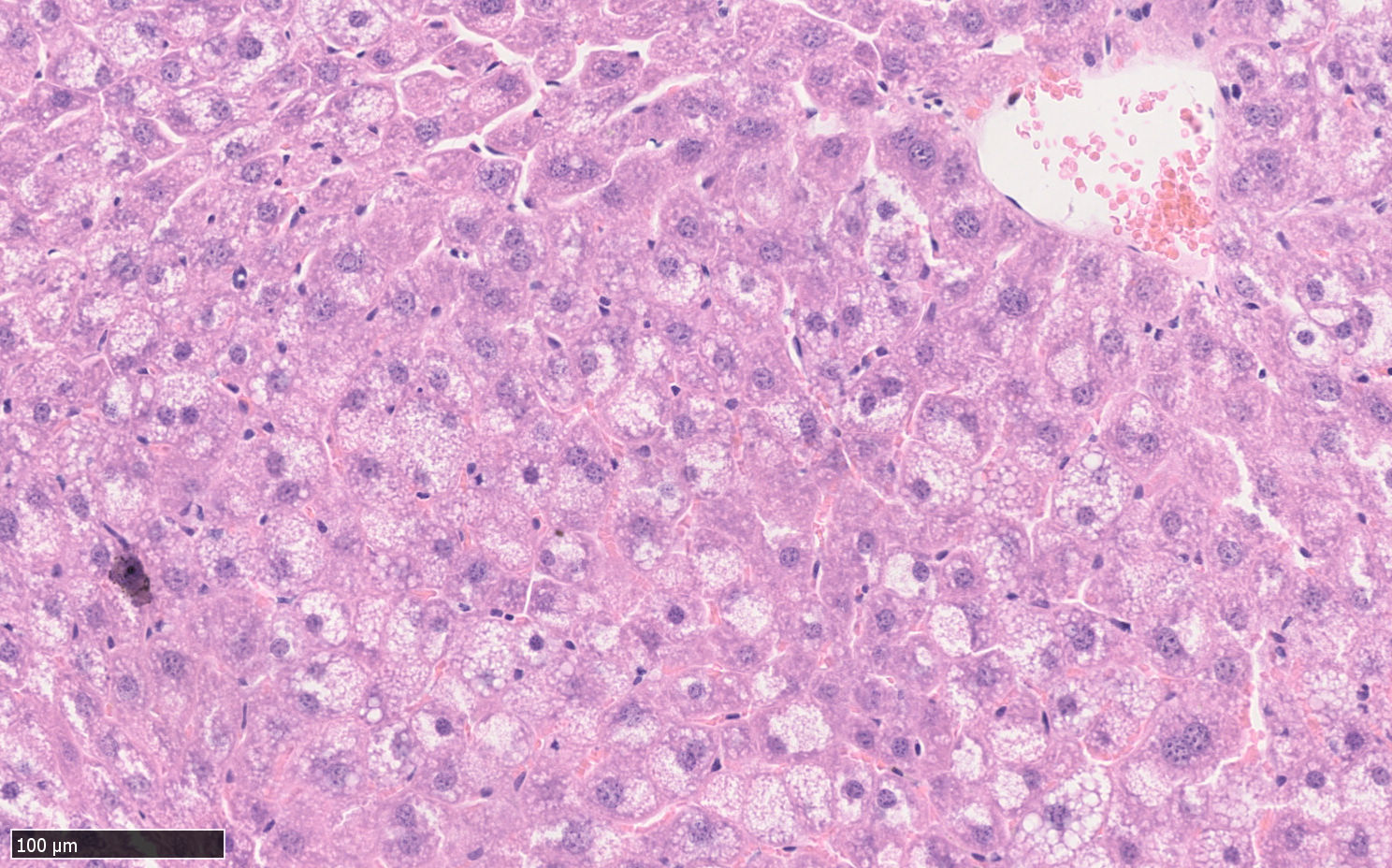

Supplement: Supplementary file 4 [file DataSheet9.ZIP › NASH SCORE-db(1)/db8,16/20.jpg]

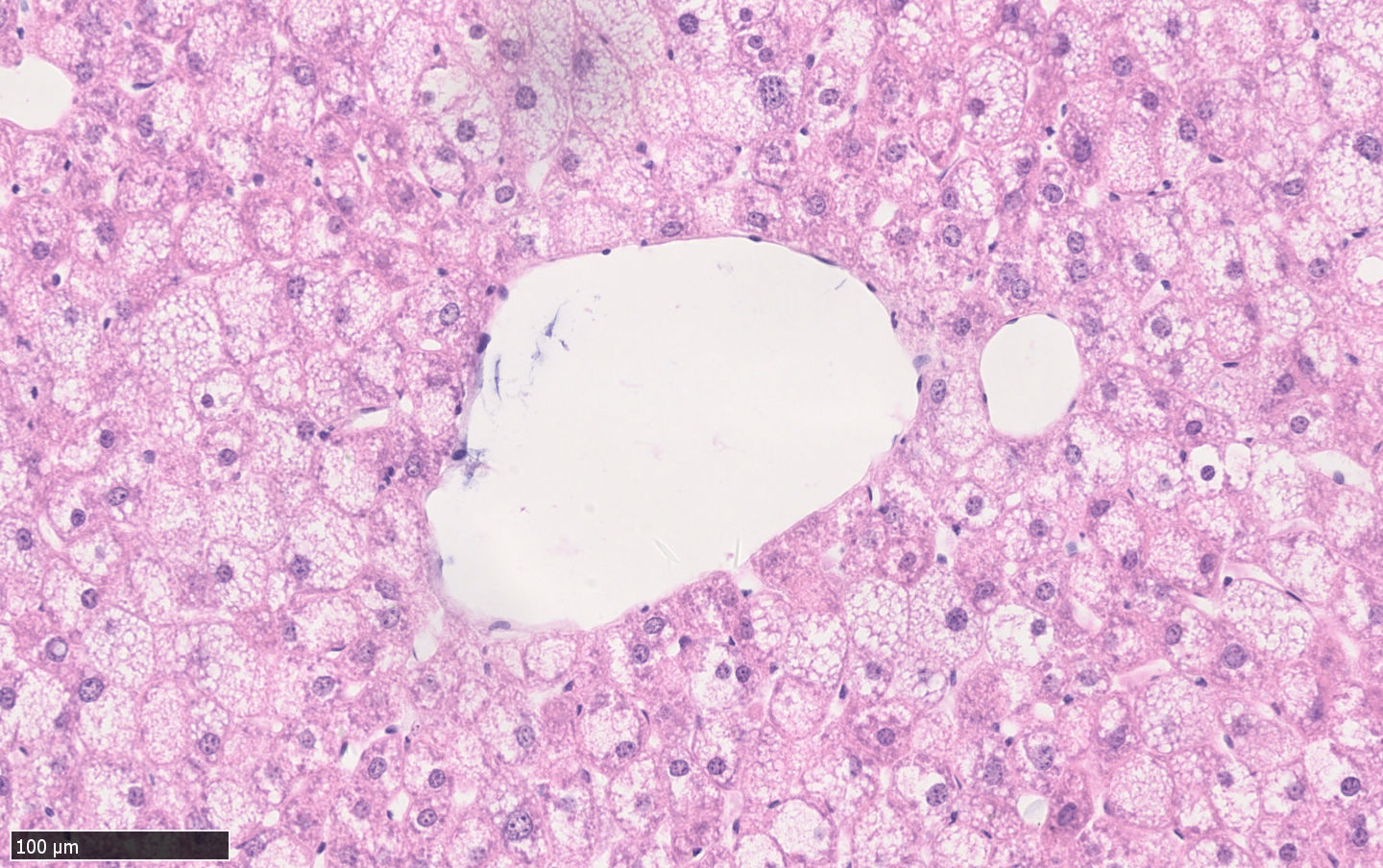

Supplement: Supplementary file 4 [file DataSheet9.ZIP › NASH SCORE-db(1)/db8,16/3.jpg]

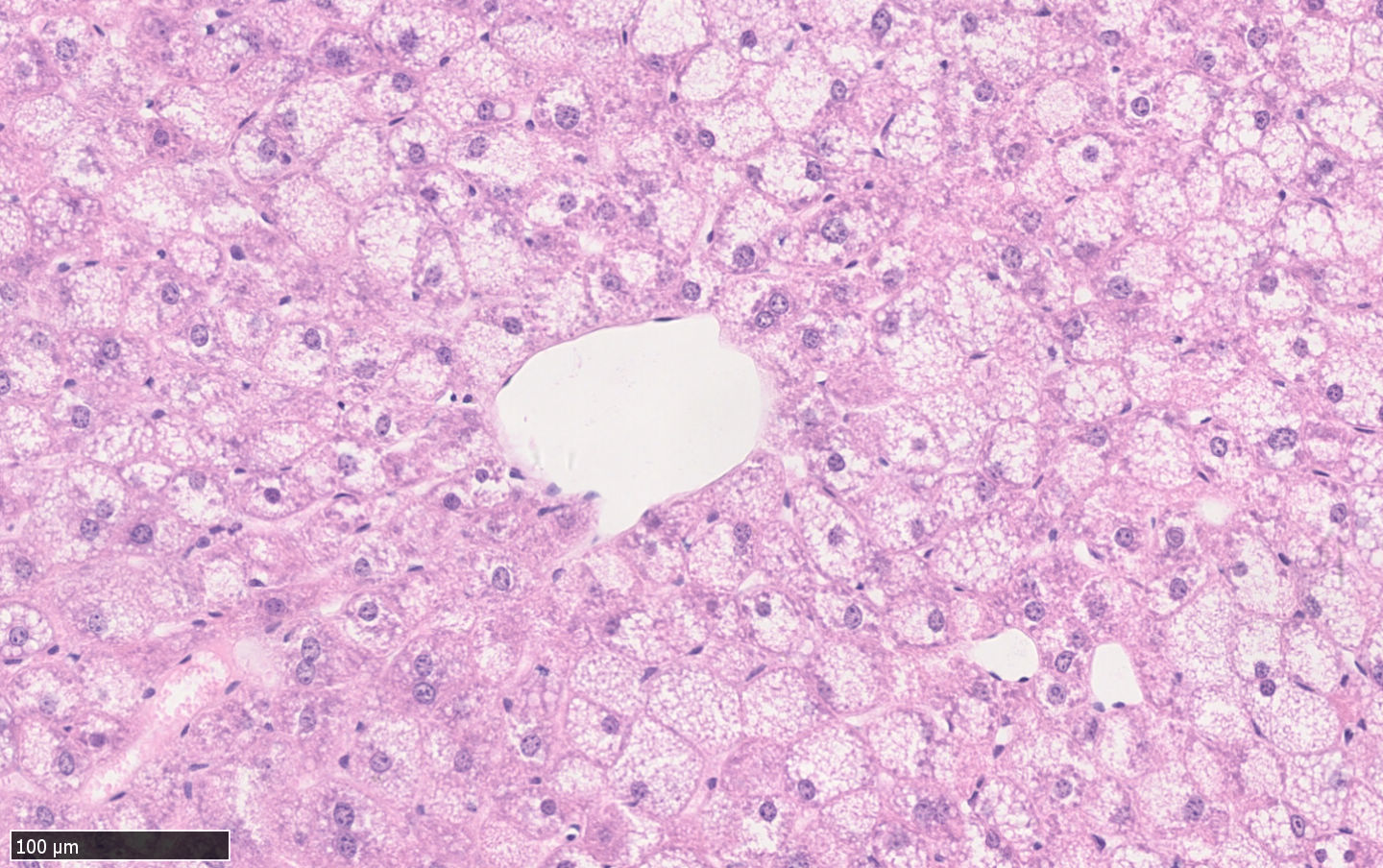

Supplement: Supplementary file 4 [file DataSheet9.ZIP › NASH SCORE-db(1)/db8,16/4.jpg]

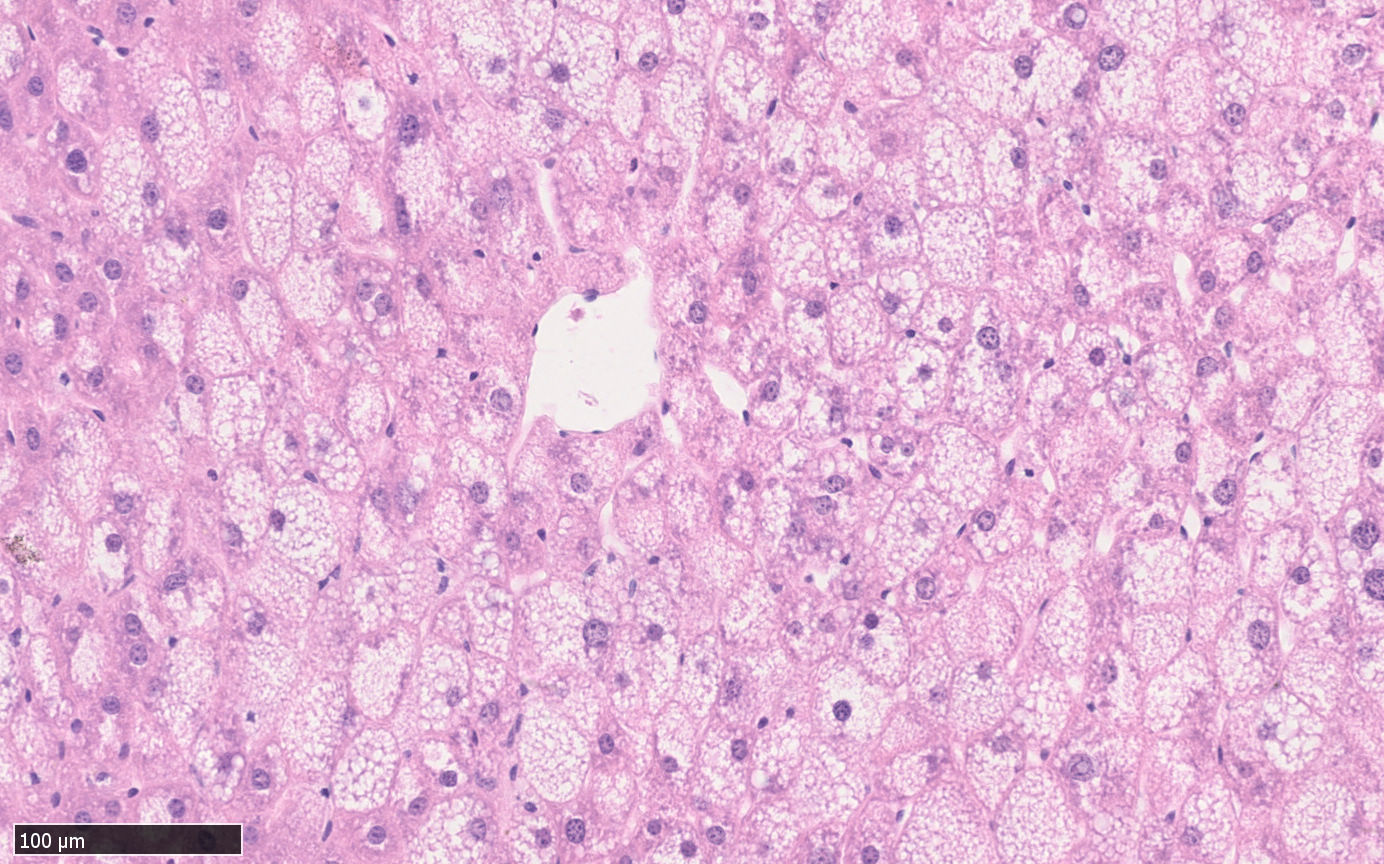

Supplement: Supplementary file 4 [file DataSheet9.ZIP › NASH SCORE-db(1)/db8,16/5.jpg]

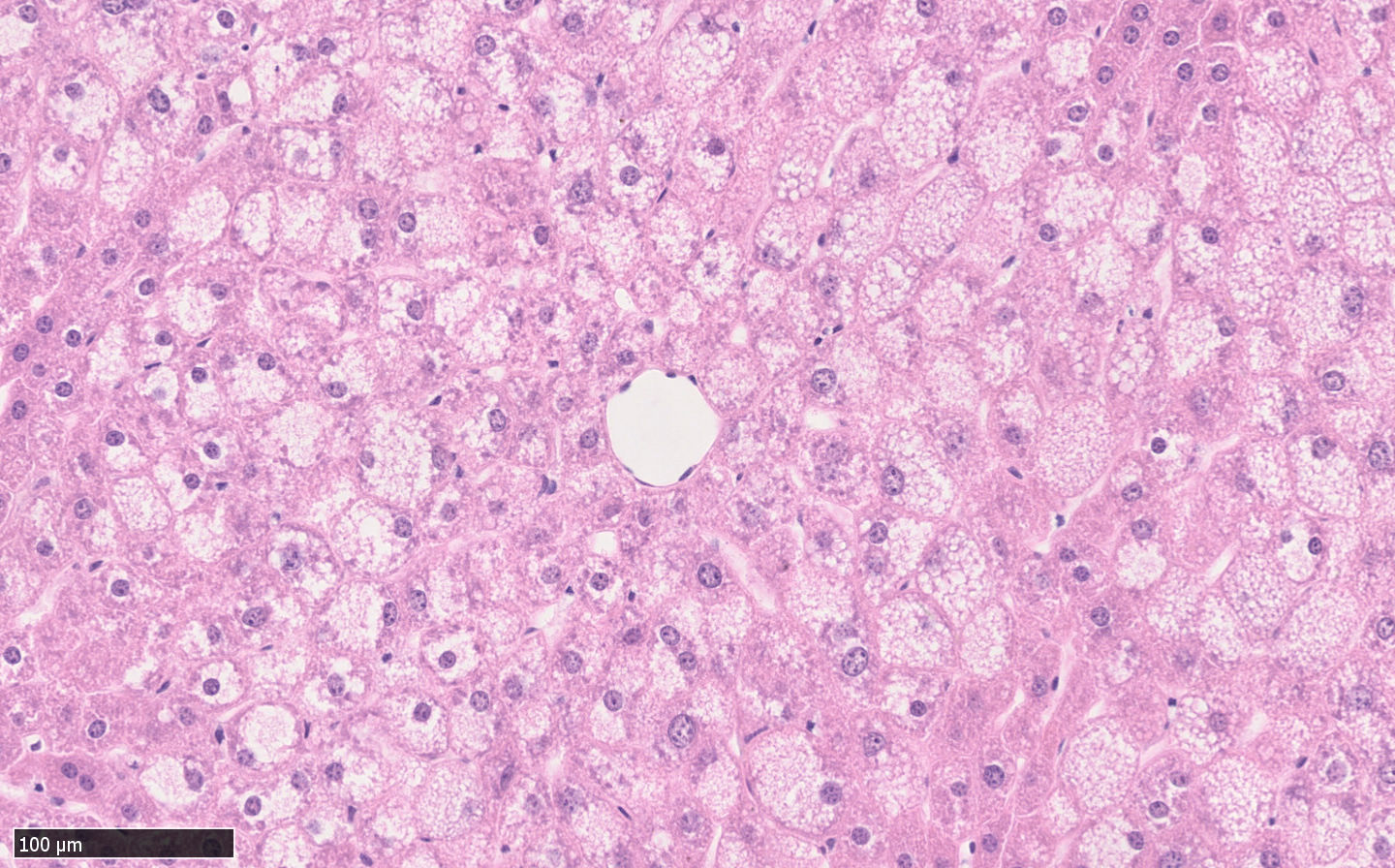

Supplement: Supplementary file 4 [file DataSheet9.ZIP › NASH SCORE-db(1)/db8,16/6.jpg]

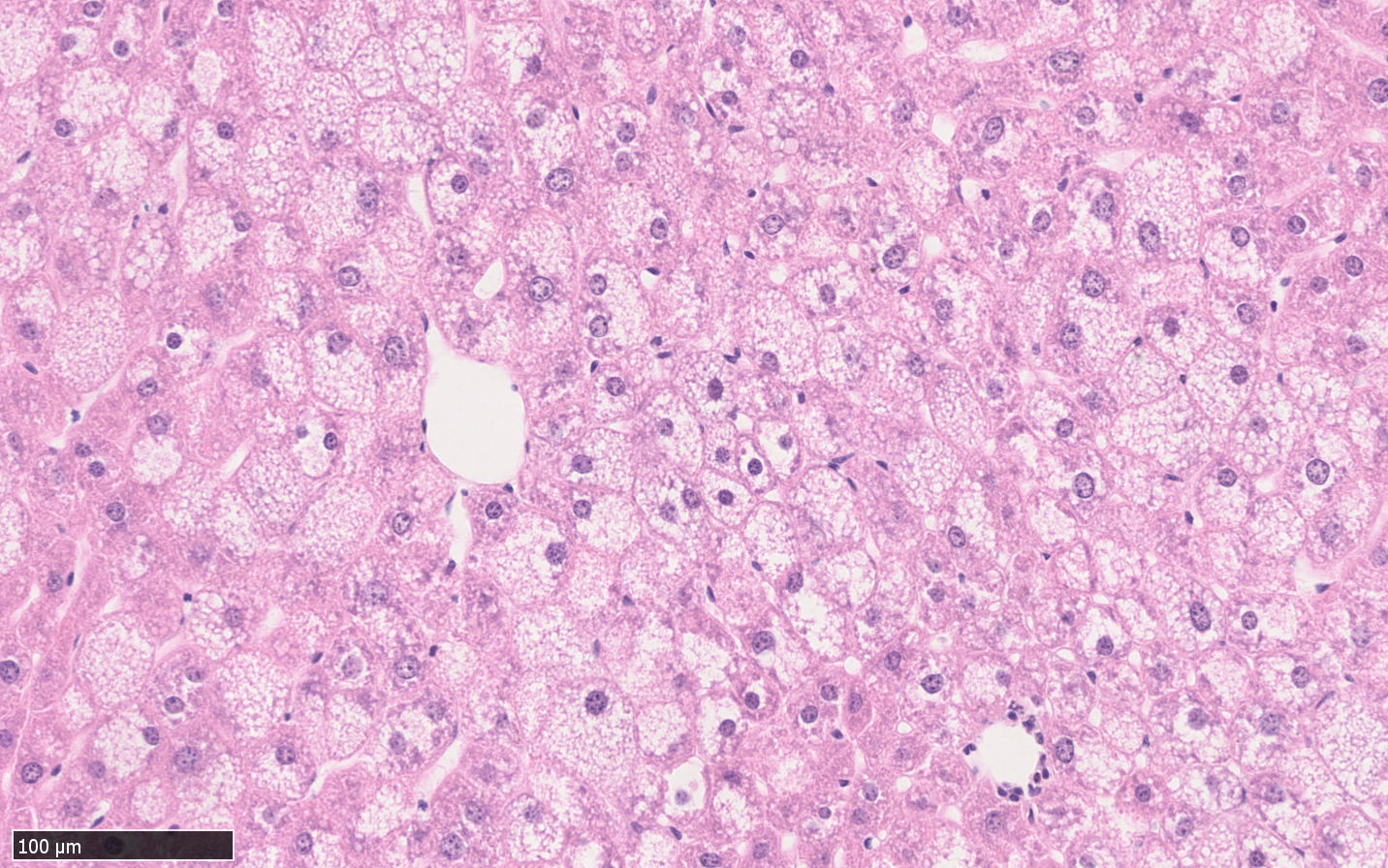

Supplement: Supplementary file 4 [file DataSheet9.ZIP › NASH SCORE-db(1)/db8,16/7.jpg]

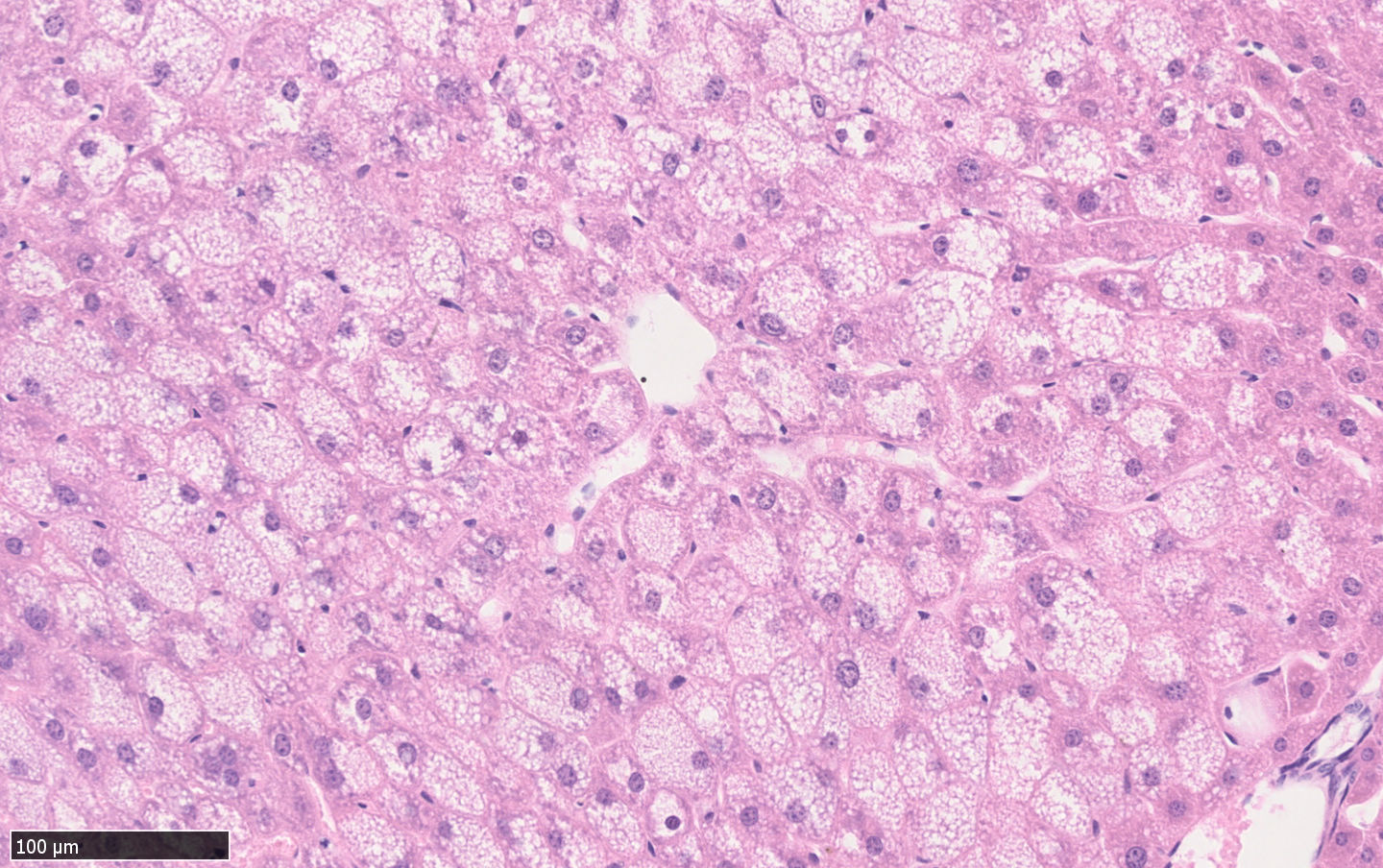

Supplement: Supplementary file 4 [file DataSheet9.ZIP › NASH SCORE-db(1)/db8,16/8.jpg]

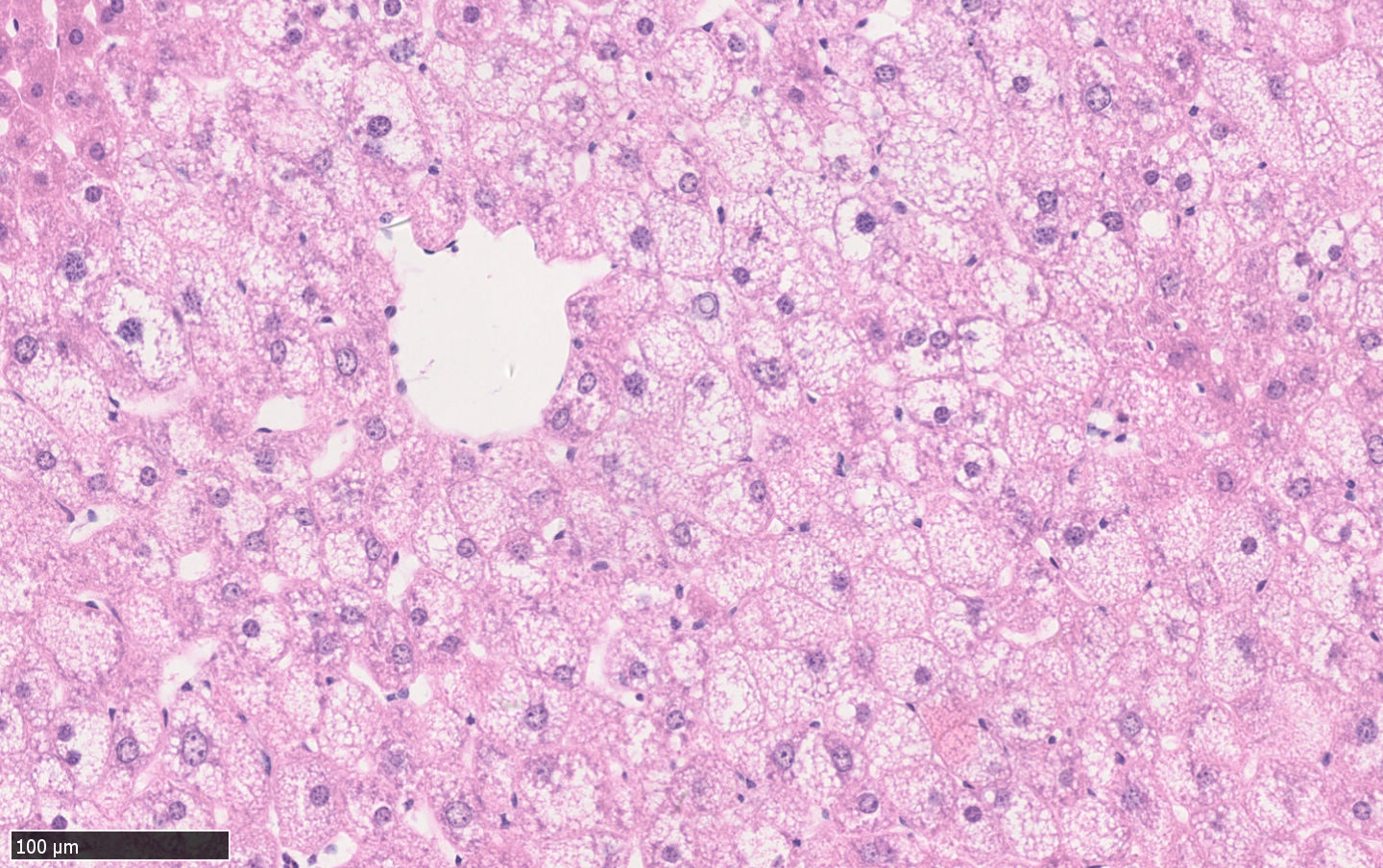

Supplement: Supplementary file 4 [file DataSheet9.ZIP › NASH SCORE-db(1)/db8,16/9.jpg]

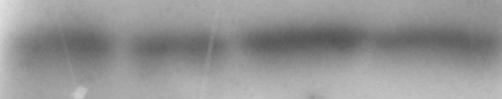

Supplement: Supplementary file 5 [file DataSheet4.ZIP › WB screenshot/1 3 TIMP1 (3)-2.tif]

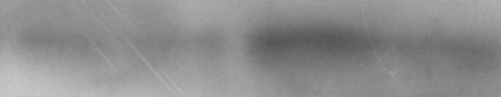

Supplement: Supplementary file 5 [file DataSheet4.ZIP › WB screenshot/1 3 TIMP1 (3).tif]

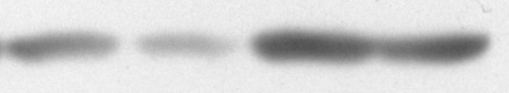

Supplement: Supplementary file 5 [file DataSheet4.ZIP › WB screenshot/1,2 ColIV MMP7 (3).tif]

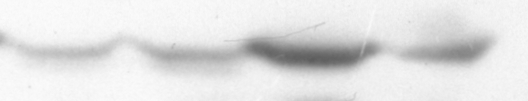

Supplement: Supplementary file 5 [file DataSheet4.ZIP › WB screenshot/1,2 PAI-1 (2).tif]

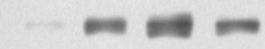

Supplement: Supplementary file 5 [file DataSheet4.ZIP › WB screenshot/3 ACC p-AKt2 (8).tif]

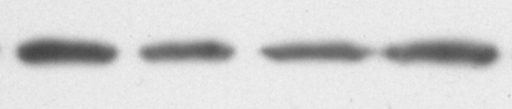

Supplement: Supplementary file 5 [file DataSheet4.ZIP › WB screenshot/3 FXR (1).tif]

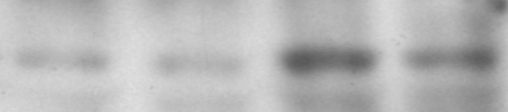

Supplement: Supplementary file 5 [file DataSheet4.ZIP › WB screenshot/3 liver MMP9 (2).tif]

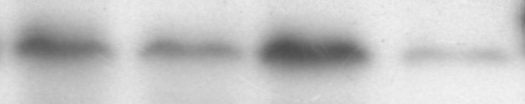

Supplement: Supplementary file 5 [file DataSheet4.ZIP › WB screenshot/3 p-p65 (2).tif]

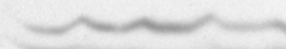

Supplement: Supplementary file 5 [file DataSheet4.ZIP › WB screenshot/a-SMA 1,2 3 (1).tif]

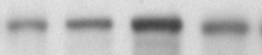

Supplement: Supplementary file 5 [file DataSheet4.ZIP › WB screenshot/ACC liver 1.tif]

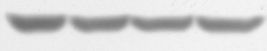

Supplement: Supplementary file 5 [file DataSheet4.ZIP › WB screenshot/b-actin (2)-1.tif]

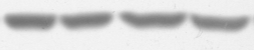

Supplement: Supplementary file 5 [file DataSheet4.ZIP › WB screenshot/b-actin (2)-2.tif]

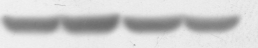

Supplement: Supplementary file 5 [file DataSheet4.ZIP › WB screenshot/b-actin (2)-3.tif]

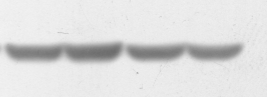

Supplement: Supplementary file 5 [file DataSheet4.ZIP › WB screenshot/b-actin 17-9-26.tif]

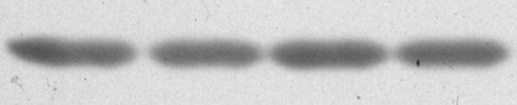

Supplement: Supplementary file 5 [file DataSheet4.ZIP › WB screenshot/b-actin 2.tif]

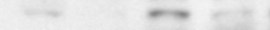

Supplement: Supplementary file 5 [file DataSheet4.ZIP › WB screenshot/COX-2 1,2 (1).tif]

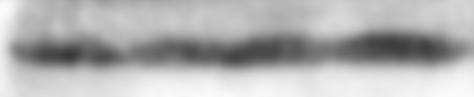

Supplement: Supplementary file 5 [file DataSheet4.ZIP › WB screenshot/CTGF (2).tif]

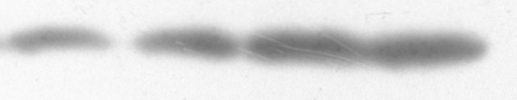

Supplement: Supplementary file 5 [file DataSheet4.ZIP › WB screenshot/CTGF.tif]

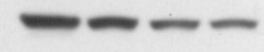

Supplement: Supplementary file 5 [file DataSheet4.ZIP › WB screenshot/CYP2E 3 pi CPT1a 1 2,3 (2).tif]

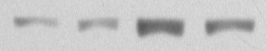

Supplement: Supplementary file 5 [file DataSheet4.ZIP › WB screenshot/FAS 1,2批 3批 (6).tif]

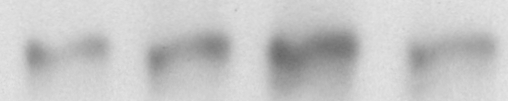

Supplement: Supplementary file 5 [file DataSheet4.ZIP › WB screenshot/FN p-IRS1 p-Gsk3b (8).tif]

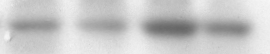

Supplement: Supplementary file 5 [file DataSheet4.ZIP › WB screenshot/glut1 1,2 3批,liver (7).tif]

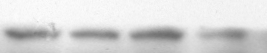

Supplement: Supplementary file 5 [file DataSheet4.ZIP › WB screenshot/glut2 1批.tif]

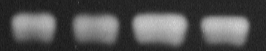

Supplement: Supplementary file 5 [file DataSheet4.ZIP › WB screenshot/InRb 3批 (1) - 副本.tif]
