# Supplementary figures and images for: Dapagliflozin protects against nonalcoholic steatohepatitis in db/db mice (part 3 of 3)
Source: Front Pharmacol. 2022 Aug 19;13:934136. doi: 10.3389/fphar.2022.934136 (PMC9437261; doi:10.3389/fphar.2022.934136)

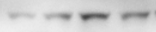

Supplement: Supplementary file 5 [file DataSheet4.ZIP › WB screenshot/liver 3pi 上FGFR4(95) 下CHREBP(93) (19).tif]

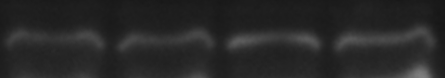

Supplement: Supplementary file 5 [file DataSheet4.ZIP › WB screenshot/liver 上1,2 p65,下3 p65 (1) - 副本.tif]

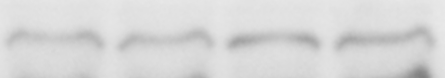

Supplement: Supplementary file 5 [file DataSheet4.ZIP › WB screenshot/liver 上1,2 p65,下3 p65 (1).tif]

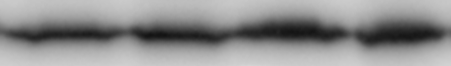

Supplement: Supplementary file 5 [file DataSheet4.ZIP › WB screenshot/LXRa 上1,2 下3批 (4).tif]

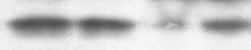

Supplement: Supplementary file 5 [file DataSheet4.ZIP › WB screenshot/SHP 1,2 3批 liver (6).tif]

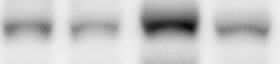

Supplement: Supplementary file 5 [file DataSheet4.ZIP › WB screenshot/SREBP1 liver (db+da) 上1,2批,下3批 (3).tif]

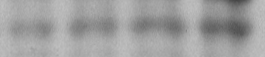

Supplement: Supplementary file 5 [file DataSheet4.ZIP › WB screenshot/TGF-b1 2016-4.tif]

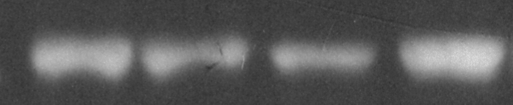

Supplement: Supplementary file 5 [file DataSheet4.ZIP › WB screenshot/模板 2.tif]

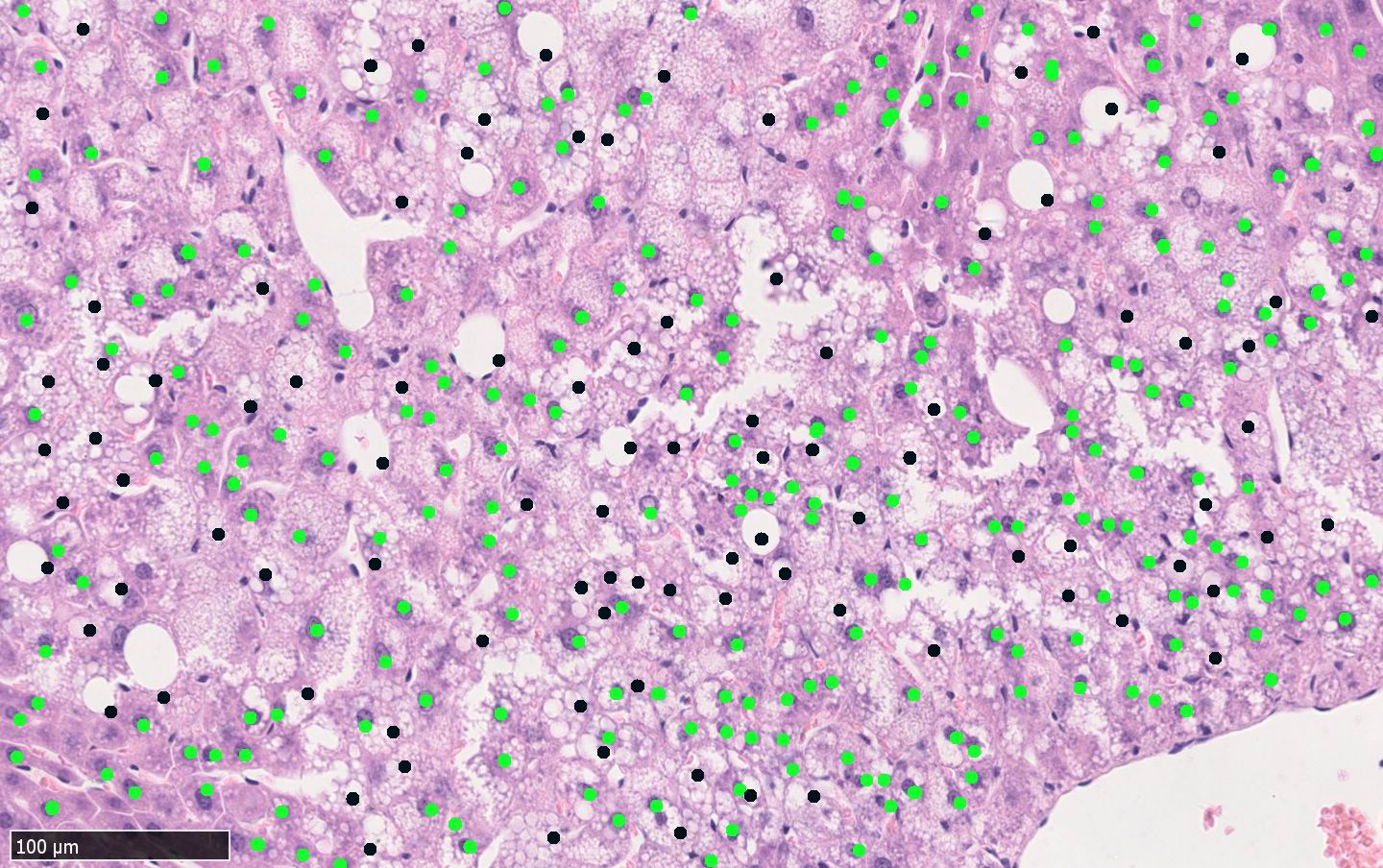

Supplement: Supplementary file 7 [file DataSheet10.ZIP › NASH SCORE-db(2)/db17,18 - 副本/1.jpg]

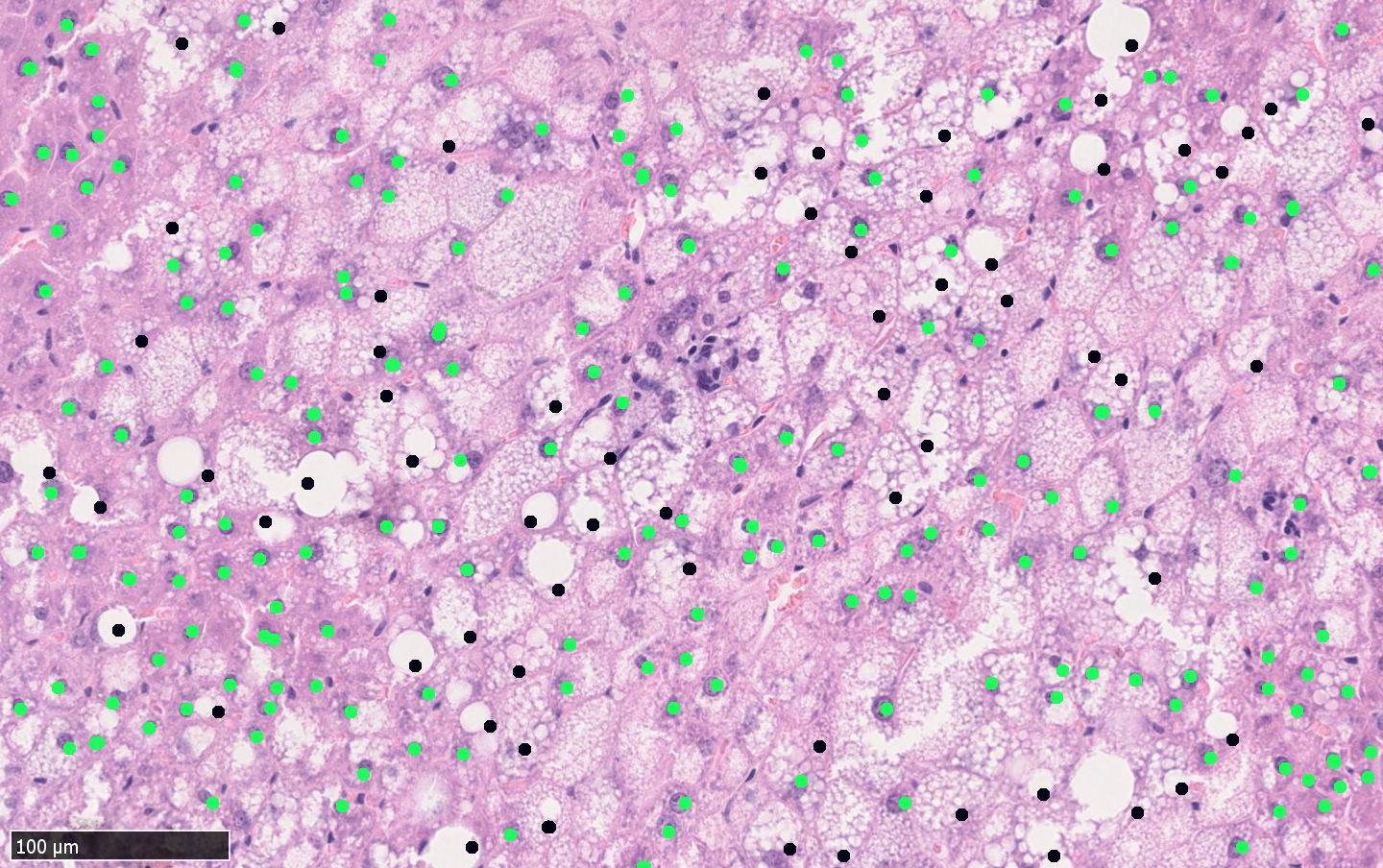

Supplement: Supplementary file 7 [file DataSheet10.ZIP › NASH SCORE-db(2)/db17,18 - 副本/10.jpg]

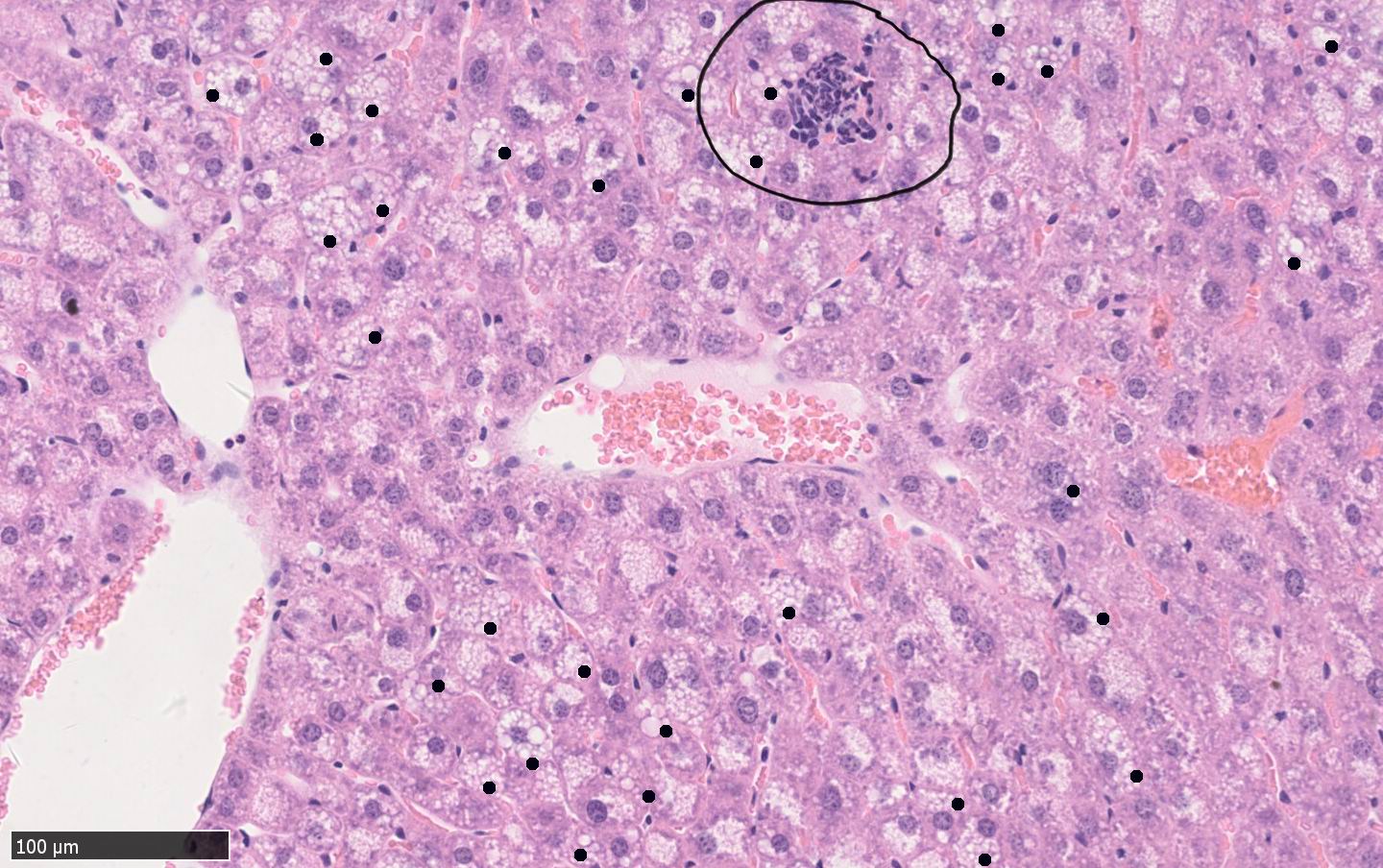

Supplement: Supplementary file 7 [file DataSheet10.ZIP › NASH SCORE-db(2)/db17,18 - 副本/11.jpg]

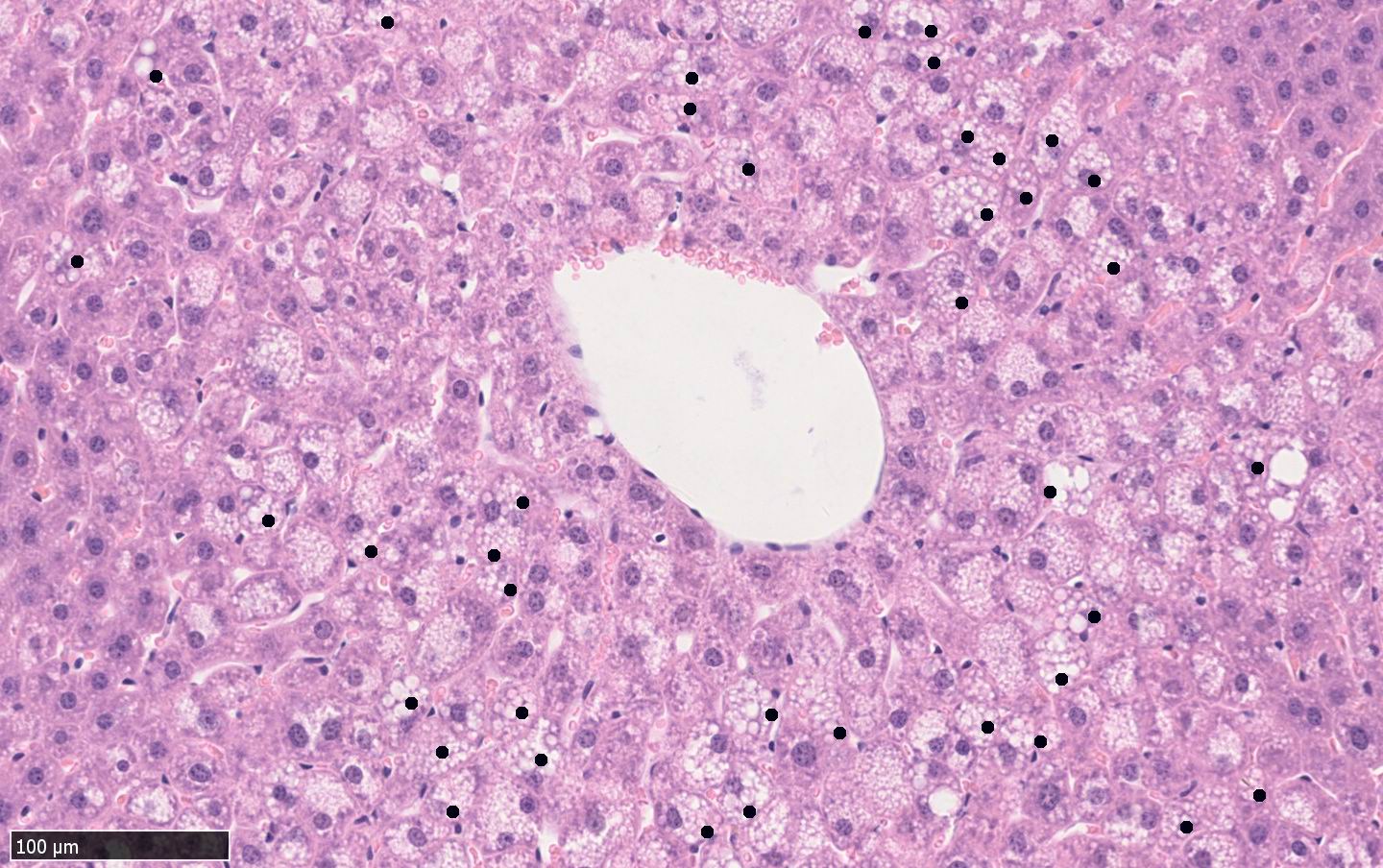

Supplement: Supplementary file 7 [file DataSheet10.ZIP › NASH SCORE-db(2)/db17,18 - 副本/12.jpg]

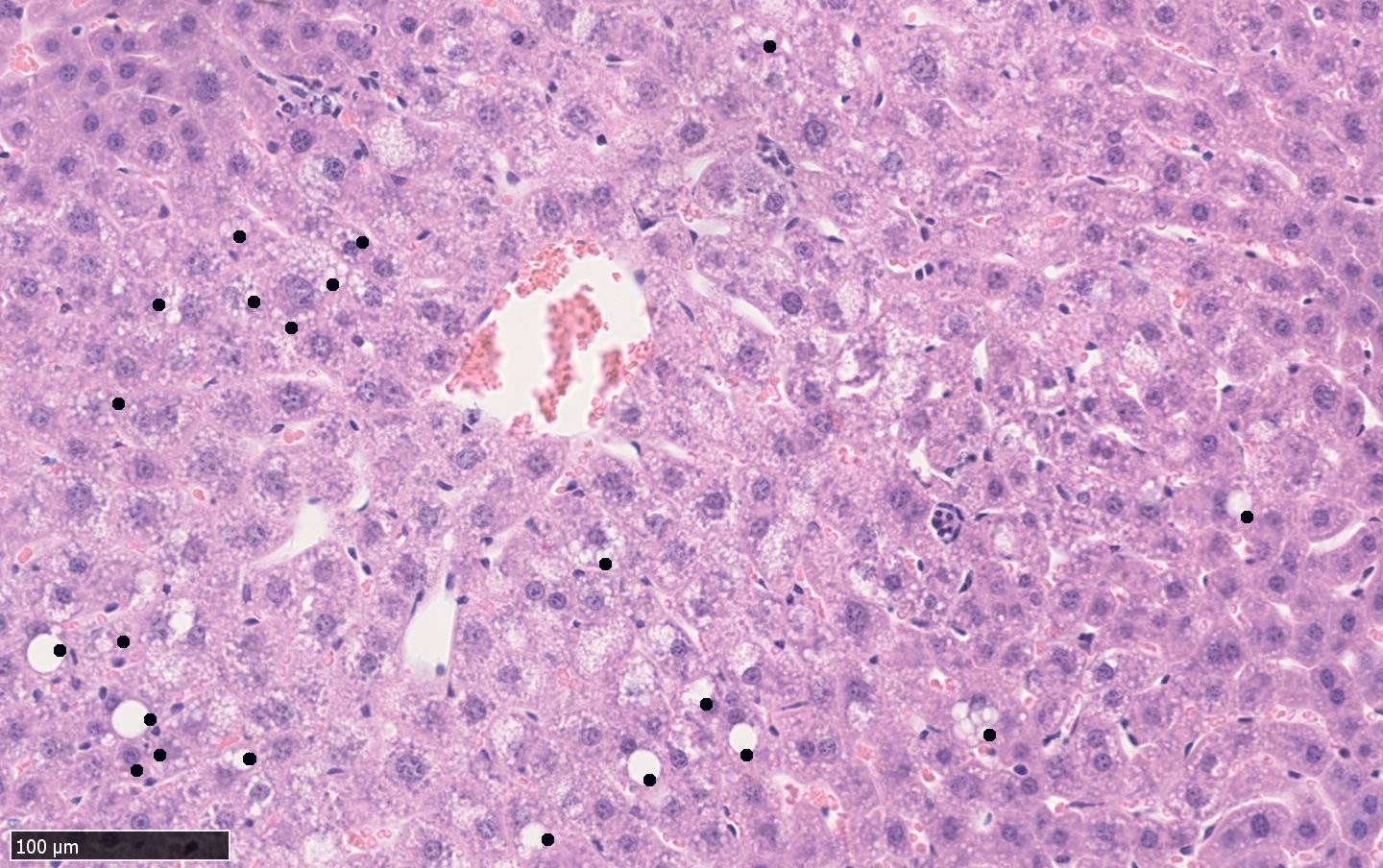

Supplement: Supplementary file 7 [file DataSheet10.ZIP › NASH SCORE-db(2)/db17,18 - 副本/13.jpg]

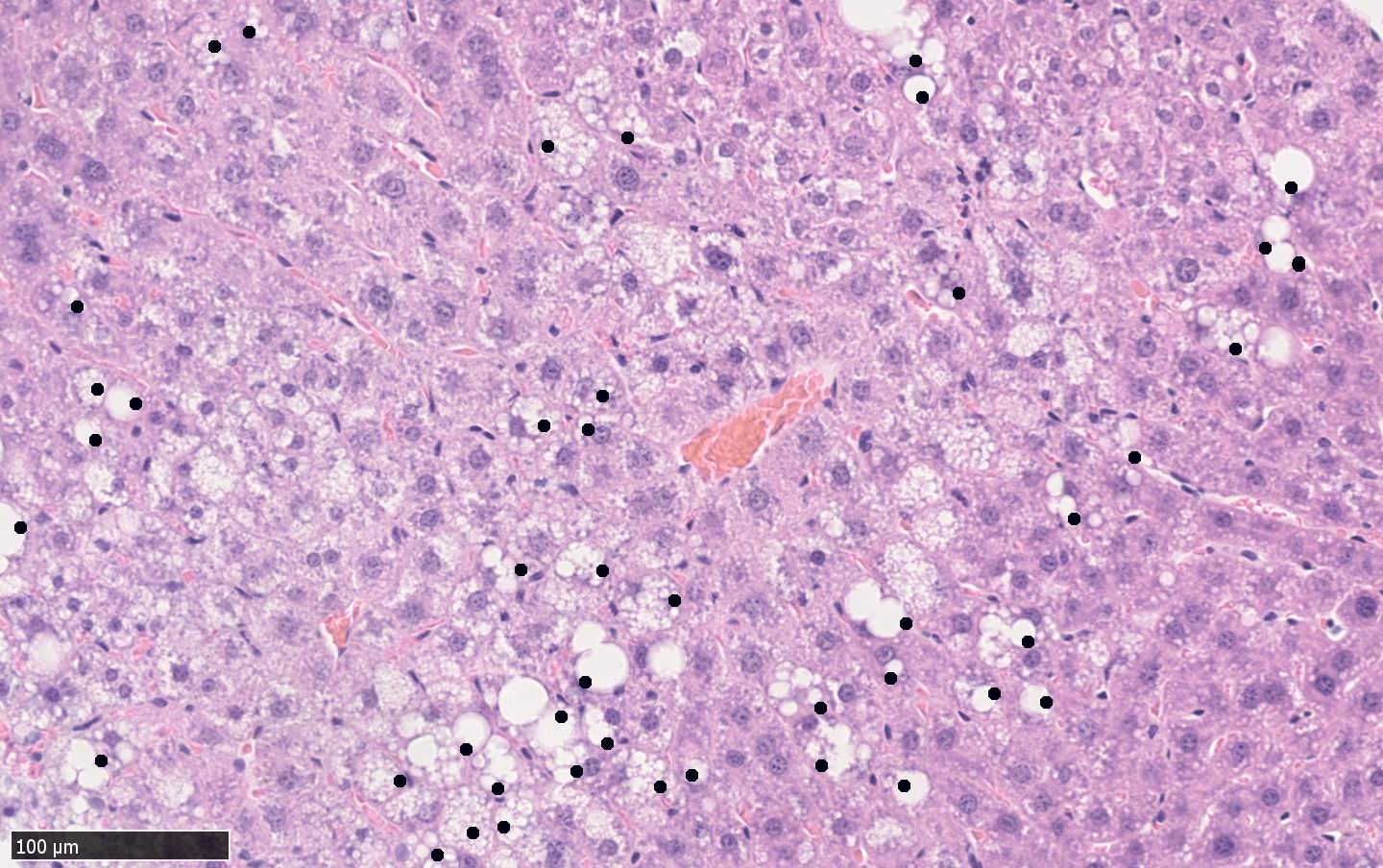

Supplement: Supplementary file 7 [file DataSheet10.ZIP › NASH SCORE-db(2)/db17,18 - 副本/14.jpg]

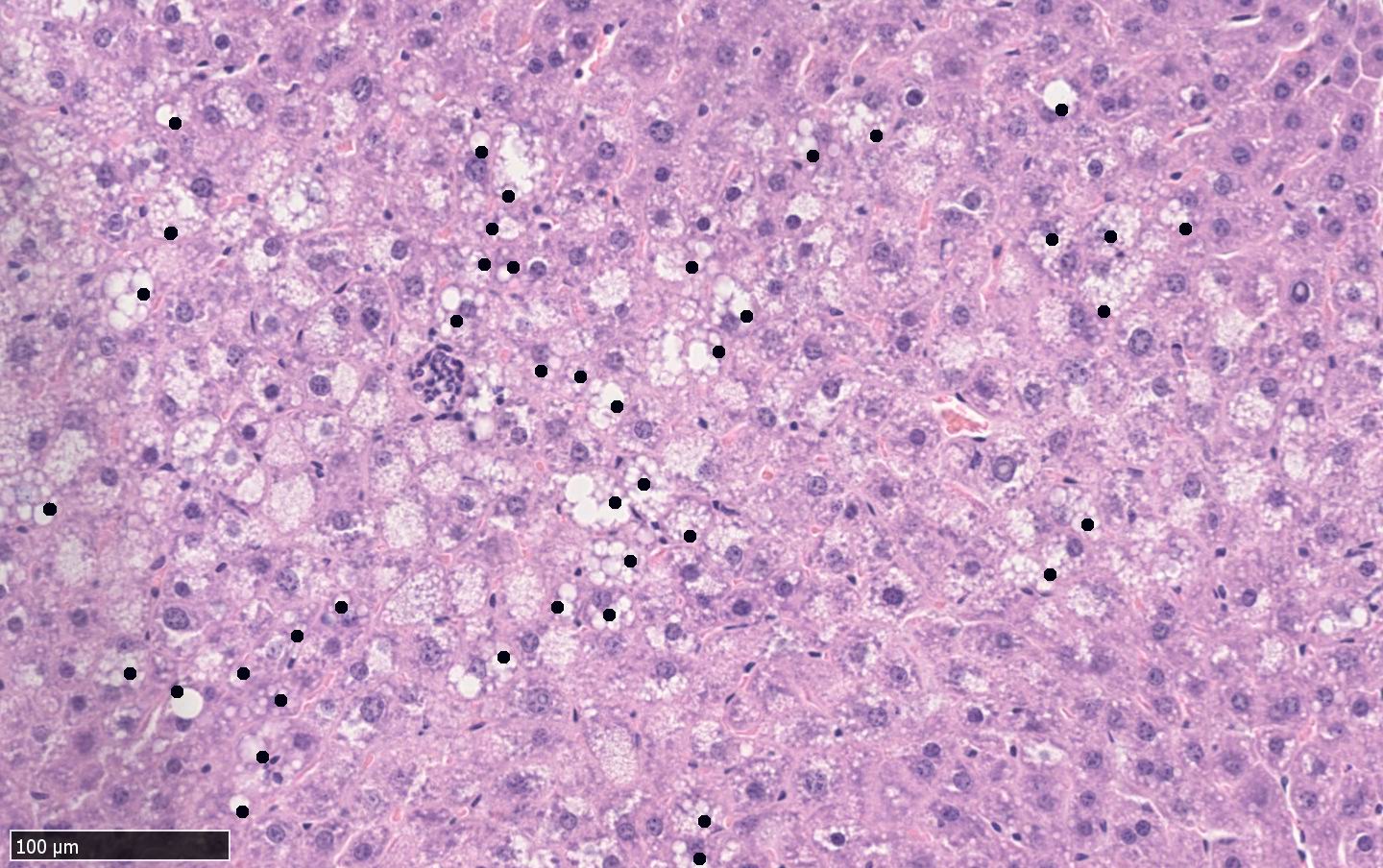

Supplement: Supplementary file 7 [file DataSheet10.ZIP › NASH SCORE-db(2)/db17,18 - 副本/15.jpg]

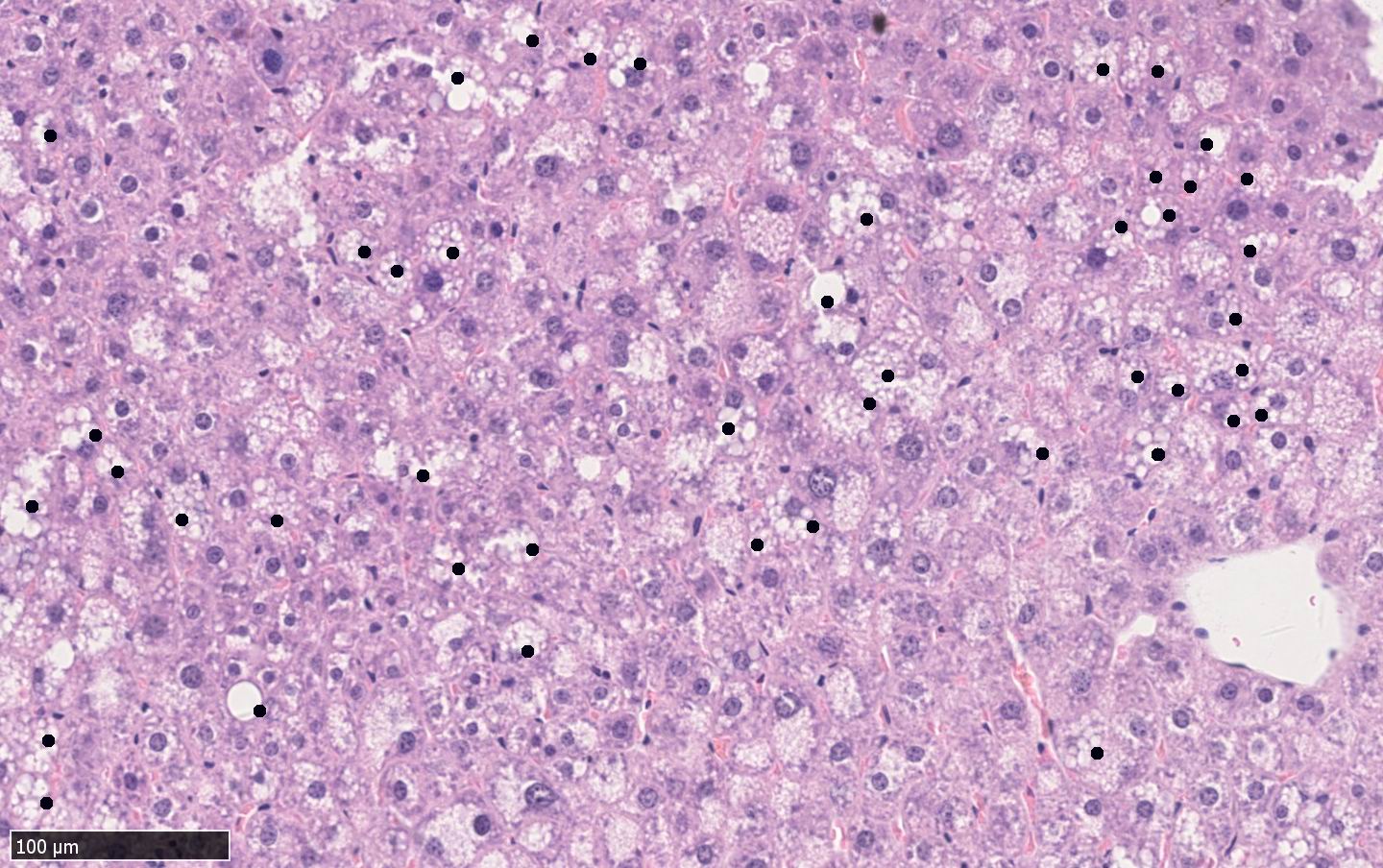

Supplement: Supplementary file 7 [file DataSheet10.ZIP › NASH SCORE-db(2)/db17,18 - 副本/16.jpg]

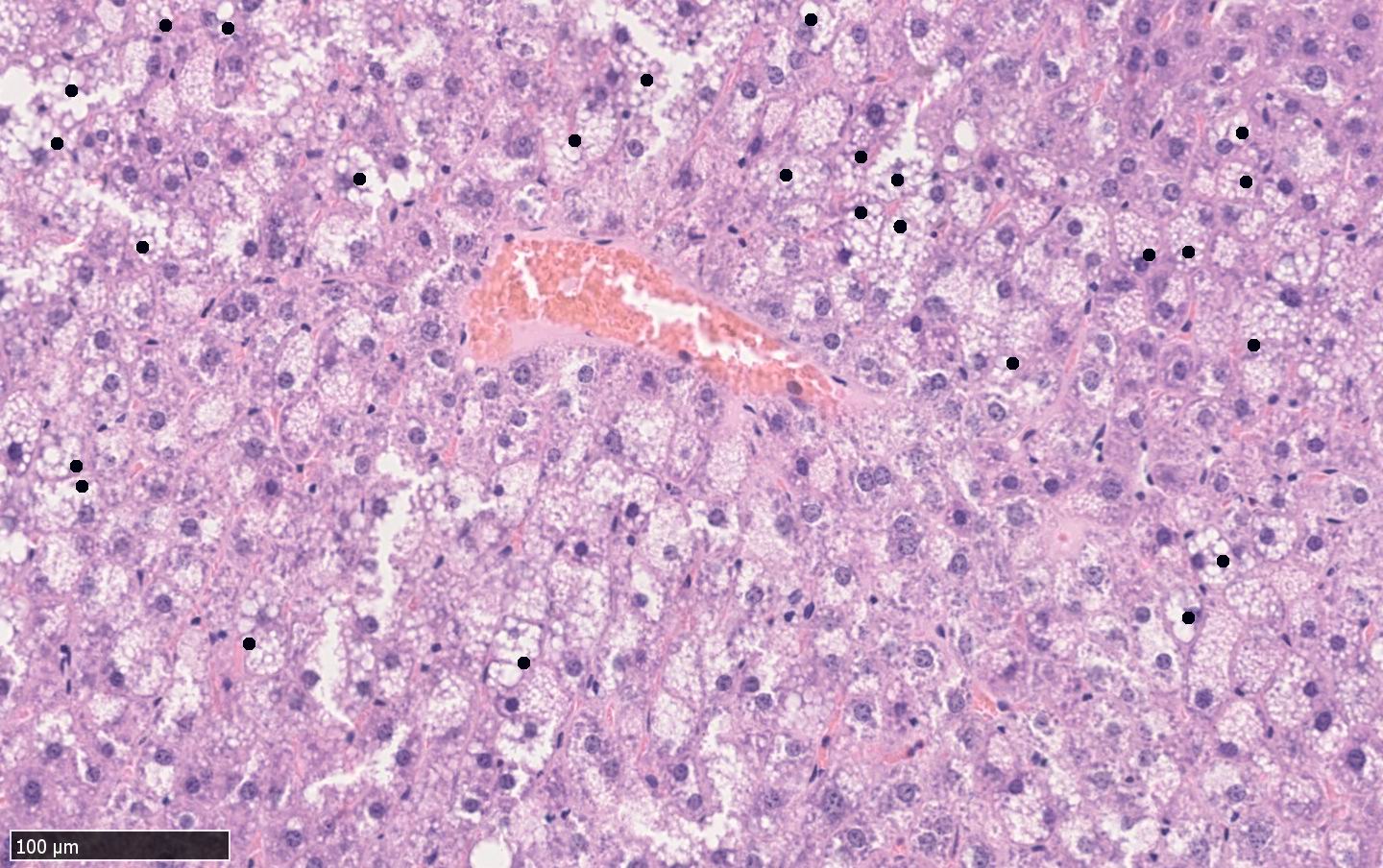

Supplement: Supplementary file 7 [file DataSheet10.ZIP › NASH SCORE-db(2)/db17,18 - 副本/17.jpg]

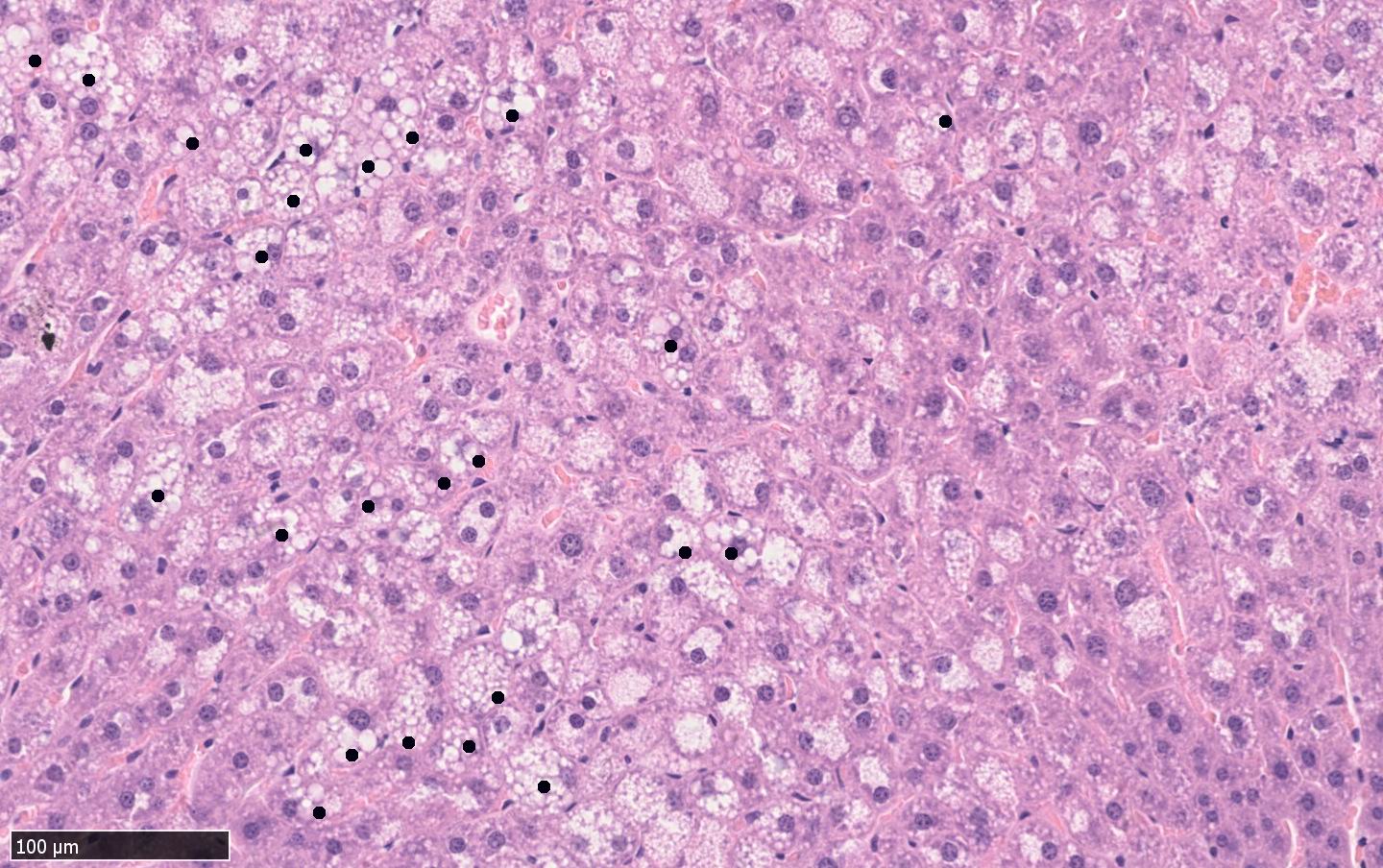

Supplement: Supplementary file 7 [file DataSheet10.ZIP › NASH SCORE-db(2)/db17,18 - 副本/18.jpg]

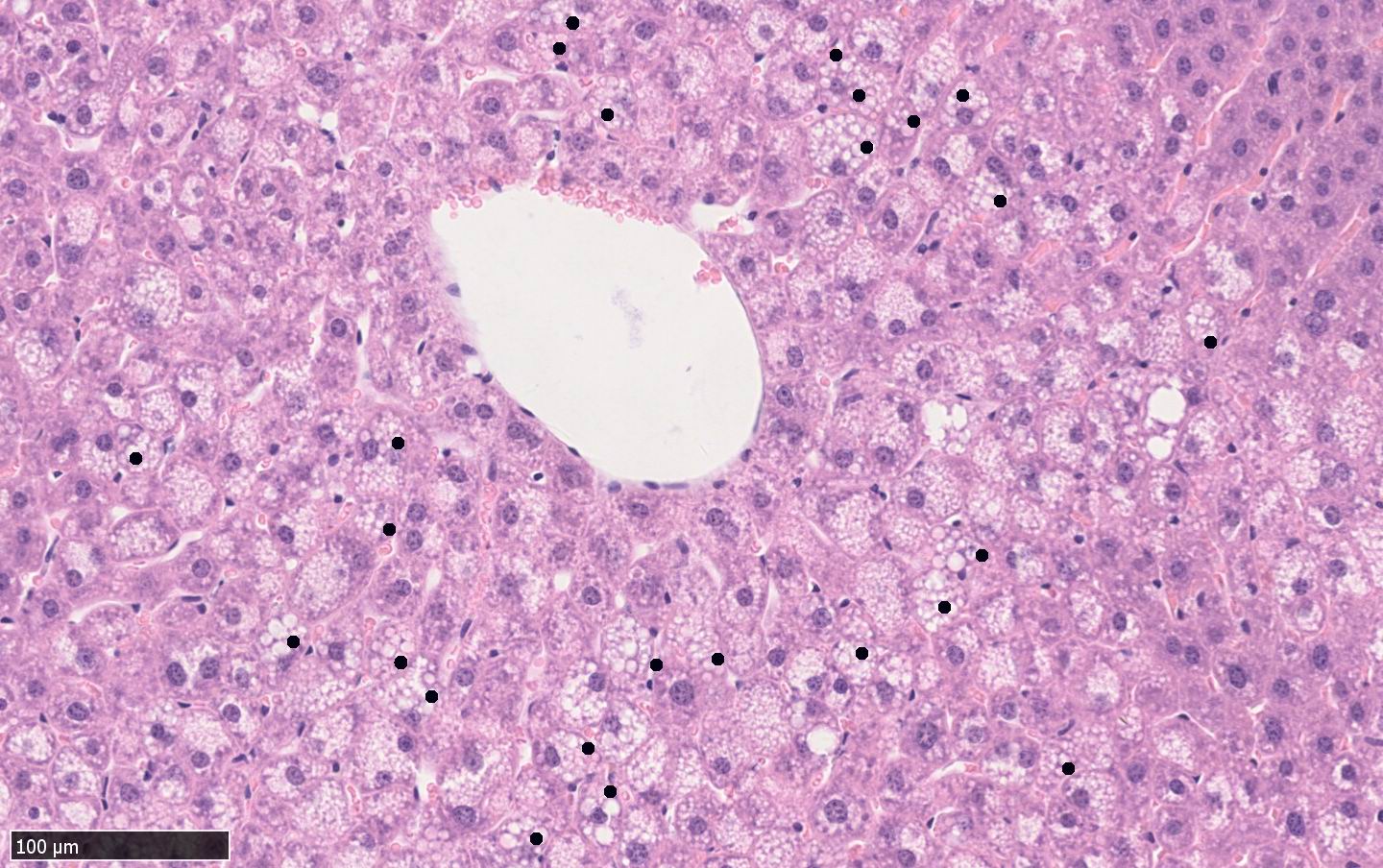

Supplement: Supplementary file 7 [file DataSheet10.ZIP › NASH SCORE-db(2)/db17,18 - 副本/19.jpg]

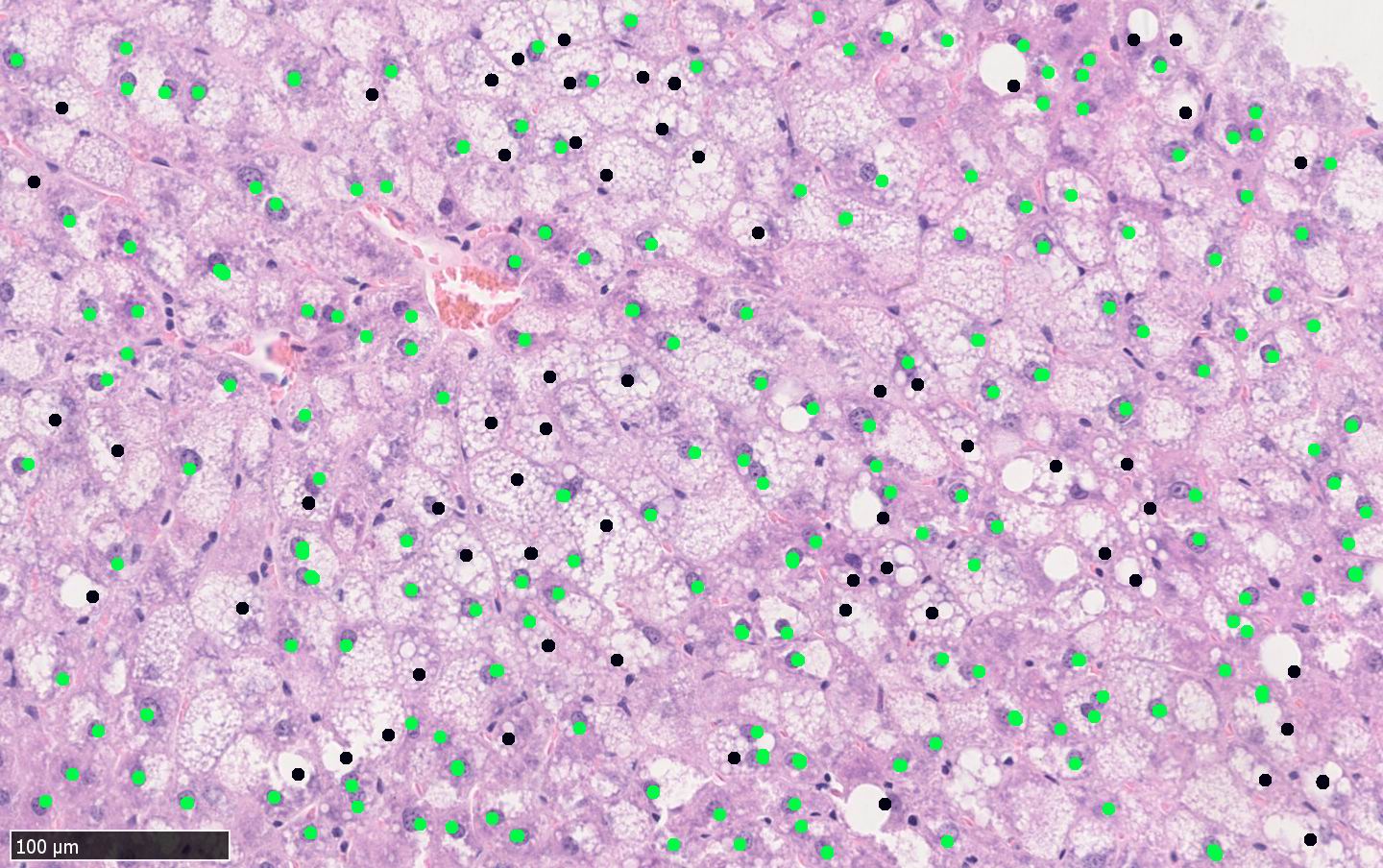

Supplement: Supplementary file 7 [file DataSheet10.ZIP › NASH SCORE-db(2)/db17,18 - 副本/2.jpg]

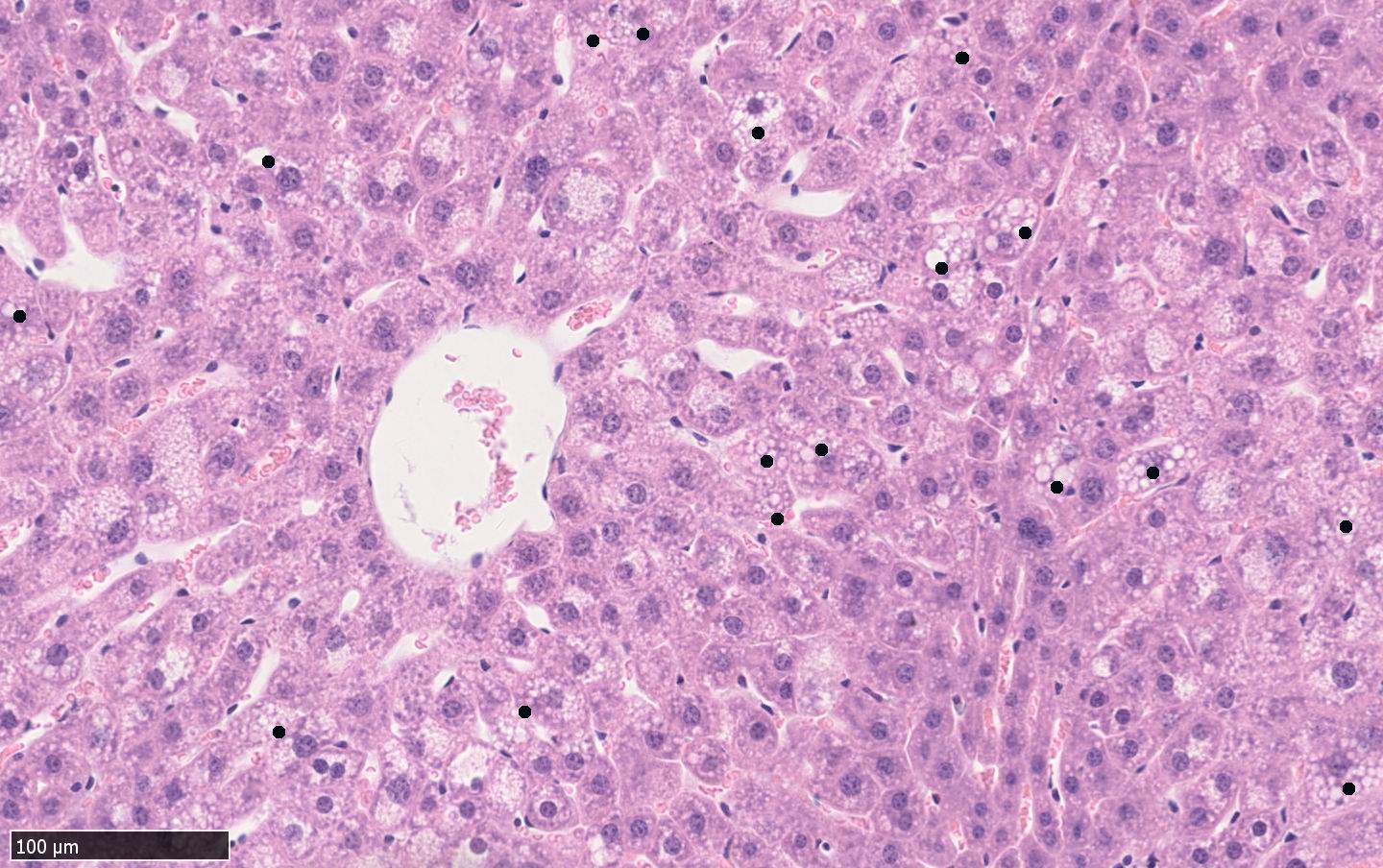

Supplement: Supplementary file 7 [file DataSheet10.ZIP › NASH SCORE-db(2)/db17,18 - 副本/20.jpg]

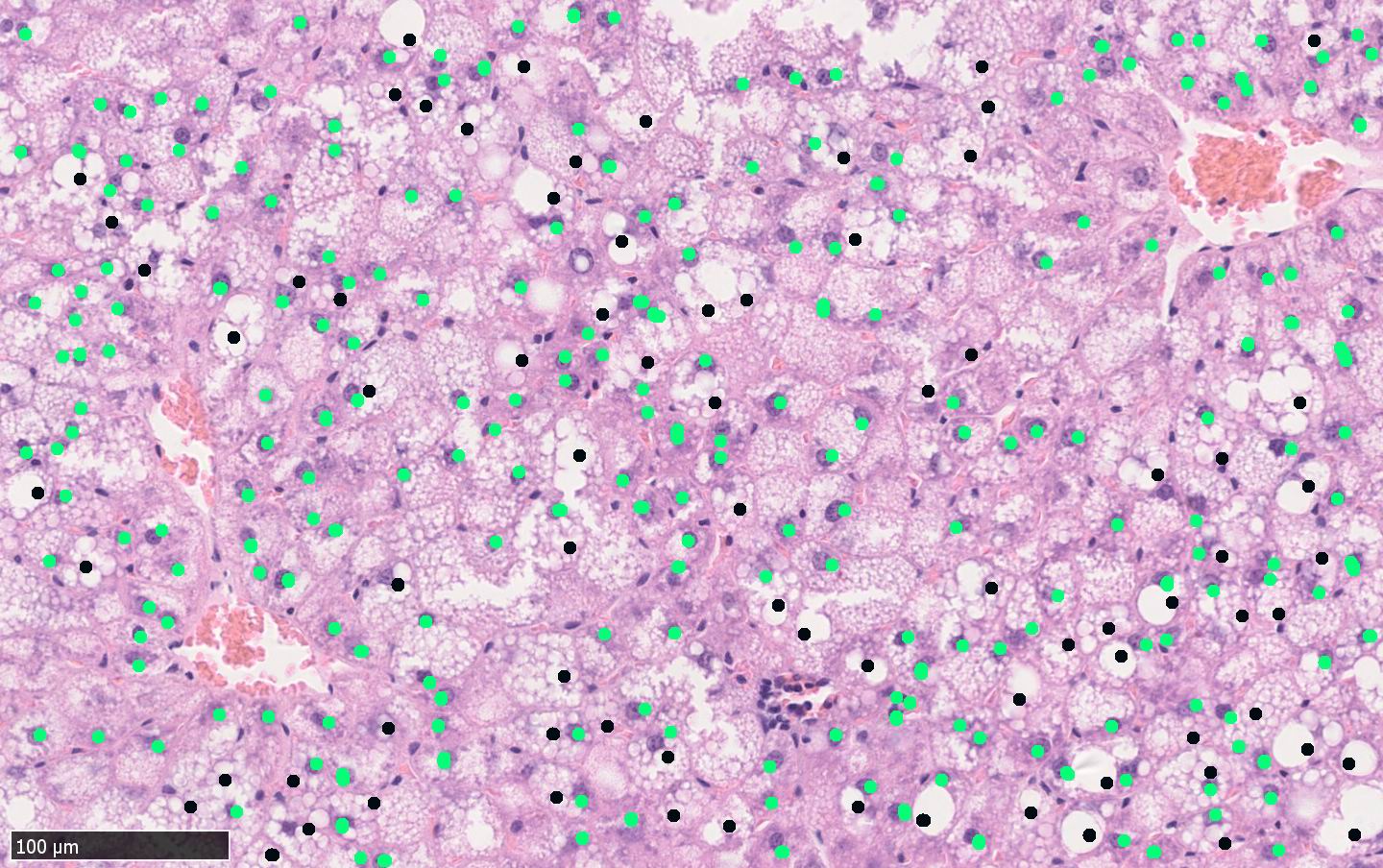

Supplement: Supplementary file 7 [file DataSheet10.ZIP › NASH SCORE-db(2)/db17,18 - 副本/7.jpg]

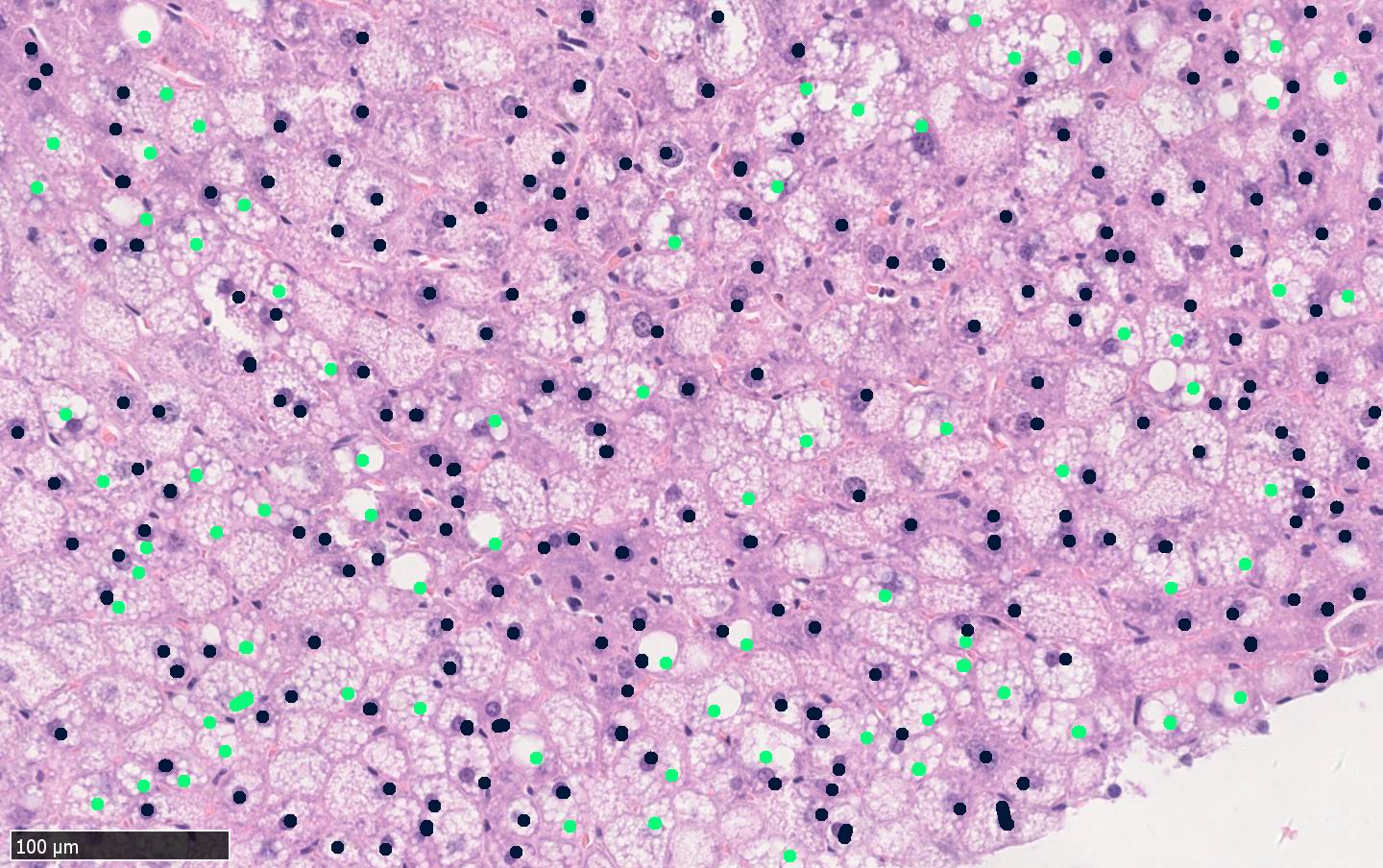

Supplement: Supplementary file 7 [file DataSheet10.ZIP › NASH SCORE-db(2)/db17,18 - 副本/8.jpg]

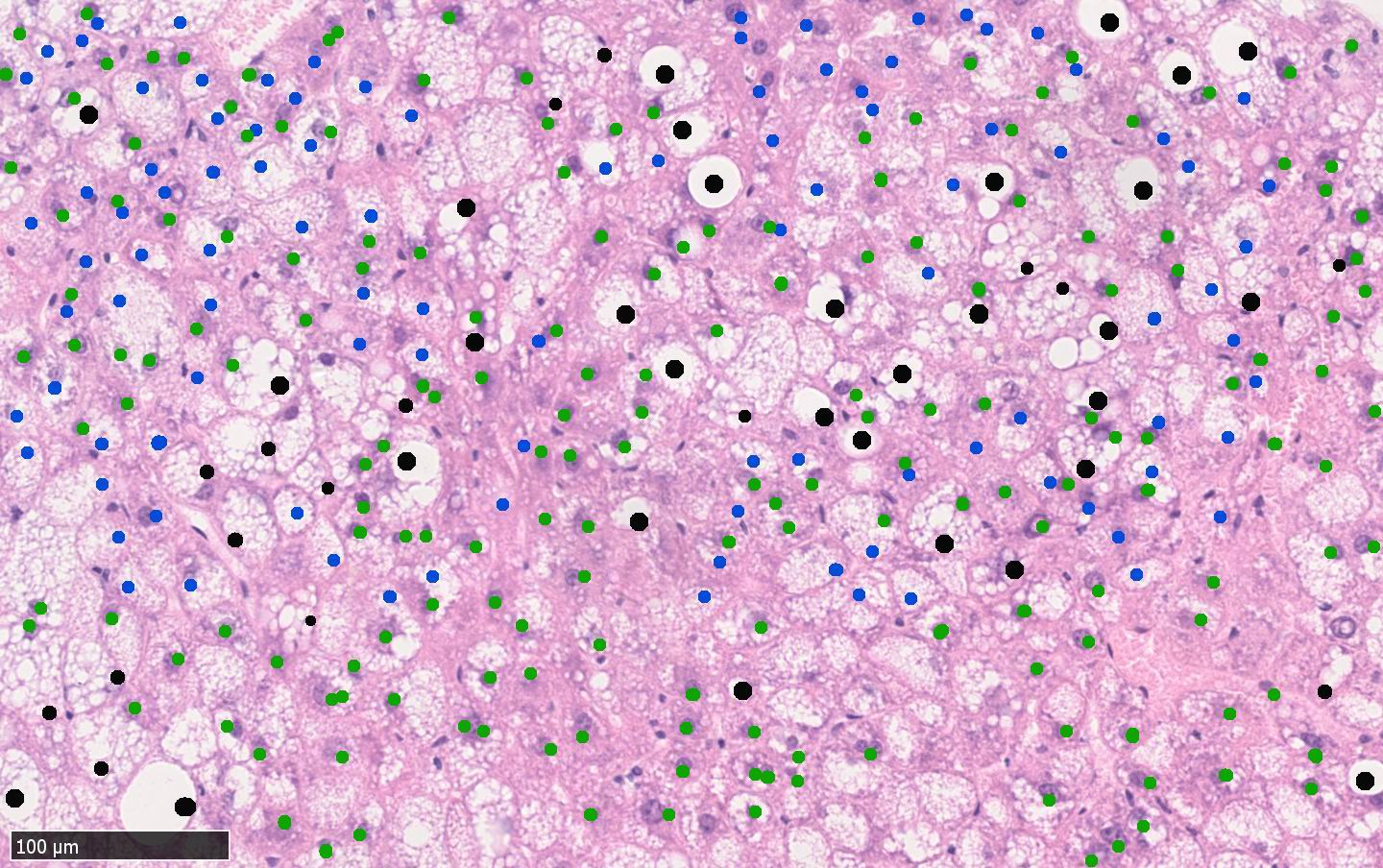

Supplement: Supplementary file 7 [file DataSheet10.ZIP › NASH SCORE-db(2)/db8,16 - 副本/1.jpg]

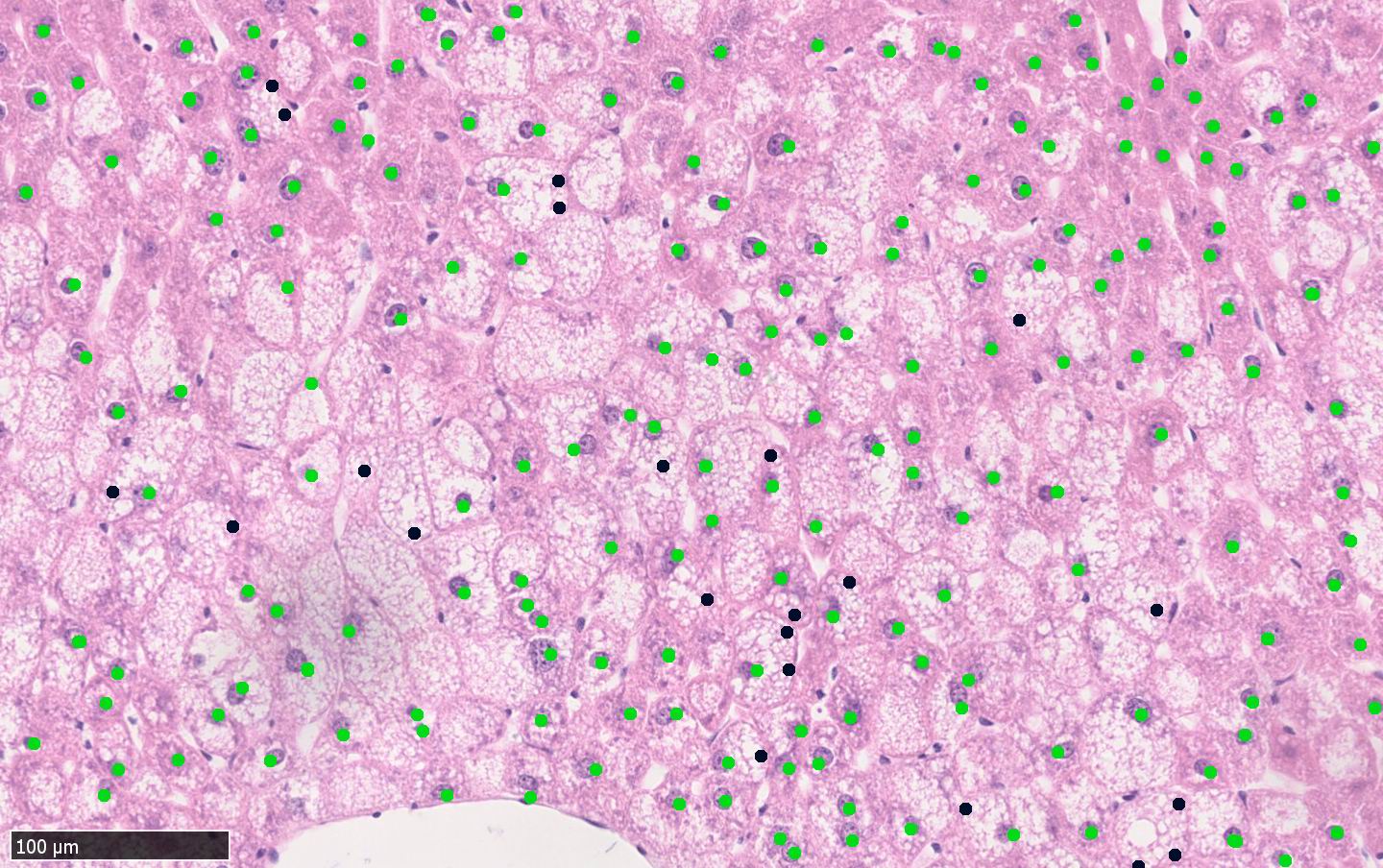

Supplement: Supplementary file 7 [file DataSheet10.ZIP › NASH SCORE-db(2)/db8,16 - 副本/10.jpg]

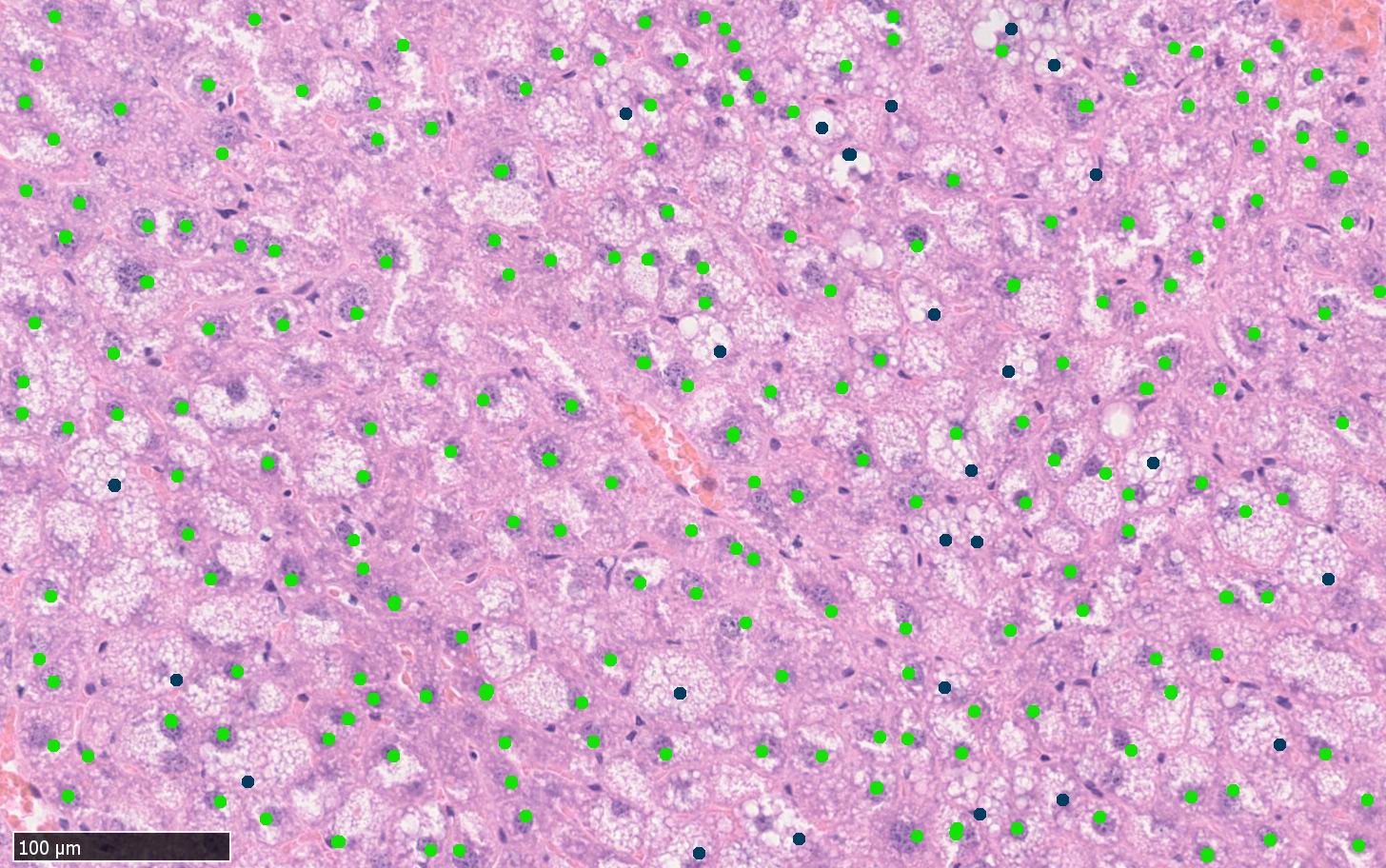

Supplement: Supplementary file 7 [file DataSheet10.ZIP › NASH SCORE-db(2)/db8,16 - 副本/11.jpg]

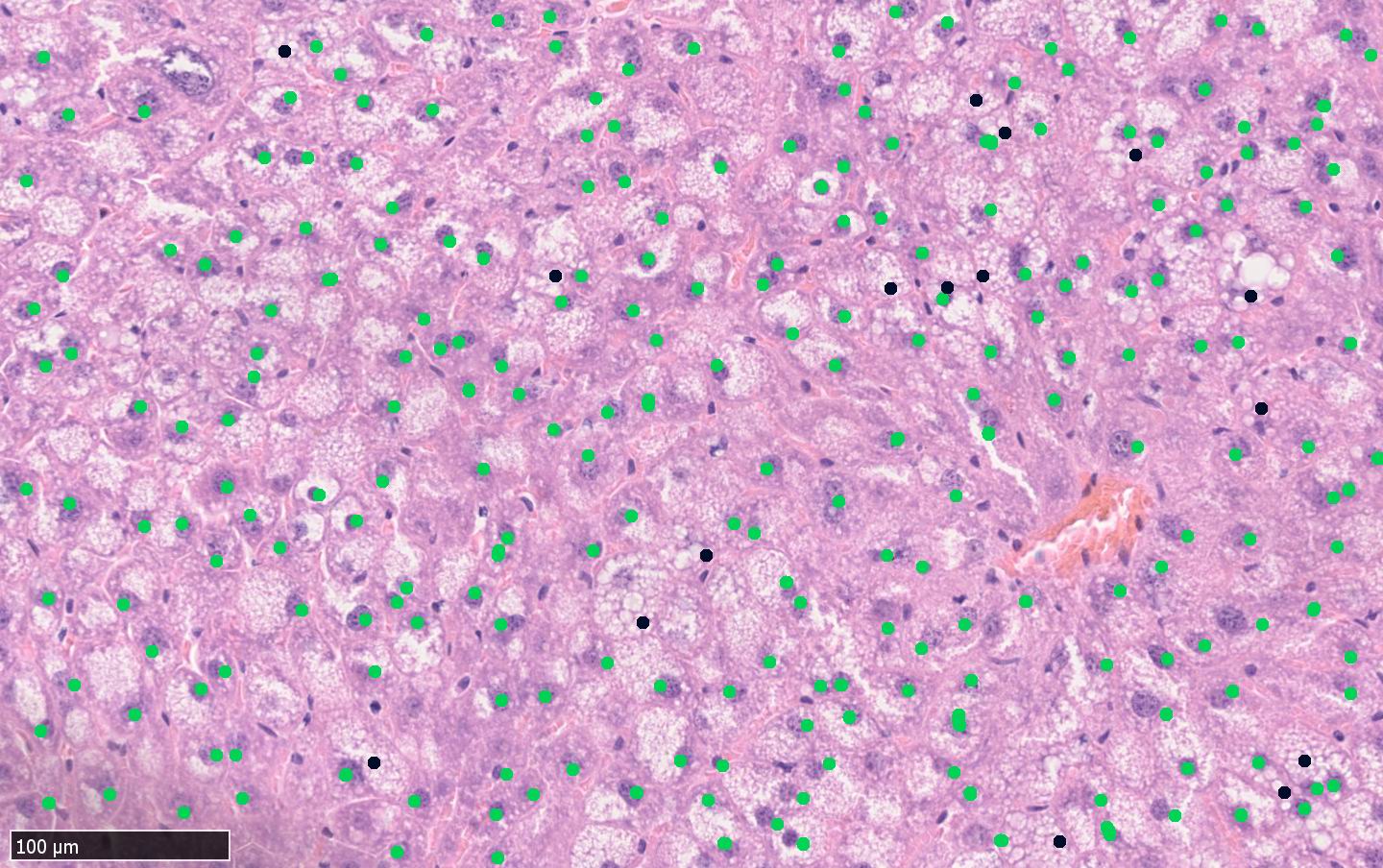

Supplement: Supplementary file 7 [file DataSheet10.ZIP › NASH SCORE-db(2)/db8,16 - 副本/12.jpg]

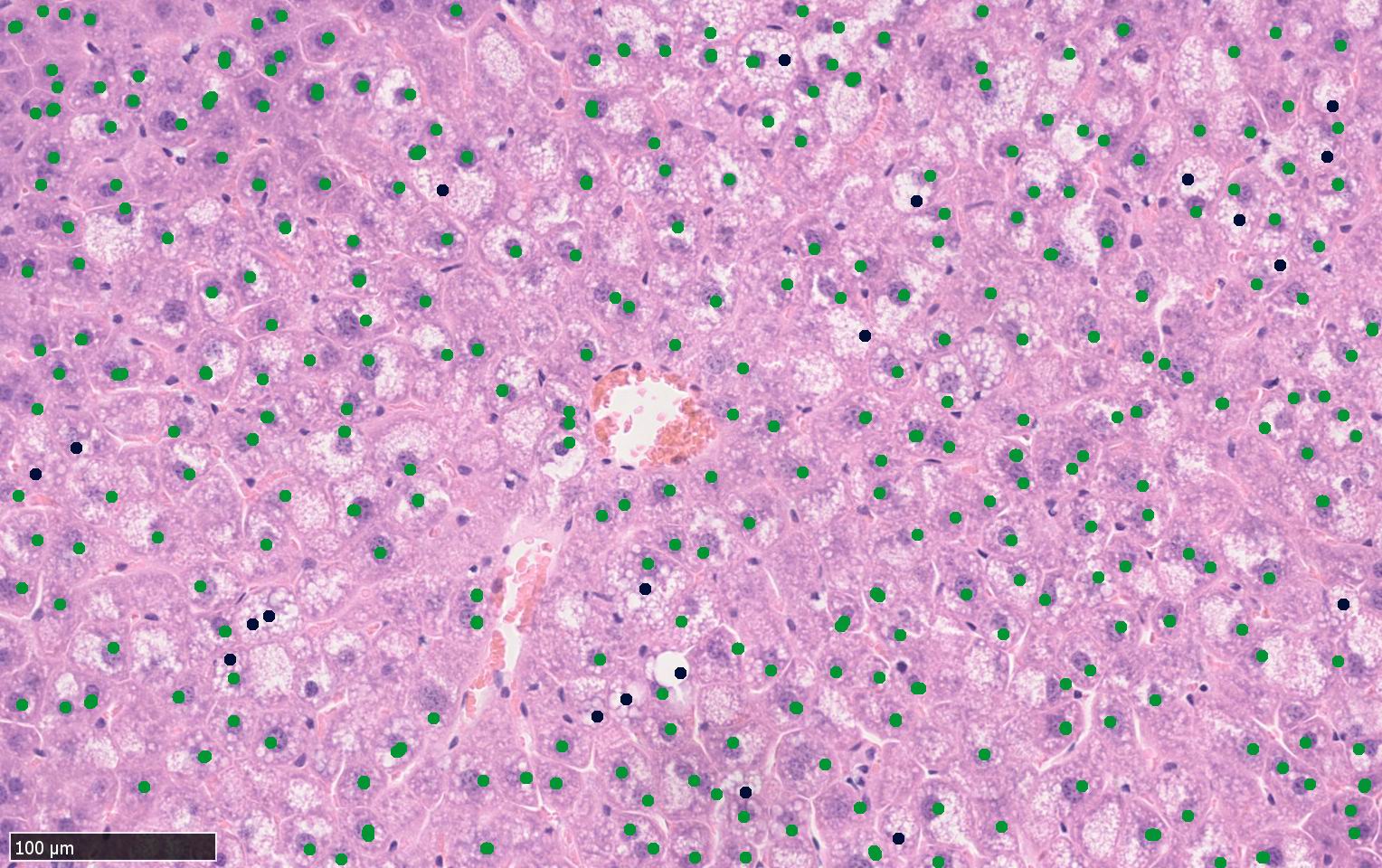

Supplement: Supplementary file 7 [file DataSheet10.ZIP › NASH SCORE-db(2)/db8,16 - 副本/13.jpg]

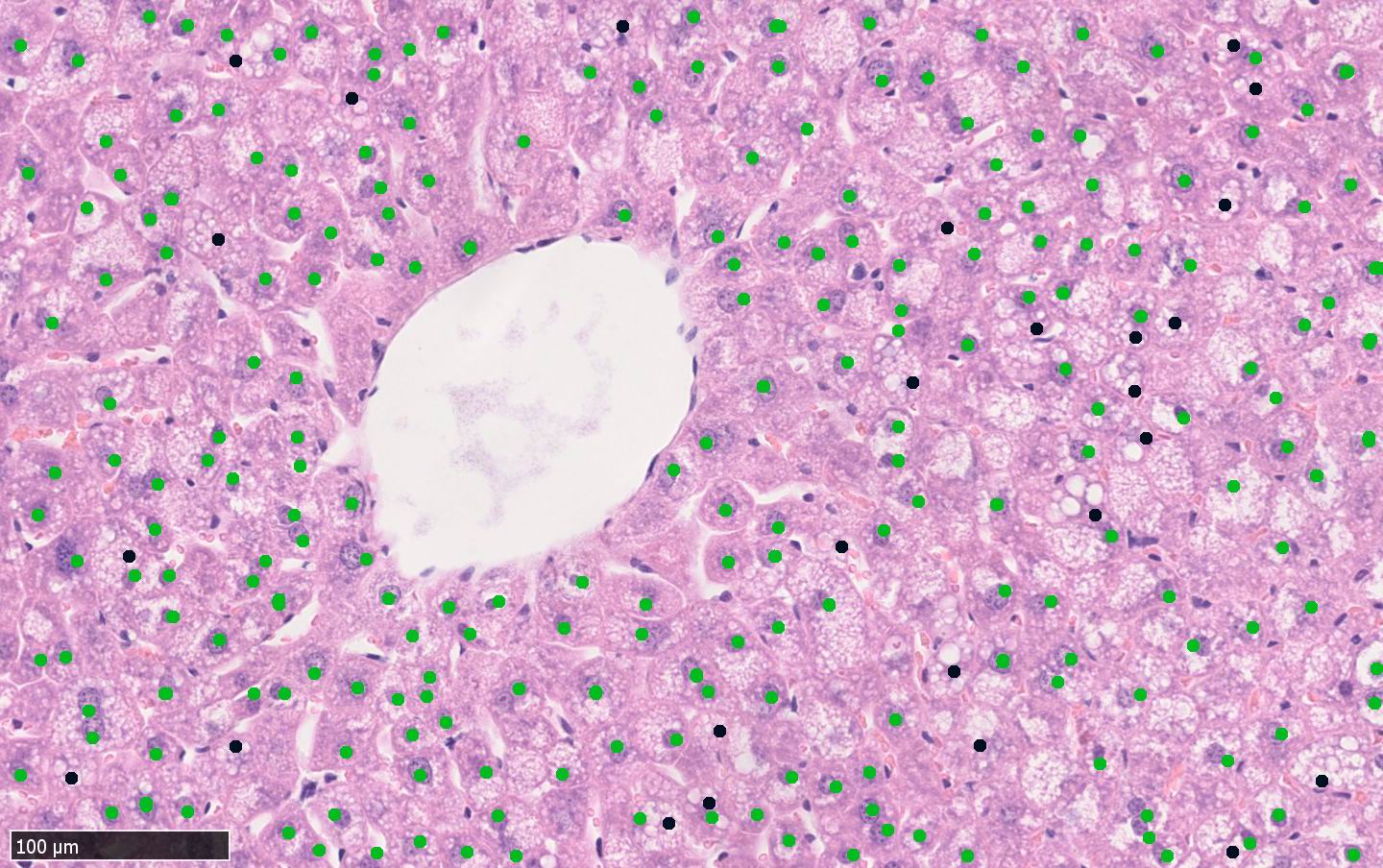

Supplement: Supplementary file 7 [file DataSheet10.ZIP › NASH SCORE-db(2)/db8,16 - 副本/14.jpg]

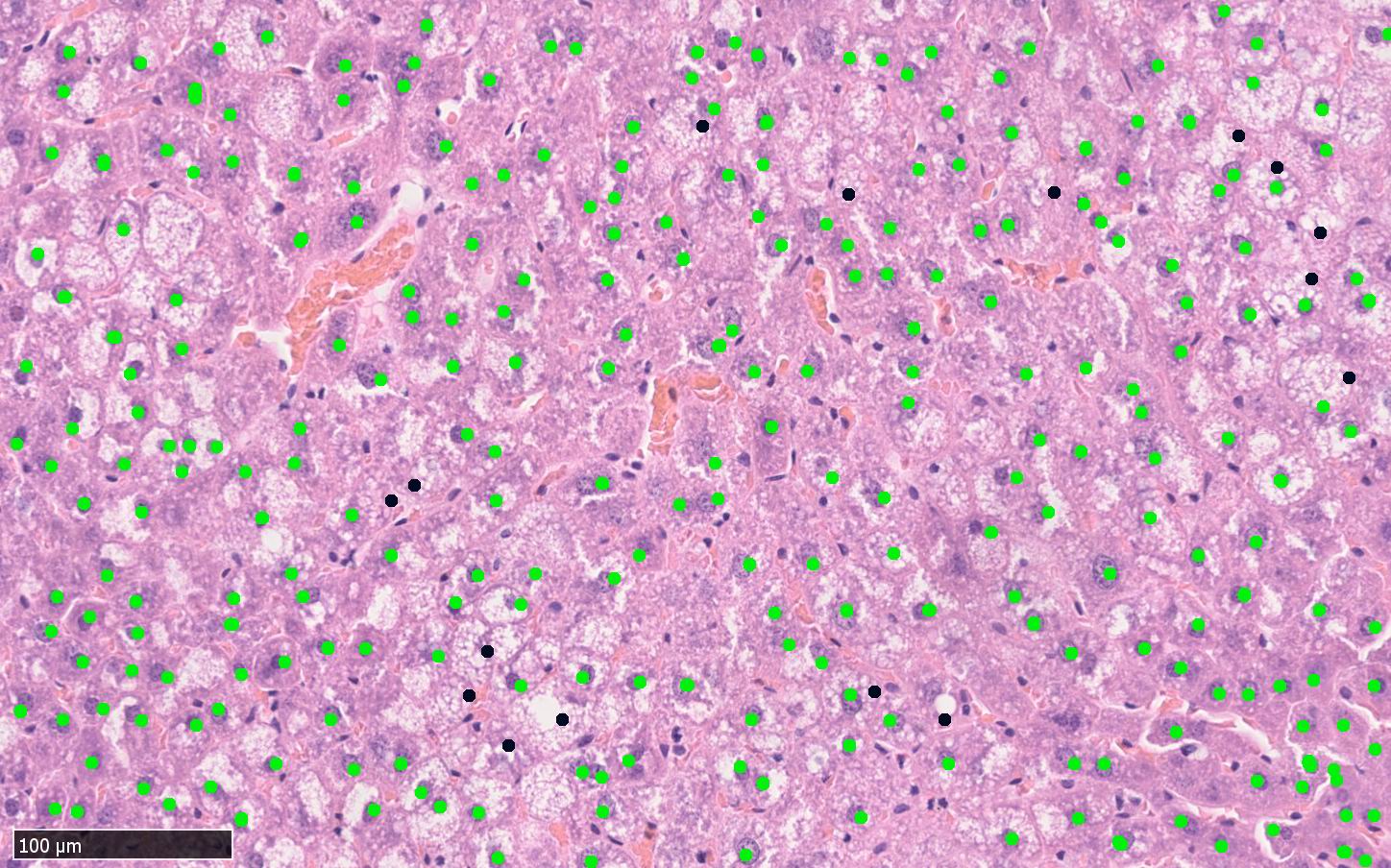

Supplement: Supplementary file 7 [file DataSheet10.ZIP › NASH SCORE-db(2)/db8,16 - 副本/15.jpg]

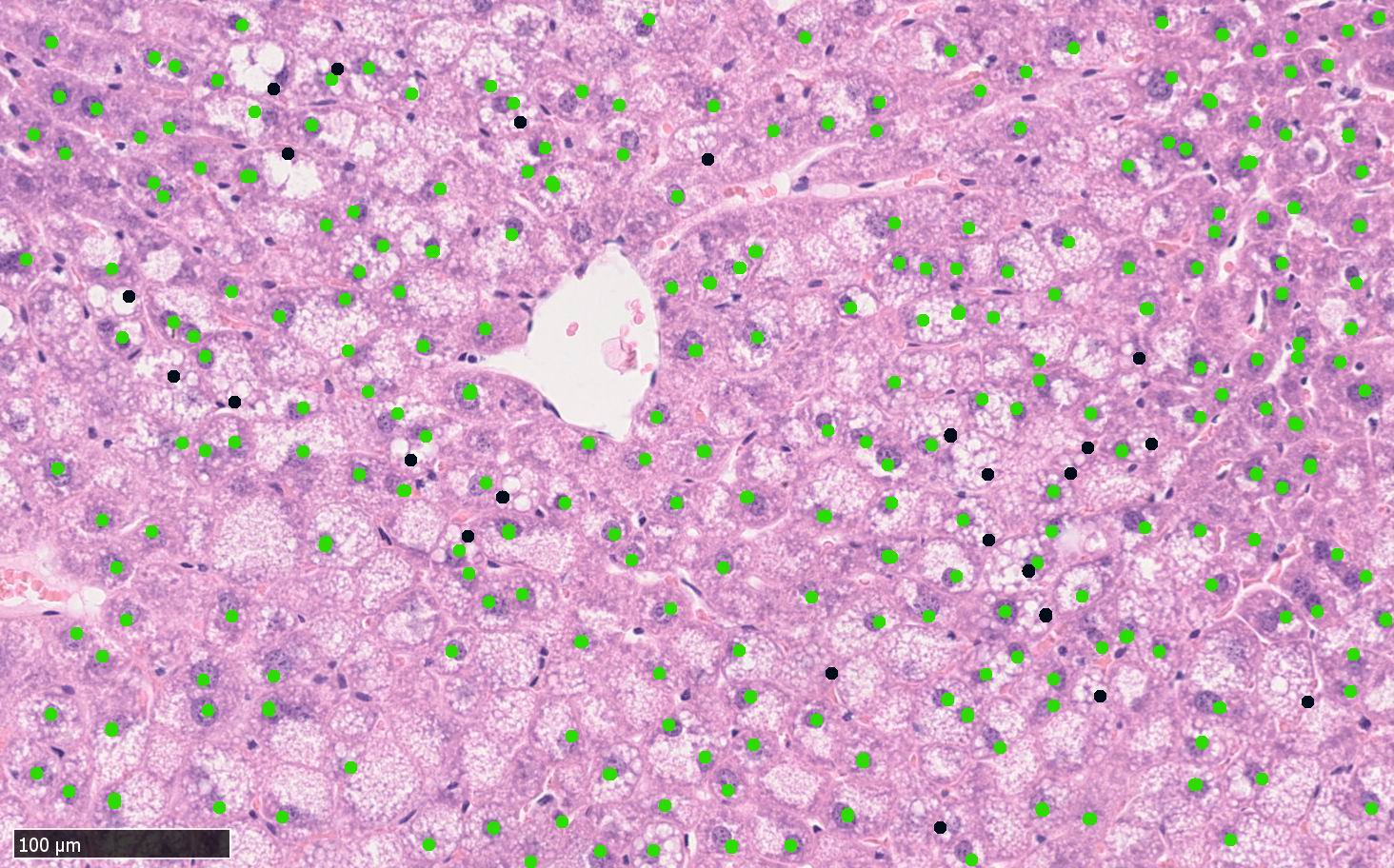

Supplement: Supplementary file 7 [file DataSheet10.ZIP › NASH SCORE-db(2)/db8,16 - 副本/16.jpg]

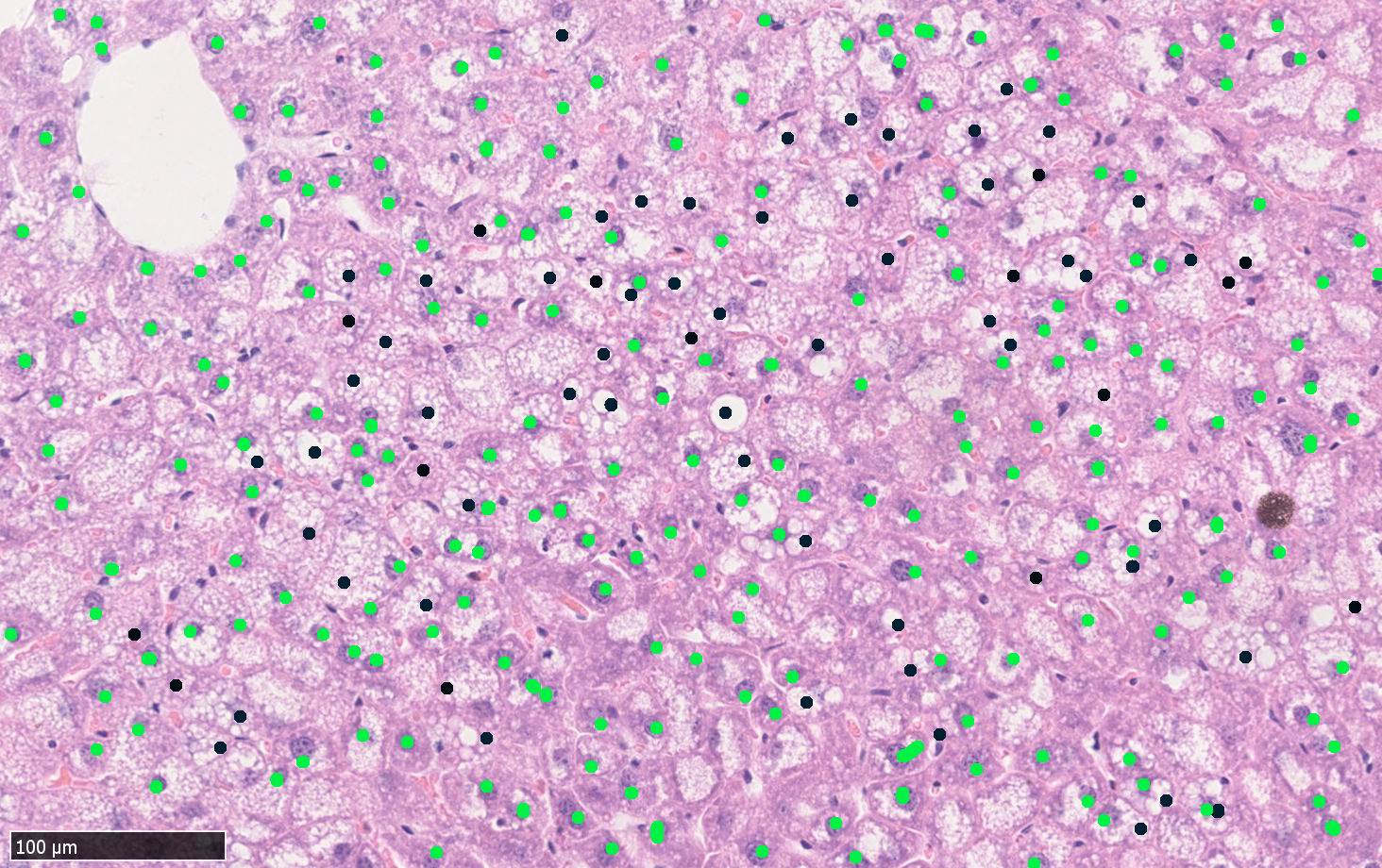

Supplement: Supplementary file 7 [file DataSheet10.ZIP › NASH SCORE-db(2)/db8,16 - 副本/17.jpg]

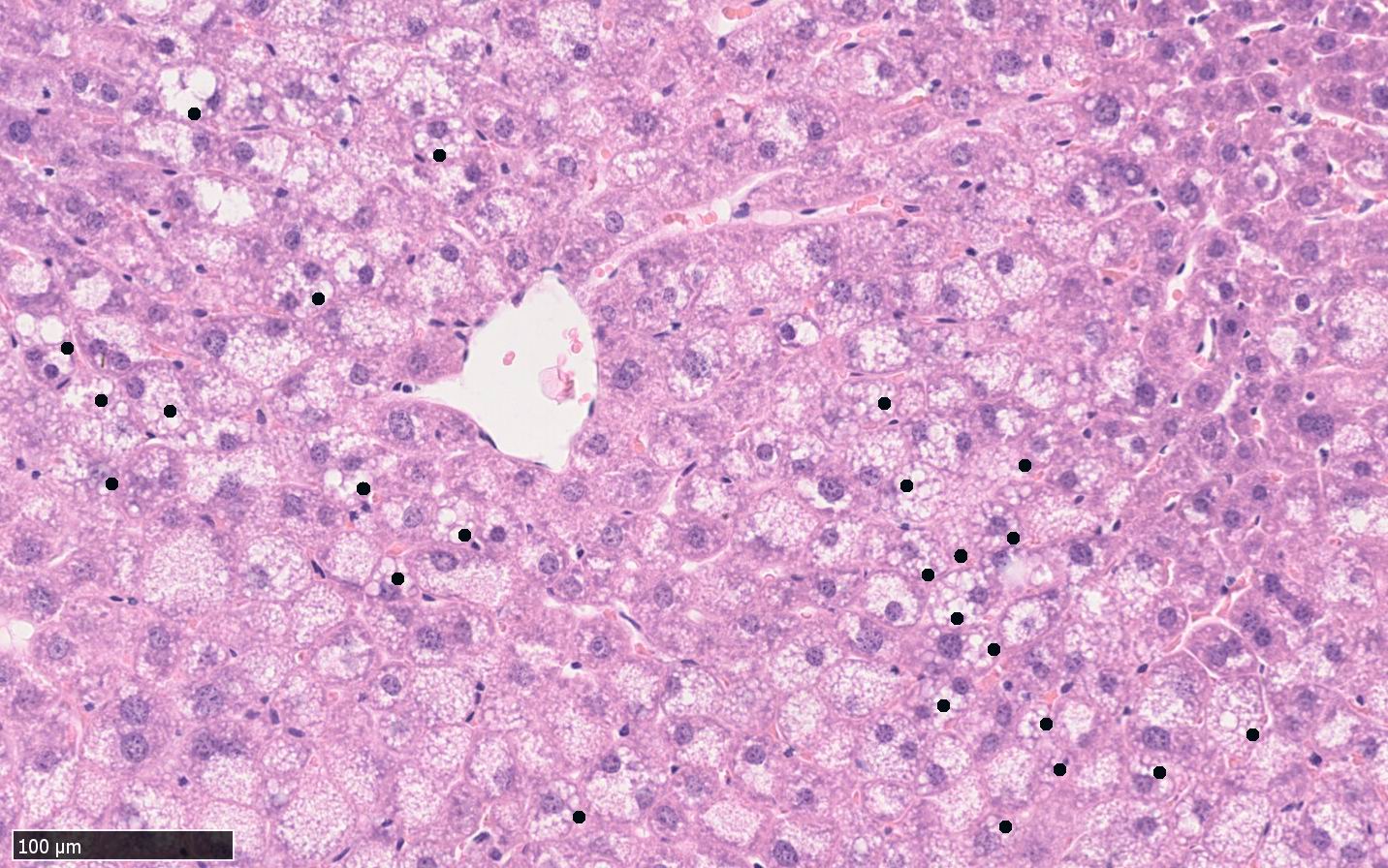

Supplement: Supplementary file 7 [file DataSheet10.ZIP › NASH SCORE-db(2)/db8,16 - 副本/18.jpg]

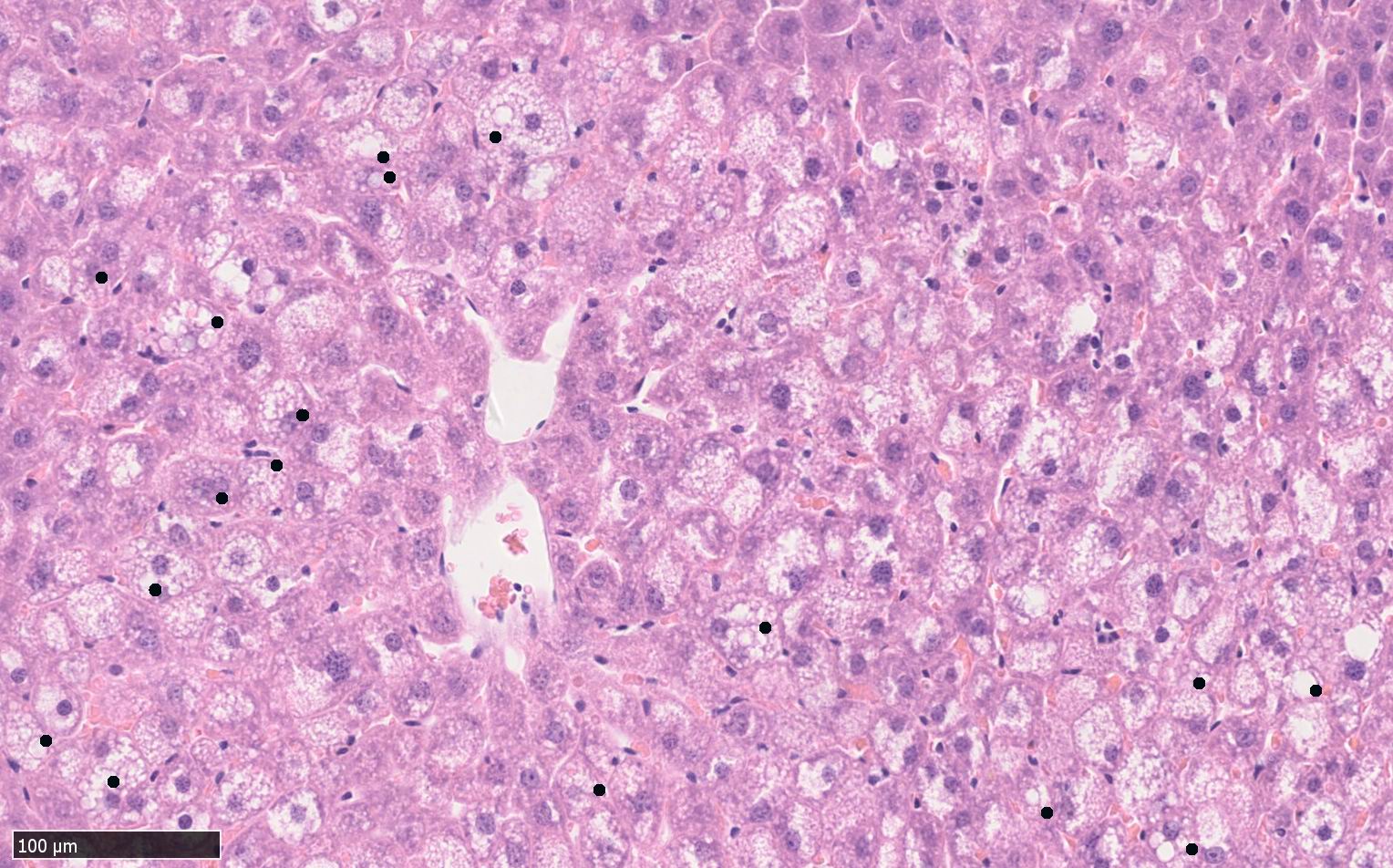

Supplement: Supplementary file 7 [file DataSheet10.ZIP › NASH SCORE-db(2)/db8,16 - 副本/19.jpg]

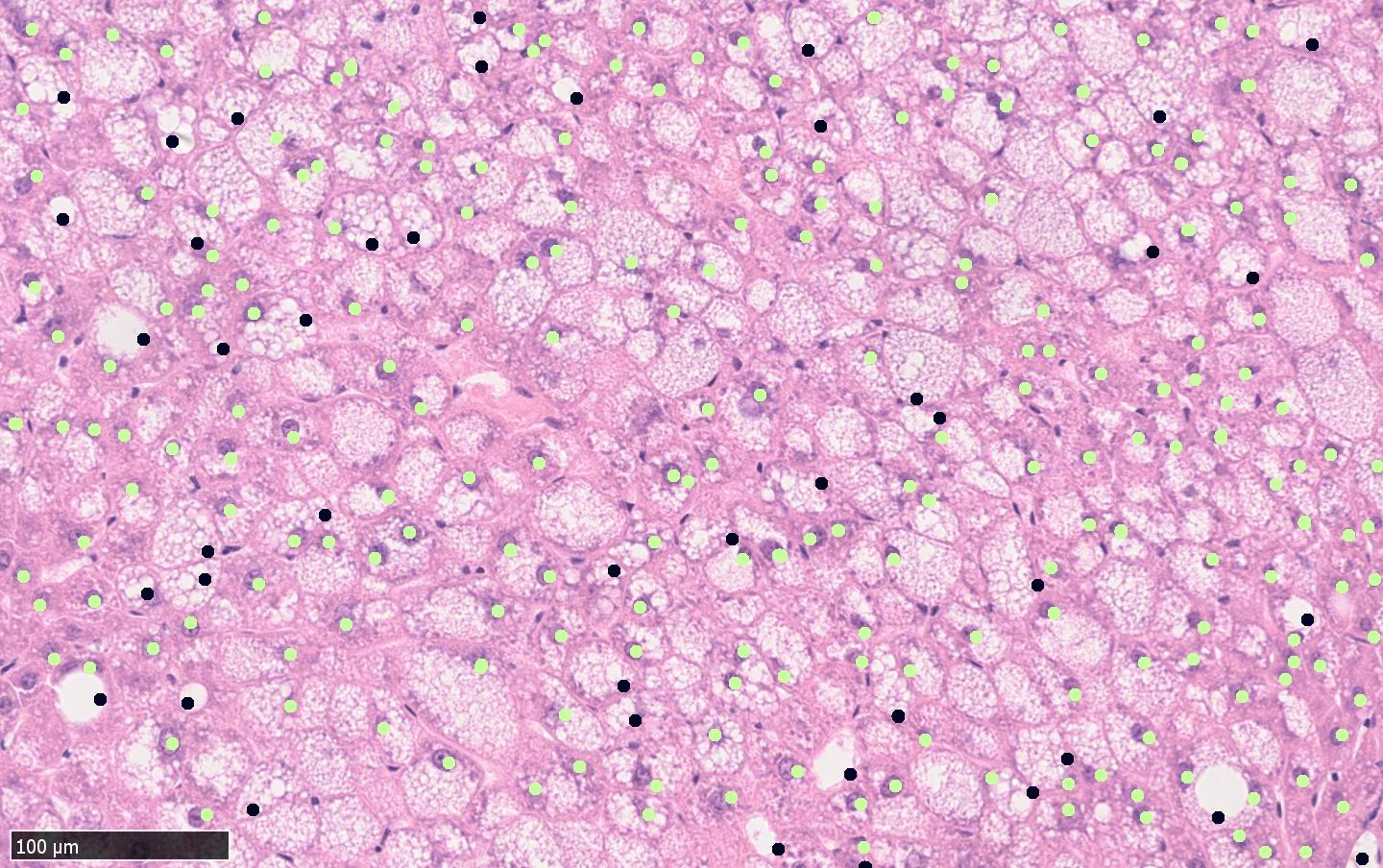

Supplement: Supplementary file 7 [file DataSheet10.ZIP › NASH SCORE-db(2)/db8,16 - 副本/2.jpg]

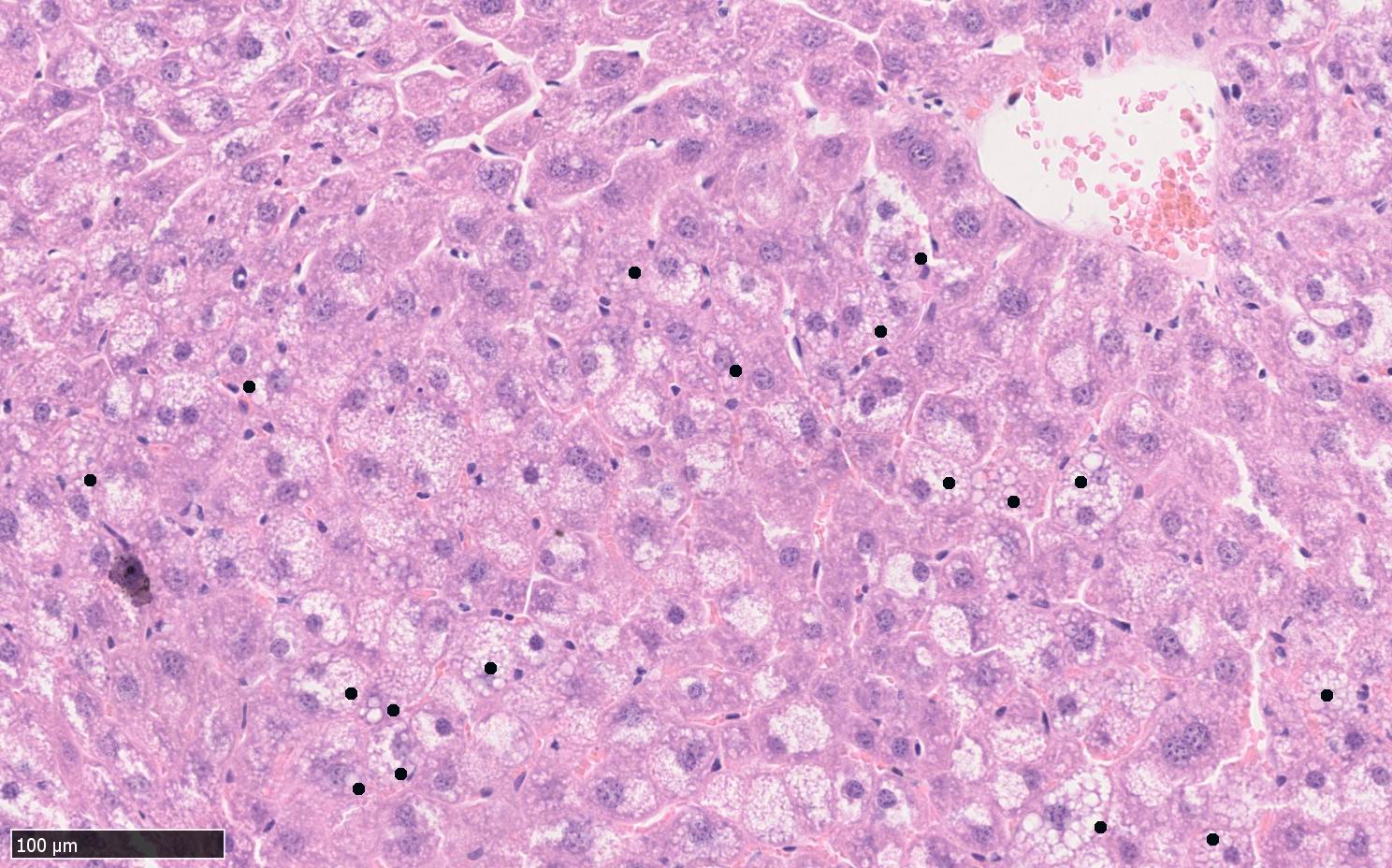

Supplement: Supplementary file 7 [file DataSheet10.ZIP › NASH SCORE-db(2)/db8,16 - 副本/20.jpg]

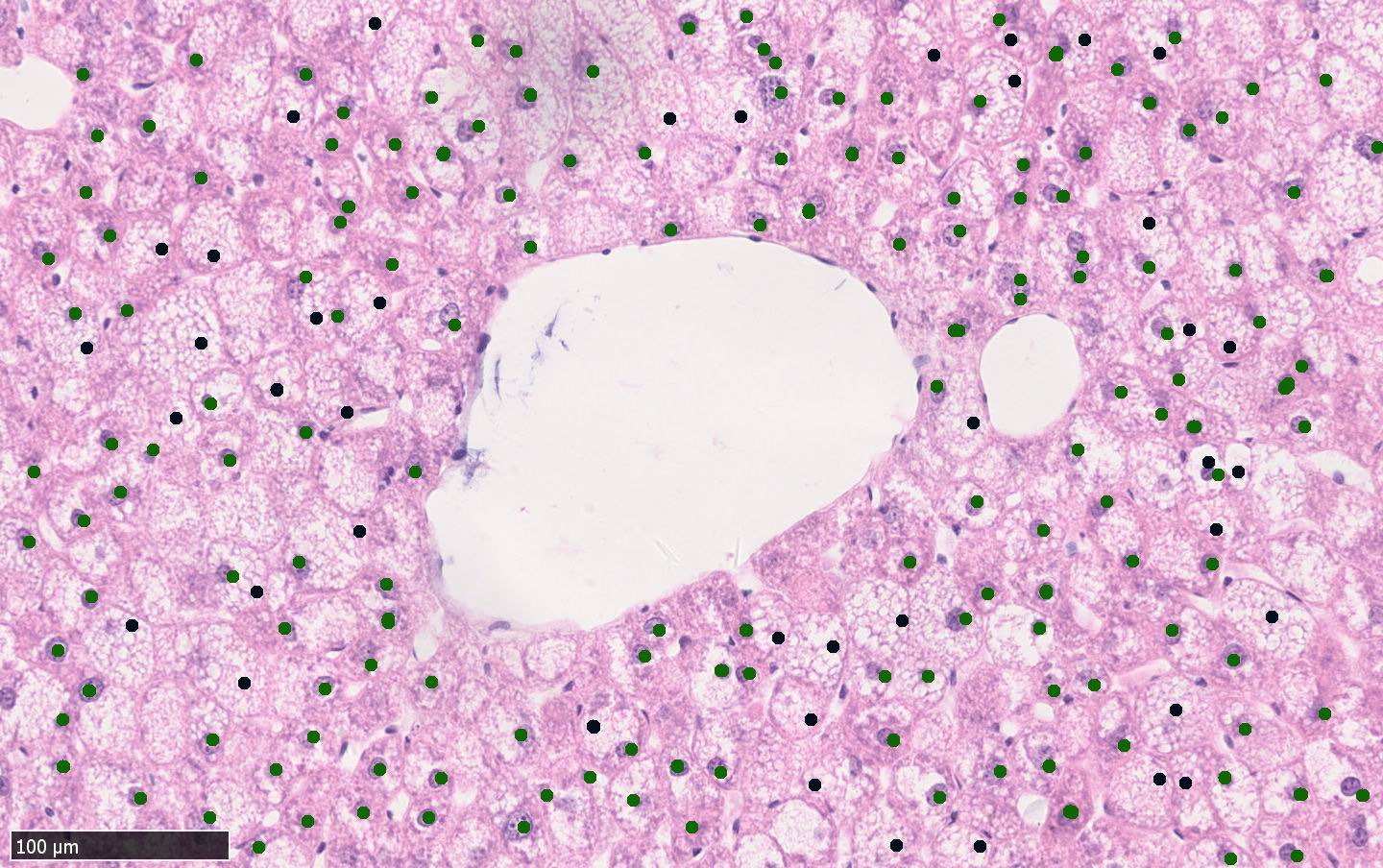

Supplement: Supplementary file 7 [file DataSheet10.ZIP › NASH SCORE-db(2)/db8,16 - 副本/3.jpg]

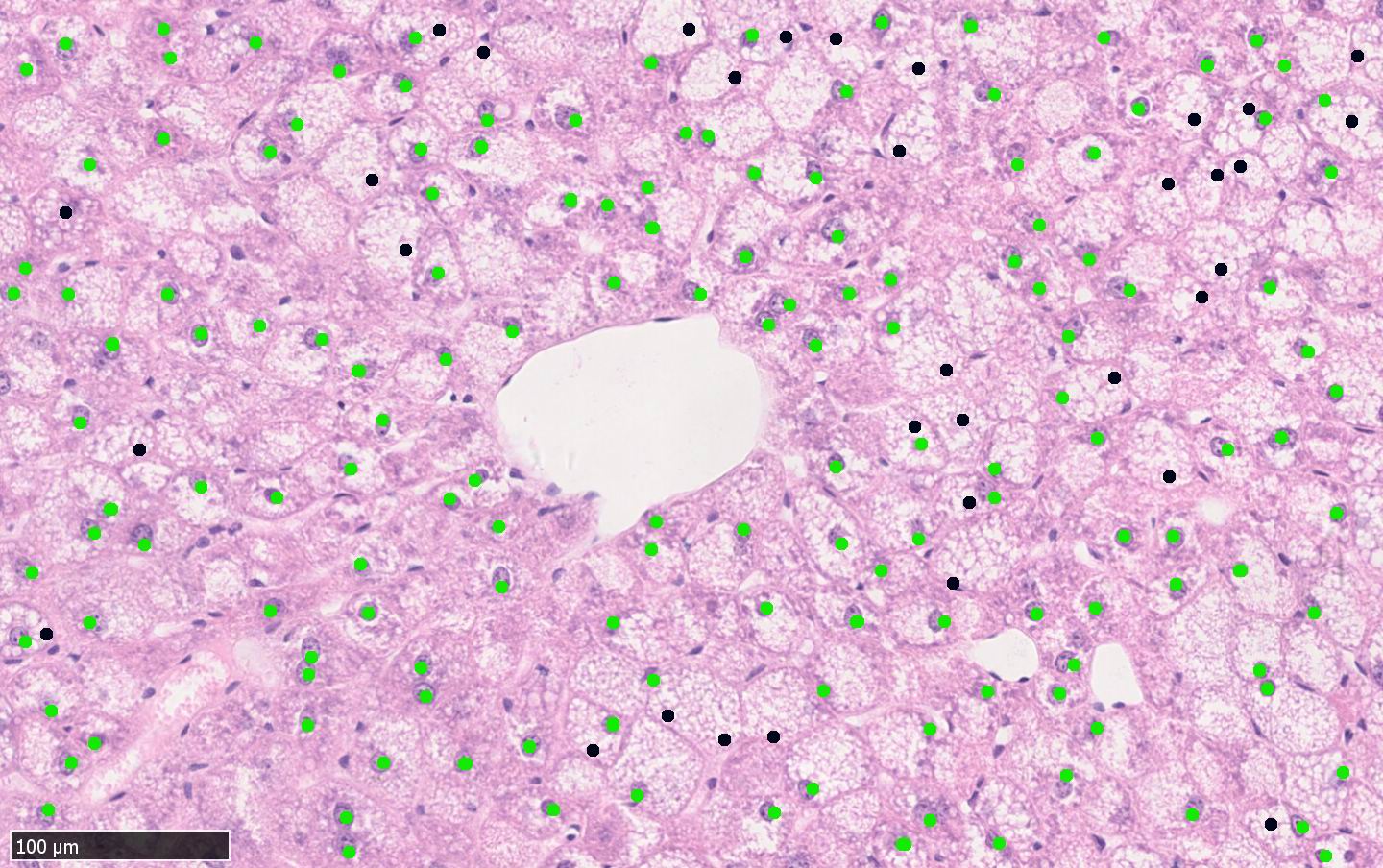

Supplement: Supplementary file 7 [file DataSheet10.ZIP › NASH SCORE-db(2)/db8,16 - 副本/4.jpg]

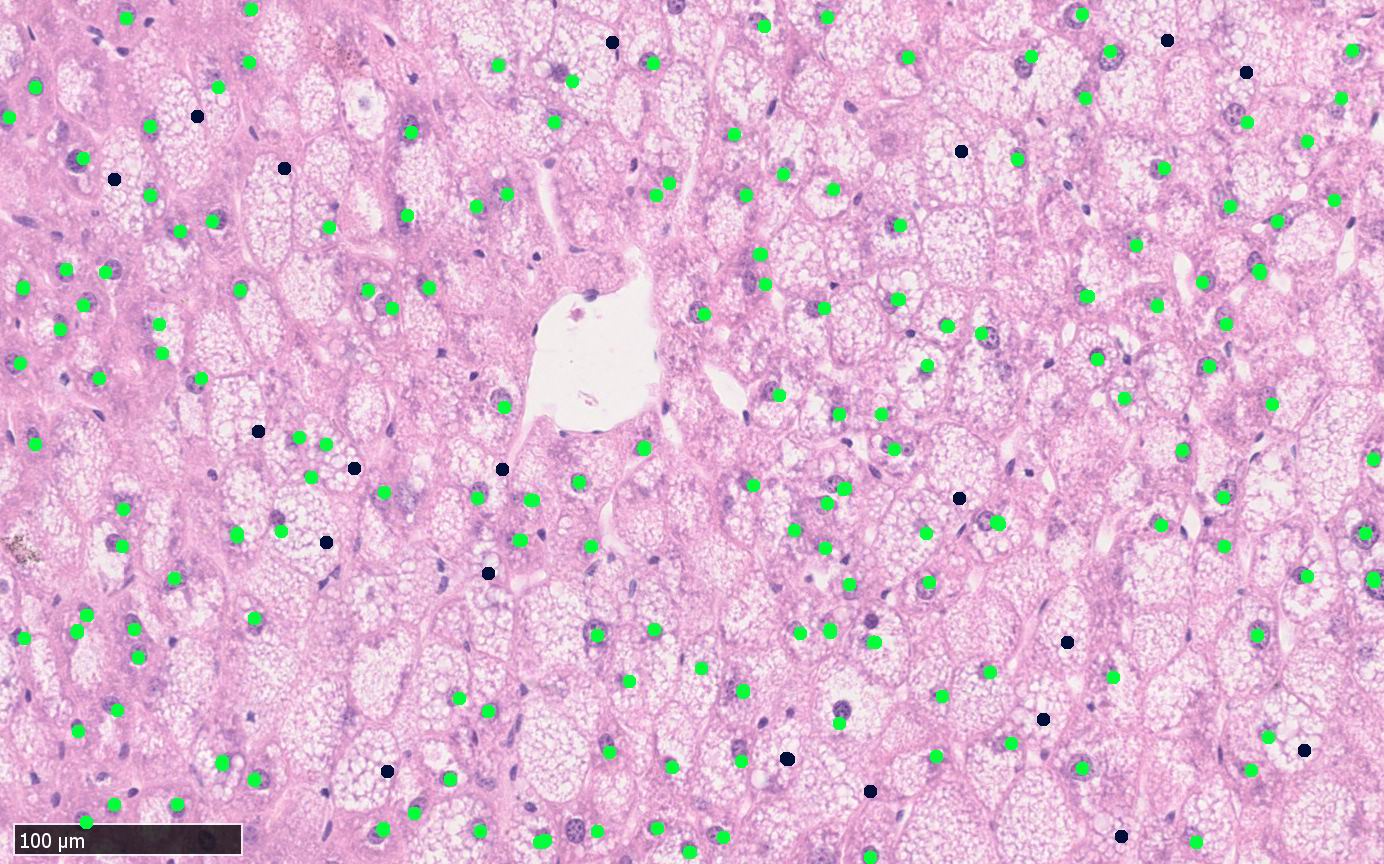

Supplement: Supplementary file 7 [file DataSheet10.ZIP › NASH SCORE-db(2)/db8,16 - 副本/5.jpg]

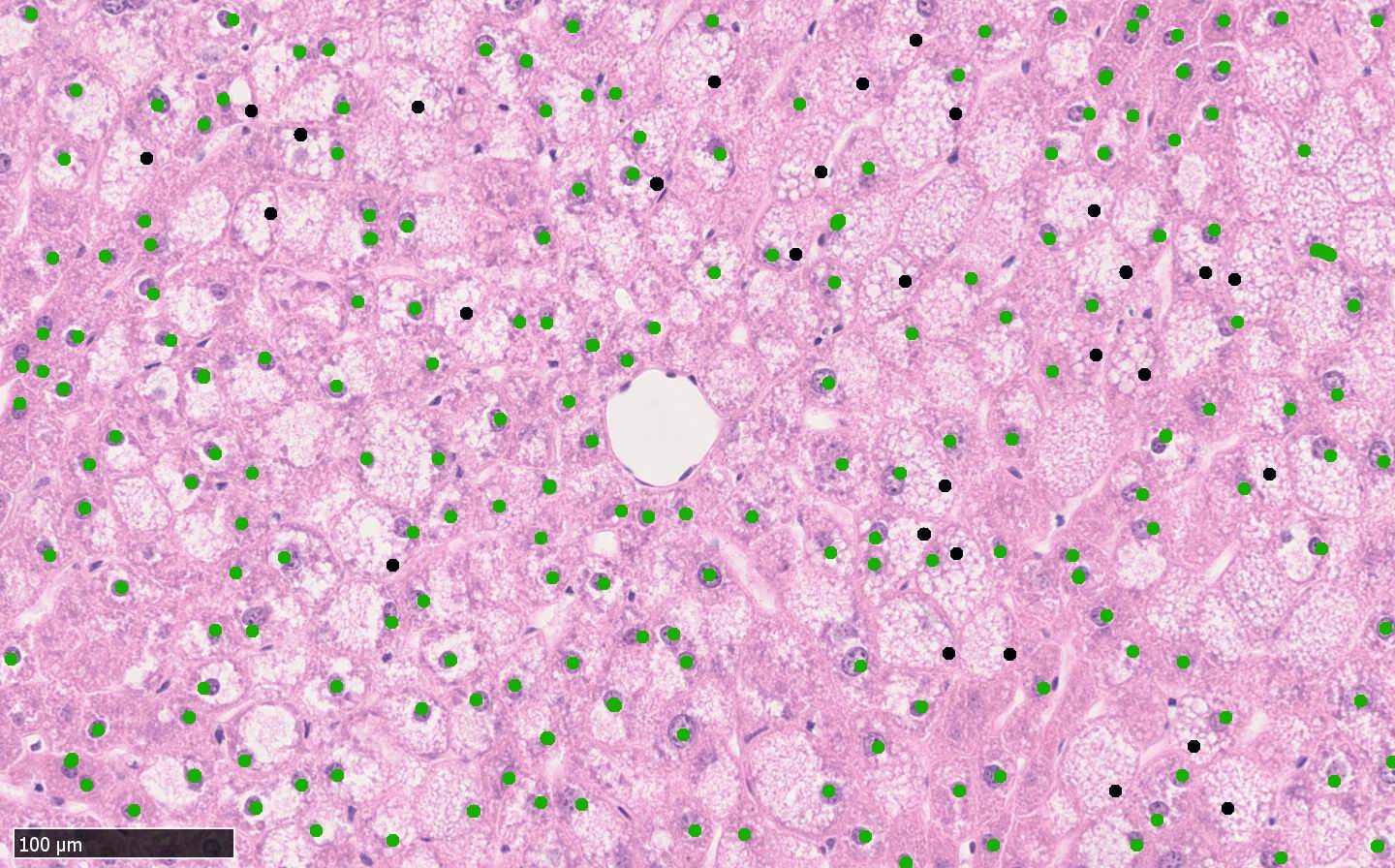

Supplement: Supplementary file 7 [file DataSheet10.ZIP › NASH SCORE-db(2)/db8,16 - 副本/6.jpg]

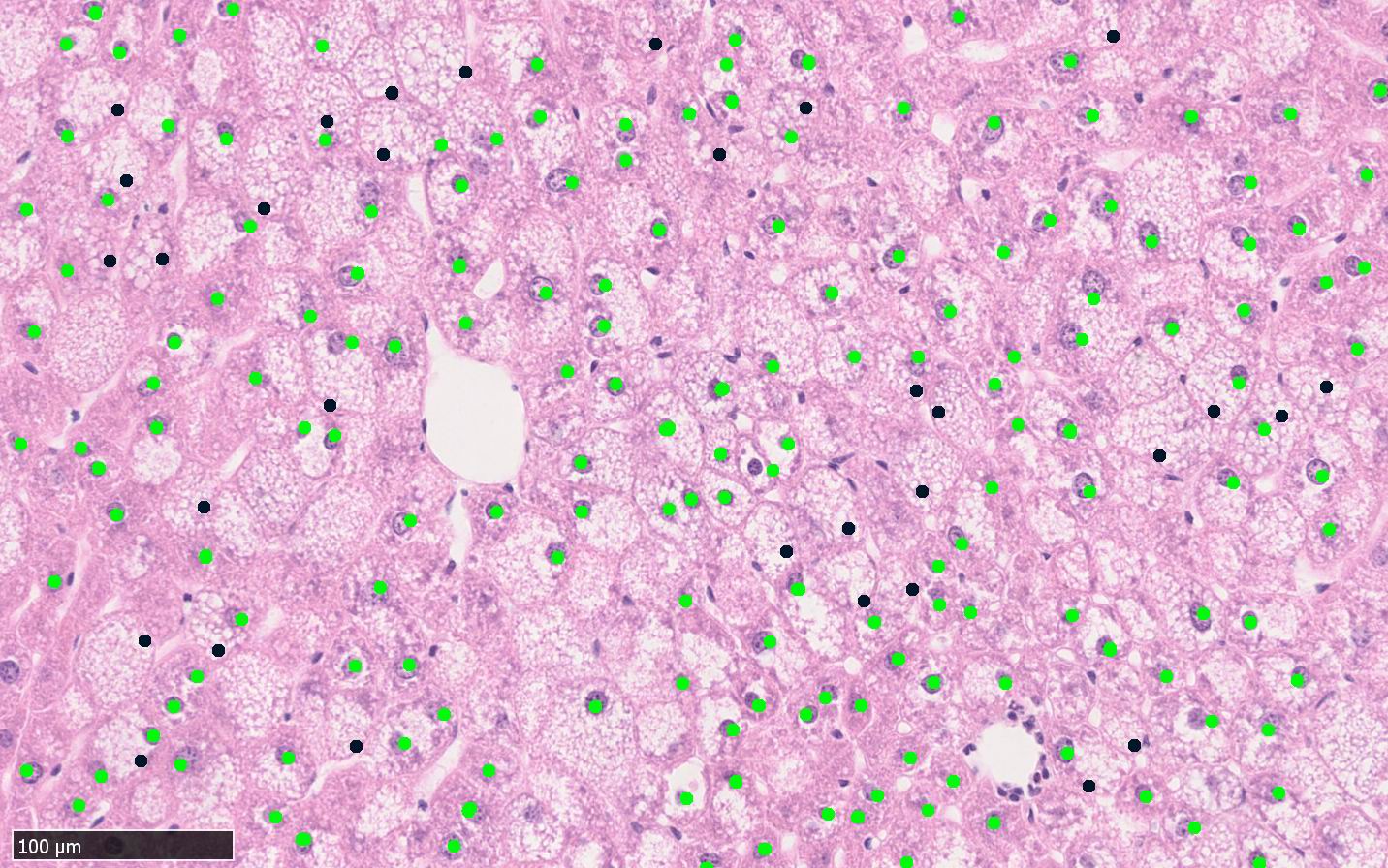

Supplement: Supplementary file 7 [file DataSheet10.ZIP › NASH SCORE-db(2)/db8,16 - 副本/7.jpg]

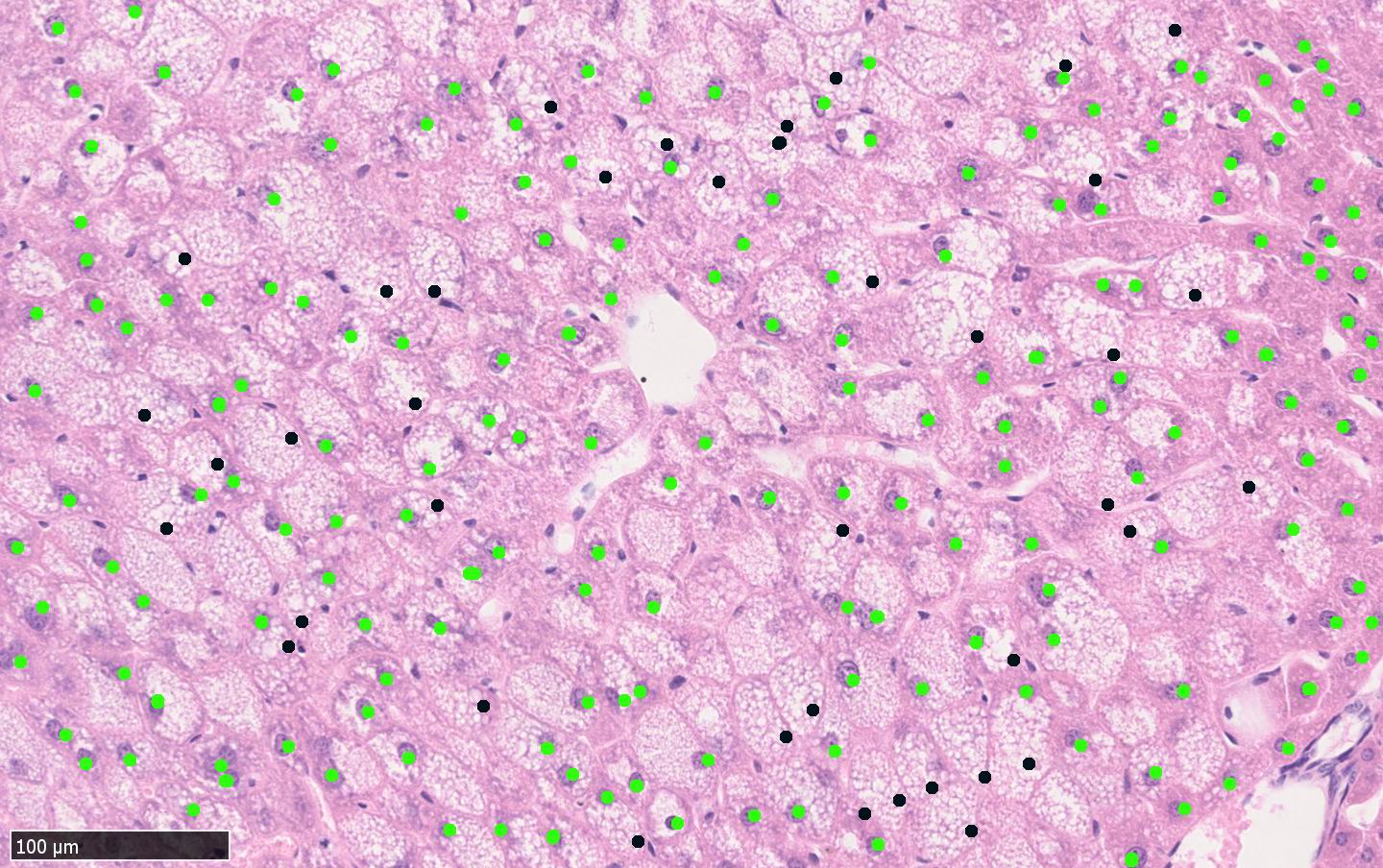

Supplement: Supplementary file 7 [file DataSheet10.ZIP › NASH SCORE-db(2)/db8,16 - 副本/8.jpg]

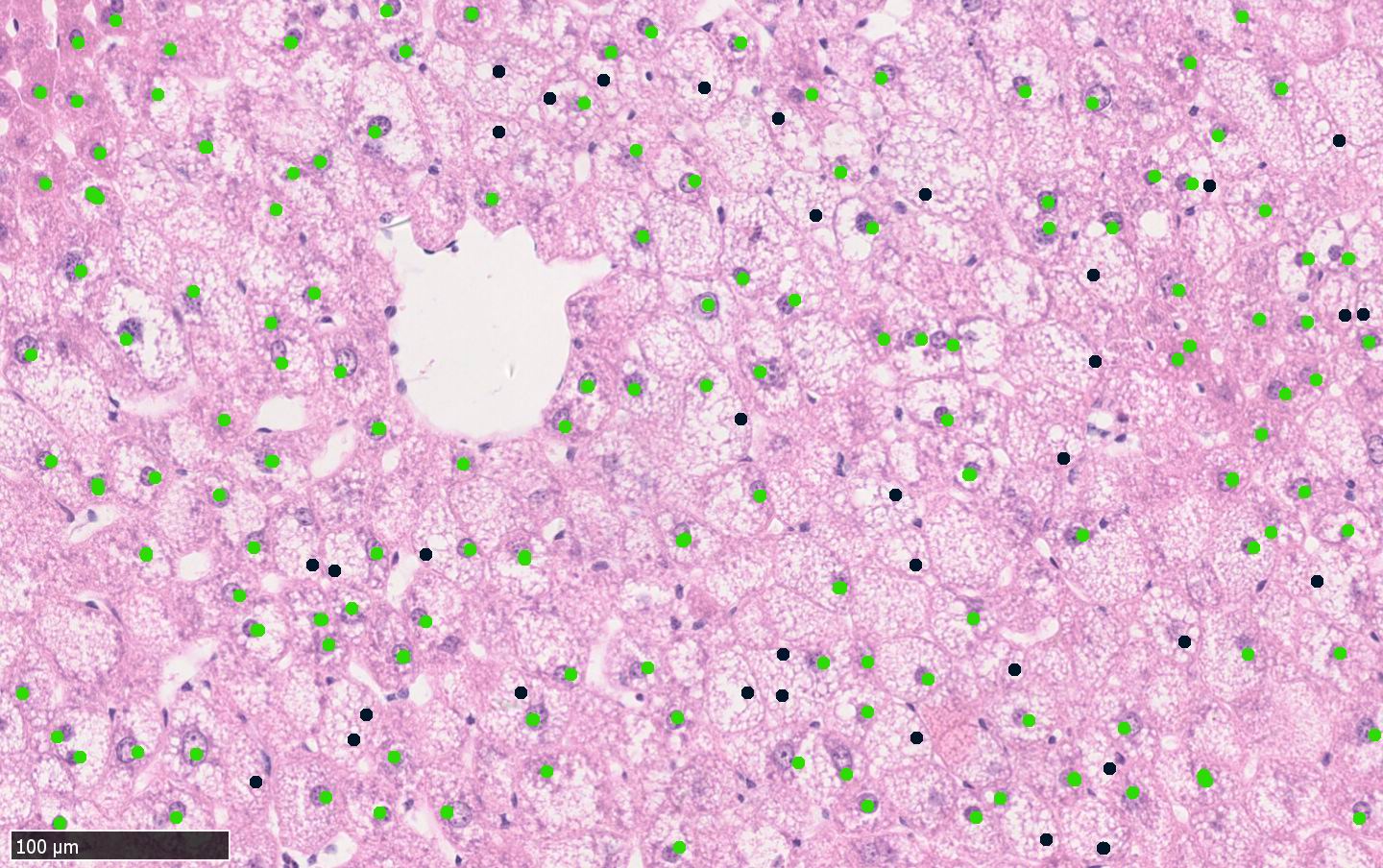

Supplement: Supplementary file 7 [file DataSheet10.ZIP › NASH SCORE-db(2)/db8,16 - 副本/9.jpg]

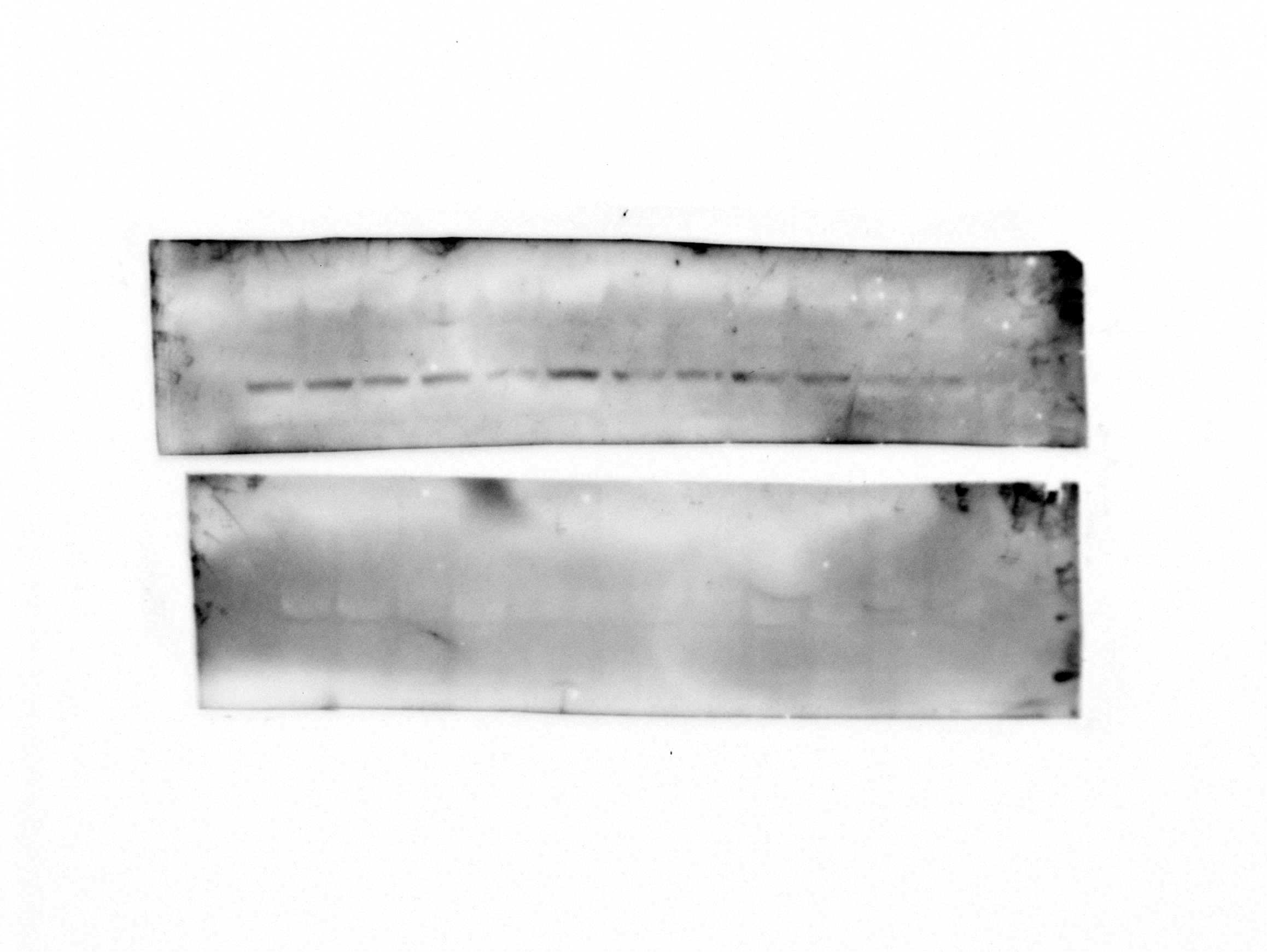

Supplement: Supplementary file 8 [file DataSheet6.ZIP › WB2/JAK1.tif]

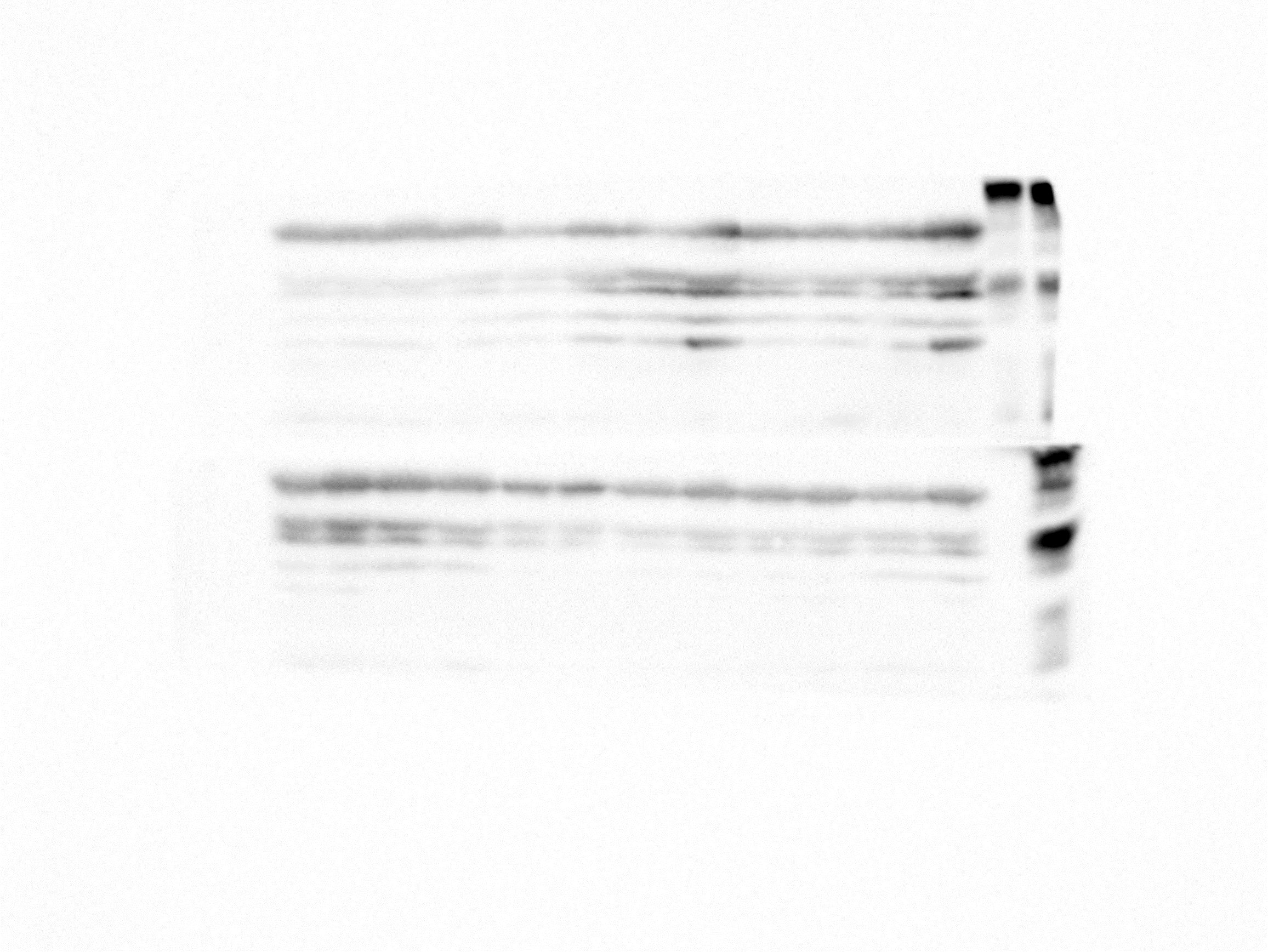

Supplement: Supplementary file 8 [file DataSheet6.ZIP › WB2/JNK2 上1,2 下3 (3) liver.tif]

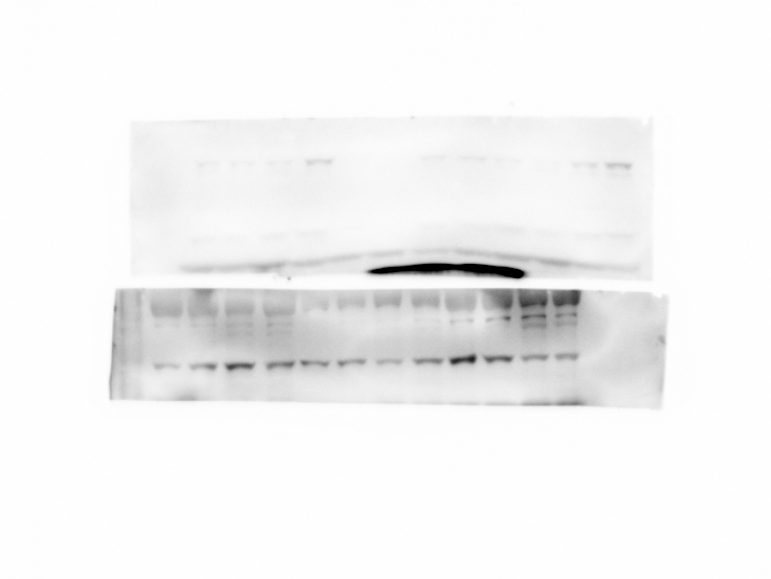

Supplement: Supplementary file 8 [file DataSheet6.ZIP › WB2/liver 3pi 上FGFR4(95) 下CHREBP(93) (19).tif]

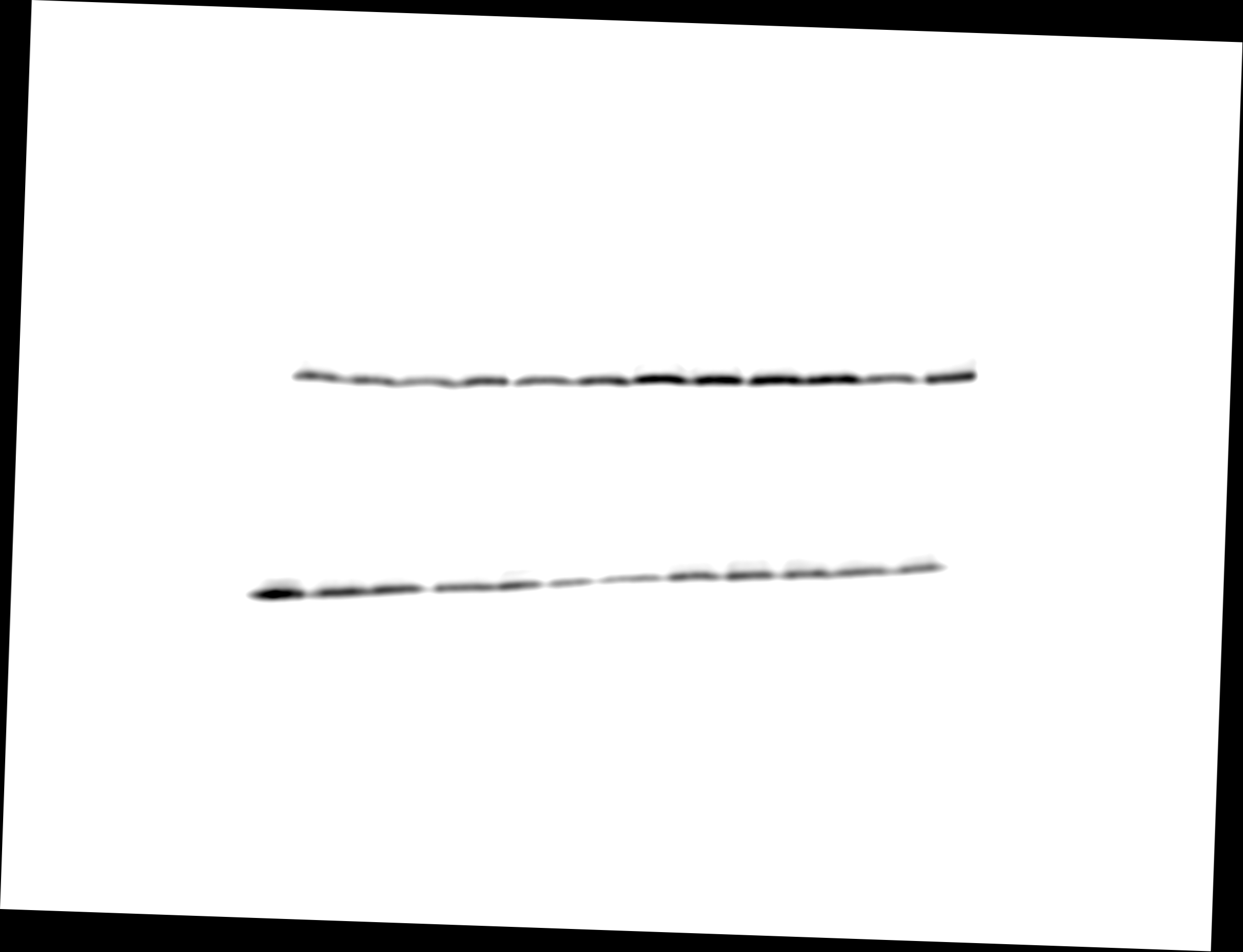

Supplement: Supplementary file 8 [file DataSheet6.ZIP › WB2/liver ERK2 上1,2 下3 (3).tif]

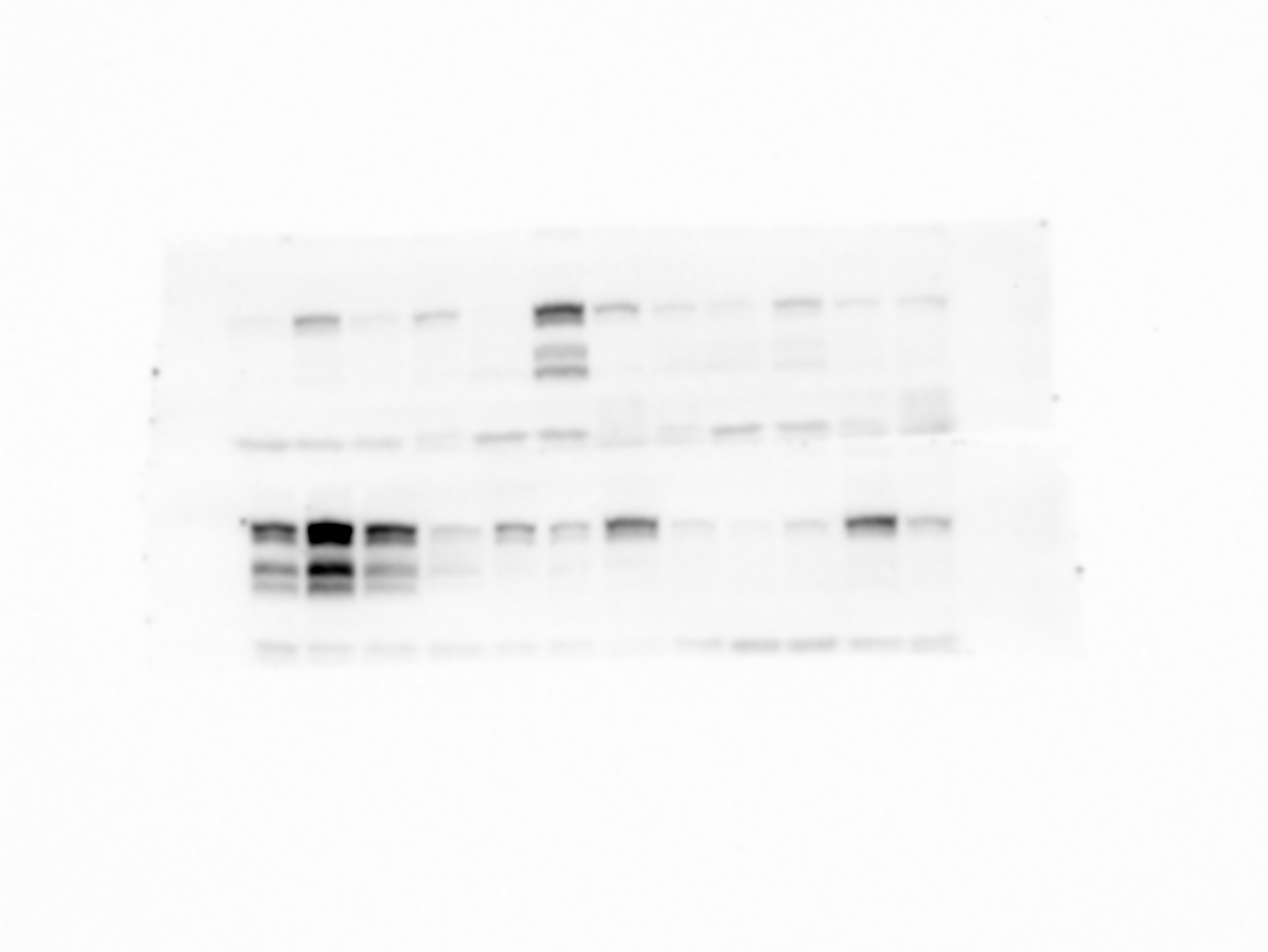

Supplement: Supplementary file 8 [file DataSheet6.ZIP › WB2/liver p-stat3 上 1,2 下3 (8).tif]

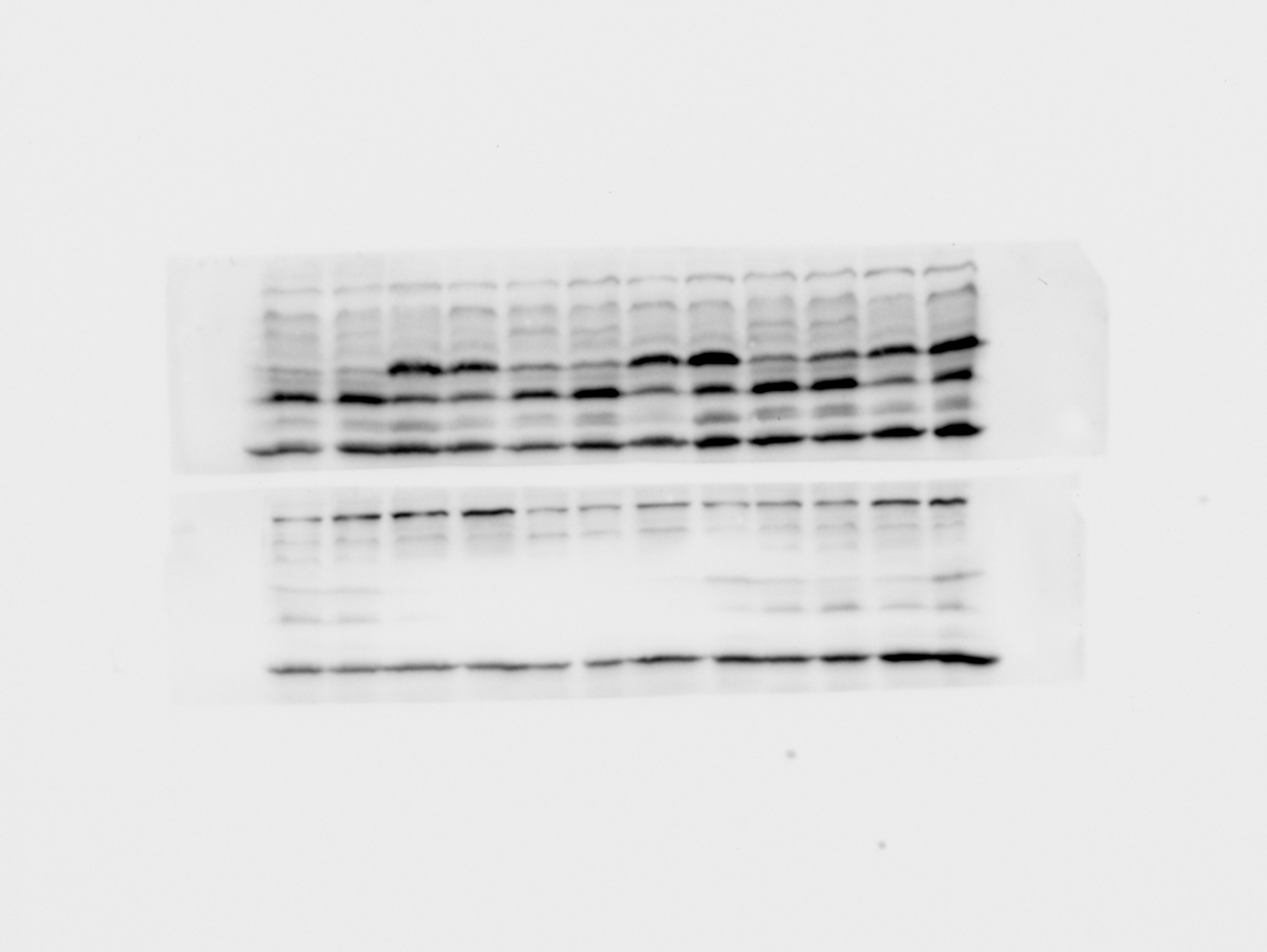

Supplement: Supplementary file 8 [file DataSheet6.ZIP › WB2/liver 上1,2 p65,下3 p65 (1).tif]

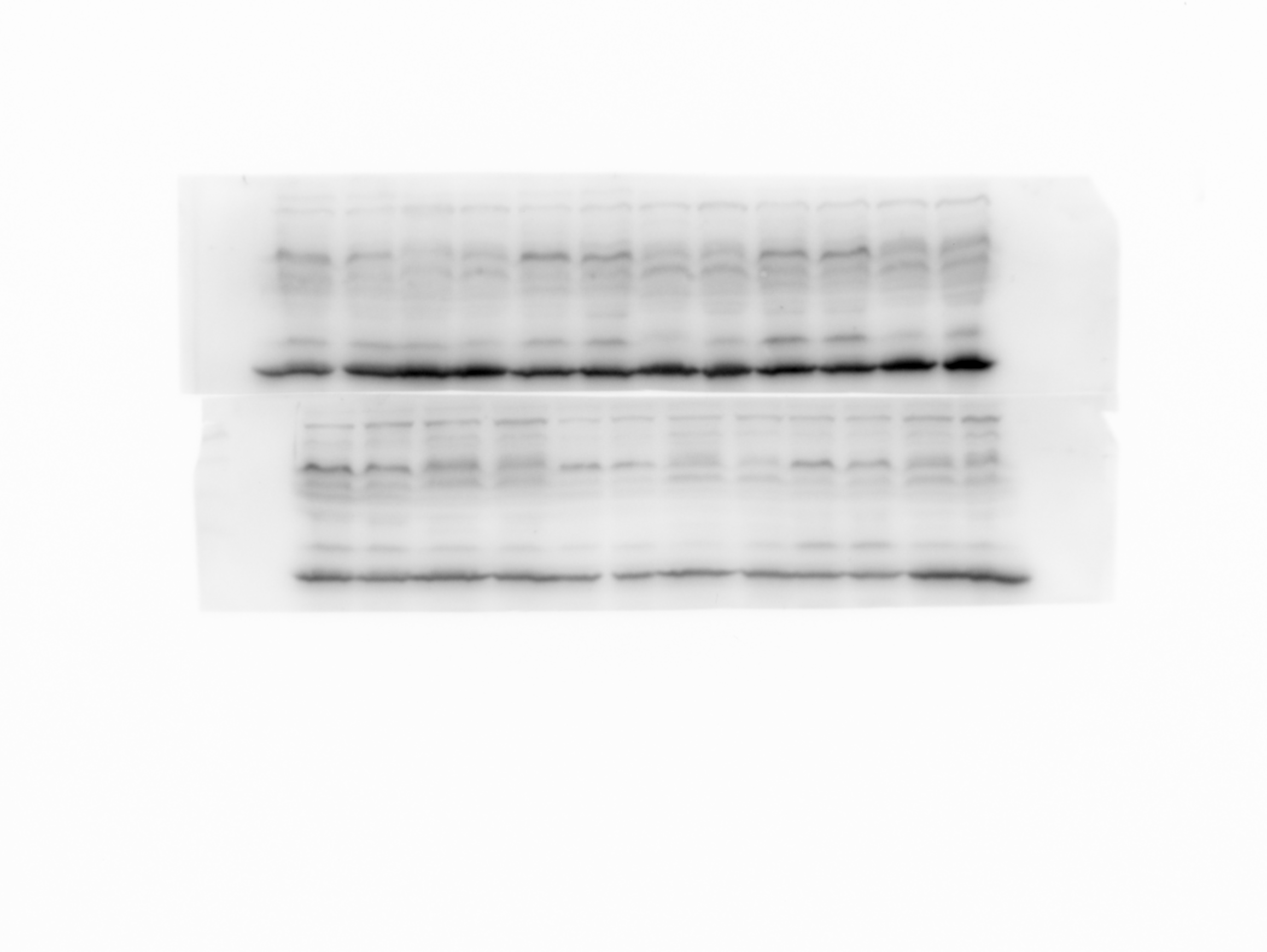

Supplement: Supplementary file 8 [file DataSheet6.ZIP › WB2/LXRa 上1,2 下3批 (4).tif]

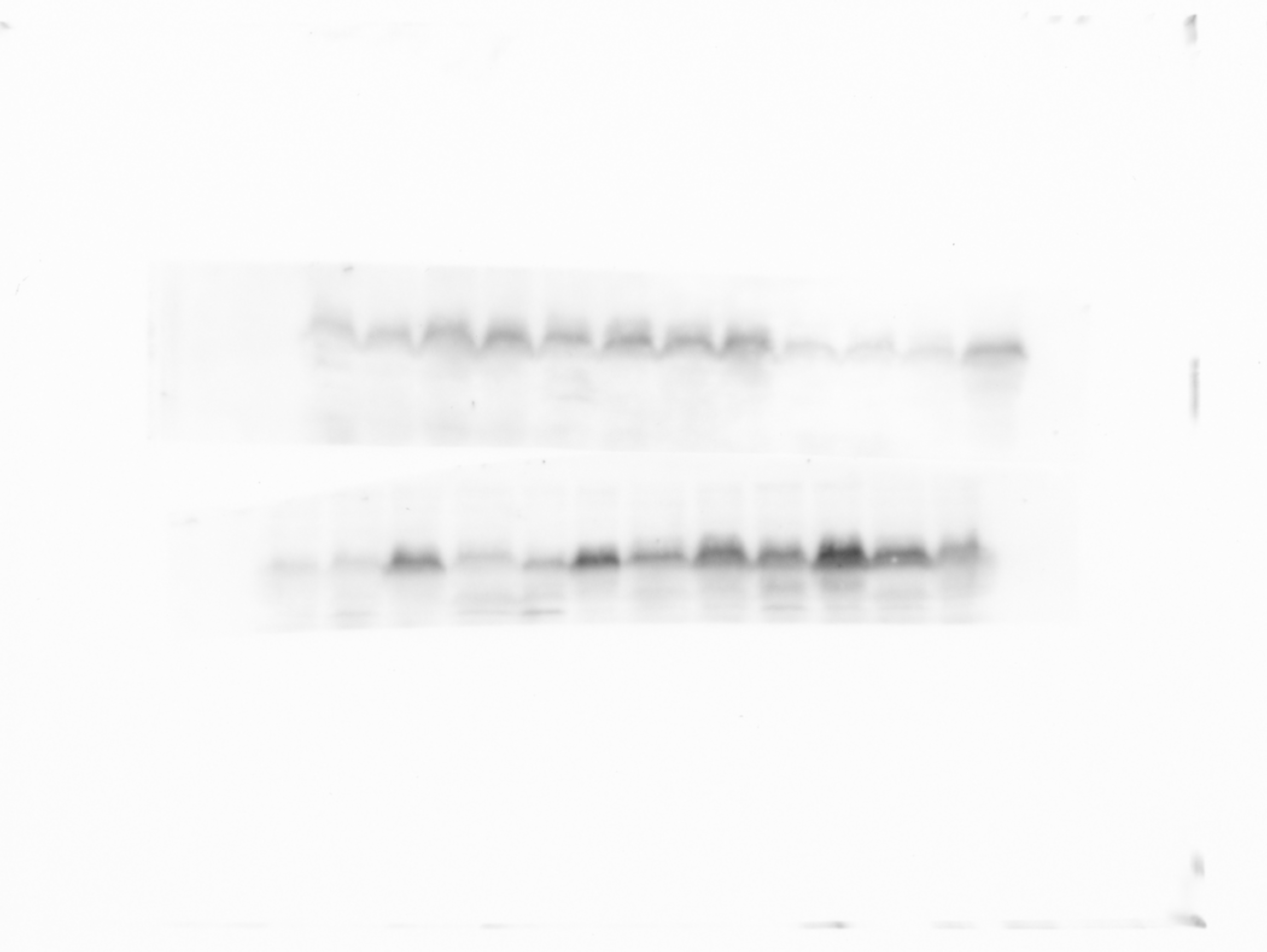

Supplement: Supplementary file 8 [file DataSheet6.ZIP › WB2/p-ERK(42,44) 上1,2 下3 liver (8).tif]

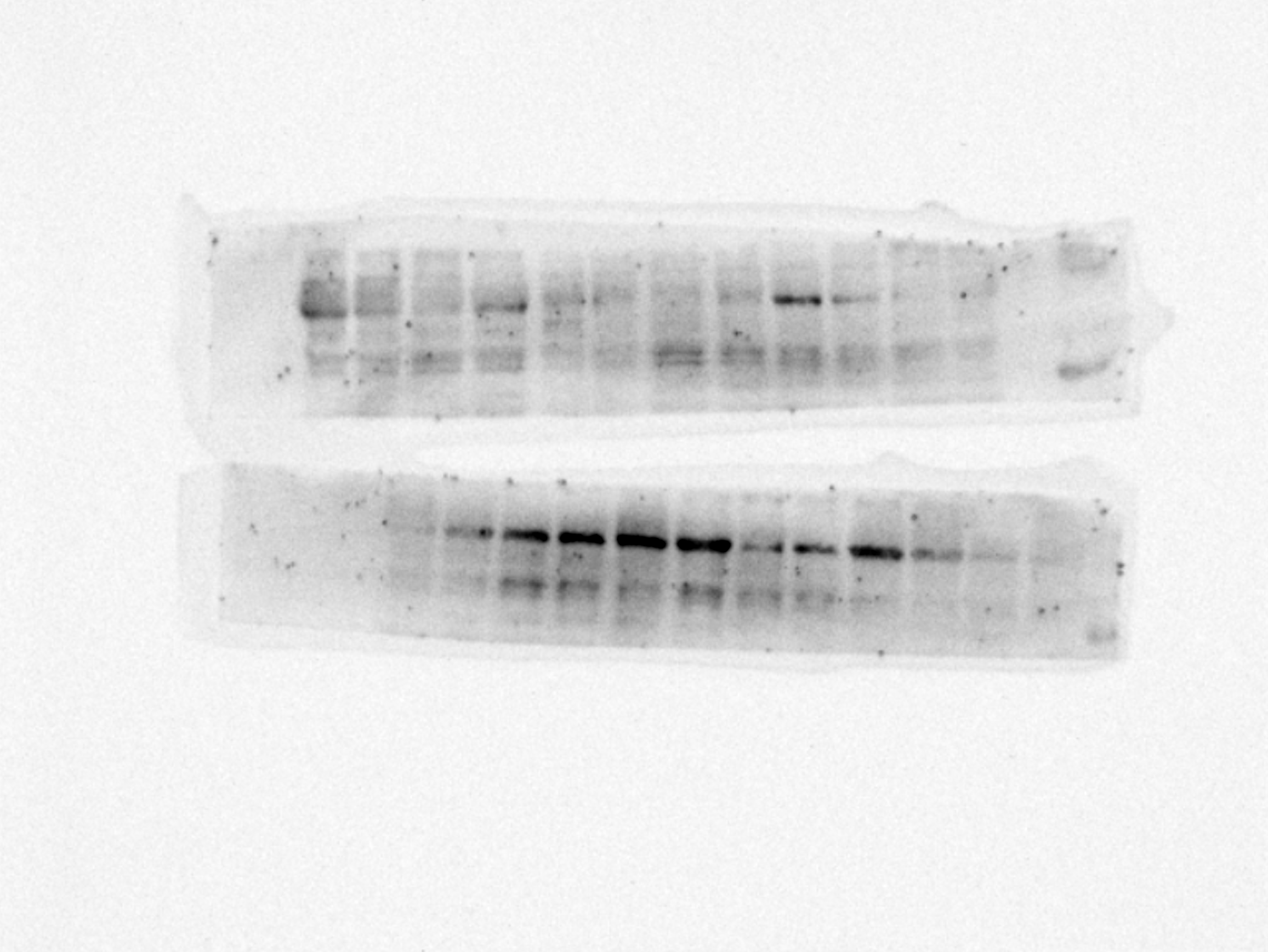

Supplement: Supplementary file 8 [file DataSheet6.ZIP › WB2/p-JAK1(137) 上3 下1,2 liver (7).tif]

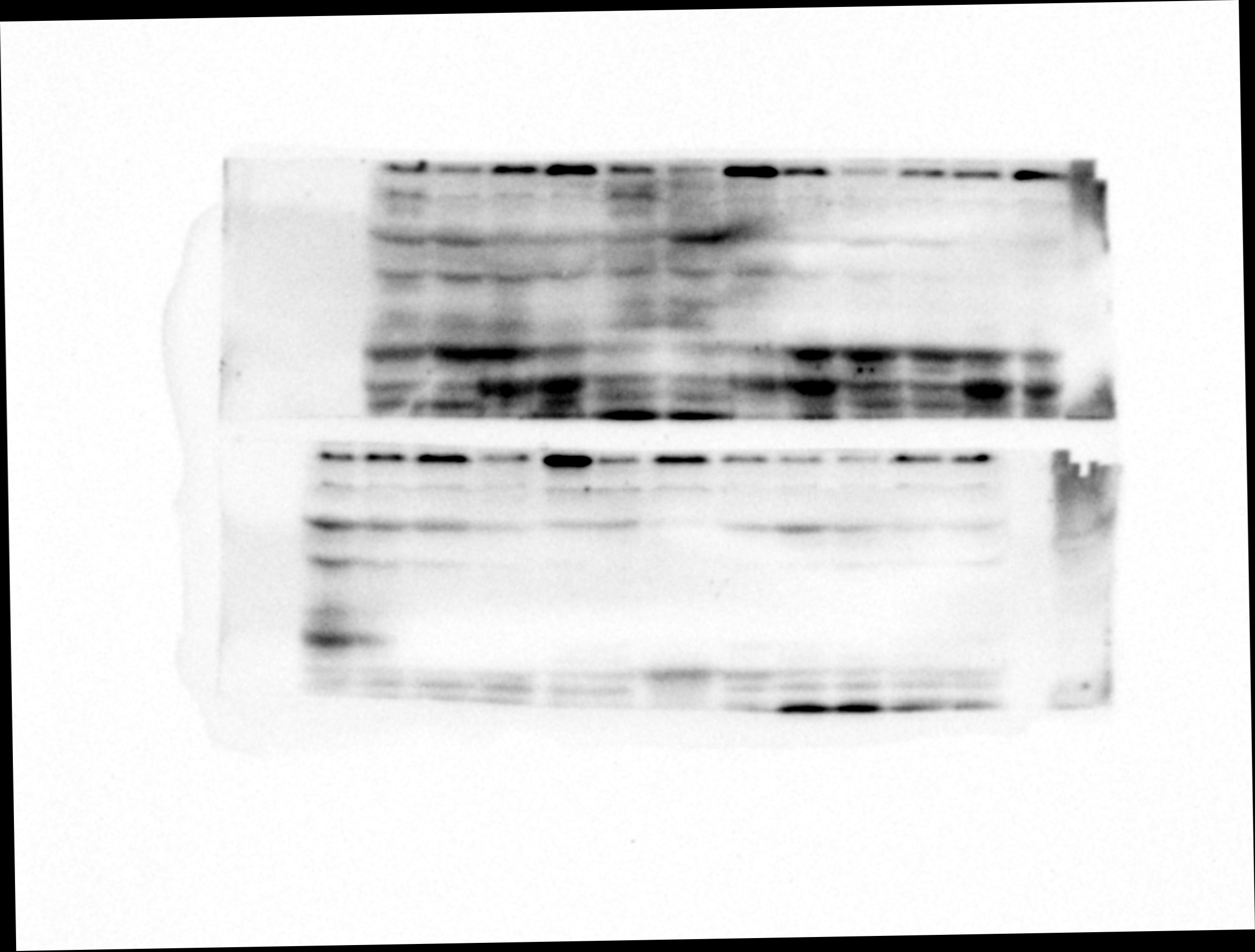

Supplement: Supplementary file 8 [file DataSheet6.ZIP › WB2/p-JNK(46,54) 上1,2 下3 liver (3).tif]

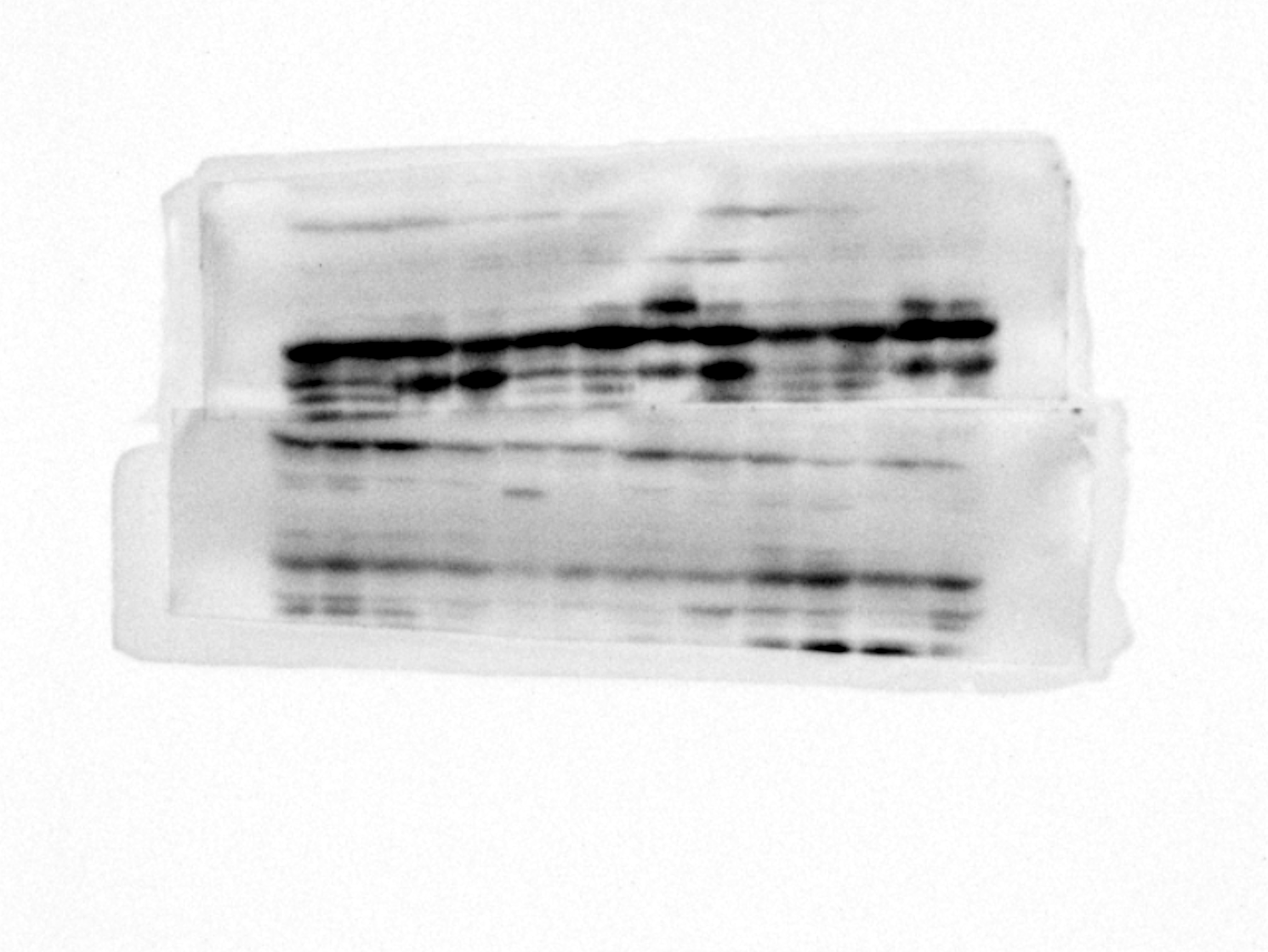

Supplement: Supplementary file 8 [file DataSheet6.ZIP › WB2/p-p38(43) 上1,2 下3 liver (3).tif]

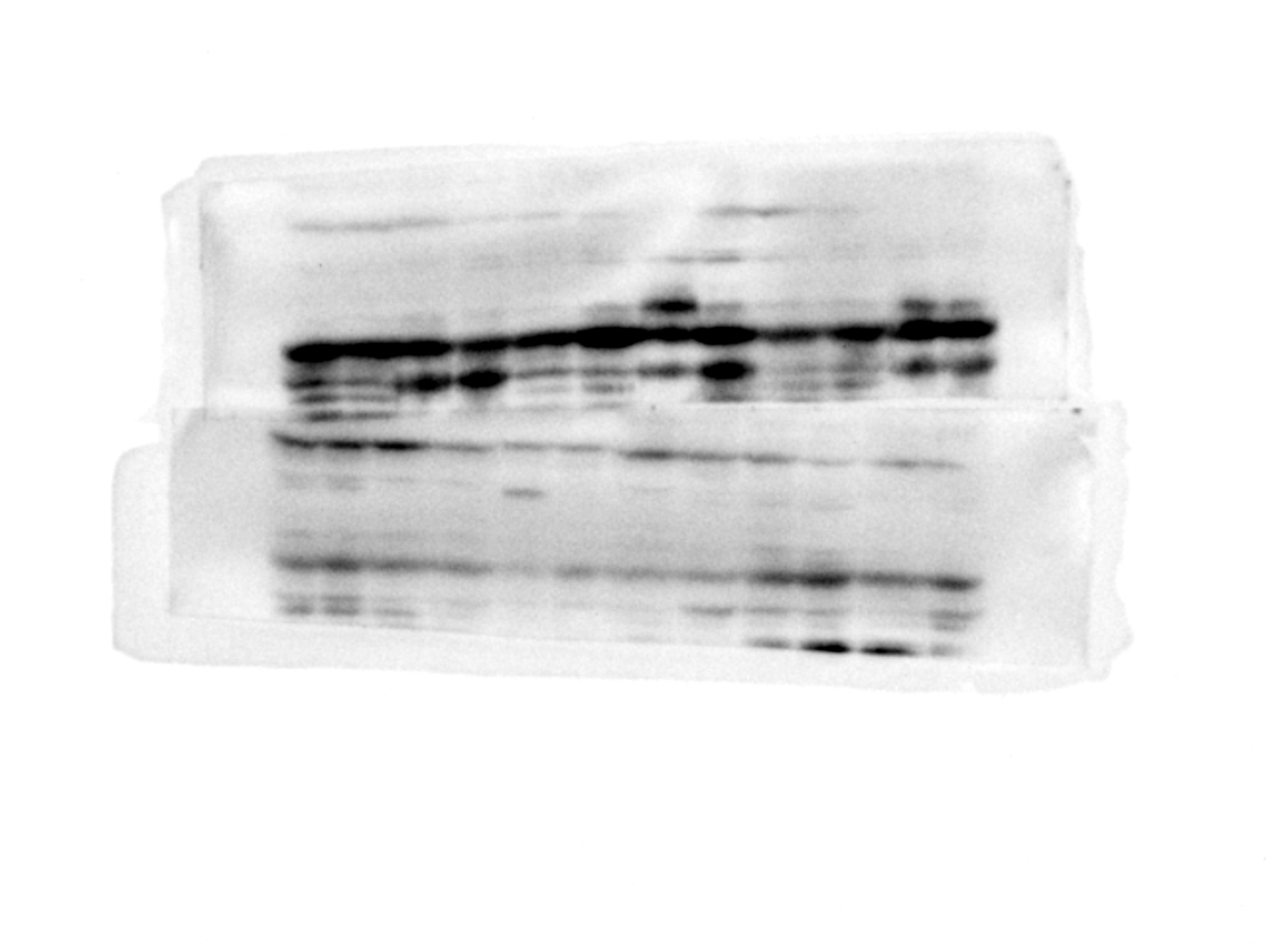

Supplement: Supplementary file 8 [file DataSheet6.ZIP › WB2/p-p38(43) 上1,2 下3 liver (5).tif]

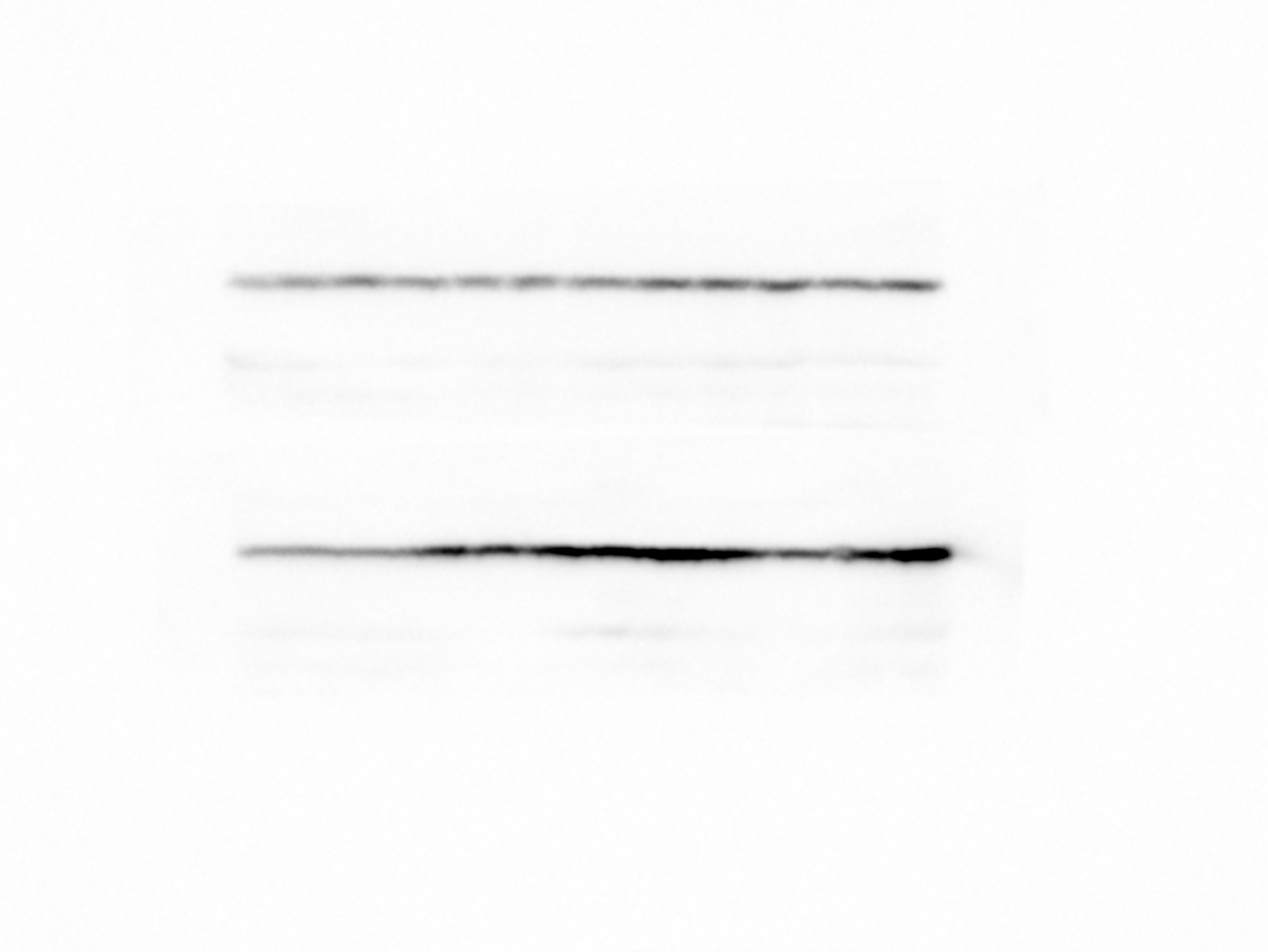

Supplement: Supplementary file 8 [file DataSheet6.ZIP › WB2/p38(45) 上3 下1,2 liver.tif]

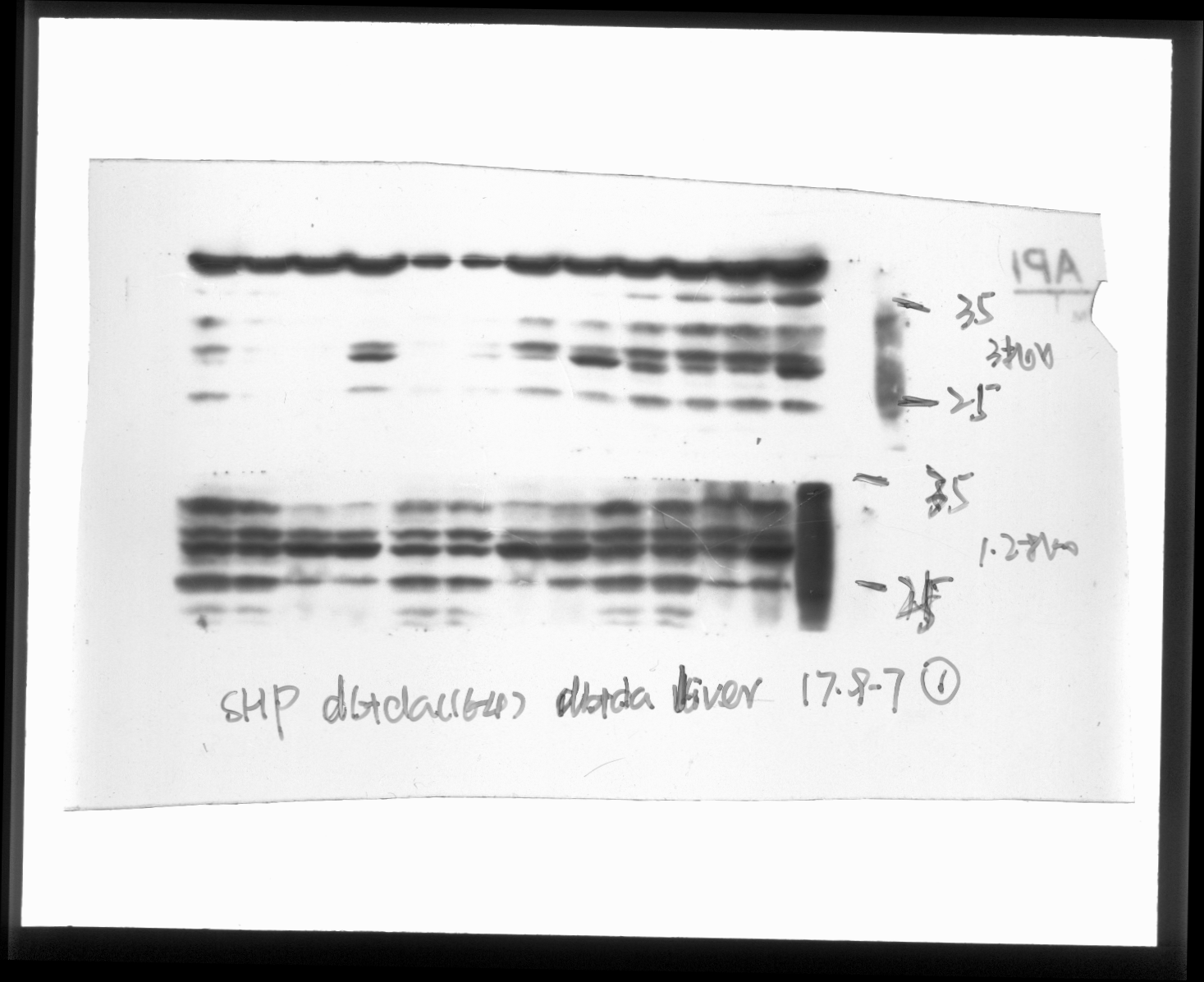

Supplement: Supplementary file 8 [file DataSheet6.ZIP › WB2/SHP 1,2 3批 liver (6).tif]

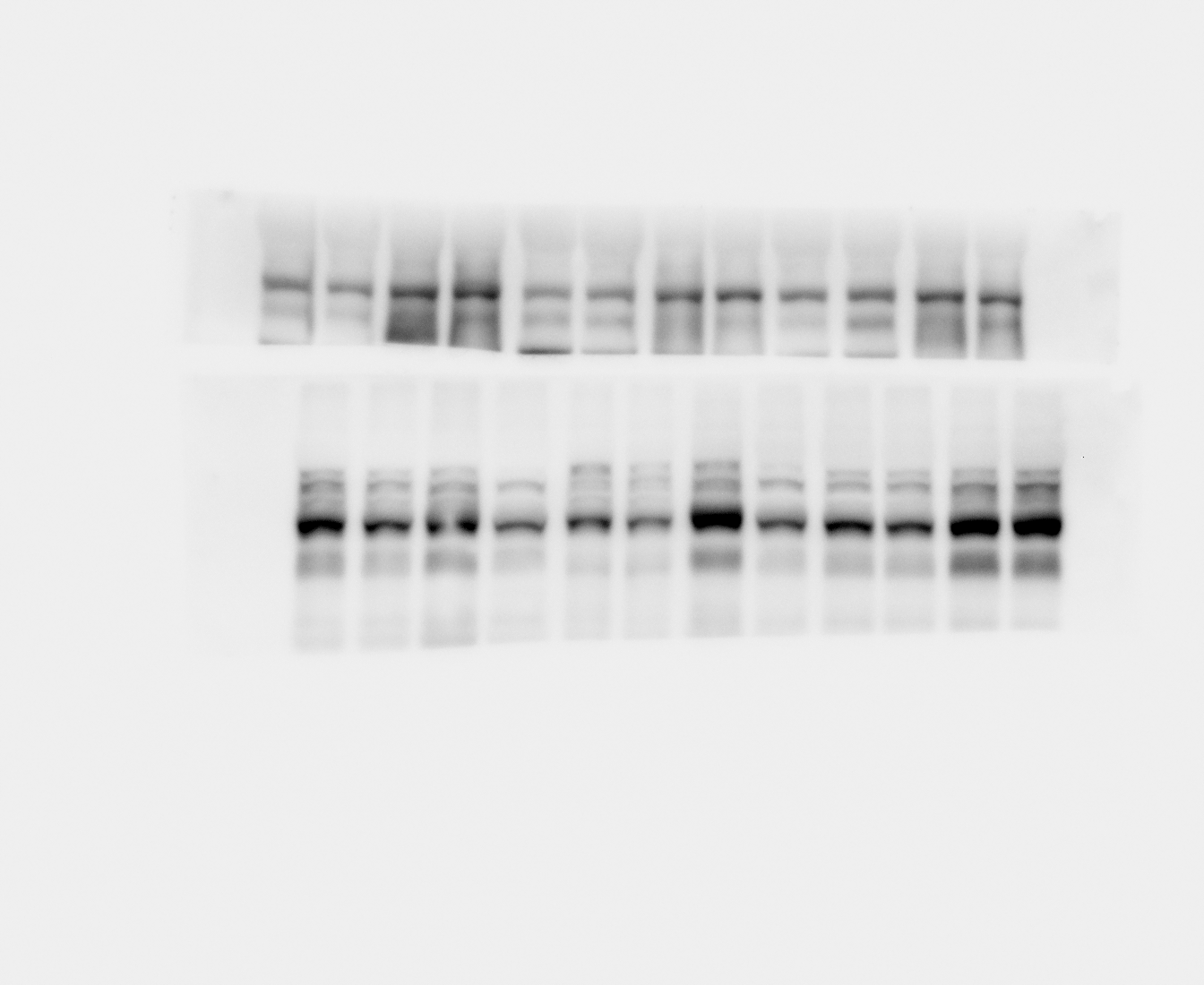

Supplement: Supplementary file 8 [file DataSheet6.ZIP › WB2/SREBP1 liver (db+da) 上1,2批,下3批 (2).tif]

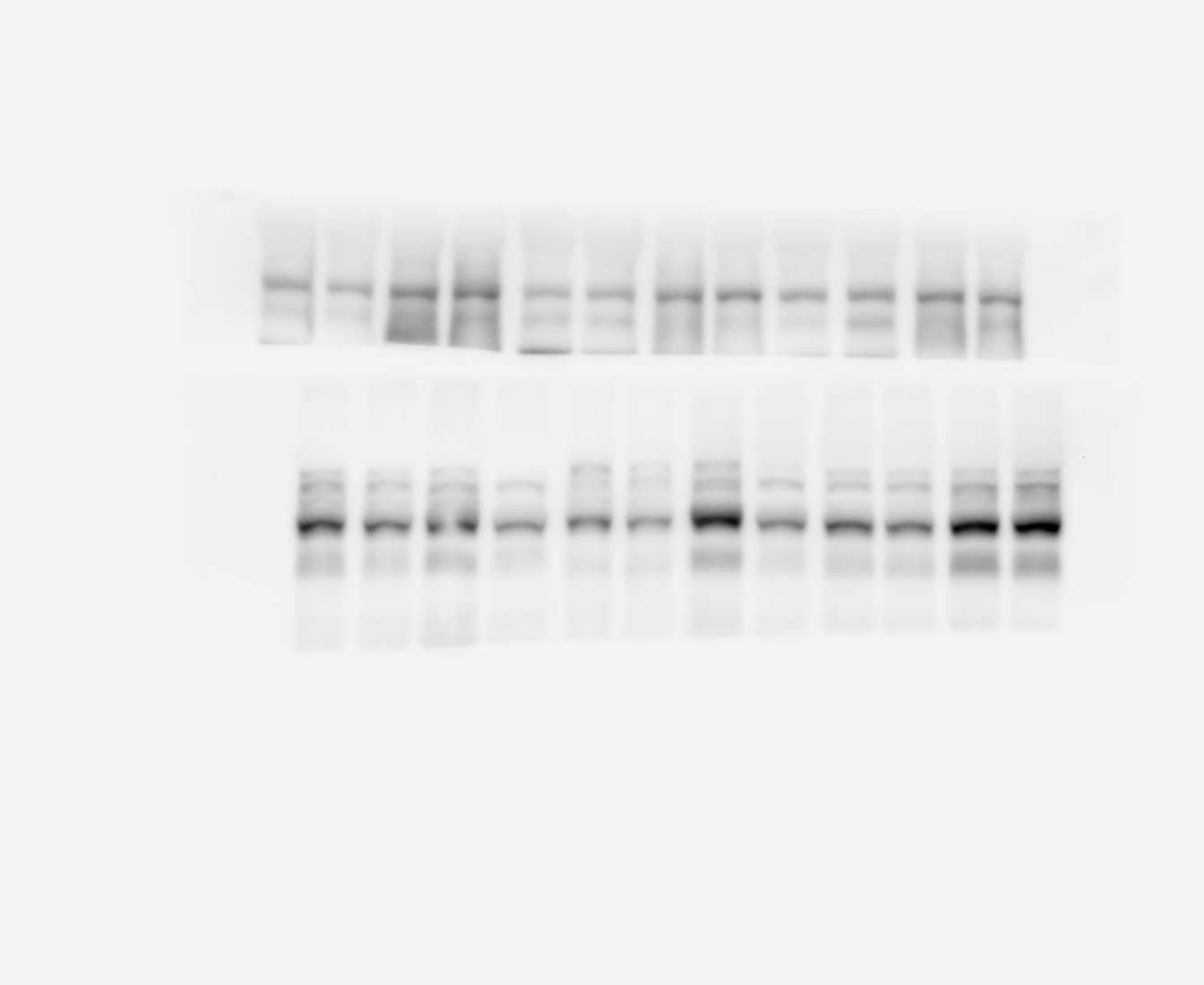

Supplement: Supplementary file 8 [file DataSheet6.ZIP › WB2/SREBP1 liver (db+da) 上1,2批,下3批 (3).tif]

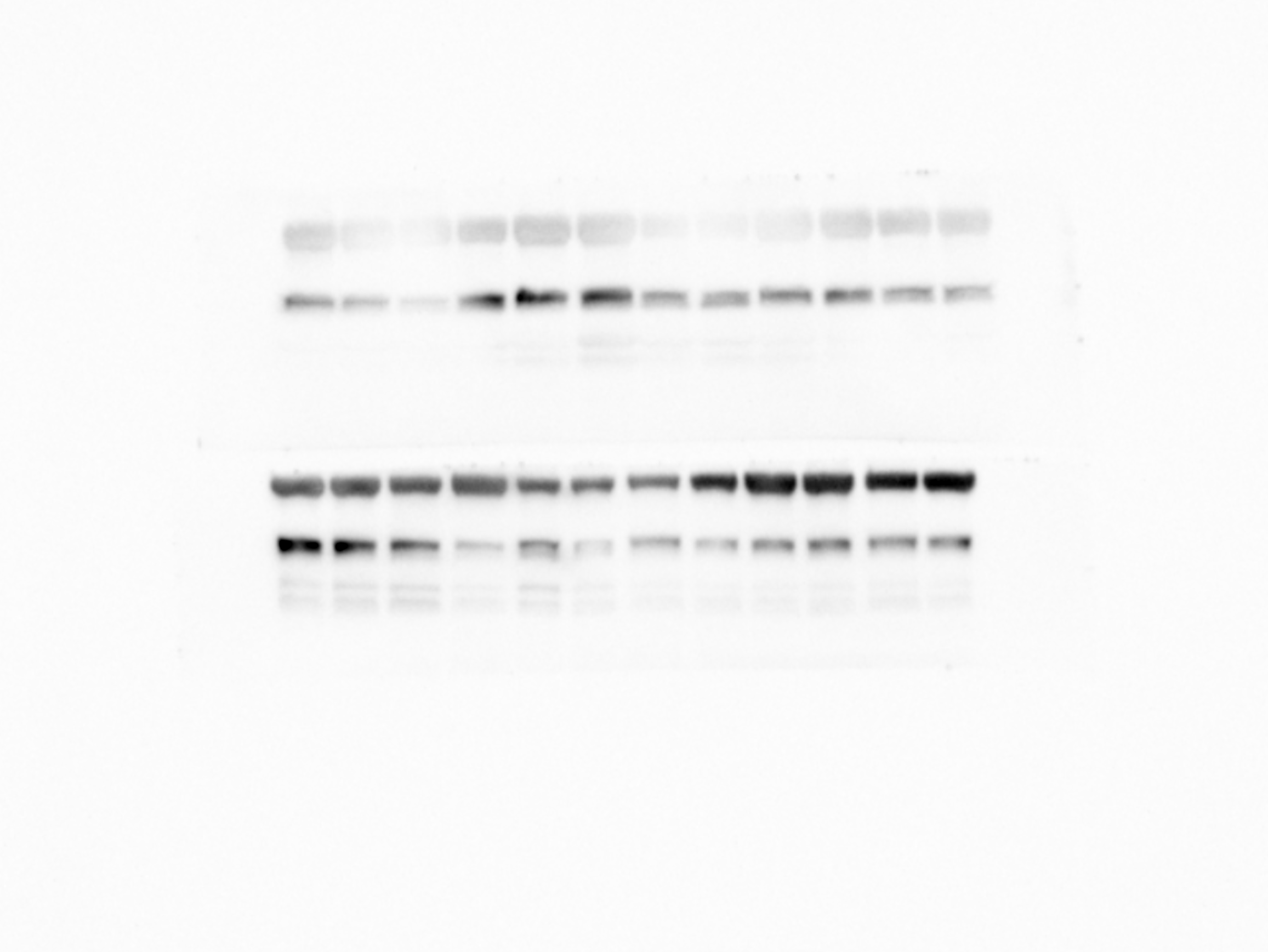

Supplement: Supplementary file 8 [file DataSheet6.ZIP › WB2/stat3(79,86) liver上1,2 下3 (10).tif]

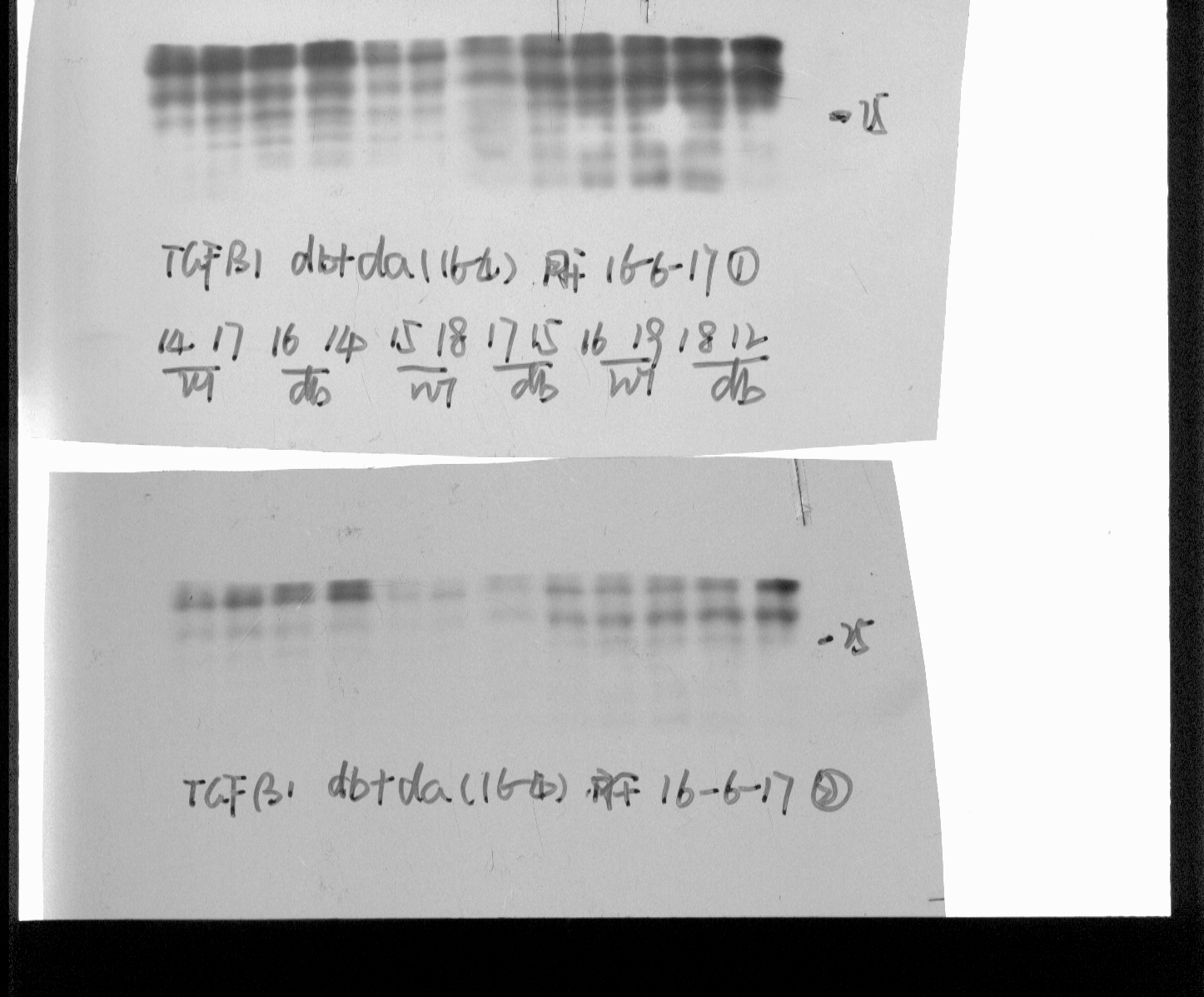

Supplement: Supplementary file 8 [file DataSheet6.ZIP › WB2/TGF-b1 2016-4.tif]

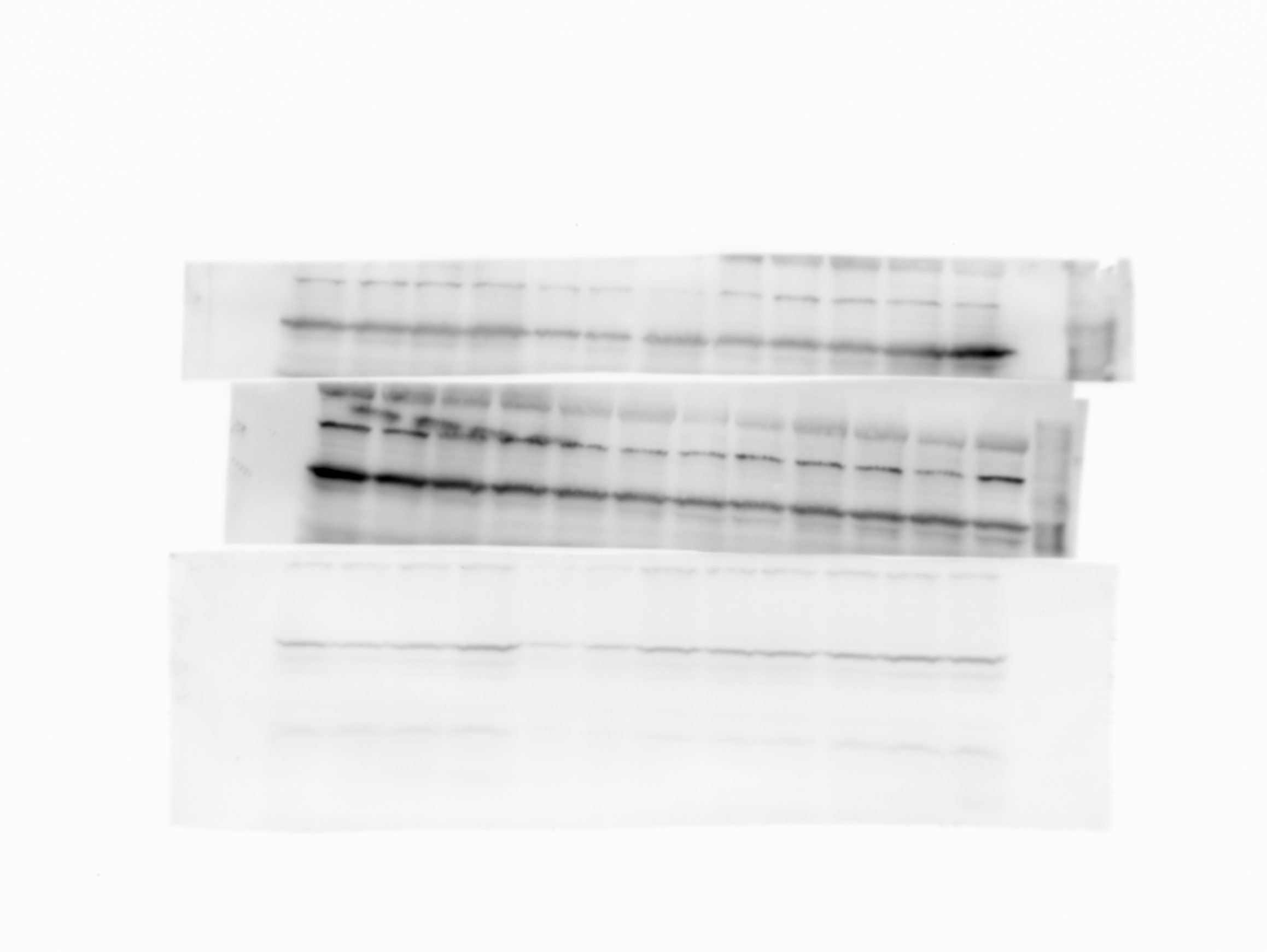

Supplement: Supplementary file 8 [file DataSheet6.ZIP › WB2/上 3 CHREBP 中 1,2 CHREBP 下 3 a-SMA (20).tif]

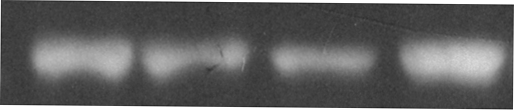

Supplement: Supplementary file 8 [file DataSheet6.ZIP › WB2/模板 2.tif]

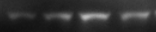

Supplement: Supplementary file 8 [file DataSheet6.ZIP › WB2/模版.tif]

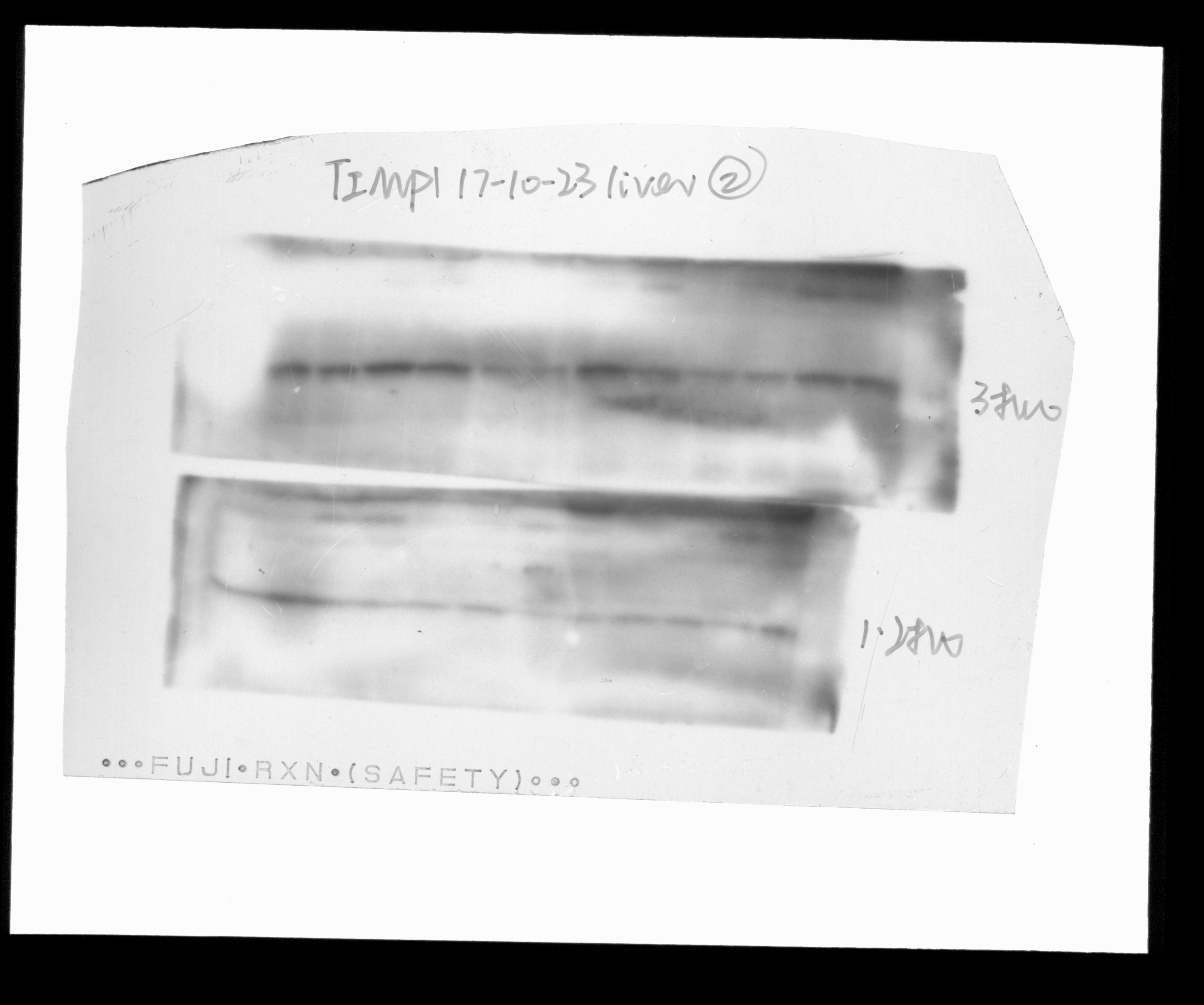

Supplement: Supplementary file 11 [file DataSheet5.ZIP › WB1/1 3 TIMP1 (3).tif]

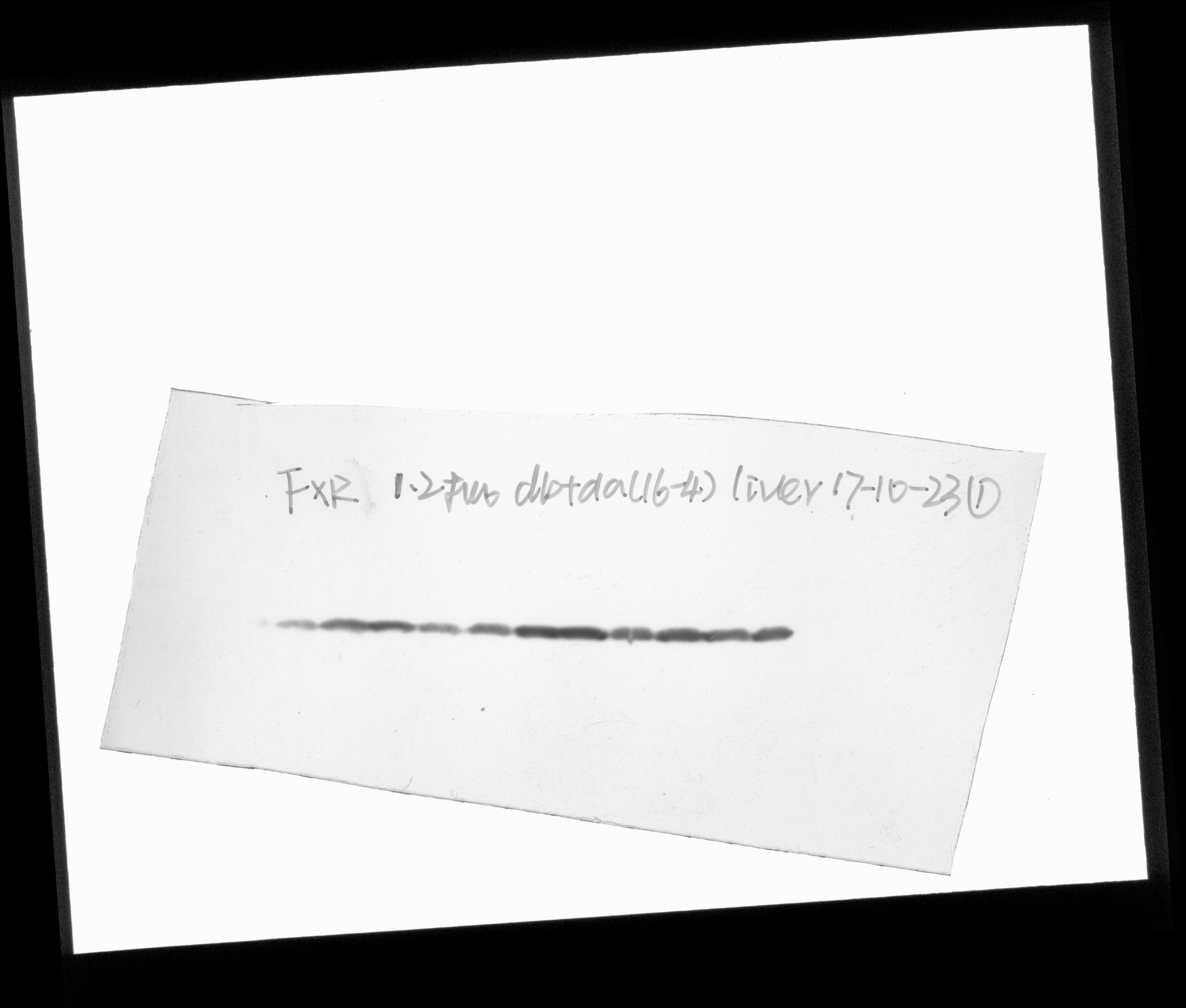

Supplement: Supplementary file 11 [file DataSheet5.ZIP › WB1/1 FXR (1).tif]

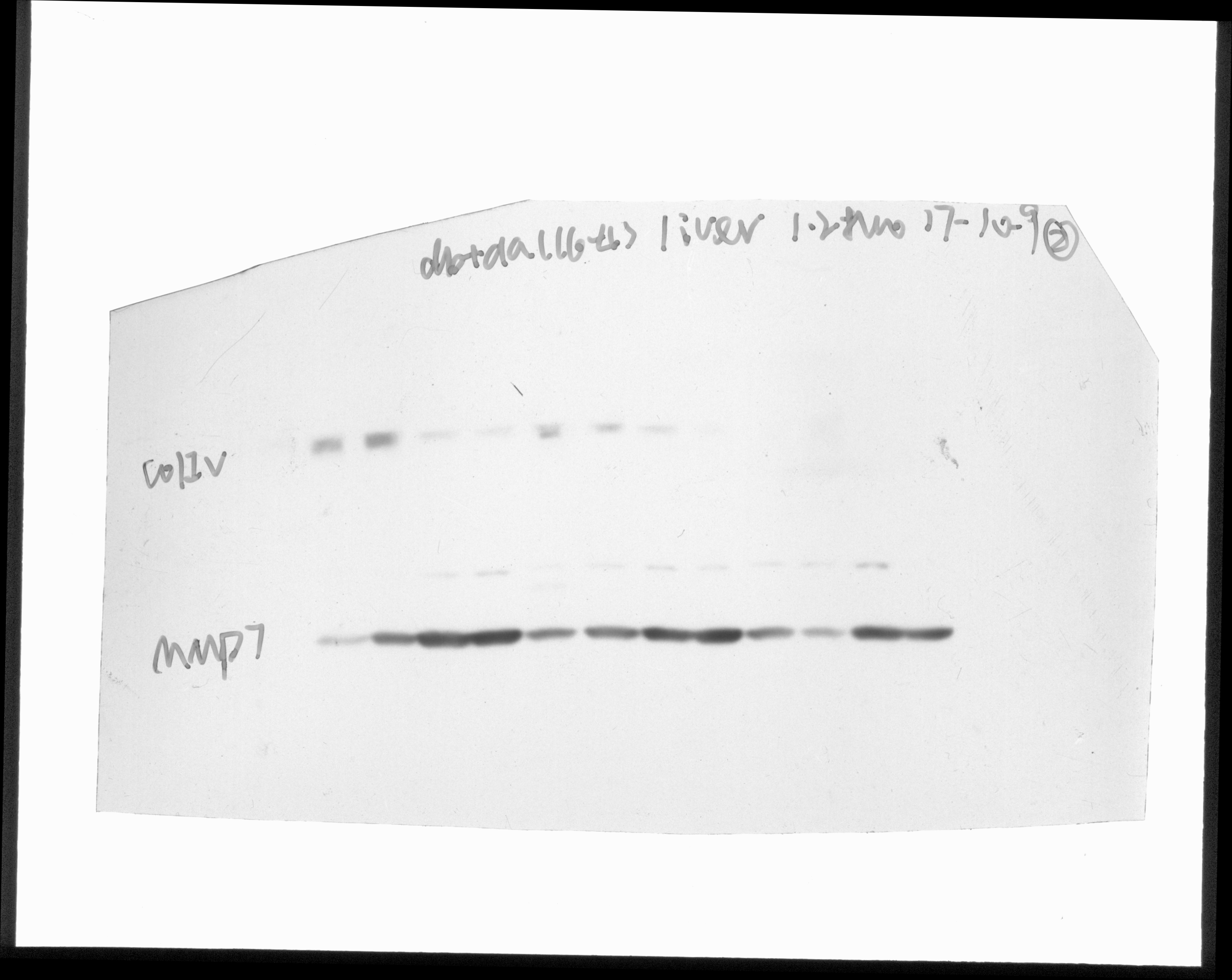

Supplement: Supplementary file 11 [file DataSheet5.ZIP › WB1/1,2 ColIV MMP7 (3).tif]

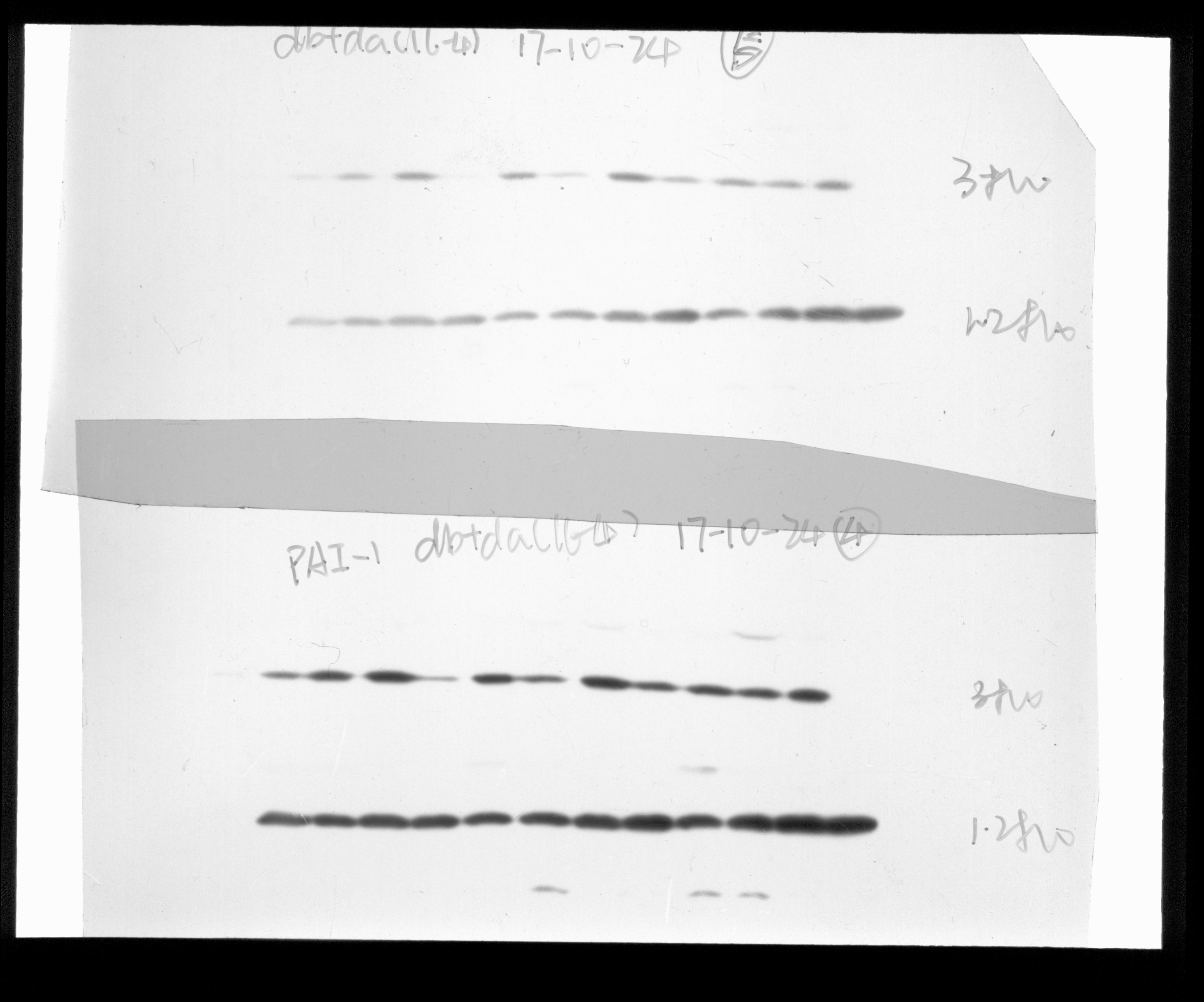

Supplement: Supplementary file 11 [file DataSheet5.ZIP › WB1/1,2 PAI-1 (3).tif]

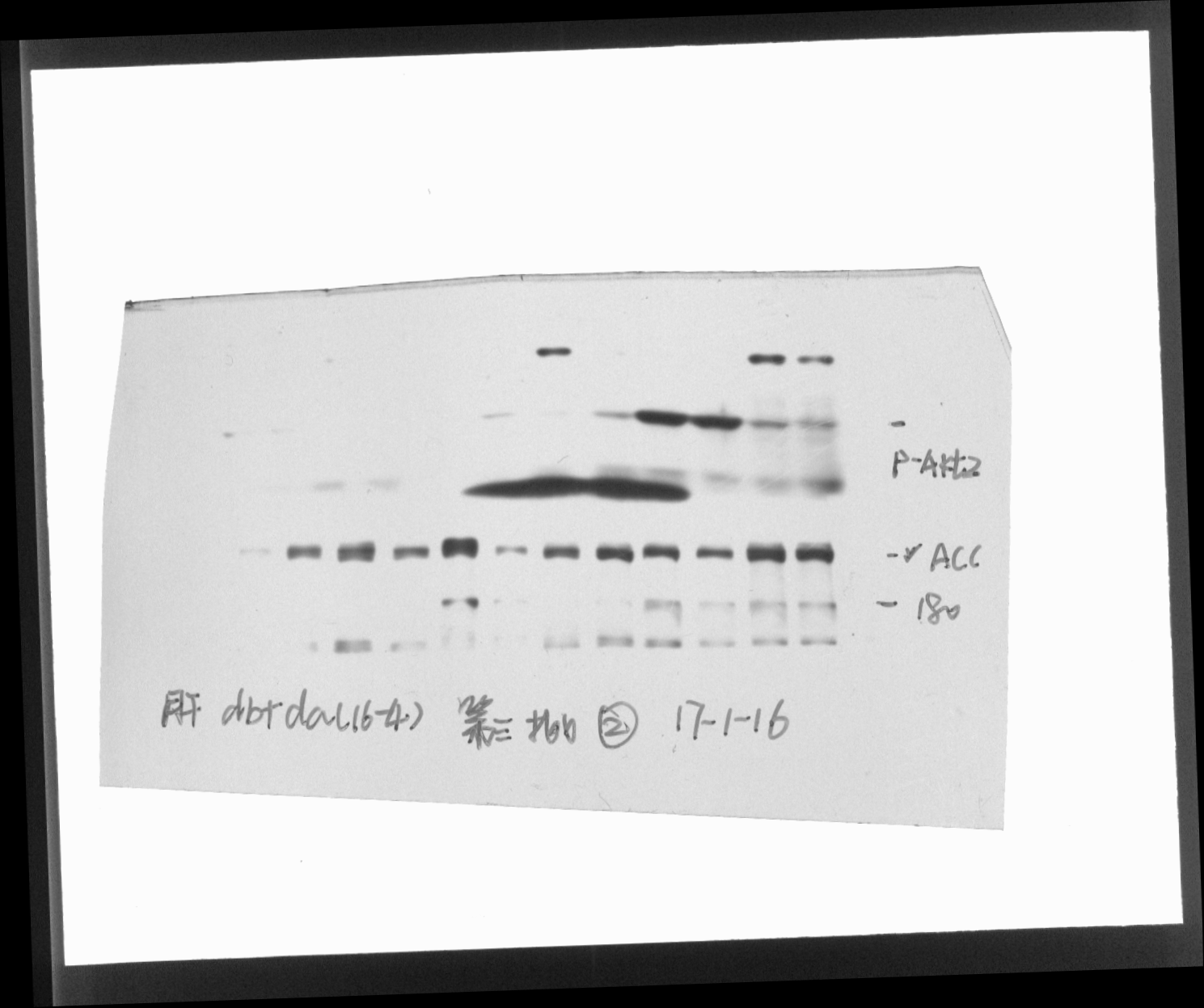

Supplement: Supplementary file 11 [file DataSheet5.ZIP › WB1/3 ACC p-AKt2 (8).tif]

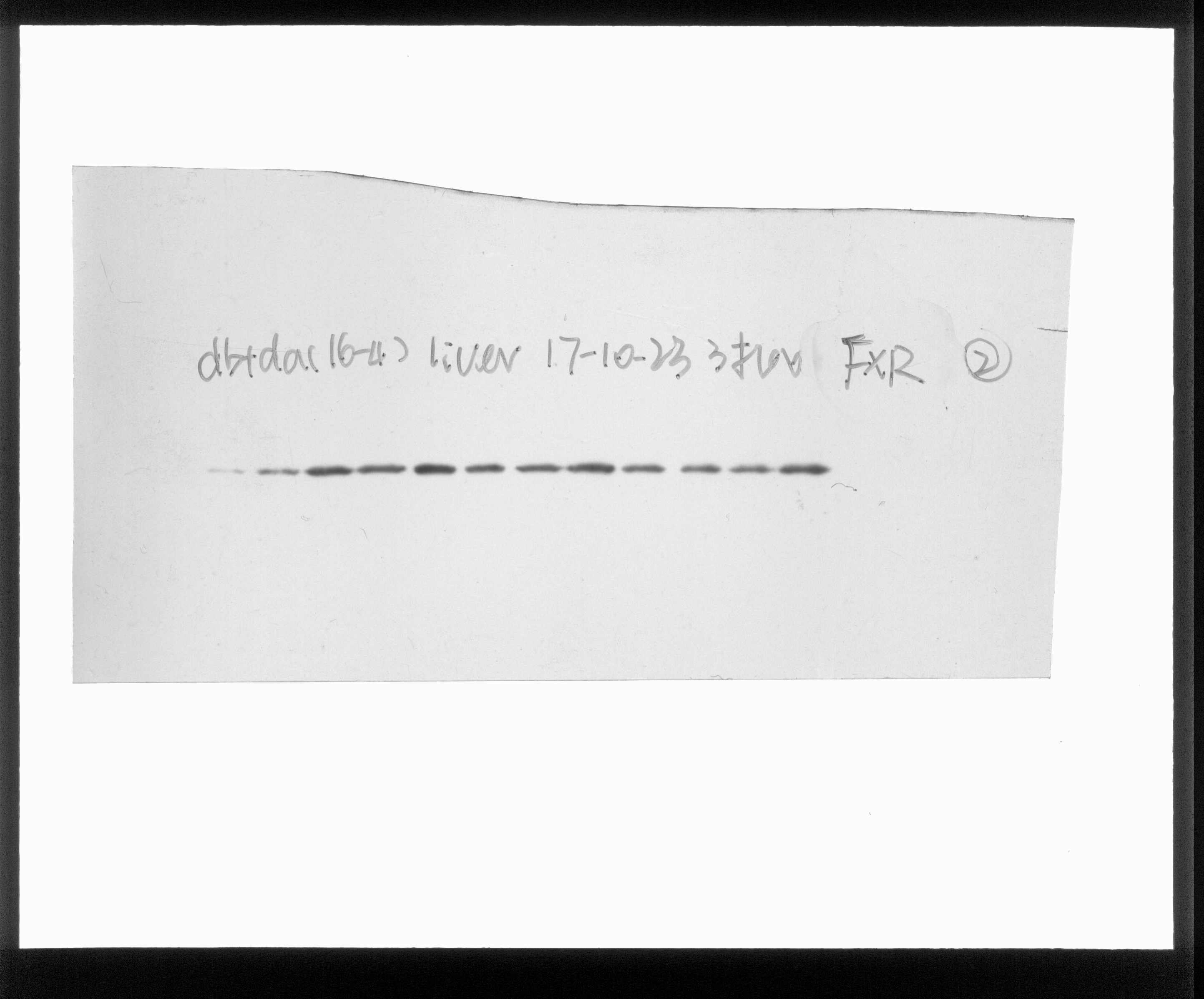

Supplement: Supplementary file 11 [file DataSheet5.ZIP › WB1/3 FXR (1).tif]

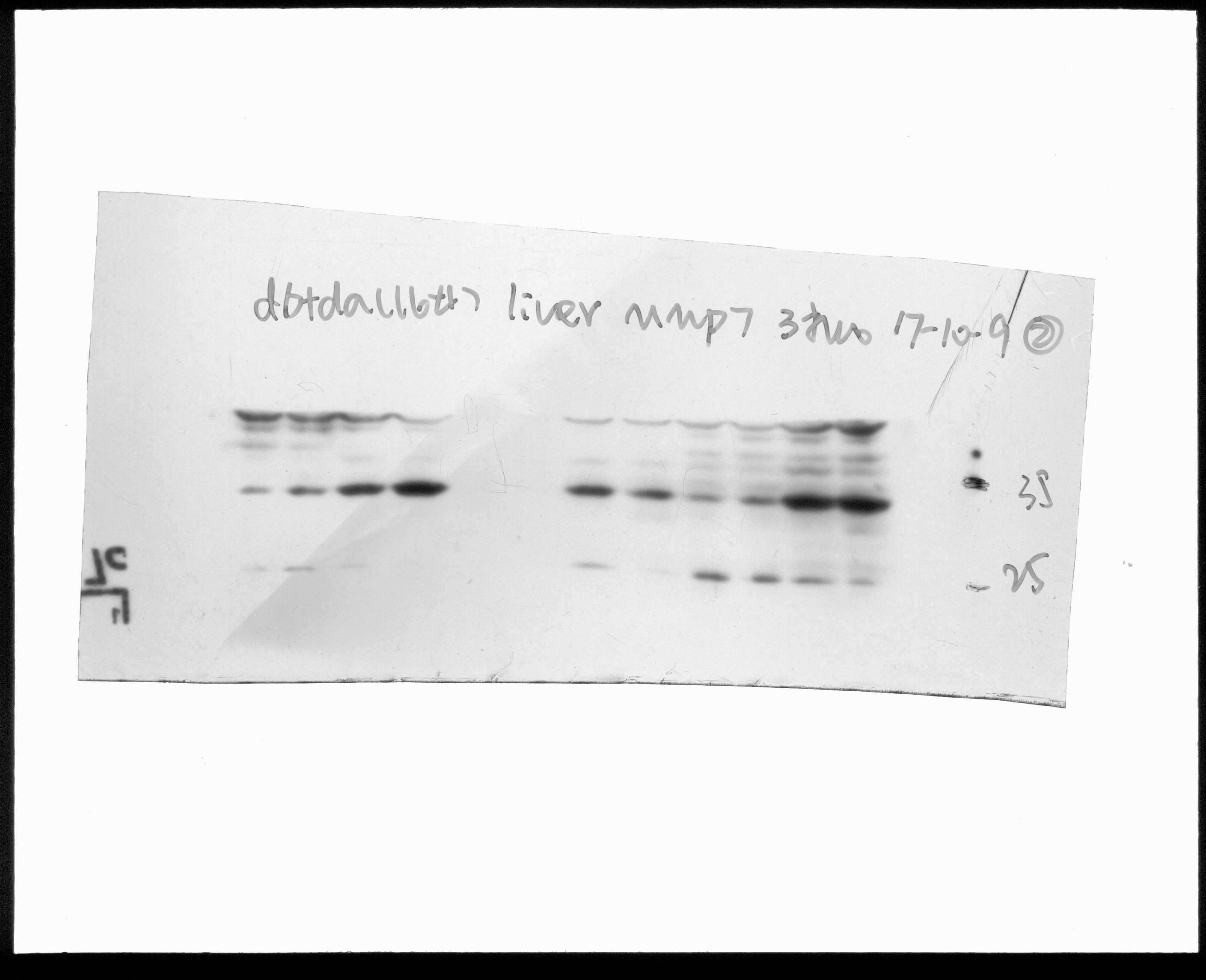

Supplement: Supplementary file 11 [file DataSheet5.ZIP › WB1/3 liver MMP7 (2).tif]

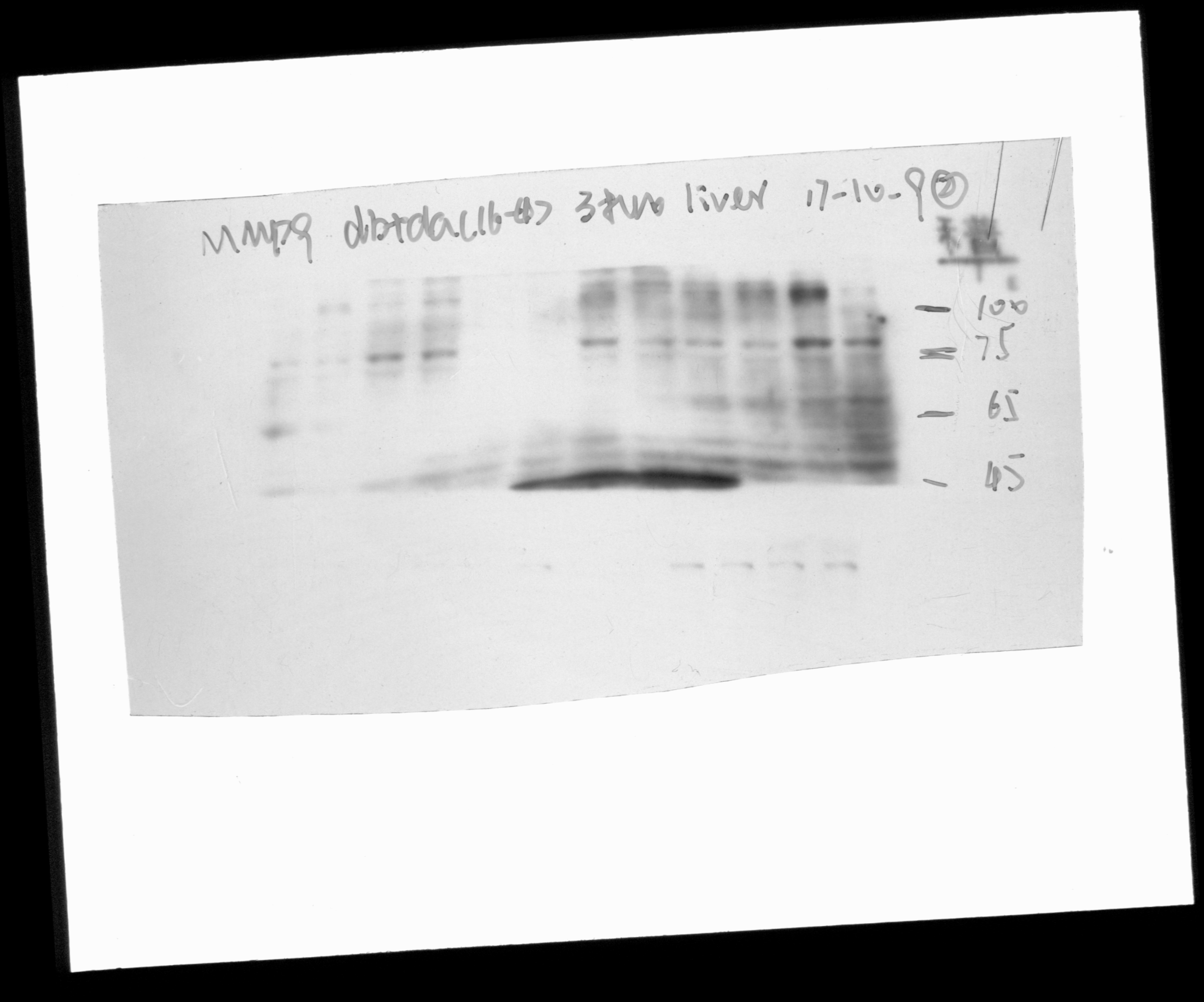

Supplement: Supplementary file 11 [file DataSheet5.ZIP › WB1/3 liver MMP9 (2).tif]

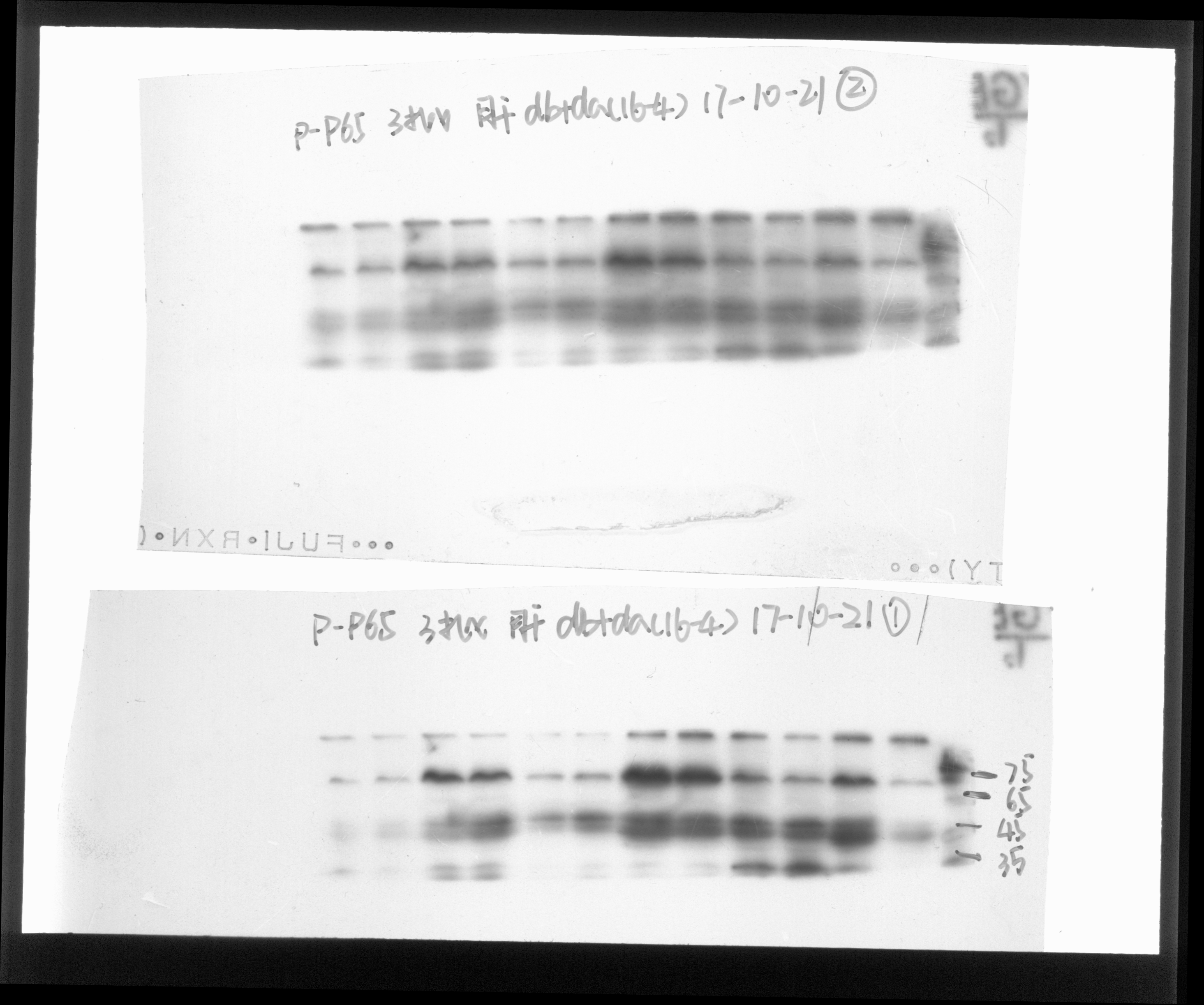

Supplement: Supplementary file 11 [file DataSheet5.ZIP › WB1/3 p-p65 (2).tif]

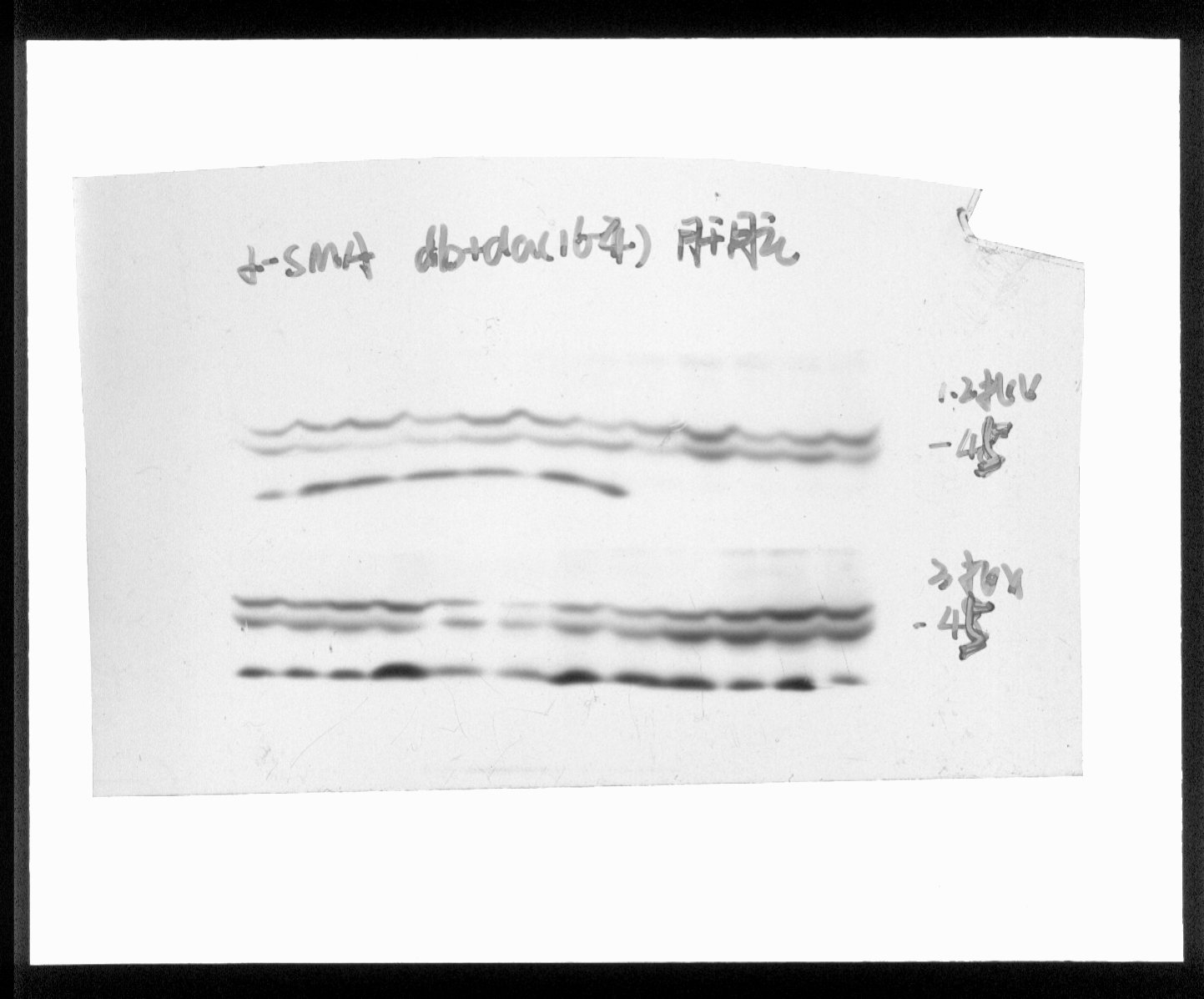

Supplement: Supplementary file 11 [file DataSheet5.ZIP › WB1/a-SMA 1,2 3 (1).tif]

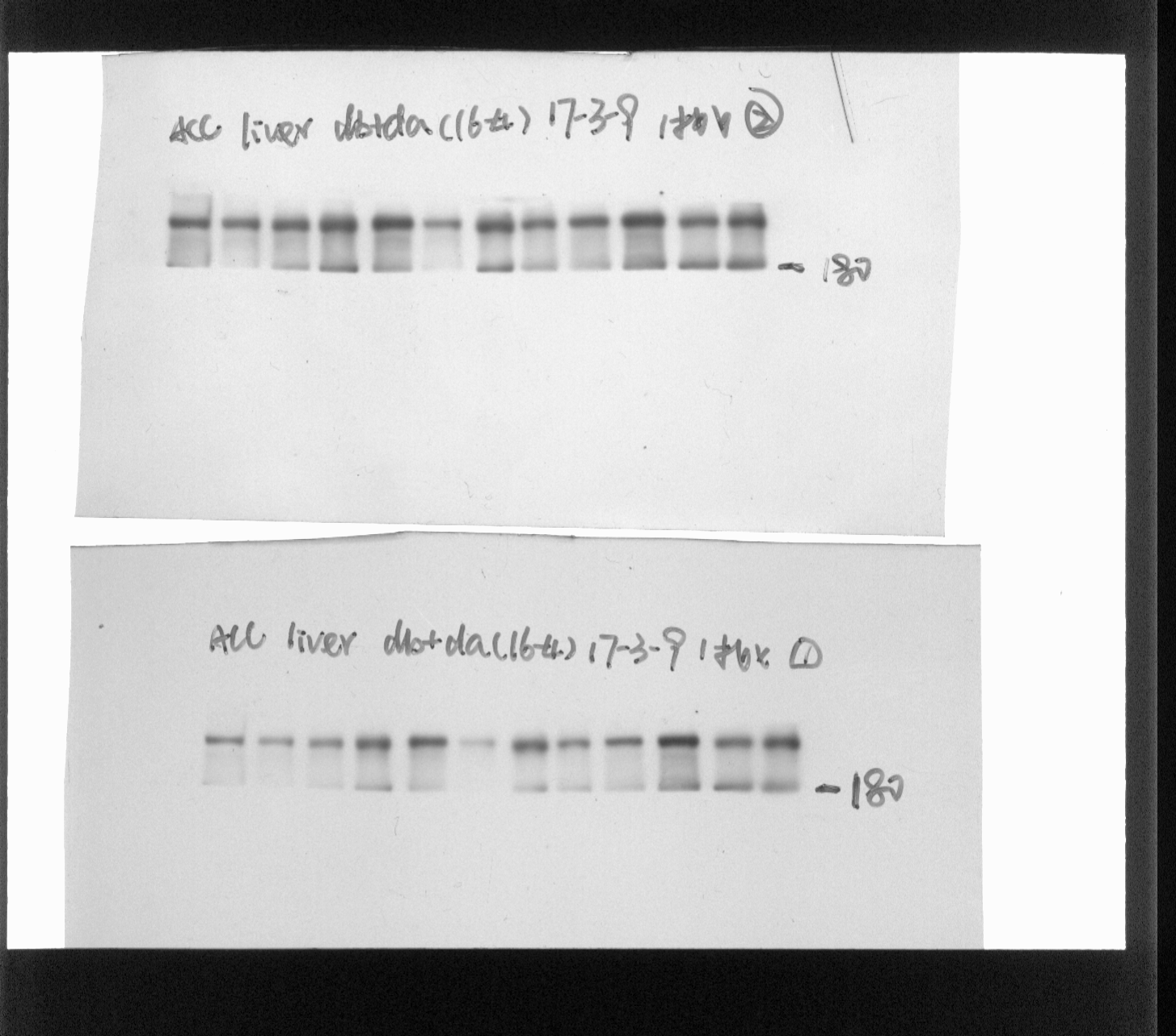

Supplement: Supplementary file 11 [file DataSheet5.ZIP › WB1/ACC liver 1.tif]

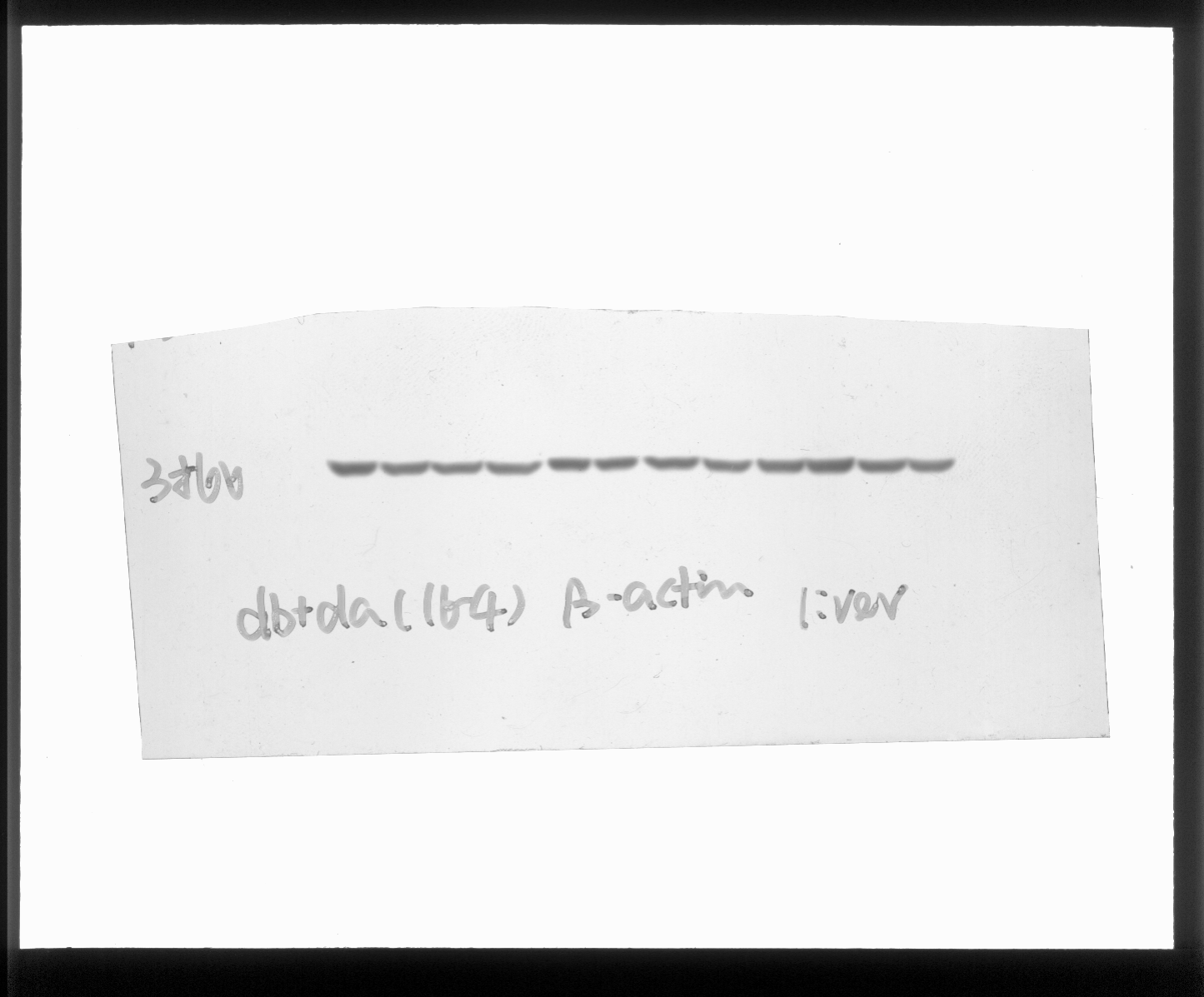

Supplement: Supplementary file 11 [file DataSheet5.ZIP › WB1/b-actin (2).tif]

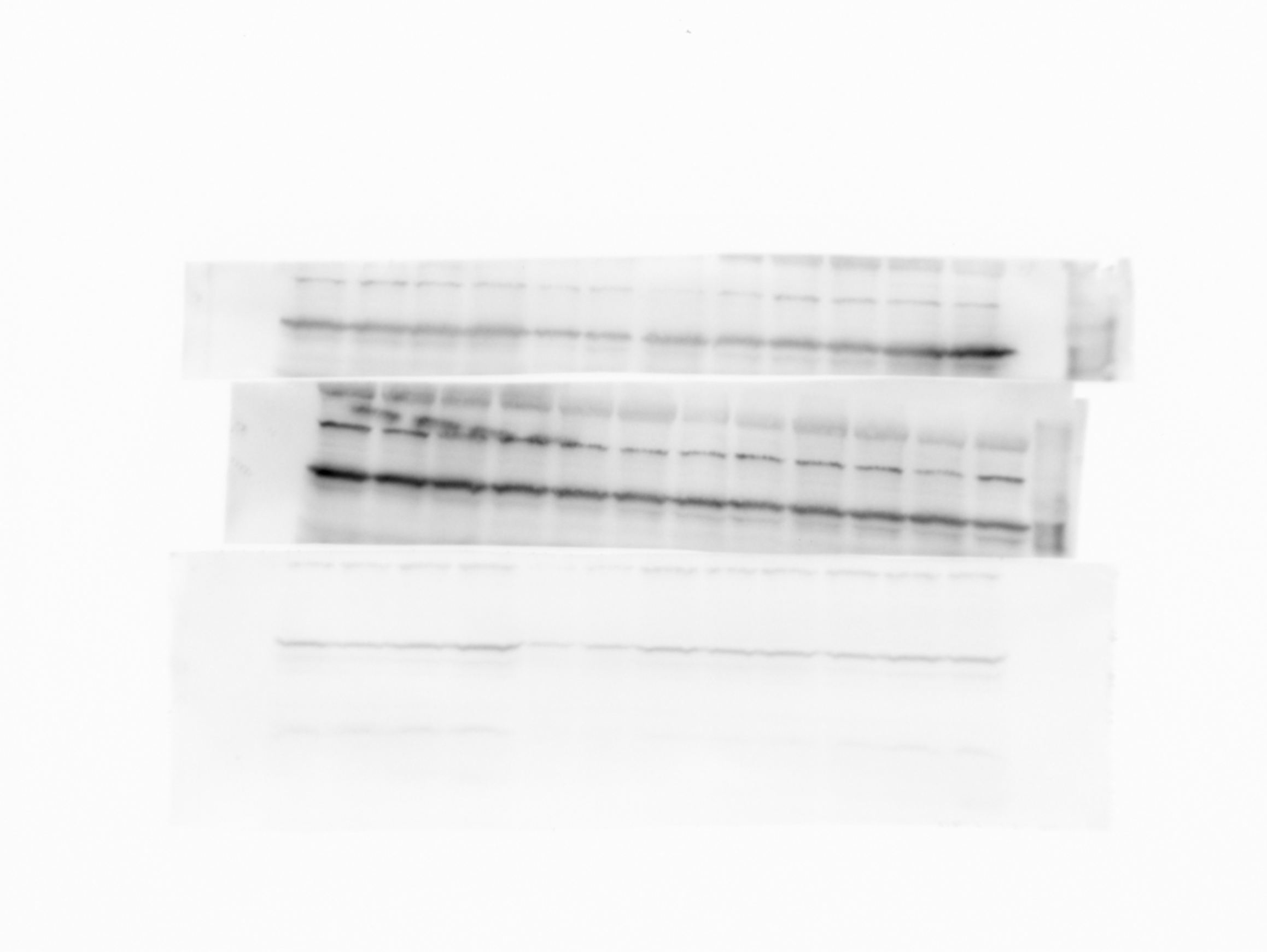

Supplement: Supplementary file 11 [file DataSheet5.ZIP › WB1/CHREBP 上3批,下1,2.tif]

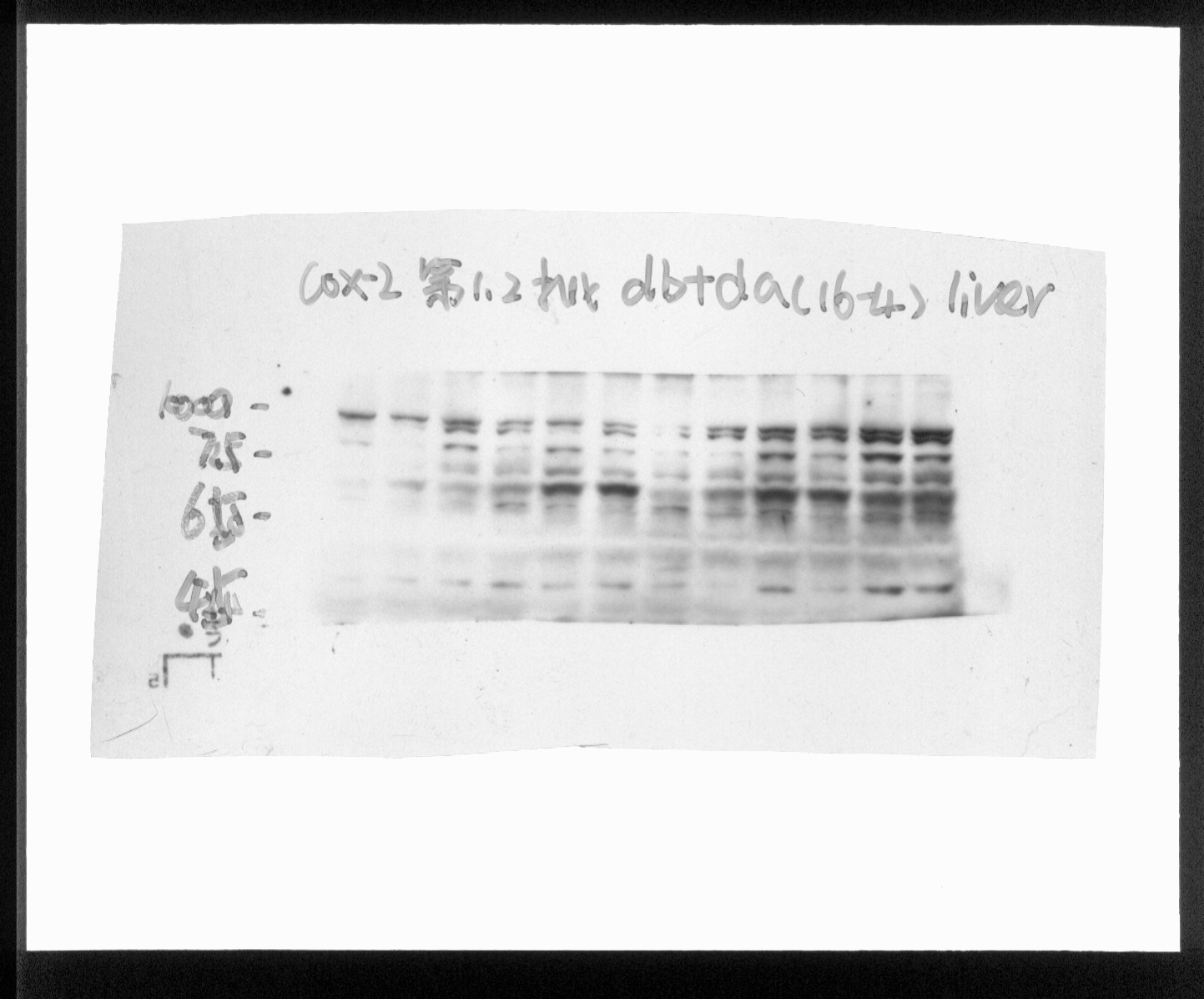

Supplement: Supplementary file 11 [file DataSheet5.ZIP › WB1/COX-2 1,2 (1).tif]

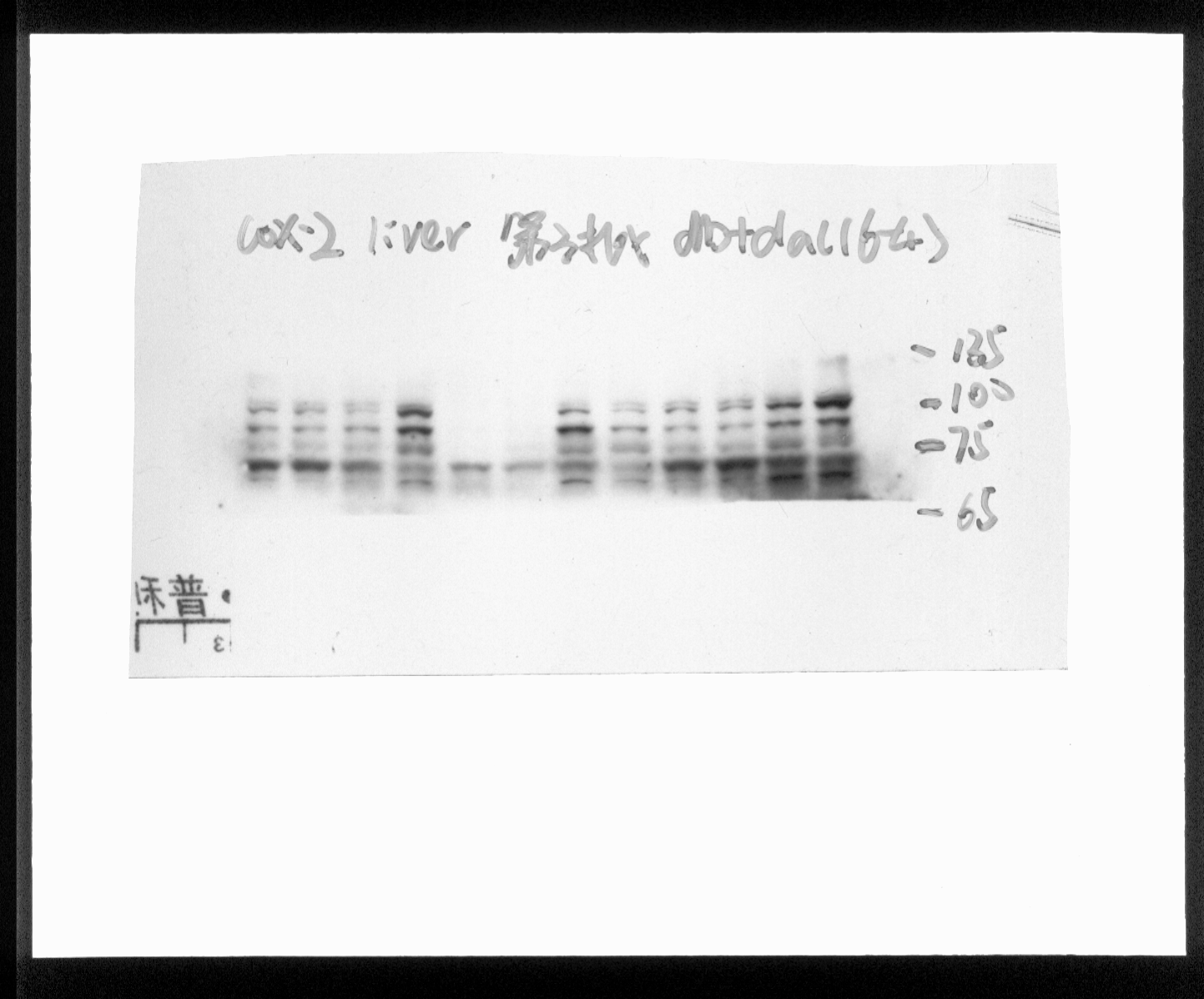

Supplement: Supplementary file 11 [file DataSheet5.ZIP › WB1/COX-2 3 (1).tif]

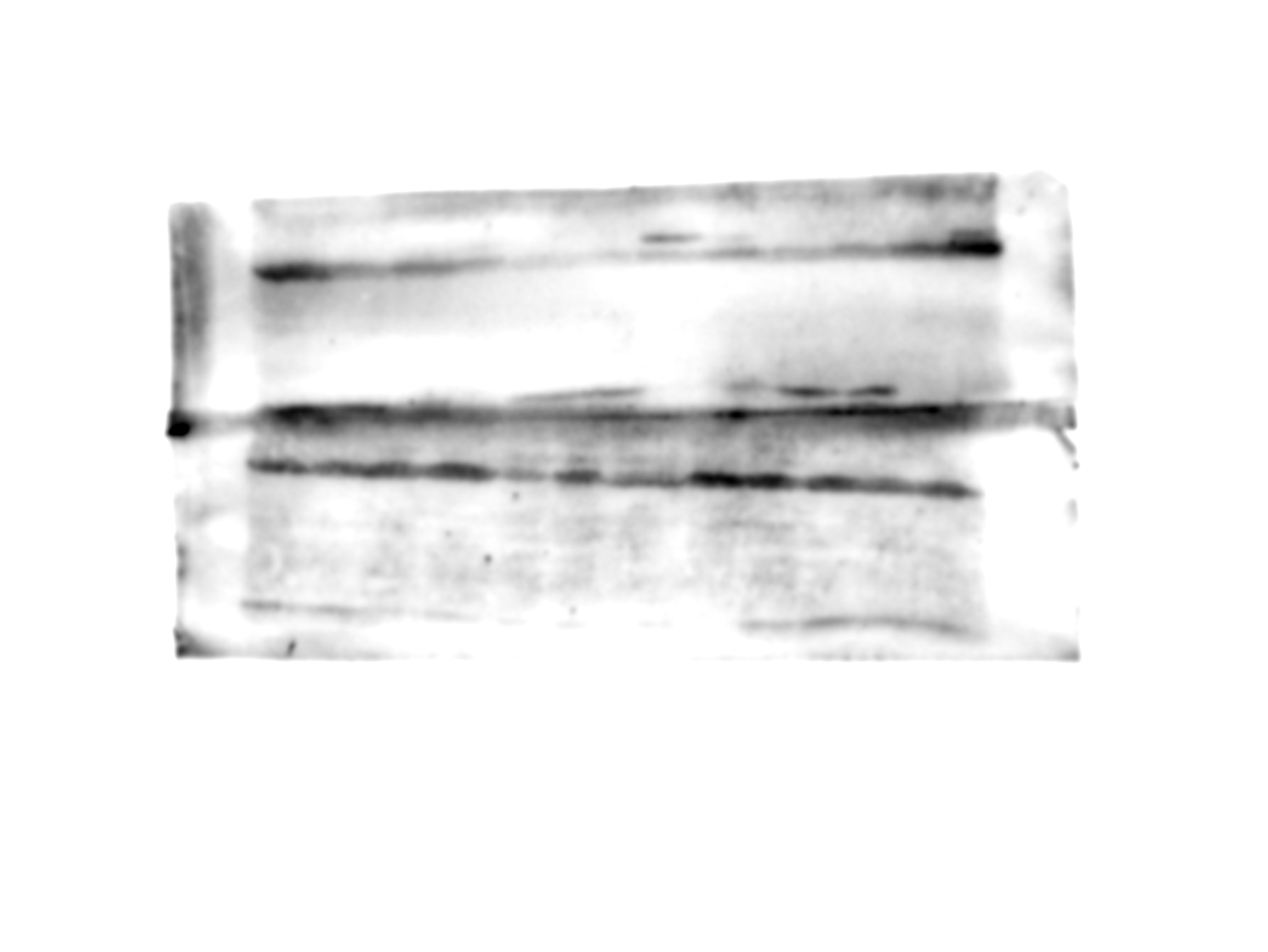

Supplement: Supplementary file 11 [file DataSheet5.ZIP › WB1/CTGF (2).tif]

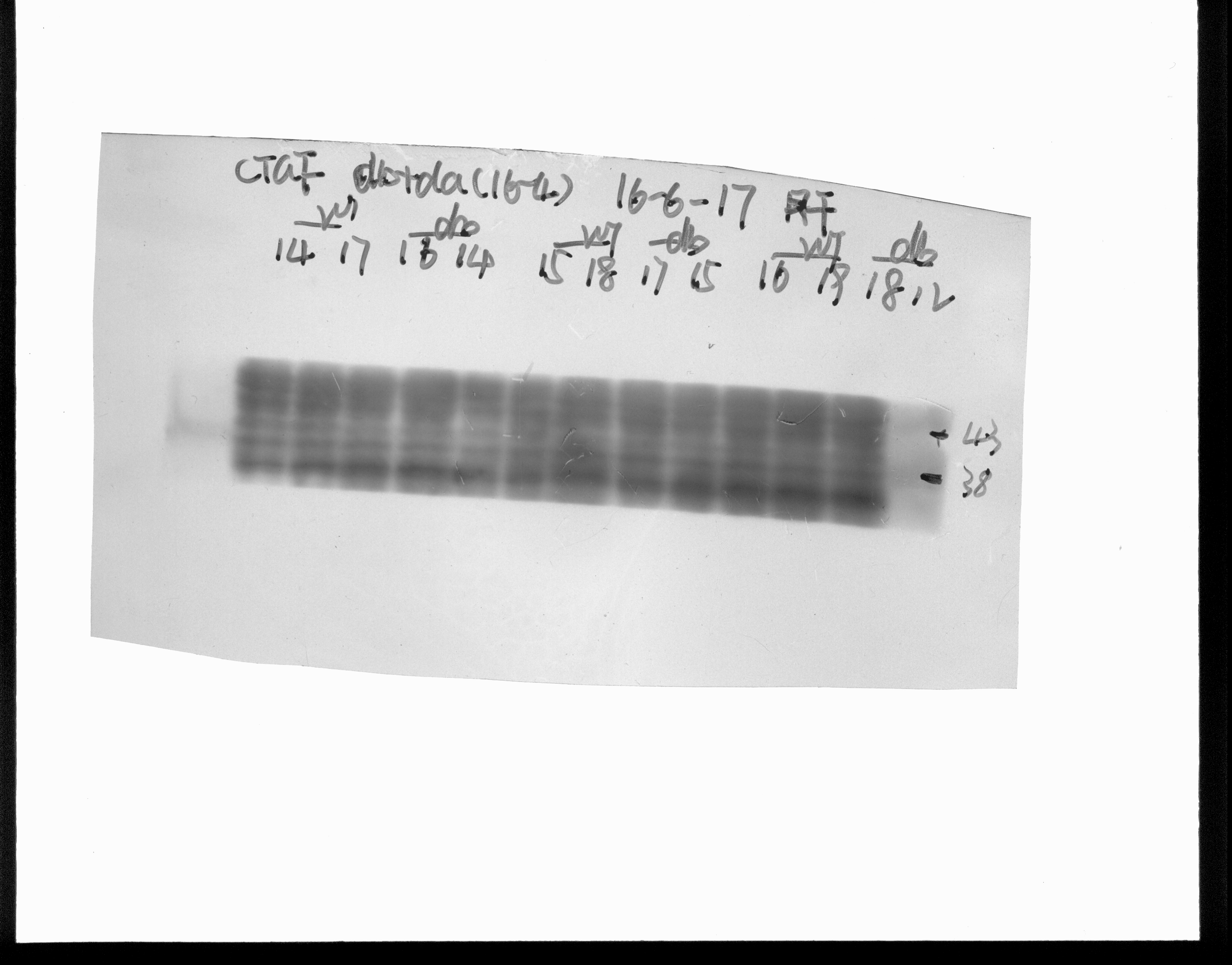

Supplement: Supplementary file 11 [file DataSheet5.ZIP › WB1/CTGF.tif]

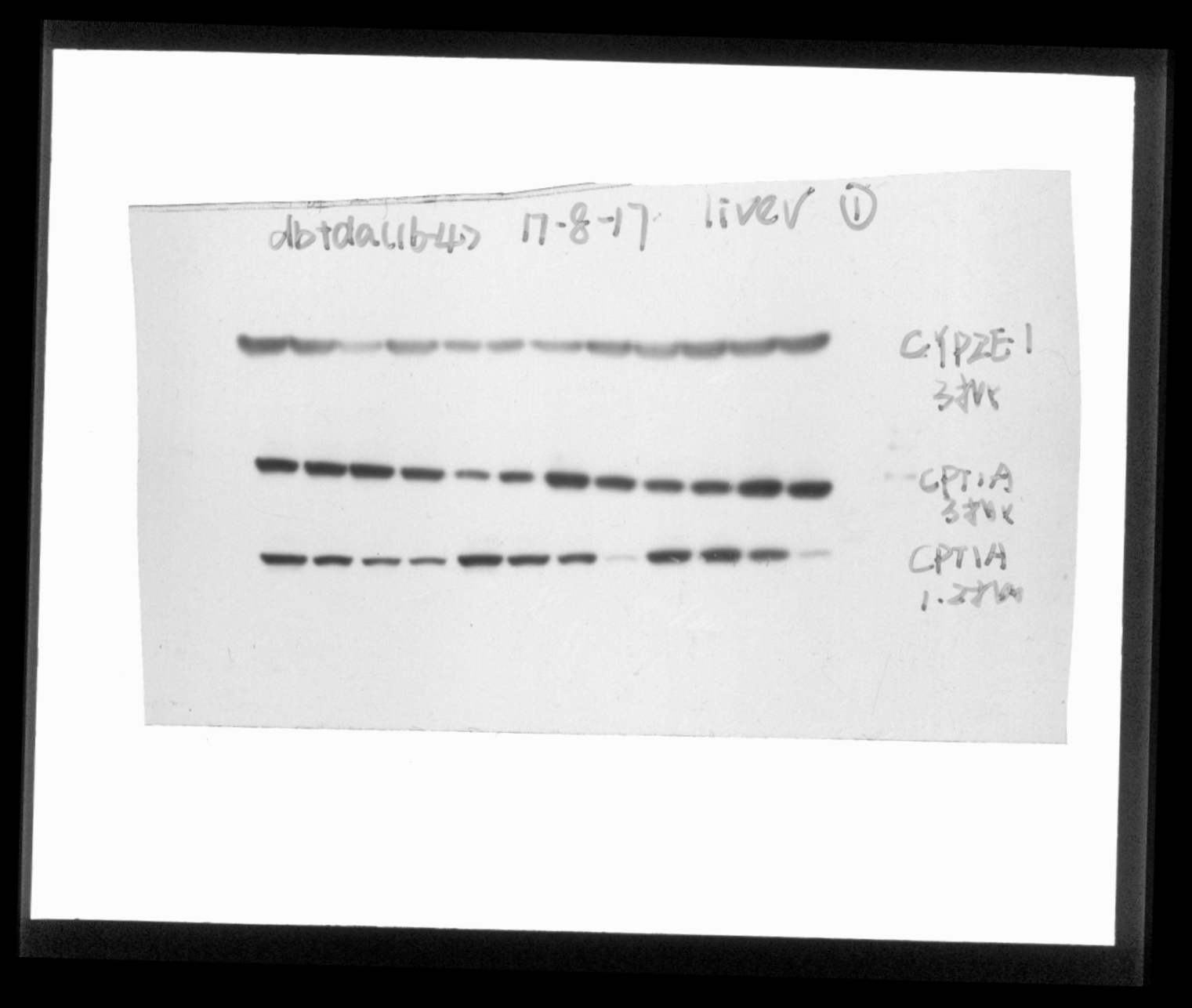

Supplement: Supplementary file 11 [file DataSheet5.ZIP › WB1/CYP2E 3 pi CPT1a 1 2,3 (2).tif]

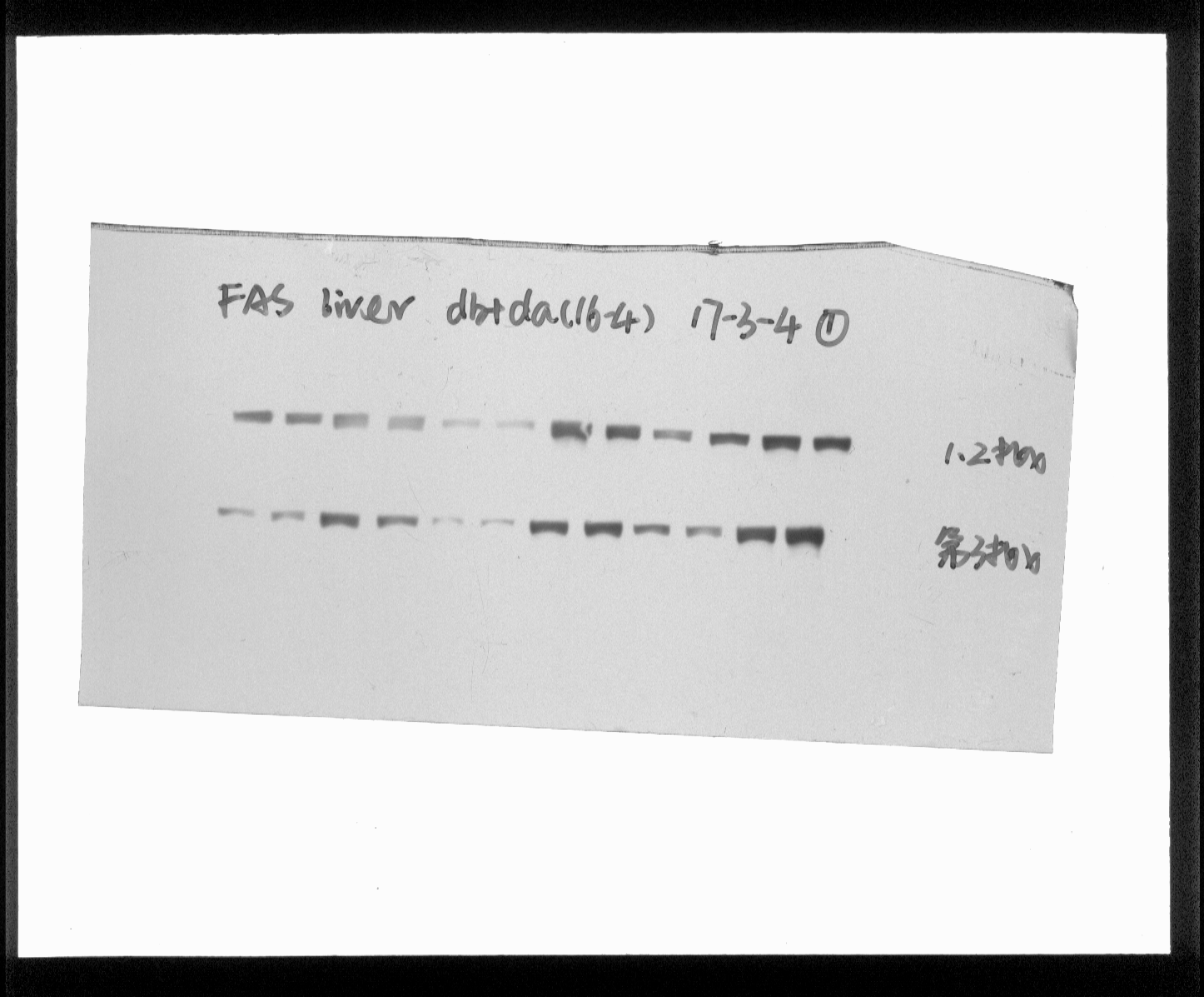

Supplement: Supplementary file 11 [file DataSheet5.ZIP › WB1/FAS 1,2批 3批 (6).tif]

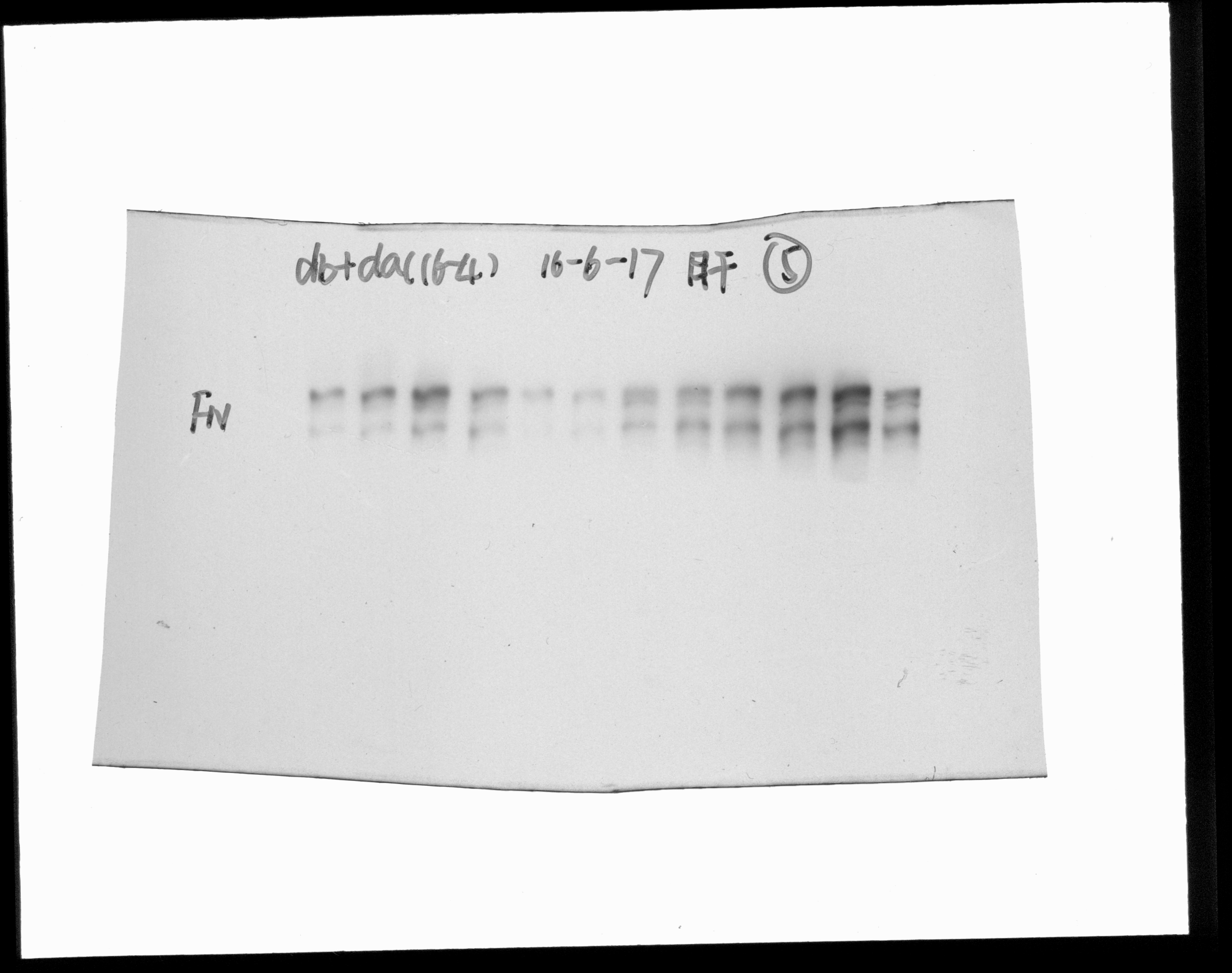

Supplement: Supplementary file 11 [file DataSheet5.ZIP › WB1/FN p-IRS1 p-Gsk3b (8).tif]

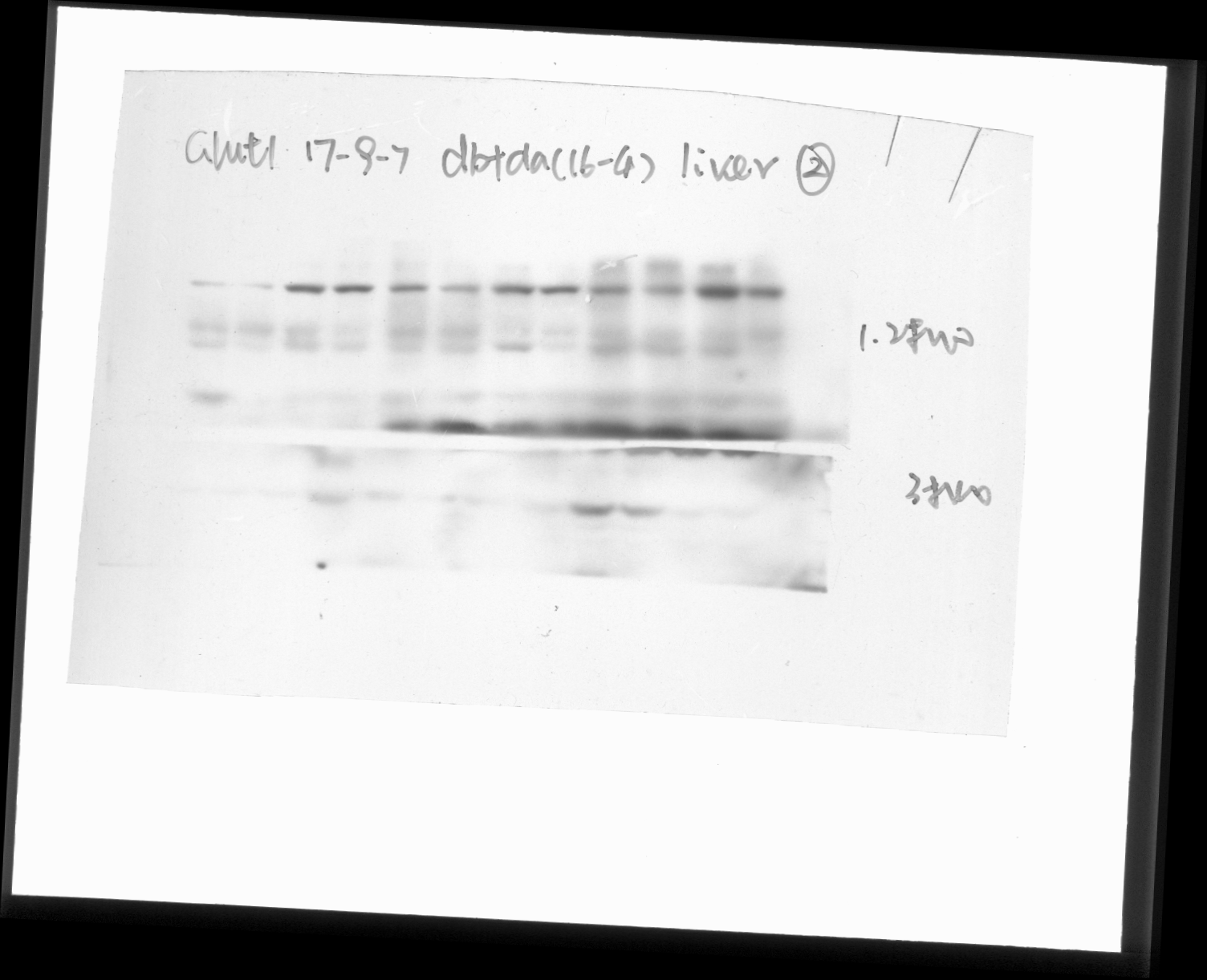

Supplement: Supplementary file 11 [file DataSheet5.ZIP › WB1/glut1 1,2 3批,liver (7).tif]

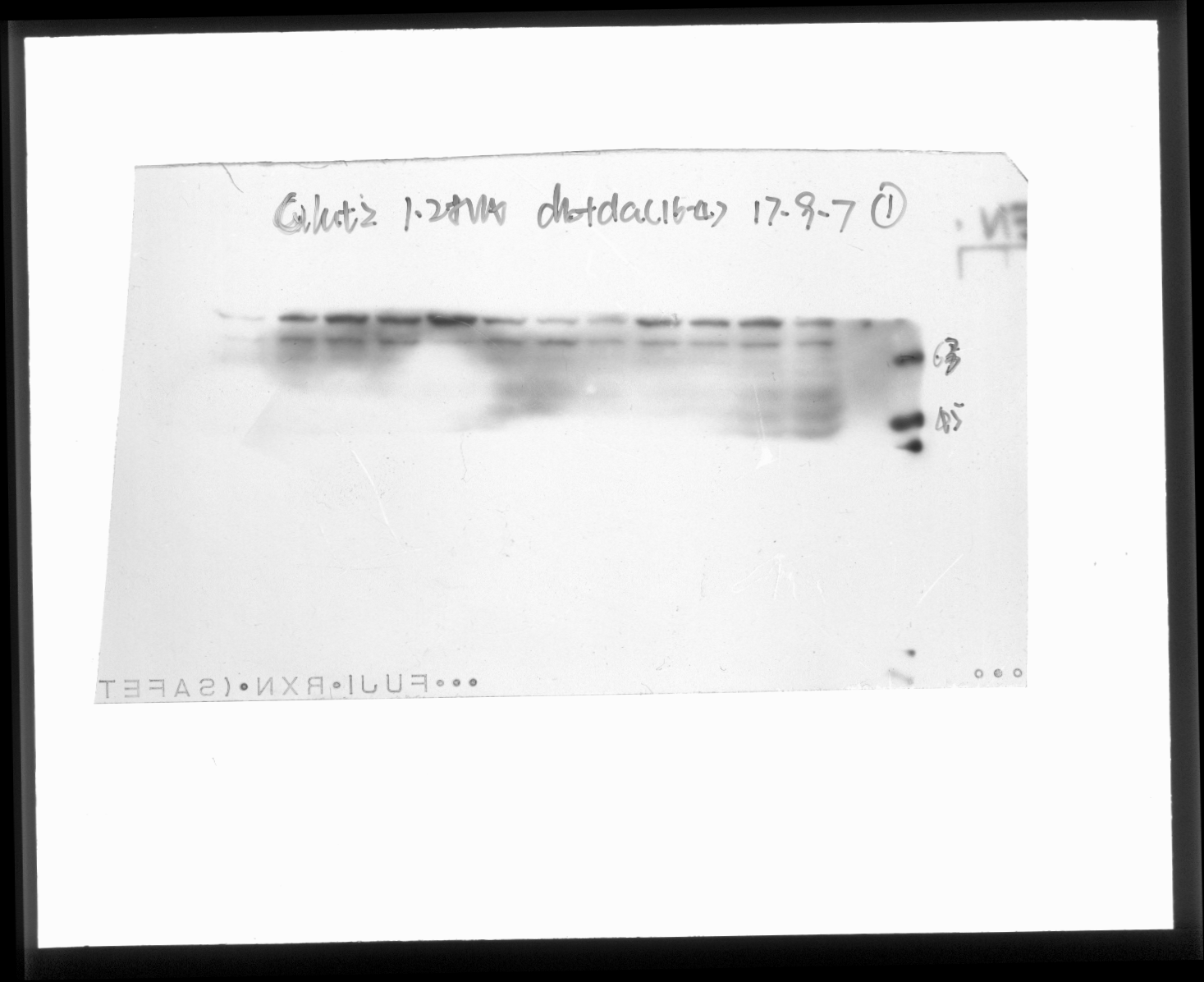

Supplement: Supplementary file 11 [file DataSheet5.ZIP › WB1/glut2 1批.tif]

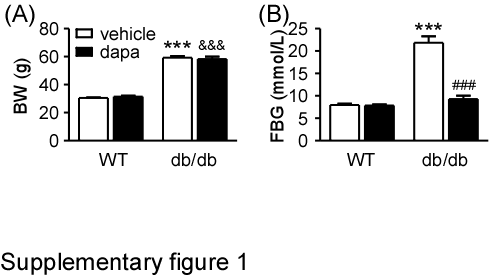

Supplement: Supplementary file 13 [file DataSheet1.docx]
