# Supplementary material for: Dapagliflozin protects against nonalcoholic steatohepatitis in db/db mice
Source: Front Pharmacol. 2022 Aug 19;13:934136. doi: 10.3389/fphar.2022.934136 (PMC9437261; doi:10.3389/fphar.2022.934136)
Supplement: Supplementary file 9 [file DataSheet12.ZIP › 原图/新建 Microsoft Office Word 文档.docx]

将电镜图片，直接导入canvas（宽2.100，高1.400），剪切为（宽1.613，高0.968），剪切掉标尺。
